# The MUC5B-associated variant rs35705950 resides within an enhancer subject to lineage- and disease-dependent epigenetic remodeling

**DOI:** 10.1172/jci.insight.144294

**Published:** 2021-01-25

**Authors:** Fabienne Gally, Sarah K. Sasse, Jonathan S. Kurche, Margaret A. Gruca, Jonathan H. Cardwell, Tsukasa Okamoto, Hong W. Chu, Xiaomeng Hou, Olivier B. Poirion, Justin Buchanan, Sebastian Preissl, Bing Ren, Sean P. Colgan, Robin D. Dowell, Ivana V. Yang, David A. Schwartz, Anthony N. Gerber

**Affiliations:** 1 Department of Immunology and Genomic Medicine, National Jewish Health, Denver, Colorado, USA.; 2 Department of Medicine, University of Colorado, Aurora, Colorado, USA.; 3 Department of Medicine, National Jewish Health, Denver, Colorado, USA.; 4 BioFrontiers Institute, University of Colorado-Boulder (CU Boulder), Boulder, Colorado, USA.; 5 Department of Respiratory Medicine, Tokyo Medical and Dental University, Tokyo, Japan.; 6 Center for Epigenomics, Department of Cellular and Molecular Medicine, University of California, San Diego School of Medicine, La Jolla, California, USA.; 7 Ludwig Institute for Cancer Research, La Jolla, California, USA.; 8 Molecular, Cellular and Developmental Biology, and; 9 Computer Science, CU Boulder, Boulder, Colorado, USA.

**Keywords:** Pulmonology, Fibrosis, Genetic variation

## Abstract

The G/T transversion rs35705950, located approximately 3 kb upstream of the *MUC5B* start site, is the cardinal risk factor for idiopathic pulmonary fibrosis (IPF). Here, we investigate the function and chromatin structure of this –3 kb region and provide evidence that it functions as a classically defined enhancer subject to epigenetic programming. We use nascent transcript analysis to show that RNA polymerase II loads within 10 bp of the G/T transversion site, definitively establishing enhancer function for the region. By integrating Assay for Transposase-Accessible Chromatin using sequencing (ATAC-seq) analysis of fresh and cultured human airway epithelial cells with nuclease sensitivity data, we demonstrate that this region is in accessible chromatin that affects the expression of *MUC5B*. Through applying paired single-nucleus RNA- and ATAC-seq to frozen tissue from IPF lungs, we extend these findings directly to disease, with results indicating that epigenetic programming of the –3 kb enhancer in IPF occurs in both *MUC5B*-expressing and nonexpressing lineages. In aggregate, our results indicate that the *MUC5B*-associated variant rs35705950 resides within an enhancer that is subject to epigenetic remodeling and contributes to pathologic misexpression in IPF.

## Introduction

Idiopathic pulmonary fibrosis (IPF) affects 5 million people worldwide and is associated with a poor prognosis (1). MUC5B, a gel-forming mucin normally secreted by submucosal glands and involved in mucociliary clearance and innate immunity (2, 3), has been implicated in the pathogenesis of IPF. Multiple independent genetic studies have consistently identified the gain-of-function *MUC5B* promoter variant rs35705950, which is a G/T transversion, as the dominant risk factor for developing IPF (4–8); the minor T allele is the disease-associated genotype. Moreover, MUC5B overexpression and mislocalization to terminal airways and honeycomb cysts are commonly observed in IPF and are associated with the *MUC5B* promoter variant (9, 10), and transgenic *Muc5b* mice have been shown to be more susceptible to the fibroproliferative effects of bleomycin (11).

Given the relevance of MUC5B expression to IPF, a number of studies have investigated MUC5B regulation and the function of the region approximately 3 kb upstream of the *MUC5B* start site that harbors the SNP (the –3 kb region). Transcription factors have been reported to directly bind in the region of the *MUC5B* variant (12), increase *MUC5B* expression (12–15), and affect goblet cell differentiation (16). In addition, the transcription factor XBP1, a mediator of endoplasmic reticulum (ER) stress, binds directly to the –3 kb regulatory region and affects expression of *MUC5B* in a genotype-dependent manner (17). We have characterized a functional interaction between FOXA2 and a highly conserved binding site located within 32 bp of the variant site (15), and we have observed methylation of this region associated with IPF, MUC5B expression, and the *MUC5B* promoter variant (15). Histone acetylation has also been implicated in regulating MUC5B (18, 19), suggesting a dynamic role for epigenetic modifications in controlling MUC5B expression. However, despite this growing understanding of MUC5B regulation and epigenetics, a comprehensive mechanistic understanding of MUC5B regulation and heterogeneous expression in association with IPF remains elusive.

In this study, therefore, we set out to determine whether specific transcription factors confer enhancer activity to the *MUC5B* –3 kb region and whether the underlying chromatin structure of this region and the rs35705950 variant might contribute to regulatory control. Our results form the basis for a model in which epigenetic control of the –3 kb enhancer promotes pathologic misexpression of MUC5B in IPF, especially in the presence of the *MUC5B* promoter variant.

## Results

### The region surrounding the MUC5B SNP has enhancer activity that is modulated by STAT3 and SPDEF.

**Figure 1:**
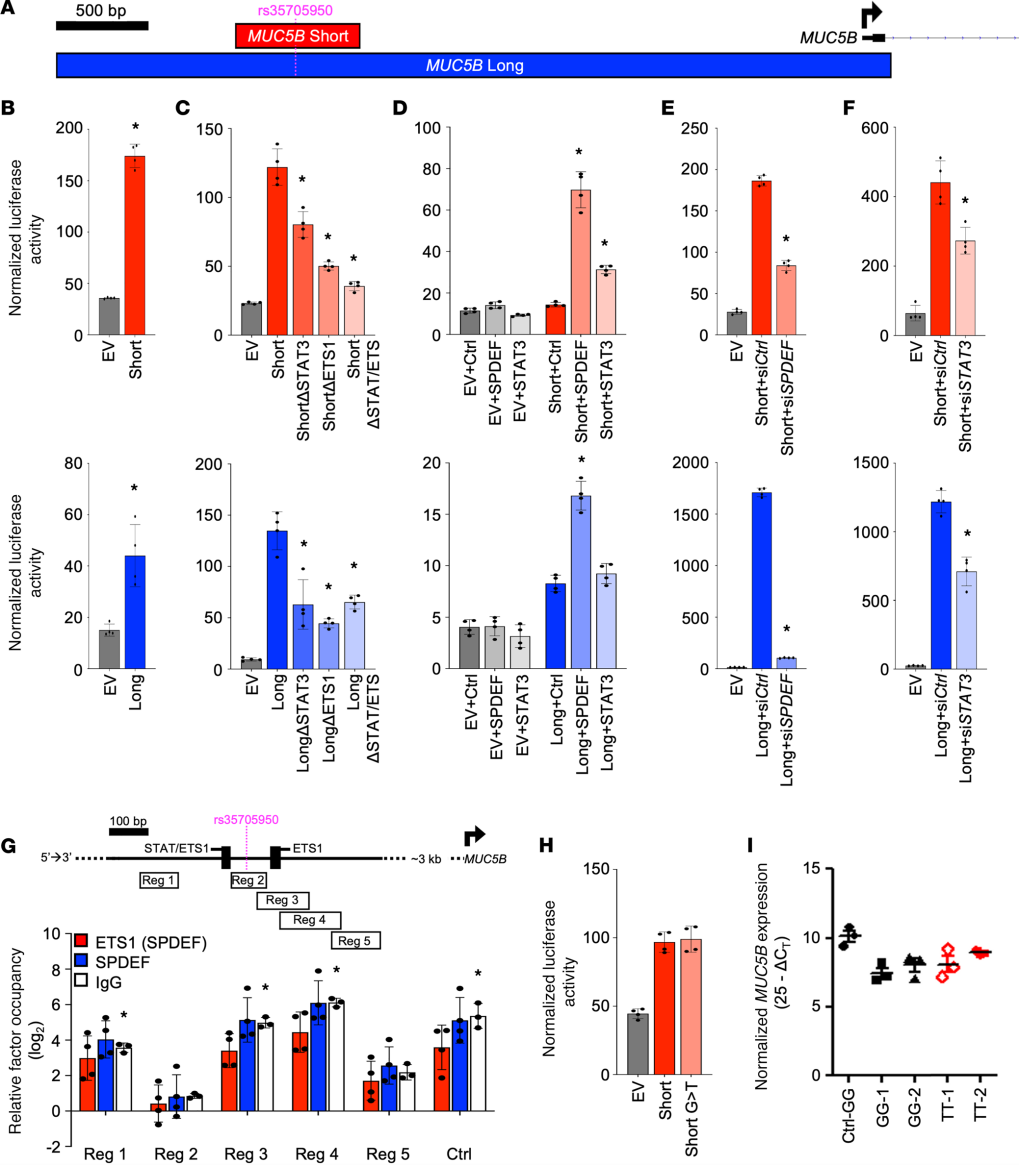
The *MUC5B* –3 kb region exhibits enhancer activity that is regulated by STAT3 and SPDEF. (**A**) Location of *MUC5B* Short (red, 766 bp) and Long (blue, 4532 bp) regions cloned into reporter constructs relative to the transcription start site (black arrow). (**B**–**F**) Mean (±SD) normalized luciferase activity of indicated Short (red; top) and Long (blue; bottom) *MUC5B* reporter constructs and respective empty vector (EV) control in A549 cells. (**B**) WT constructs under basal culture conditions (*n* = 4/group, *P ≤ 0.05 versus EV via *t* test). (**C**) WT versus mutant constructs with binding site mutations for STAT3, ETS1, or both (*n* = 4/group, *P ≤ 0.0001 versus WT via 1-way ANOVA with Bonferroni correction). (**D**) WT constructs cotransfected with SPDEF or STAT3 cDNA expression constructs or empty expression vector control (Ctrl; *n* = 4/group, *P ≤ 0.003 versus WT + Ctrl via 1-way ANOVA with Bonferroni correction). (**E** and **F**) WT constructs cotransfected with siRNA targeting *SPDEF* (**E**), *STAT3* (**F**), or control (si*Ctrl*; *n* = 4/group, *P ≤ 0.001 versus WT + si*Ctrl* via 1-way ANOVA with Bonferroni correction). (**G**) ChIP-qPCR analysis of mean (±SD) relative occupancy of SPDEF and IgG across the *MUC5B* –3 kb region in A549 cells (*n* = 3 (IgG) or 4 (SPDEF) per group; *P ≤ 0.05 versus geometric mean of IgG occupancy at 3 negative control regions via *t* test. Schematic indicates locations of putative STAT/ETS1 binding sites and regions targeted by ChIP-qPCR primers. (**H**) Mean (±SD) normalized luciferase activity of WT and mutant *MUC5B* Short reporter constructs with G or variant T at the G/T transversion site, respectively (*n* = 4/group). (**I**) qPCR analysis quantifying relative *MUC5B* expression in untransfected WT control (Ctrl-GG) and CRISPR-Cas9–edited WT (GG) and variant (TT) A549 clones (*n* = 3/group). Data in each panel are representative of at least 3 independent experiments, except for **I**, which was performed in 2 independent clones for each genotype.

To establish that the *MUC5B* –3 kb region is an enhancer, we cloned approximately 750 bp around the G/T transversion into a luciferase reporter with a heterologous minimal promoter (Figure 1A; Short). We used cancer-derived A549 airway epithelial cells to interrogate the activity of this reporter in comparison with a construct spanning approximately 5 kb upstream of the *MUC5B* start site through the native promoter (Figure 1A; Long) (20). The Short fragment conferred transcriptional activity in the context of a minimal promoter, confirming that it is an enhancer (Figure 1B). Next, we used the MatInspector software tool to identify binding sites for candidate transcription factors to regulate *MUC5B* expression within the –3 kb region and uncovered putative STAT and ETS sites (5′ and 3′ of the SNP, respectively) within a distance of approximately 300 bp. The ETS family member, SPDEF, has been reported to regulate *MUC5B* expression through this region in A549 cells (12). To test whether STAT3 and/or SPDEF act through this region, we mutated the canonical binding sites for STAT and ETS family members that surround the SNP in the Short and Long constructs. As shown in Figure 1C, both single–binding site mutant constructs and the double STAT/ETS mutants exhibited reduced activity compared with the WT constructs. Moreover, cotransfection of cDNA expression constructs for SPDEF and STAT3 increased activity of both the Short and Long constructs (Figure 1D). In converse, siRNA-mediated knockdown of these factors reduced activity (Figure 1, E and F), with the effect of *SPDEF* knockdown particularly prominent. Taken together, these data indicate that the *MUC5B* –3 kb region functions as an enhancer and can be regulated by STAT3 and SPDEF.

To interrogate direct interactions between SPDEF and the *MUC5B* –3 kb region in A549 cells, we used ChIP–quantitative PCR (ChIP-qPCR). Whereas ChIP assays performed with 2 different antibodies appeared to indicate SPDEF occupancy at both putative binding sites, pulldown using a polyclonal IgG antibody control led to a similar degree of enrichment (Figure 1G). This suggests that the –3 kb region is hyper-ChIPable, a well-described phenomenon in which chromatin within certain genomic regions — typically in association with regulatory activity — interacts nonspecifically with many antibodies (21–23). These nonspecific interactions between IgG and the *MUC5B* enhancer, which we observed in a wide range of cell types (Supplemental Figure 1; supplemental material available online with this article; https://doi.org/10.1172/jci.insight.144294DS1), precluded conclusions about interactions between specific transcription factors and the –3 kb region.

We also tested whether the G/T transversion is sufficient to influence *MUC5B* expression in A549 cells. The Short enhancer construct, modified by site-directed mutagenesis (SDM) to have the variant T at the transversion site, displayed similar activity to the WT reporter in transfection assays (Figure 1H). Moreover, homozygous TT A549 cells derived through CRISPR-Cas9–targeted genome editing exhibited *MUC5B* expression levels that were similar to edited control clones with the WT GG genotype (Figure 1I). Taken together, although our data establish that the region surrounding the transversion site functions as an enhancer, the activity of the enhancer was not directly influenced by the G/T transversion in a cell-autonomous manner in the A549 model.

### Annotation of eRNA signatures localizes RNA polymerase II loading to the MUC5B –3 kb enhancer.

**Figure 2:**
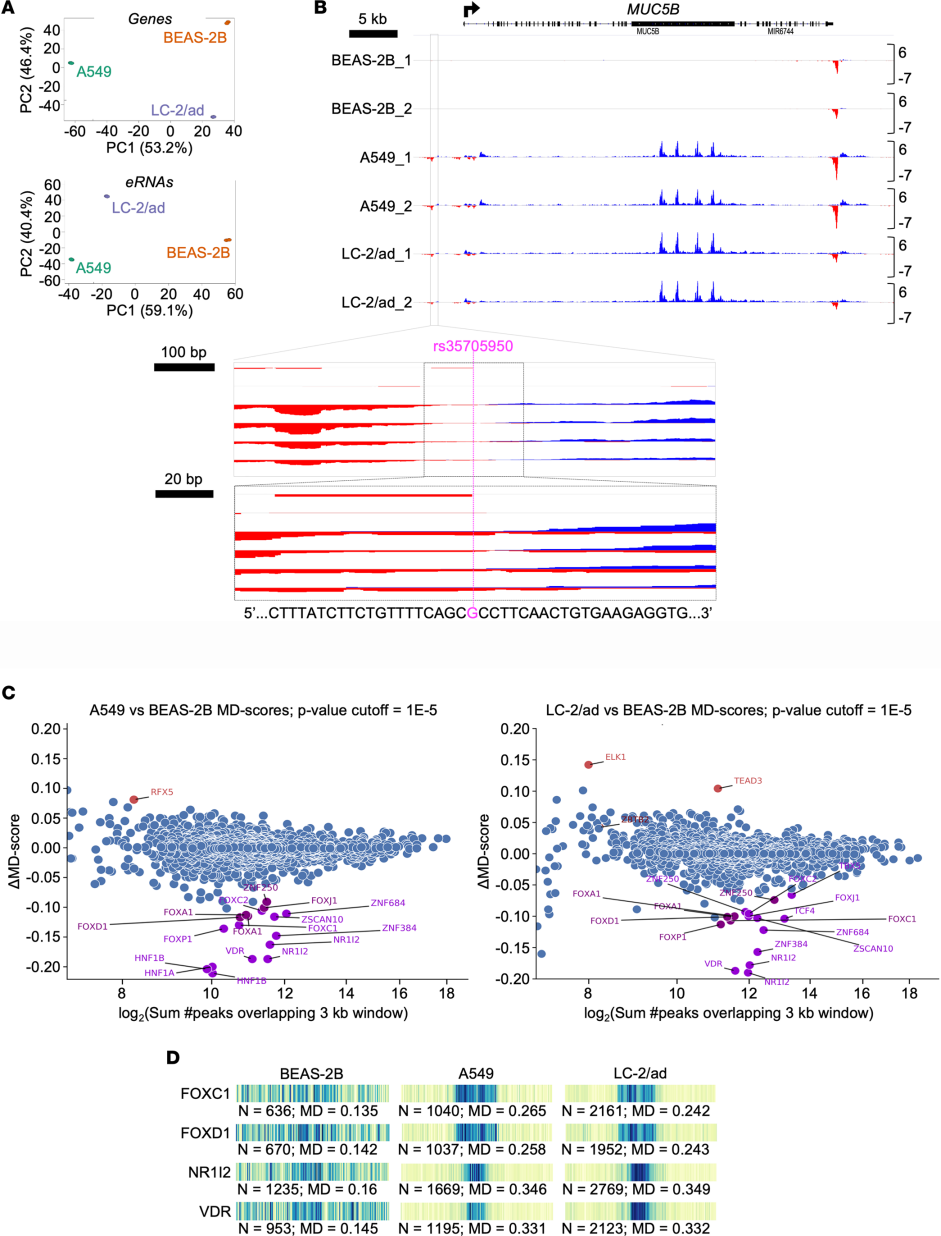
PRO-seq nascent transcript profiling identifies RNAPII loading at the *MUC5B* G/T transversion site in A549 and LC-2/ad cells and differential transcription factor activity compared with BEAS-2B cells. (**A**) Principal Component Analysis (PCA) of gene (top) and eRNA (bottom) transcription indicates significant separation by cell type. (**B**) PRO-seq data visualized in the Integrative Genomics Viewer (IGV) Genome Browser at the *MUC5B* locus in indicated cell types sequenced in duplicate (*n* = 2). Color signifies direction of RNAPII processivity (5′ to 3′, blue; 3′ to 5′, red), and vertical scales indicate counts per million mapped reads; arrow shows transcription start site and direction of transcription. Each lower panel is a progressively zoomed-in view of the *MUC5B* –3 kb enhancer centered on rs35705950 (purple) and its surrounding genomic sequence (bottom). (**C**) Motif Displacement (MD) analysis of binding motifs significantly enriched (red) or reduced (purple) in A549 (left) and LC-2/ad (right) cells relative to BEAS-2B. (**D**) Barcode plots depicting frequency of sequence overlap with indicated binding motifs within ± 1500 bp of eRNA origins in indicated cell types. Heat is proportional to frequency of motif instance at that distance from an eRNA origin; darker colors signify greater enrichment.

Discrete sites of functional interactions between RNA polymerase II (RNAPII) and enhancers, which are characterized by bidirectional transcription of enhancer RNAs (eRNAs) (24), can be mapped in high resolution by deep sequencing of nascent transcripts (25, 26). Thus, to determine the relationship between the *MUC5B* –3 kb region and RNAPII loading and activity, we performed nascent transcriptional sequencing using Precision Run-On sequencing (PRO-seq) (27) in A549 and LC-2/ad cells, which both express *MUC5B*, in comparison with BEAS-2B cells, which do not express *MUC5B* (Supplemental Figure 1). While the 3 cell types clustered independently based on both nascent gene and eRNA transcription on a genome-wide basis (Figure 2A and Supplemental Figure 2), the local pattern of RNAPII activity across the *MUC5B* locus was markedly similar in A549 and LC-2/ad cells (Figure 2B). RNAPII activity was detected across the *MUC5B* gene body, and bidirectional signatures were detected in the promoter, the 3′UTR and the –3 kb enhancer. In contrast, RNAPII activity was only detected in the 3′UTR of *MUC5B* in BEAS-2B cells, consistent with the lack of *MUC5B* expression in this cell type. Thus, PRO-seq identified sites of *MUC5B* regulatory activity and confirmed active transcription of the gene in A549 and LC-2/ad cells.

Seeking to exploit the near bp resolution of RNAPII activity that is afforded by PRO-seq, we analyzed the *MUC5B* –3 kb enhancer region in greater detail. Higher-resolution visualization showed that the site of RNAPII loading at this enhancer, defined as the center of the bidirectional signature, is located within 10 bp of rs35705950 (Figure 2B, lower panels). This finding establishes that the *MUC5B* variant abuts the primary functional element of this enhancer region (i.e., the site of RNAPII loading), strongly supporting a direct role for the variant in enhancer function.

Based on these data showing that the *MUC5B* enhancer is active in A549 and LC-2/ad cells, we applied a metric called Motif Displacement (MD) to determine enrichment for transcription factor binding motifs within all active enhancers on a genome-wide basis in the 3 cell types. MD calculates the proportion of transcription factor motifs that are within a 150 bp radius in comparison with a 1500 bp radius (28) — in this case, using sequences centered on active enhancers as defined using the Tfit tool (29). We compared motifs within all active enhancers defined in the 3 cell types, reasoning that motifs enriched in *MUC5B*-expressing versus nonexpressing cells may represent transcription factors that are candidates to regulate MUC5B expression, either directly or indirectly. As shown in Figure 2C, a number of motifs were identified with differential MD scores when comparing A549 with BEAS-2B cells (top) and LC-2/ad with BEAS-2B cells (bottom). Through intersecting these sets, we identified several potentially novel regulators of MUC5B (Figure 2D). These include FOXD1, a transcription factor that regulates IPF-associated genes such as galectin-3 (30), as well as 2 nuclear receptors, the pregnane X receptor and the vitamin D receptor, both of which can bind DNA as heterodimers with retinoic acid receptors (31–34), which are strongly implicated in mucin gene regulation (35, 36). The MatInspector software tool identified 2 consensus vitamin D receptor/retinoid X receptor (VDR/RXR) heterodimer binding sites within the –3 kb enhancer region. A comprehensive list of MD scores and additional comparisons are provided in Supplemental Data Files 1 and 2.

### Chromatin accessibility of the MUC5B variant region.

**Figure 3:**
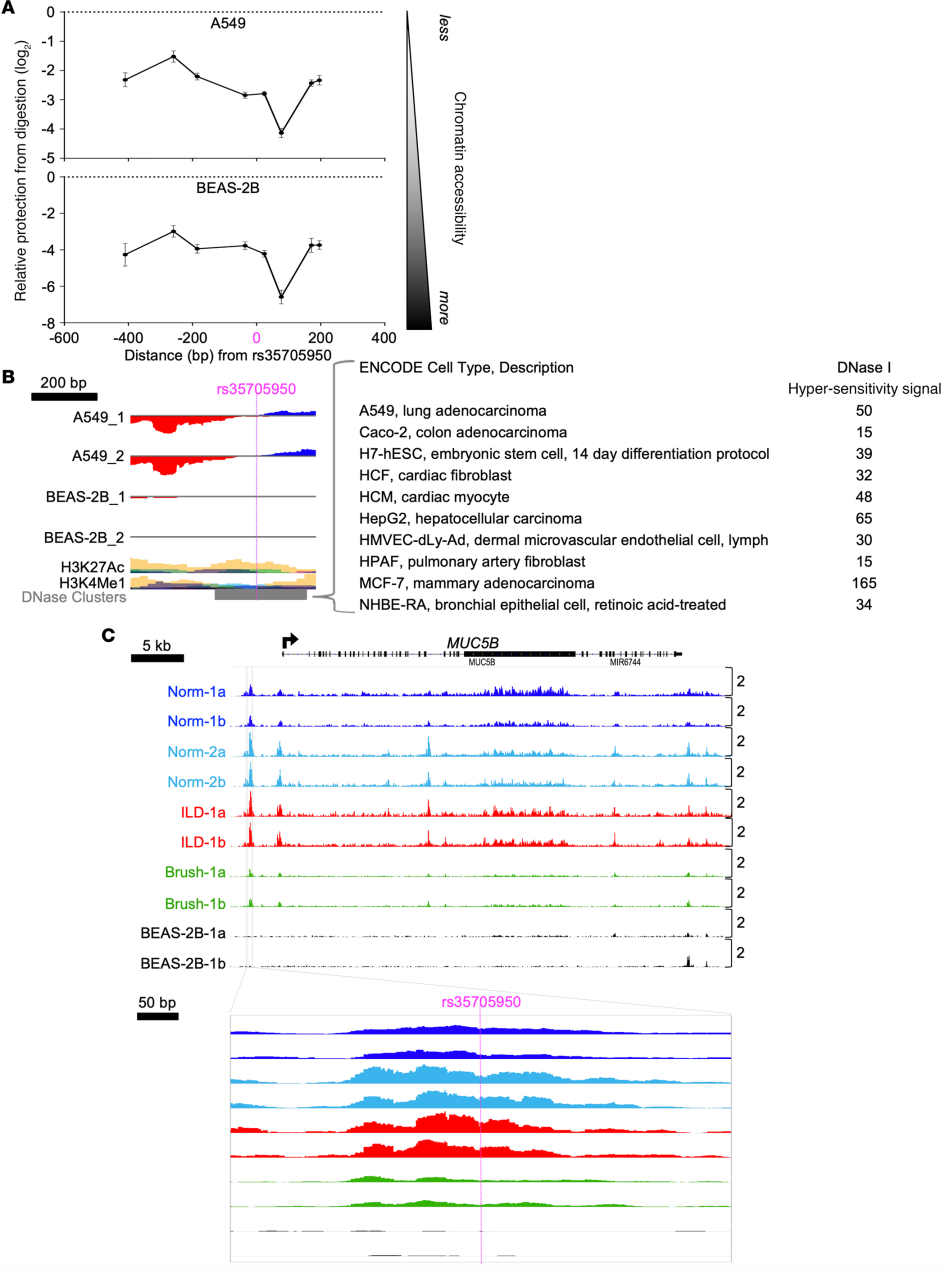
The *MUC5B* variant resides in an internucleosomal chromatin domain that is accessible to nuclease digestion in a range of cell lineages. (**A**) MNase-qPCR assay of mean (±SD) relative protection from MNase cleavage across the *MUC5B* –3 kb enhancer region in A549 (top) and BEAS-2B (bottom) cells, with less protection corresponding to a more accessible chromatin structure (*n* = 4/group; data are representative of 3 independent experiments). (**B**) UCSC Genome Browser visualization of A549 and BEAS-2B PRO-seq data aligned to scale with the region interrogated by MNase-qPCR in **A** and genome-wide DNase I accessibility data from the ENCODE consortium (gray track) representing 14 tested cell lines, a selection of which are listed to the right. (**C**) Normalized (counts per million mapped reads) ATAC-seq signatures at the *MUC5B* locus (top) and –3 kb enhancer (bottom) in primary airway epithelial cells cultured at ALI (Norm; blue, ILD; red), freshly brushed airway epithelial cells (Brush; green), and BEAS-2B cells (black), each sequenced in duplicate (*n* = 2).

Sites of RNAPII loading and enhancer activity are frequently within regions of open chromatin that are flanked by nucleosomes. Therefore, to determine nucleosome positioning in relationship to the –3 kb enhancer region, we performed micrococcal nuclease (MNase) accessibility assays (37). Using tiled qPCR, we imputed relative protection from MNase digestion across approximately 600 bp surrounding the *MUC5B* SNP in A549 cells. The results indicate a relatively unprotected region proximal to the SNP that was highly susceptible to MNase digestion (Figure 3A, top). This accessible, SNP-proximal region was flanked by areas of relative protection, most notably upstream, consistent with positioned nucleosomes on either side of the SNP. Relative protection conferred by these presumptive nucleosomes was similar to that observed in a control region (Supplemental Figure 3). An area of relative protection 5′ of the variant, consistent with a positioned nucleosome, was also observed in BEAS-2B cells (Figure 3A, bottom). Aligning these data with the ENCODE consortium DNase-seq track on the UCSC Genome Browser (https://genome.ucsc.edu/) revealed that the SNP-proximal region was also sensitive to DNase I digestion in 14 tested cell lines (Figure 3B), including *MUC5B*-expressing cell types such as A549, Caco-2, and HepG2, as well as several cell types representing lineages that do not express *MUC5B* (e.g., human cardiac fibroblasts and myocytes). Thus, the *MUC5B* variant resides in an internucleosomal chromatin domain that is accessible to nuclease digestion in a range of cell lineages.

To determine whether the *MUC5B* –3 kb RNAPII loading site resides in accessible chromatin in more physiologically relevant models of human airway epithelium, we cultured primary airway epithelial cells derived from patients with interstitial lung disease (ILD; *n* = 3) and normal controls (Norm; *n* = 3) at air-liquid interface (ALI). All ALI cultures expressed *MUC5B* under basal culture conditions and exhibited hyper-ChIPable chromatin within the *MUC5B* –3 kb enhancer region (Supplemental Figure 4). We applied the Assay for Transposase-Accessible Chromatin using sequencing (ATAC-seq) to determine chromatin accessibility in 3 of these ALI cultures (*n* = 2 Norm, 1 ILD; each in technical duplicate). Visualization of ATAC-seq data at the *MUC5B* locus revealed a clear peak at the –3 kb enhancer region in all ALI samples (Figure 3C), confirming accessible chromatin surrounding the variant site. The 5′ tail of the ATAC-seq peak precisely overlapped the site of maximal protection from MNase digestion, further suggesting nucleosome positioning at this chromatin juncture. Congruent ATAC-seq results were obtained from a freshly brushed human airway epithelial cell sample (GG at the *MUC5B* variant site) sequenced in duplicate (Figure 3C, green tracks), establishing similarities between in vivo chromatin architecture and our in vitro findings. The enhancer, however, was not accessible to transposase in BEAS-2B cells (Figure 3C, black tracks), suggesting that internucleosomal chromatin remodeling can occur in association with *MUC5B* expression.

### Integrated single-nucleus analysis of MUC5B mRNA expression and chromatin accessibility in IPF lung tissue.

**Figure 4:**
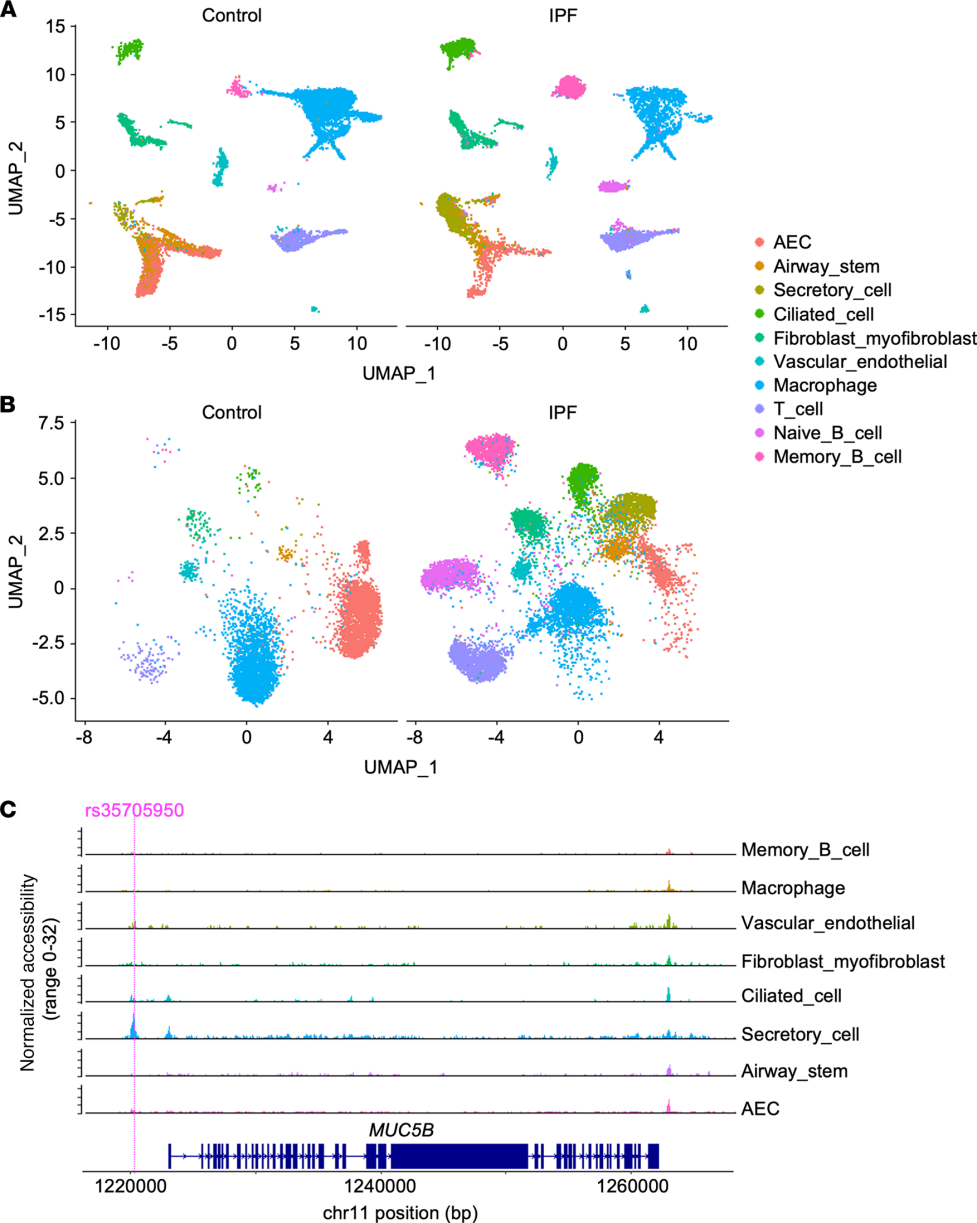
Integrated single-nucleus sequencing reveals promiscuous chromatin accessibility at the –3 kb transversion site in lung tissue from IPF patients. (**A**) Cell types identified by snRNA-seq in lung tissue from IPF patients and unaffected controls using UMAP-based cell clustering. (**B**) snATAC-seq cell clustering in IPF and control lungs using gene expression information from paired snRNA-seq to define cell types. (**C**) snATAC-seq data aggregated by cell type and visualized as chromatograms at the *MUC5B* locus. AEC, alveolar epithelial cell.

Variable *MUC5B* expression has previously been reported in single cell RNA-seq data from IPF-derived samples (38, 39). Therefore, to determine whether the *MUC5B* enhancer is in open chromatin in patient samples and whether *MUC5B* chromatin architecture can be decoupled from the secretory cell fate in IPF, we performed paired single-nucleus RNA-seq (snRNA-seq) and single-nucleus ATAC-seq (snATAC-seq) in lung tissue samples obtained from 2 patients with IPF (*n* = 1 GG and *n* = 1 TT genotype — i.e., homozygote for the risk allele) at rs35705950. Two unaffected controls were also studied (*n* = 1 GG, 1 TT genotype at rs35705950). Available clinical details with respect to these patient samples are in Supplemental Table 1. Clustering approaches applied to the snRNA-seq data sets successfully demarcated major lung cell types (Figure 4A). Although all samples were adequate for snRNA-seq, the control TT sample did not meet quality criteria for snATAC-seq after 2 attempts and was excluded from further analysis. The remaining snATAC-seq data sets were spatially distributed based on cell type assigned through the paired snRNA-seq (Figure 4B), which demonstrated substantial overlap between chromatin features and cell type as assigned by gene expression. To determine whether specific cell types exhibited accessible chromatin at the *MUC5B* enhancer, we aggregated the snATAC-seq data as a function of cell type and visualized the output as chromatograms (Figure 4C). Secretory cells showed an ATAC-seq peak aligned with the –3 kb enhancer, indicative of chromatin accessibility in vivo in this region. A peak in this location was evident in several additional cell types, including vascular endothelial cells and ciliated cells, whereas a range of other lineages did not exhibit accessible chromatin in this region. Thus, the –3 kb enhancer can undergo chromatin remodeling to adopt an open conformation in several pulmonary lineages, including those not typically associated with *MUC5B* expression.

**Figure 5:**
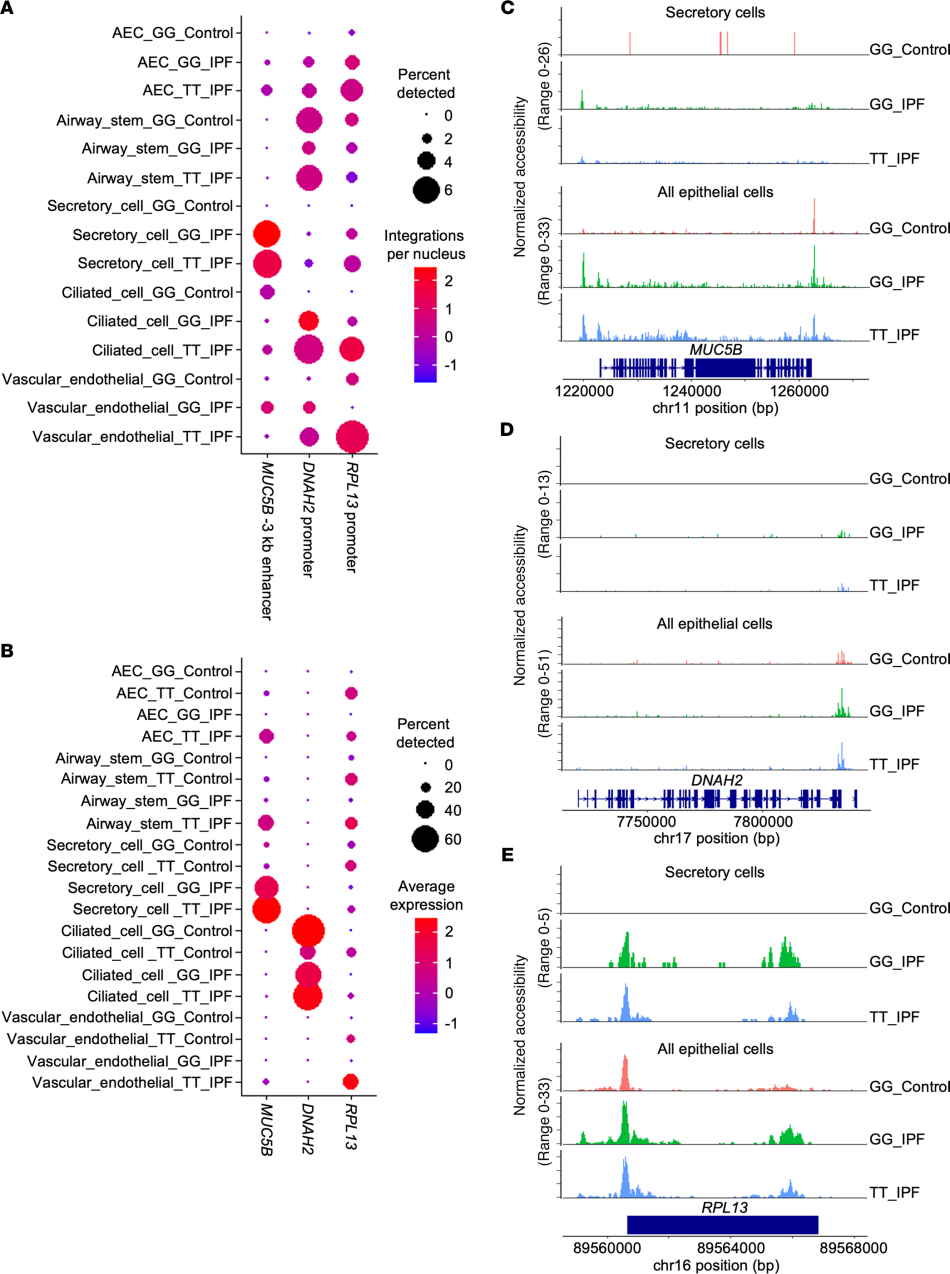
Single-nucleus analysis indicates in vivo decoupling of *MUC5B* enhancer chromatin architecture from cell type, rs35705950 genotype, and *MUC5B* gene expression. (**A**) Dot plot of normalized count data from snATAC-seq. Dot size represents frequency of any counts at the indicated region (*x* axis) across listed cell types (*y* axis); color signifies average number of integrations per nucleus, normalized to the total number of unique molecular identifiers per nucleus. (**B**) Dot plot of normalized count data from snRNA-seq. Dot size represents frequency of any counts of the listed gene (*x* axis) across listed cell types (*y* axis); color indicates average number of reads per nucleus. For both **A** and **B**, data are shown as indicated for *MUC5B*, along with 2 control loci, *DNAH2* and *RPL13*, which exhibit varied patterns of chromatin accessibilty and RNA expression relative to cell type, disease state, and genotype. (**C**–**E**) Chromatograms depicting aggregated normalized snATAC-seq data as a function of genotype and disease status for secretory cells (top) or all airway epithelial cell types (bottom) at the *MUC5B* (**C**), the *DNAH2* (**D**), and *RPL13* (**E**) loci.

We next investigated chromatin features at the MUC5B enhancer and MUC5B expression relative to disease status, genotype, and cell type (Figure 5, A and B). As depicted in Figure 5A, the snATAC-seq signal was observed at the –3 kb enhancer in secretory cells derived from IPF samples but not in controls, an effect that was inflated by the relative infrequency of secretory cells captured from control lung tissue (Figure 4A). In contrast, minimal accessibility was evident in control or IPF secretory cells at unrelated loci such as *DNAH2*, whereas *RPL13* exhibited modest accessibility across cell types. While there was a trend toward open *MUC5B* enhancer chromatin in nonsecretory lineages in IPF samples relative to control (Figure 5A), these results were not significant. When all epithelial lineages were aggregated (Figure 5C), however, there was a disease-dependent increase in accessibility at the *MUC5B* promoter (fold change for *MUC5B* chromatin accessibility in IPF versus control epithelial cells = 1.52, Bonferroni adjusted *P* value (*P*_adj_) = 7.1 × 10^–44^, based on logistic regression). Accessibility at unrelated genomic regions (e.g., *DNAH2* and *RPL13*) in aggregated epithelial cells was similar in control and IPF samples (Figure 5, D and E). Paired snRNA-seq also revealed increased *MUC5B* expression in epithelial cells clustered from IPF patients; this effect disappeared when secretory cells were removed from the analysis (Figure 5B; average secretory cell *MUC5B* expression fold change for IPF versus control = 1.91, *P*_adj_ = 4.9 × 10^–41^, based on negative binomial regression). The complete single nucleus data set including expression and accessibility data for *MUC5B*, *DNAH2*, and *RPL13* is represented by violin plots in Supplemental Figure 5.

To examine the role of genotype at rs35705950 on *MUC5B* chromatin accessibility, we focused our comparisons on cells derived from IPF lungs stratified by genotype. We found significantly increased *MUC5B* chromatin accessibility in variant-derived epithelial cells (fold change for *MUC5B* –3 kb enhancer chromatin accessibility in variant versus WT epithelial cells = 1.31, *P*_adj_ = 1.57 × 10^–12^, based on logistic regression). This difference persisted when secretory cells were removed from the analysis (fold change for *MUC5B* chromatin accessibility in variant versus WT nonsecretory cells = 1.34, *P*_adj_ = 1.08 × 10^–5^) and was not significant for the secretory cell comparison across genotypes. This difference in chromatin accessibility across genotypes was coupled to expression differences in secretory and nonsecretory cells (fold change for *MUC5B* expression in variant versus WT secretory cells = 1.36, *P*_adj_ = 3.1 × 10^–14^, based on negative binomial regression; fold change for *MUC5B* expression in variant versus WT nonsecretory cells = 1.32, *P*_adj_ = 4.2 × 10^–31^, based on negative binomial regression). Collectively, these data indicate that the rs35705950 *MUC5B* variant is associated with chromatin accessibility in nonsecretory cell types, supporting a role for epigenetic regulation of ectopic MUC5B expression in IPF.

## Discussion

**Figure 6:**
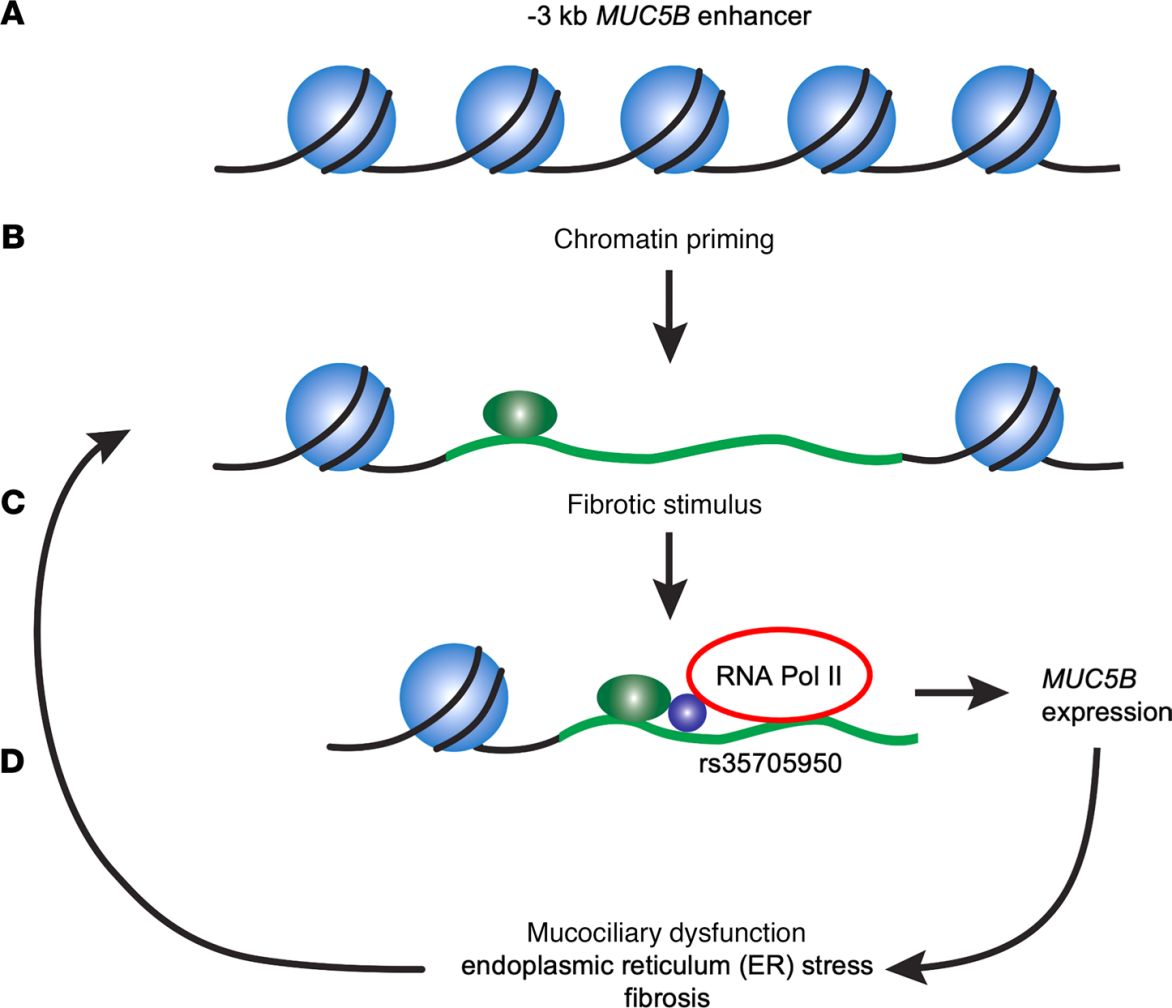
Model of epigenetic priming and positive feedback. (**A**) Basal positioned nucleosomal packaging of the *MUC5B* –3 kb enhancer. (**B**) In response to pleiotropic stimuli, chromatin remodeling occurs, priming enhancer DNA for interactions with transcription factors. (**C**) In the setting of fibrotic lung disease, the enhancer is activated by a range of transcription factors acting through semidegenerate binding sites for STAT, ETS, and Forkhead box family members, among others, leading to recruitment of RNAPII and induction of *MUC5B* expression. (**D**) *MUC5B* expression, in turn, promotes endoplasmic reticulum stress and mucociliary dysfunction, leading to additional activation of *MUC5B* in adjacent cells, thus comprising a positive feedback circuit.

Our results indicate that the *MUC5B*-associated variant rs35705950 resides within an enhancer that is subject to epigenetic remodeling and contributes to pathologic misexpression in IPF. Our findings, taken in the context of other reports on MUC5B regulation (13, 17), support a model (Figure 6) in which open chromatin structure allows stochastic access of diverse transcription factors to the *MUC5B* –3 kb enhancer, promoting variable levels of pathologic MUC5B misexpression in a process that appears to be further derepressed by the rs35705950 variant in the context of disease.

Inaccessible chromatin exerts constitutive repressive effects on transcription through inhibiting promiscuous interactions between transcription factors and stochastically occurring binding sequences (40). Consequently, as a mechanism to limit aberrant expression, regulatory elements for tissue-specific genes typically exhibit lineage-restricted open chromatin architecture that is controlled by the activity of classically defined master regulators, such as MyoD (41, 42). Viewed in this context, the DNase and MNase accessibility at the *MUC5B* enhancer in non–*MUC5B*-expressing cells types (e.g., in vitro data for embryonic stem cells, cardiac cell types, and BEAS-2B cells and in vivo data for vascular endothelial cells) is relevant to IPF-associated MUC5B expression in terminal bronchiolar-like regions, which do not normally express MUC5B (43). Indeed, many of the transcription factors reported to regulate MUC5B expression, such as XBP1, FOXA2, FOXM1, and STAT family members, are broadly expressed (44–46), indicating they lack the specificity to serve alone as master regulators of *MUC5B* chromatin programming and expression. Moreover, although SPDEF has been implicated as a master transcriptional regulator for differentiation of mucin-producing cells (16, 47), normal developmental control of *MUC5B* transcription via SPDEF is entirely dispensable for MUC5B expression in murine models of airway injury (48). Recent studies have also indicated that MUC5B expression in IPF does not require SPDEF coexpression (49). In aggregate, these findings support a model in which epigenetic and transcriptional programming of *MUC5B* in disease is decoupled from normal developmental processes, facilitating stochastic dysregulation in response to cell injury and stress. In this model, if aberrant MUC5B expression promotes additional injury or ER stress, a positive feedback circuit would be formed that would further amplify aberrant MUC5B expression (Figure 6) and potentially recruit additional factors that locally remodel *MUC5B* enhancer chromatin and/or transcribe *MUC5B* in a paracrine process.

We used several experimental systems and approaches to assay the chromatin structure of the *MUC5B* enhancer, and the data we generated were concordant across the methods. However, the small number of patient samples we analyzed using paired snRNA-seq and snATAC-seq is a clear limitation of our study. Definitive determination of the relationship between cell type, chromatin architecture of the *MUC5B* locus, and *MUC5B* expression in IPF will require analysis of additional patient samples encompassing a range of disease stages. Whether the increased variability in snATAC-seq signatures we observed on a genome-wide basis in the IPF samples (Figure 4) reflects general dysregulation of chromatin structure in IPF remains to be determined. We further note that our application of unbiased bioinformatics methodology (e.g., MD) to identify candidate regulators of MUC5B was limited to analysis of 3 cell lines with diverse transcriptomes. Expanding such approaches to encompass additional samples, including primary lung tissue, would increase their power and specificity for identifying transcriptional regulators of MUC5B.

What is the function of the G/T transversion? Although our data do not define a genotype-specific molecular interaction with the enhancer, our work does provide insight into stochastic properties of MUC5B regulation in the context of IPF. Indeed, irrespective of genotype, chromatin at this region is accessible to enzyme-based digestion (i.e., open) in alignment with clinical observations in which MUC5B expression is elevated in GG, GT, and TT IPF patients, although patients harboring the variant T tend to have the highest expression levels (15). Thus, rather than serving as a digital molecular switch, our data support a model in which the transversion stochastically biases further MUC5B misexpression through 2 possible, nonmutually exclusive mechanisms. One possibility is that the transversion creates a de novo binding site for a specific transcription factor that is able to interact with the site by virtue of the internucleosomal chromatin structure, potentially promoting further epigenetic remodeling of this region. A second possibility is that the transversion eliminates a binding site for a transcriptional repressor. In that regard, MatInspector analysis identified a putative binding site for the transcriptional repressor GCF in the context of the G allele that is not present in the sequence containing the minor variant T. Future studies are needed to definitively determine the protein complexes that interact with the region harboring the transversion and whether there are genotype-specific differences in protein-enhancer interactions.

Pharmacologic manipulation of chromatin structure and function is of emerging therapeutic importance in a range of human diseases (50–52), and there is a growing body of literature implicating epigenetic mechanisms as relevant to IPF pathogenesis (53–56). Moreover, several studies using preclinical models have suggested that targeting chromatin remodeling and its functional consequences may have therapeutic benefit in fibrotic lung disease (57–60). Our findings here provide a potential avenue for chromatin-based therapies in which *MUC5B* enhancer chromatin architecture serves as a target to block MUC5B misexpression. With general consensus emerging that IPF progression involves a multicomponent positive feedback circuit (61), combinatorial disruption of key pathologic nodes — including epigenetic control of promiscuous MUC5B expression — may ultimately be required to halt progression of this devastating disease.

## Methods

### Cell culture, fresh bronchial brush, and patient tissue samples.

A549 human lung epithelial adenocarcinoma cells (ATCC) were cultured in DMEM (Corning) with l-glutamine and 4.5 g/L glucose supplemented with 5% FBS (VWR) and 1% penicillin/streptomycin (pen/strep; Corning). LC-2/ad human lung epithelial adenocarcinoma cells were grown in a 1:1 mixture of RPMI-1640 (Corning) and Ham’s F12 medium with l-glutamine (Corning) supplemented with 10% FBS and 1% pen/strep. BEAS-2B transformed normal human airway epithelial cells (ATCC) were grown in DMEM containing 10% FBS and 1% pen/strep. Primary human airway smooth muscle (HASM) cells (provided by Reynold Panettieri, Rutgers Biomedical Health Sciences, Piscataway, New Jersey, USA) were cultured in Ham’s F12 with l-glutamine supplemented with 10% FBS and 1% pen/strep. THP-1 human monocyte-like cells (ATCC) were cultured in RPMI-1640 supplemented with 10% FBS, 0.05 mM β-mercaptoethanol, and 1% pen/strep. Primary human tracheobronchial epithelial cells were obtained from the Human Primary Cell Core at National Jewish Health. Cells were expanded in collagen-coated 6 cm tissue culture plates and cultured on collagen-coated 12 mm Transwell-Clear Polyester Inserts (0.4 μm pore size; Corning) as described (62). Cells were cultured at ALI for a minimum of 14 days before processing. All cells were maintained in 5% CO_2_ at 37°C. Bronchial brushings obtained during consented bronchoscopy at National Jewish Health were placed in PBS on ice prior to processing for ATAC-seq. The purity of epithelial cells is consistently more than 97% based on immunostaining of keratin 19 (nonbasal cells) and keratin 5 (basal cells). Genotyping at rs35705950 was performed for all cells using Taqman SNP Genotyping Assay ID C158225420 and TaqMan Genotyping Master Mix as instructed by the manufacturer (Applied Biosystems). Lung tissue for single-nucleus analysis was obtained from patients undergoing biopsy for multiple indications (nodules, structural malformations, recurrent infections) or candidate lungs for transplantation, which did not meet criteria for implant from the University of Pittsburgh and the Lung Tissue Research Consortium, according to IRB policy at the sponsoring institutions. Patients were consented for genetic studies. Lung tissues were flash frozen directly from buffered medium and stored at –80°C prior to use.

### Plasmids, transfection, and reporter assays.

The 675 bp *MUC5B* Short reporter construct was amplified from genomic DNA by PCR, cloned into pCR2.1-TOPO (Invitrogen), and subsequently ligated into the pGL3-Promoter vector (Invitrogen) using KpnI/XhoI. The 4532 bp *MUC5B* Long reporter construct cloned into the pGL4.10 vector backbone (Promega) has been described (20). *MUC5B* Short and Long reporter constructs with mutated STAT3 and/or ETS1/SPDEF binding sites and the *MUC5B* Short G > T transversion construct were generated using the QuikChange II SDM Kit from Agilent Technologies as instructed by the manufacturer. PCR primer sequences used for cloning and SDM are shown in Supplemental Table 2. The STAT/ETS double-mutant construct was generated using the ETS1 SDM primers with the STAT3 SDM construct as a template.

Standard plasmid transfection and cotransfection for overexpression/knockdown studies were performed in A549 cells, and luciferase activity was assayed as described (63). Luciferase activity was normalized to that of a *Renilla* luciferase internal control (pSV40-RL; Promega). Each experiment was performed in biologic quadruplicate and repeated at least twice with qualitatively similar results. Expression constructs for SPDEF (pcDNA-SPDEF) and STAT3 (pcDNA-STAT3) were obtained from GenScript and Addgene, respectively; pcDNA3.1(+) empty vector control was purchased from Invitrogen. Small interfering RNA (siRNA) ON-TARGETplus SMARTpool constructs targeting SPDEF (si*SPDEF*) or STAT3 (si*STAT3*) and the nontargeting control (si*Ctrl*) were supplied by Dharmacon.

### ChIP-qPCR.

ChIP was performed as reported previously (63), using the fixation and sonication conditions optimized for each cell type (Supplemental Table 3). DNA was immunoprecipitated using 5 μg of the following antibodies: anti-ETS1 (catalog 39580; Active Motif; targets ETS family member SPDEF), anti-SPDEF (catalog MBS2518460; myBiosource), or rabbit polyclonal IgG (catalog 910801; BioLegend). Crosslink-reversed and purified ChIP DNA was analyzed by qPCR, with relative factor occupancy calculated as described (63). Sequences of primers used for ChIP-qPCR are shown in Supplemental Table 4.

### CRISPR-Cas9 targeted genome editing.

The pSpCas9(BB)-2A-GFP (PX458) plasmid was obtained from Addgene (catalog 48138) and its Cbh promoter replaced with CMV to create pCMV-px458-GFP. The sequence 5′-cagcG/Tccttcaactgtgaag-3′, with G/T representing the rs35705950 variant site, was cloned into pCMV-px458-GFP and cotransfected into A549 cells with a 200 bp ssDNA donor fragment using the Cell Line Nucleofector Kit T and Nucleofector 2b Device from Lonza. Genotype of each clone at rs35705950 was screened by a Taqman SNP Genotyping Assay and verified by Sanger DNA sequencing.

### RNA purification and qPCR.

RNA preparation and qPCR were performed as previously described with normalization to **RPL19** (63). Sequences of primers used for qPCR were MUC5B forward, 5′-CACATCCACCCTTCCAA-3′; MUC5B reverse, 5′-GGCTCATTGTCGTCTCTG-3′; RPL19 forward, 5′-ATCGATCGCCACATGTATCA-3′; RPL19 reverse, 5′-GCGTGCTTCCTTGGTCTTAG-3′.

### PRO-seq.

BEAS-2B, A549, and LC-2/ad cells were each plated on 2 × 15 cm tissue culture dishes and grown to confluence. Cells were harvested and nuclei prepared as described (22). Aliquots containing 10E6 nuclei in 100 μL Freezing Buffer (50 mM Tris-HCl [pH 8.3], 5 mM MgCl_2_, 40% glycerol, 0.1 mM EDTA [pH 8.0], 4 U/mL SUPERase-In) were flash frozen in a dry ice/ethanol bath and stored at –80°C. After briefly thawing on ice, 100 μL aliquots of 10E6 nuclei were added to 100 μL of Reaction Buffer (5 mM Tris-HCl [pH 8.0]; 2.5 mM MgCl_2_; 0.5 mM DTT; 150 mM KCl; 0.025 mM each of Biotin-11-CTP [PerkinElmer] and ribonucleoside CTP; 0.125 mM each of ribonucleoside ATP, ribonucleoside GTP, and ribonucleoside UTP; 1% Sarkosyl; 20 U SUPERase-In) preheated to 37°C and incubated for exactly 3 minutes at 37°C. PRO-seq was then performed in duplicate as described (27). Due to previously determined intrinsic cell type differences in basal transcriptional activity, each of the duplicate LC-2/ad libraries were built using 2 separate run-on reactions (or 20E6 nuclei) as input that were pooled at the first RNA pellet resuspension step. Uniquely indexed libraries were pooled and sequenced on an Illumina NextSeq instrument using 75 bp single-end reads by the BioFrontiers Sequencing Facility at the CU Boulder.

### PRO-seq computational analysis.

PRO-seq data were processed using a standardized Nextflow pipeline (https://github.com/Dowell-Lab/Nascent-Flow; master; commit d5946017c7e33bda3e990b10c177a693a3164dc1). A complete pipeline report detailing all software programs and versions utilized and a detailed quality control report including trimming, mapping, coverage, and complexity metrics are included in Supplemental Data File 3. Normalized TDF coverage files (reads per million mapped) output by the pipeline was visualized using the Integrative Genomics Viewer (IGV; see ref. 64). FStitch (v. 1.0) and Tfit (v. 1.0) were used to identify regions with bidirectional transcriptional activity (eRNAs) as described (22). Counts were calculated for each sorted BAM file using multiBamCov from the BEDTools suite (v. 2.25.0) (65) and RefSeq NCBI Reference Sequences for hg38 downloaded from the UCSC track browser (May 18, 2018) (66). Genes and lncRNAs were then filtered such that only the isoform with the highest number of reads per annotated length was kept, and DESeq2 (v. 1.20.0, Bioconductor release v. 3.7) was used to determine which genes were differentially transcribed between the different cell types separately. For bidirectional/eRNA comparisons, all bidirectional prediction Tfit calls were merged using mergeBed from BEDTools (v. 2.25.0) to generate an annotation file. Counts were then calculated for each sample using multicov (BEDTools v. 2.25.0), and DESeq2 was used to calculate differentially transcribed bidirectionals/eRNAs. PCA was performed within DESeq2 using the ggplot2 function.

### MD analysis.

Tfit-called bidirectionals/eRNAs were used as input for DAStk (v. 0.1.5; https://github.com/Dowell-Lab/dastk; run as part of the Nascent-Flow pipeline, referenced above) to calculate MD scores (67), which quantify the degree of colocalization of transcription factor consensus binding motifs with the center of each eRNA origin. FIMO (68) was used to identify matches to consensus binding motifs as defined by a set of binding motif position weight matrices (PWMs) obtained from a curated human transcription factor database (69, 70) using a *P* value cutoff of 1 × 10^−5^ with arguments “-max-stored-scores 10,000,000 -thresh 1 × 10^−5^.” Barcode plots were generated by mapping consensus binding motifs to hg38 using a *P* value cutoff of 1 × 10^−5^. For each motif instance, the number of hits using the eRNA center was used to calculate MD in a 3000 bp window around the center of the feature. A *z* test of 2 proportions was then used to determine statistically significant differences in the calculated MD scores between cell types.

### MNase-qPCR assay.

MNase-qPCR chromatin accessibility assays were performed as described (22). Assays were generally performed in biologic quadruplicate and repeated at least 3 times with qualitatively similar results. Tiled primer sets used for qPCR analysis are shown in Supplemental Table 5.

### ATAC-seq.

Primary airway epithelial cells cultured at ALI, cells from fresh bronchial brushes, or BEAS-2B cells cultured to confluence were washed twice with 1× PBS and collected by scraping or pelleting prior to counting. Approximately 50,000 cells were pelleted and processed in duplicate for Omni-ATAC-seq as described (71). Uniquely indexed libraries were pooled and sequenced on an Illumina NextSeq instrument using 75 bp single-end reads (sample Norm-1) or 37 bp paired-end reads (all other samples) by the BioFrontiers Sequencing Facility at the CU Boulder.

### ATAC-seq computational analysis.

ATAC-seq data were processed using a standardized Nextflow pipeline (https://github.com/Dowell-Lab/ChIP-Flow; master; commit 44fd202d9fa2355366e1ac20f41cf67fd4a6ebc4). Supplemental Data File 4 contains detailed pipeline and quality control reports. Normalized TDF coverage files (reads per million mapped) output by the pipeline was visualized using IGV.

### snRNA-seq.

snRNA-seq for human lung tissue was performed as described (72). In brief, tissue was pulverized in liquid nitrogen using a mortar and pestle. Tissue was suspended in PBS containing 2% BSA, 0.1% Triton X-100, 1× protease inhibitors (Roche), 0.2 U/μL RNase inhibitor (Promega), and 1 mM DTT and incubated for 5 minutes on a rotator at 4°C. Nuclei were pelleted by centrifugation in a swinging bucket centrifuge (500*g*, 5 minutes, 4°C) and resuspended in PBS containing 2% BSA, 1 mM EDTA, 0.2 U/μL RNase inhibitor, and 1:100 DRAQ7 (Cell Signaling Technology). Nuclei were sorted into collection buffer (PBS containing 5% BSA and 1 U/μL RNase inhibitor) using an SH800 sorter (Sony), pelleted (1000*g*, 15 minutes), and resuspended in reaction buffer (0.2 U/μL RNase inhibitor [Promega], 2% BSA [Sigma] in PBS). Twelve thousand nuclei were loaded onto a Chromium Controller (10× Genomics), and libraries were generated using the Chromium Single Cell 3′ GEM, Library & Gel Bead Kit v3 (10× Genomics) following manufacturer instructions. Final libraries were sequenced on a HiSeq4000 and NextSeq500 sequencer (Illumina) with the following read lengths: 28 + 8 + 91 (Read1 + Index1 + Read2).

### snATAC-seq.

snATAC-seq using combinatorial barcoding (73) for human lung tissue was performed as described (72). In brief, pulverized tissue was suspended in nuclei permeabilization buffer (10 mM Tris-HCl [pH 7.5], 10 mM NaCl, 3 mM MgCl_2_, 0.1% Tween 20 [MilliporeSigma], 0.1% IGEPAL-CA630 [MilliporeSigma], and 0.01% Digitonin [Promega] in water; ref. 71) by pipetting, incubated for 10 minutes at 4°C, and filtered with a 30 μm filter (CellTrics). Nuclei were pelleted in a swinging bucket centrifuge (500*g*, 5 minutes, 4°C), resuspended in 500 μL high-salt tagmentation buffer (36.3 mM Tris-acetate [pH 7.8], 72.6 mM potassium-acetate, 11 mM Mg-acetate, 17.6% DMF), and counted using a hemocytometer. Two thousand nuclei were added to individual wells of a 96-well plate and tagmented with 1 μL barcoded Tn5 transposomes for 60 minutes at 37°C (74). After tagmentation, nuclei were combined, and 20 diploid nuclei were sorted per well into eight 96-well plates (total of 768 wells). Tagmented DNA was PCR amplified using primers with well-specific barcodes, and all wells were combined after completion of PCR. Purified and size-selected libraries were sequenced on a HiSeq4000 sequencer (Illumina) using custom sequencing primers with the following read lengths: 50 + 10 + 12 + 50 (Read1 + Index1 + Index2 + Read2).

### Single-nucleus sequencing computational analysis.

For snRNA-seq, fastq files were trimmed, quality filtered, and aligned to the hg38 reference genome using standardized Cell Ranger analysis pipelines from 10× Genomics to generate a count matrix for each sample of cell barcodes × aligned genes. This matrix was imported into Seurat (v. 3; https://satijalab.org/seurat/; see ref. 75), selected to exclude samples with > 10% mitochondrial reads and integrated using the sctransform wrapper (Seurat v. 3), which applies a regularized, negative binomial regression approach to per-cell read depth as a normalization across samples (76). Principal components were calculated and selected by looking at the inflection point of a histogram of principal components by SD. Dimensionality was reduced, and cell groups were defined by unbiased hierarchical clustering using the Uniform Manifold Approximation and Projection (UMAP) method (available through Seurat v. 3). Cell types were identified using several approaches, including marker analysis to summarize discriminating genes between clusters and graphical approaches testing for known cell type–specific markers; marker genes used in this analysis are summarized in Supplemental Table 6.

For snATAC-seq, reads were demultiplexed (https://gitlab.com/Grouumf/ATACdemultiplex; master; commit 0a237edd1536a9ec22734be271df6192a979afef), and subsequently, fastq files were trimmed, filtered, and aligned to the hg38 reference genome using SnapATAC (v. 2.0; https://github.com/r3fang/SnapATAC; master; commit c3ab177558f0fe9c47cbd68969df7b06de5b07d9). Resulting BAM files were converted into fragment files using Sinto (v. 0.7.1.; https://github.com/timoast/sinto; master; commit 32d8733be9ba79372001318174d3612dc73c28b0), and peak calls and counts tables for each sample were constructed with Genrich (v. 0.6; https://github.com/jsh58/Genrich#atacseq; master; commit d896ab193a2c399ae533f7f470f3450da425131f). Count matrices were imported into Signac (v. 1.1.0; ref. 77), unaligned counts were removed, and cells with nucleosome signal > 10, transcription start site enrichment < 2, alignment to error-prone regions > 5%, and <15% read fractions in peaks were filtered out. Acceptable signal/noise ratio was evidenced by an average TSS enrichment > 7 for each of the 3 libraries. Sample integration and dimensionality reduction were performed in Signac using term frequency–inverse document frequency (TF-IDF) weighted integration, singular value decomposition, and latent semantic indexing dimensionality reduction. Cell type labels were transferred from cells labeled using the snRNA-seq pipeline above by performing canonical correlation analysis across data sets with UMAP. Pseudo-bulk chromatograms were created from aggregated fragment files using Signac.

### Data and materials availability.

Genomics data have been deposited in GEO under SuperSeries accession GSE157691 (https://www.ncbi.nlm.nih.gov/geo/query/acc.cgi?acc=GSE157691). All plasmids and cell lines are available upon request to corresponding authors.

### Statistics.

Statistical comparisons for luciferase reporter and ChIP-qPCR assays were made by 2-tailed *t* test or 1-way ANOVA with post hoc Bonferroni correction where appropriate. Nonparametric analysis was performed for qPCR experiments using Mann-Whitney *U* tests. These analyses were conducted in Prism v. 8 (GraphPad), with a *P* value of less than 0.05 considered significant.

For MD score comparisons, the significance threshold was set at *P* = 0.0001. For single nucleus sequencing, fold-change comparisons were based on logistic or negative binomial regression, as indicated in the text, and *P*_adj_ < 0.05; single nucleus statistical analyses utilized R (v. 3.6.1; R Foundation for Statistical Computing).

### Study approval.

This study did not include identifiable animal or human data. Primary human airway epithelial samples were obtained and used in a deidentified manner using the National Jewish Health Institutional Honest Broker Services under IRB protocol HS-2604. Samples obtained for snRNA-seq and snATAC-seq were identifiable only to the institution of origin (Lung Transplant Research Consortium in the case of sample GG_IPF; University of Pittsburgh in the case of GG_control, TT_control, and TT_IPF). Participant age, race, sex, and diagnosis were the only demographic information available to investigators; thus, these samples were deemed exempt human subject research under the terms of the Colorado Multiple IRB (COMIRB 15-1147). Consent for the use of these tissues in genetic research was obtained by the donor institutions.

## Author contributions

RDD, BR, SPC, DAS, and ANG designed the research studies; FG, SKS, JSK, XH, OBP, JB, TO, and SP conducted the experiments; FG, SKS, JSK, JHC, and MAG acquired and analyzed the data; HWC, IVY, and DAS provided the reagents; FG, SKS, JSK, DAS, and ANG wrote and edited the manuscript; all authors reviewed and approved the final manuscript. The order of the co–first authors was based on the timeline of contributions to the work.

## Supplementary Material

Supplemental data

Supplemental Data Set 1

Supplemental Data Set 2

Supplemental Data Set 3

Supplemental Data Set 4

## Version 1 12/15/2020

In-Press Preview

## Version 2 01/25/2021

Electronic publication
