# Supplementary material for: The MUC5B-associated variant rs35705950 resides within an enhancer subject to lineage- and disease-dependent epigenetic remodeling
Source: JCI Insight. 2021 Jan 25;6(2):e144294. doi: 10.1172/jci.insight.144294 (PMC7934873; doi:10.1172/jci.insight.144294)
Supplement: Supplemental data [file jciinsight-6-144294-s075.pdf]

Supplementary Materials

Figure S1. The *MUC5B* -3 kb region is hyper-ChIPable in multiple airway cell types despite variable levels of endogenous gene expression.

Figure S2. PRO-seq nascent transcript profiling reveals shared and unique patterns of basal gene and eRNA transcription in *MUC5B*-expressing A549 and LC-2/ad cells and non-*MUC5B*-expressing BEAS-2B cells.

Figure S3. MNase accessibility assay reliably detects relative insensitivity to MNase digestion in a predicted nucleosome-protected negative control region.

Figure S4. Primary human airway epithelial cells differentiated at air-liquid interface (ALI) exhibit basal *MUC5B* expression and hyper-ChIPable chromatin within the *MUC5B* -3 kb enhancer.

Figure S5. Violin-style plots depicting snATAC-seq integration and snRNA-seq expression data as a function of indicated cell type, disease status and genotype at the *MUC5B* (A), *DNAH2* (B) and *RPL13* (C) loci.

Table S1. Single nuclear RNA- and ATAC-seq sequencing and sample characteristics

Table S2. Primers for generating *MUC5B* reporters

Table S3. ChIP conditions

Table S4. Primers for ChIP-qPCR

Table S5. Sequences of primers used for tiled qPCR

Table S6. Lineage-specific markers used to define indicated airway cell subpopulations for snRNA-seq and snATAC-seq

File S1. Output files resulting from motif displacement (MD) analysis of PRO-seq data performed by DAStk tool (https://github.com/Dowell-Lab/DAStk) with no TSS.

File S2. Output files resulting from motif displacement (MD) analysis of PRO-seq data performed by DAStk tool (https://github.com/Dowell-Lab/DAStk) with TSS.

File S3. Detailed MultiQC quality control and Nextflow pipeline reports, including all software programs and versions used, in the analysis of PRO-seq data (https://github.com/Dowell-Lab/Nascent-Flow).

File S4. Detailed MultiQC quality control and Nextflow pipeline reports, including all software programs and versions used, in the analysis of ATAC-seq data (https://github.com/Dowell-Lab/ChIP-Flow).

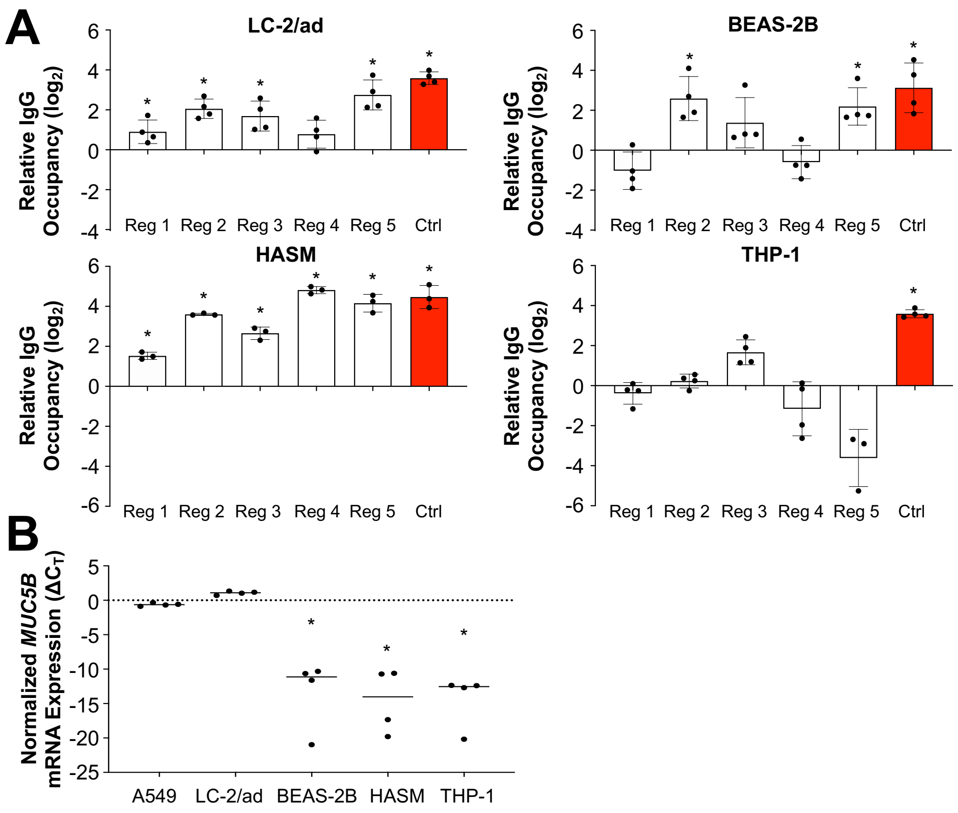

Figure S1. The *MUC5B* -3 kb region is hyper-ChIPable in multiple airway cell types despite variable levels of endogenous gene expression. (A) ChIP-qPCR analysis of mean (±SD) relative IgG occupancy across the *MUC5B* enhancer region in LC-2/ad, BEAS-2B, primary human airway smooth muscle (HASM) and THP-1 cells (*p $\leq$ 0.05 vs occupancy at negative controls for each sample, t-test, n = 4). Data are representative of 3 independent experiments. See Fig. 1G for regions targeted by qPCR primers. A universally hyper-ChIPable region in the *TNFAIP3* locus was used as a positive control (Ctrl; red bars). **(B)** qRT-PCR analysis of *MUC5B* mRNA expression normalized to *RPL19* of indicated cell types (*p $\leq$ 0.05 vs combined levels for A549 and LC-2/ad, Mann Whitney, n = 4).

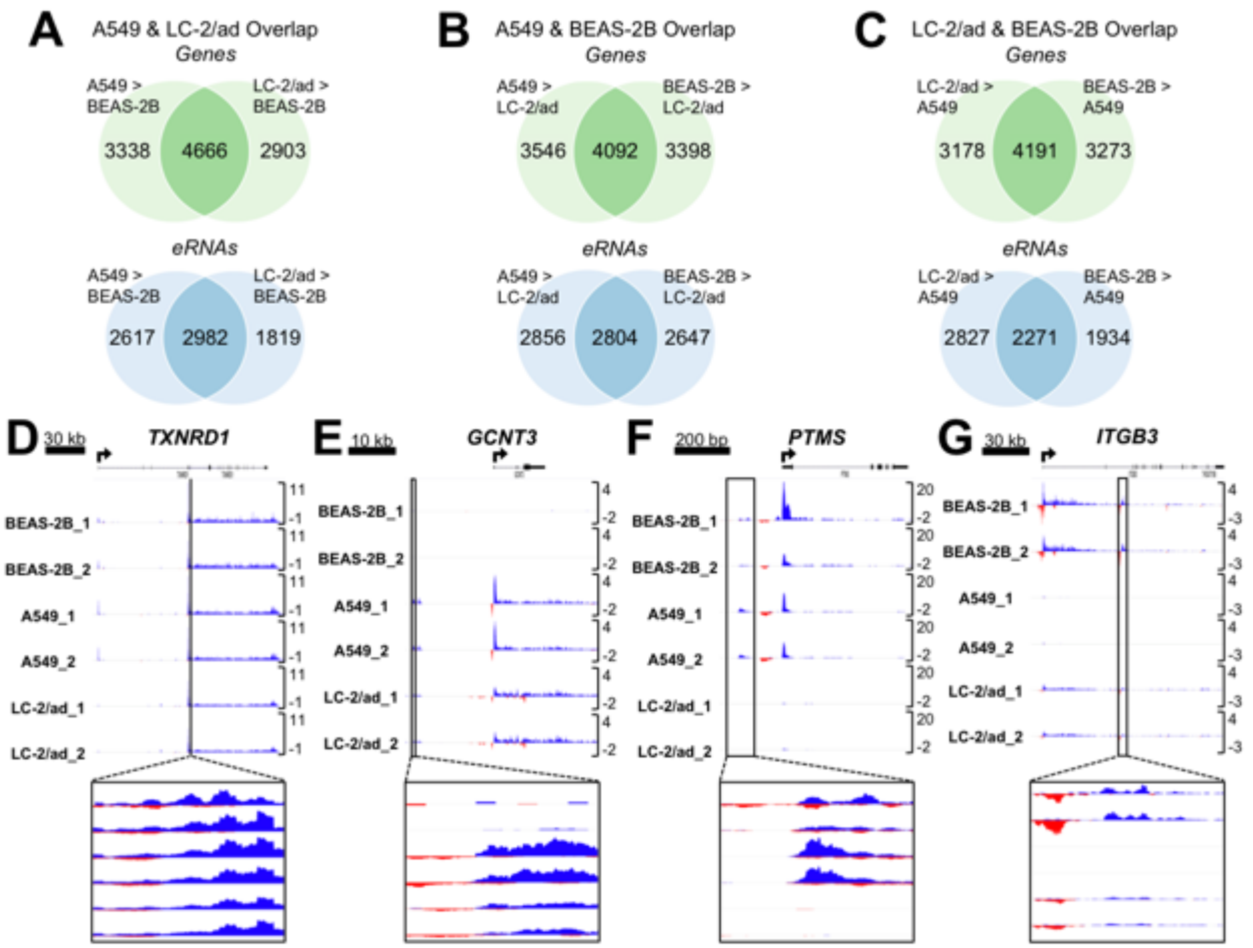

Figure S2. PRO-seq nascent transcript profiling reveals shared and unique patterns of basal gene and eRNA transcription in *MUC5B*-expressing A549 and LC-2/ad cells and non-*MUC5B*-expressing BEAS-2B cells. (A-C) Venn diagrams illustrate differentially regulated genes (top) and eRNAs (bottom) identified by DESeq2 ($p_{adj} < 0.05$) and overlap using indicated pairwise comparisons. **(D-G)** PRO-seq data visualized in the IGV Genome Browser depicting different patterns of gene and eRNA transcription in the three cell types sequenced in duplicate (n = 2).

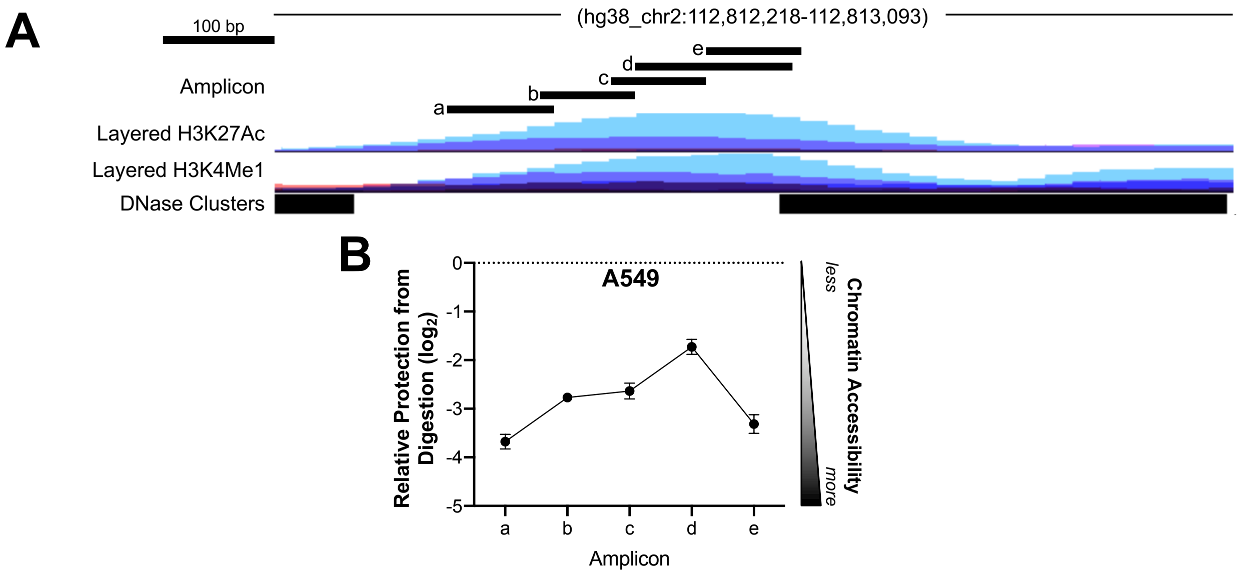

Figure S3. MNase accessibility assay detects relative insensitivity to MNase digestion in a predicted nucleosome-protected negative control region. (A) UCSC Genome Browser visualization of a chromatin region predicted to be relatively protected from nuclease access based on histone marks and DNase-seq data available through ENCODE. Tiled primers yielding indicated amplicons (a-e) were designed to interrogate this region using qPCR **(B)** in the same A549 chromatin samples tested in Fig. 3A (n = 4).

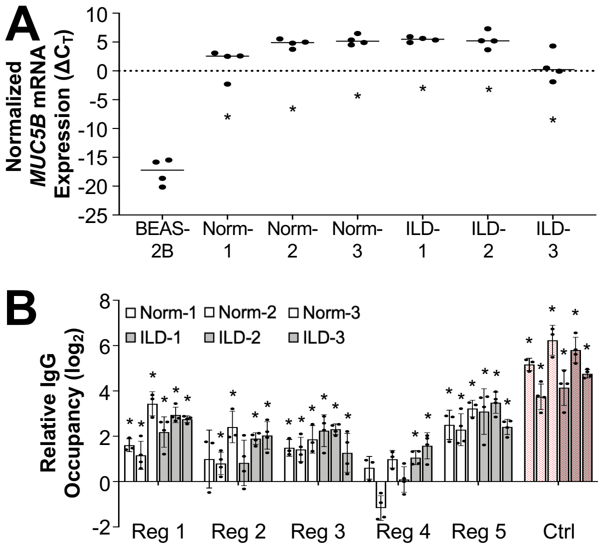

Figure S4. Primary human airway epithelial cells differentiated at air-liquid interface (ALI) exhibit basal *MUC5B* expression and hyper-ChIPable chromatin within the *MUC5B* -3 kb enhancer. (A) qRT-PCR analysis quantifying normalized *MUC5B* mRNA expression in indicated airway cell samples (*$p < 0.05$ vs BEAS-2B, t-test, $n = 4$). **(B)** ChIP-qPCR analysis of mean (±SD) relative IgG occupancy across the *MUC5B* enhancer region in primary airway epithelial cells from normal and ILD donors cultured at ALI (*$p < 0.05$ vs occupancy at negative controls for each sample, t-test, $n = 4$). See Fig. 1G for regions targeted by qPCR primers. A universally hyper-ChIPable region in the *TNFAIP3* locus was used as a positive control (Ctrl; red bars).

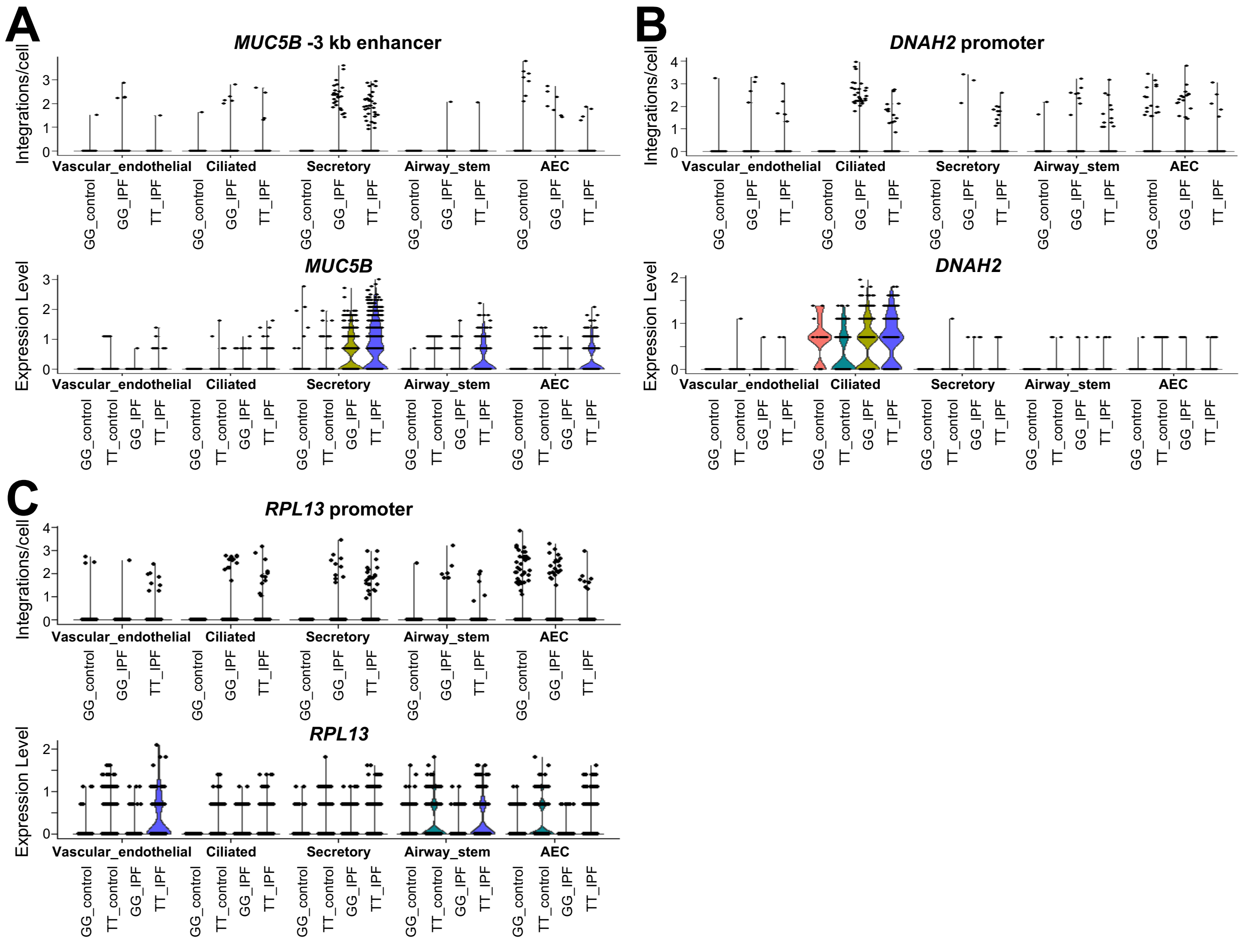

Figure S5. Violin-style plots depicting snATAC-seq integration and snRNA-seq expression data as a function of indicated cell type, disease status and genotype at the *MUC5B* (A), *DNAH2* (B) and *RPL13* (C) loci.

Table S1. Single nuclear RNA- and ATAC-seq sequencing and sample characteristics

	Control GG	Control TT	IPF GG	IPF TT
Age	64	69	65	64
Race	White	White	White	White
Sex	Male	Male	Male	Male
RNA unique barcodes[1]	2247	7229	4800	4757
RNA counts /nucleus[2]	4242	1354	1761	3965
ATAC-seq unique barcodes[1]	5854	NA	5502	4831
ATAC-seq counts /nucleus[2]	5403	NA	5493	9051
Cell-type prediction[3]	0.61	NA	0.66	0.77

[1]Number of nuclei recovered (based on unique barcodes) and the number of aligned reads (counts) per nucleus (barcode)
[2]Number of aligned reads (counts) per nucleus (barcode). For ATAC-seq, cells were prefiltered based on alignment, and cells with an excess of "blacklist" sites are removed
[3]Prediction rate of matching cell type calls of RNA-seq data to cells in the ATAC-seq dataset based on canonical correlation analysis

Table S2. Primers for generating *MUC5B* reporters

	Forward Sequence (5' to 3')	Reverse Sequence (5' to 3')
MUC5B Short	TGATCACTGGTGCCTGGA	ACCTCTGCATCAGCGAGATA
MUC5B STAT3 SDM	GAGCCGCAGGGCAAGAAGCTTGCACGTCCGAGGTGG	CCACCTCGGACGTGCAAGCTTCTTGCCCTGCGGCTC
MUC5B ETS1/SPDEF SDM	CCGACTCCCAGGGCCGCACTAGTGATGTCTCAATAGCTGTG	CACAGCTATTGAGACATCACTAGTGCGGCCCTGGGAGTCGG
MUC5B Short G>T SDM	CTTTATCTTCTGTTTTCAGCTCCTTCAACTGTGAAGAGGTG	CACCTCTTCACAGTTGAAGGAGCTGAAAACAGAAGATAAAG

Table S3. ChIP conditions

	Fixation (min)	Sonication (cycles of 30 sec on/30 sec off)
A549	5	25
LC-2/ad	10	35
HASM	5	27
BEAS-2B	5	37
THP-1	5	20
Primary airway epithelial cells	5	35

Table S4. Primers for ChIP-qPCR

	Forward Sequence (5' to 3')	Reverse Sequence (5' to 3')
Reg 1	CCCATTCATGGCAGGATTAAC	GGTCACTGTCCCTCATTCT
Reg 2	TGTGTCCCTTTCCTTCCTTTATC	CTGTTTGCTCAGCGTGTTTG
Reg 3	CTTCAAACACGCTGAGCAAAC	GGGCCCATTGGGAACTG
Reg 4	CTCAATAGCTGTGGCCTTGA	CACACAGTGACACCAAACAAG
Reg 5	CACACAGTGACACCAAACAAG	TCAGGCAGCTCCTCTGTC
Ctrl (*TNFAIP3*)	GACCACACCCACTTGGAAA	TTTGACTAGCAATTGAGCAACAG
Negative Ctrl 1	CGGCTACTAGCGGTTTTACG	AAGAAGATGCGGCTGACTGT
Negative Ctrl 2	TGCAGGAGATGAAATACTAAGCAAGTA	AGATTGGAAACTGAGGACTTTAGTTAGAG
Negative Ctrl 3	GGCAAGGACAGAGACAATCATA	CTCTGTGTTCTCGCTTTGGA

Table S5. Sequences of primers used for tiled qPCR

	Forward Sequence (5' to 3')	Reverse Sequence (5' to 3')	bp from rs35705950
MUC5B -3 kb region			
5’ Reg 1	CCCAGTGACGACTCTTTGC	TGATCAGGGCCACCATCT	-309
Reg 1	Same as for ChIP-qPCR		-259
5’ Reg 2	GCCAGAATGAGGGACAGTG	GGACGTGCCAGGAACTTG	-185
Reg 2	Same as for ChIP-qPCR		-36
Reg 3	Same as for ChIP-qPCR		24
Reg 4	Same as for ChIP-qPCR		77
5’ Reg 5	GAGGGACGAGGGCAAAG	CAGTGCCTGTGGTCCAA	171
Reg 5	Same as for ChIP-qPCR		196
Negative control region			
a	GGCCTGACTCTCTTTACCTTTC	GGCTGGAACTCAAGCATACA	
b	GCTTGAGTTCCAGCCATTTG	CGGGTTTAGCACACAGTCTAT	
c	GACTGCCATGTGTGTAGACTT	AGAACCATGGACATTCCTCATC	
d	GCCACTTCCAAGGAGTAGATG	GGCCCTAGTACAGACGAGTTA	
e	GCAGACATTCCAAAGCTCATTT	CTCCTGGGCCCTAGTACA	

Table S6. Lineage-specific markers used to define indicated airway cell subpopulations for snRNA-seq and snATAC-seq

**Cell type**	**snRNA-seq**	**snATAC-seq**
Alveolar epithelial	*HOPX*, *AGER*, *SFTPB*, *SFTPC*, *SLC34A2*, *ABCA3*	*CELSR1, SFTPB, SLC34A2*
Secretory	*SCGB1A1*, *SCGB3A2*, *SPDEF*, *PIGR*, *BPIF1A*	*CELSR1, ELF3*
Ciliated	*DNAH12*, *PIFO*, *TUB1A1, IFT88, CDC20B, RFX3*	*CELSR1, ERICH3, TPPP3*
Airway stem	*KRT5*, *KRT17*, *EYA2*, *TP63*, *ITGB4*, *SOX2*, *COL17A1*	*WNT3A, CELSR1*
Vascular endothelial	*VWF, PECAM, EMCN, EPAS1*	*TEK, CAVIN1*
Fibroblast	*COL1A1*, COL1A2*, COL3A1, COL4A2, PDGFRA, LAMA2, FBLN1*	*FBLN1, SMTNL2, COL6A1,*
Myofibroblast	*COL3A1, PDGFRA, ACTA2*	*FBLN1, SMTNL2, COL6A1, FRZB*
Alveolar macrophage	*HLA-DMB*, *ZEB2*, *CD74*, *MARCO*	*MARCO, PPARG*
Macrophage	*HLA-DMB, ZEB2, CD74*	*ABR, ENG, PIK3R5, GPR137B*
Monocyte	*ZEB2*	*HCK, SULF2, VASH1*
Mast cell	*KIT*	
T cell	*THEMIS, CD3E*	*ITK, BCL11B, PRKCQ*
Naïve B cell	*MS4A1, HLA-DMB*	*BTLA*
Memory B cell	*IGKC, IGHA1*	*FCRL5*
Plasma cell	*PRDM1, IRF4, IGKC*	
