# Supplementary figures and images for: The MUC5B-associated variant rs35705950 resides within an enhancer subject to lineage- and disease-dependent epigenetic remodeling (part 2 of 3)
Source: JCI Insight. 2021 Jan 25;6(2):e144294. doi: 10.1172/jci.insight.144294 (PMC7934873; doi:10.1172/jci.insight.144294)

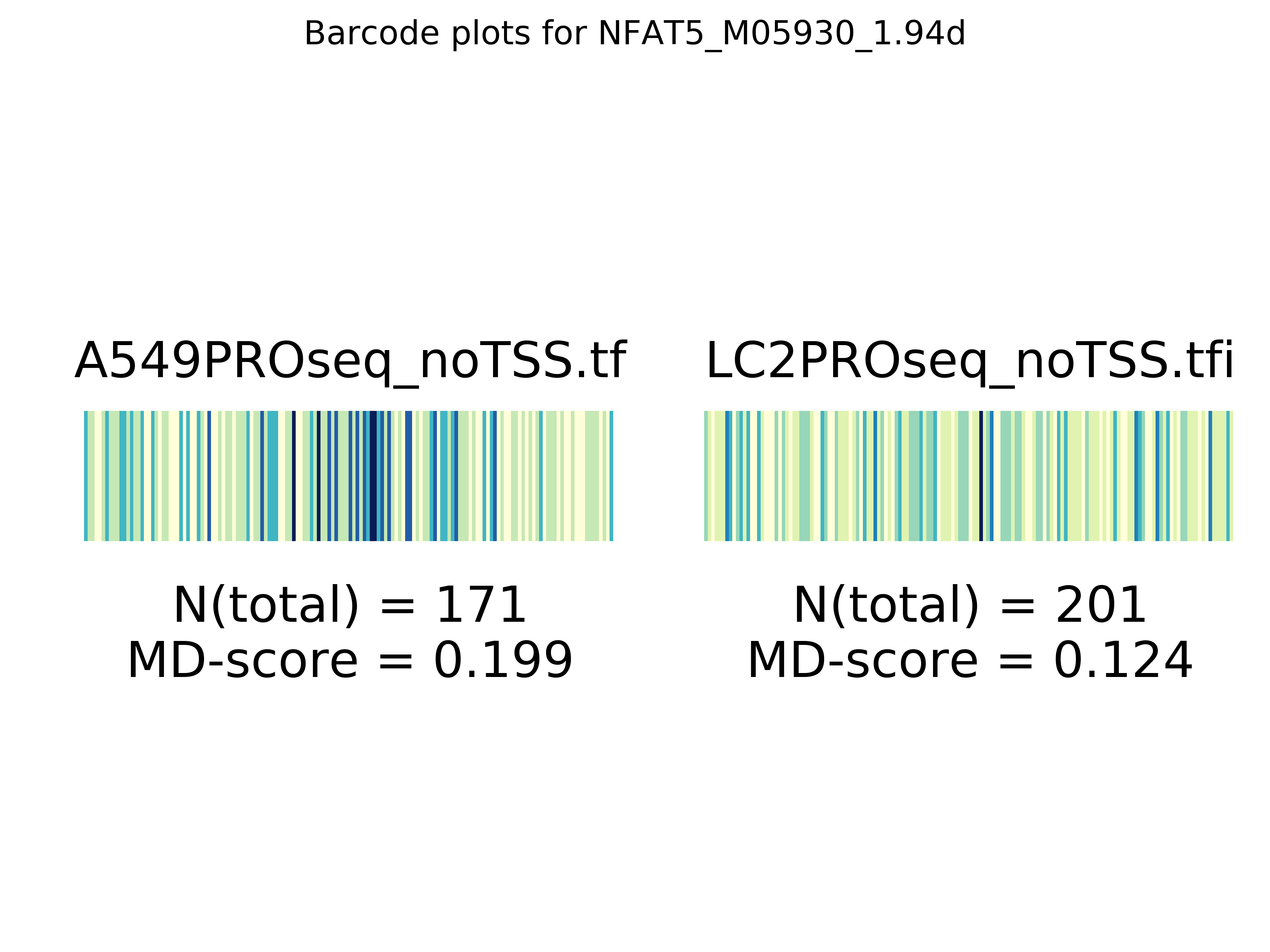

Supplement: Supplemental Data Set 1 [file jciinsight-6-144294-s076.zip › noTSS/best_curated_Human_TFs_p1e-6_grch38/A549_vs_LC2/NFAT5_M05930_1.94d_barcode_A549PROseq_noTSS.tfit_merged_vs_LC2PROseq_noTSS.tfit_merged.png]

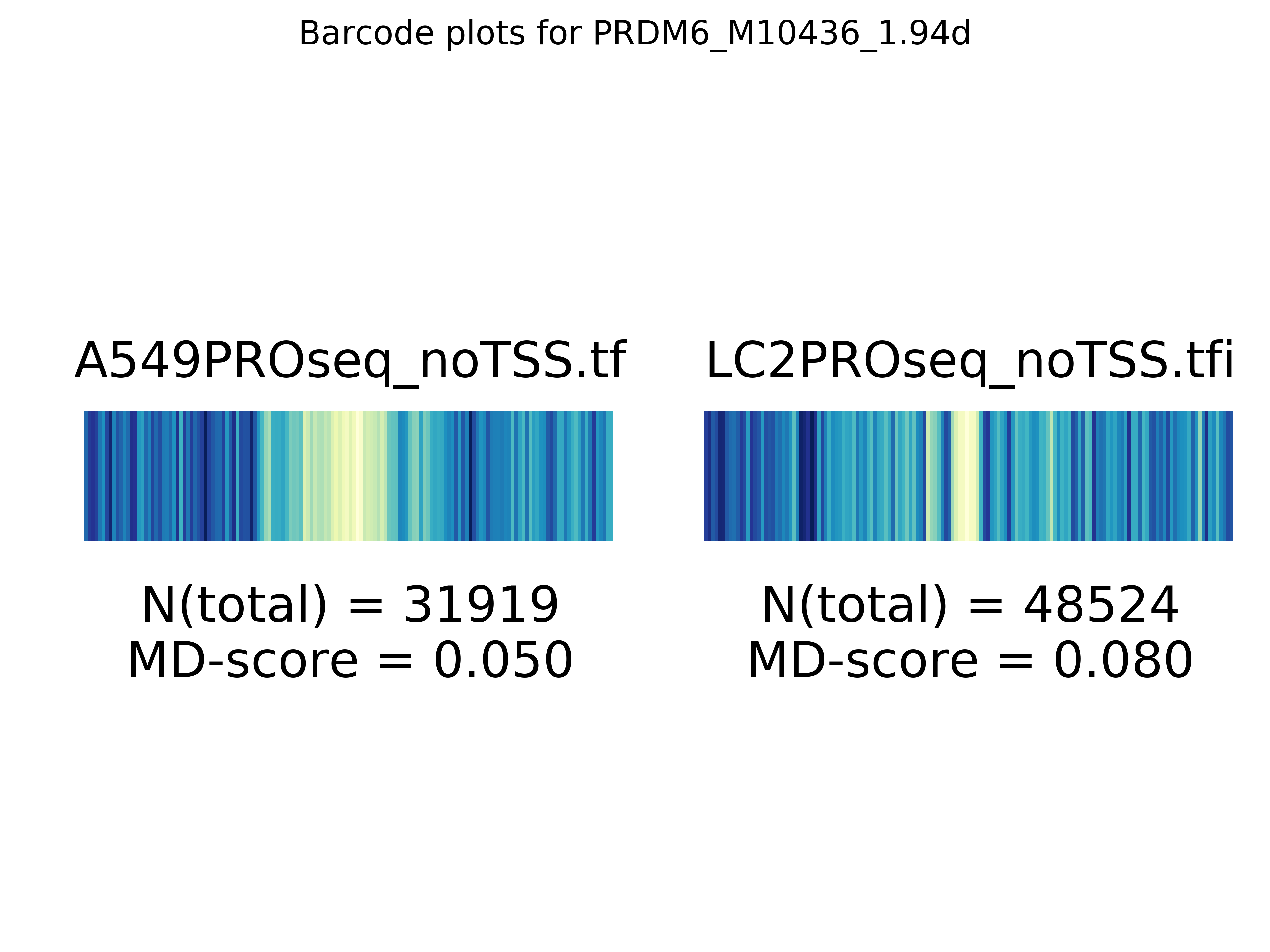

Supplement: Supplemental Data Set 1 [file jciinsight-6-144294-s076.zip › noTSS/best_curated_Human_TFs_p1e-6_grch38/A549_vs_LC2/PRDM6_M10436_1.94d_barcode_A549PROseq_noTSS.tfit_merged_vs_LC2PROseq_noTSS.tfit_merged.png]

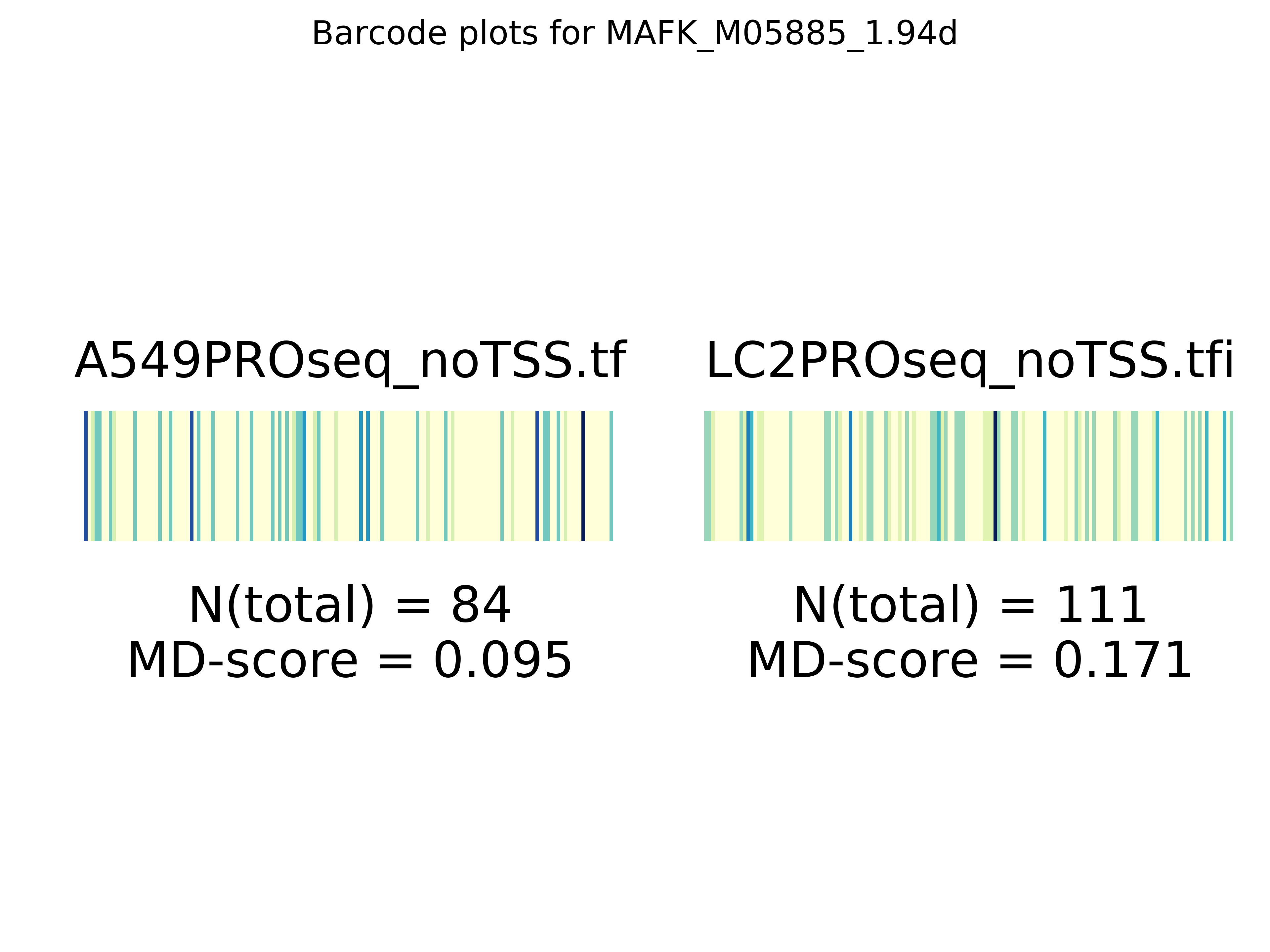

Supplement: Supplemental Data Set 1 [file jciinsight-6-144294-s076.zip › noTSS/best_curated_Human_TFs_p1e-6_grch38/A549_vs_LC2/MAFK_M05885_1.94d_barcode_A549PROseq_noTSS.tfit_merged_vs_LC2PROseq_noTSS.tfit_merged.png]

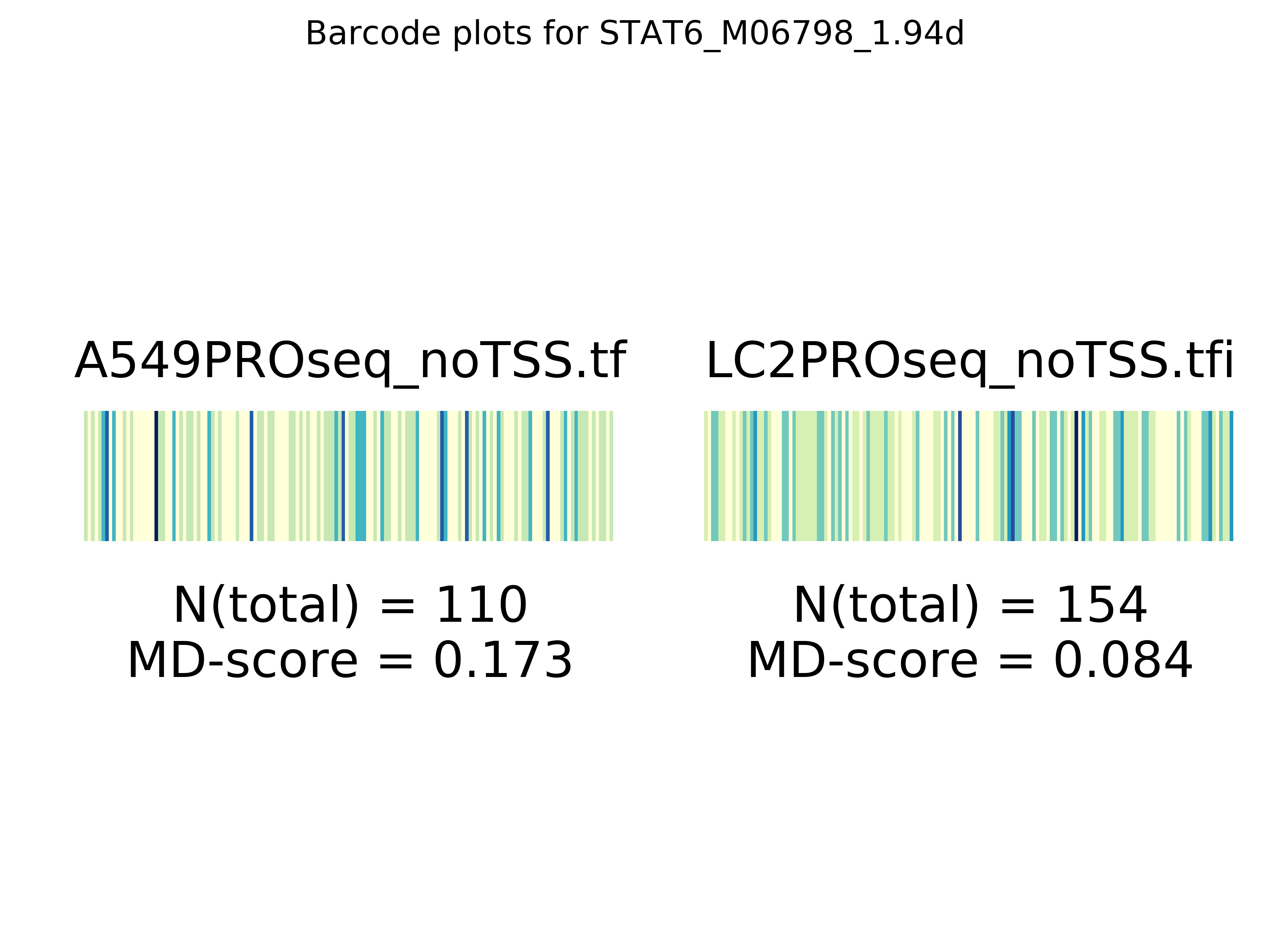

Supplement: Supplemental Data Set 1 [file jciinsight-6-144294-s076.zip › noTSS/best_curated_Human_TFs_p1e-6_grch38/A549_vs_LC2/STAT6_M06798_1.94d_barcode_A549PROseq_noTSS.tfit_merged_vs_LC2PROseq_noTSS.tfit_merged.png]

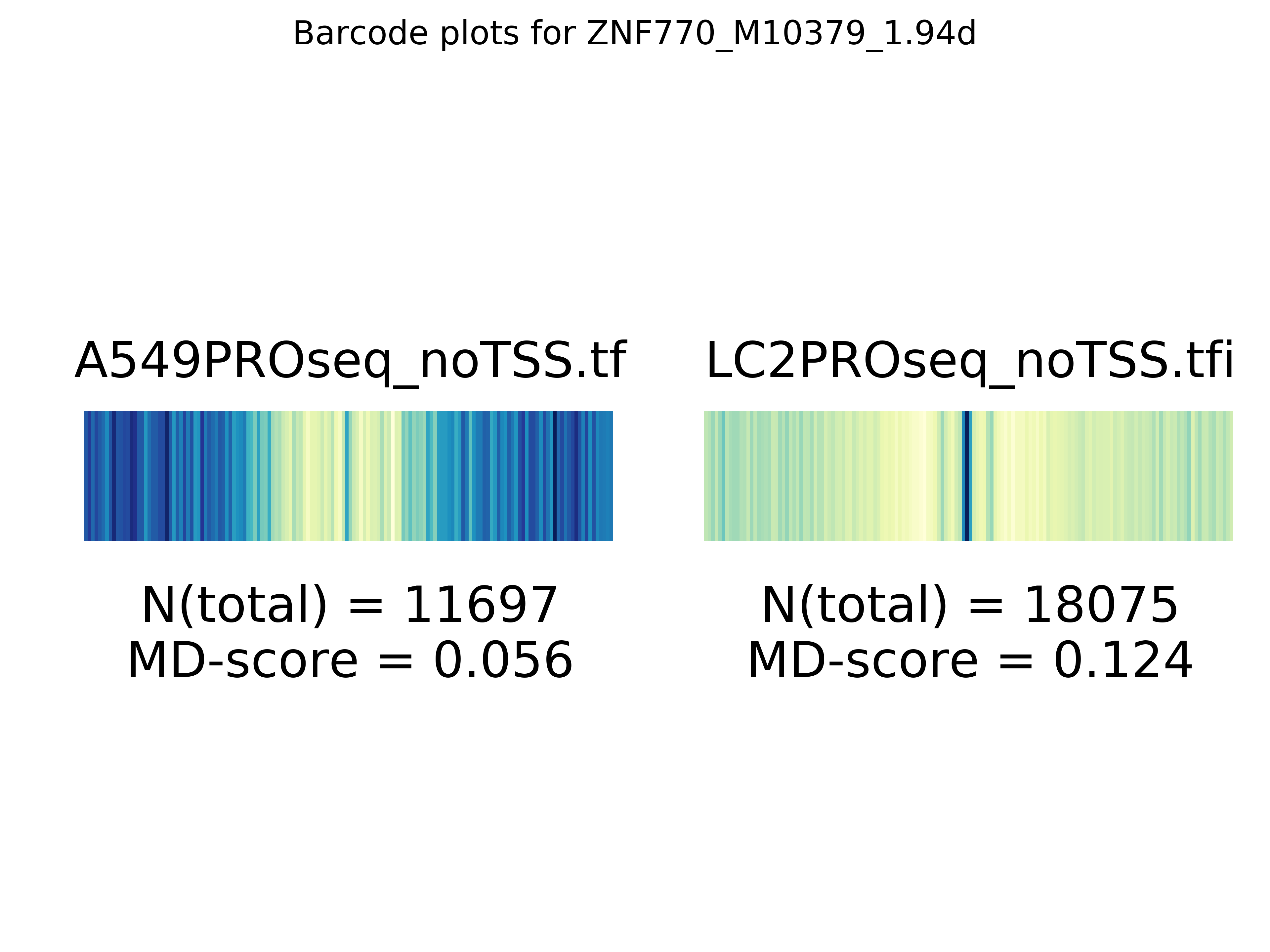

Supplement: Supplemental Data Set 1 [file jciinsight-6-144294-s076.zip › noTSS/best_curated_Human_TFs_p1e-6_grch38/A549_vs_LC2/ZNF770_M10379_1.94d_barcode_A549PROseq_noTSS.tfit_merged_vs_LC2PROseq_noTSS.tfit_merged.png]

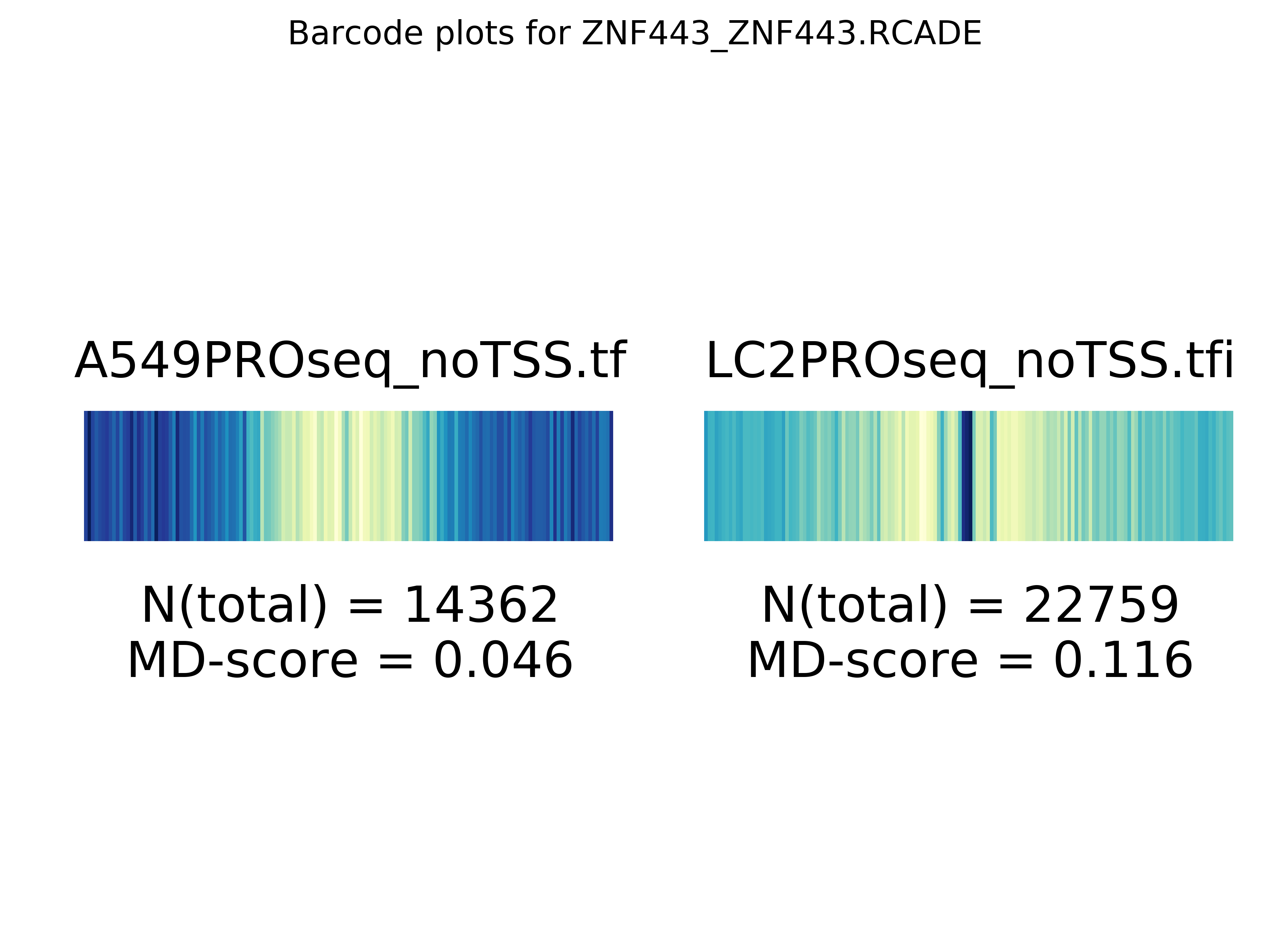

Supplement: Supplemental Data Set 1 [file jciinsight-6-144294-s076.zip › noTSS/best_curated_Human_TFs_p1e-6_grch38/A549_vs_LC2/ZNF443_ZNF443.RCADE_barcode_A549PROseq_noTSS.tfit_merged_vs_LC2PROseq_noTSS.tfit_merged.png]

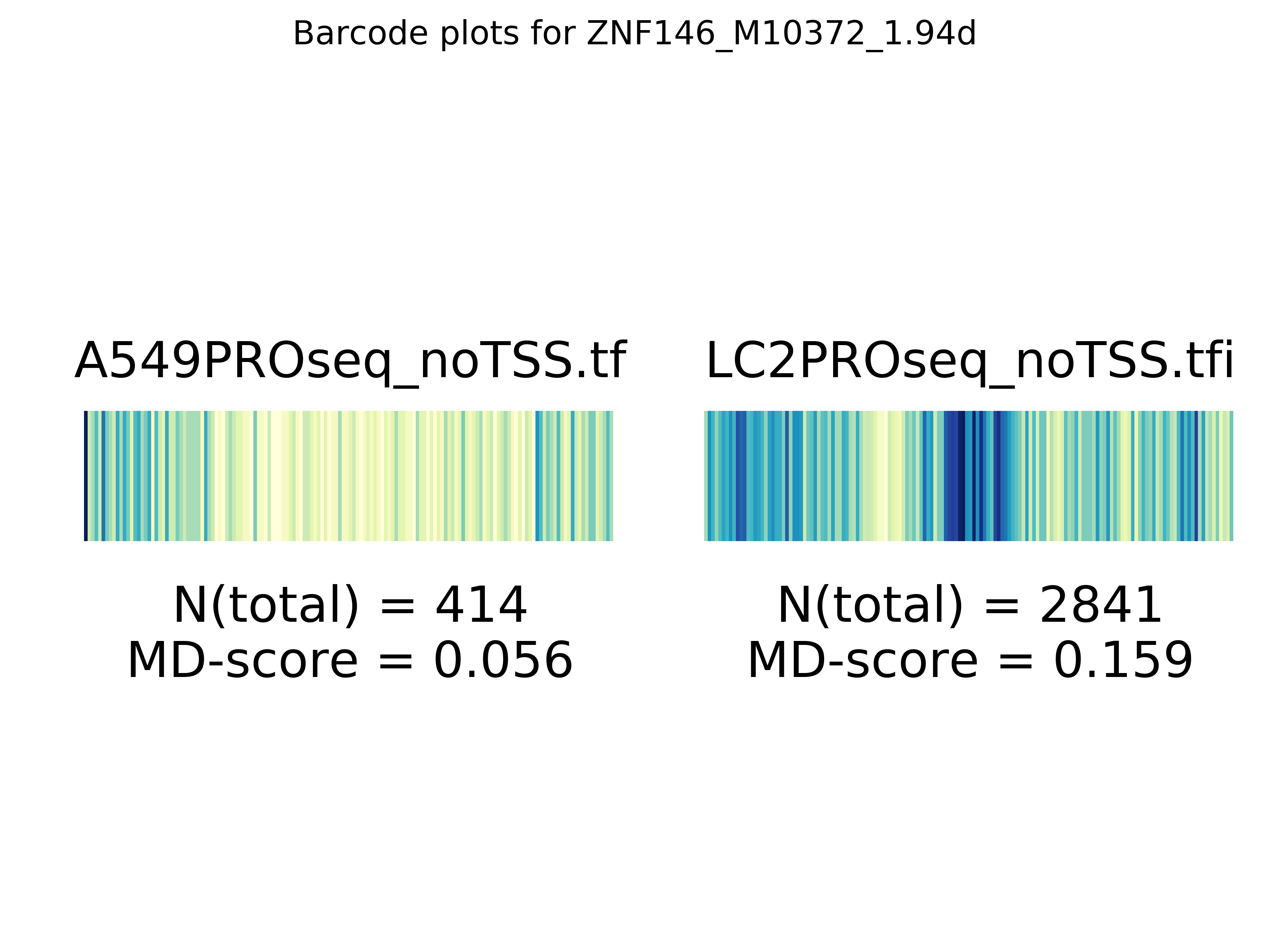

Supplement: Supplemental Data Set 1 [file jciinsight-6-144294-s076.zip › noTSS/best_curated_Human_TFs_p1e-6_grch38/A549_vs_LC2/ZNF146_M10372_1.94d_barcode_A549PROseq_noTSS.tfit_merged_vs_LC2PROseq_noTSS.tfit_merged.png]

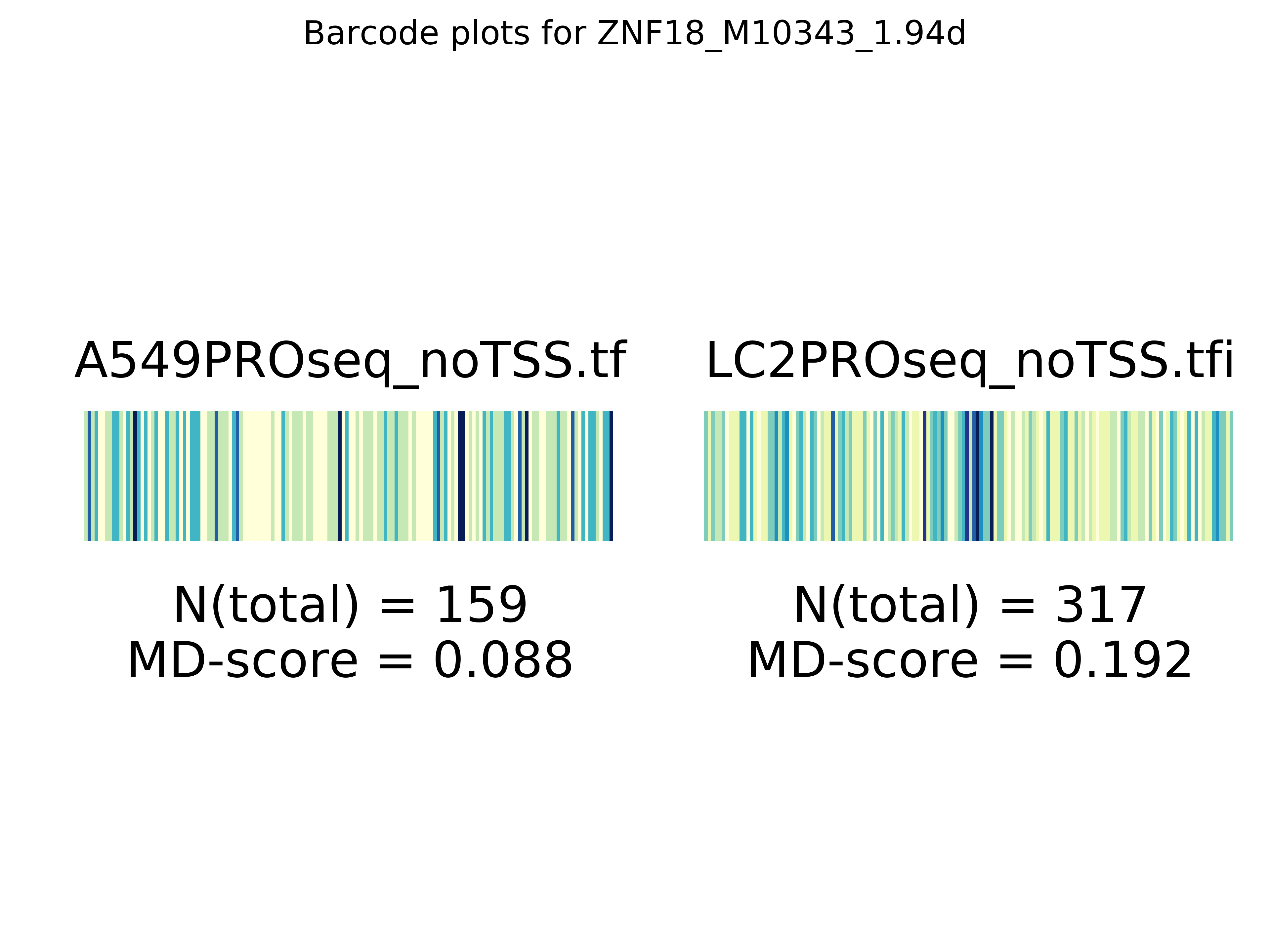

Supplement: Supplemental Data Set 1 [file jciinsight-6-144294-s076.zip › noTSS/best_curated_Human_TFs_p1e-6_grch38/A549_vs_LC2/ZNF18_M10343_1.94d_barcode_A549PROseq_noTSS.tfit_merged_vs_LC2PROseq_noTSS.tfit_merged.png]

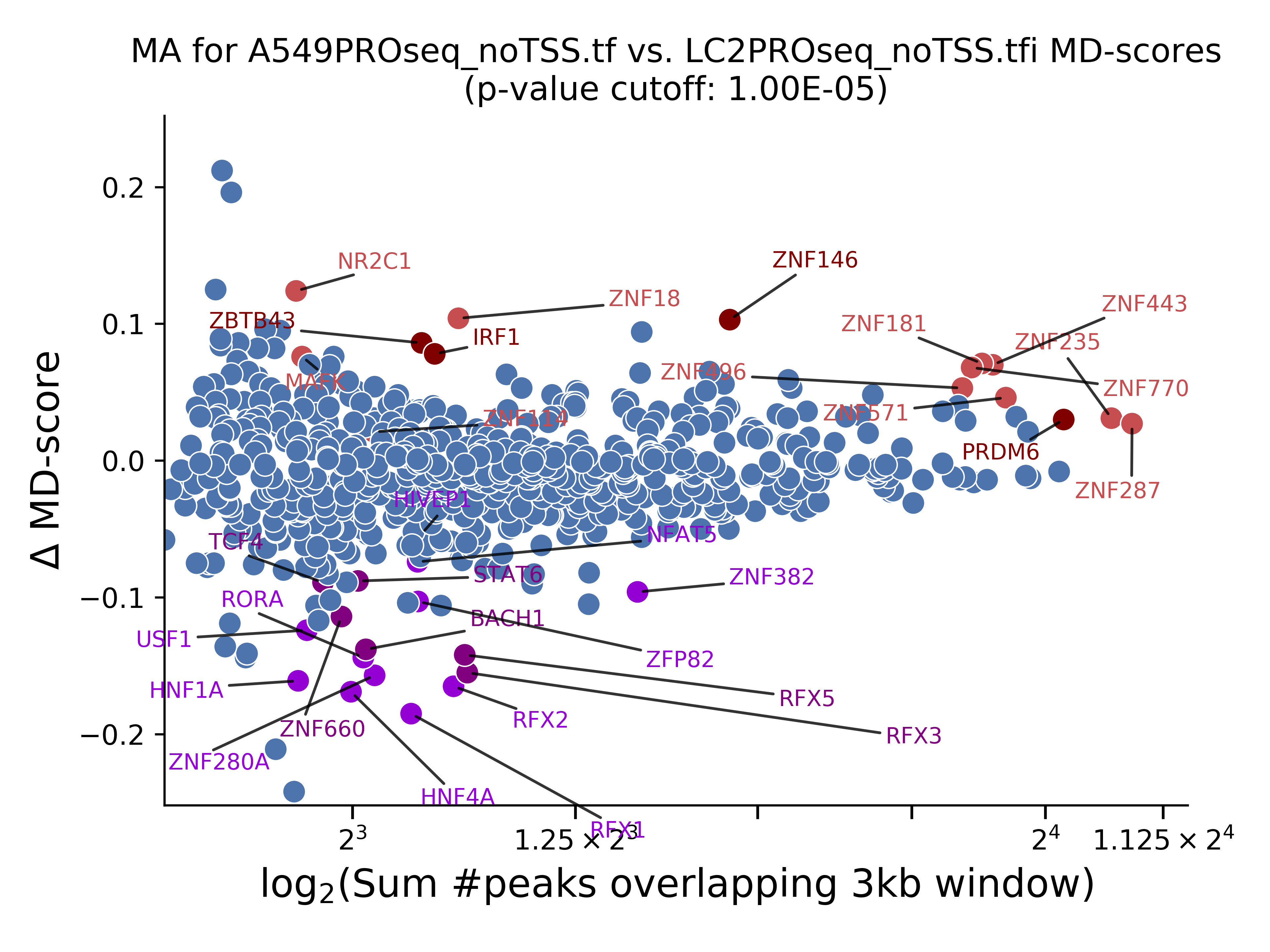

Supplement: Supplemental Data Set 1 [file jciinsight-6-144294-s076.zip › noTSS/best_curated_Human_TFs_p1e-6_grch38/A549_vs_LC2/MA_A549PROseq_noTSS.tfit_merged_to_LC2PROseq_noTSS.tfit_merged_md_score.png]

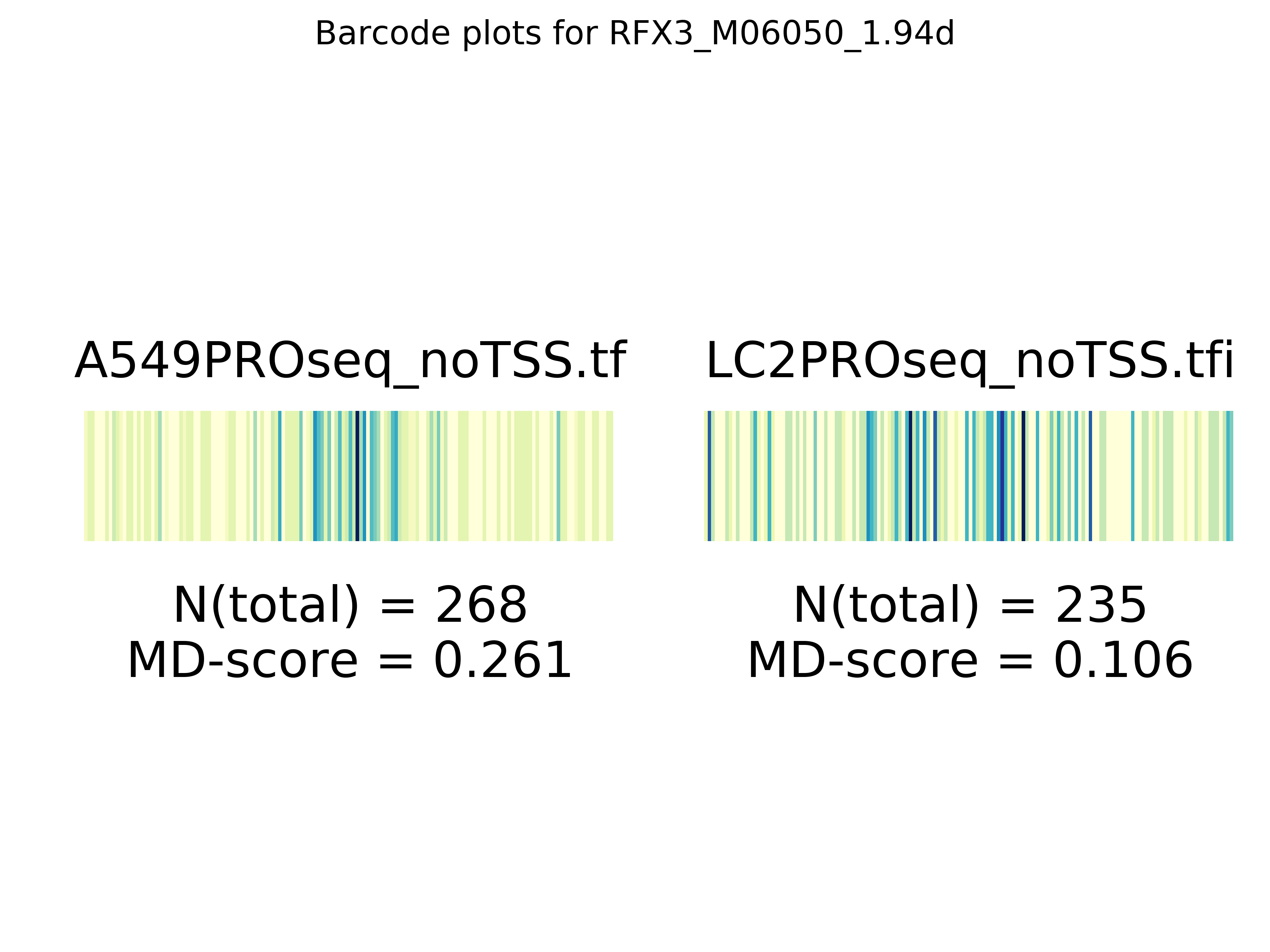

Supplement: Supplemental Data Set 1 [file jciinsight-6-144294-s076.zip › noTSS/best_curated_Human_TFs_p1e-6_grch38/A549_vs_LC2/RFX3_M06050_1.94d_barcode_A549PROseq_noTSS.tfit_merged_vs_LC2PROseq_noTSS.tfit_merged.png]

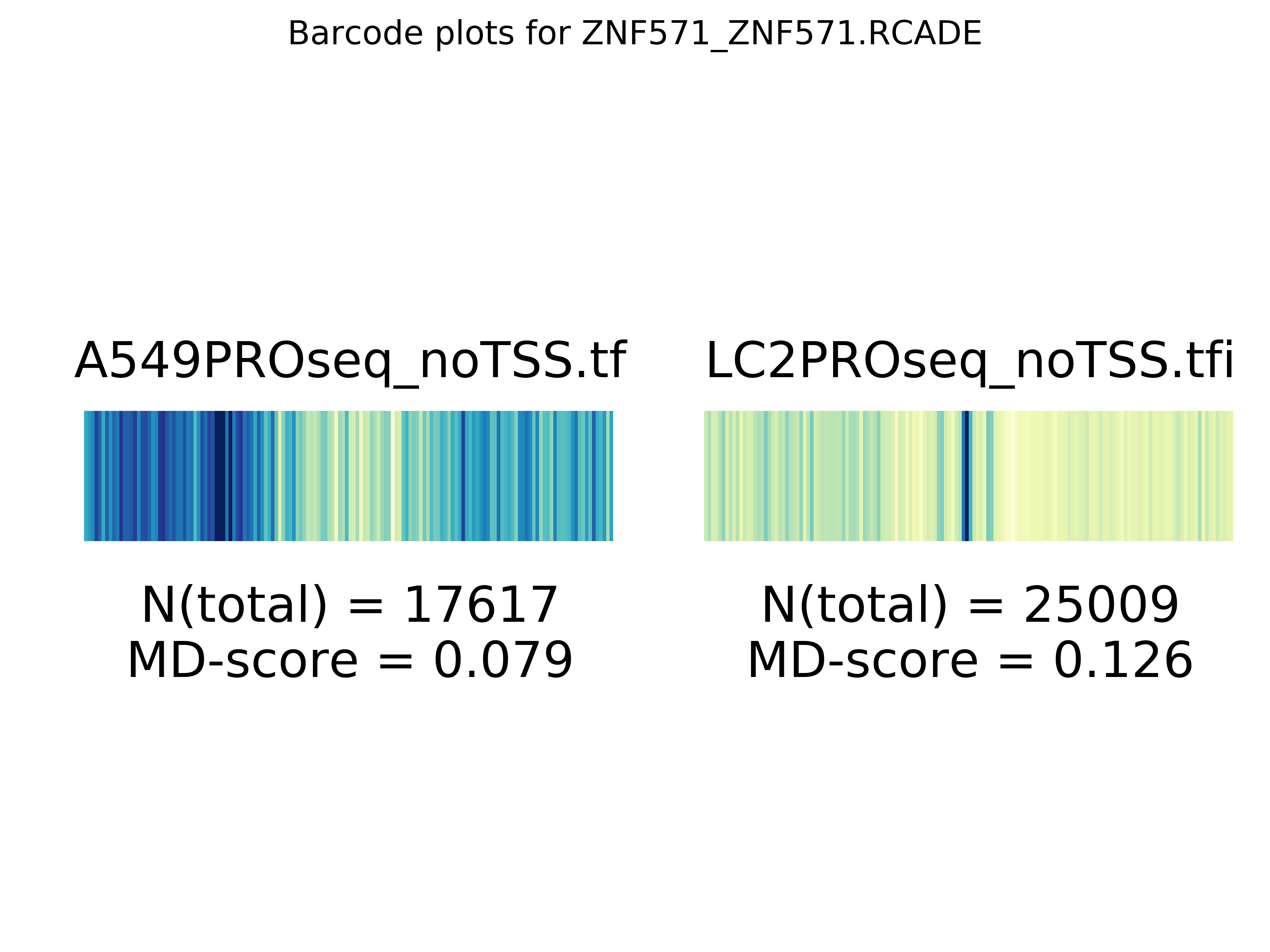

Supplement: Supplemental Data Set 1 [file jciinsight-6-144294-s076.zip › noTSS/best_curated_Human_TFs_p1e-6_grch38/A549_vs_LC2/ZNF571_ZNF571.RCADE_barcode_A549PROseq_noTSS.tfit_merged_vs_LC2PROseq_noTSS.tfit_merged.png]

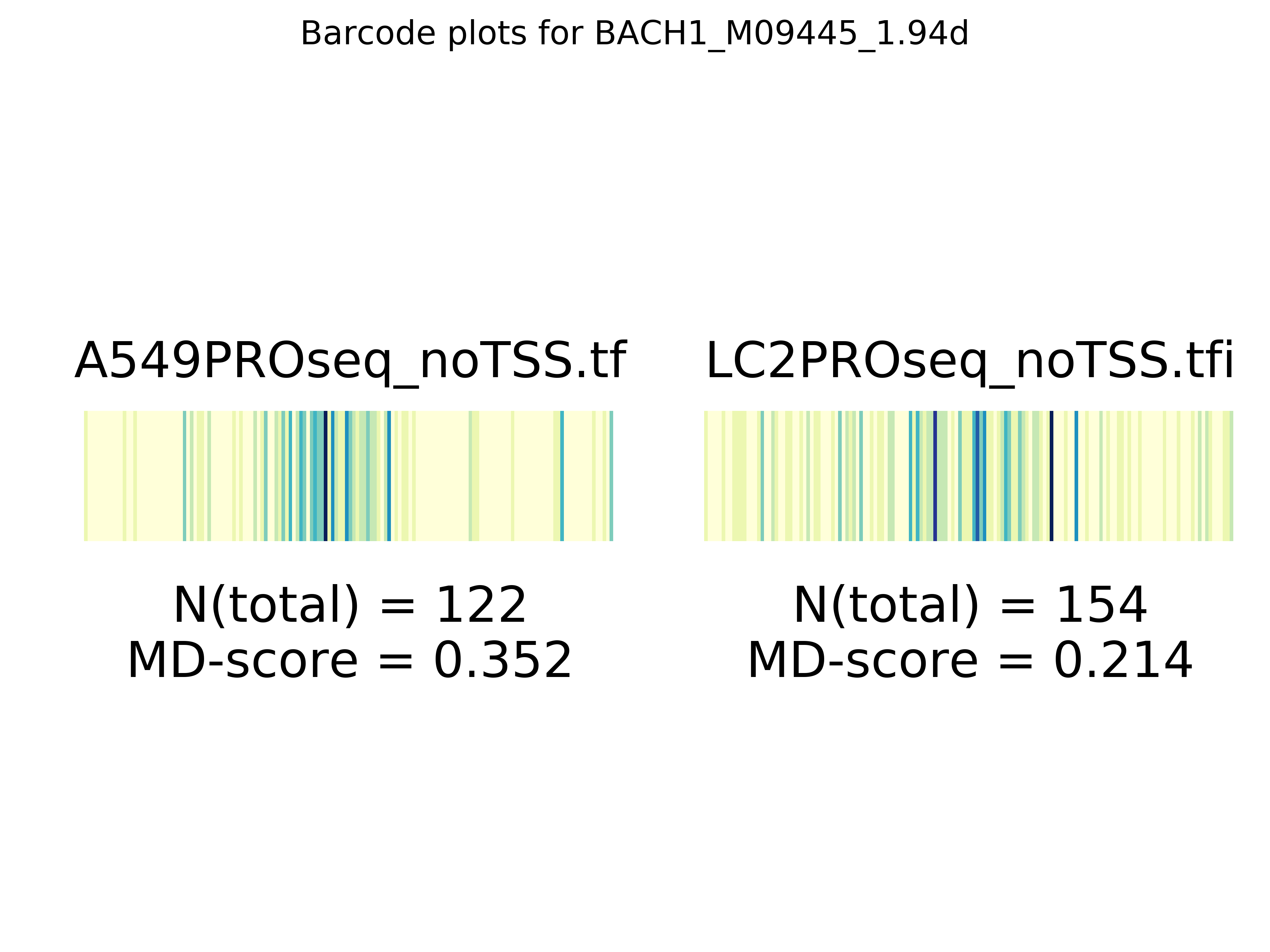

Supplement: Supplemental Data Set 1 [file jciinsight-6-144294-s076.zip › noTSS/best_curated_Human_TFs_p1e-6_grch38/A549_vs_LC2/BACH1_M09445_1.94d_barcode_A549PROseq_noTSS.tfit_merged_vs_LC2PROseq_noTSS.tfit_merged.png]

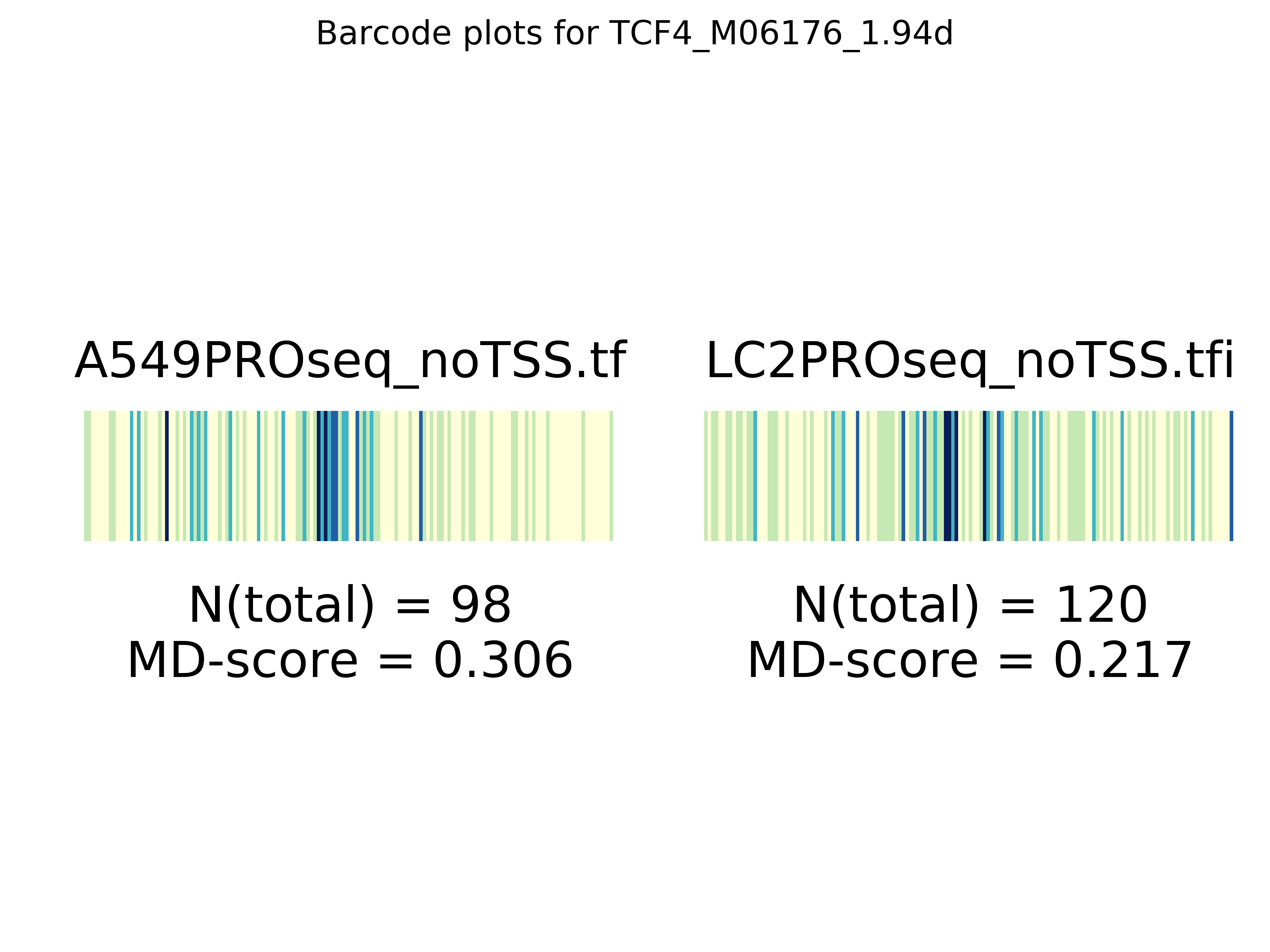

Supplement: Supplemental Data Set 1 [file jciinsight-6-144294-s076.zip › noTSS/best_curated_Human_TFs_p1e-6_grch38/A549_vs_LC2/TCF4_M06176_1.94d_barcode_A549PROseq_noTSS.tfit_merged_vs_LC2PROseq_noTSS.tfit_merged.png]

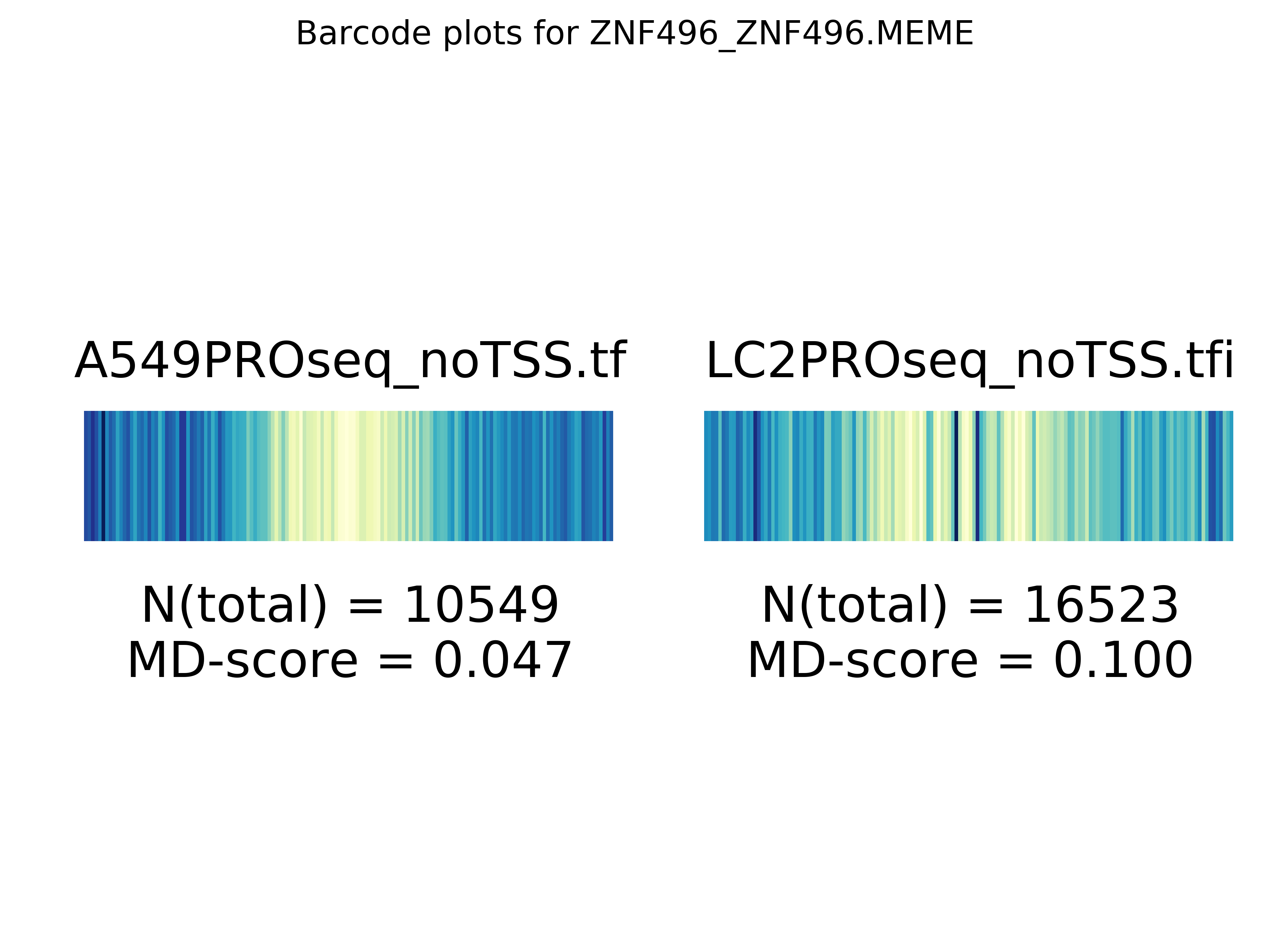

Supplement: Supplemental Data Set 1 [file jciinsight-6-144294-s076.zip › noTSS/best_curated_Human_TFs_p1e-6_grch38/A549_vs_LC2/ZNF496_ZNF496.MEME_barcode_A549PROseq_noTSS.tfit_merged_vs_LC2PROseq_noTSS.tfit_merged.png]

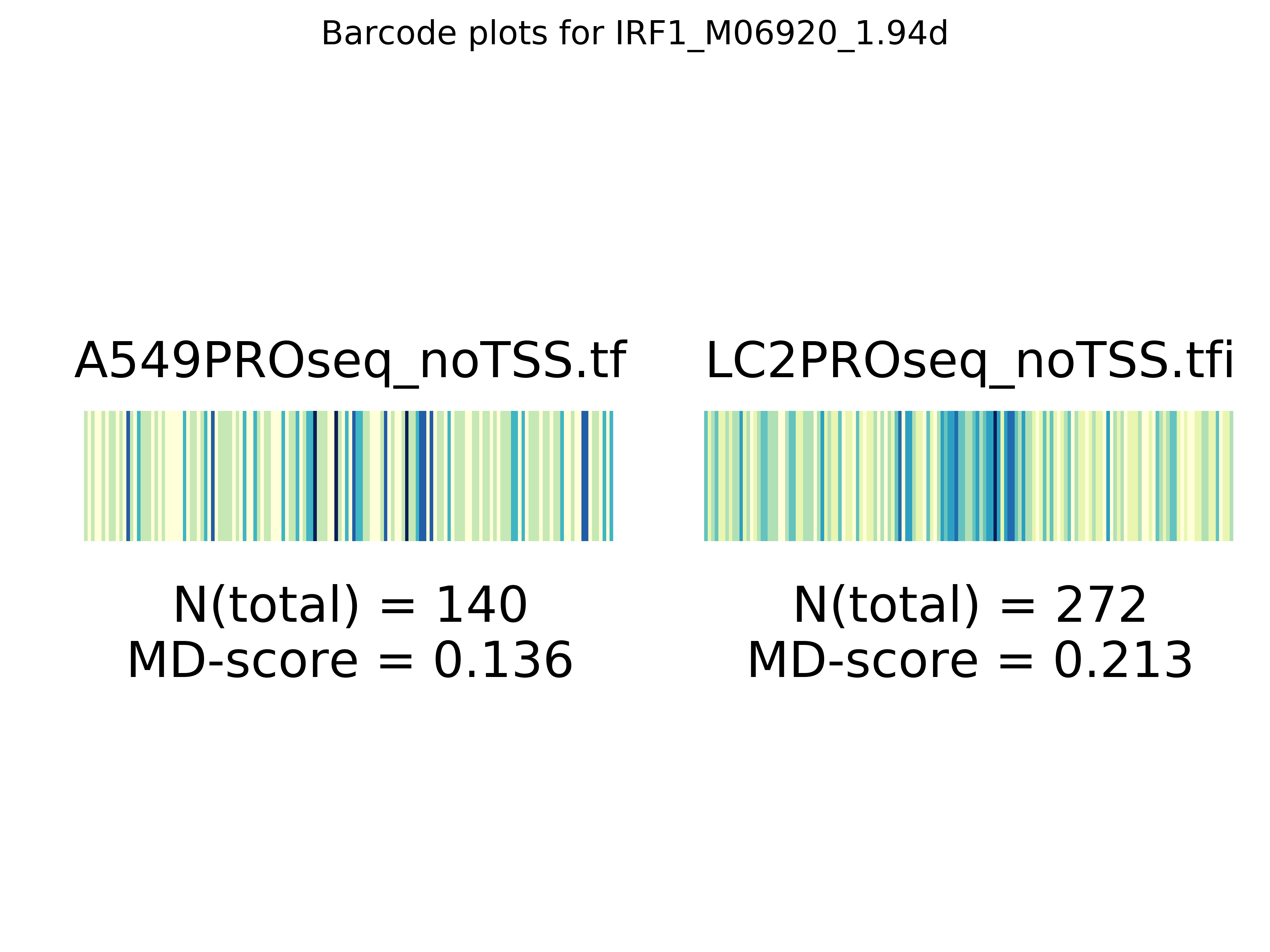

Supplement: Supplemental Data Set 1 [file jciinsight-6-144294-s076.zip › noTSS/best_curated_Human_TFs_p1e-6_grch38/A549_vs_LC2/IRF1_M06920_1.94d_barcode_A549PROseq_noTSS.tfit_merged_vs_LC2PROseq_noTSS.tfit_merged.png]

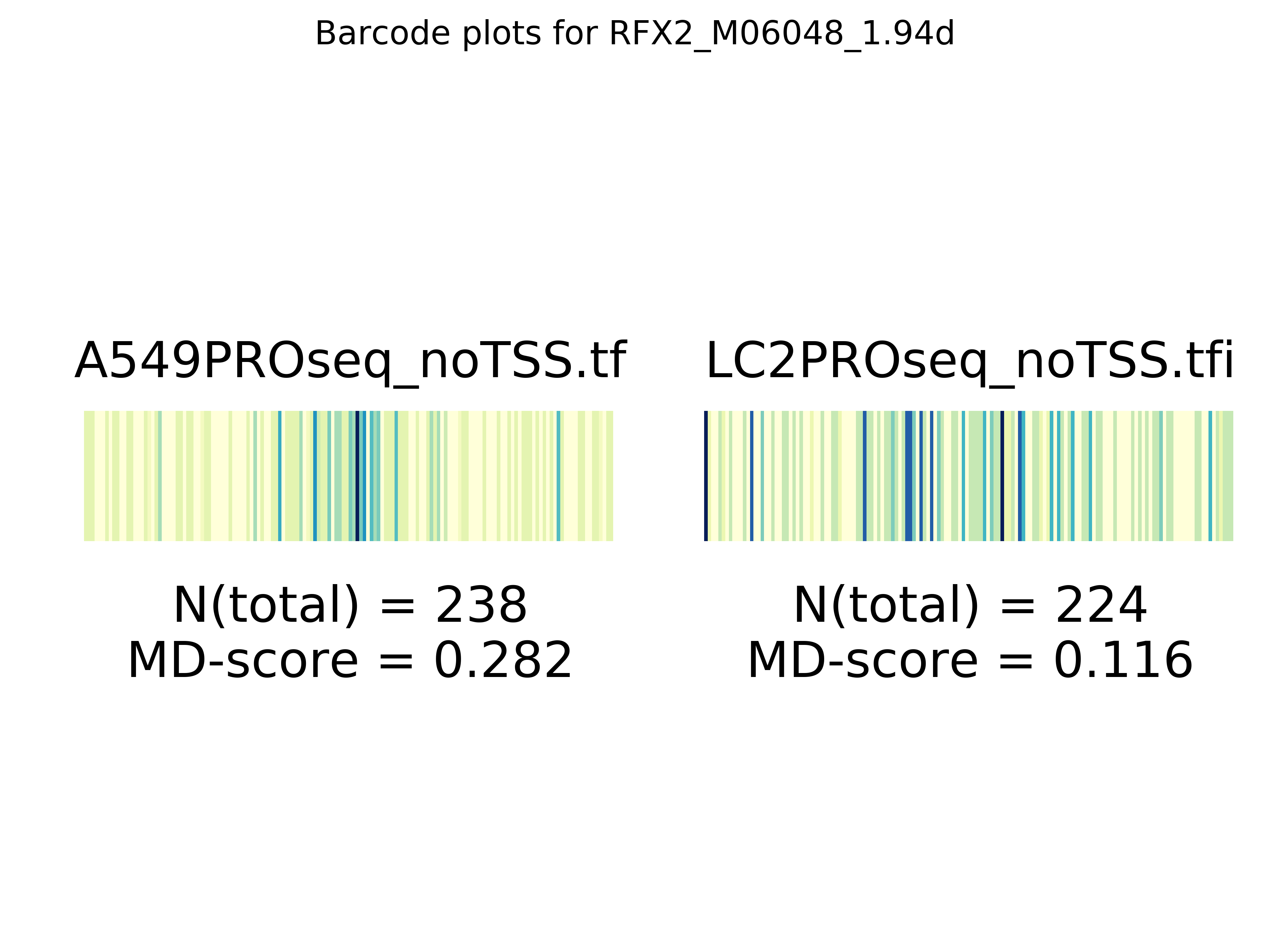

Supplement: Supplemental Data Set 1 [file jciinsight-6-144294-s076.zip › noTSS/best_curated_Human_TFs_p1e-6_grch38/A549_vs_LC2/RFX2_M06048_1.94d_barcode_A549PROseq_noTSS.tfit_merged_vs_LC2PROseq_noTSS.tfit_merged.png]

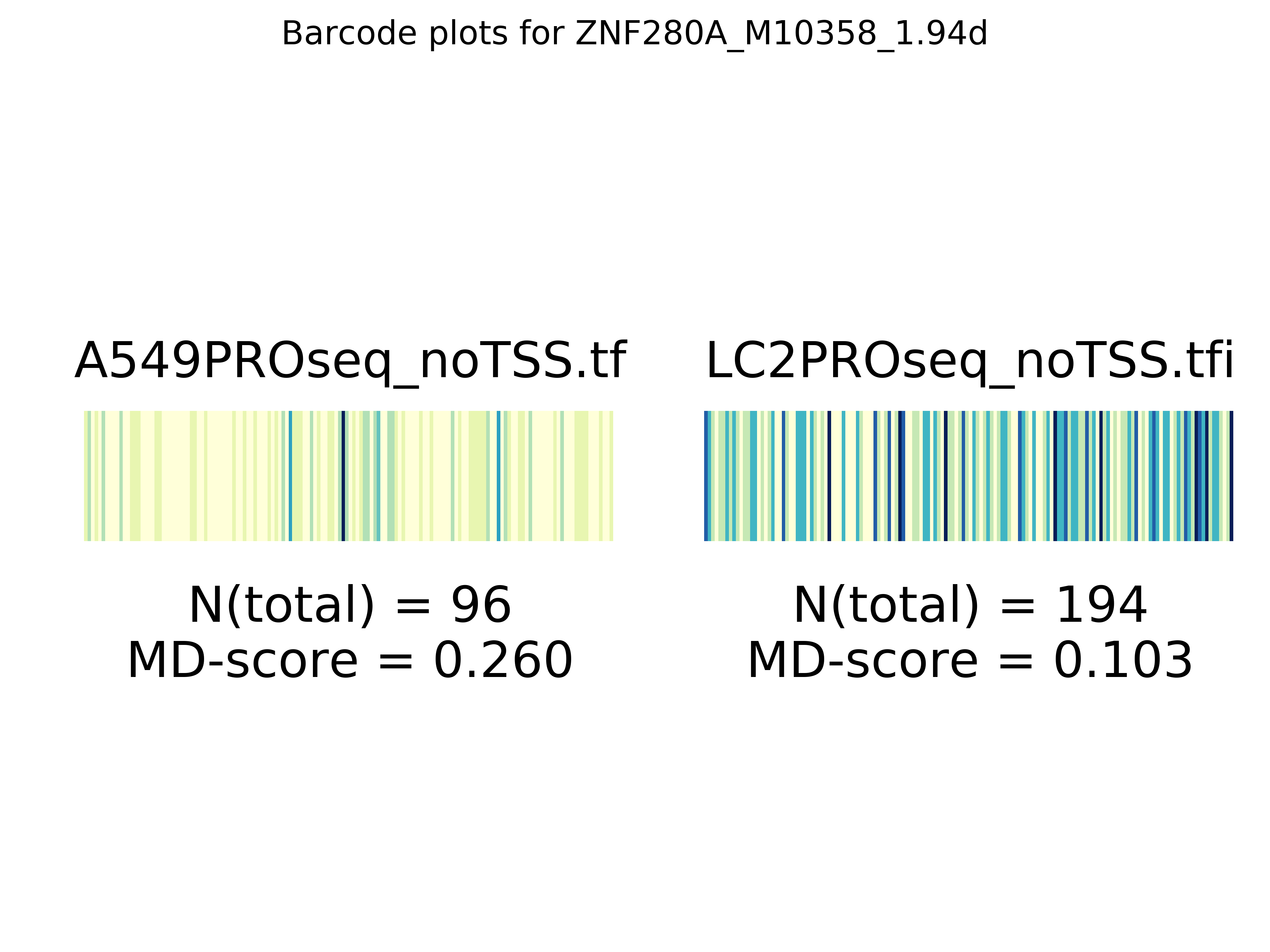

Supplement: Supplemental Data Set 1 [file jciinsight-6-144294-s076.zip › noTSS/best_curated_Human_TFs_p1e-6_grch38/A549_vs_LC2/ZNF280A_M10358_1.94d_barcode_A549PROseq_noTSS.tfit_merged_vs_LC2PROseq_noTSS.tfit_merged.png]

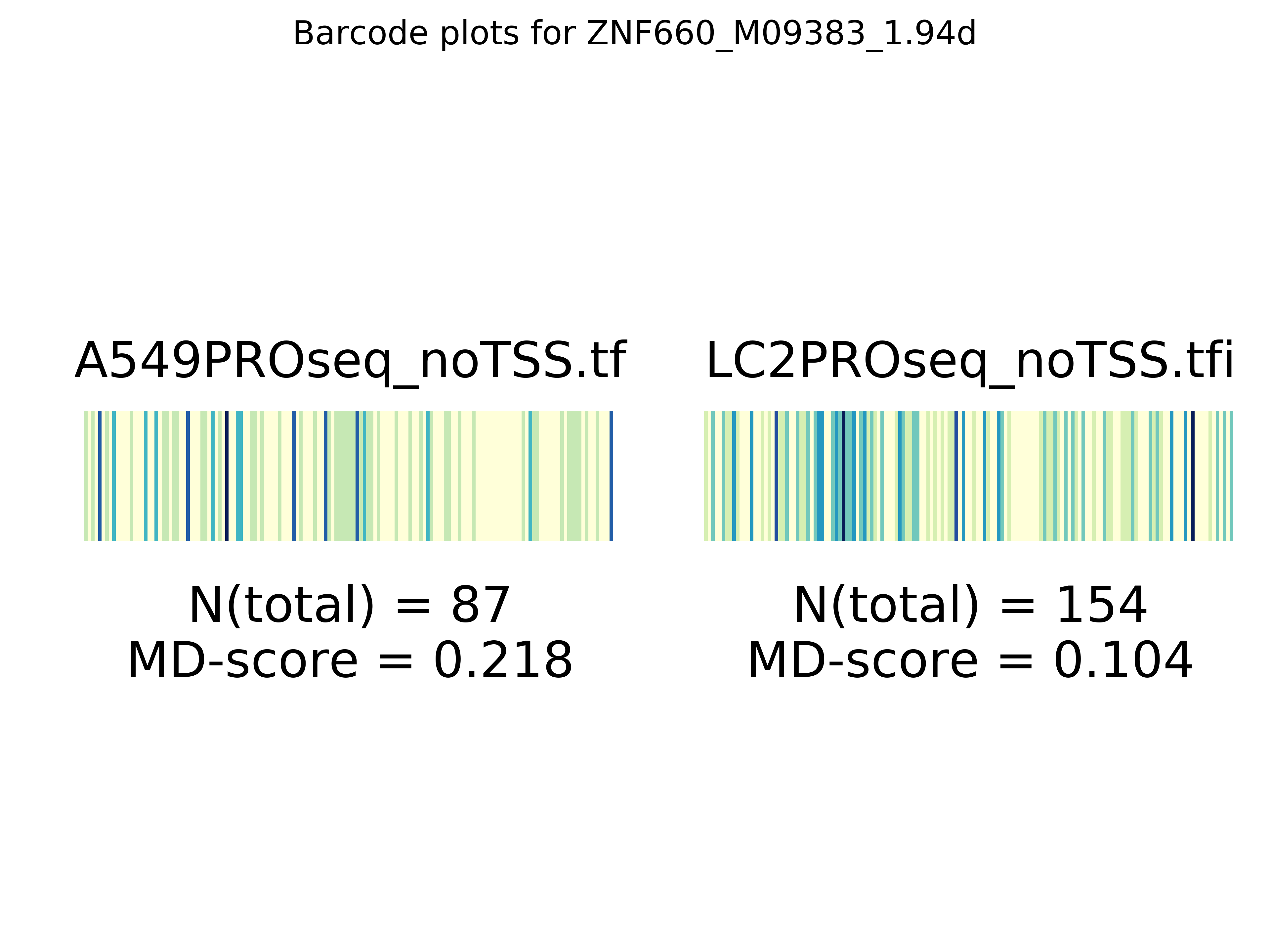

Supplement: Supplemental Data Set 1 [file jciinsight-6-144294-s076.zip › noTSS/best_curated_Human_TFs_p1e-6_grch38/A549_vs_LC2/ZNF660_M09383_1.94d_barcode_A549PROseq_noTSS.tfit_merged_vs_LC2PROseq_noTSS.tfit_merged.png]

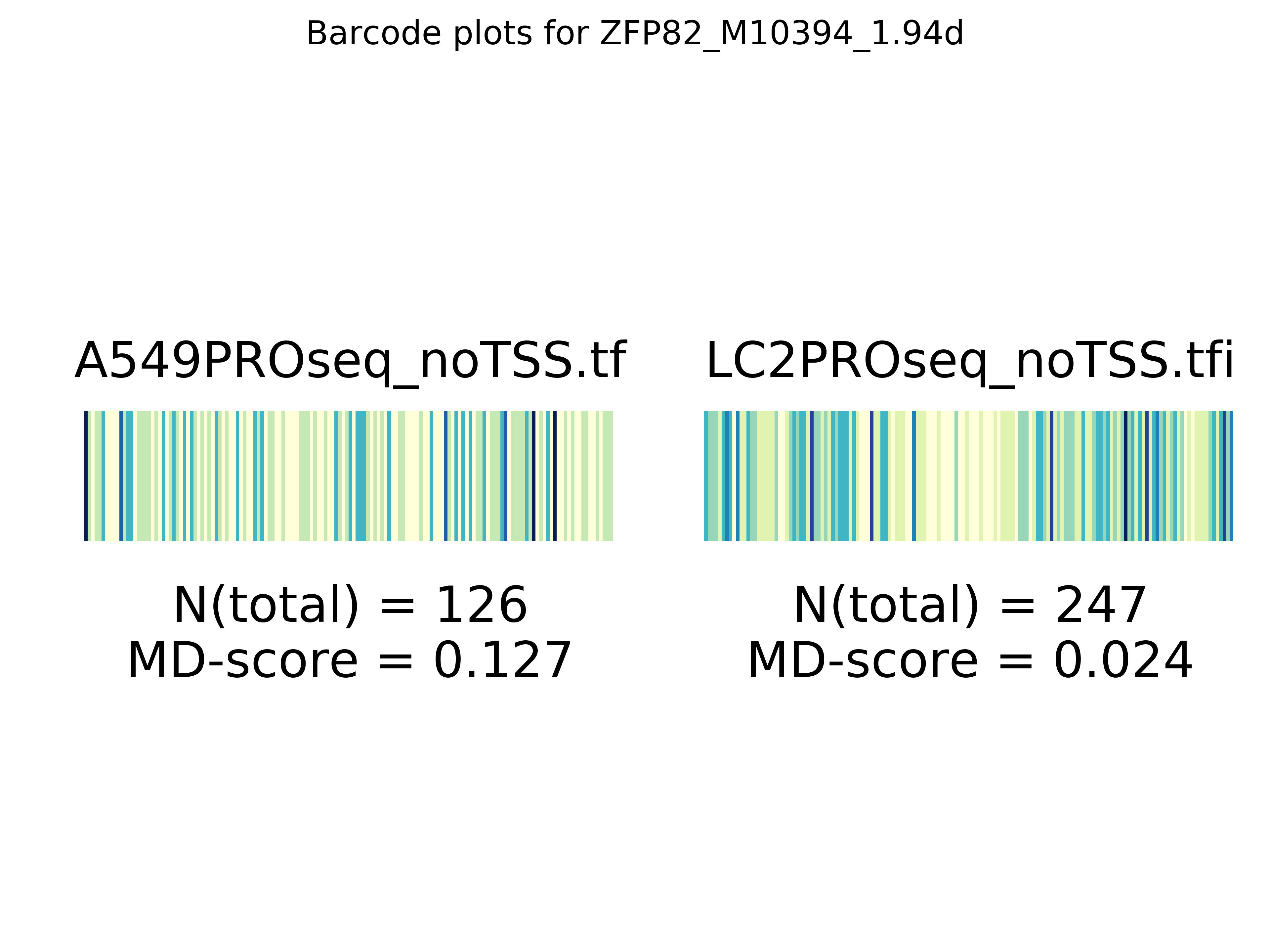

Supplement: Supplemental Data Set 1 [file jciinsight-6-144294-s076.zip › noTSS/best_curated_Human_TFs_p1e-6_grch38/A549_vs_LC2/ZFP82_M10394_1.94d_barcode_A549PROseq_noTSS.tfit_merged_vs_LC2PROseq_noTSS.tfit_merged.png]

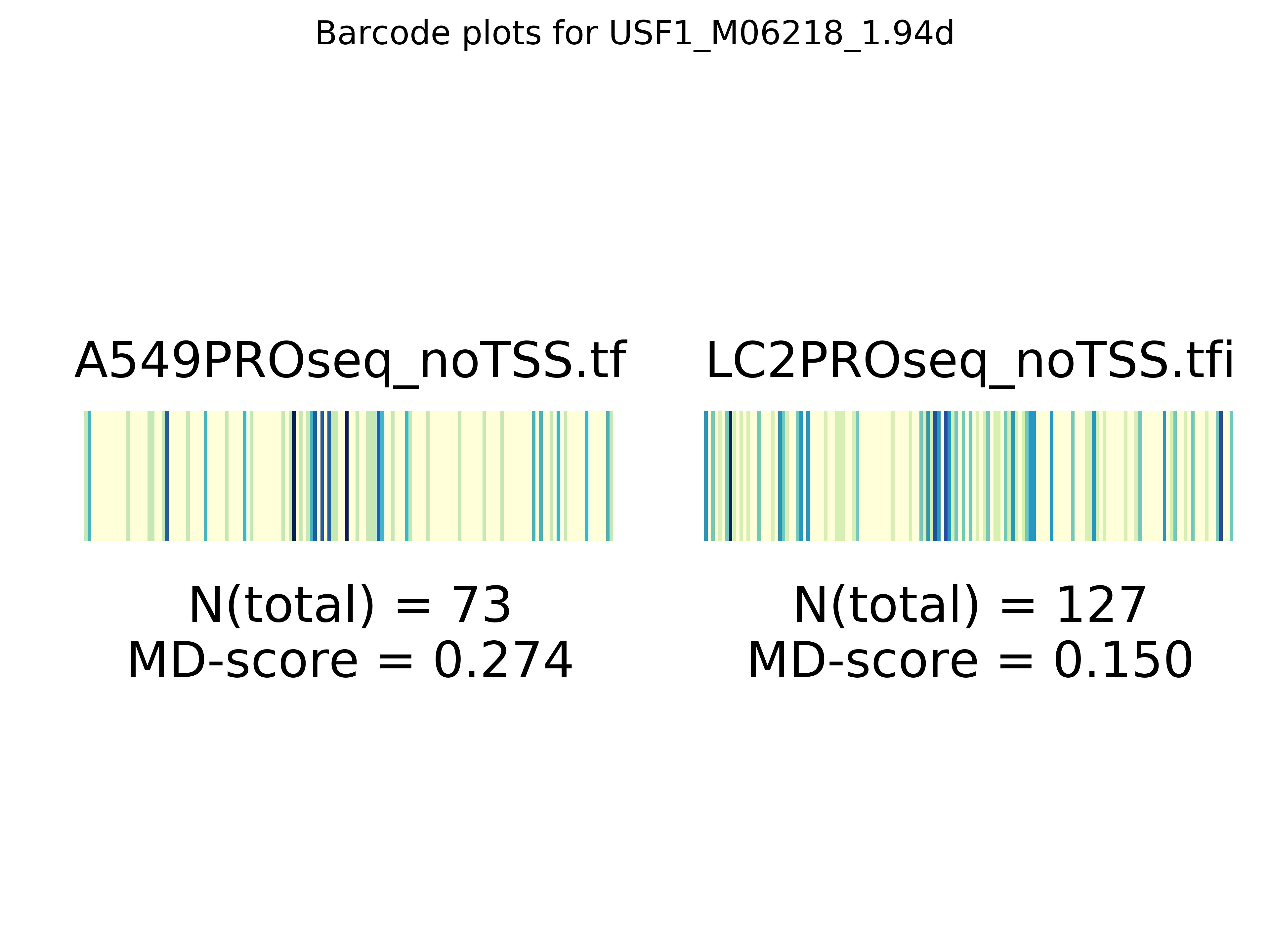

Supplement: Supplemental Data Set 1 [file jciinsight-6-144294-s076.zip › noTSS/best_curated_Human_TFs_p1e-6_grch38/A549_vs_LC2/USF1_M06218_1.94d_barcode_A549PROseq_noTSS.tfit_merged_vs_LC2PROseq_noTSS.tfit_merged.png]

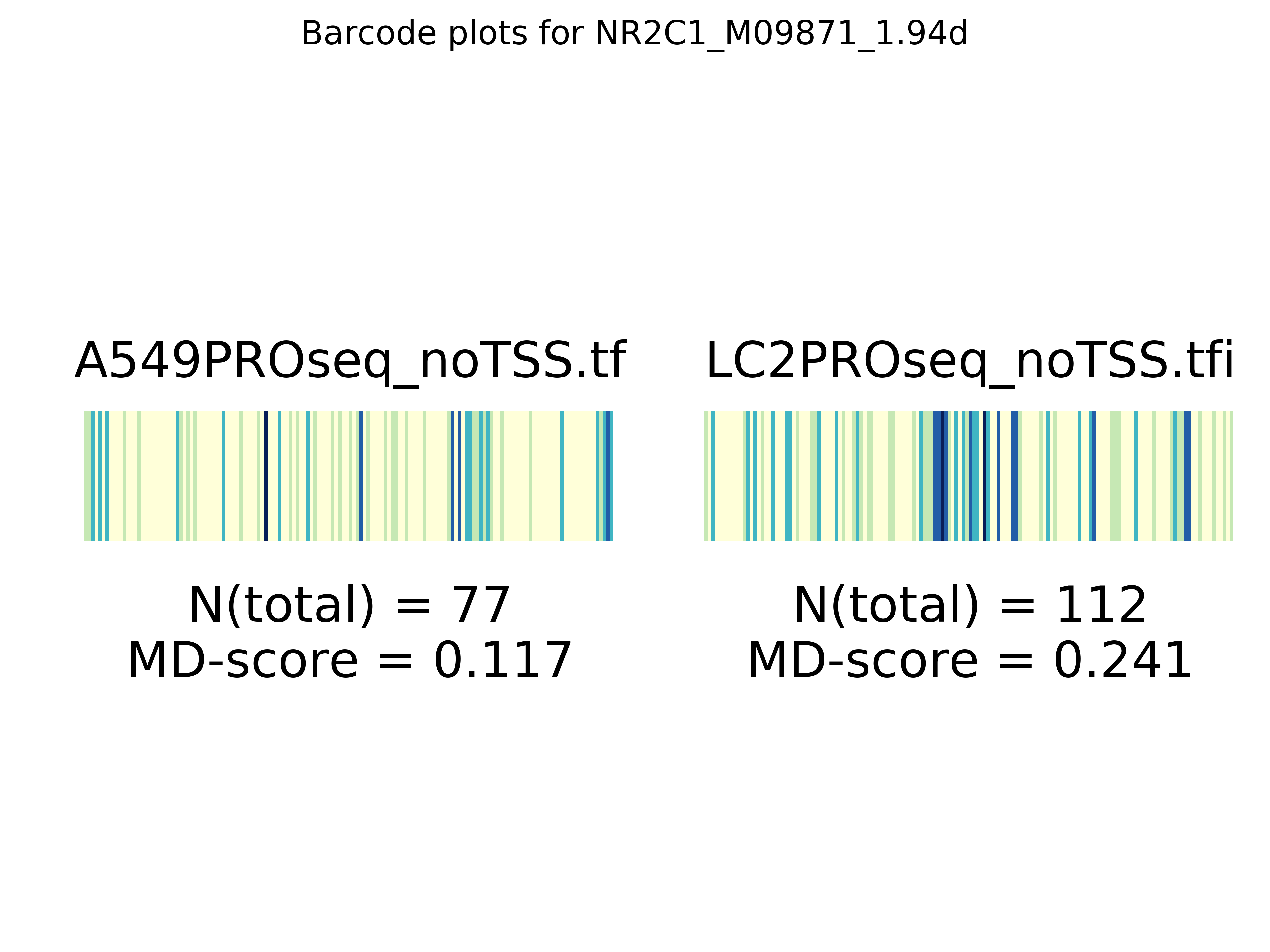

Supplement: Supplemental Data Set 1 [file jciinsight-6-144294-s076.zip › noTSS/best_curated_Human_TFs_p1e-6_grch38/A549_vs_LC2/NR2C1_M09871_1.94d_barcode_A549PROseq_noTSS.tfit_merged_vs_LC2PROseq_noTSS.tfit_merged.png]

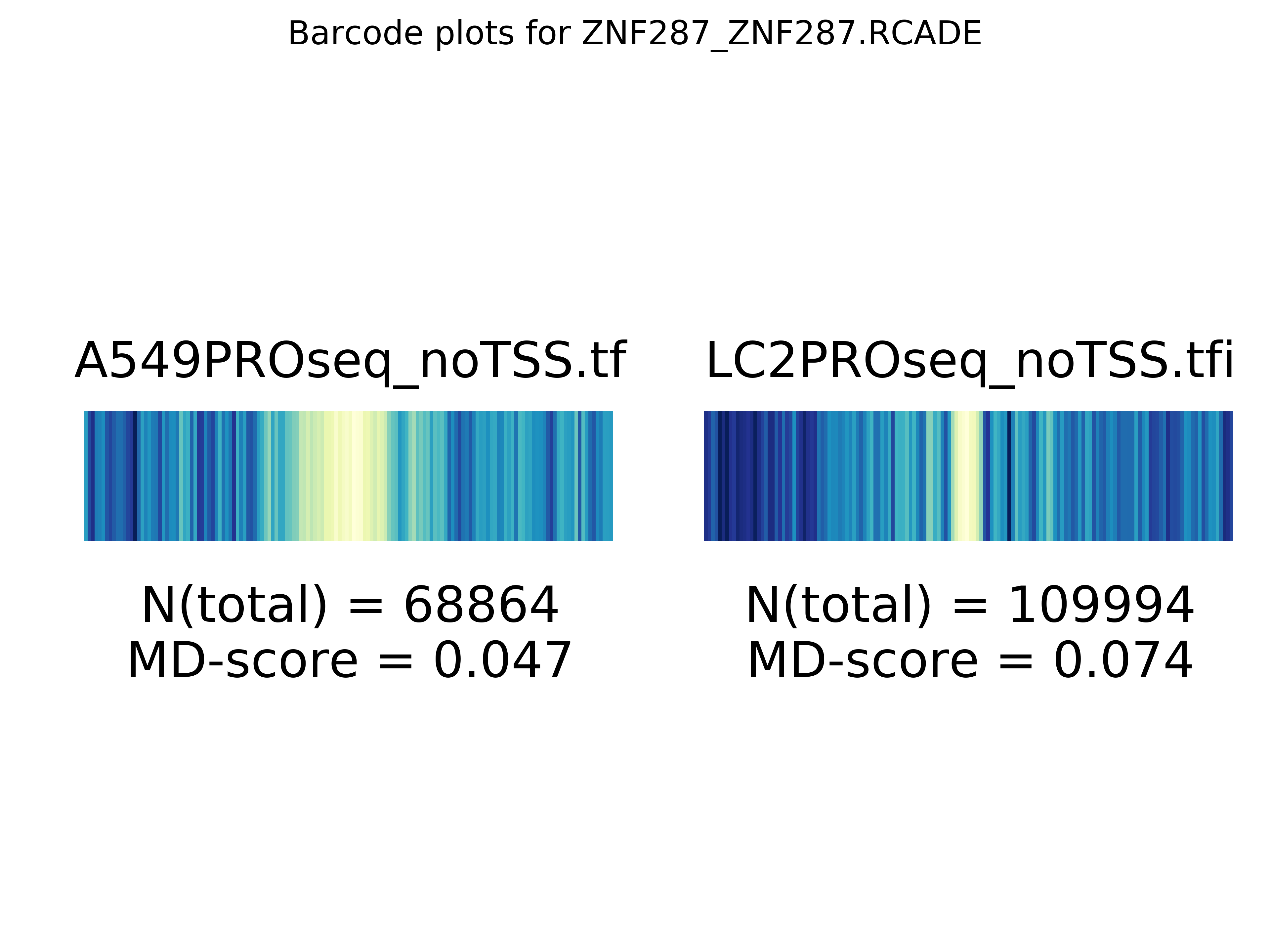

Supplement: Supplemental Data Set 1 [file jciinsight-6-144294-s076.zip › noTSS/best_curated_Human_TFs_p1e-6_grch38/A549_vs_LC2/ZNF287_ZNF287.RCADE_barcode_A549PROseq_noTSS.tfit_merged_vs_LC2PROseq_noTSS.tfit_merged.png]

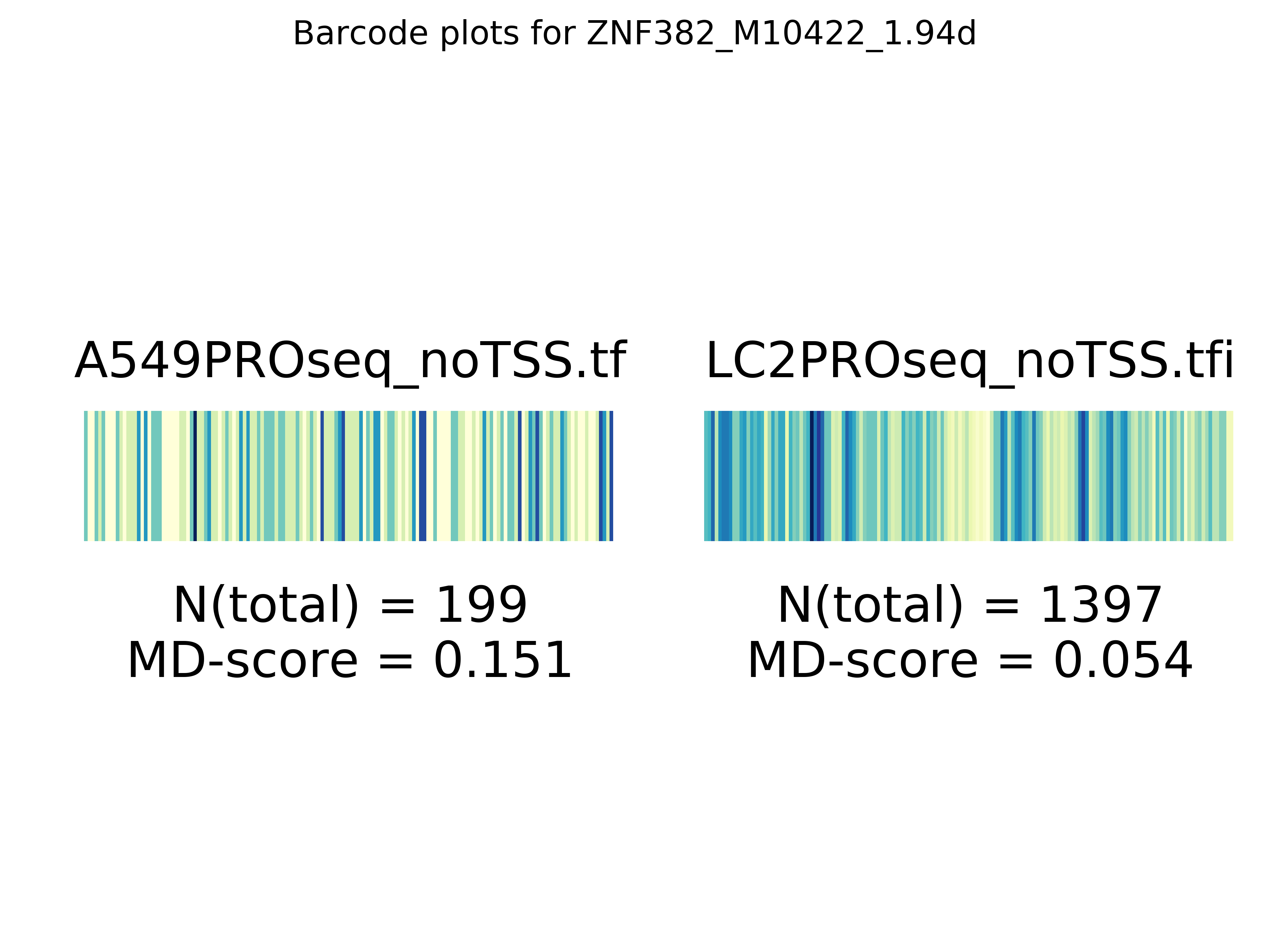

Supplement: Supplemental Data Set 1 [file jciinsight-6-144294-s076.zip › noTSS/best_curated_Human_TFs_p1e-6_grch38/A549_vs_LC2/ZNF382_M10422_1.94d_barcode_A549PROseq_noTSS.tfit_merged_vs_LC2PROseq_noTSS.tfit_merged.png]

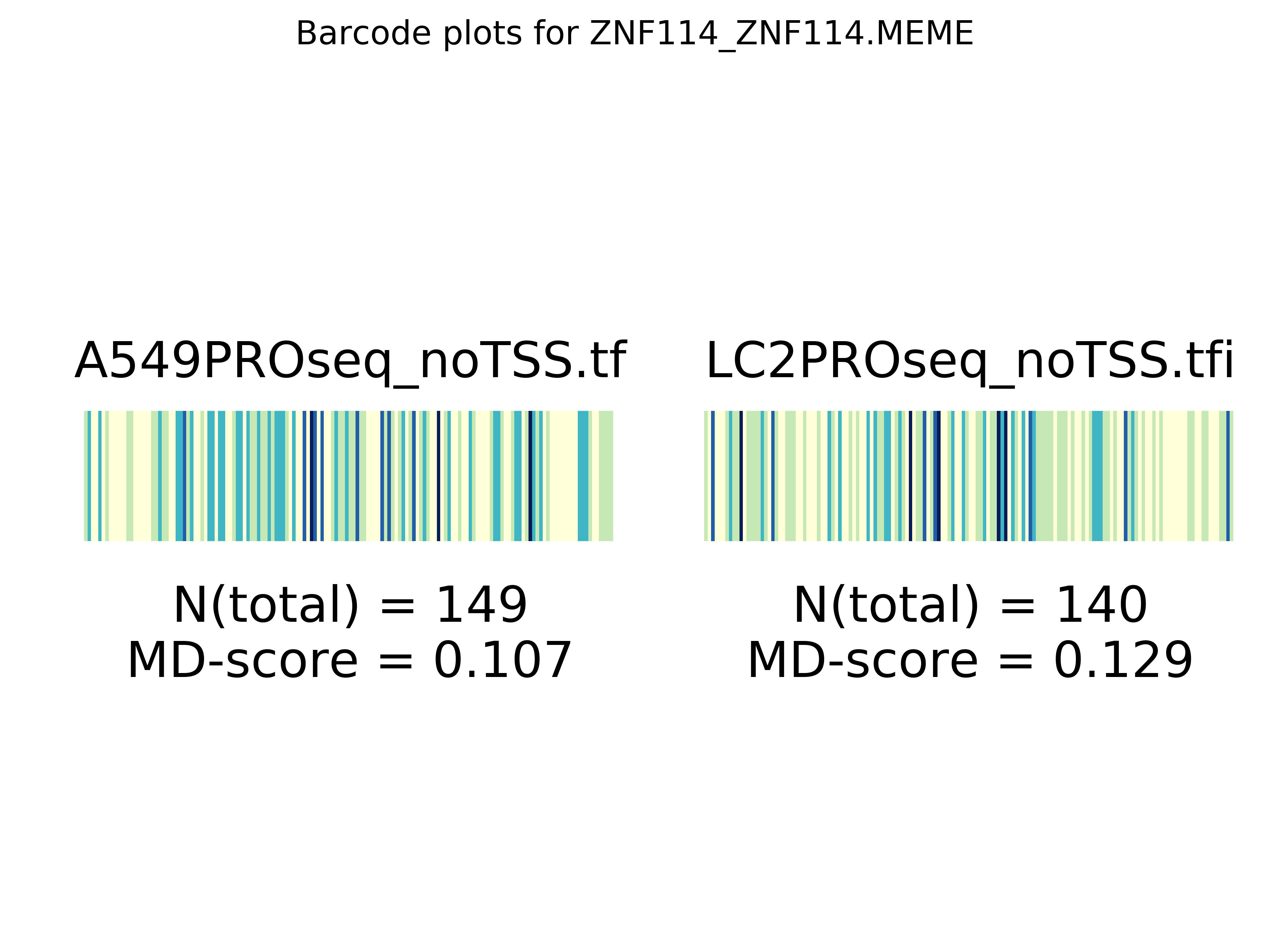

Supplement: Supplemental Data Set 1 [file jciinsight-6-144294-s076.zip › noTSS/best_curated_Human_TFs_p1e-6_grch38/A549_vs_LC2/ZNF114_ZNF114.MEME_barcode_A549PROseq_noTSS.tfit_merged_vs_LC2PROseq_noTSS.tfit_merged.png]

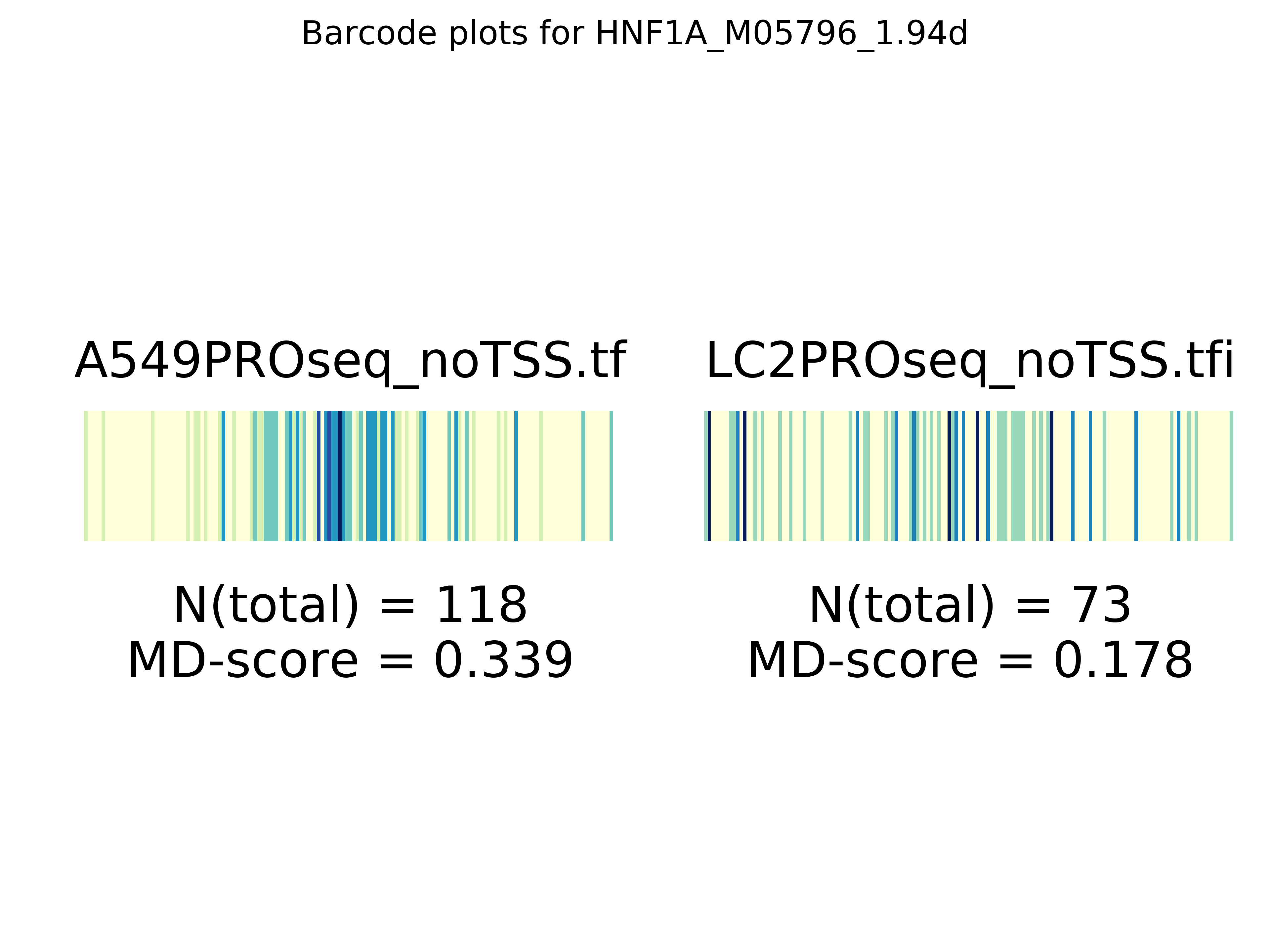

Supplement: Supplemental Data Set 1 [file jciinsight-6-144294-s076.zip › noTSS/best_curated_Human_TFs_p1e-6_grch38/A549_vs_LC2/HNF1A_M05796_1.94d_barcode_A549PROseq_noTSS.tfit_merged_vs_LC2PROseq_noTSS.tfit_merged.png]

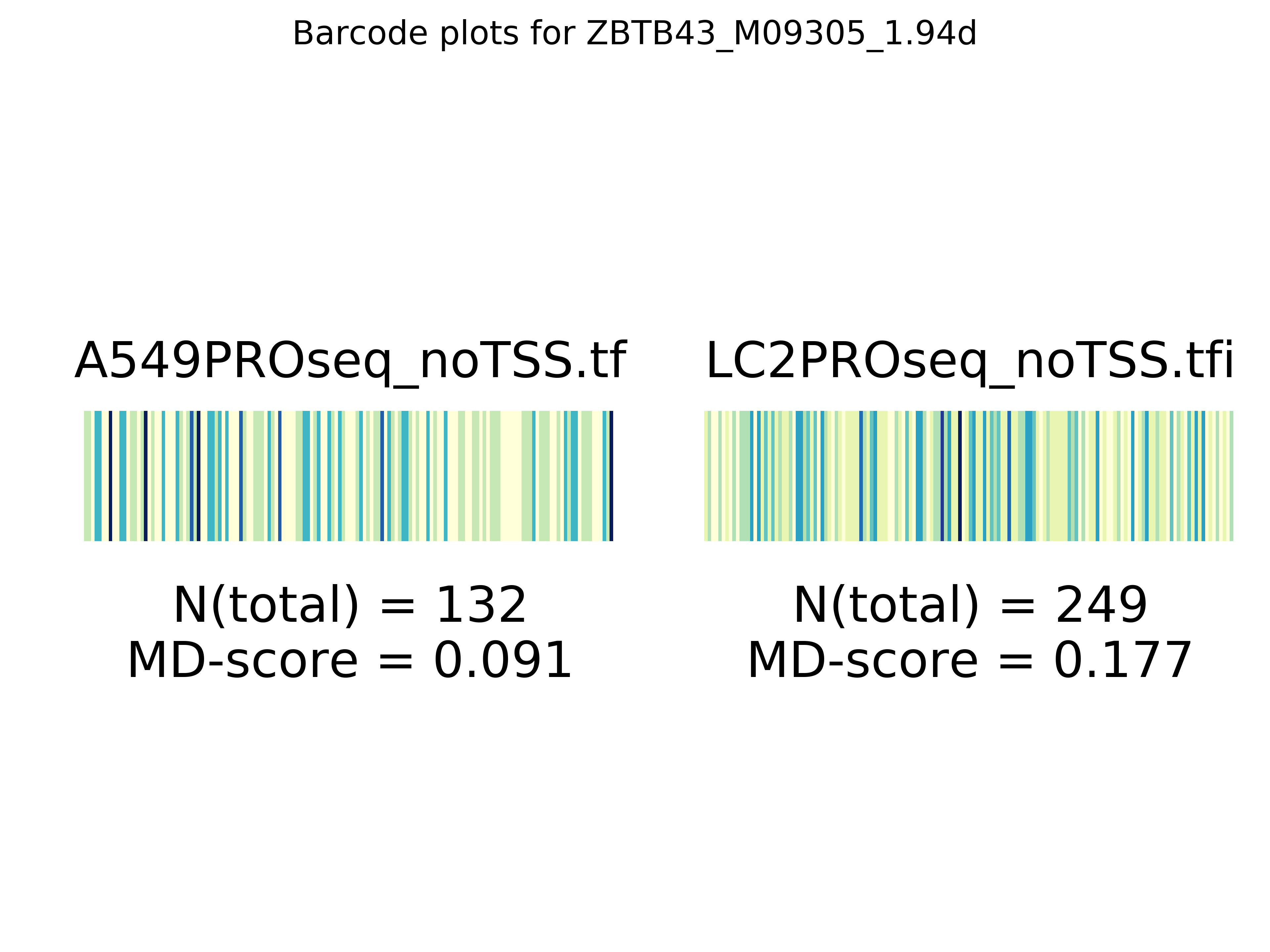

Supplement: Supplemental Data Set 1 [file jciinsight-6-144294-s076.zip › noTSS/best_curated_Human_TFs_p1e-6_grch38/A549_vs_LC2/ZBTB43_M09305_1.94d_barcode_A549PROseq_noTSS.tfit_merged_vs_LC2PROseq_noTSS.tfit_merged.png]

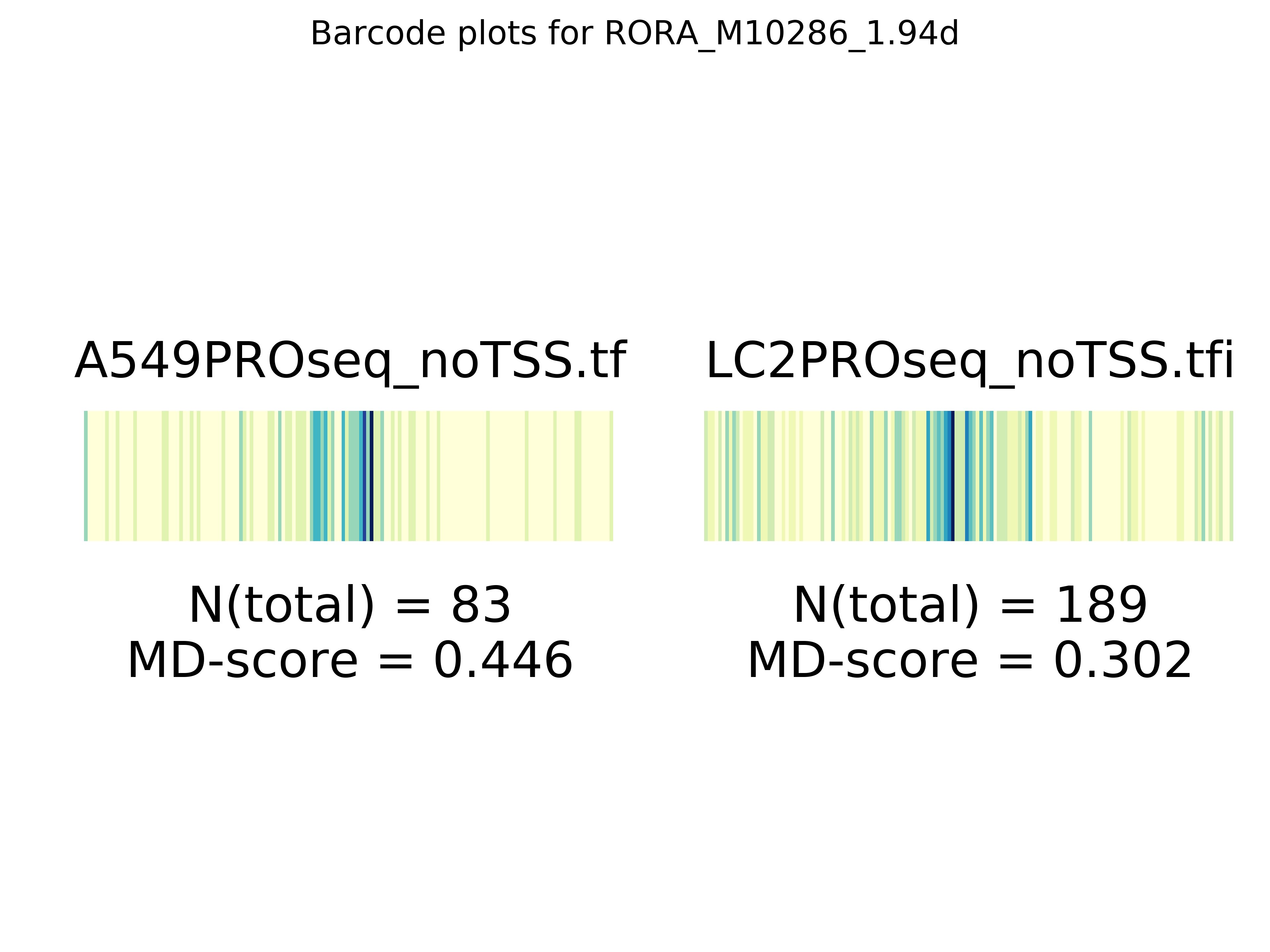

Supplement: Supplemental Data Set 1 [file jciinsight-6-144294-s076.zip › noTSS/best_curated_Human_TFs_p1e-6_grch38/A549_vs_LC2/RORA_M10286_1.94d_barcode_A549PROseq_noTSS.tfit_merged_vs_LC2PROseq_noTSS.tfit_merged.png]

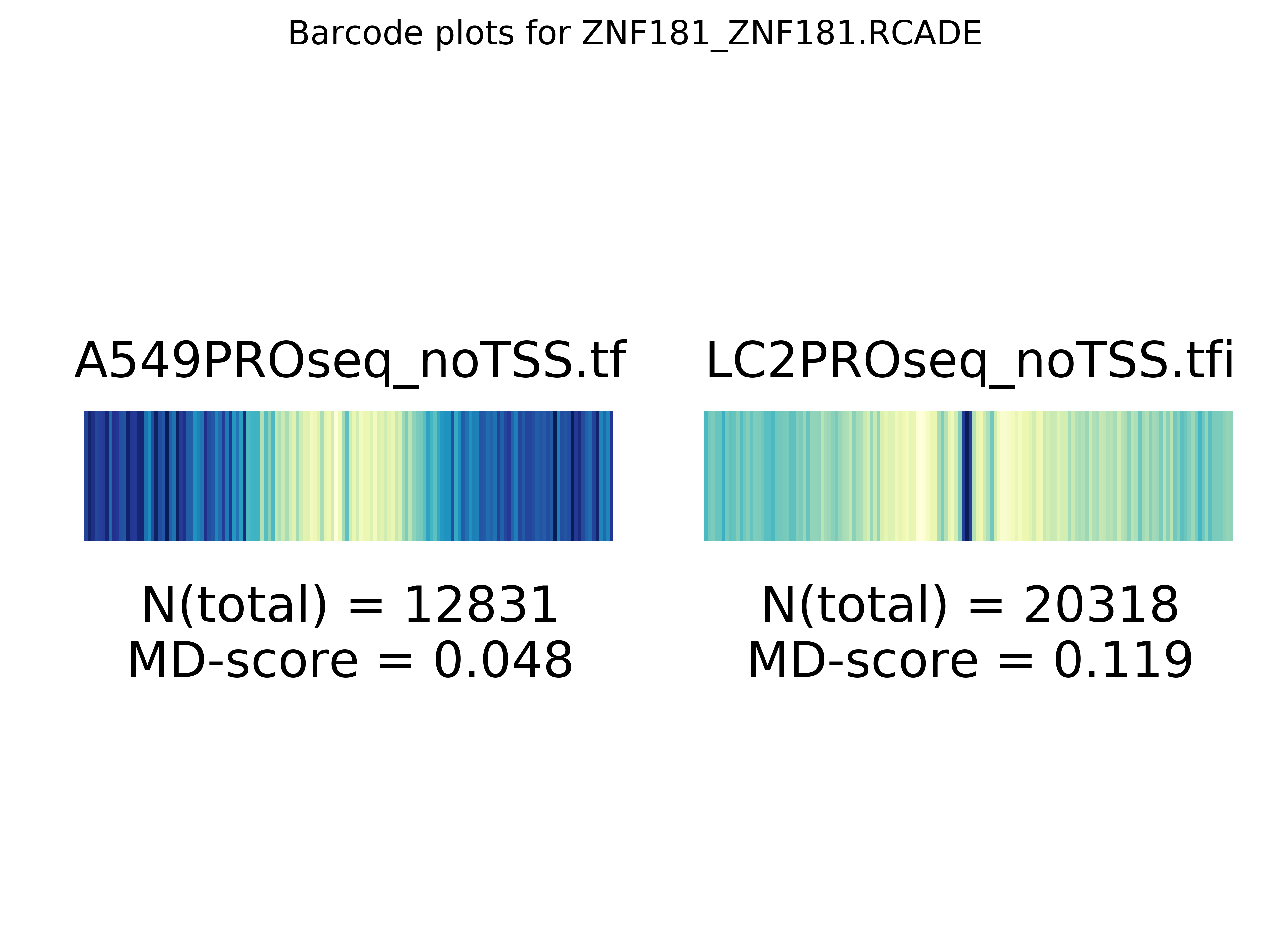

Supplement: Supplemental Data Set 1 [file jciinsight-6-144294-s076.zip › noTSS/best_curated_Human_TFs_p1e-6_grch38/A549_vs_LC2/ZNF181_ZNF181.RCADE_barcode_A549PROseq_noTSS.tfit_merged_vs_LC2PROseq_noTSS.tfit_merged.png]

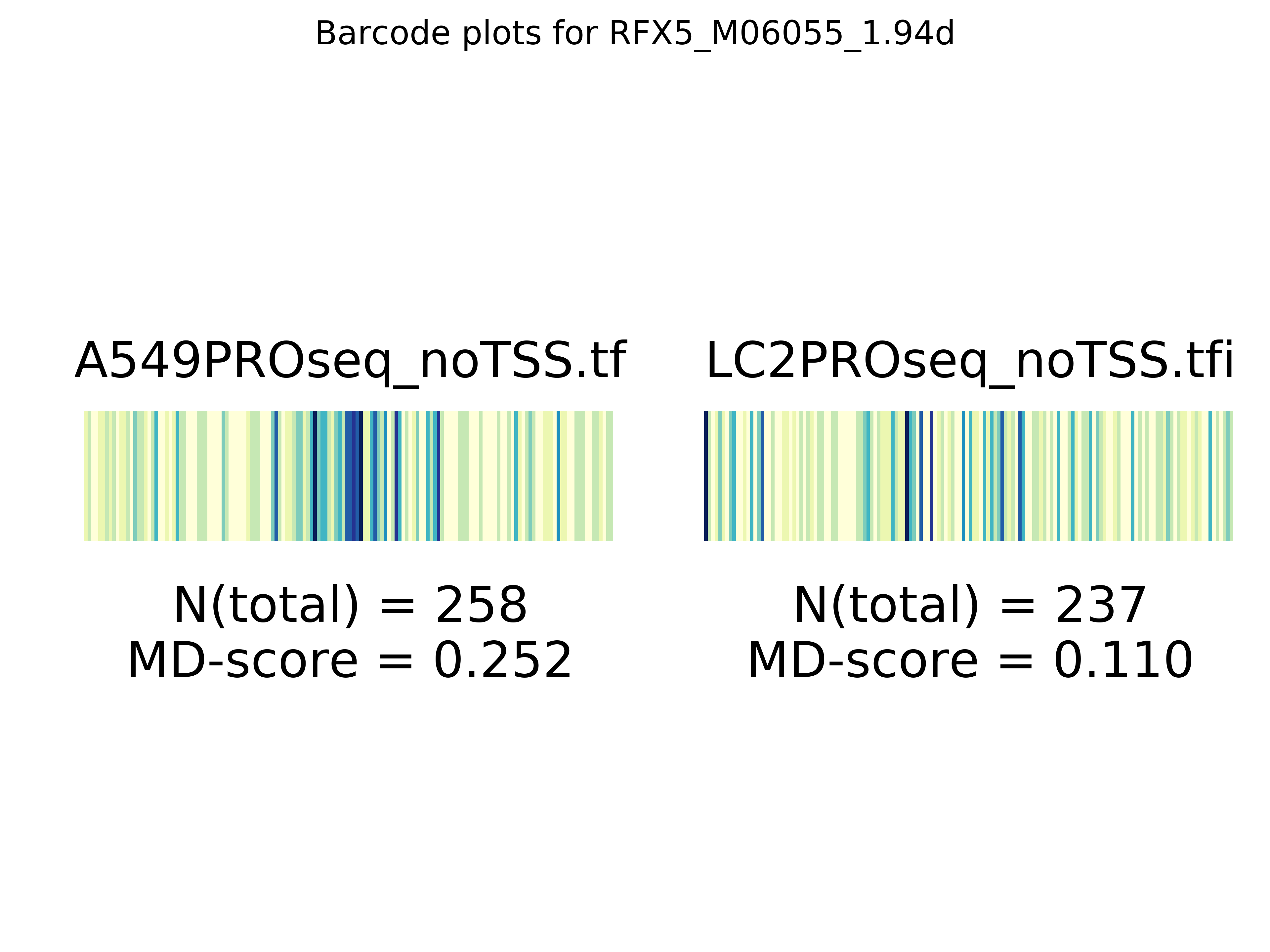

Supplement: Supplemental Data Set 1 [file jciinsight-6-144294-s076.zip › noTSS/best_curated_Human_TFs_p1e-6_grch38/A549_vs_LC2/RFX5_M06055_1.94d_barcode_A549PROseq_noTSS.tfit_merged_vs_LC2PROseq_noTSS.tfit_merged.png]

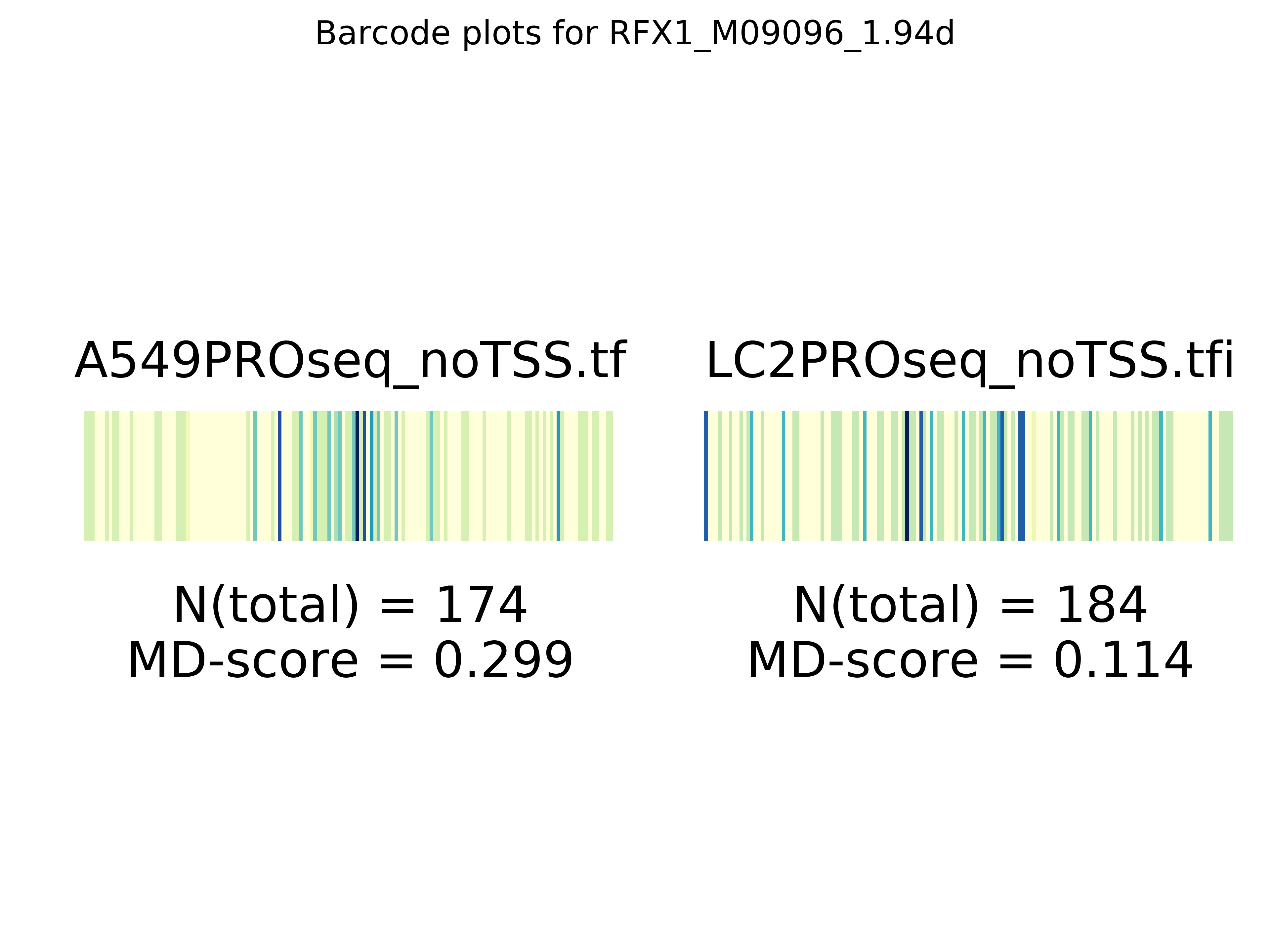

Supplement: Supplemental Data Set 1 [file jciinsight-6-144294-s076.zip › noTSS/best_curated_Human_TFs_p1e-6_grch38/A549_vs_LC2/RFX1_M09096_1.94d_barcode_A549PROseq_noTSS.tfit_merged_vs_LC2PROseq_noTSS.tfit_merged.png]

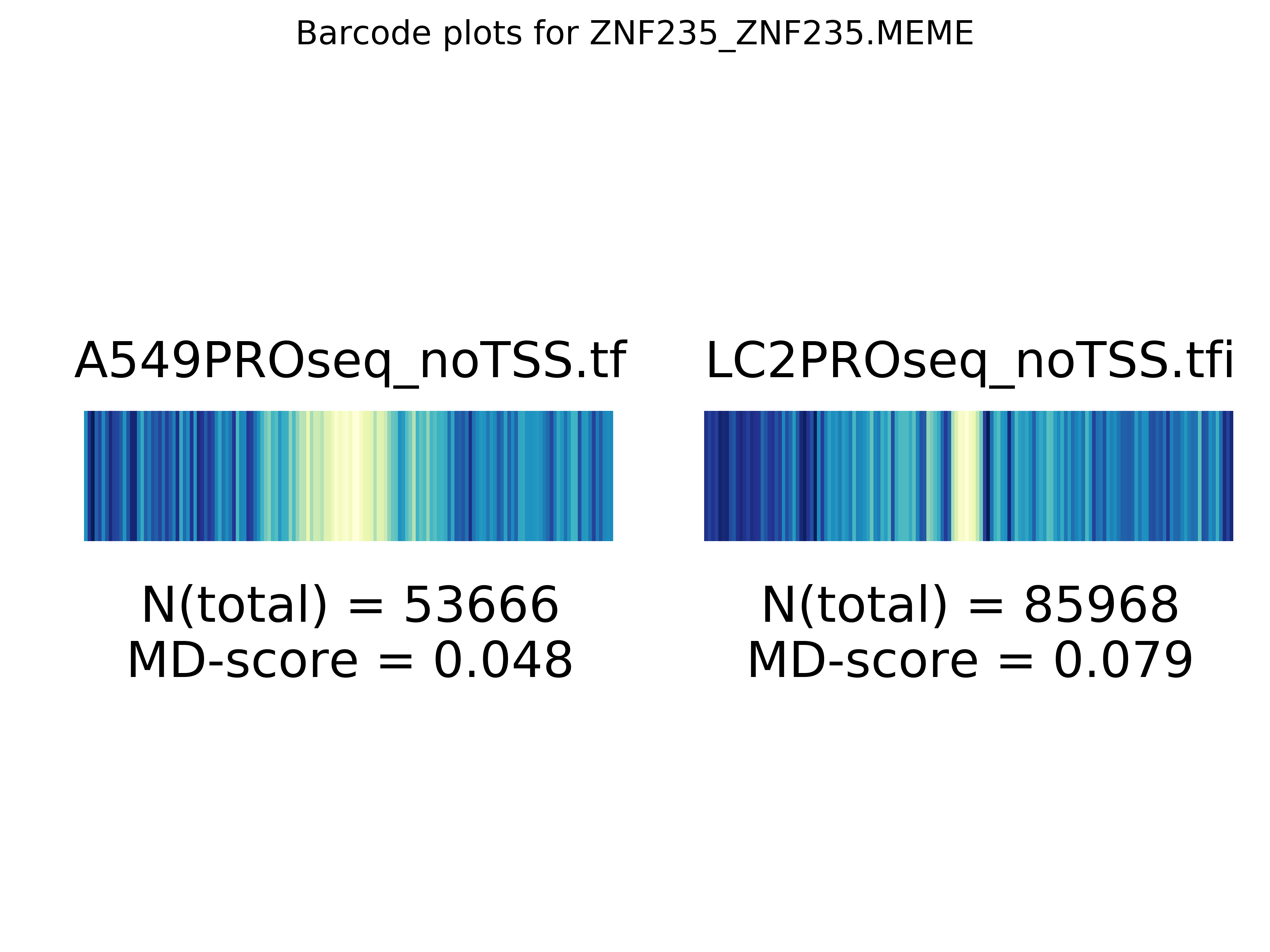

Supplement: Supplemental Data Set 1 [file jciinsight-6-144294-s076.zip › noTSS/best_curated_Human_TFs_p1e-6_grch38/A549_vs_LC2/ZNF235_ZNF235.MEME_barcode_A549PROseq_noTSS.tfit_merged_vs_LC2PROseq_noTSS.tfit_merged.png]

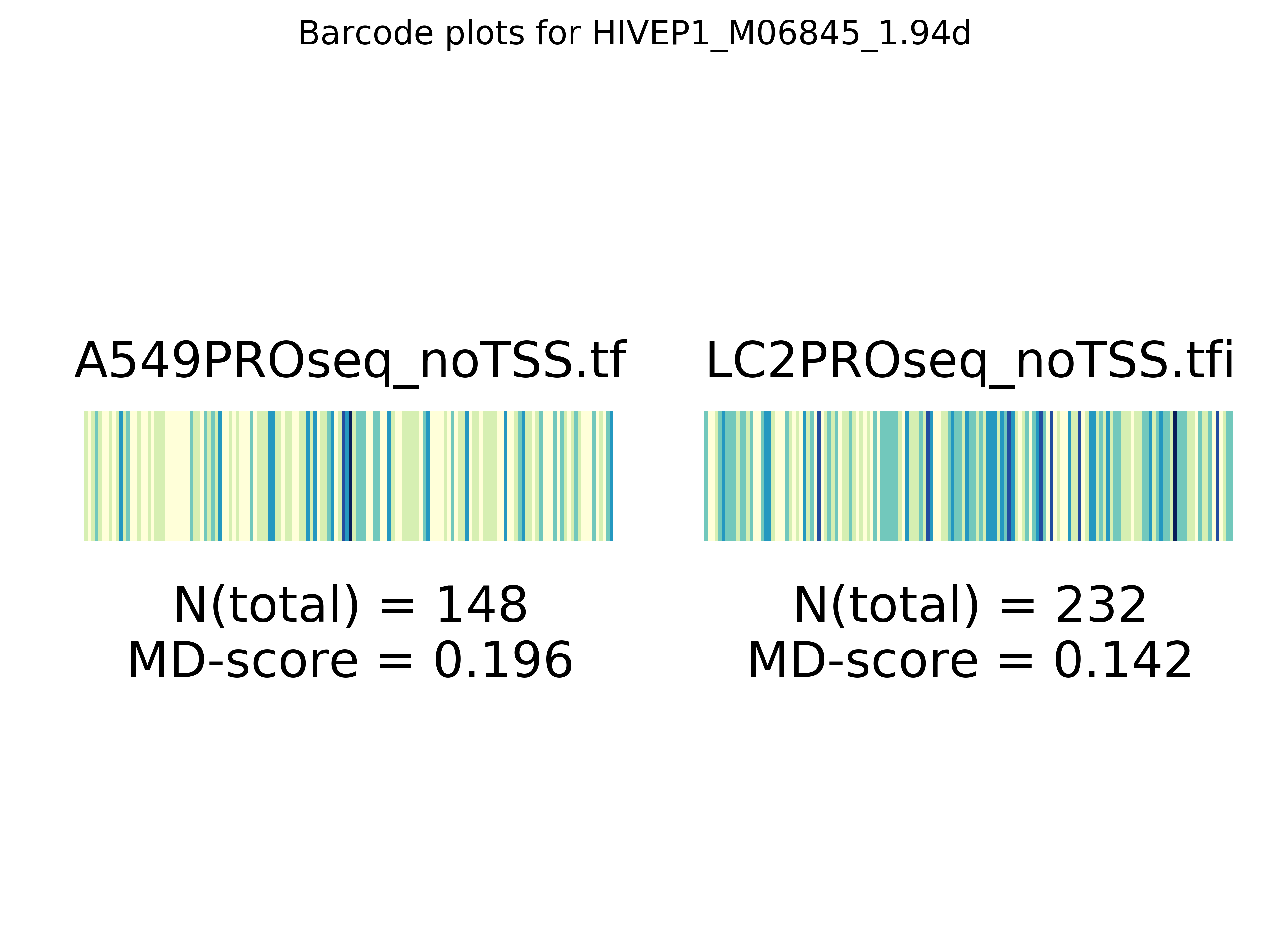

Supplement: Supplemental Data Set 1 [file jciinsight-6-144294-s076.zip › noTSS/best_curated_Human_TFs_p1e-6_grch38/A549_vs_LC2/HIVEP1_M06845_1.94d_barcode_A549PROseq_noTSS.tfit_merged_vs_LC2PROseq_noTSS.tfit_merged.png]

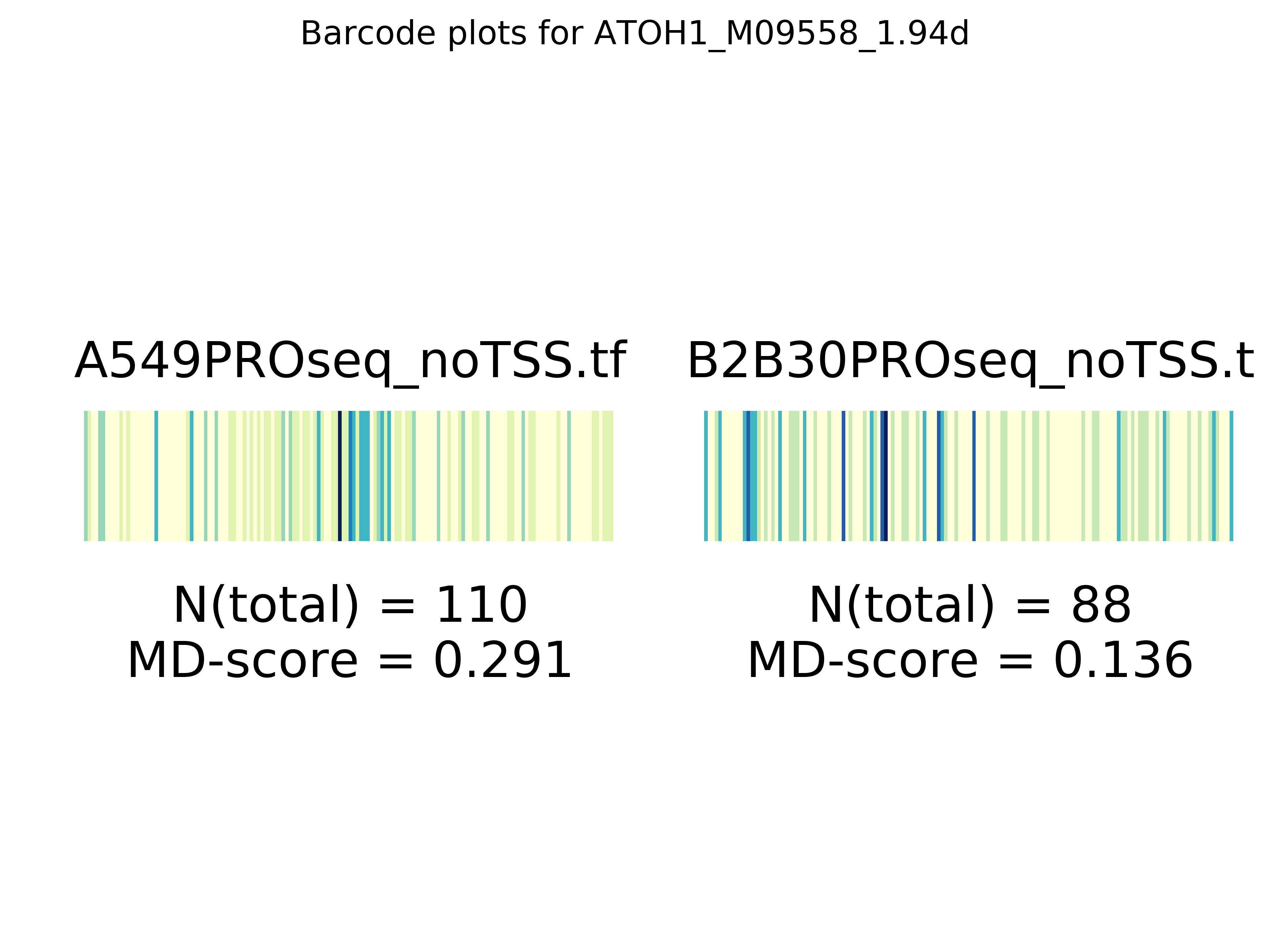

Supplement: Supplemental Data Set 1 [file jciinsight-6-144294-s076.zip › noTSS/best_curated_Human_TFs_p1e-6_grch38/A549_vs_B2B/ATOH1_M09558_1.94d_barcode_A549PROseq_noTSS.tfit_merged_vs_B2B30PROseq_noTSS.tfit_merged.png]

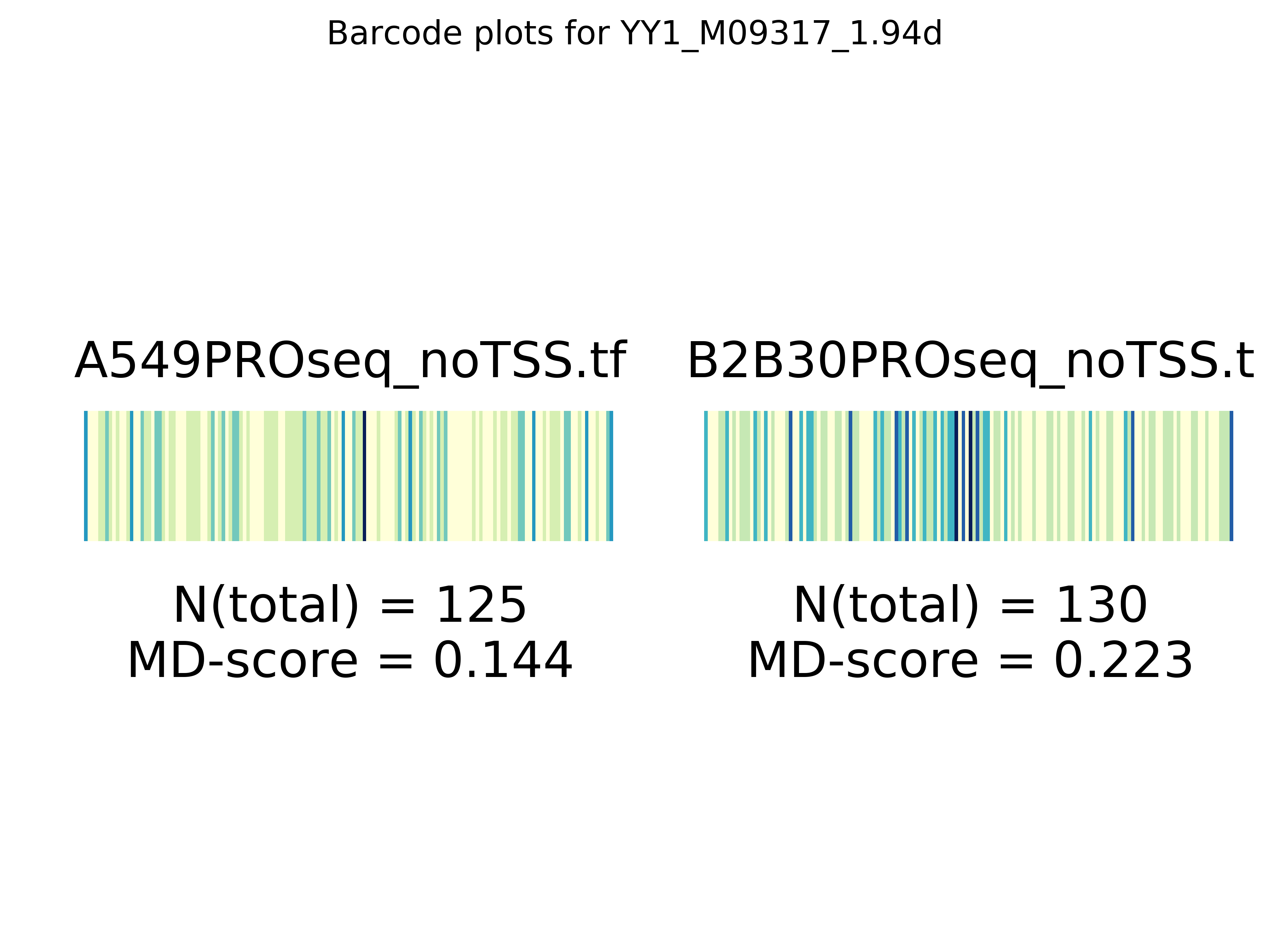

Supplement: Supplemental Data Set 1 [file jciinsight-6-144294-s076.zip › noTSS/best_curated_Human_TFs_p1e-6_grch38/A549_vs_B2B/YY1_M09317_1.94d_barcode_A549PROseq_noTSS.tfit_merged_vs_B2B30PROseq_noTSS.tfit_merged.png]

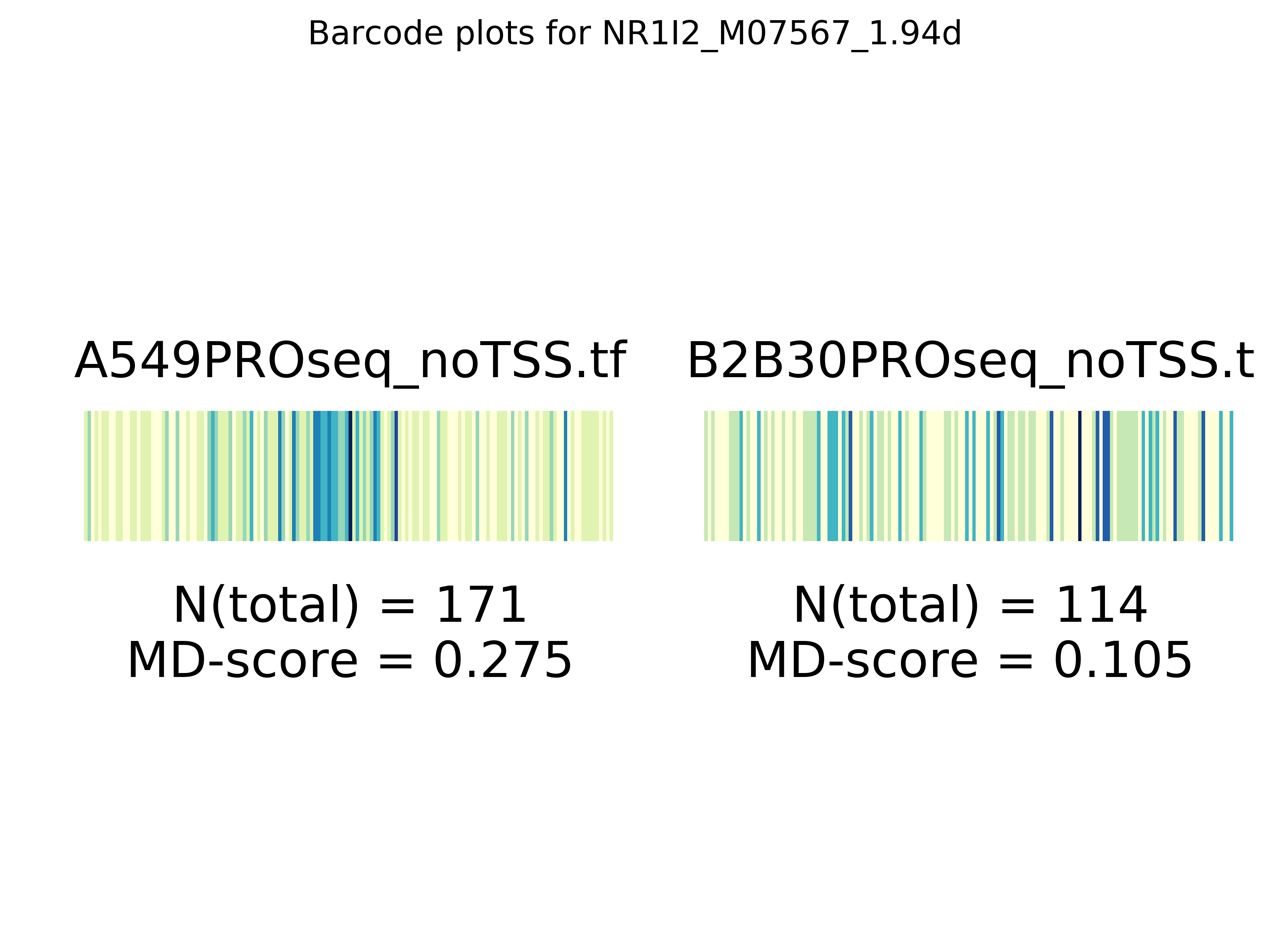

Supplement: Supplemental Data Set 1 [file jciinsight-6-144294-s076.zip › noTSS/best_curated_Human_TFs_p1e-6_grch38/A549_vs_B2B/NR1I2_M07567_1.94d_barcode_A549PROseq_noTSS.tfit_merged_vs_B2B30PROseq_noTSS.tfit_merged.png]

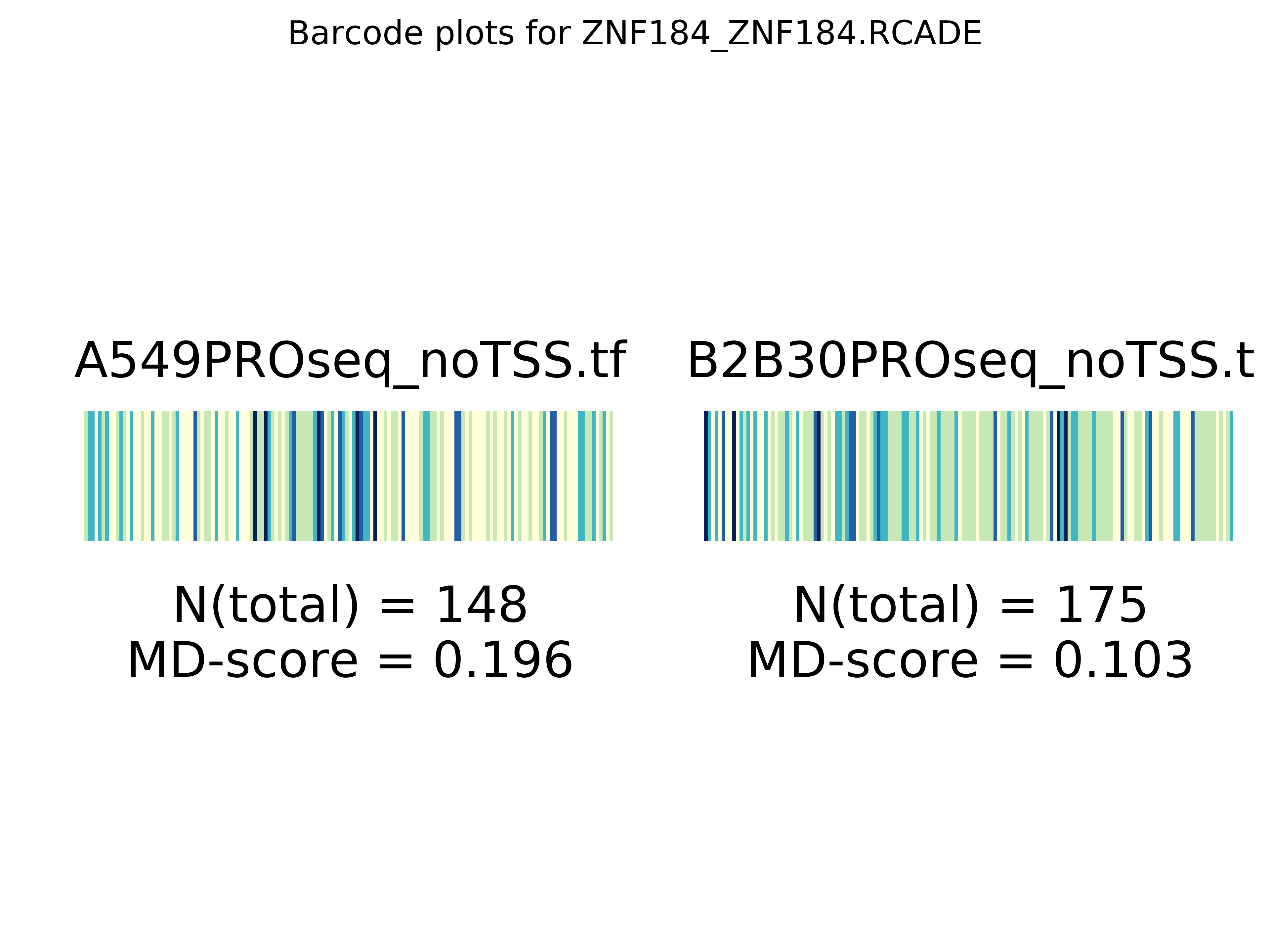

Supplement: Supplemental Data Set 1 [file jciinsight-6-144294-s076.zip › noTSS/best_curated_Human_TFs_p1e-6_grch38/A549_vs_B2B/ZNF184_ZNF184.RCADE_barcode_A549PROseq_noTSS.tfit_merged_vs_B2B30PROseq_noTSS.tfit_merged.png]

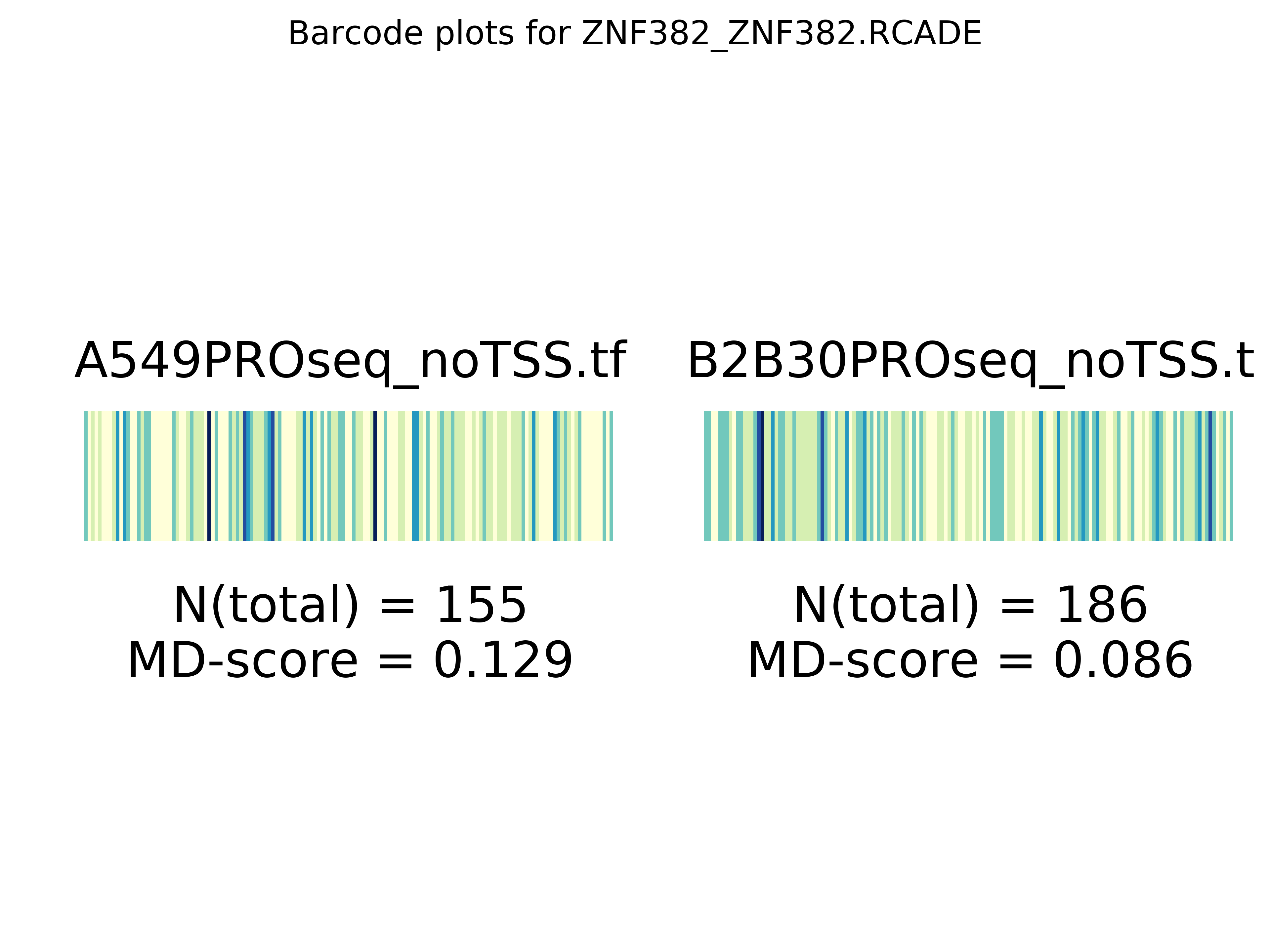

Supplement: Supplemental Data Set 1 [file jciinsight-6-144294-s076.zip › noTSS/best_curated_Human_TFs_p1e-6_grch38/A549_vs_B2B/ZNF382_ZNF382.RCADE_barcode_A549PROseq_noTSS.tfit_merged_vs_B2B30PROseq_noTSS.tfit_merged.png]

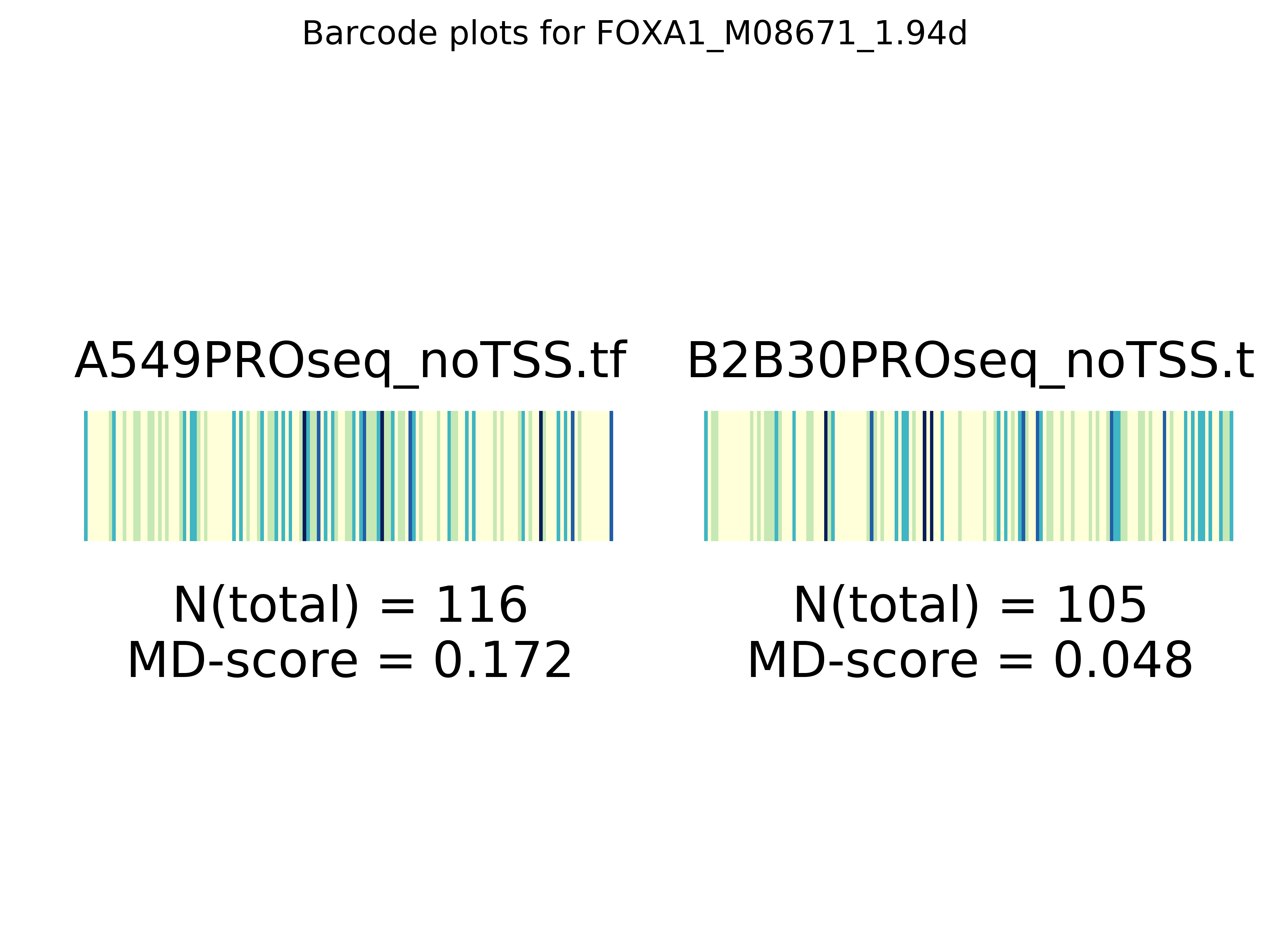

Supplement: Supplemental Data Set 1 [file jciinsight-6-144294-s076.zip › noTSS/best_curated_Human_TFs_p1e-6_grch38/A549_vs_B2B/FOXA1_M08671_1.94d_barcode_A549PROseq_noTSS.tfit_merged_vs_B2B30PROseq_noTSS.tfit_merged.png]

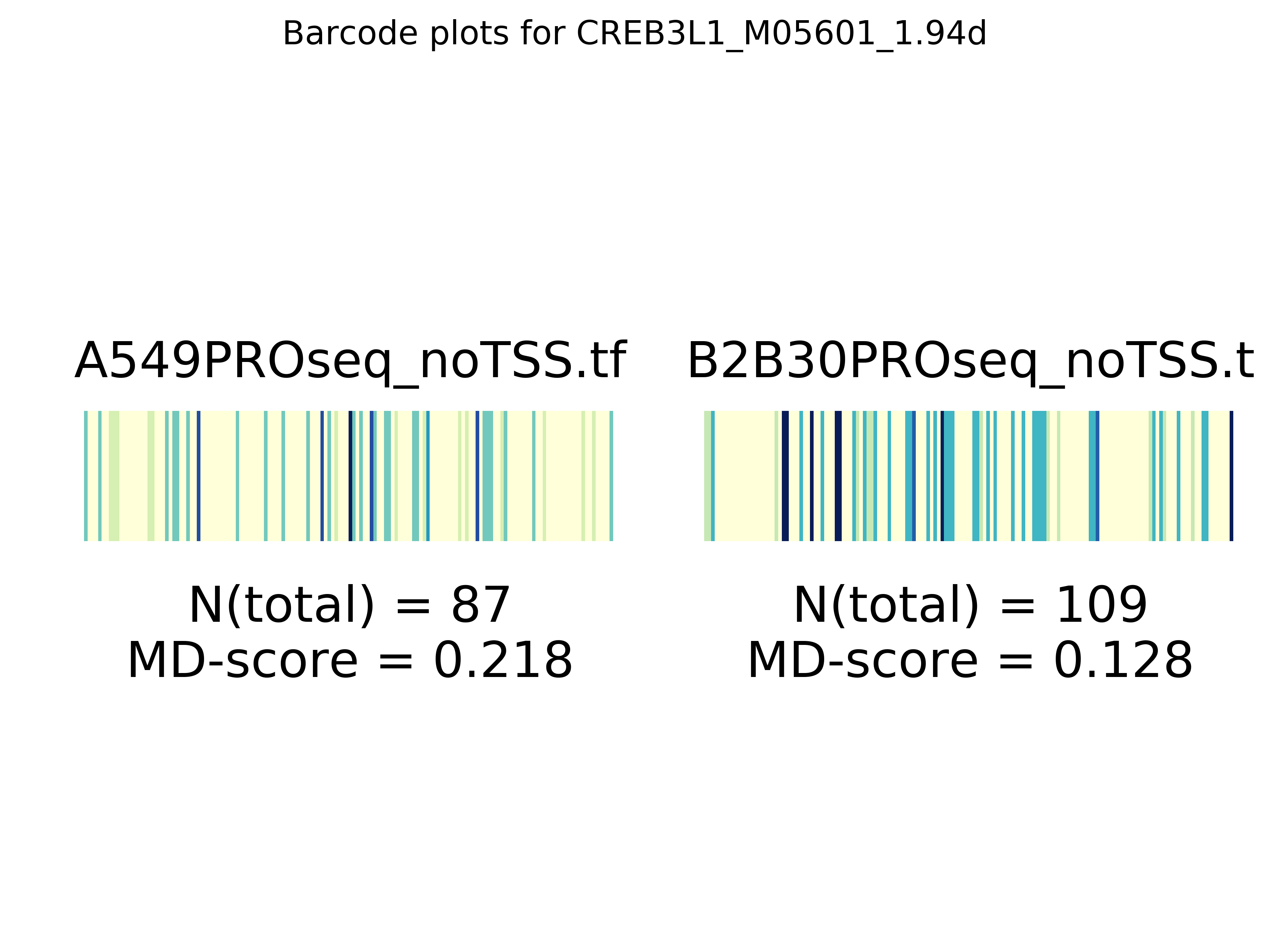

Supplement: Supplemental Data Set 1 [file jciinsight-6-144294-s076.zip › noTSS/best_curated_Human_TFs_p1e-6_grch38/A549_vs_B2B/CREB3L1_M05601_1.94d_barcode_A549PROseq_noTSS.tfit_merged_vs_B2B30PROseq_noTSS.tfit_merged.png]

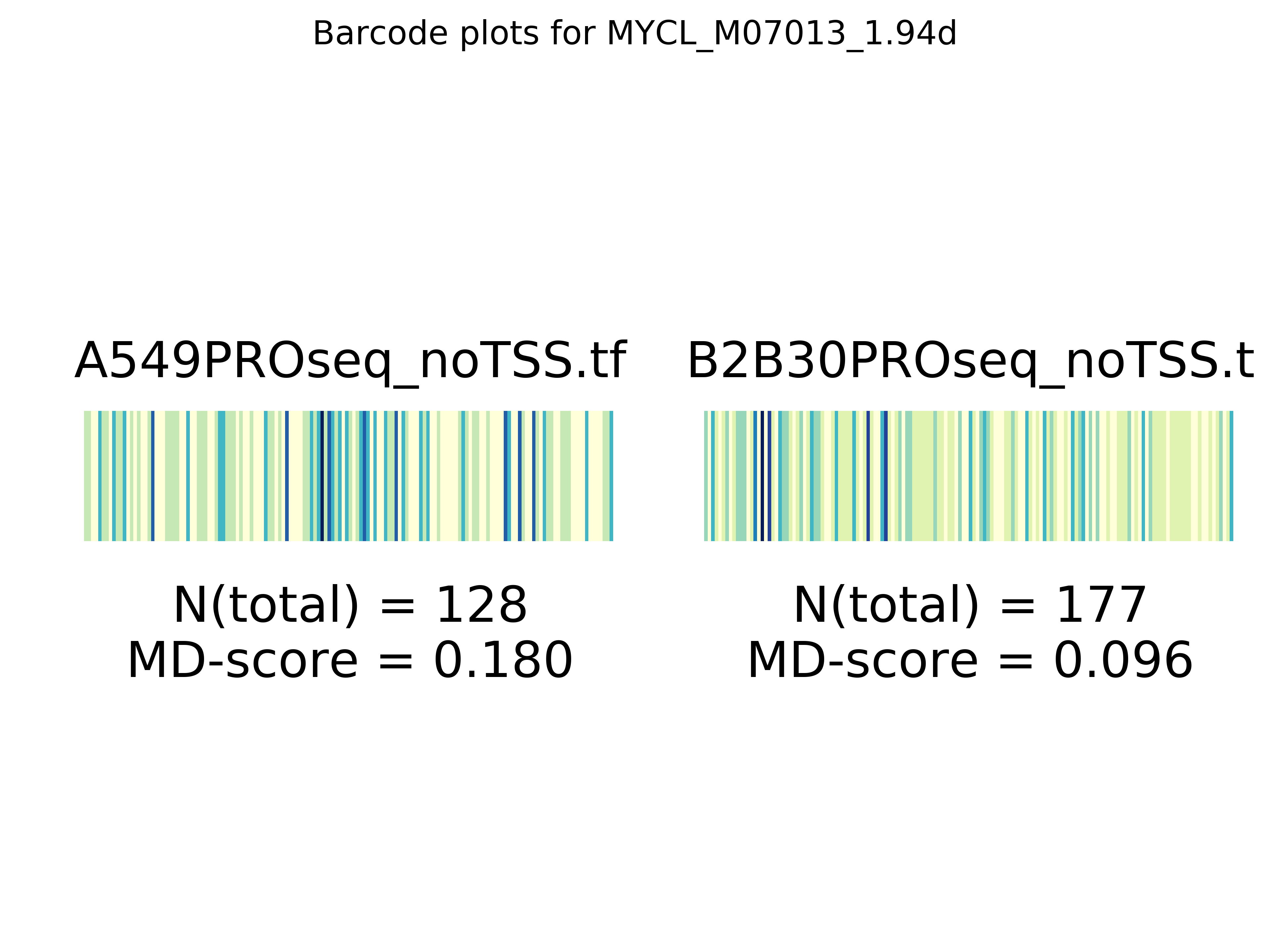

Supplement: Supplemental Data Set 1 [file jciinsight-6-144294-s076.zip › noTSS/best_curated_Human_TFs_p1e-6_grch38/A549_vs_B2B/MYCL_M07013_1.94d_barcode_A549PROseq_noTSS.tfit_merged_vs_B2B30PROseq_noTSS.tfit_merged.png]

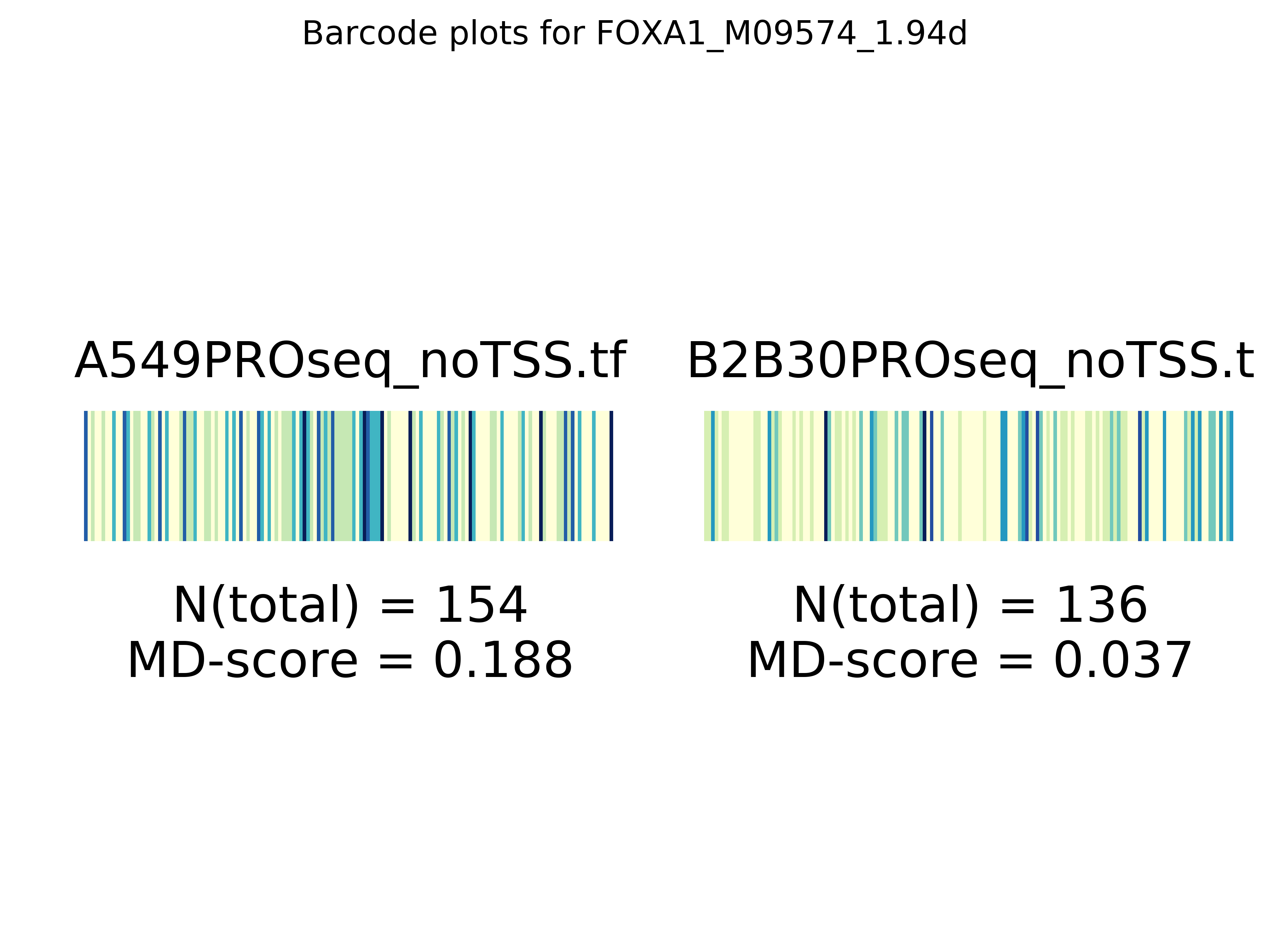

Supplement: Supplemental Data Set 1 [file jciinsight-6-144294-s076.zip › noTSS/best_curated_Human_TFs_p1e-6_grch38/A549_vs_B2B/FOXA1_M09574_1.94d_barcode_A549PROseq_noTSS.tfit_merged_vs_B2B30PROseq_noTSS.tfit_merged.png]

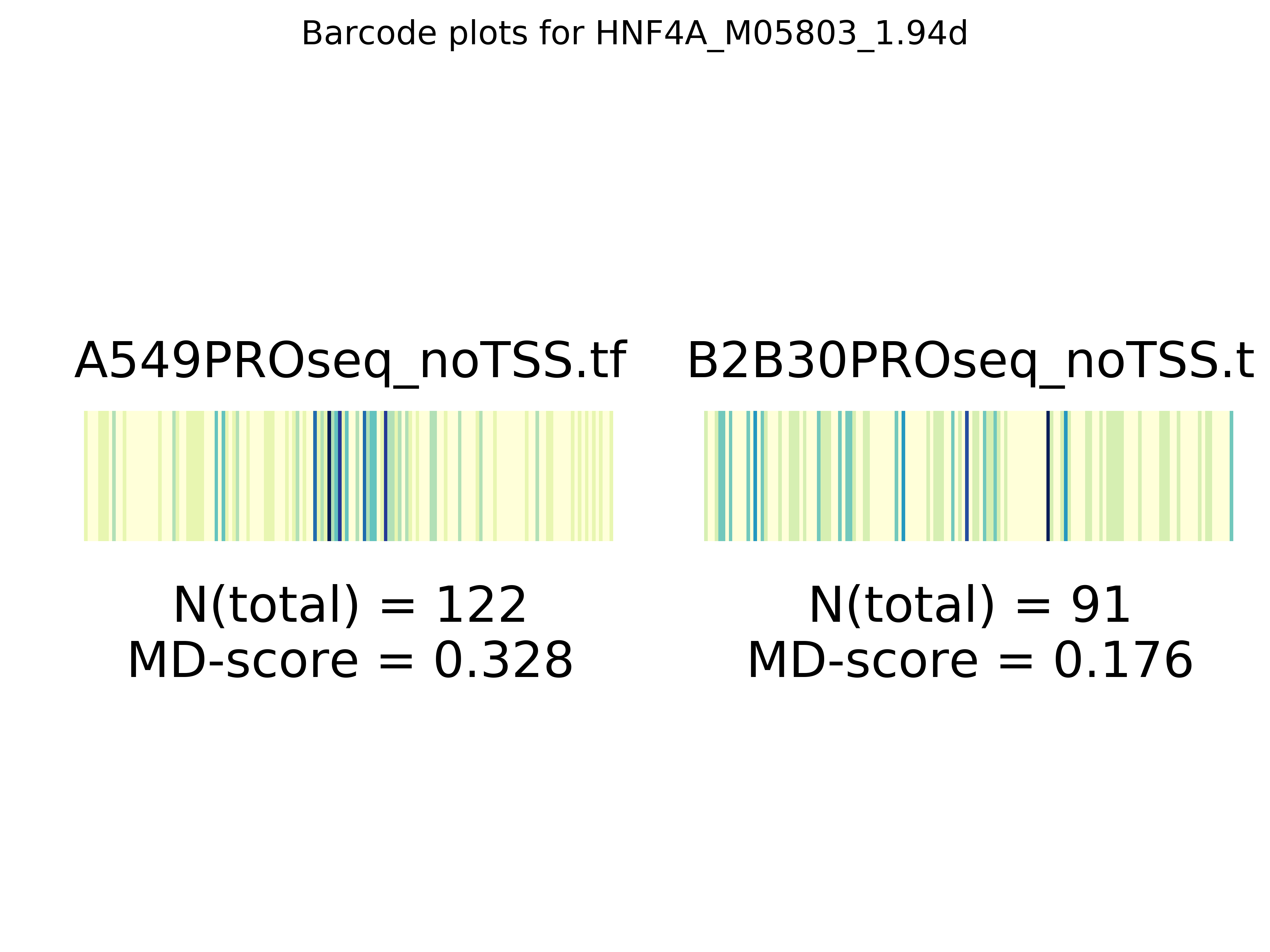

Supplement: Supplemental Data Set 1 [file jciinsight-6-144294-s076.zip › noTSS/best_curated_Human_TFs_p1e-6_grch38/A549_vs_B2B/HNF4A_M05803_1.94d_barcode_A549PROseq_noTSS.tfit_merged_vs_B2B30PROseq_noTSS.tfit_merged.png]

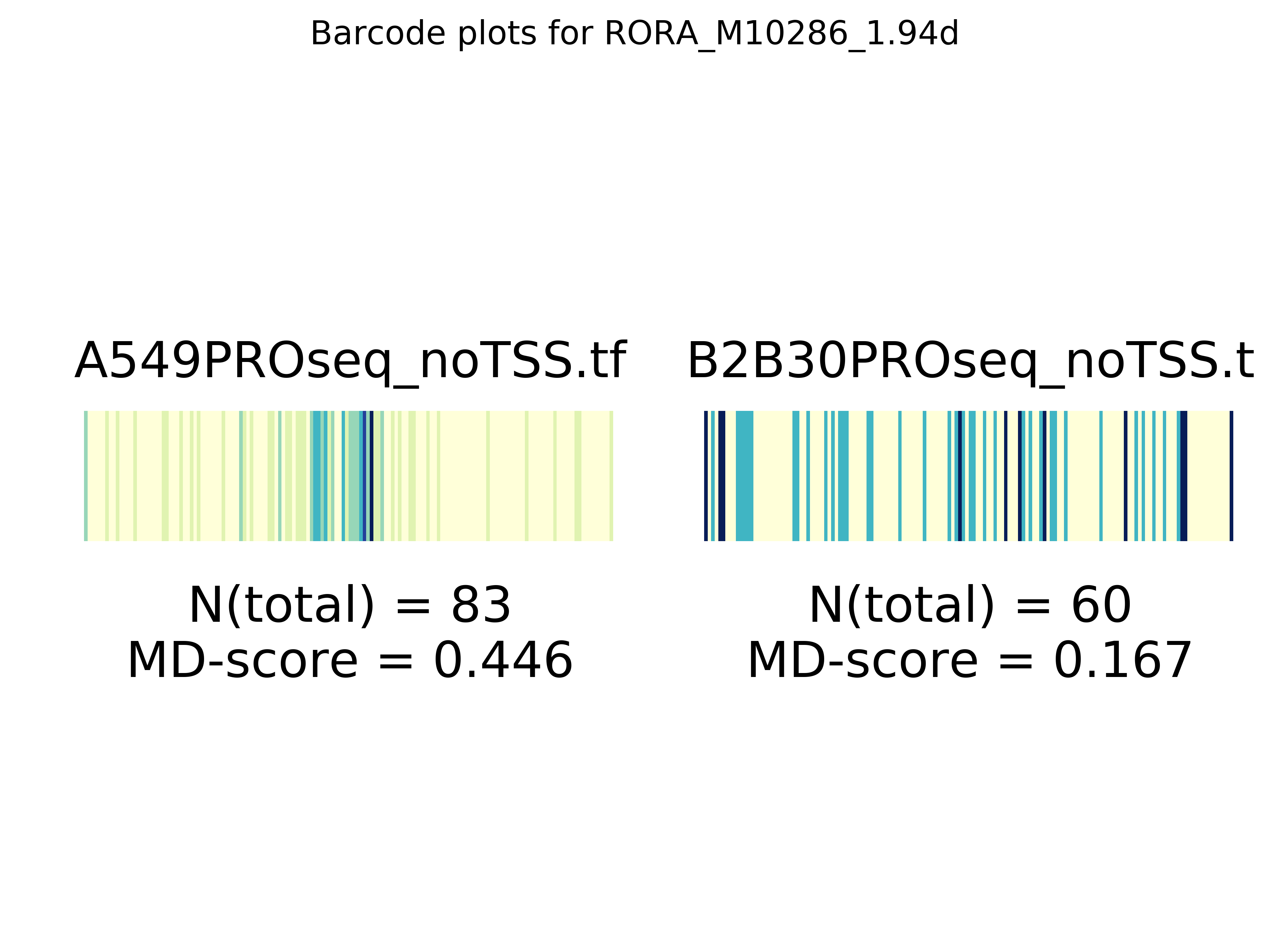

Supplement: Supplemental Data Set 1 [file jciinsight-6-144294-s076.zip › noTSS/best_curated_Human_TFs_p1e-6_grch38/A549_vs_B2B/RORA_M10286_1.94d_barcode_A549PROseq_noTSS.tfit_merged_vs_B2B30PROseq_noTSS.tfit_merged.png]

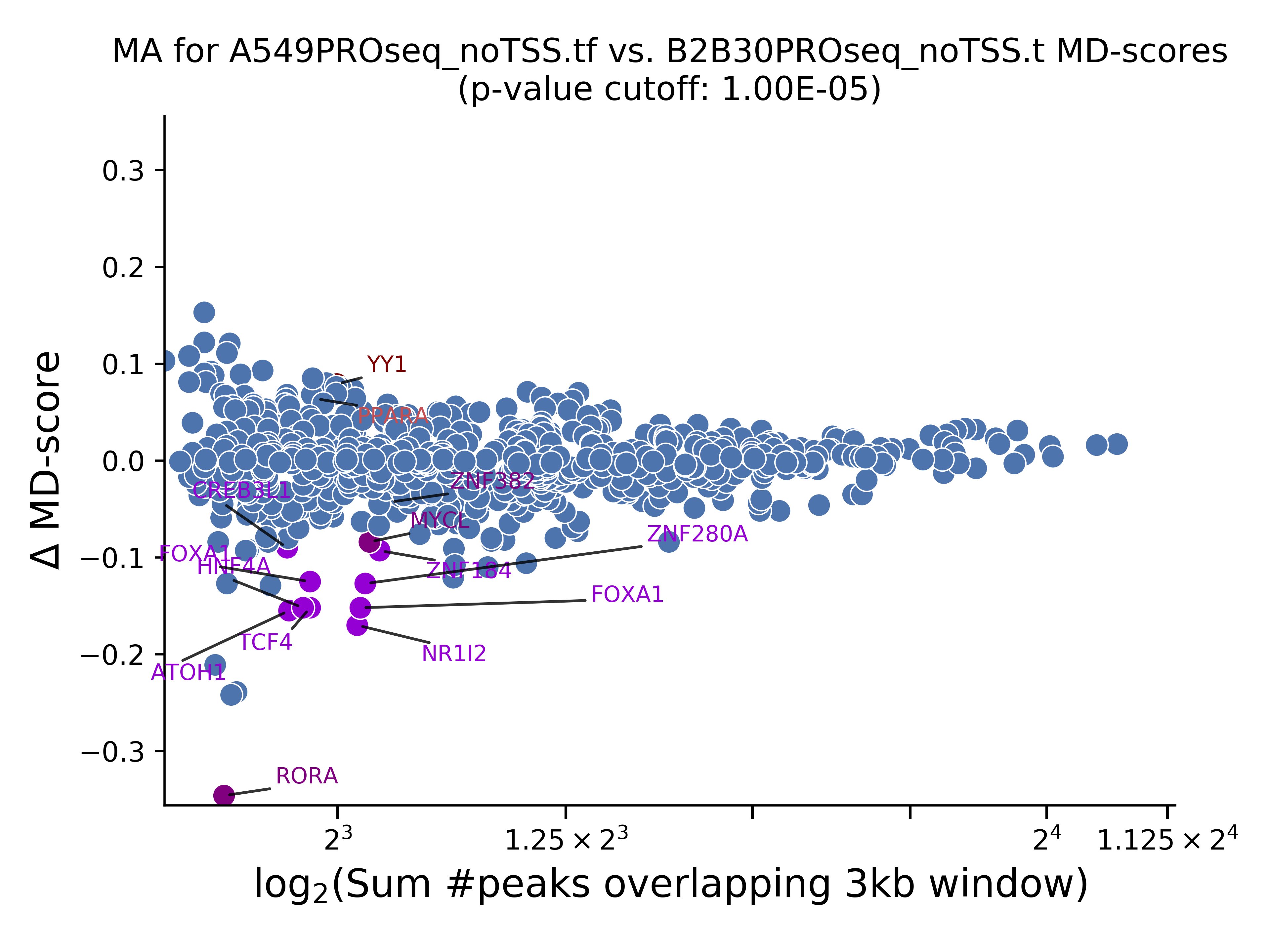

Supplement: Supplemental Data Set 1 [file jciinsight-6-144294-s076.zip › noTSS/best_curated_Human_TFs_p1e-6_grch38/A549_vs_B2B/MA_A549PROseq_noTSS.tfit_merged_to_B2B30PROseq_noTSS.tfit_merged_md_score.png]

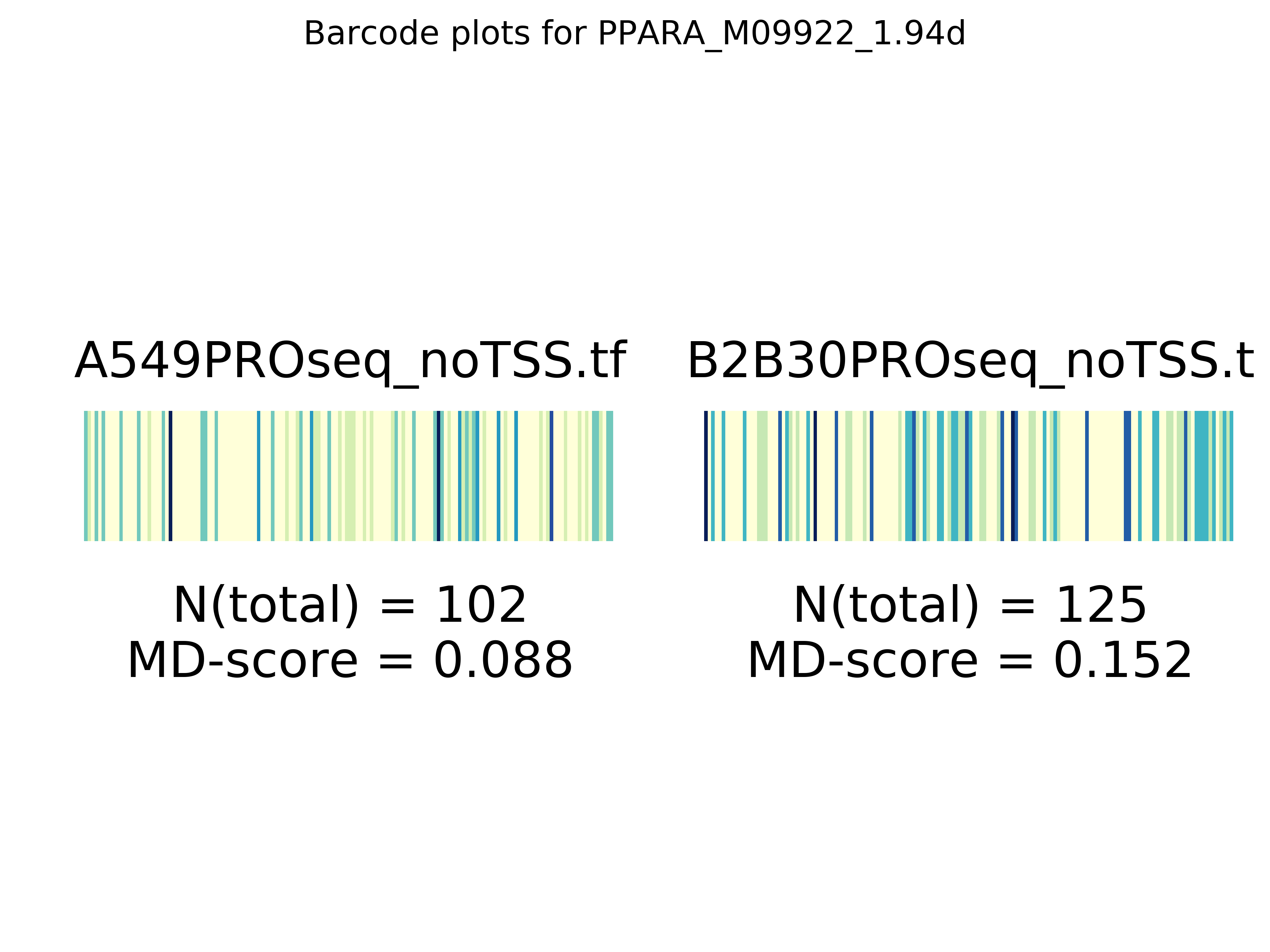

Supplement: Supplemental Data Set 1 [file jciinsight-6-144294-s076.zip › noTSS/best_curated_Human_TFs_p1e-6_grch38/A549_vs_B2B/PPARA_M09922_1.94d_barcode_A549PROseq_noTSS.tfit_merged_vs_B2B30PROseq_noTSS.tfit_merged.png]

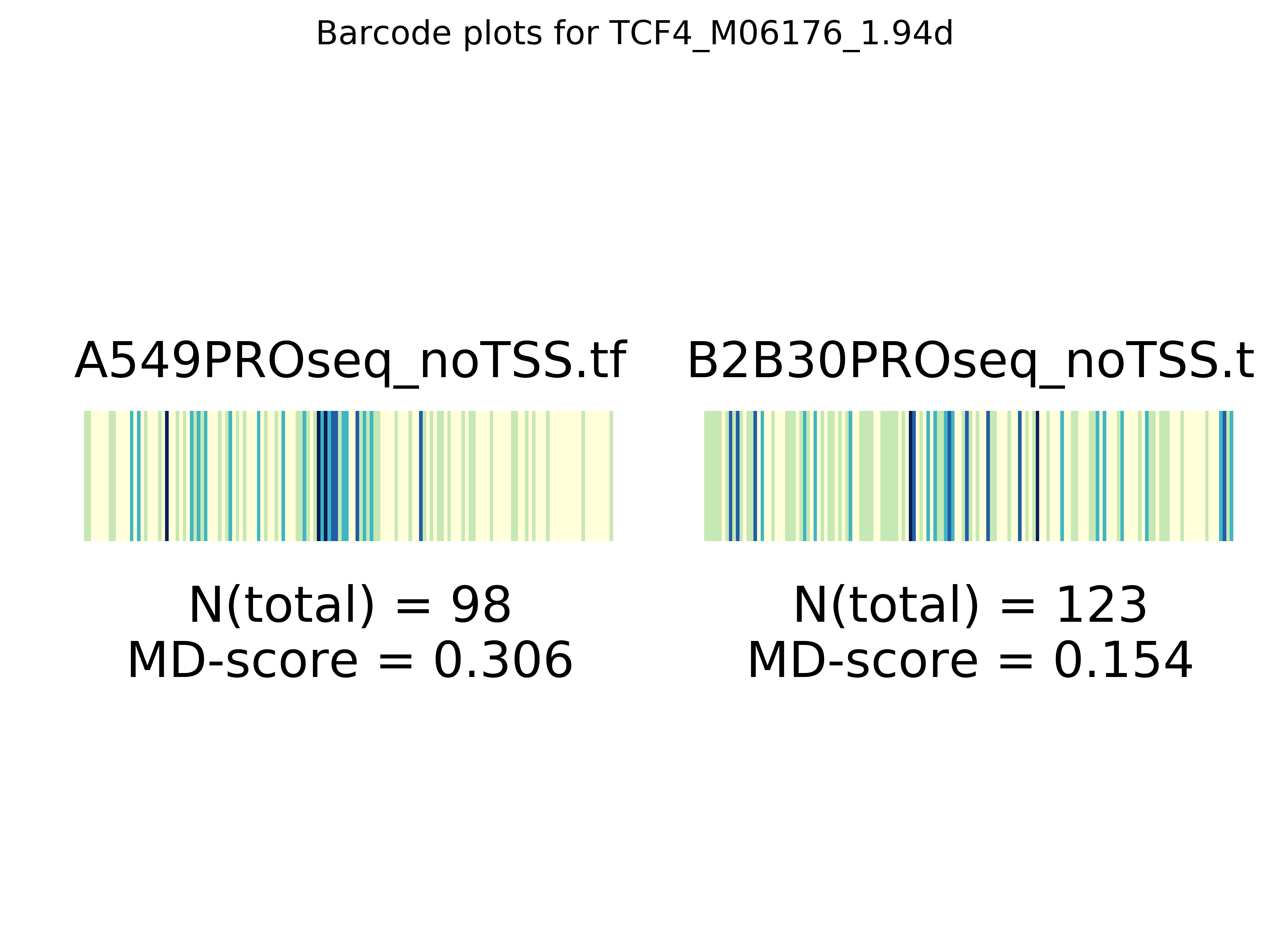

Supplement: Supplemental Data Set 1 [file jciinsight-6-144294-s076.zip › noTSS/best_curated_Human_TFs_p1e-6_grch38/A549_vs_B2B/TCF4_M06176_1.94d_barcode_A549PROseq_noTSS.tfit_merged_vs_B2B30PROseq_noTSS.tfit_merged.png]

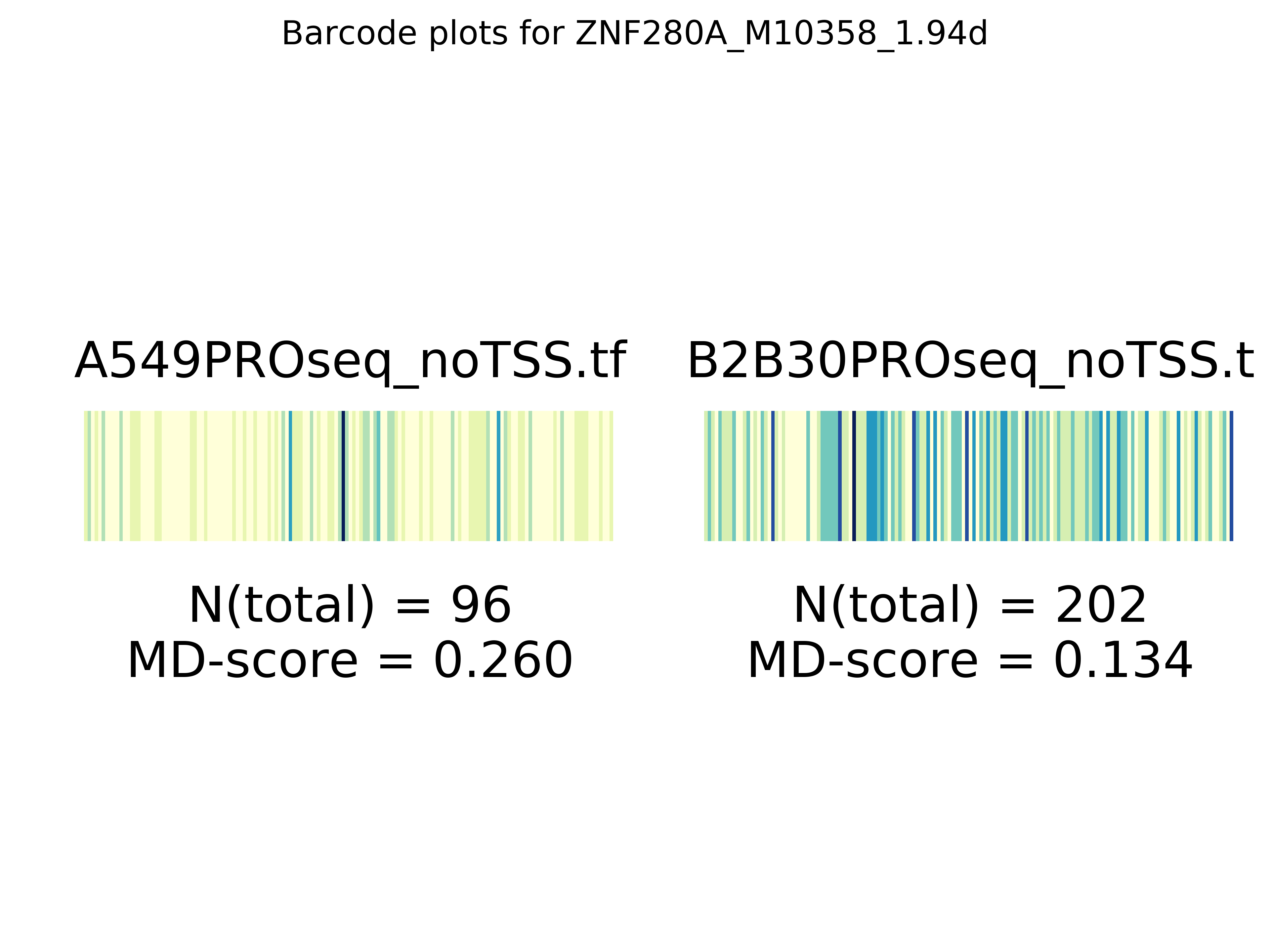

Supplement: Supplemental Data Set 1 [file jciinsight-6-144294-s076.zip › noTSS/best_curated_Human_TFs_p1e-6_grch38/A549_vs_B2B/ZNF280A_M10358_1.94d_barcode_A549PROseq_noTSS.tfit_merged_vs_B2B30PROseq_noTSS.tfit_merged.png]

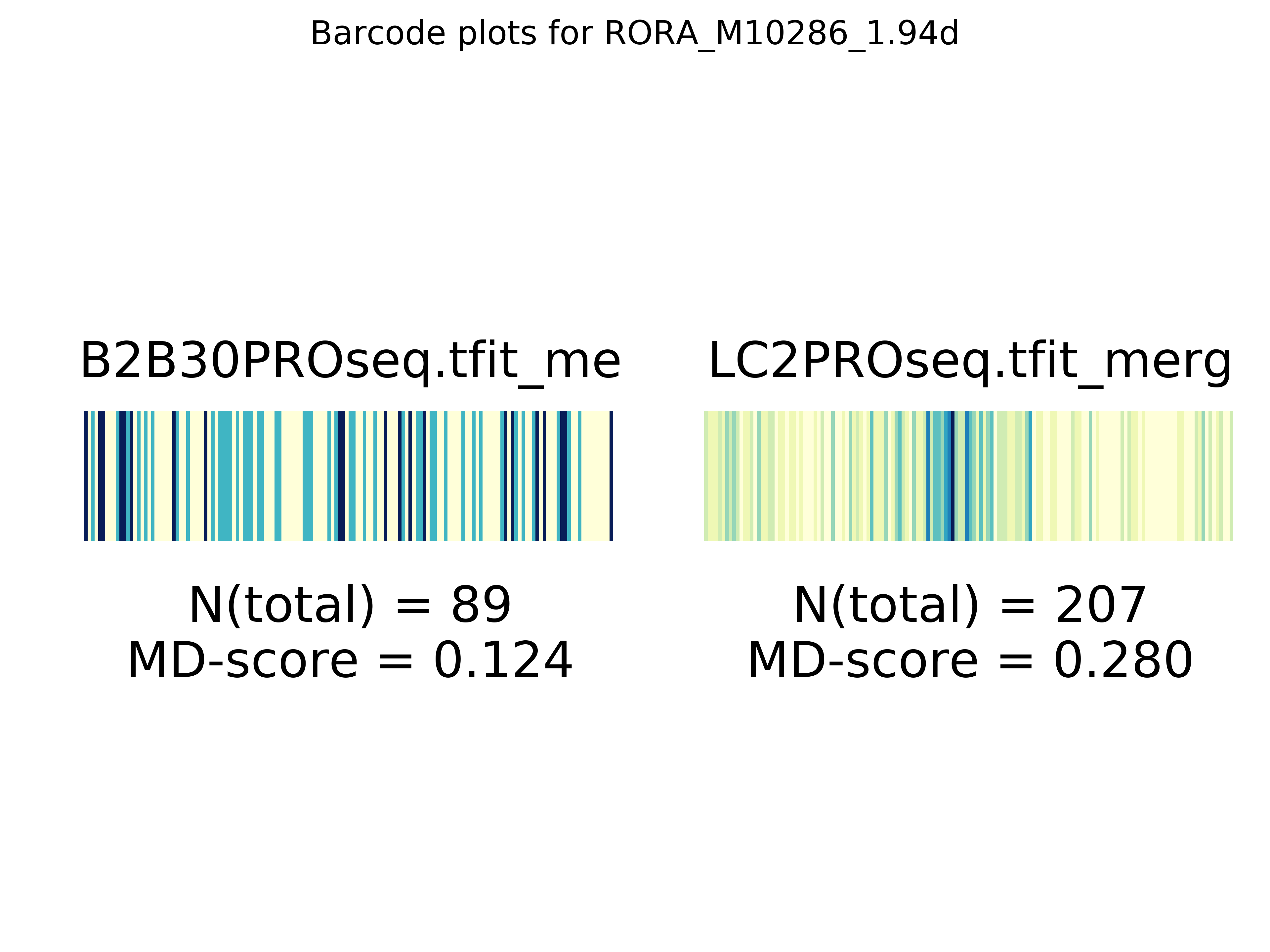

Supplement: Supplemental Data Set 2 [file jciinsight-6-144294-s077.zip › best_curated_Human_TFs_p1e-6_grch38/B2B_vs_LC2/RORA_M10286_1.94d_barcode_B2B30PROseq.tfit_merged_vs_LC2PROseq.tfit_merged.png]

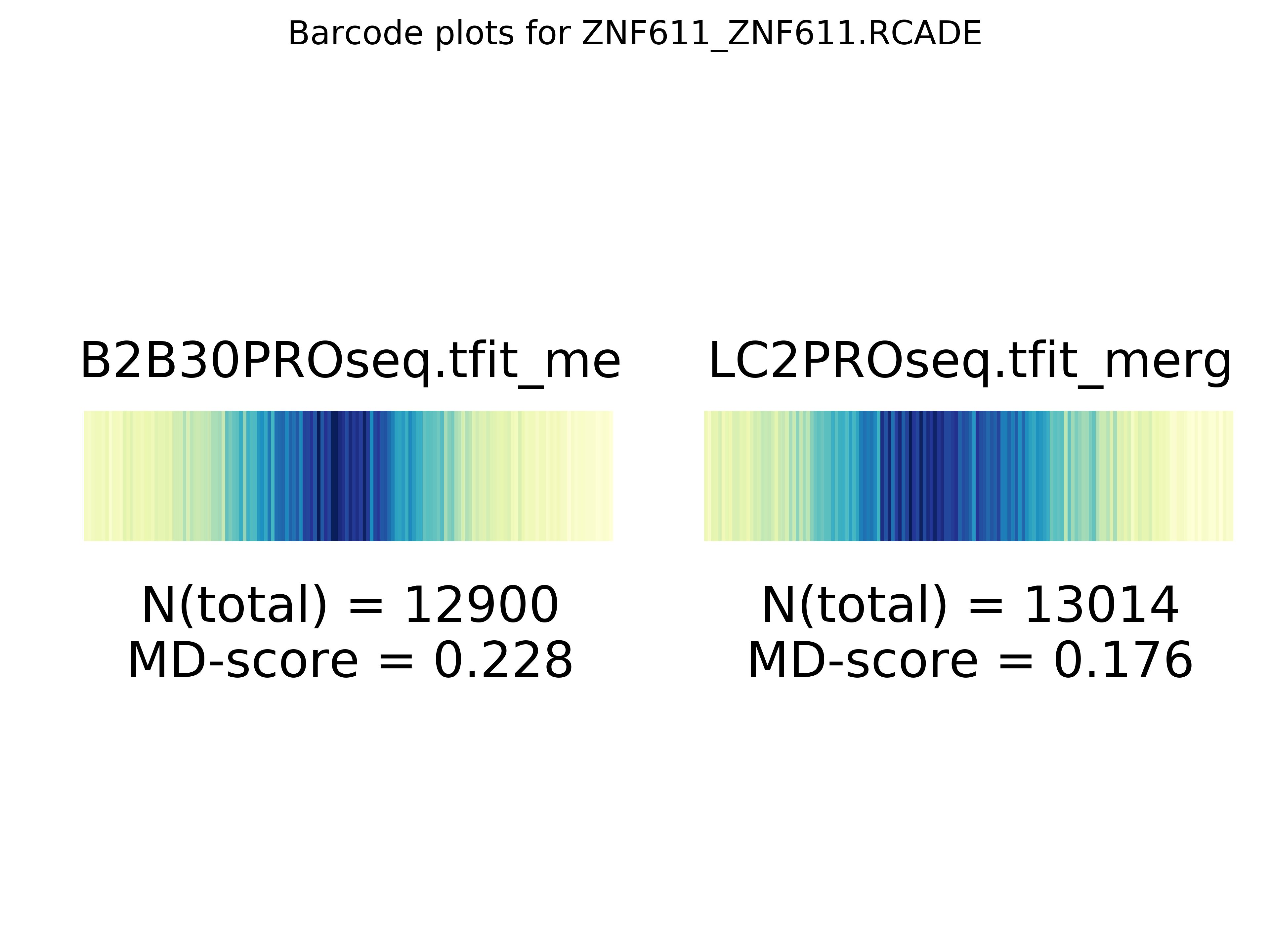

Supplement: Supplemental Data Set 2 [file jciinsight-6-144294-s077.zip › best_curated_Human_TFs_p1e-6_grch38/B2B_vs_LC2/ZNF611_ZNF611.RCADE_barcode_B2B30PROseq.tfit_merged_vs_LC2PROseq.tfit_merged.png]

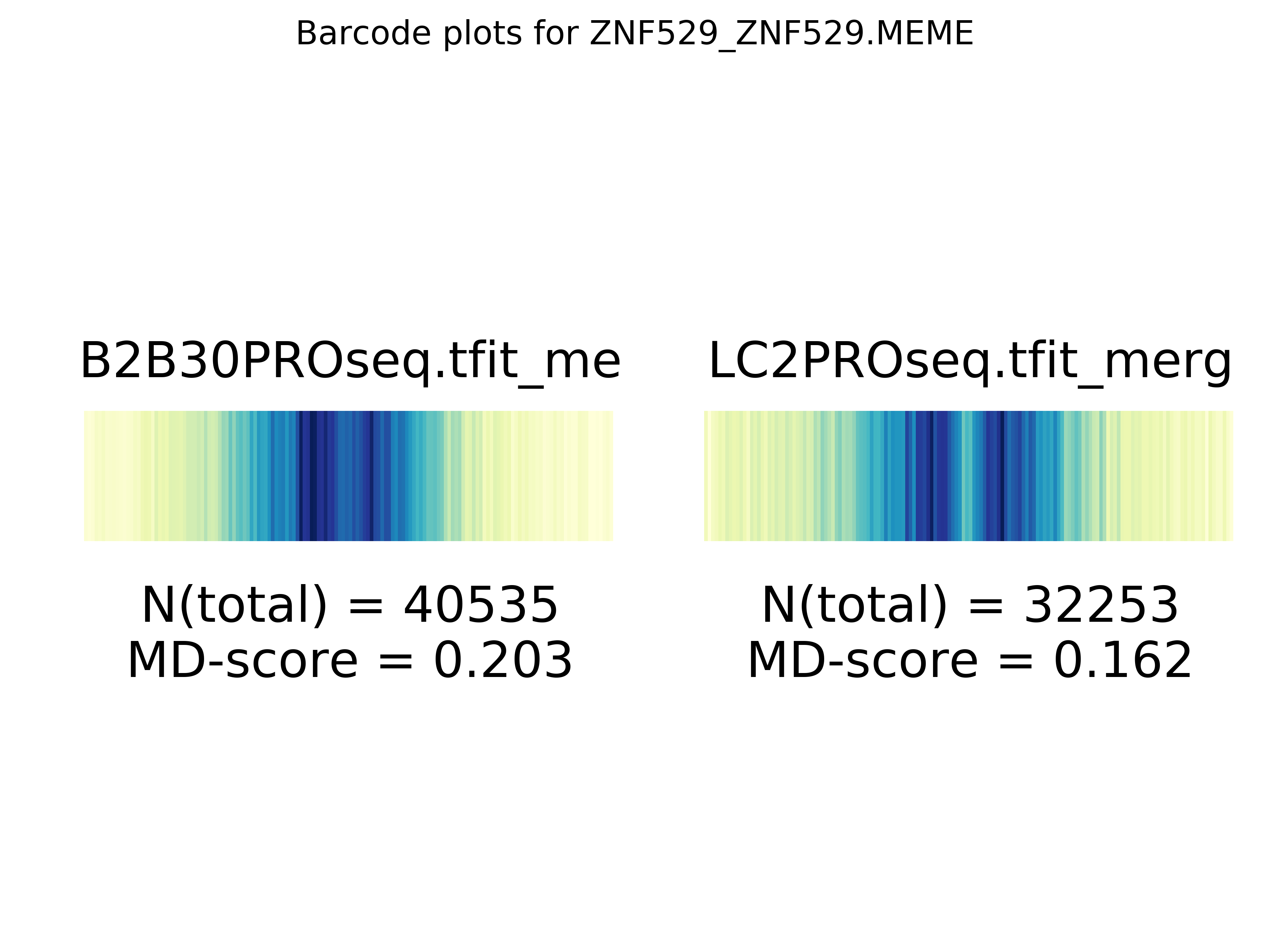

Supplement: Supplemental Data Set 2 [file jciinsight-6-144294-s077.zip › best_curated_Human_TFs_p1e-6_grch38/B2B_vs_LC2/ZNF529_ZNF529.MEME_barcode_B2B30PROseq.tfit_merged_vs_LC2PROseq.tfit_merged.png]

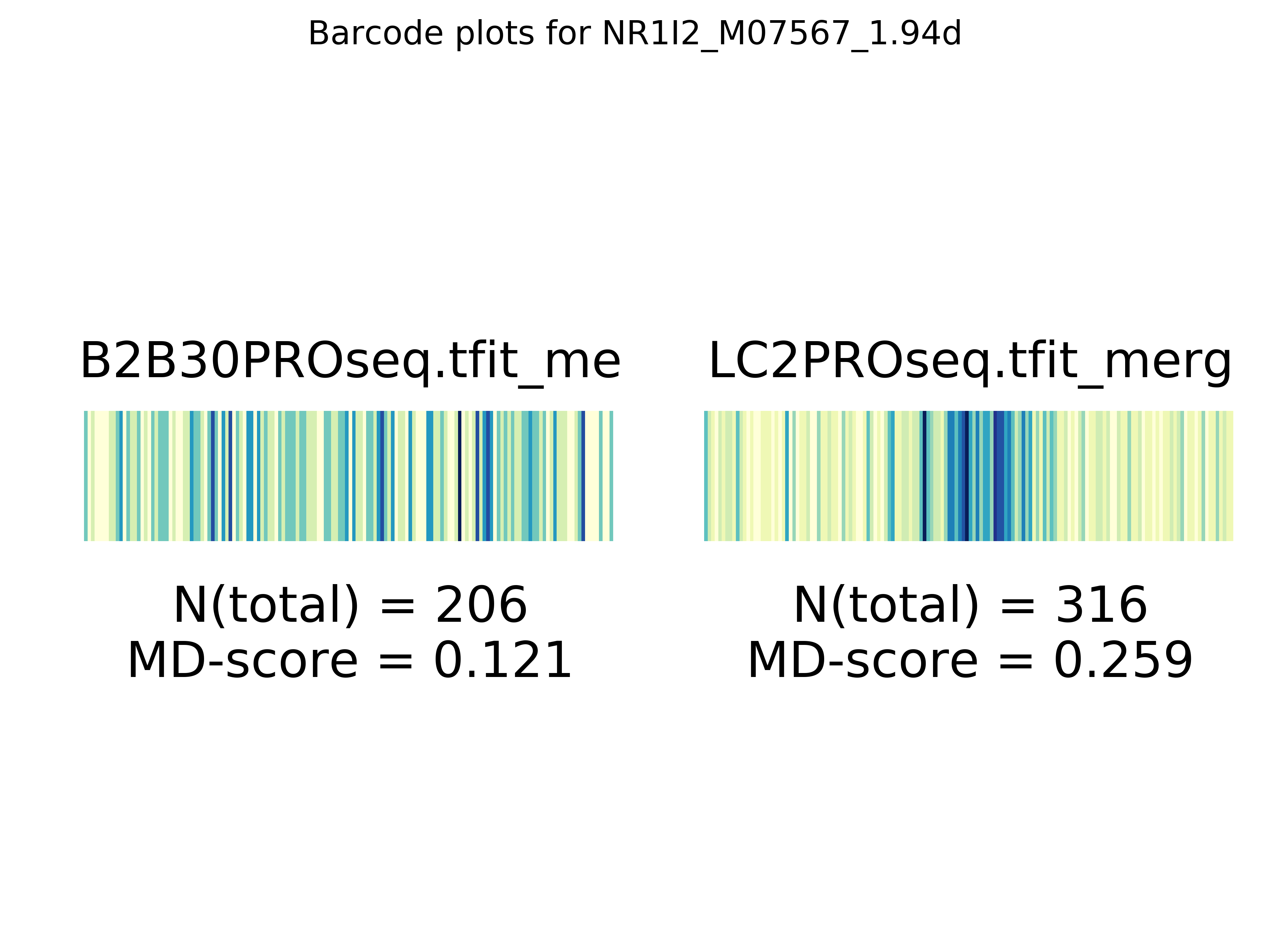

Supplement: Supplemental Data Set 2 [file jciinsight-6-144294-s077.zip › best_curated_Human_TFs_p1e-6_grch38/B2B_vs_LC2/NR1I2_M07567_1.94d_barcode_B2B30PROseq.tfit_merged_vs_LC2PROseq.tfit_merged.png]

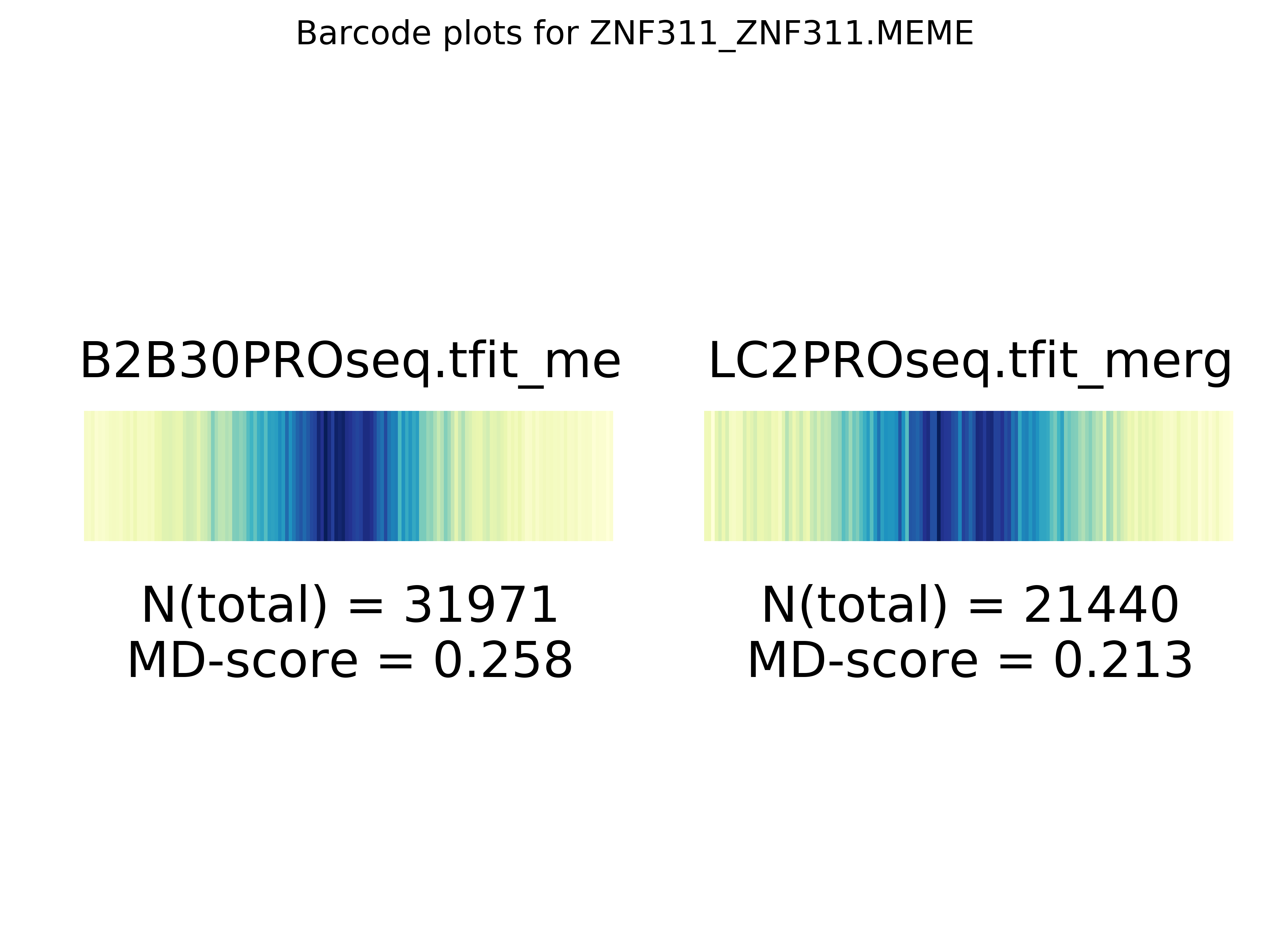

Supplement: Supplemental Data Set 2 [file jciinsight-6-144294-s077.zip › best_curated_Human_TFs_p1e-6_grch38/B2B_vs_LC2/ZNF311_ZNF311.MEME_barcode_B2B30PROseq.tfit_merged_vs_LC2PROseq.tfit_merged.png]

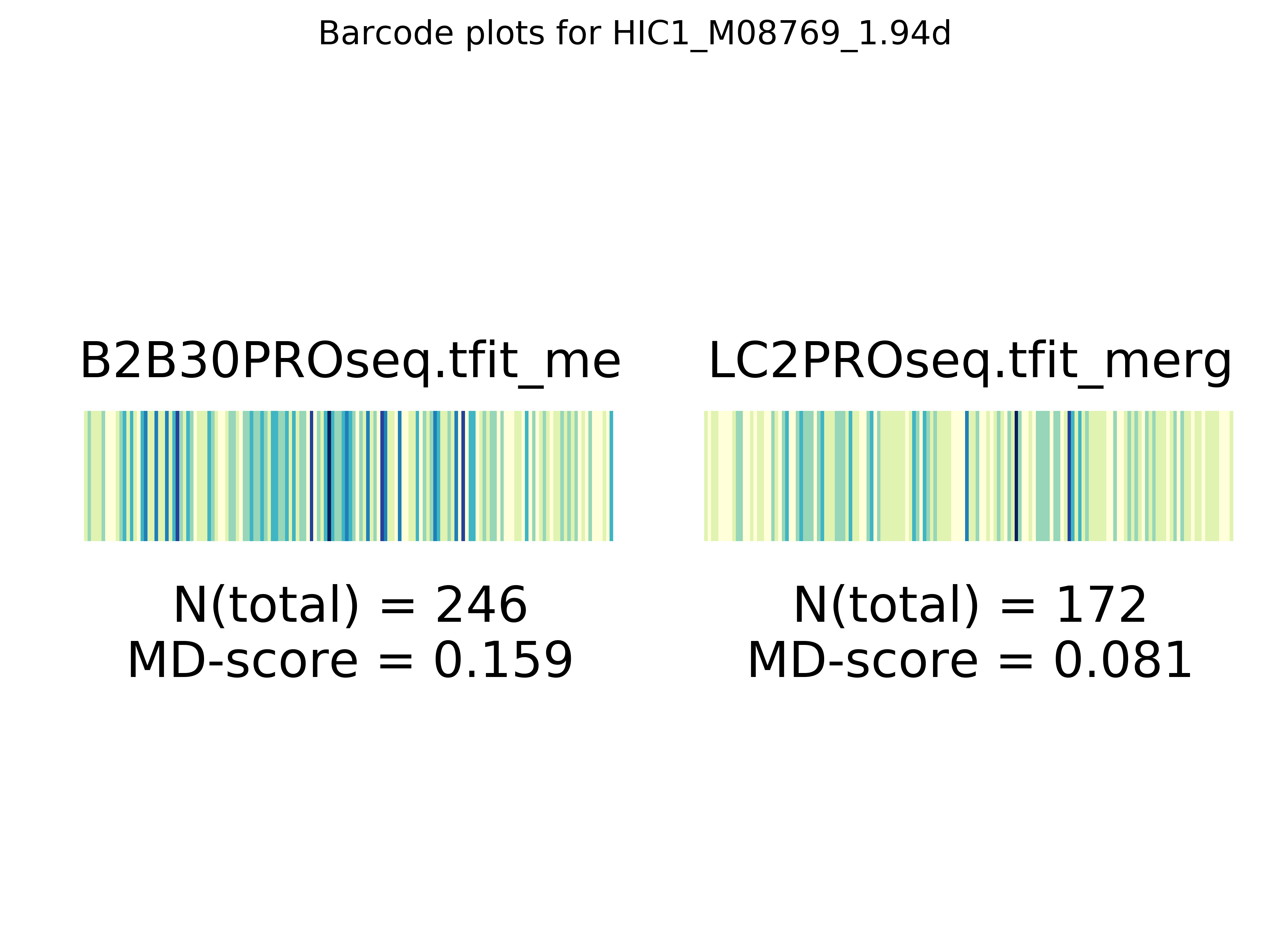

Supplement: Supplemental Data Set 2 [file jciinsight-6-144294-s077.zip › best_curated_Human_TFs_p1e-6_grch38/B2B_vs_LC2/HIC1_M08769_1.94d_barcode_B2B30PROseq.tfit_merged_vs_LC2PROseq.tfit_merged.png]

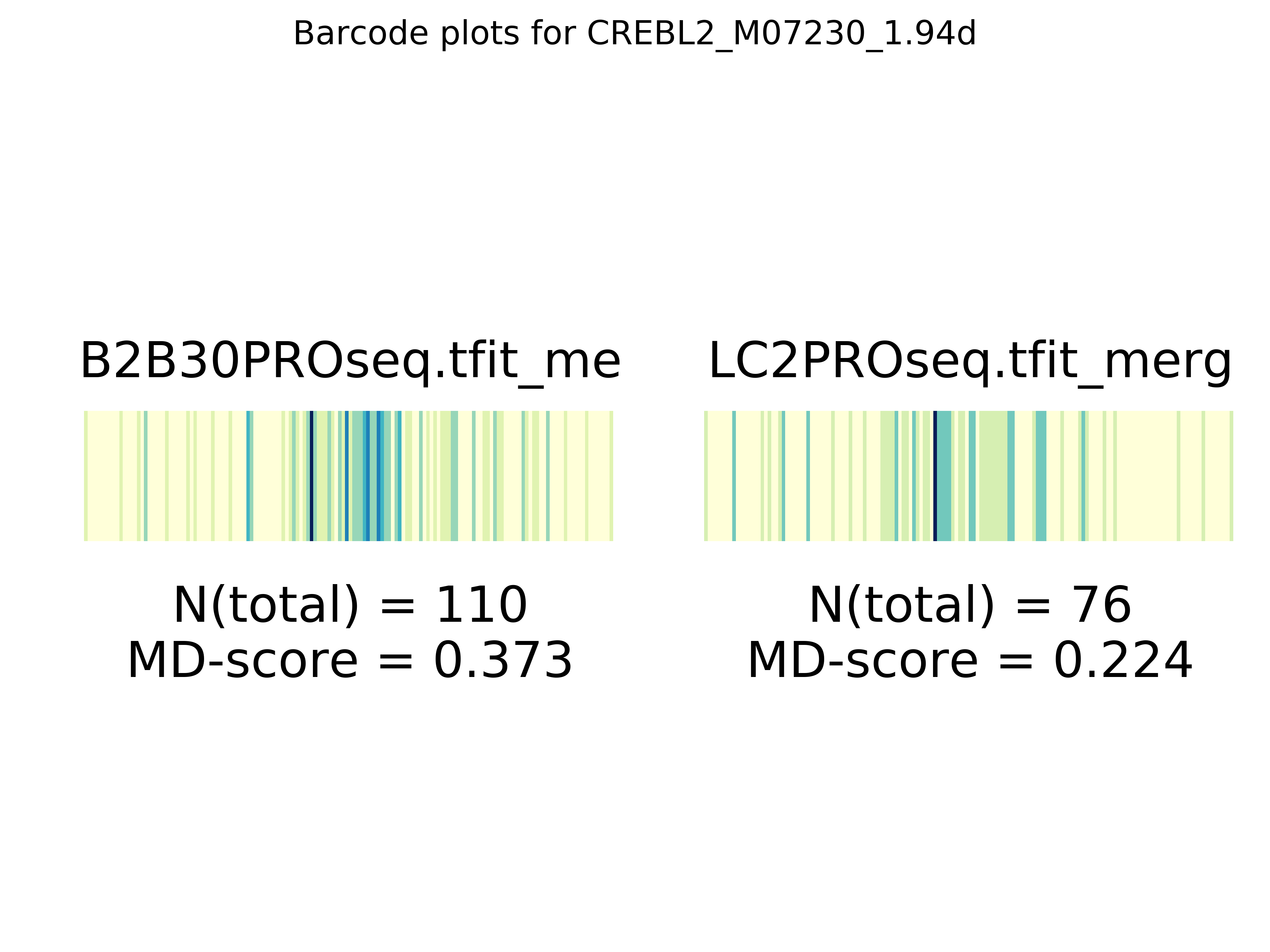

Supplement: Supplemental Data Set 2 [file jciinsight-6-144294-s077.zip › best_curated_Human_TFs_p1e-6_grch38/B2B_vs_LC2/CREBL2_M07230_1.94d_barcode_B2B30PROseq.tfit_merged_vs_LC2PROseq.tfit_merged.png]

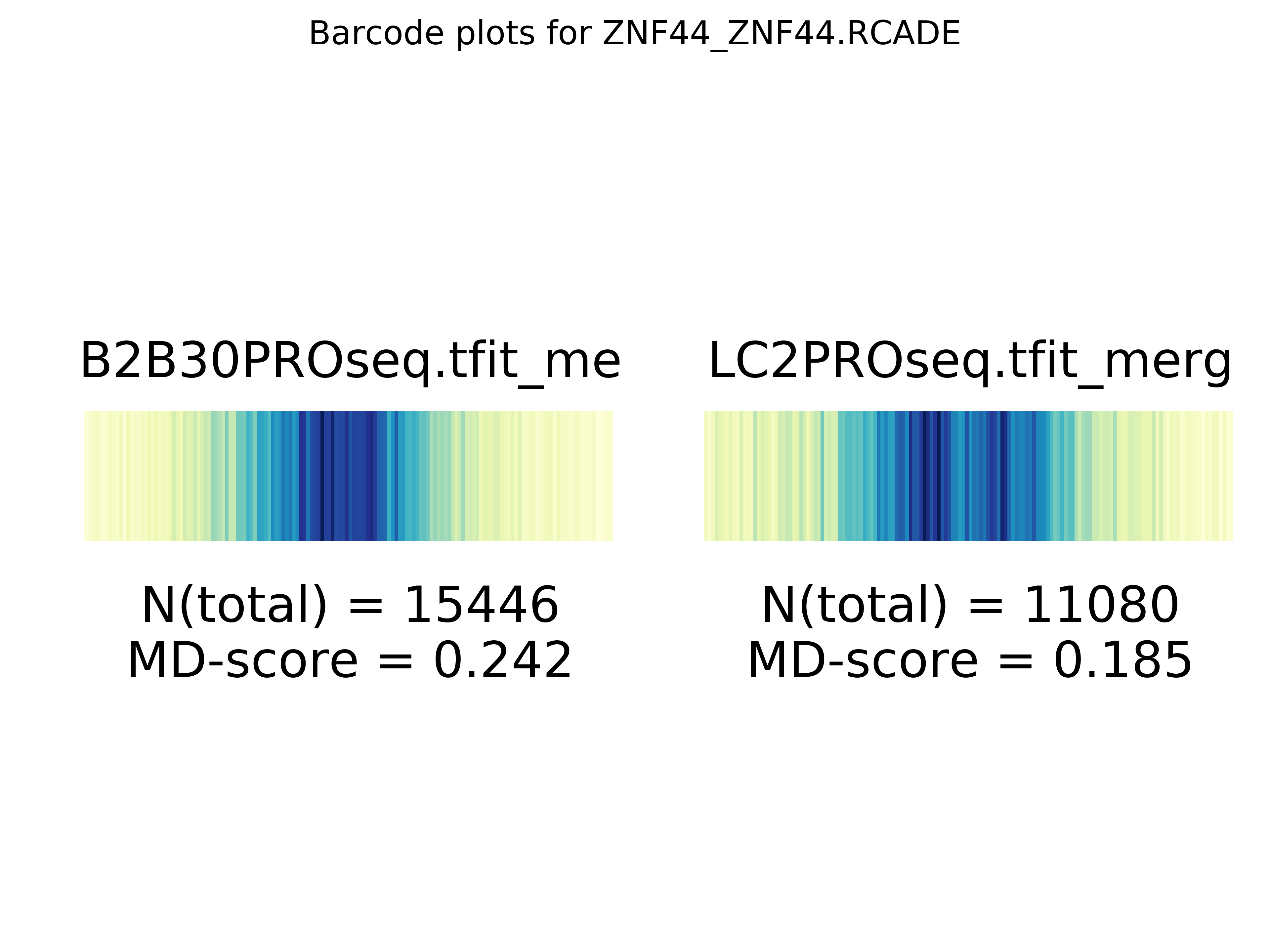

Supplement: Supplemental Data Set 2 [file jciinsight-6-144294-s077.zip › best_curated_Human_TFs_p1e-6_grch38/B2B_vs_LC2/ZNF44_ZNF44.RCADE_barcode_B2B30PROseq.tfit_merged_vs_LC2PROseq.tfit_merged.png]

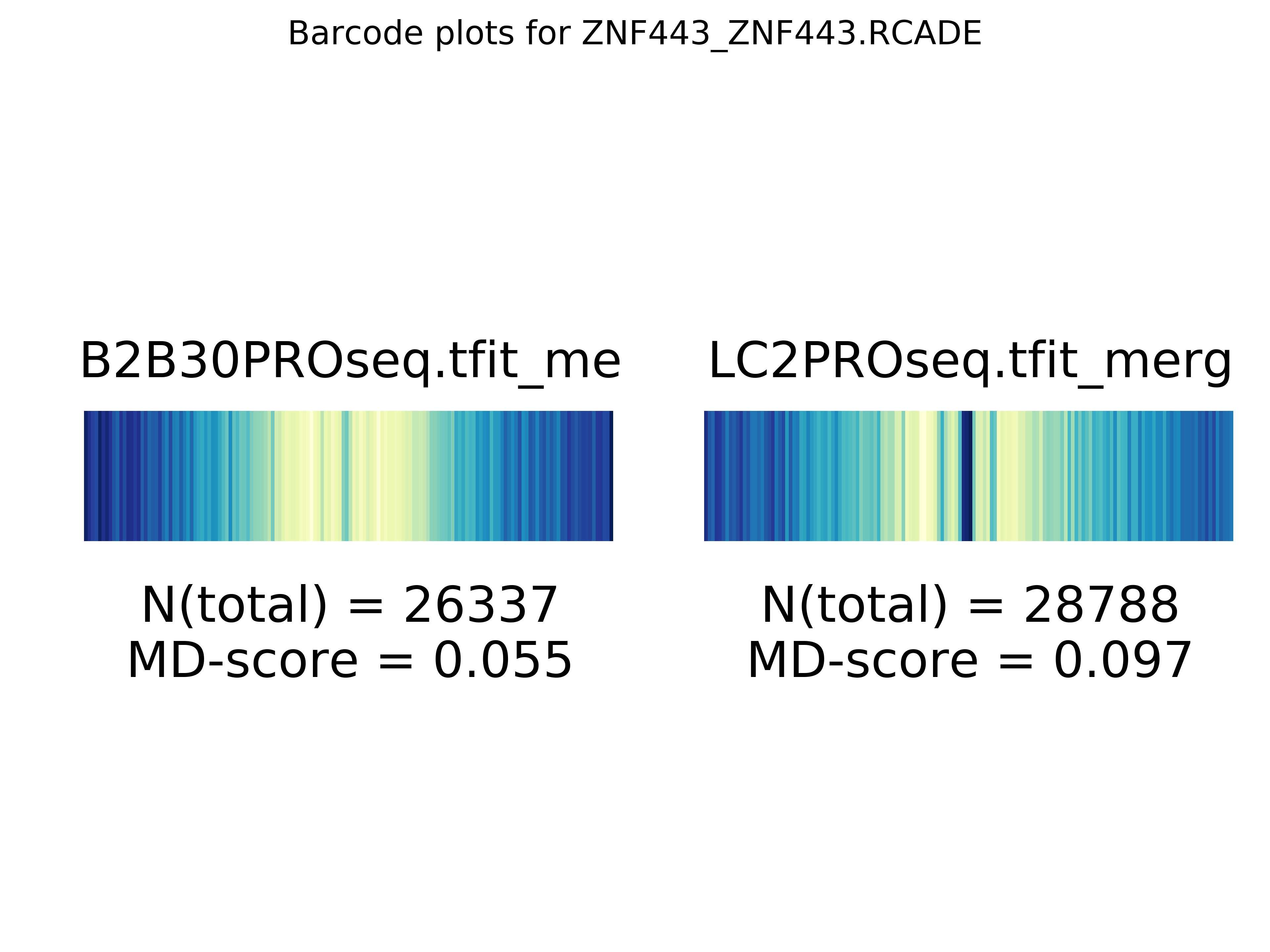

Supplement: Supplemental Data Set 2 [file jciinsight-6-144294-s077.zip › best_curated_Human_TFs_p1e-6_grch38/B2B_vs_LC2/ZNF443_ZNF443.RCADE_barcode_B2B30PROseq.tfit_merged_vs_LC2PROseq.tfit_merged.png]

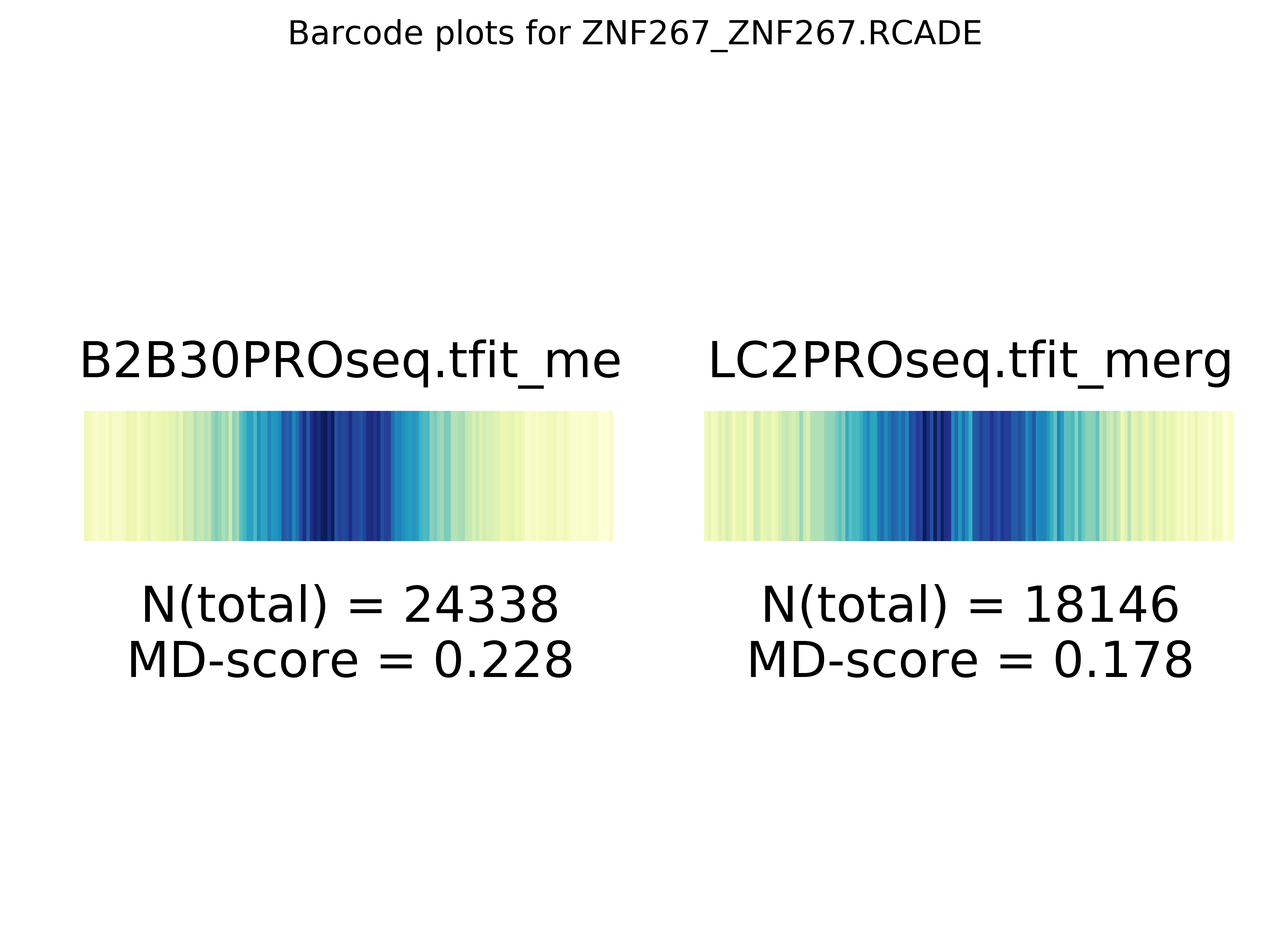

Supplement: Supplemental Data Set 2 [file jciinsight-6-144294-s077.zip › best_curated_Human_TFs_p1e-6_grch38/B2B_vs_LC2/ZNF267_ZNF267.RCADE_barcode_B2B30PROseq.tfit_merged_vs_LC2PROseq.tfit_merged.png]

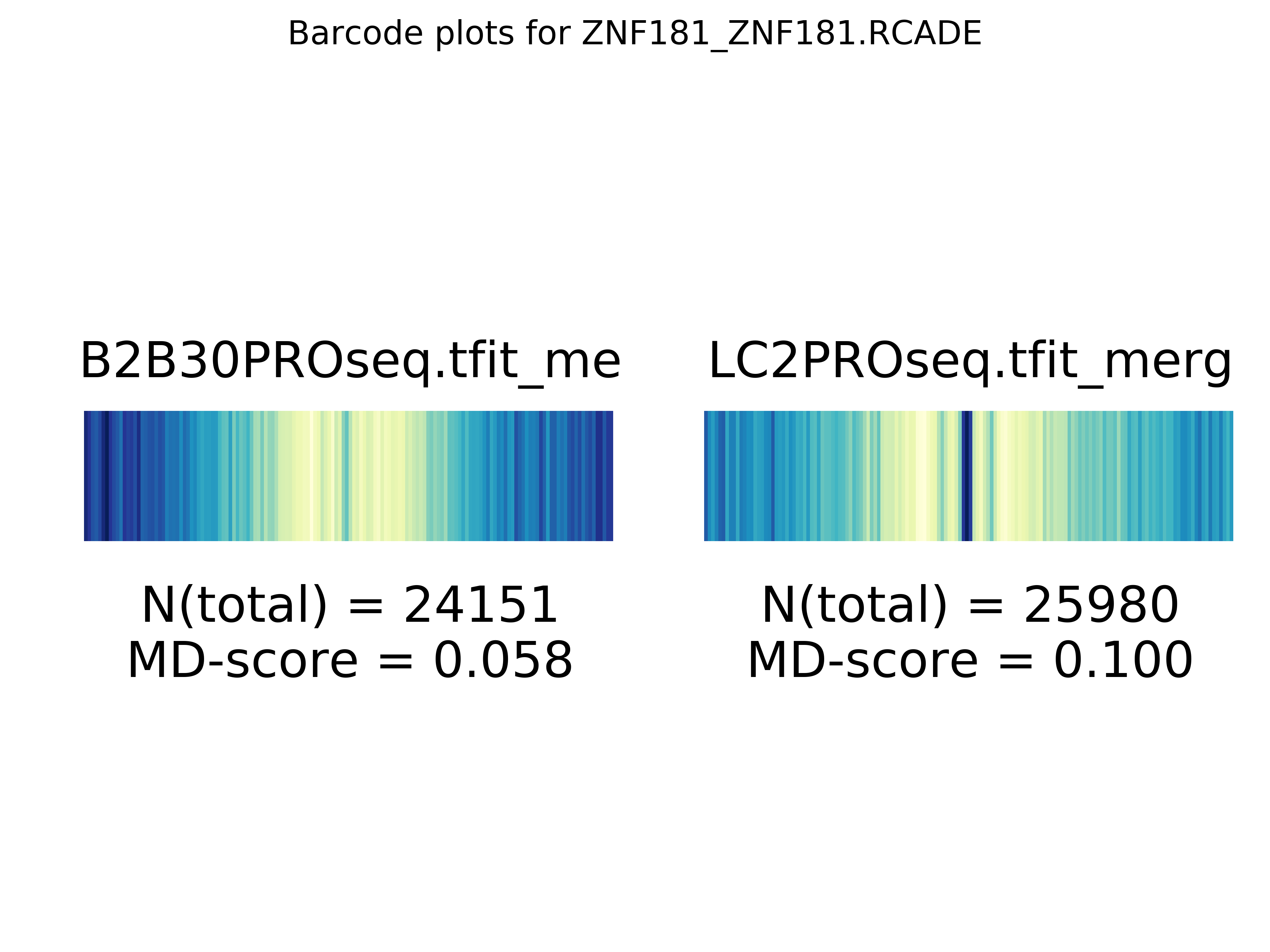

Supplement: Supplemental Data Set 2 [file jciinsight-6-144294-s077.zip › best_curated_Human_TFs_p1e-6_grch38/B2B_vs_LC2/ZNF181_ZNF181.RCADE_barcode_B2B30PROseq.tfit_merged_vs_LC2PROseq.tfit_merged.png]

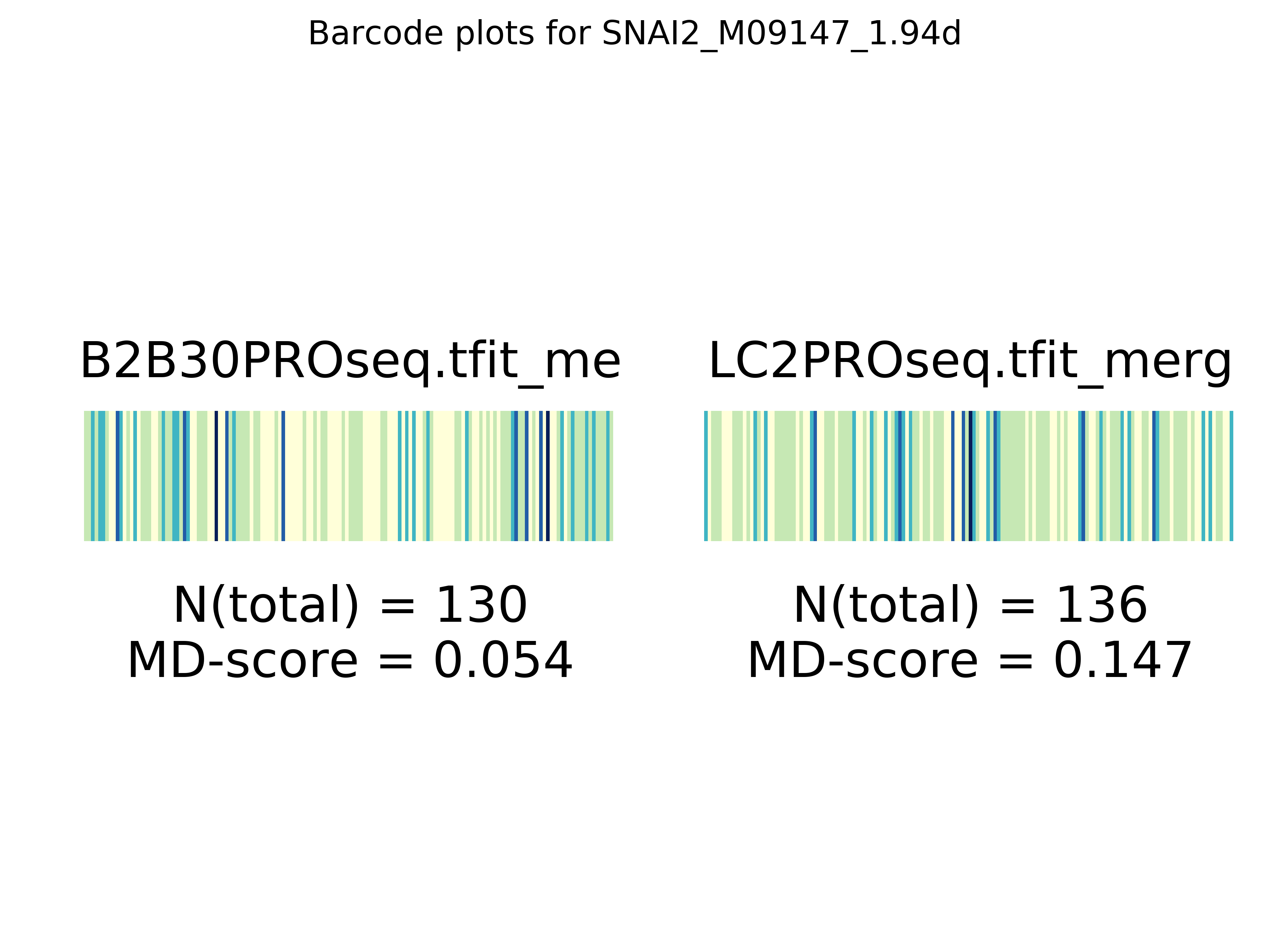

Supplement: Supplemental Data Set 2 [file jciinsight-6-144294-s077.zip › best_curated_Human_TFs_p1e-6_grch38/B2B_vs_LC2/SNAI2_M09147_1.94d_barcode_B2B30PROseq.tfit_merged_vs_LC2PROseq.tfit_merged.png]

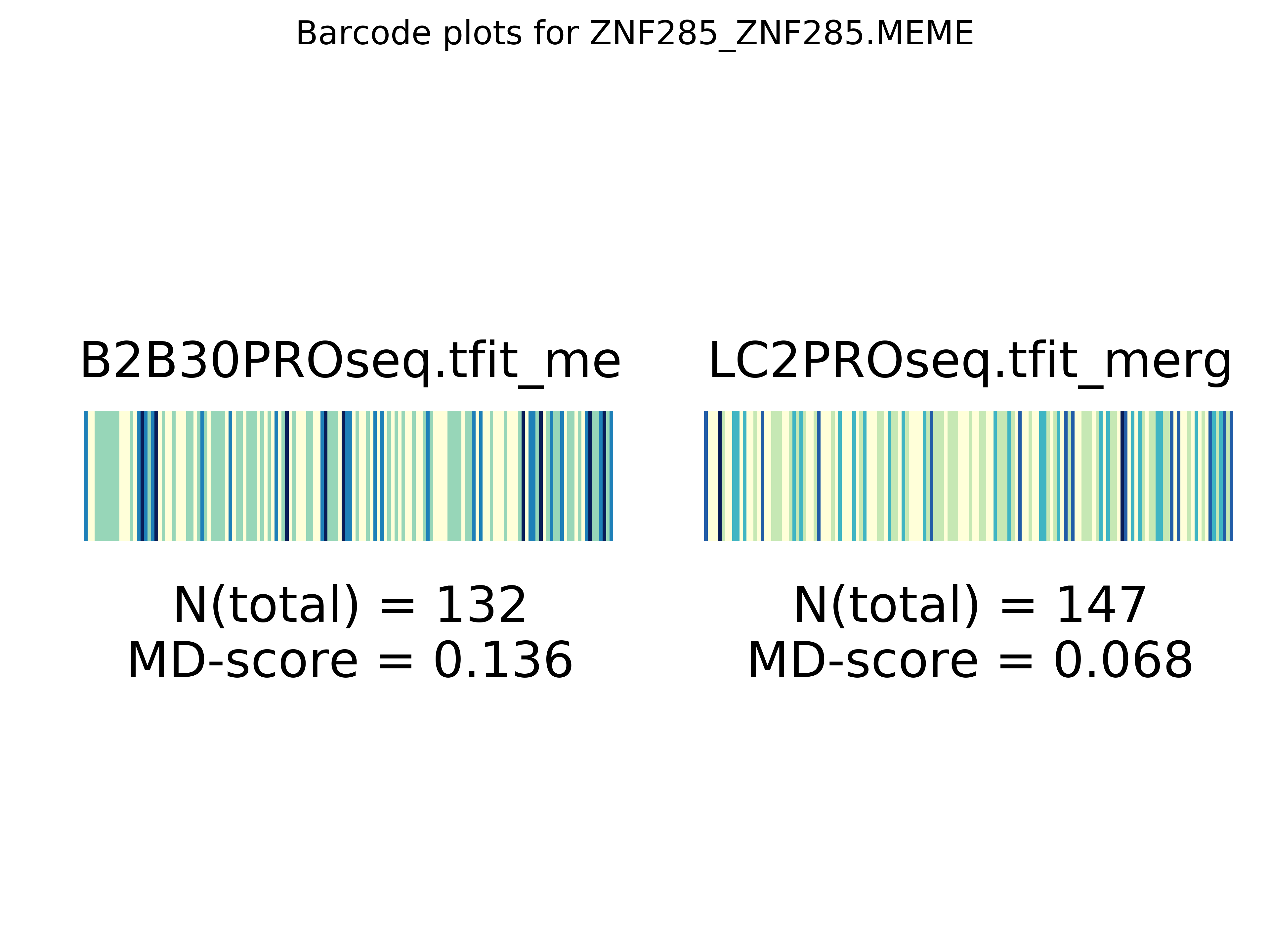

Supplement: Supplemental Data Set 2 [file jciinsight-6-144294-s077.zip › best_curated_Human_TFs_p1e-6_grch38/B2B_vs_LC2/ZNF285_ZNF285.MEME_barcode_B2B30PROseq.tfit_merged_vs_LC2PROseq.tfit_merged.png]

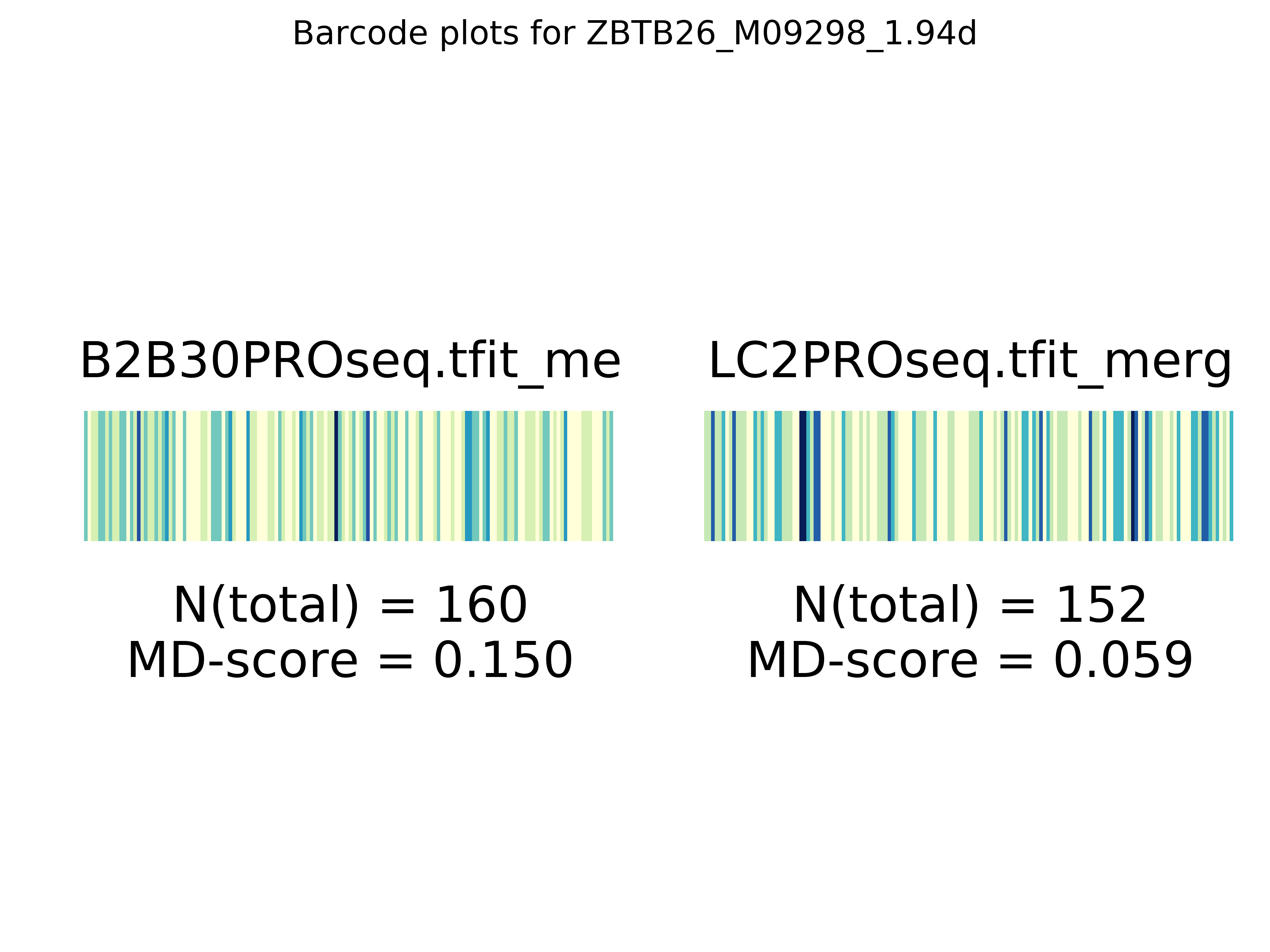

Supplement: Supplemental Data Set 2 [file jciinsight-6-144294-s077.zip › best_curated_Human_TFs_p1e-6_grch38/B2B_vs_LC2/ZBTB26_M09298_1.94d_barcode_B2B30PROseq.tfit_merged_vs_LC2PROseq.tfit_merged.png]

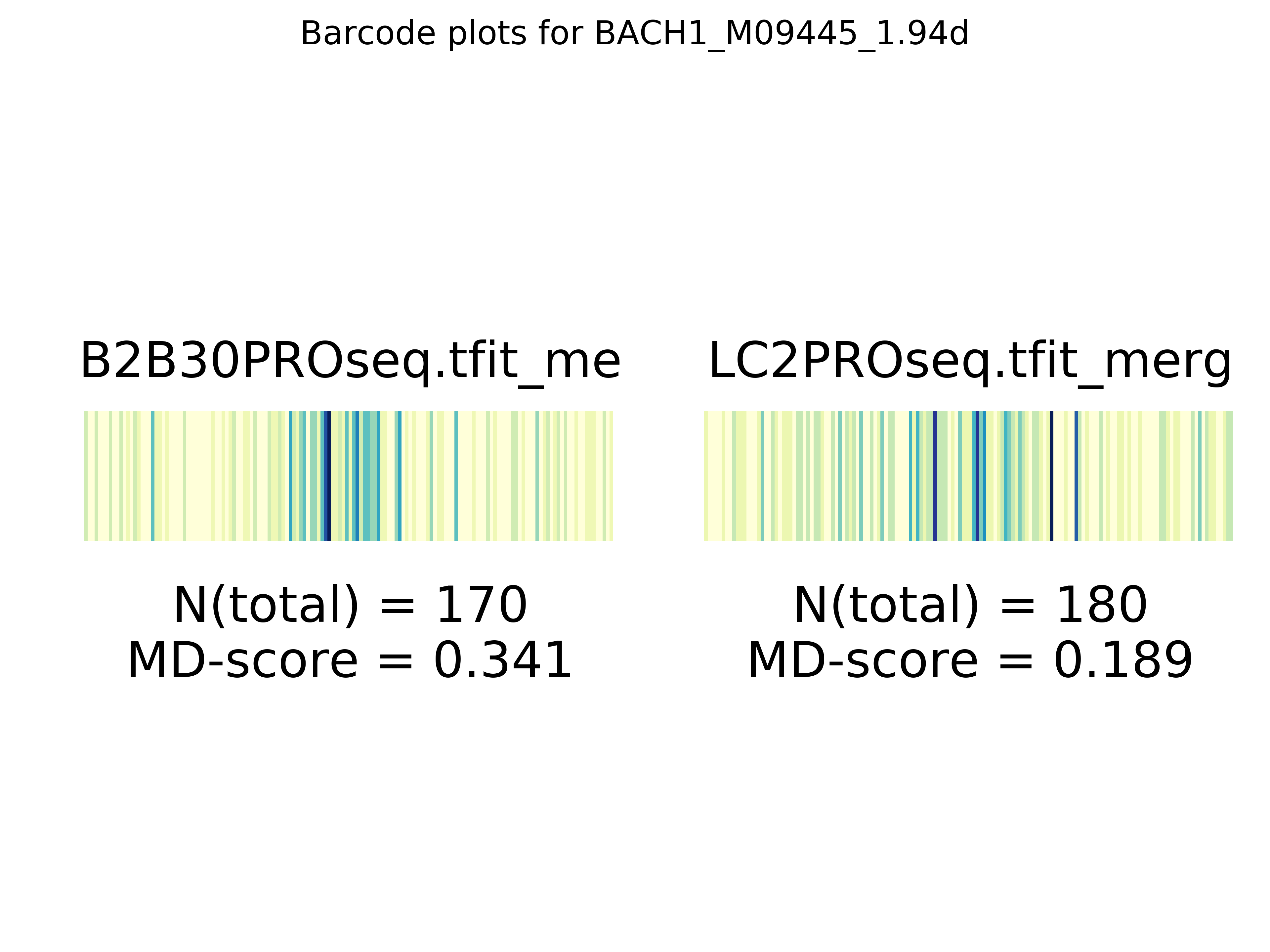

Supplement: Supplemental Data Set 2 [file jciinsight-6-144294-s077.zip › best_curated_Human_TFs_p1e-6_grch38/B2B_vs_LC2/BACH1_M09445_1.94d_barcode_B2B30PROseq.tfit_merged_vs_LC2PROseq.tfit_merged.png]

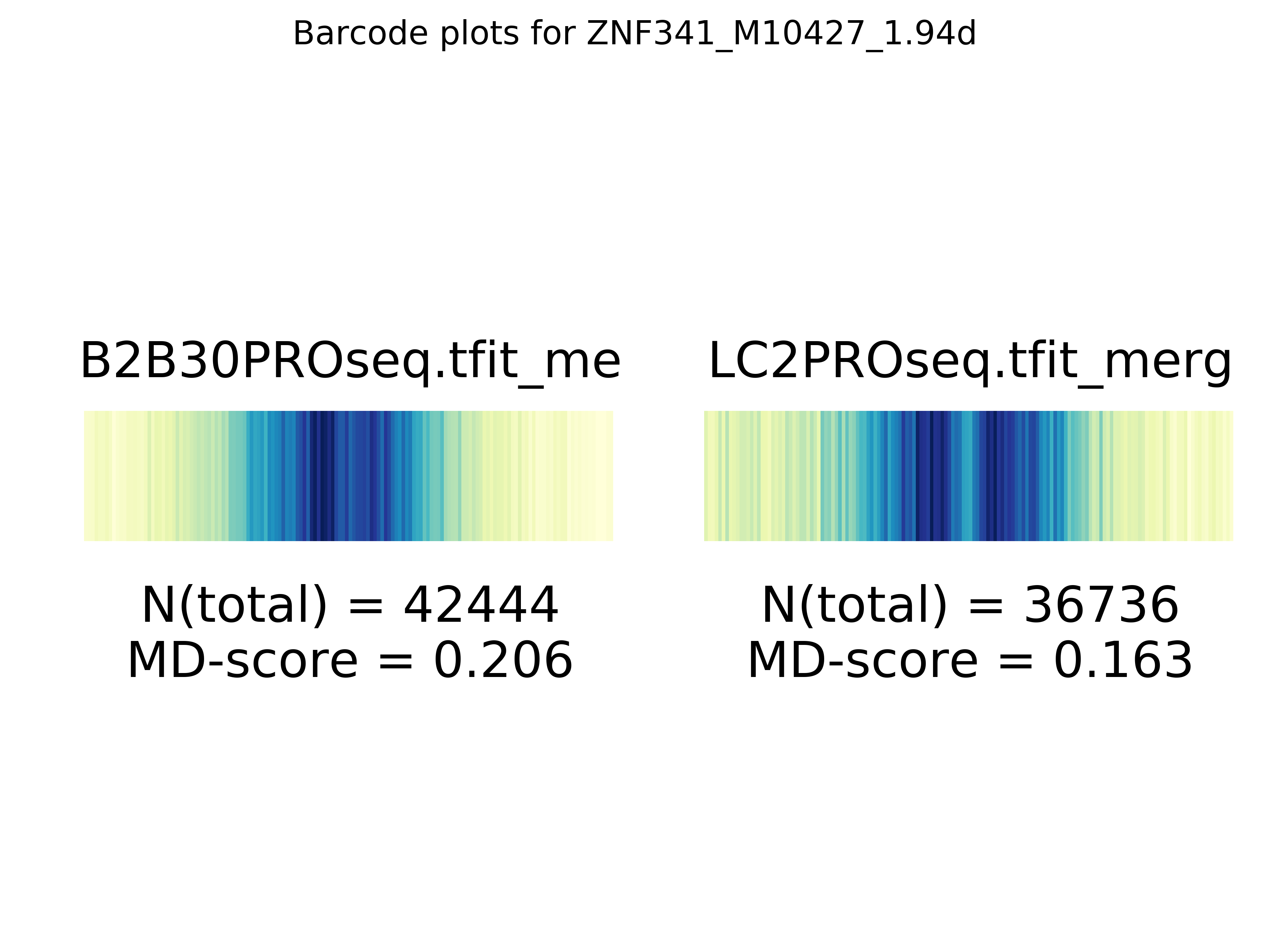

Supplement: Supplemental Data Set 2 [file jciinsight-6-144294-s077.zip › best_curated_Human_TFs_p1e-6_grch38/B2B_vs_LC2/ZNF341_M10427_1.94d_barcode_B2B30PROseq.tfit_merged_vs_LC2PROseq.tfit_merged.png]

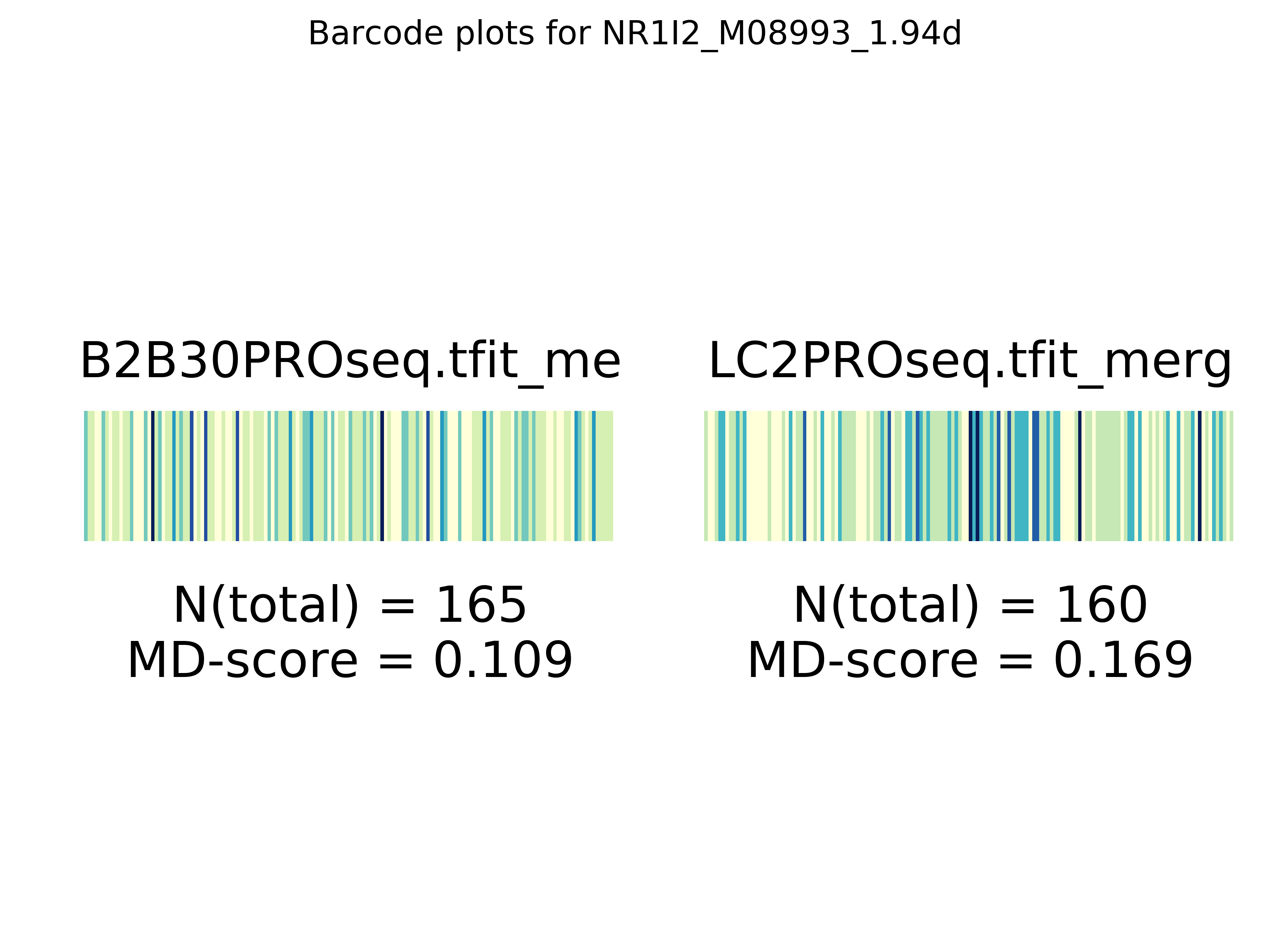

Supplement: Supplemental Data Set 2 [file jciinsight-6-144294-s077.zip › best_curated_Human_TFs_p1e-6_grch38/B2B_vs_LC2/NR1I2_M08993_1.94d_barcode_B2B30PROseq.tfit_merged_vs_LC2PROseq.tfit_merged.png]

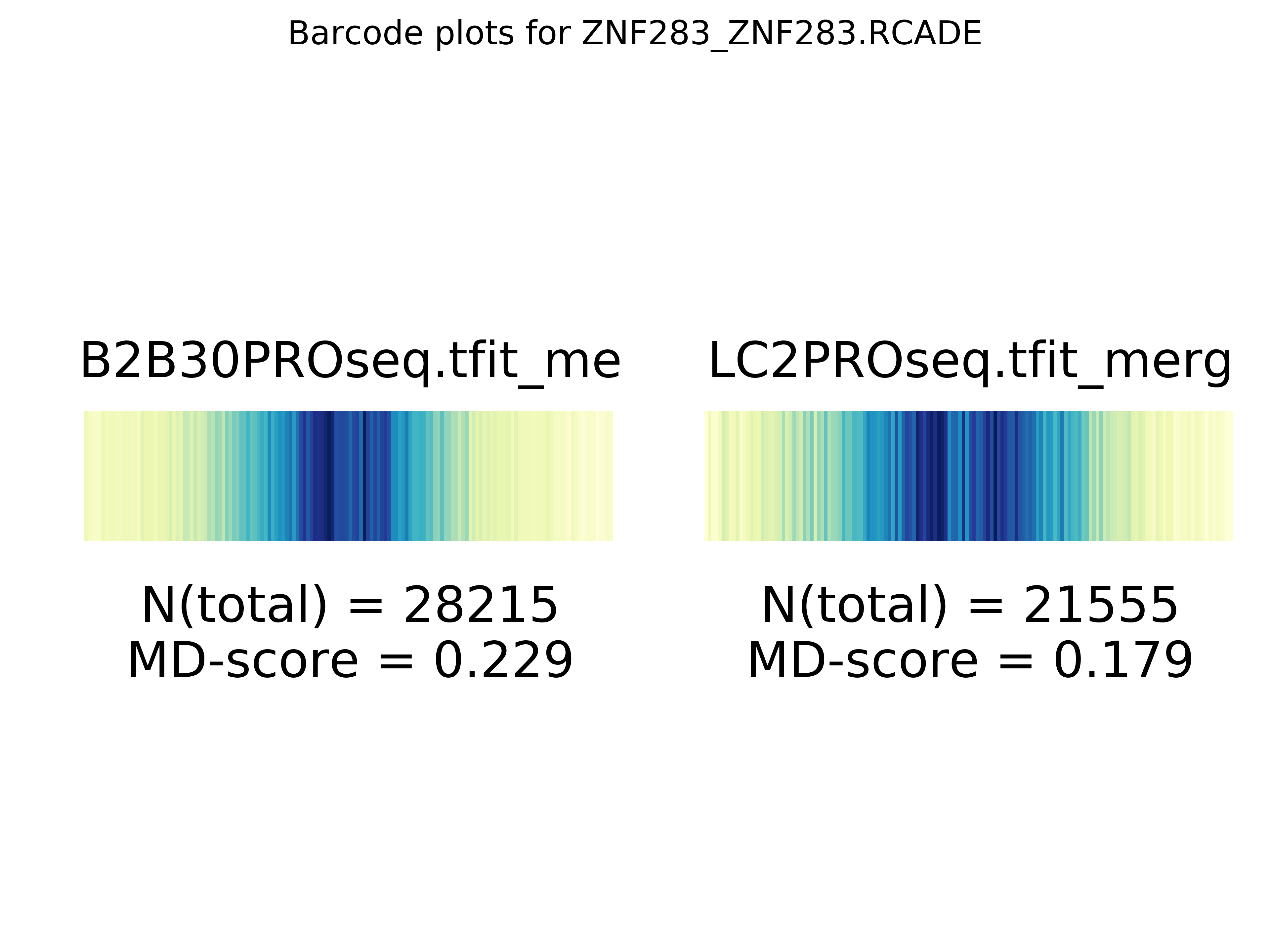

Supplement: Supplemental Data Set 2 [file jciinsight-6-144294-s077.zip › best_curated_Human_TFs_p1e-6_grch38/B2B_vs_LC2/ZNF283_ZNF283.RCADE_barcode_B2B30PROseq.tfit_merged_vs_LC2PROseq.tfit_merged.png]

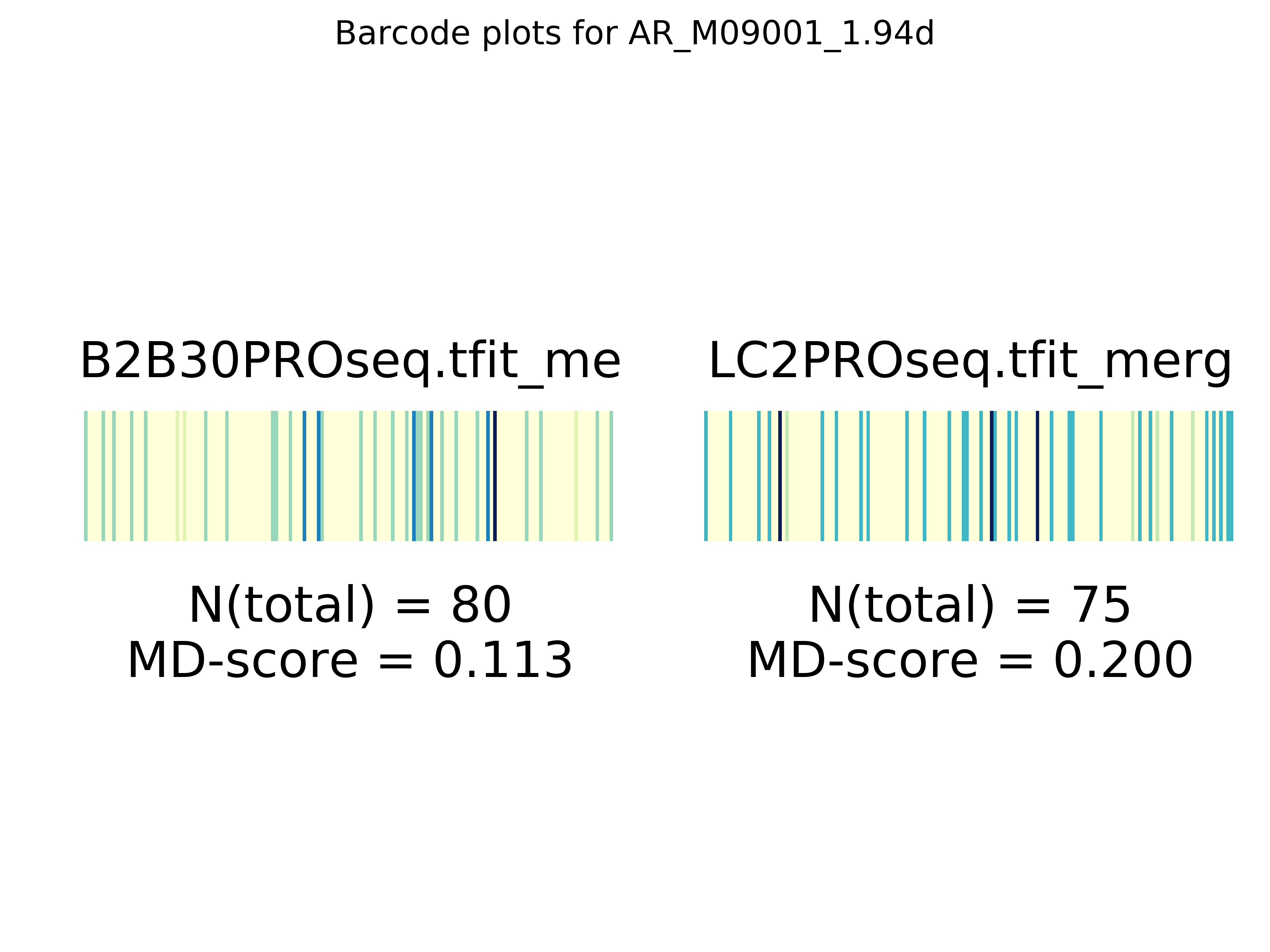

Supplement: Supplemental Data Set 2 [file jciinsight-6-144294-s077.zip › best_curated_Human_TFs_p1e-6_grch38/B2B_vs_LC2/AR_M09001_1.94d_barcode_B2B30PROseq.tfit_merged_vs_LC2PROseq.tfit_merged.png]

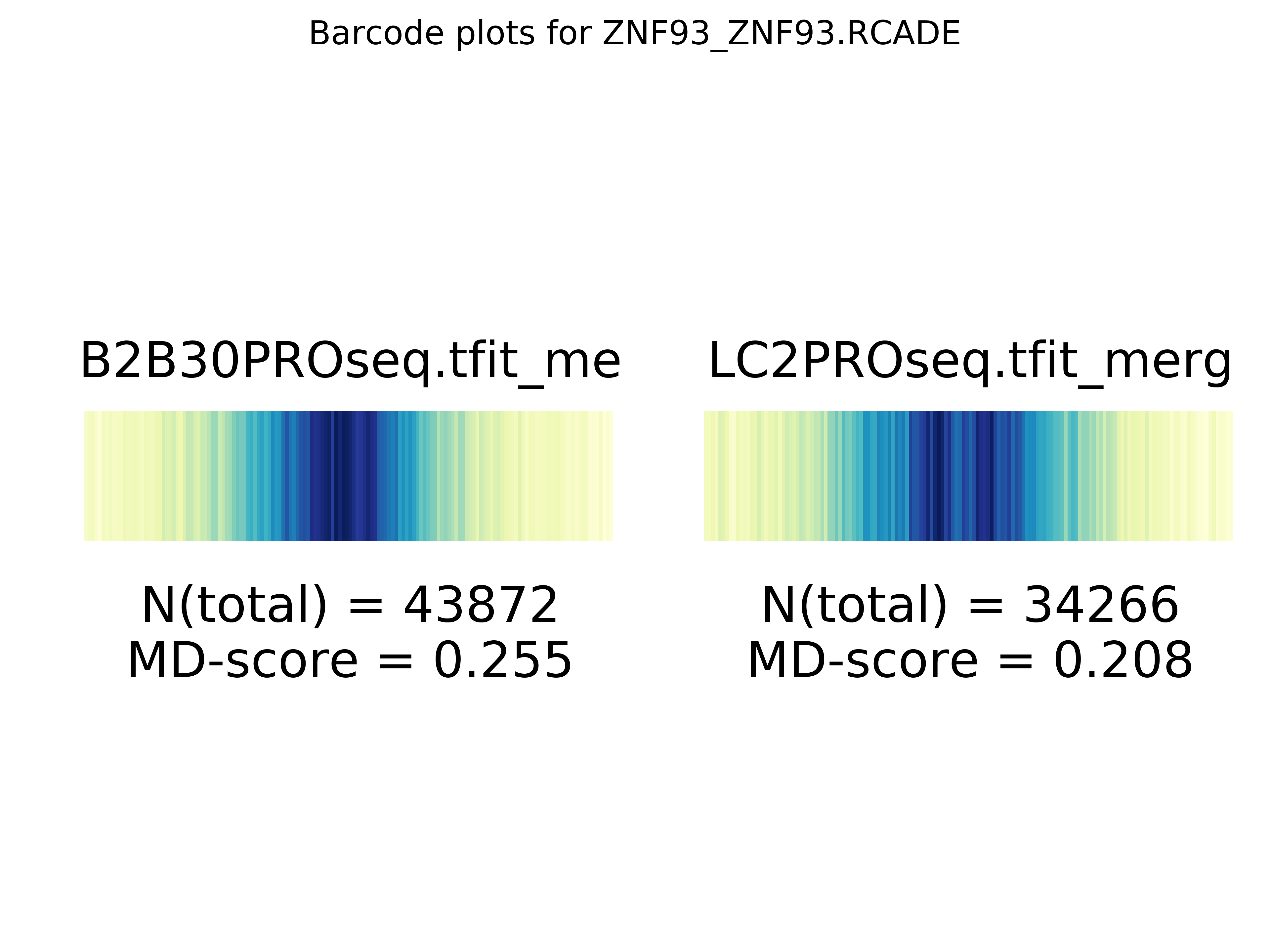

Supplement: Supplemental Data Set 2 [file jciinsight-6-144294-s077.zip › best_curated_Human_TFs_p1e-6_grch38/B2B_vs_LC2/ZNF93_ZNF93.RCADE_barcode_B2B30PROseq.tfit_merged_vs_LC2PROseq.tfit_merged.png]

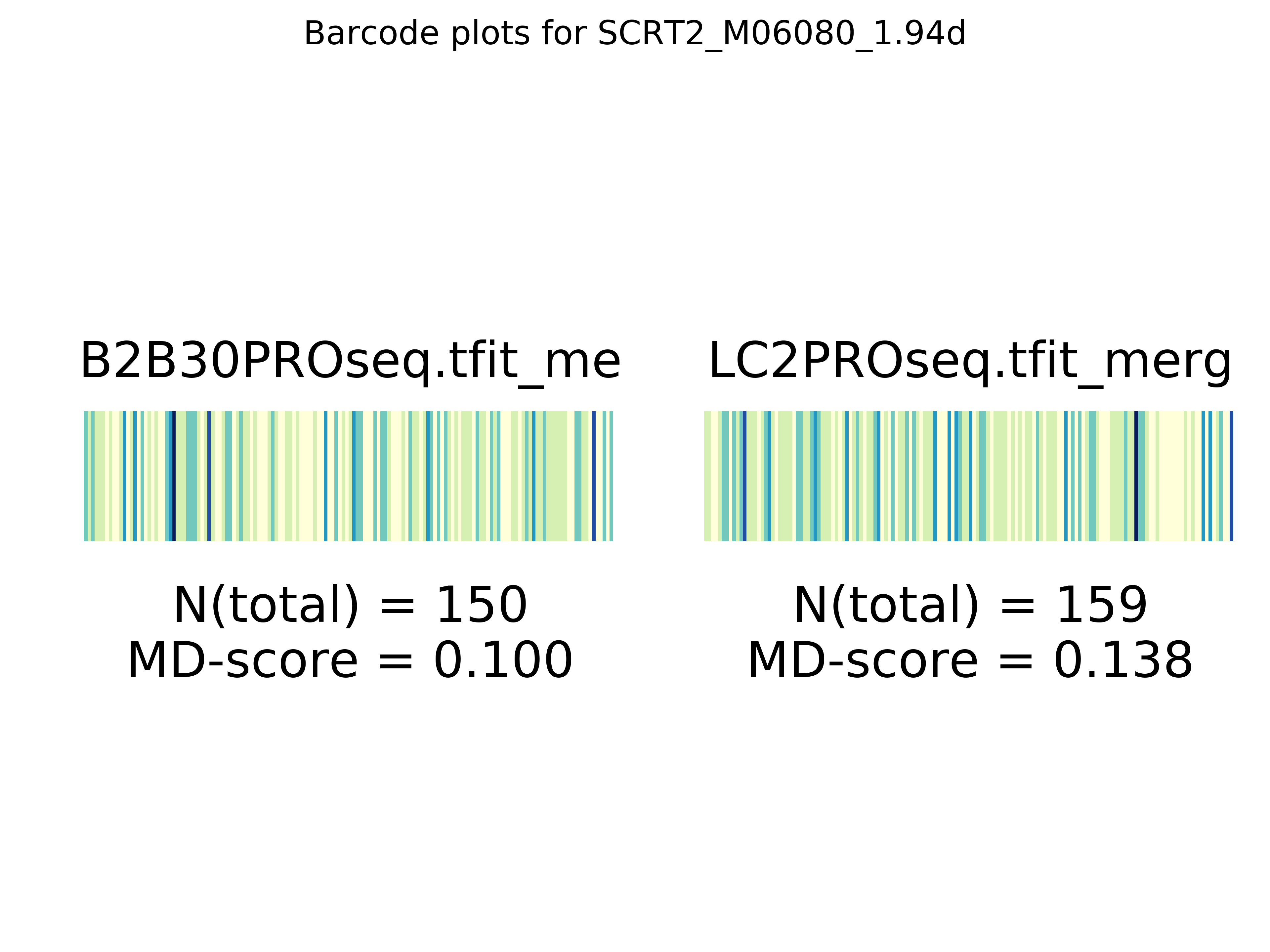

Supplement: Supplemental Data Set 2 [file jciinsight-6-144294-s077.zip › best_curated_Human_TFs_p1e-6_grch38/B2B_vs_LC2/SCRT2_M06080_1.94d_barcode_B2B30PROseq.tfit_merged_vs_LC2PROseq.tfit_merged.png]

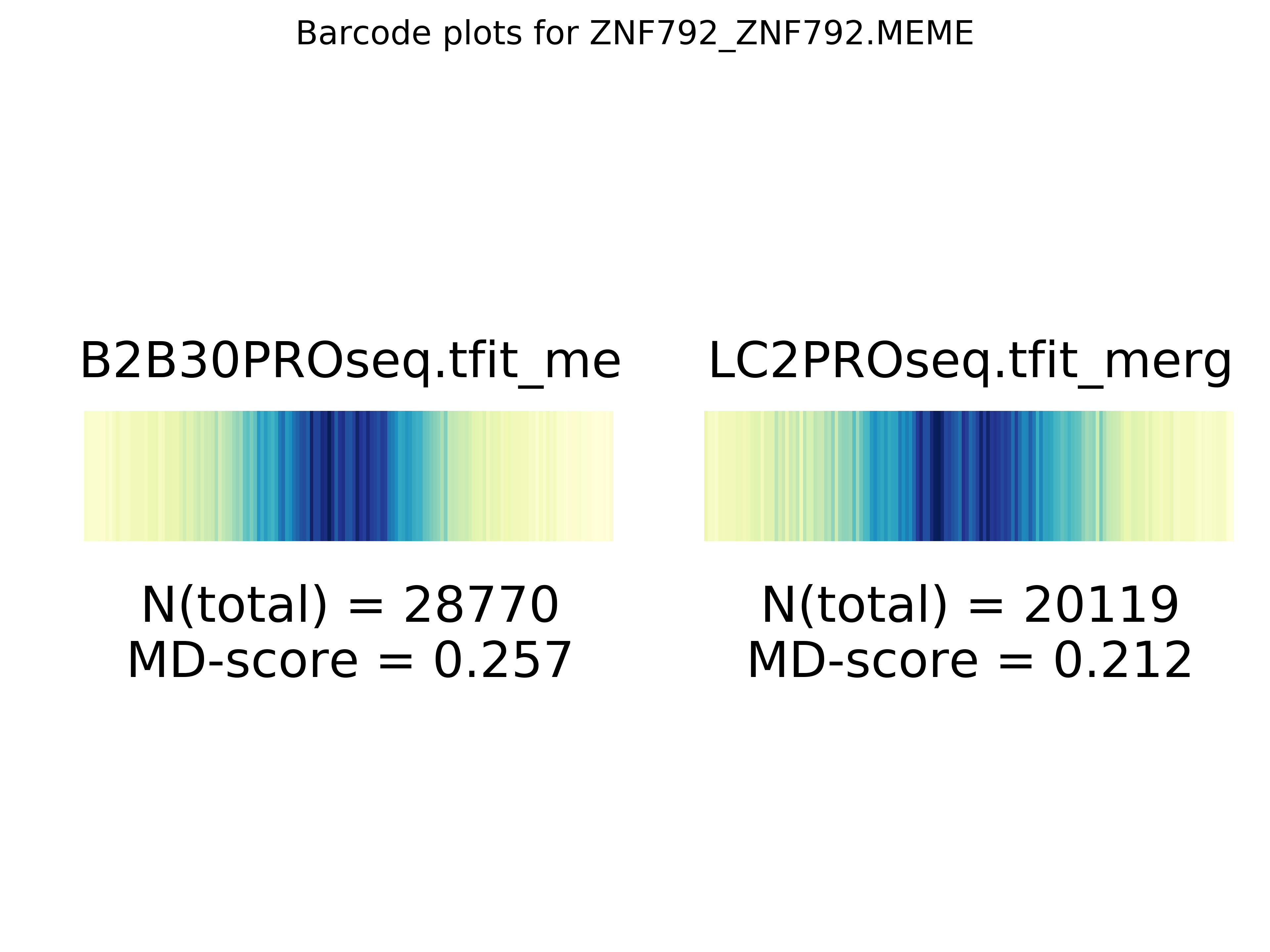

Supplement: Supplemental Data Set 2 [file jciinsight-6-144294-s077.zip › best_curated_Human_TFs_p1e-6_grch38/B2B_vs_LC2/ZNF792_ZNF792.MEME_barcode_B2B30PROseq.tfit_merged_vs_LC2PROseq.tfit_merged.png]

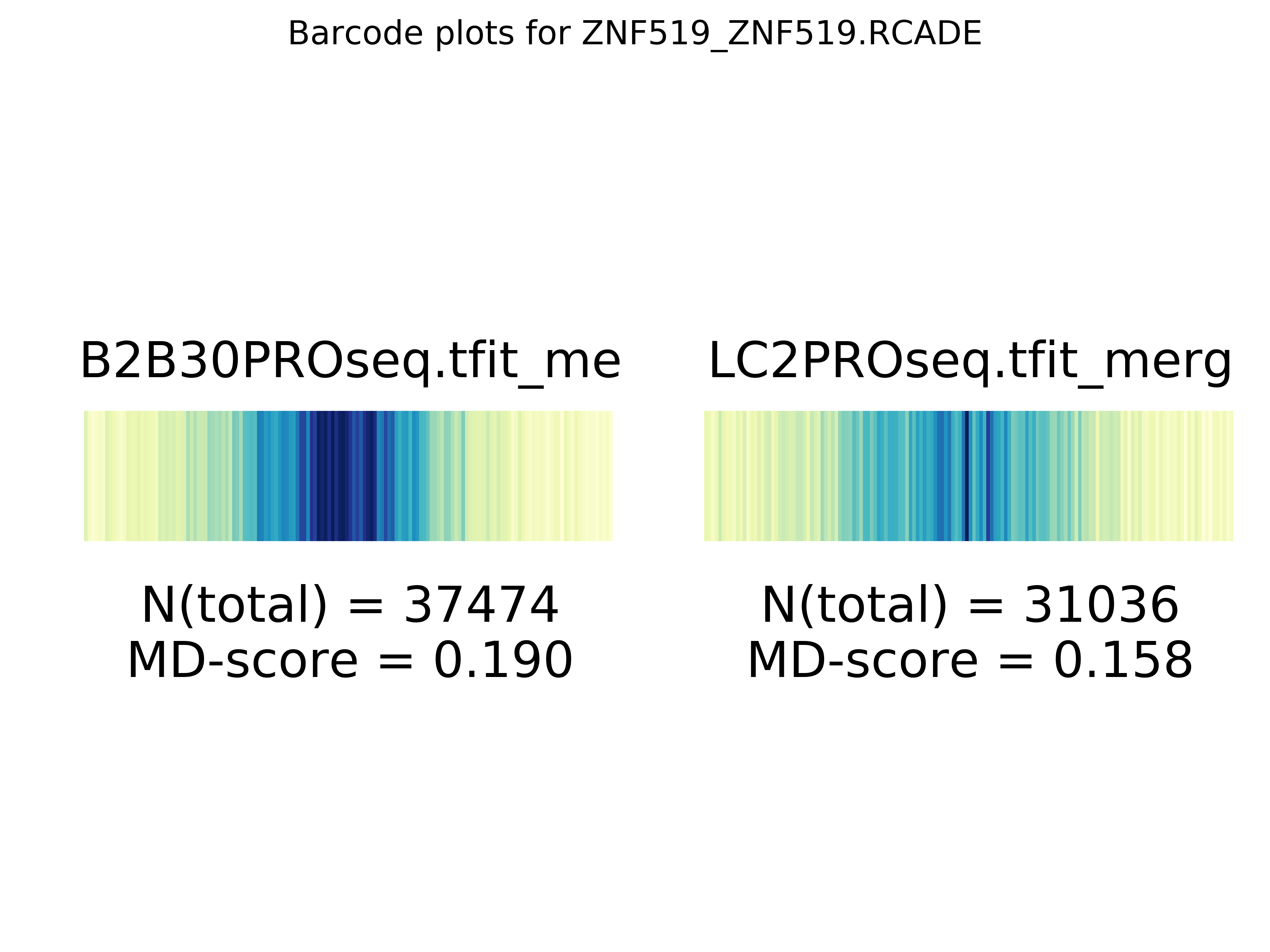

Supplement: Supplemental Data Set 2 [file jciinsight-6-144294-s077.zip › best_curated_Human_TFs_p1e-6_grch38/B2B_vs_LC2/ZNF519_ZNF519.RCADE_barcode_B2B30PROseq.tfit_merged_vs_LC2PROseq.tfit_merged.png]

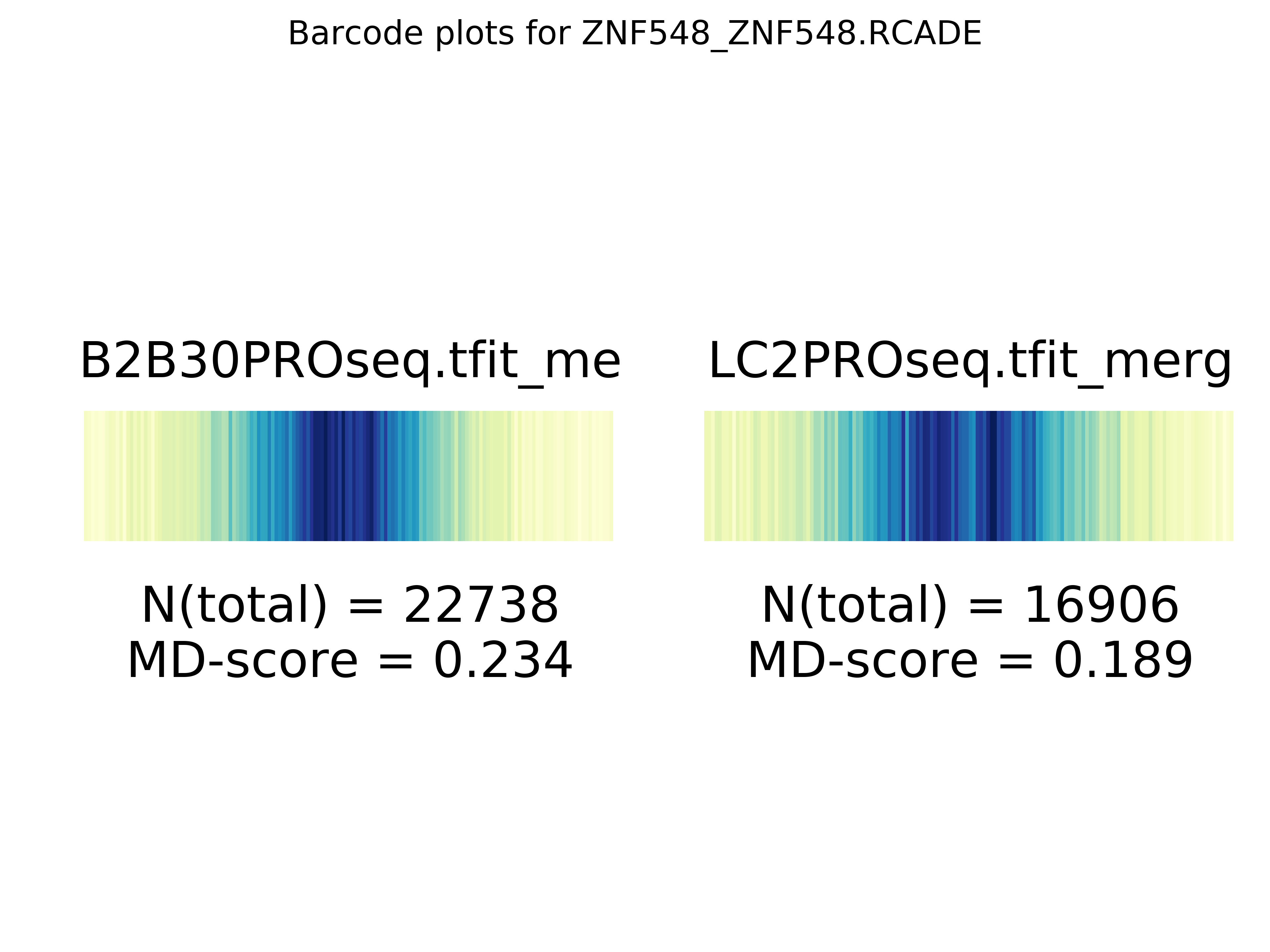

Supplement: Supplemental Data Set 2 [file jciinsight-6-144294-s077.zip › best_curated_Human_TFs_p1e-6_grch38/B2B_vs_LC2/ZNF548_ZNF548.RCADE_barcode_B2B30PROseq.tfit_merged_vs_LC2PROseq.tfit_merged.png]

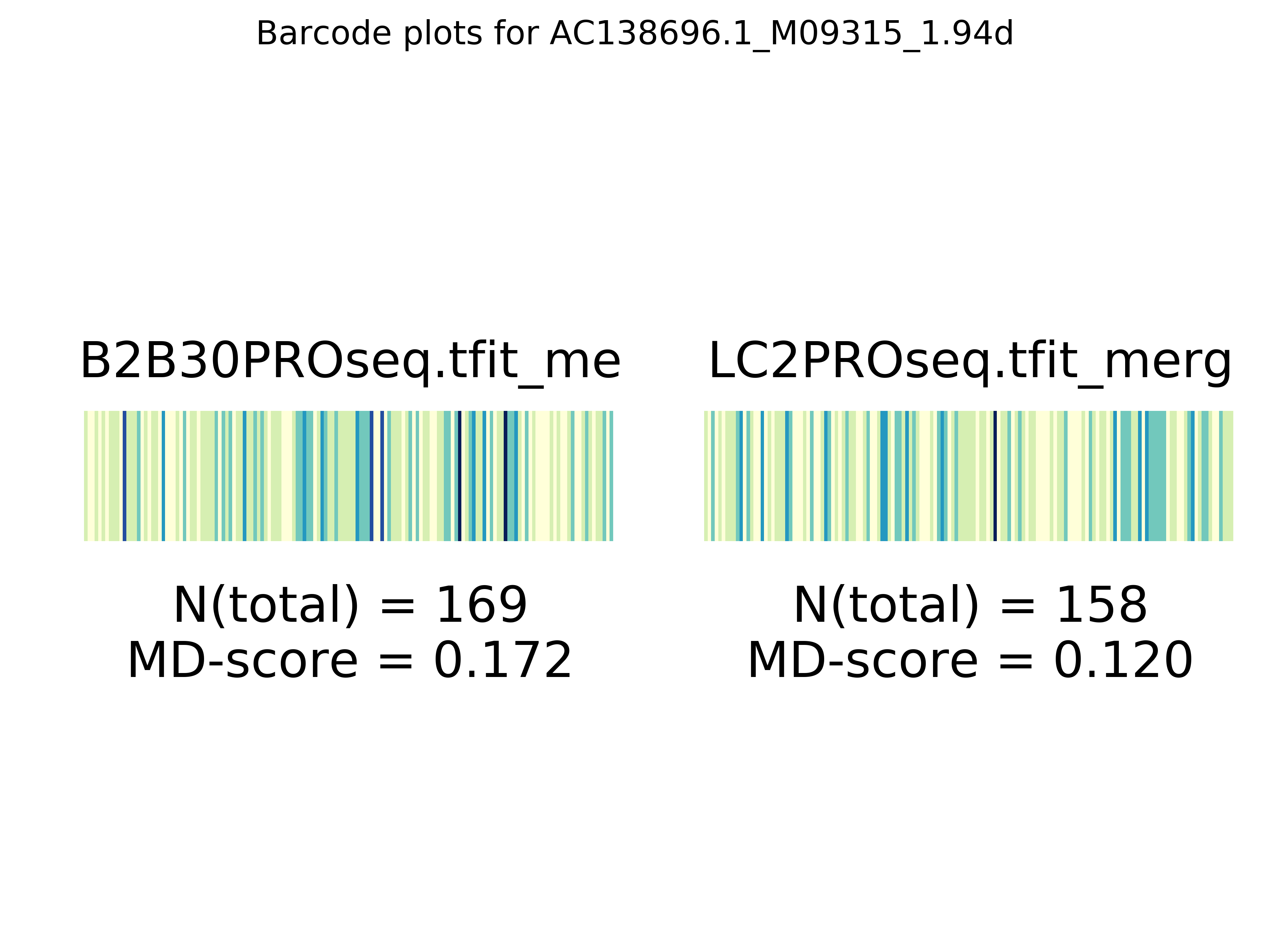

Supplement: Supplemental Data Set 2 [file jciinsight-6-144294-s077.zip › best_curated_Human_TFs_p1e-6_grch38/B2B_vs_LC2/AC138696.1_M09315_1.94d_barcode_B2B30PROseq.tfit_merged_vs_LC2PROseq.tfit_merged.png]

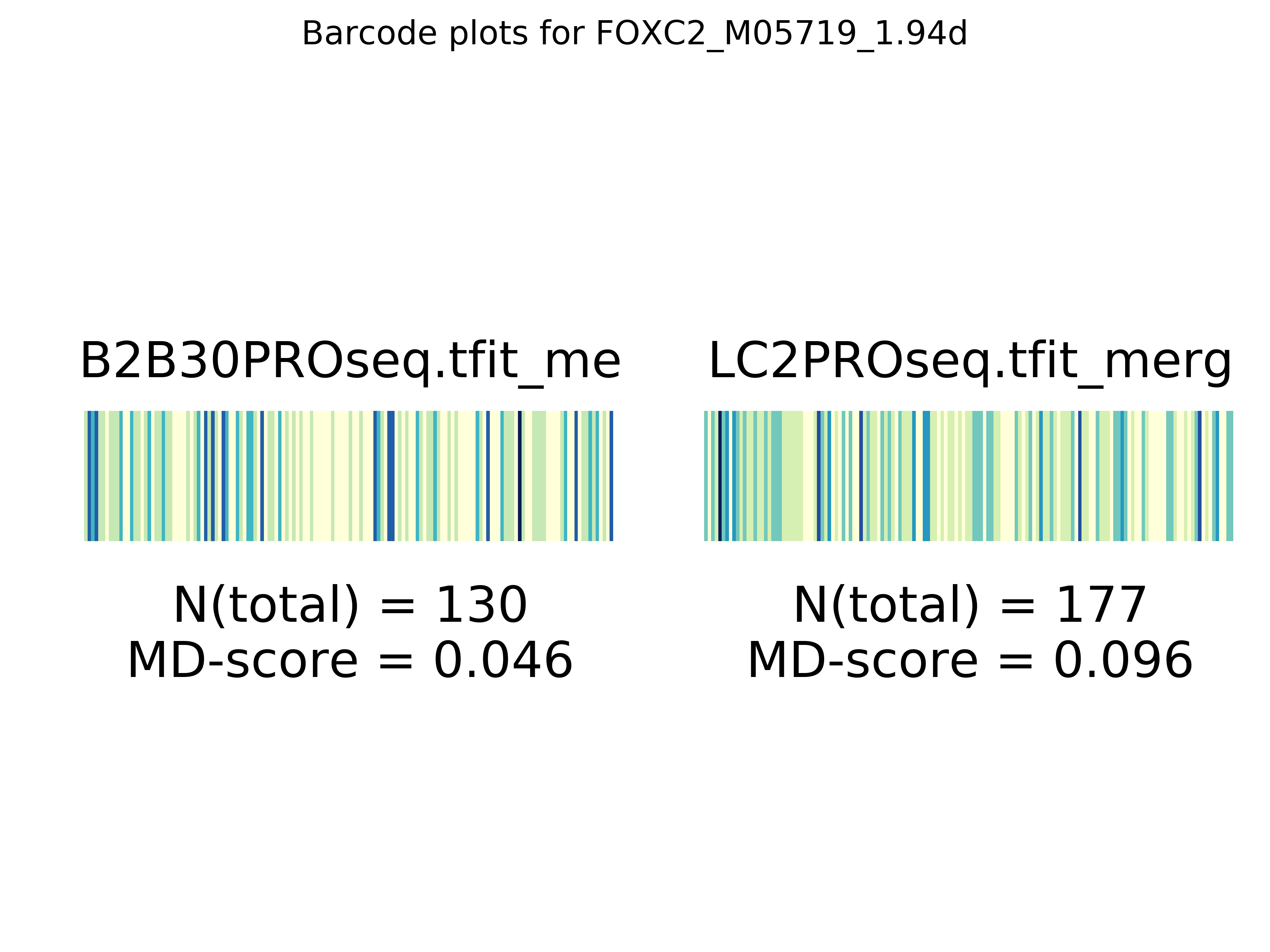

Supplement: Supplemental Data Set 2 [file jciinsight-6-144294-s077.zip › best_curated_Human_TFs_p1e-6_grch38/B2B_vs_LC2/FOXC2_M05719_1.94d_barcode_B2B30PROseq.tfit_merged_vs_LC2PROseq.tfit_merged.png]

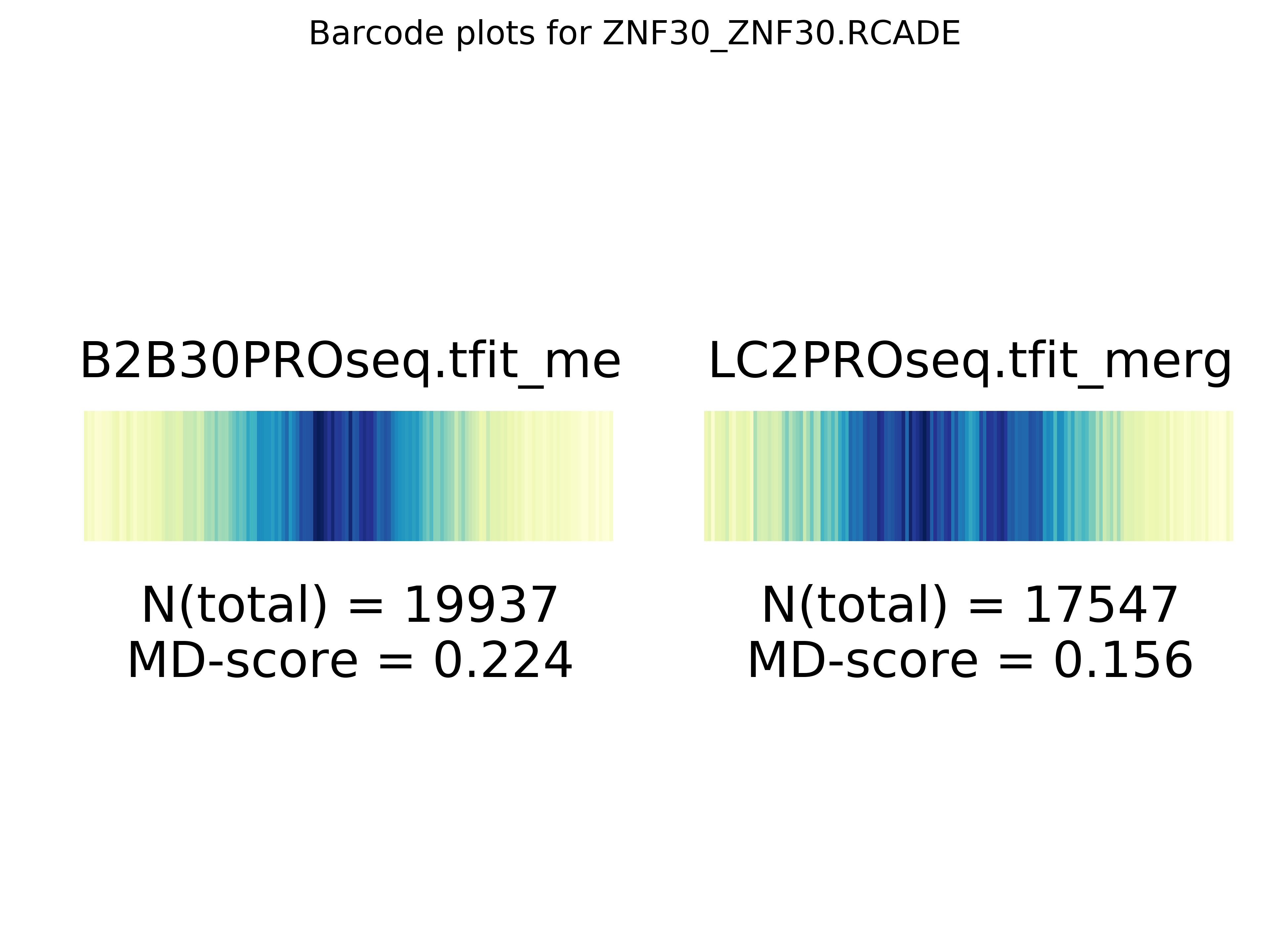

Supplement: Supplemental Data Set 2 [file jciinsight-6-144294-s077.zip › best_curated_Human_TFs_p1e-6_grch38/B2B_vs_LC2/ZNF30_ZNF30.RCADE_barcode_B2B30PROseq.tfit_merged_vs_LC2PROseq.tfit_merged.png]

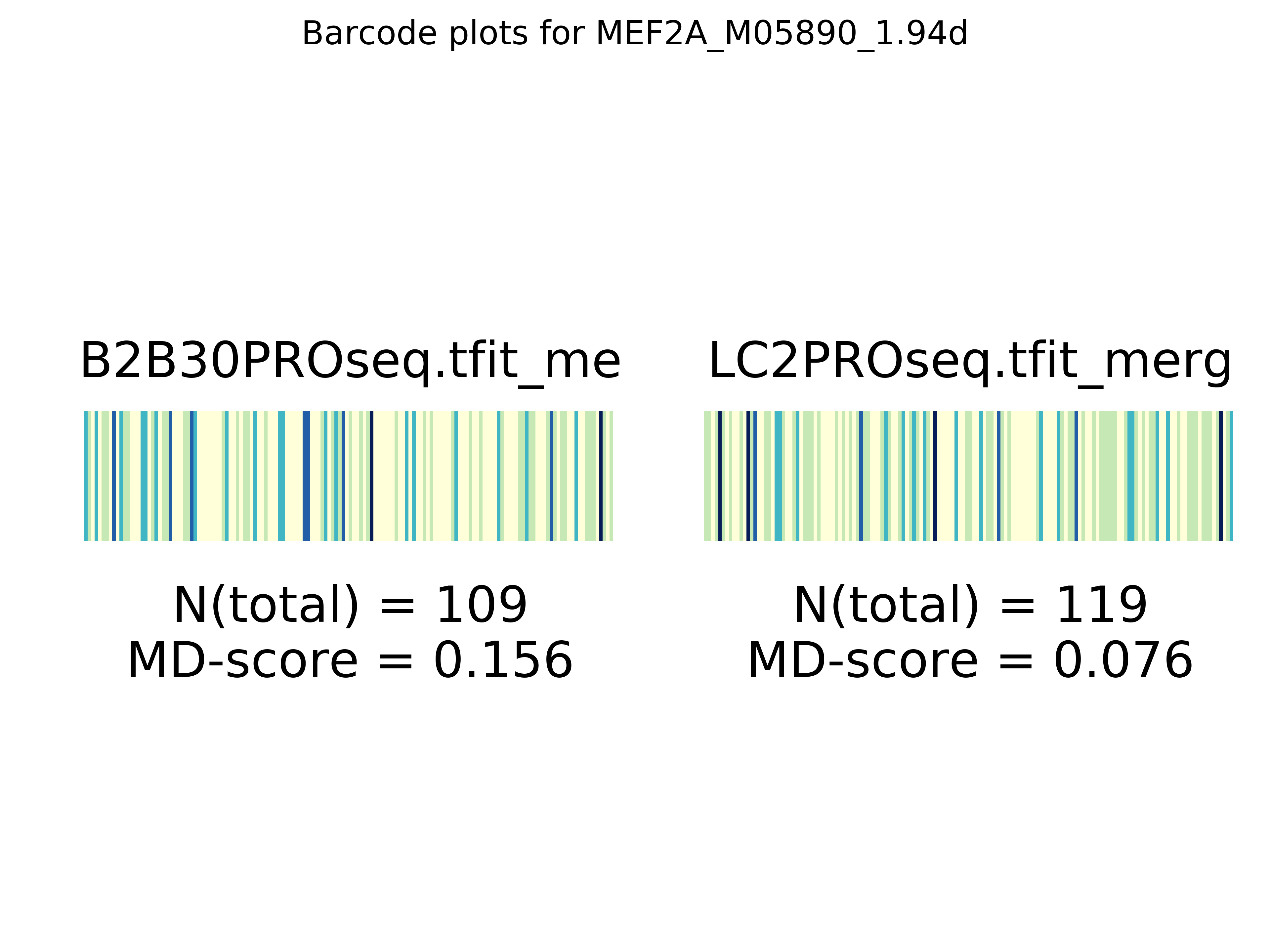

Supplement: Supplemental Data Set 2 [file jciinsight-6-144294-s077.zip › best_curated_Human_TFs_p1e-6_grch38/B2B_vs_LC2/MEF2A_M05890_1.94d_barcode_B2B30PROseq.tfit_merged_vs_LC2PROseq.tfit_merged.png]

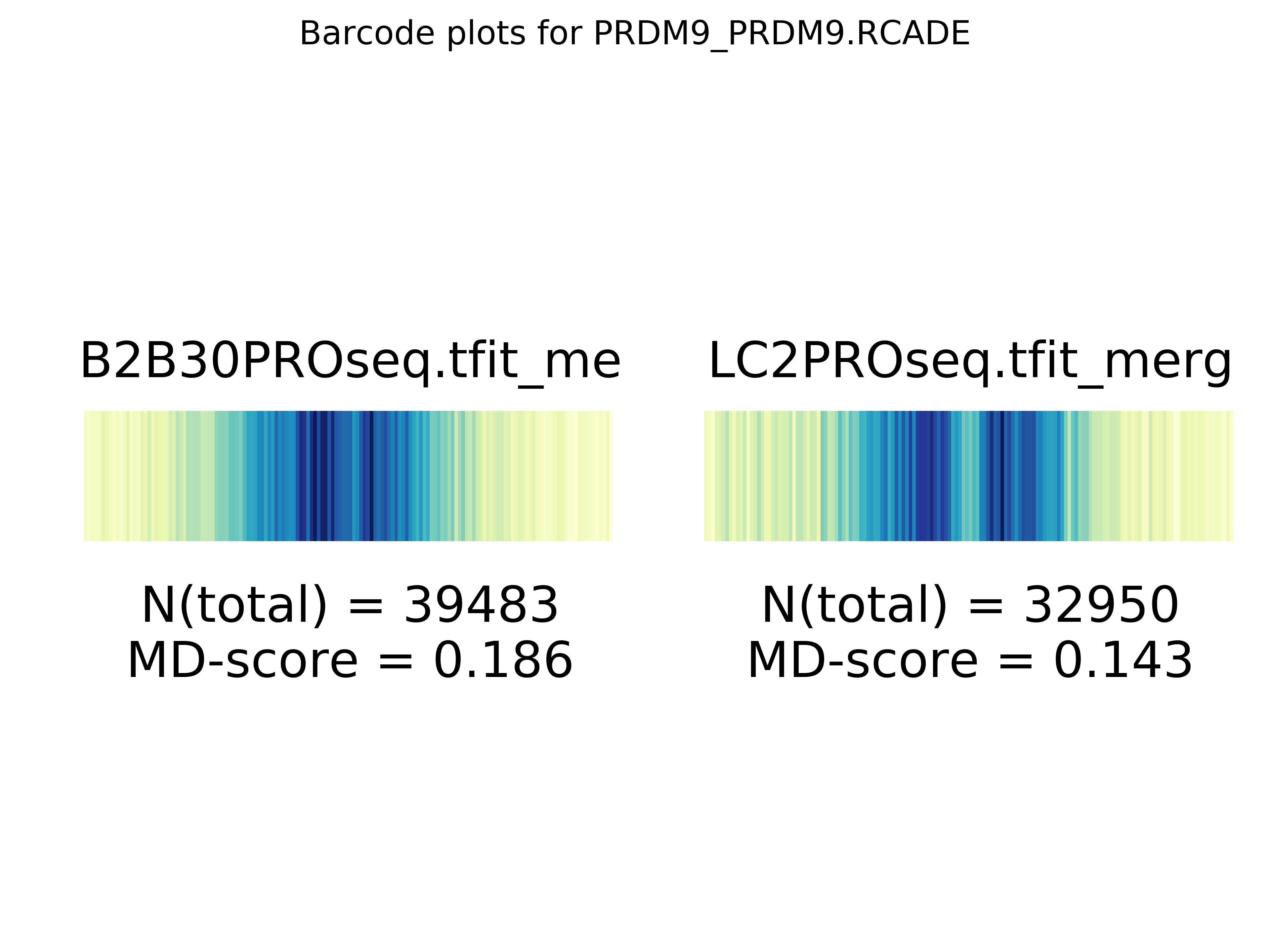

Supplement: Supplemental Data Set 2 [file jciinsight-6-144294-s077.zip › best_curated_Human_TFs_p1e-6_grch38/B2B_vs_LC2/PRDM9_PRDM9.RCADE_barcode_B2B30PROseq.tfit_merged_vs_LC2PROseq.tfit_merged.png]

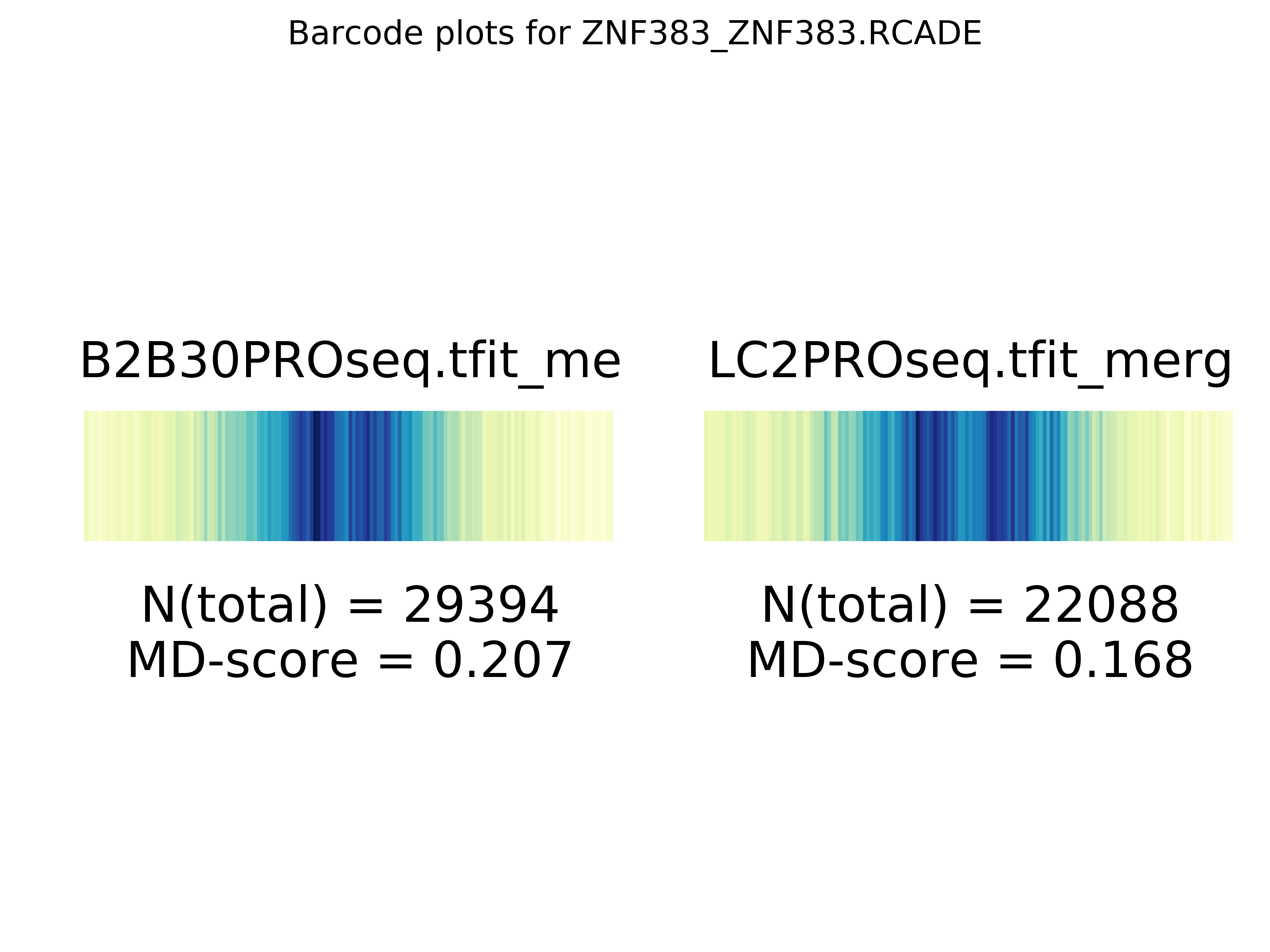

Supplement: Supplemental Data Set 2 [file jciinsight-6-144294-s077.zip › best_curated_Human_TFs_p1e-6_grch38/B2B_vs_LC2/ZNF383_ZNF383.RCADE_barcode_B2B30PROseq.tfit_merged_vs_LC2PROseq.tfit_merged.png]

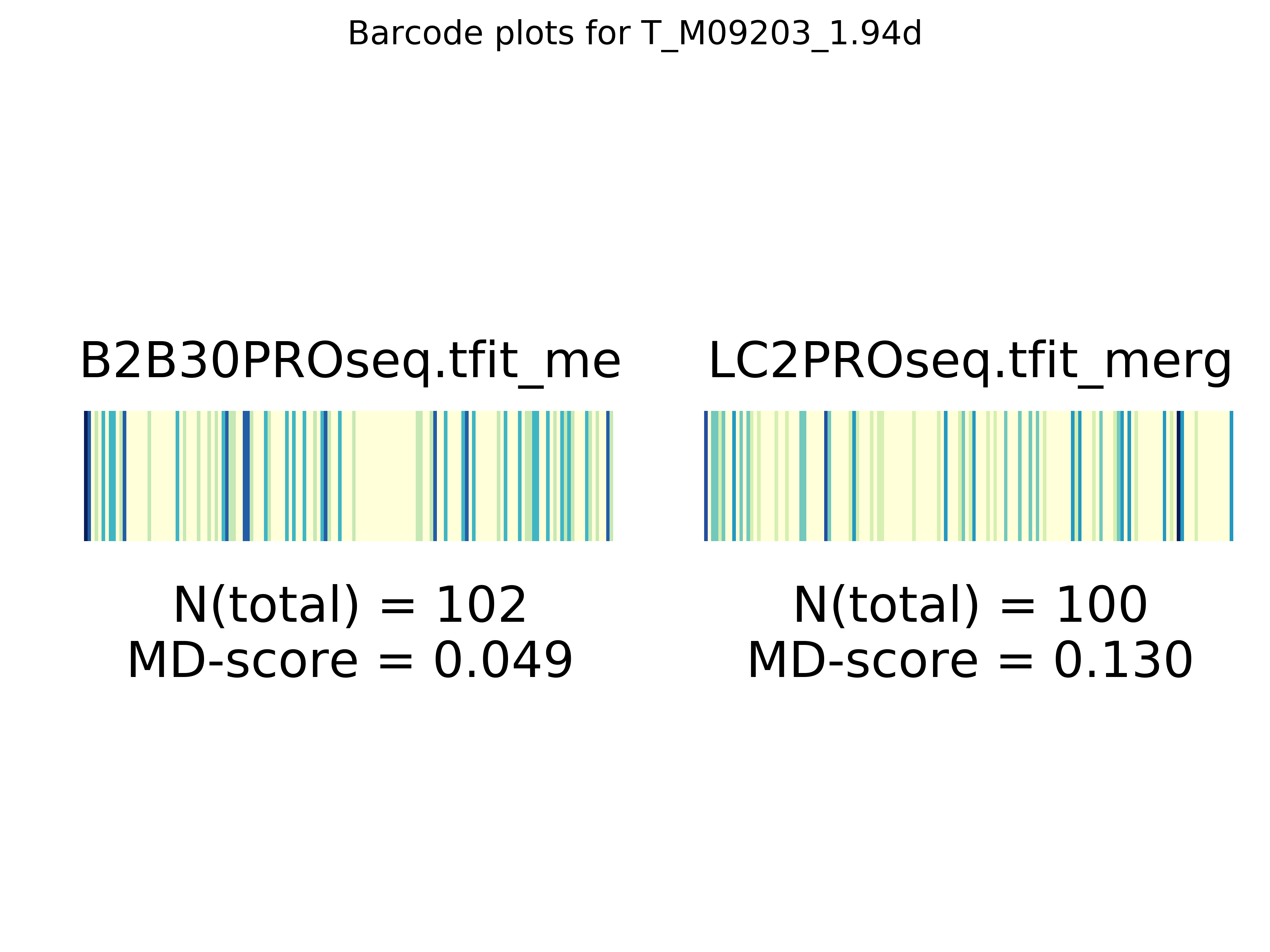

Supplement: Supplemental Data Set 2 [file jciinsight-6-144294-s077.zip › best_curated_Human_TFs_p1e-6_grch38/B2B_vs_LC2/T_M09203_1.94d_barcode_B2B30PROseq.tfit_merged_vs_LC2PROseq.tfit_merged.png]

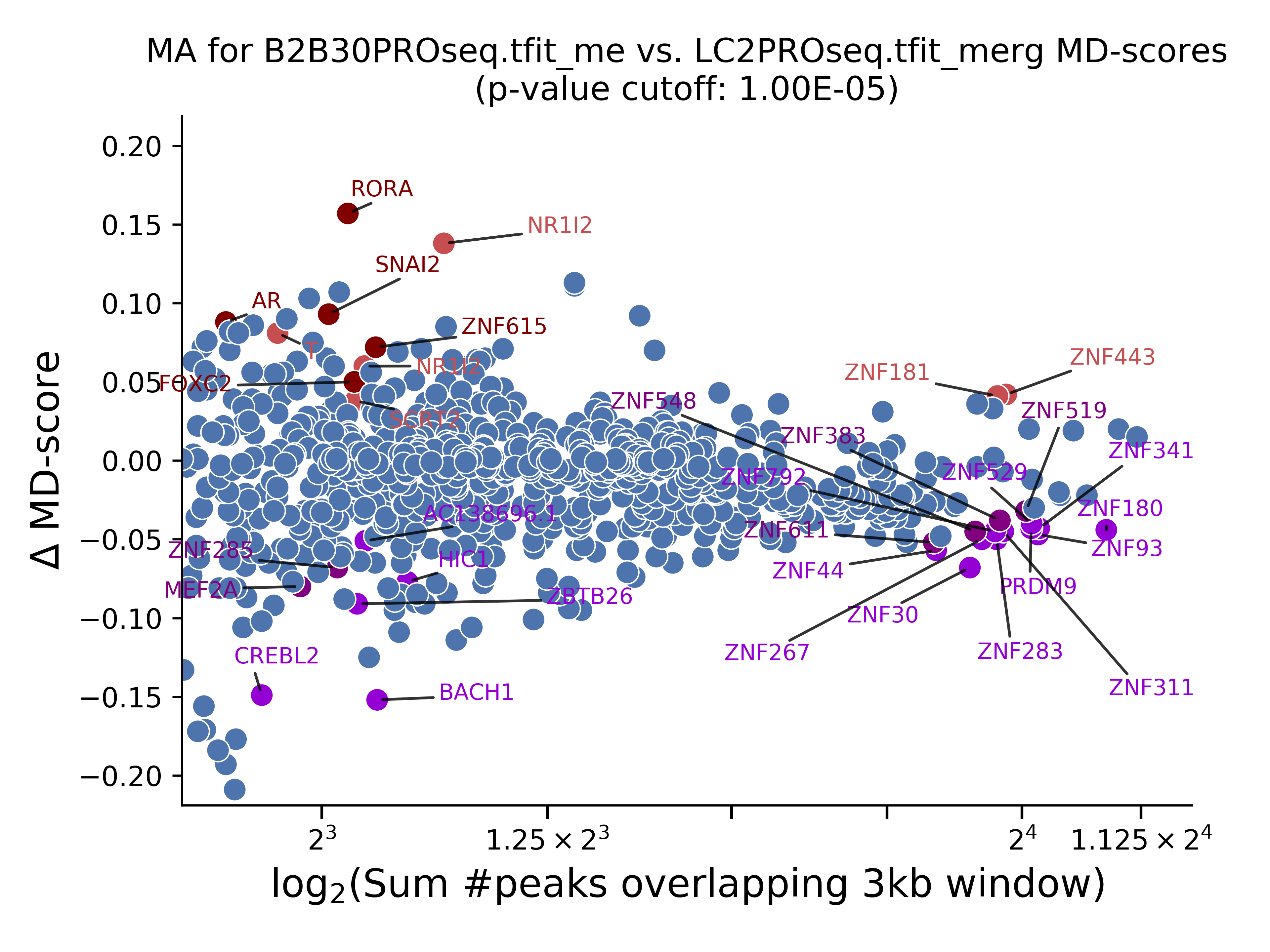

Supplement: Supplemental Data Set 2 [file jciinsight-6-144294-s077.zip › best_curated_Human_TFs_p1e-6_grch38/B2B_vs_LC2/MA_B2B30PROseq.tfit_merged_to_LC2PROseq.tfit_merged_md_score.png]

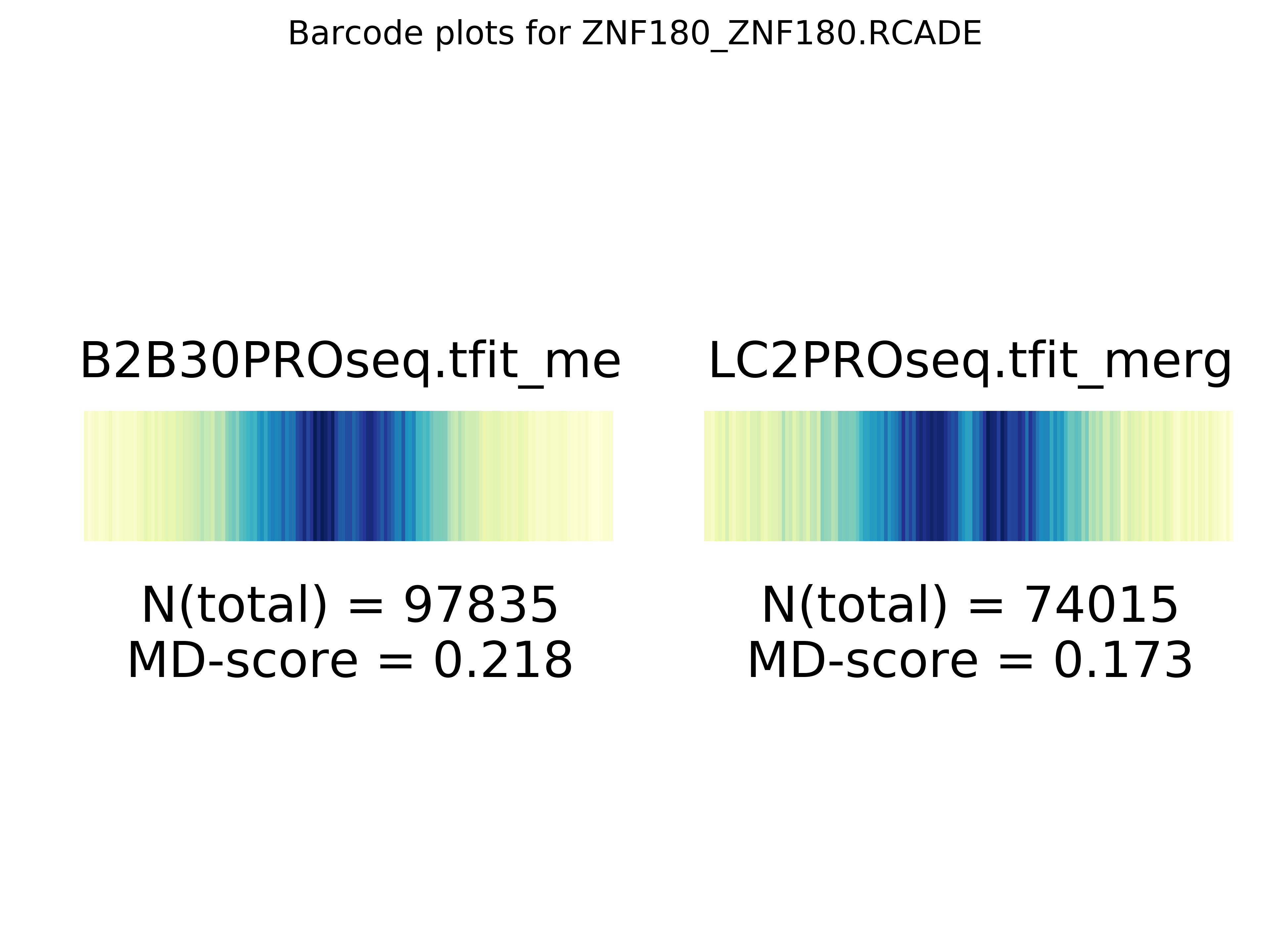

Supplement: Supplemental Data Set 2 [file jciinsight-6-144294-s077.zip › best_curated_Human_TFs_p1e-6_grch38/B2B_vs_LC2/ZNF180_ZNF180.RCADE_barcode_B2B30PROseq.tfit_merged_vs_LC2PROseq.tfit_merged.png]

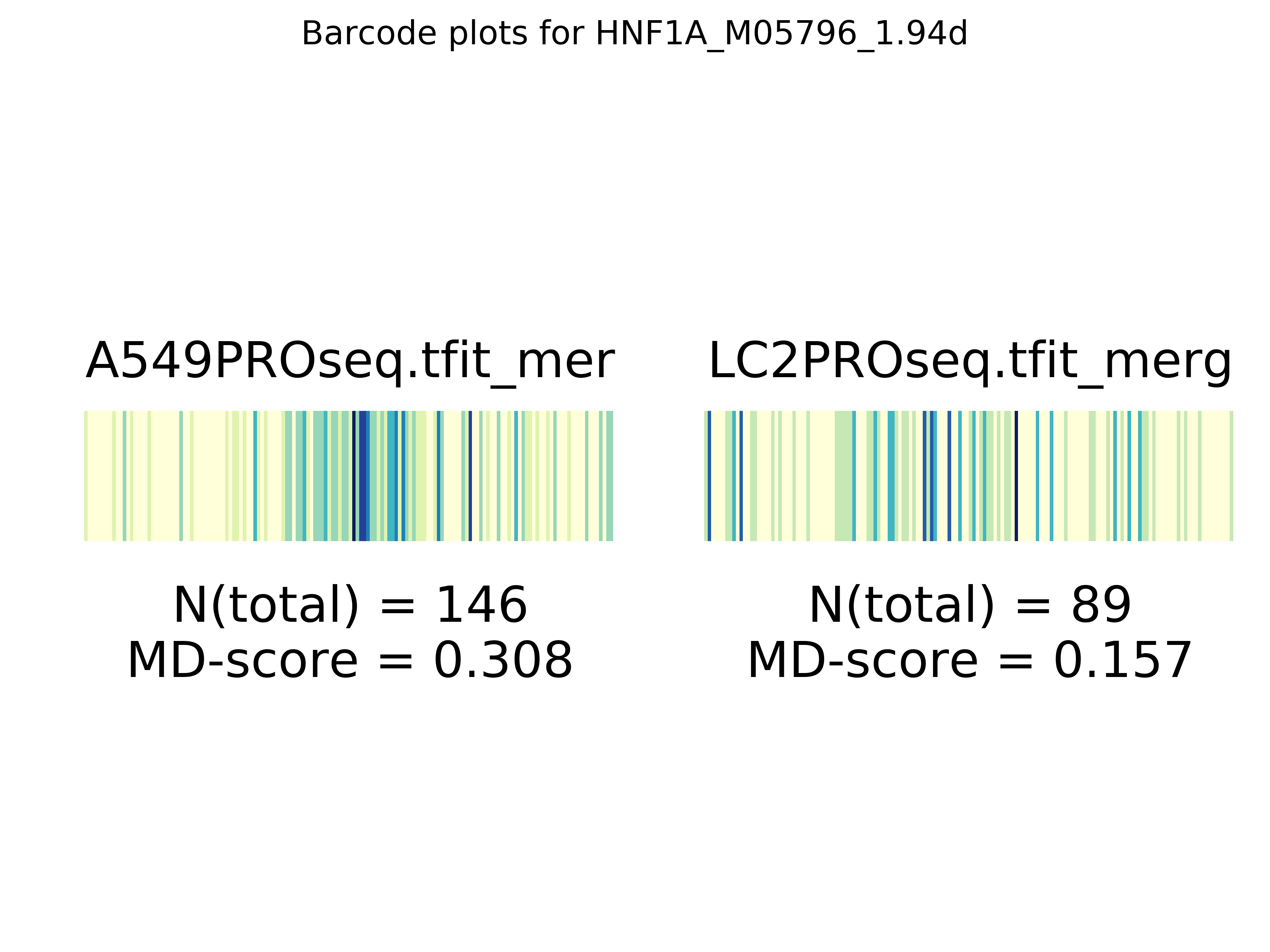

Supplement: Supplemental Data Set 2 [file jciinsight-6-144294-s077.zip › best_curated_Human_TFs_p1e-6_grch38/A549_vs_LC2/HNF1A_M05796_1.94d_barcode_A549PROseq.tfit_merged_vs_LC2PROseq.tfit_merged.png]

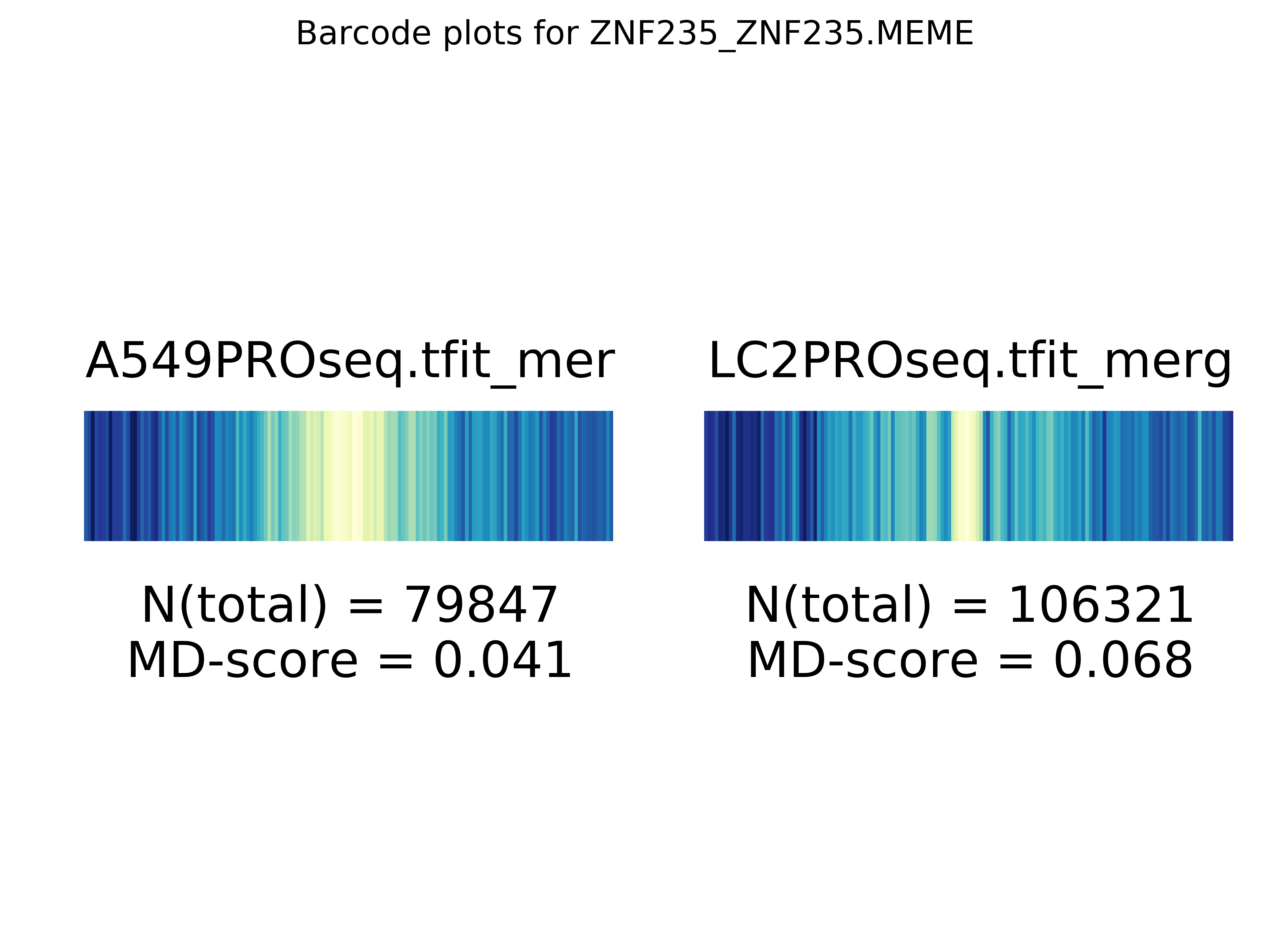

Supplement: Supplemental Data Set 2 [file jciinsight-6-144294-s077.zip › best_curated_Human_TFs_p1e-6_grch38/A549_vs_LC2/ZNF235_ZNF235.MEME_barcode_A549PROseq.tfit_merged_vs_LC2PROseq.tfit_merged.png]

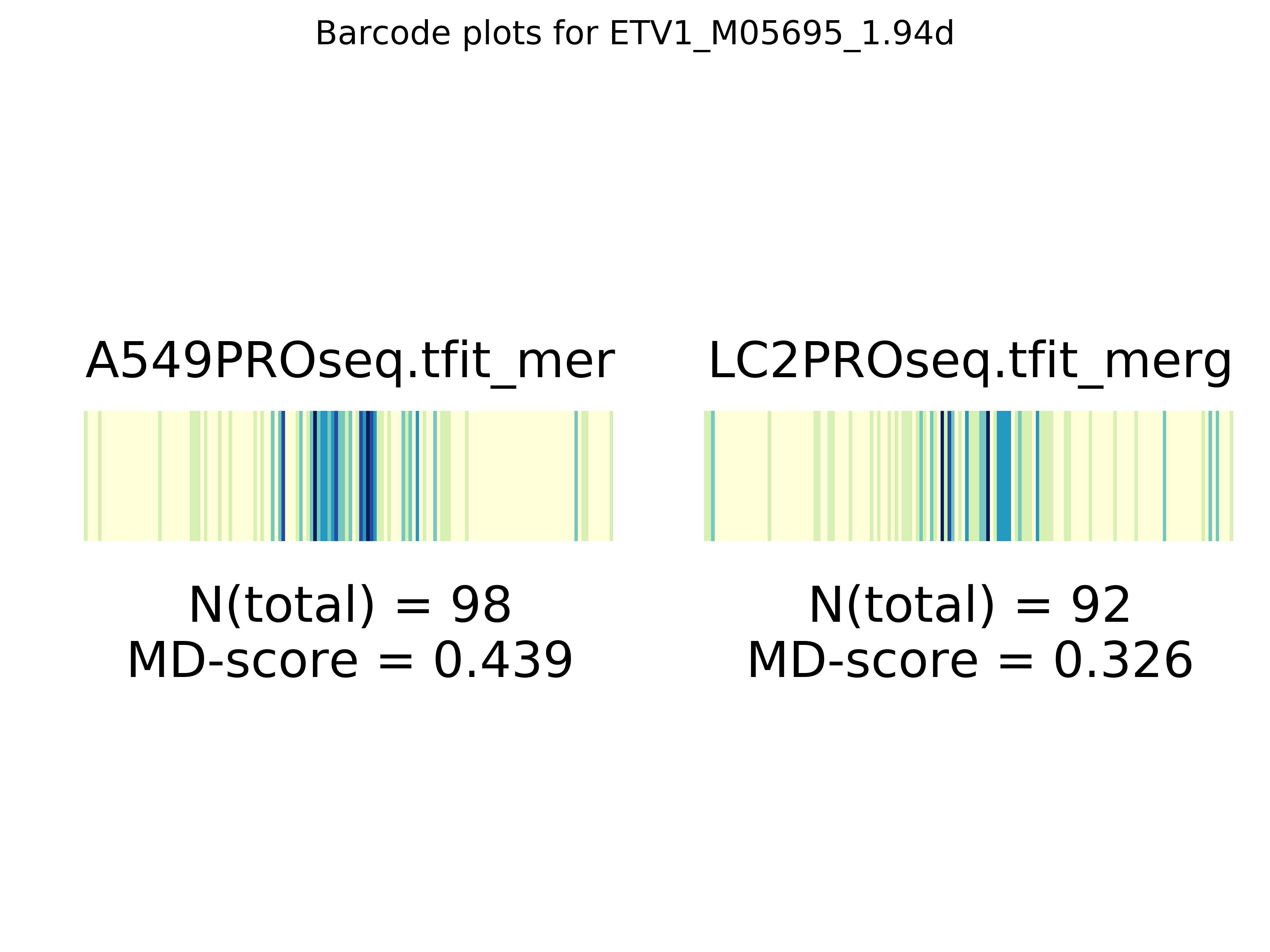

Supplement: Supplemental Data Set 2 [file jciinsight-6-144294-s077.zip › best_curated_Human_TFs_p1e-6_grch38/A549_vs_LC2/ETV1_M05695_1.94d_barcode_A549PROseq.tfit_merged_vs_LC2PROseq.tfit_merged.png]

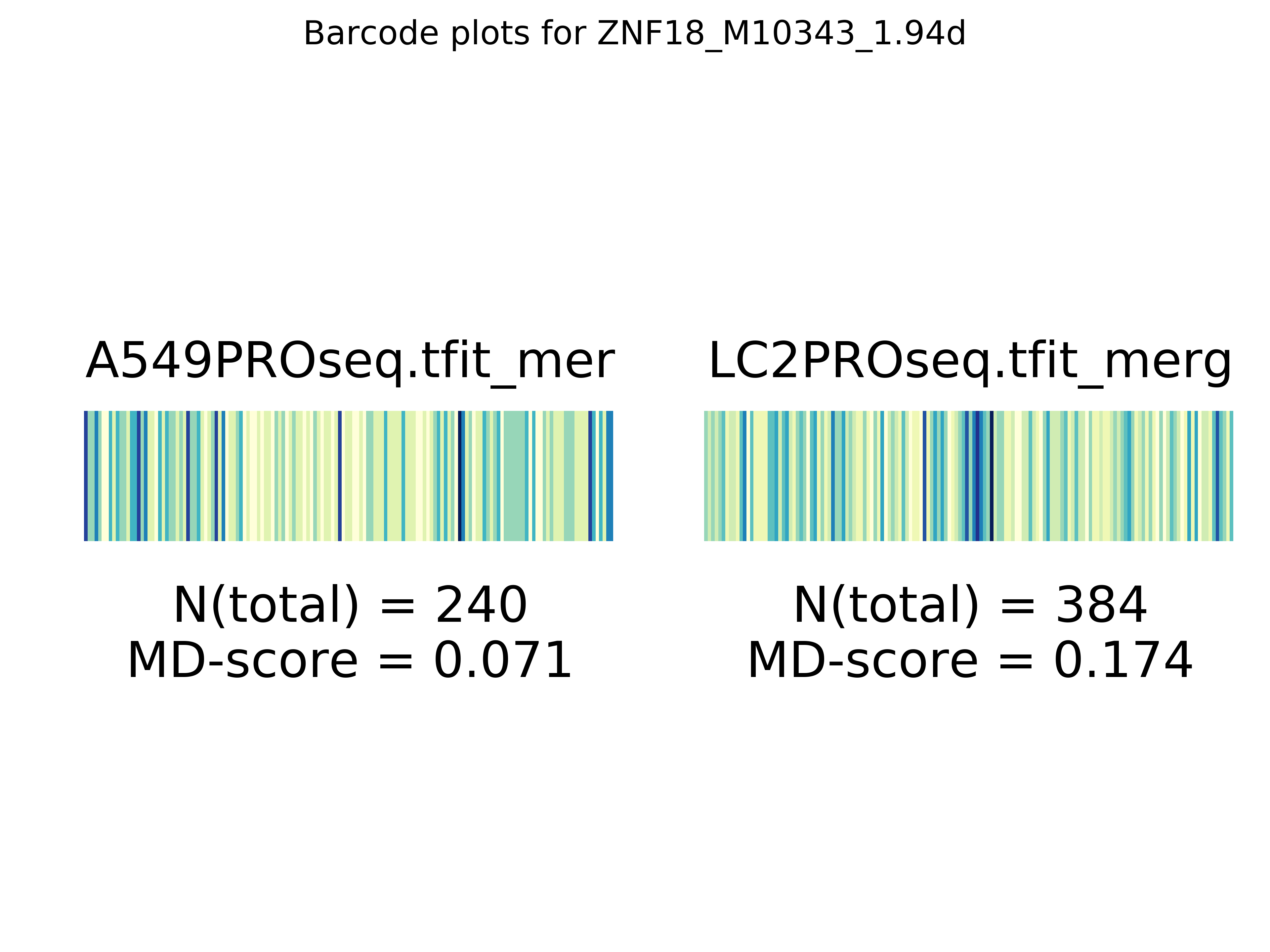

Supplement: Supplemental Data Set 2 [file jciinsight-6-144294-s077.zip › best_curated_Human_TFs_p1e-6_grch38/A549_vs_LC2/ZNF18_M10343_1.94d_barcode_A549PROseq.tfit_merged_vs_LC2PROseq.tfit_merged.png]

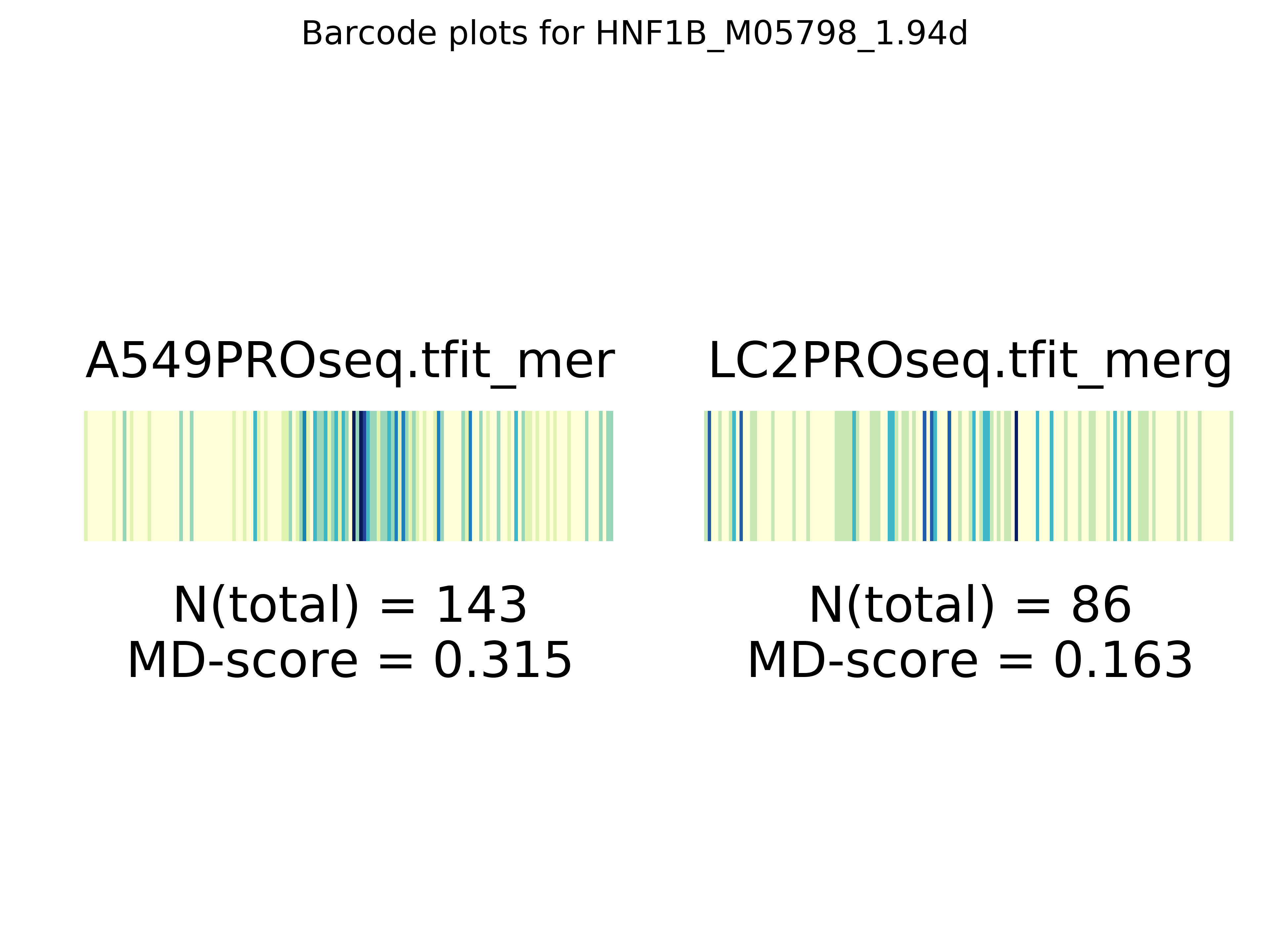

Supplement: Supplemental Data Set 2 [file jciinsight-6-144294-s077.zip › best_curated_Human_TFs_p1e-6_grch38/A549_vs_LC2/HNF1B_M05798_1.94d_barcode_A549PROseq.tfit_merged_vs_LC2PROseq.tfit_merged.png]

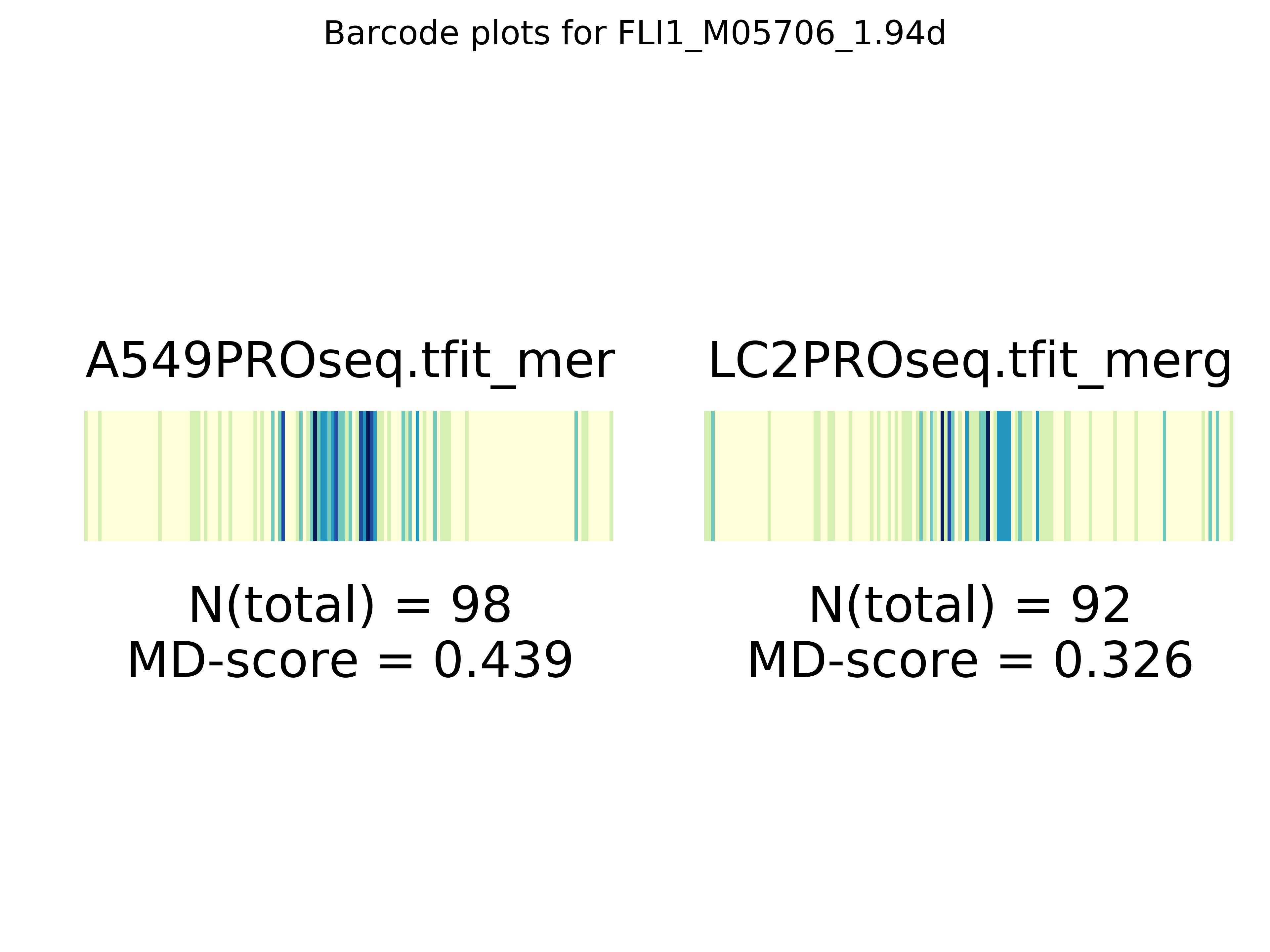

Supplement: Supplemental Data Set 2 [file jciinsight-6-144294-s077.zip › best_curated_Human_TFs_p1e-6_grch38/A549_vs_LC2/FLI1_M05706_1.94d_barcode_A549PROseq.tfit_merged_vs_LC2PROseq.tfit_merged.png]

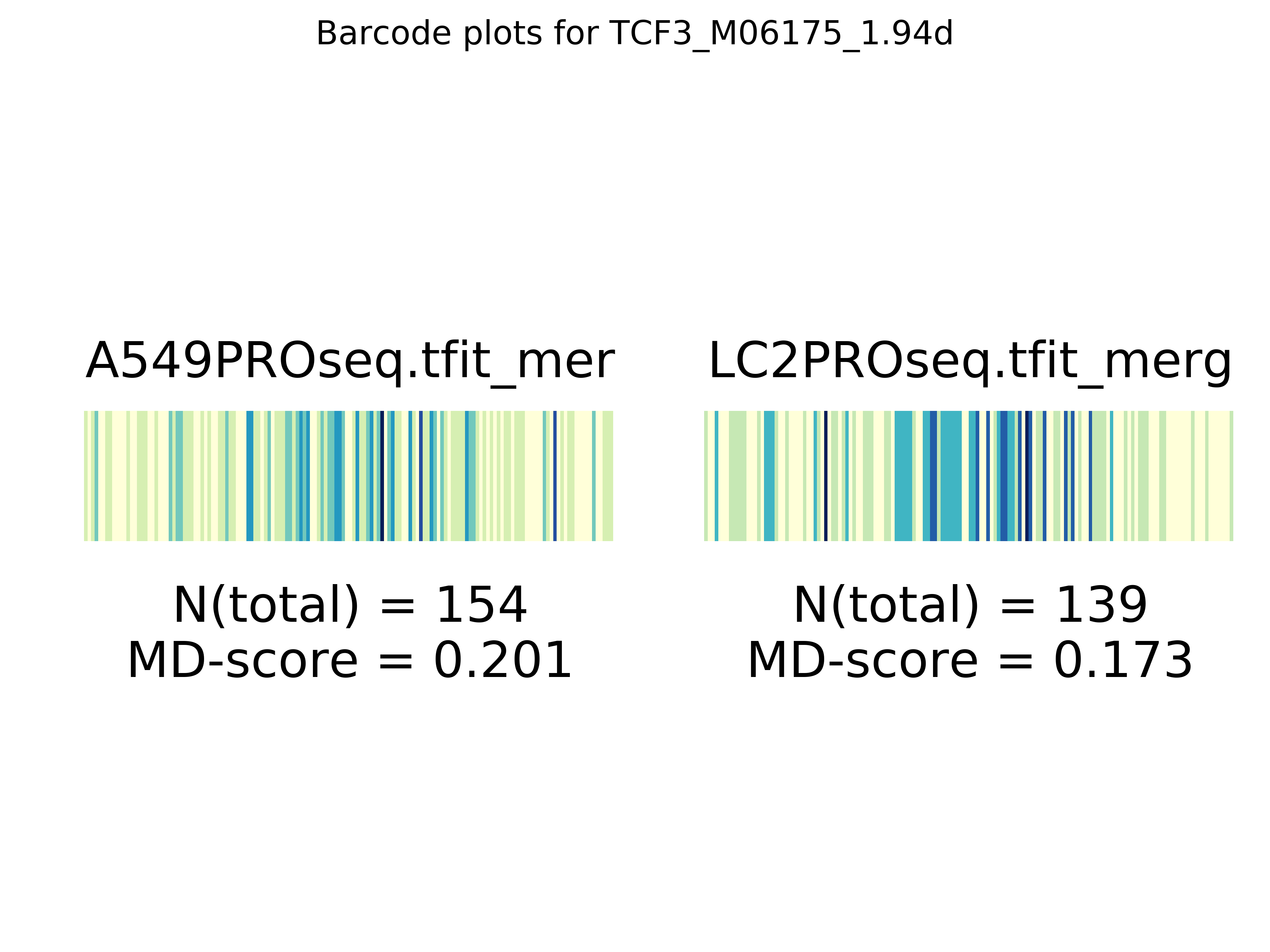

Supplement: Supplemental Data Set 2 [file jciinsight-6-144294-s077.zip › best_curated_Human_TFs_p1e-6_grch38/A549_vs_LC2/TCF3_M06175_1.94d_barcode_A549PROseq.tfit_merged_vs_LC2PROseq.tfit_merged.png]

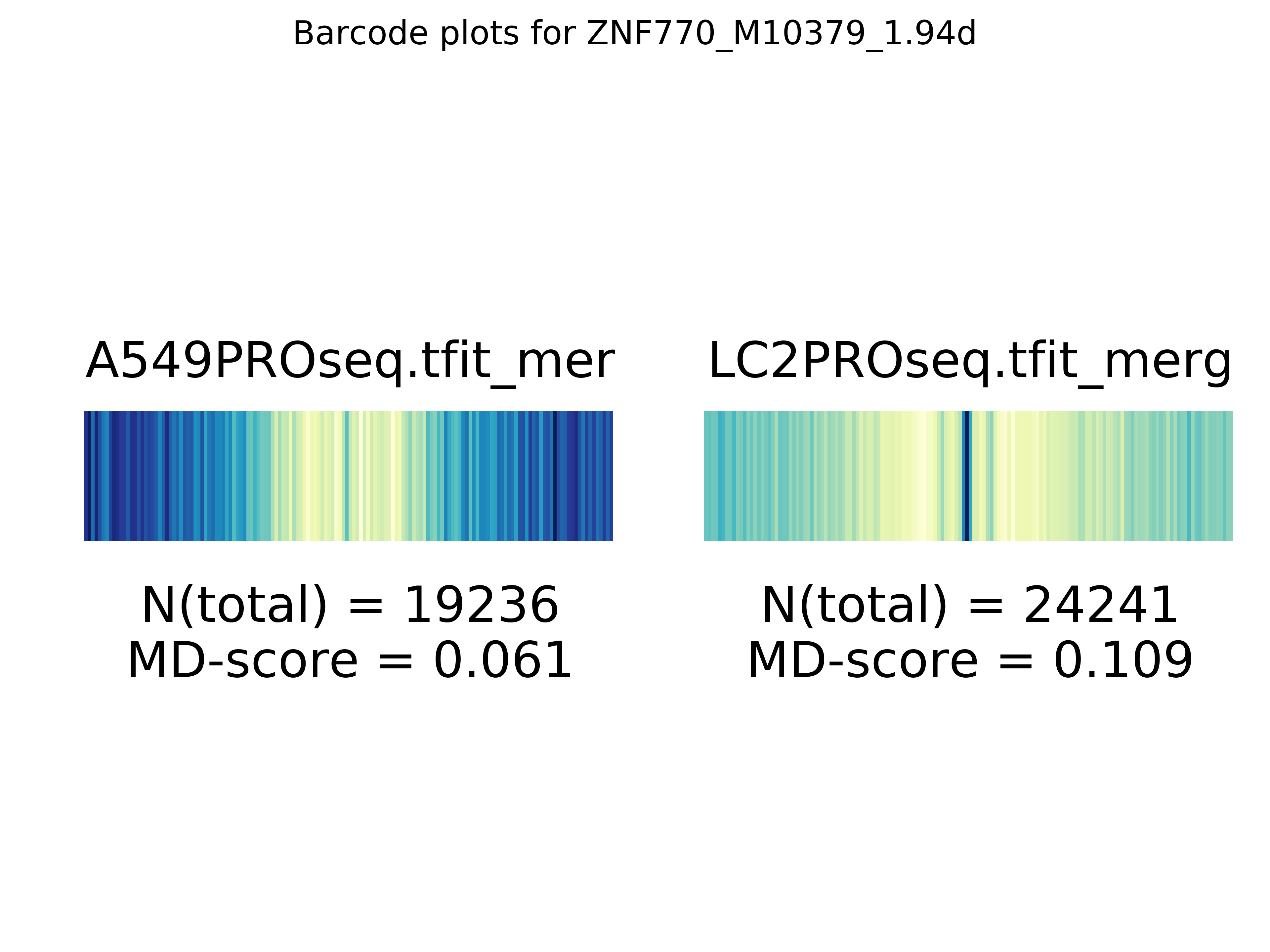

Supplement: Supplemental Data Set 2 [file jciinsight-6-144294-s077.zip › best_curated_Human_TFs_p1e-6_grch38/A549_vs_LC2/ZNF770_M10379_1.94d_barcode_A549PROseq.tfit_merged_vs_LC2PROseq.tfit_merged.png]

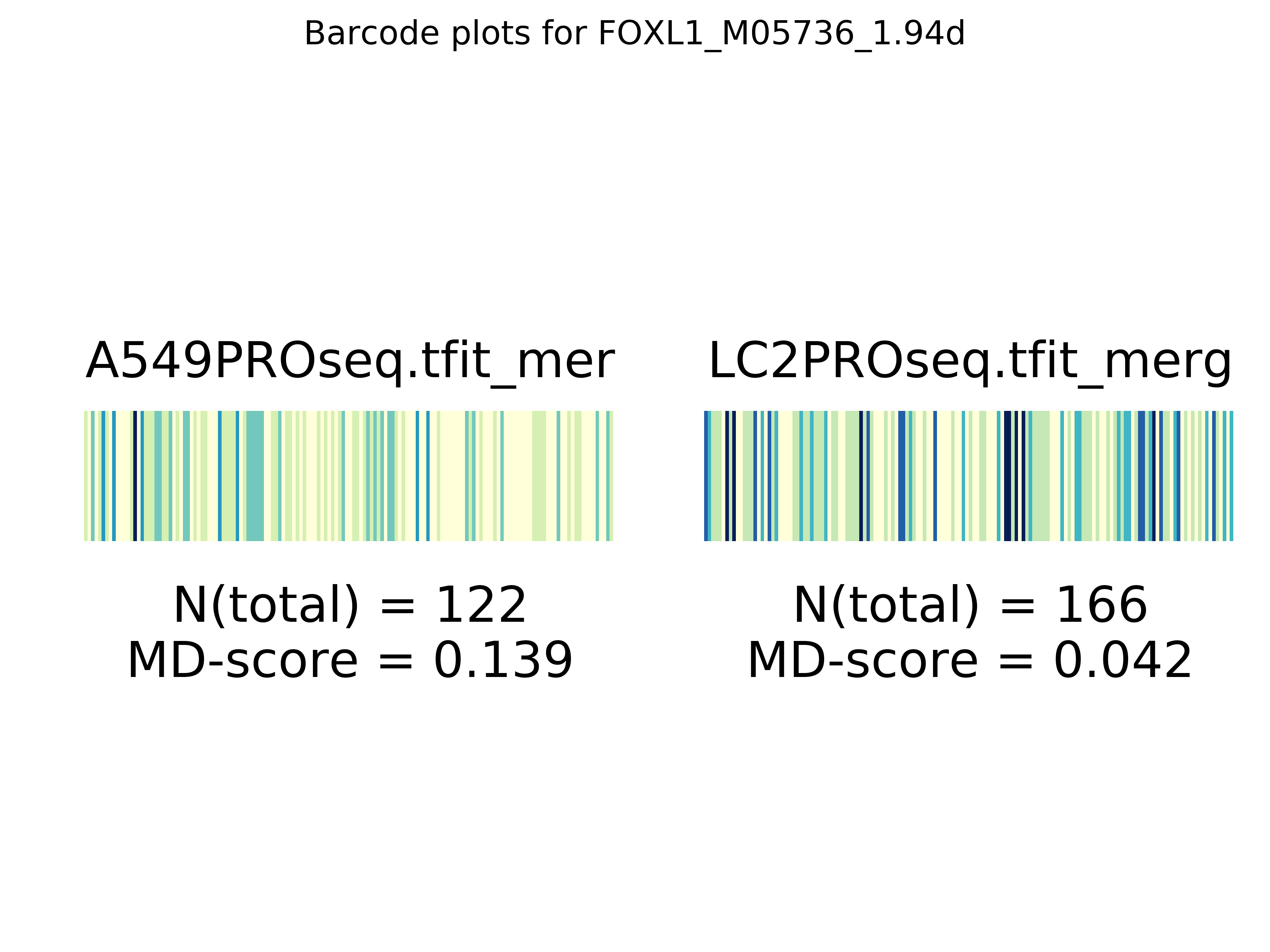

Supplement: Supplemental Data Set 2 [file jciinsight-6-144294-s077.zip › best_curated_Human_TFs_p1e-6_grch38/A549_vs_LC2/FOXL1_M05736_1.94d_barcode_A549PROseq.tfit_merged_vs_LC2PROseq.tfit_merged.png]

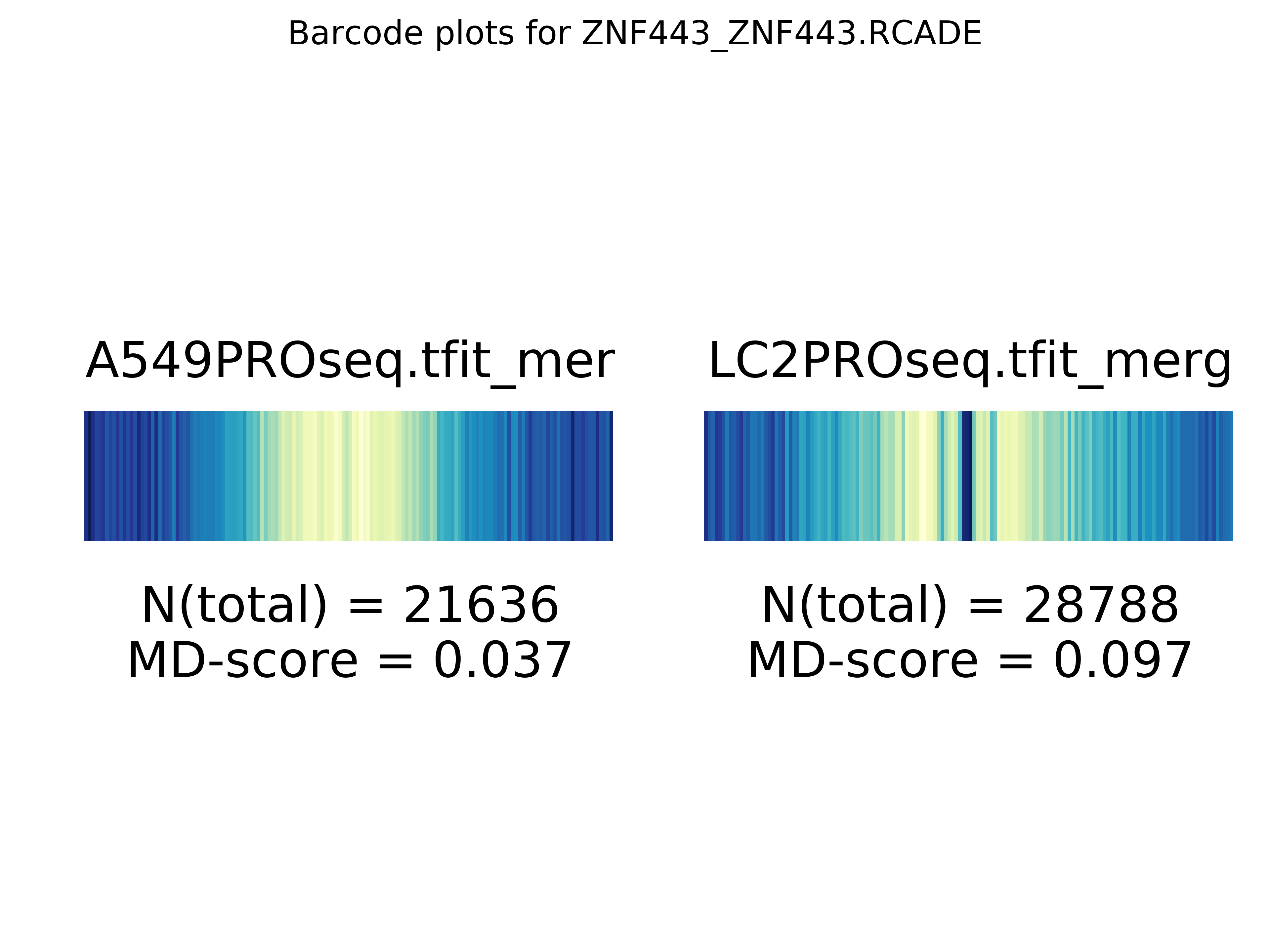

Supplement: Supplemental Data Set 2 [file jciinsight-6-144294-s077.zip › best_curated_Human_TFs_p1e-6_grch38/A549_vs_LC2/ZNF443_ZNF443.RCADE_barcode_A549PROseq.tfit_merged_vs_LC2PROseq.tfit_merged.png]

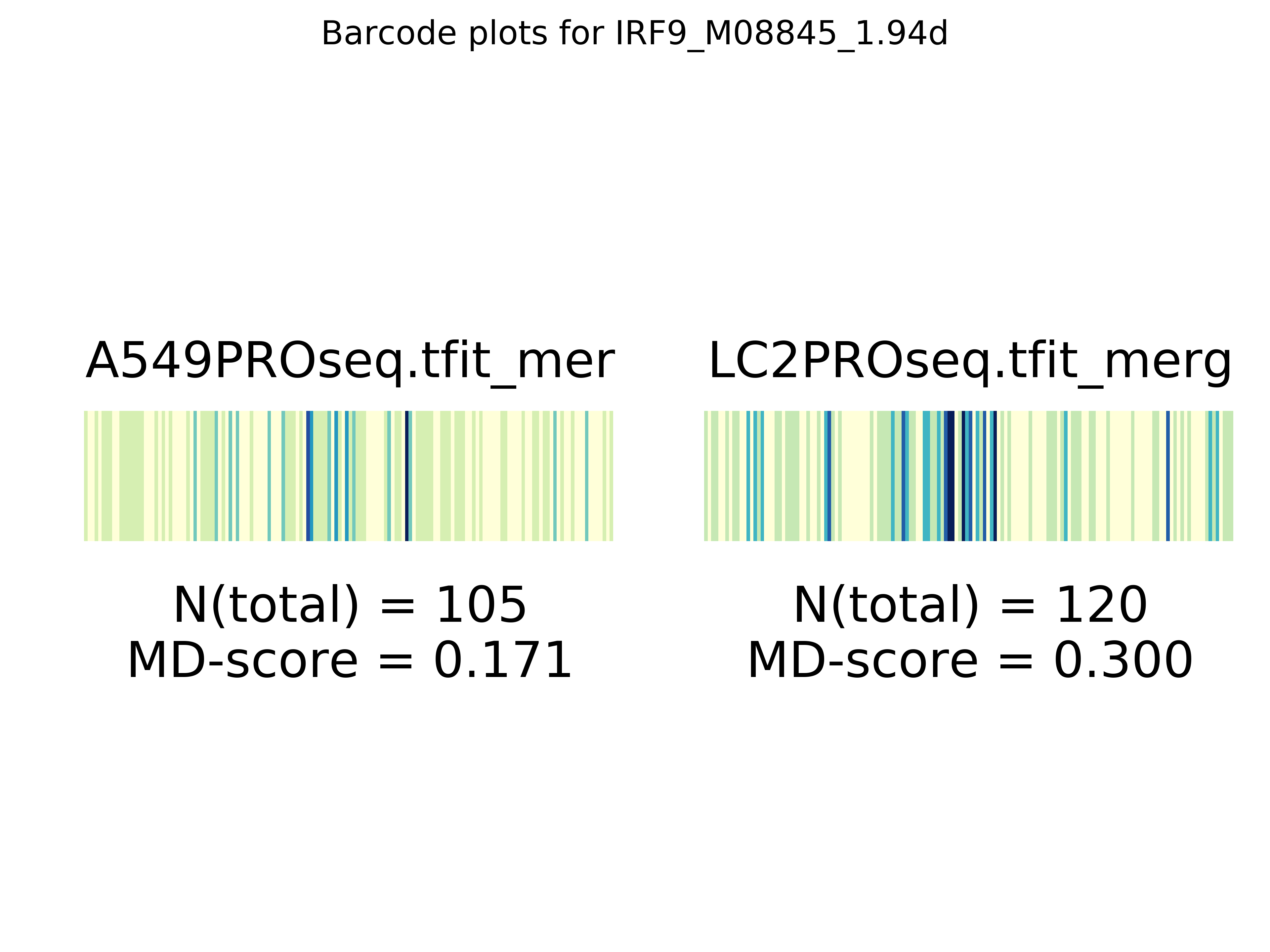

Supplement: Supplemental Data Set 2 [file jciinsight-6-144294-s077.zip › best_curated_Human_TFs_p1e-6_grch38/A549_vs_LC2/IRF9_M08845_1.94d_barcode_A549PROseq.tfit_merged_vs_LC2PROseq.tfit_merged.png]

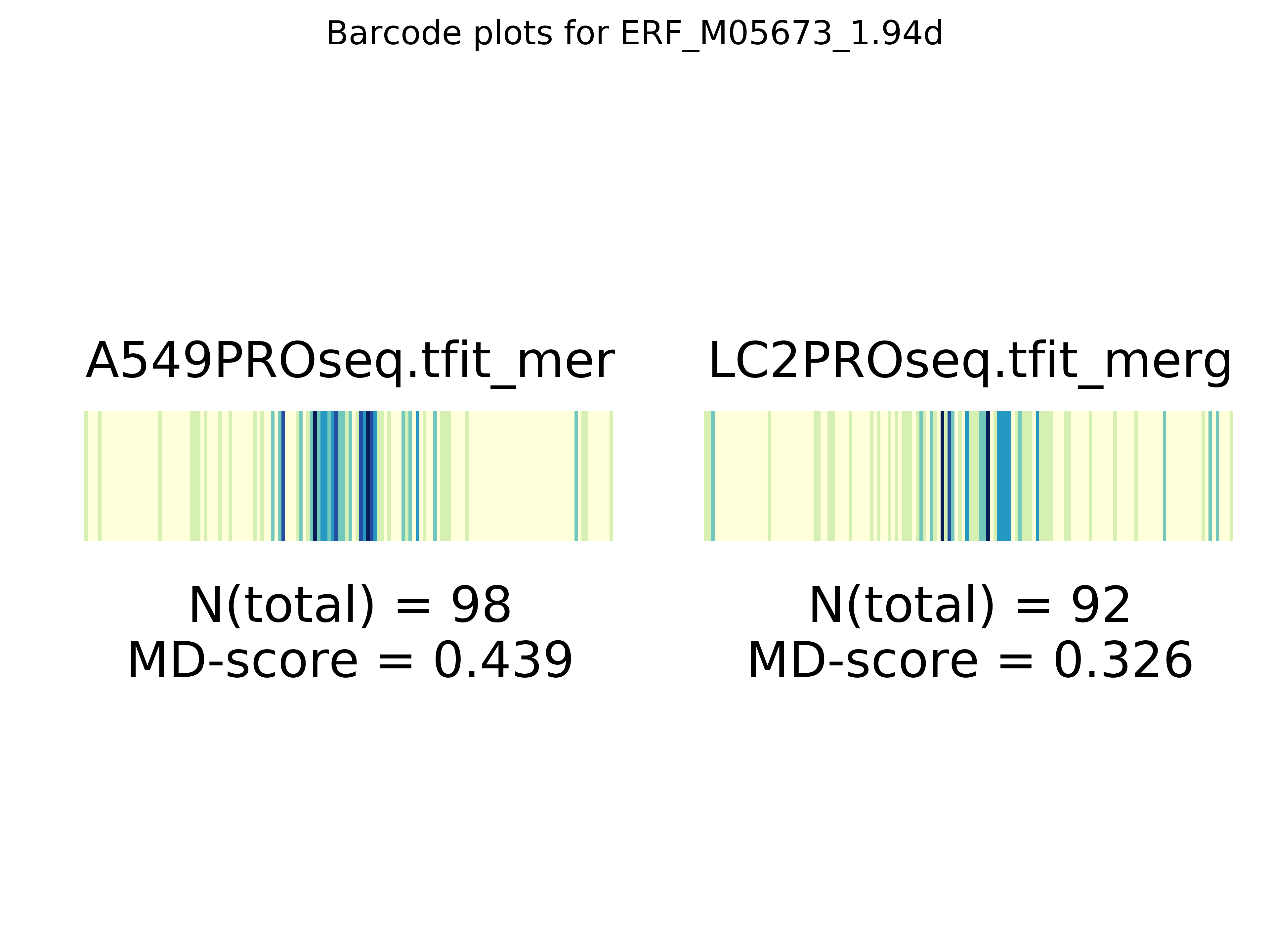

Supplement: Supplemental Data Set 2 [file jciinsight-6-144294-s077.zip › best_curated_Human_TFs_p1e-6_grch38/A549_vs_LC2/ERF_M05673_1.94d_barcode_A549PROseq.tfit_merged_vs_LC2PROseq.tfit_merged.png]

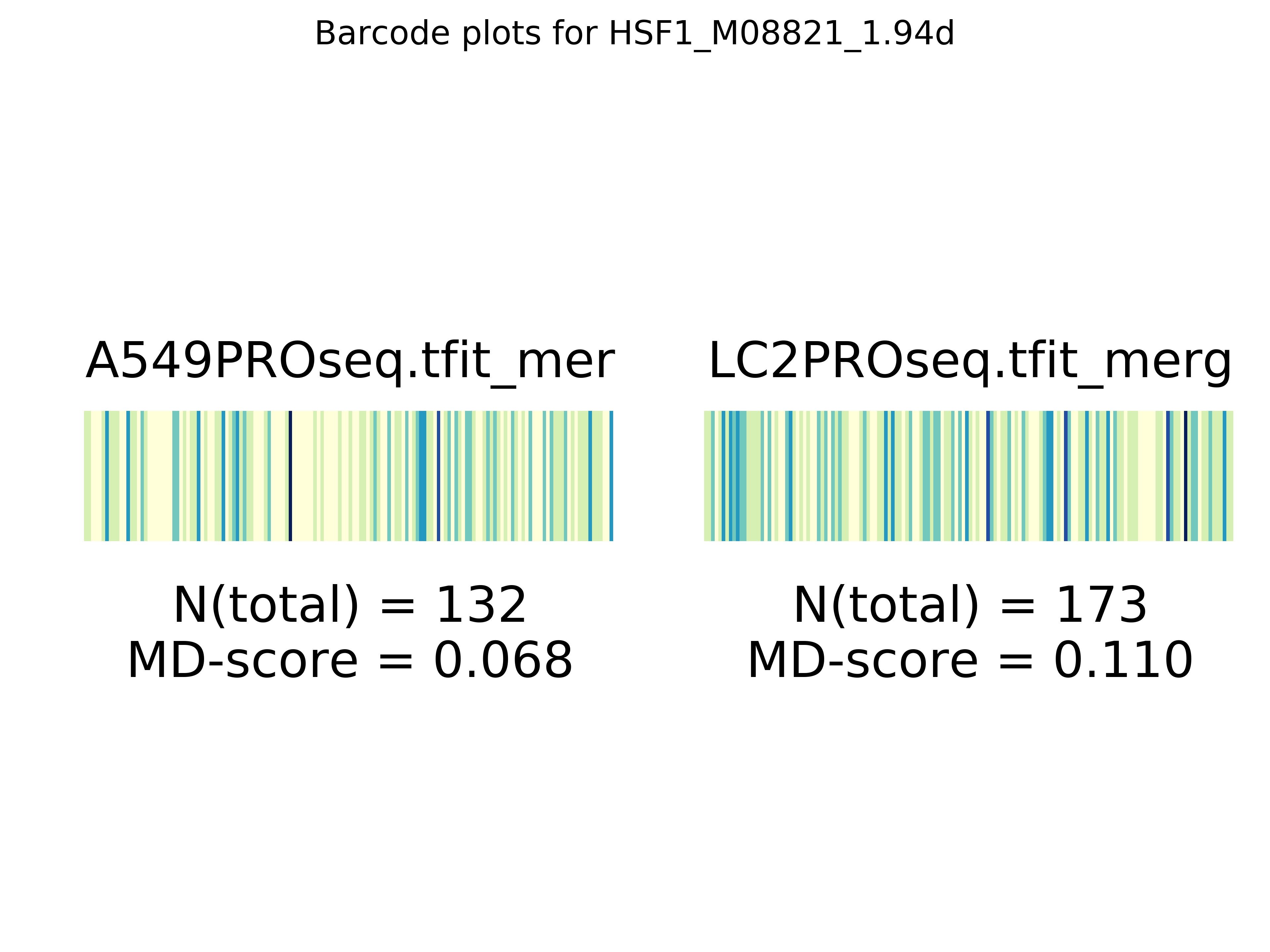

Supplement: Supplemental Data Set 2 [file jciinsight-6-144294-s077.zip › best_curated_Human_TFs_p1e-6_grch38/A549_vs_LC2/HSF1_M08821_1.94d_barcode_A549PROseq.tfit_merged_vs_LC2PROseq.tfit_merged.png]

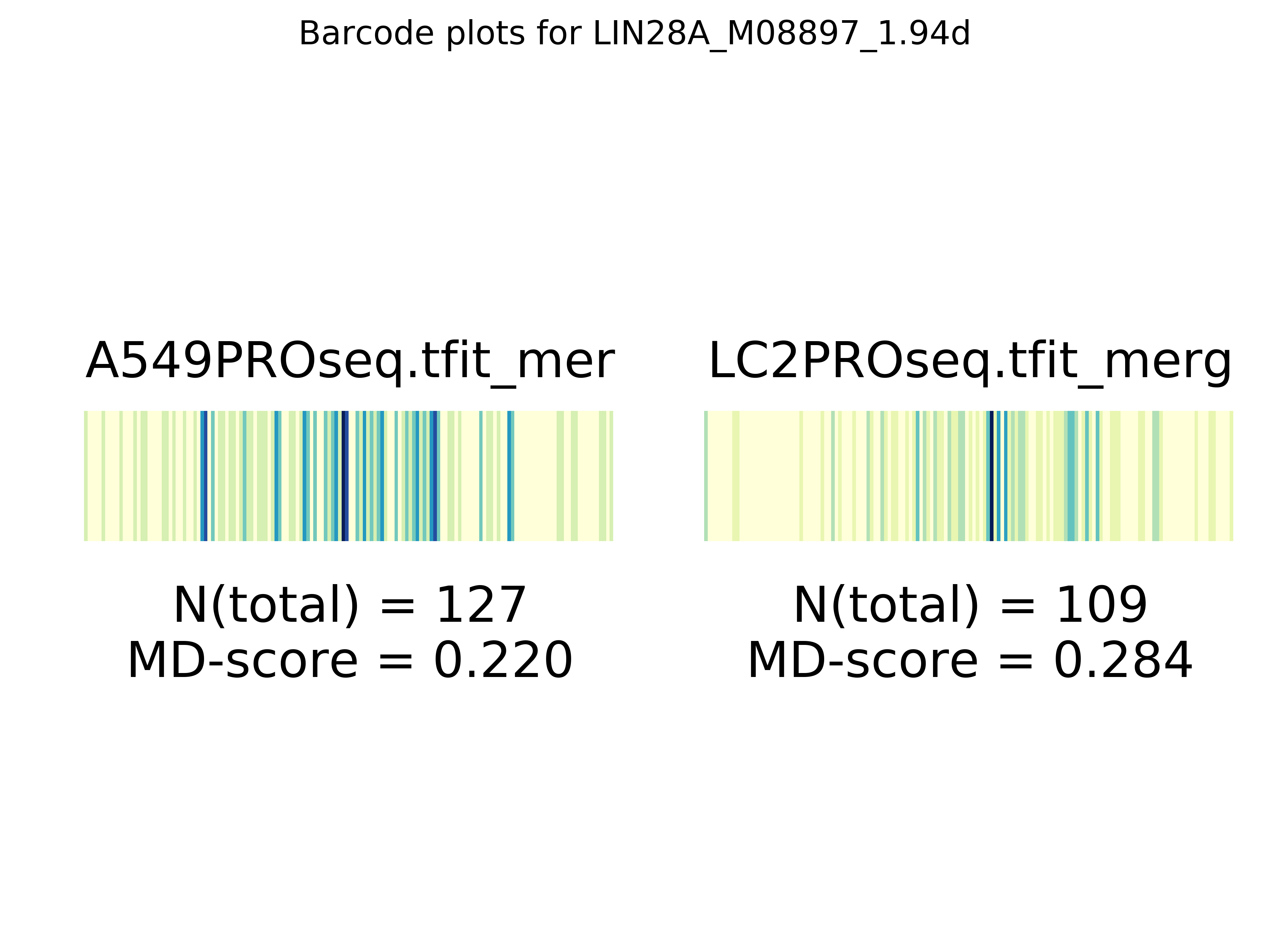

Supplement: Supplemental Data Set 2 [file jciinsight-6-144294-s077.zip › best_curated_Human_TFs_p1e-6_grch38/A549_vs_LC2/LIN28A_M08897_1.94d_barcode_A549PROseq.tfit_merged_vs_LC2PROseq.tfit_merged.png]

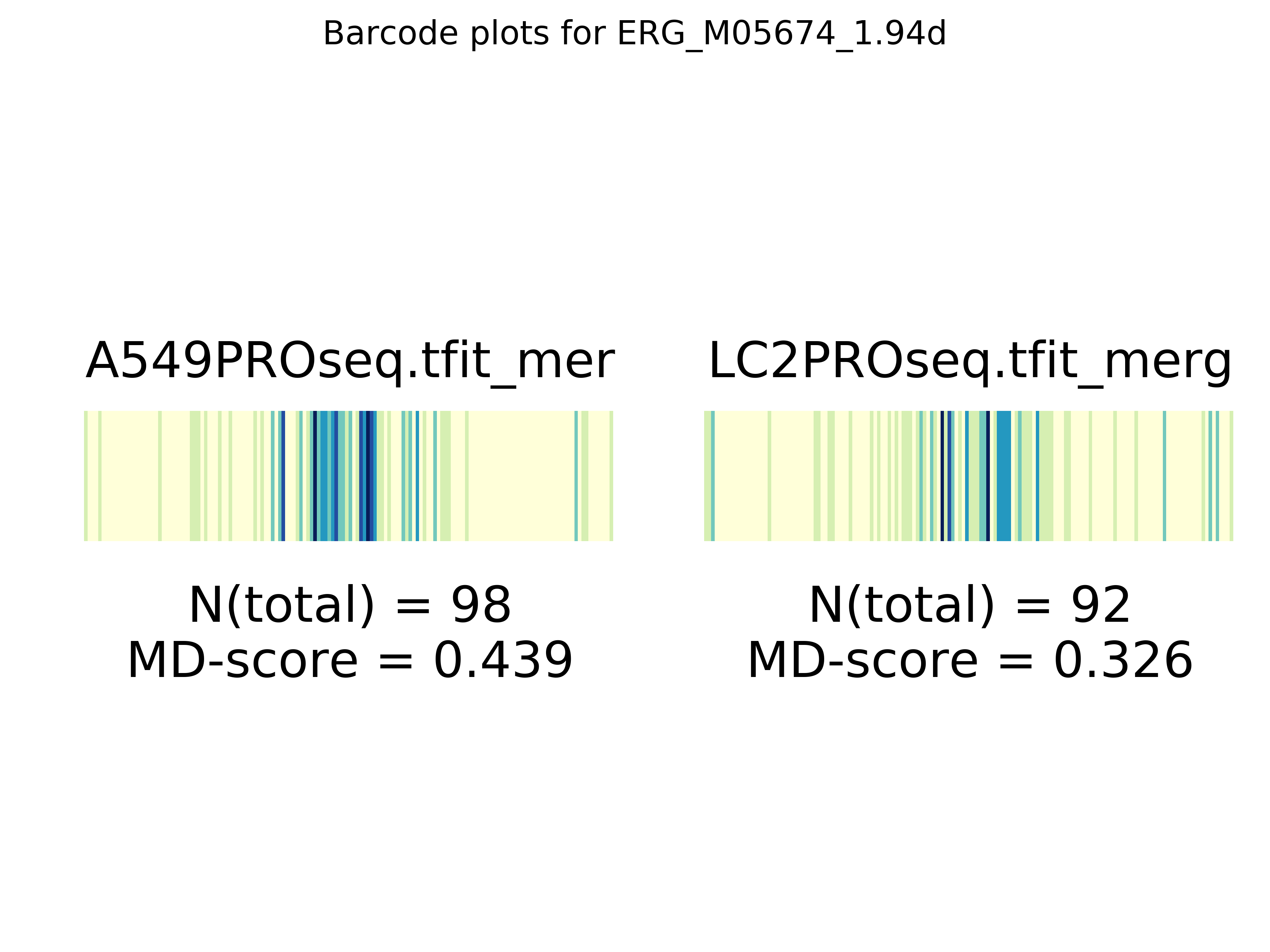

Supplement: Supplemental Data Set 2 [file jciinsight-6-144294-s077.zip › best_curated_Human_TFs_p1e-6_grch38/A549_vs_LC2/ERG_M05674_1.94d_barcode_A549PROseq.tfit_merged_vs_LC2PROseq.tfit_merged.png]

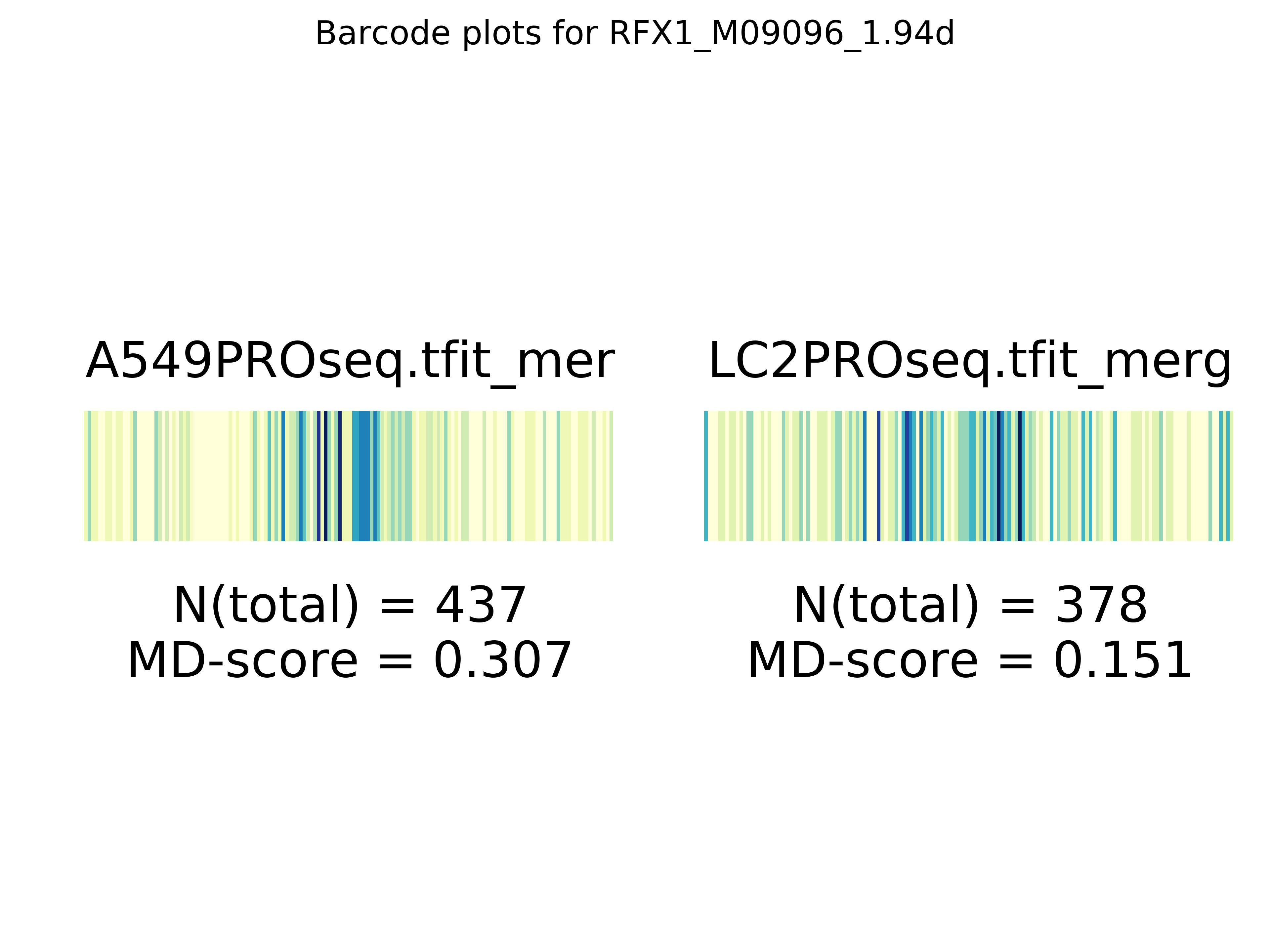

Supplement: Supplemental Data Set 2 [file jciinsight-6-144294-s077.zip › best_curated_Human_TFs_p1e-6_grch38/A549_vs_LC2/RFX1_M09096_1.94d_barcode_A549PROseq.tfit_merged_vs_LC2PROseq.tfit_merged.png]

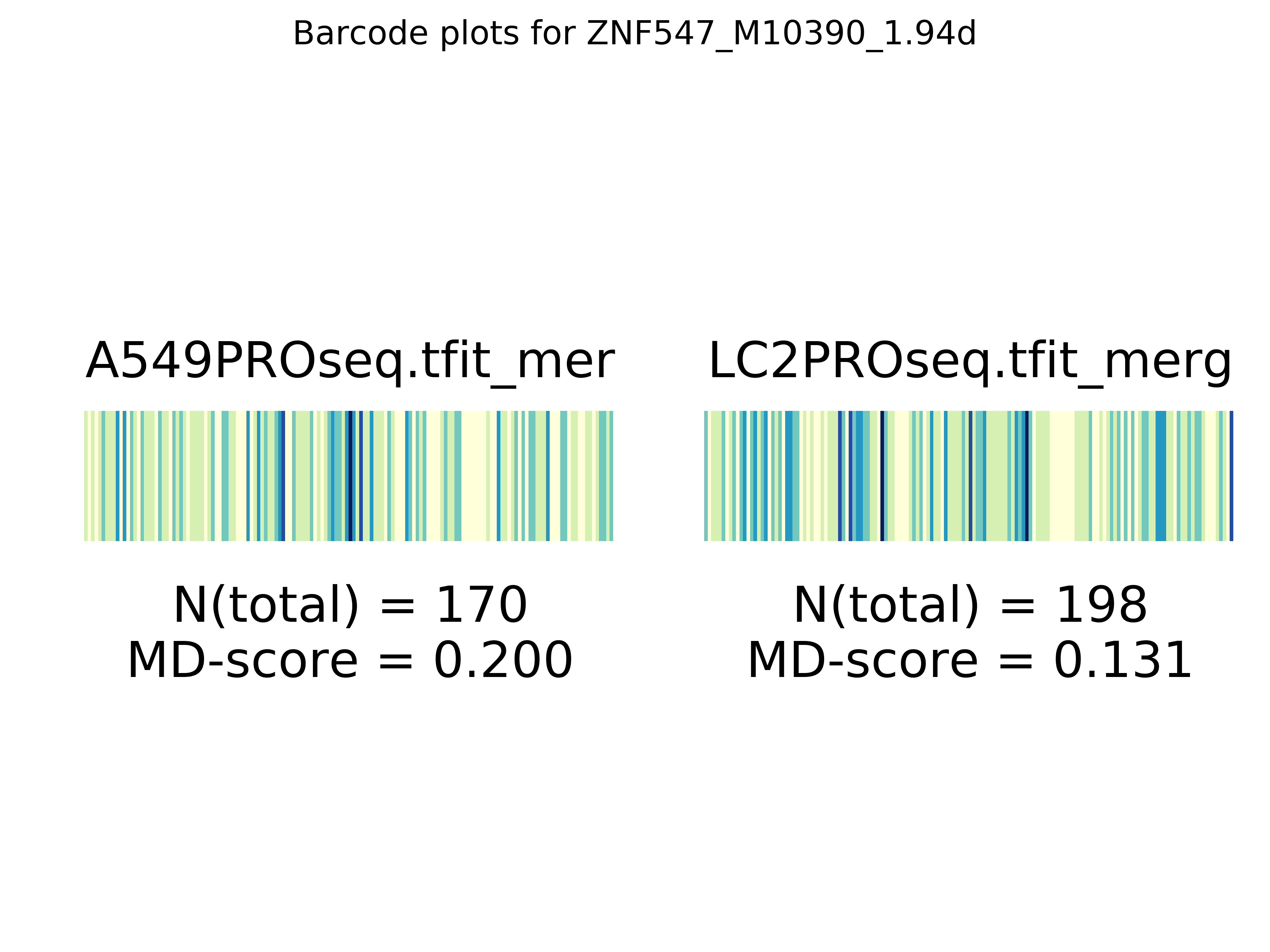

Supplement: Supplemental Data Set 2 [file jciinsight-6-144294-s077.zip › best_curated_Human_TFs_p1e-6_grch38/A549_vs_LC2/ZNF547_M10390_1.94d_barcode_A549PROseq.tfit_merged_vs_LC2PROseq.tfit_merged.png]

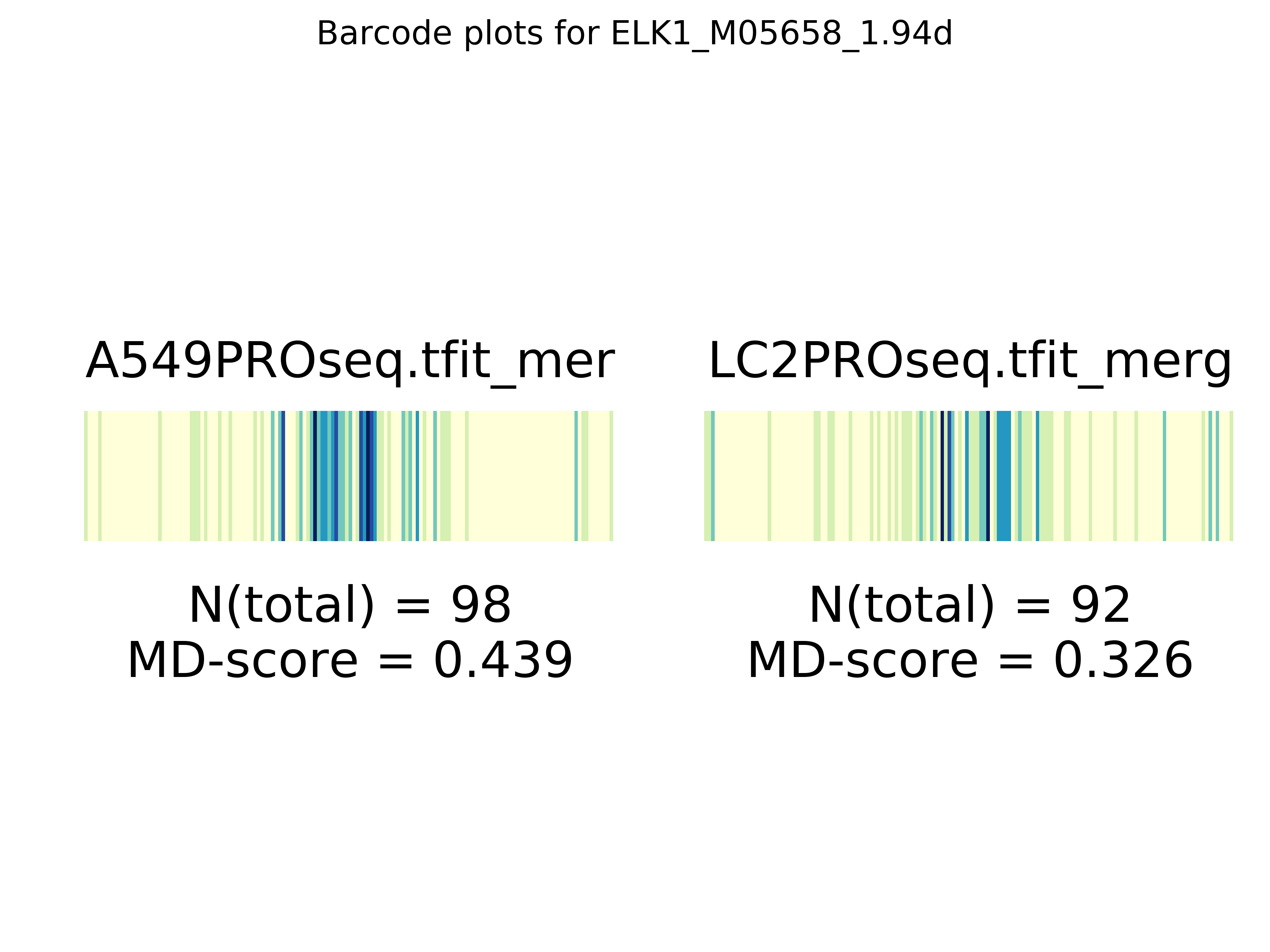

Supplement: Supplemental Data Set 2 [file jciinsight-6-144294-s077.zip › best_curated_Human_TFs_p1e-6_grch38/A549_vs_LC2/ELK1_M05658_1.94d_barcode_A549PROseq.tfit_merged_vs_LC2PROseq.tfit_merged.png]

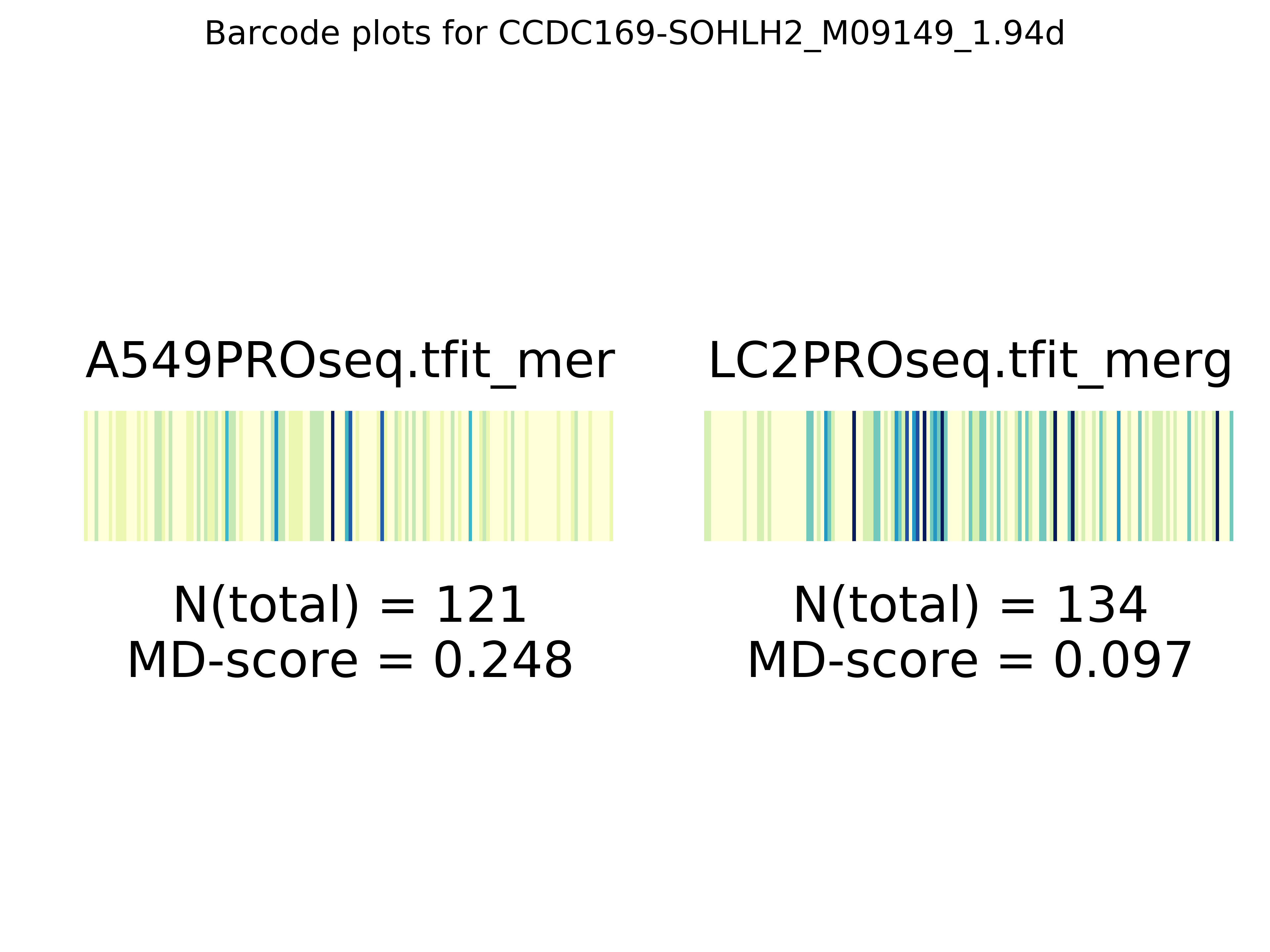

Supplement: Supplemental Data Set 2 [file jciinsight-6-144294-s077.zip › best_curated_Human_TFs_p1e-6_grch38/A549_vs_LC2/CCDC169-SOHLH2_M09149_1.94d_barcode_A549PROseq.tfit_merged_vs_LC2PROseq.tfit_merged.png]

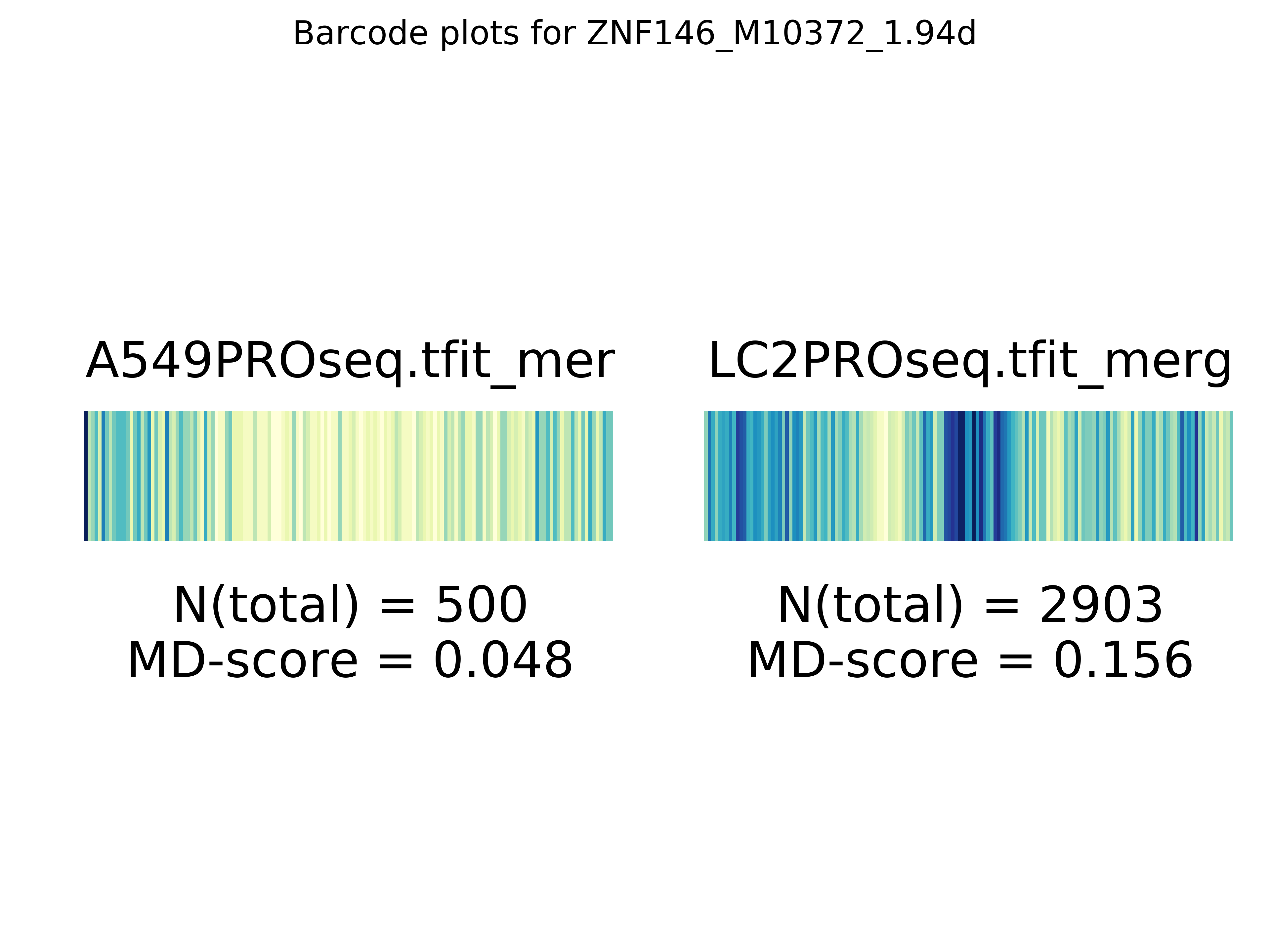

Supplement: Supplemental Data Set 2 [file jciinsight-6-144294-s077.zip › best_curated_Human_TFs_p1e-6_grch38/A549_vs_LC2/ZNF146_M10372_1.94d_barcode_A549PROseq.tfit_merged_vs_LC2PROseq.tfit_merged.png]
