# Supplementary figures and images for: The MUC5B-associated variant rs35705950 resides within an enhancer subject to lineage- and disease-dependent epigenetic remodeling (part 1 of 3)
Source: JCI Insight. 2021 Jan 25;6(2):e144294. doi: 10.1172/jci.insight.144294 (PMC7934873; doi:10.1172/jci.insight.144294)

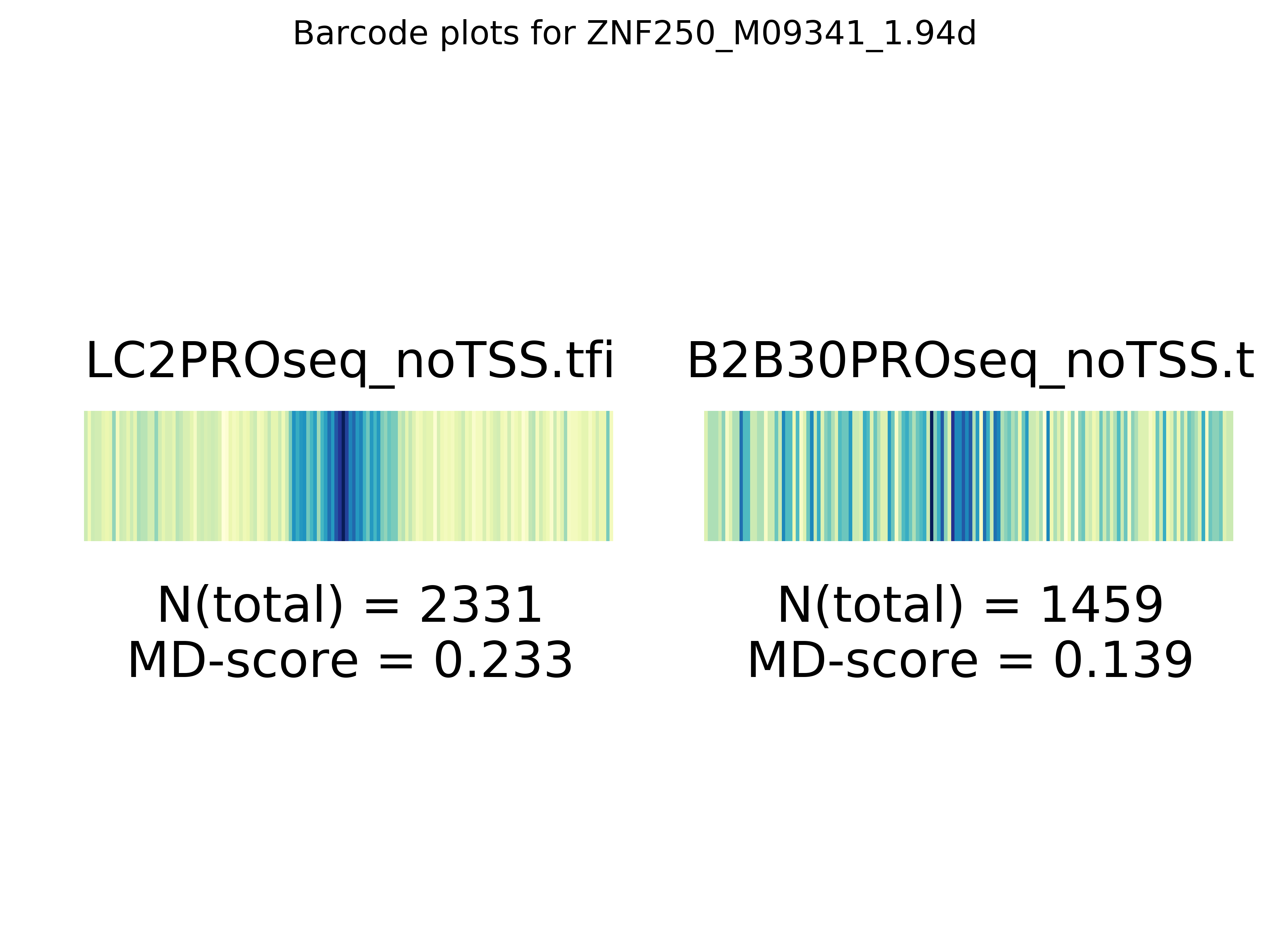

Supplement: Supplemental Data Set 1 [file jciinsight-6-144294-s076.zip › noTSS/best_curated_Human_TFs_p1e-5_grch38/LC2_vs_B2B/ZNF250_M09341_1.94d_barcode_LC2PROseq_noTSS.tfit_merged_vs_B2B30PROseq_noTSS.tfit_merged.png]

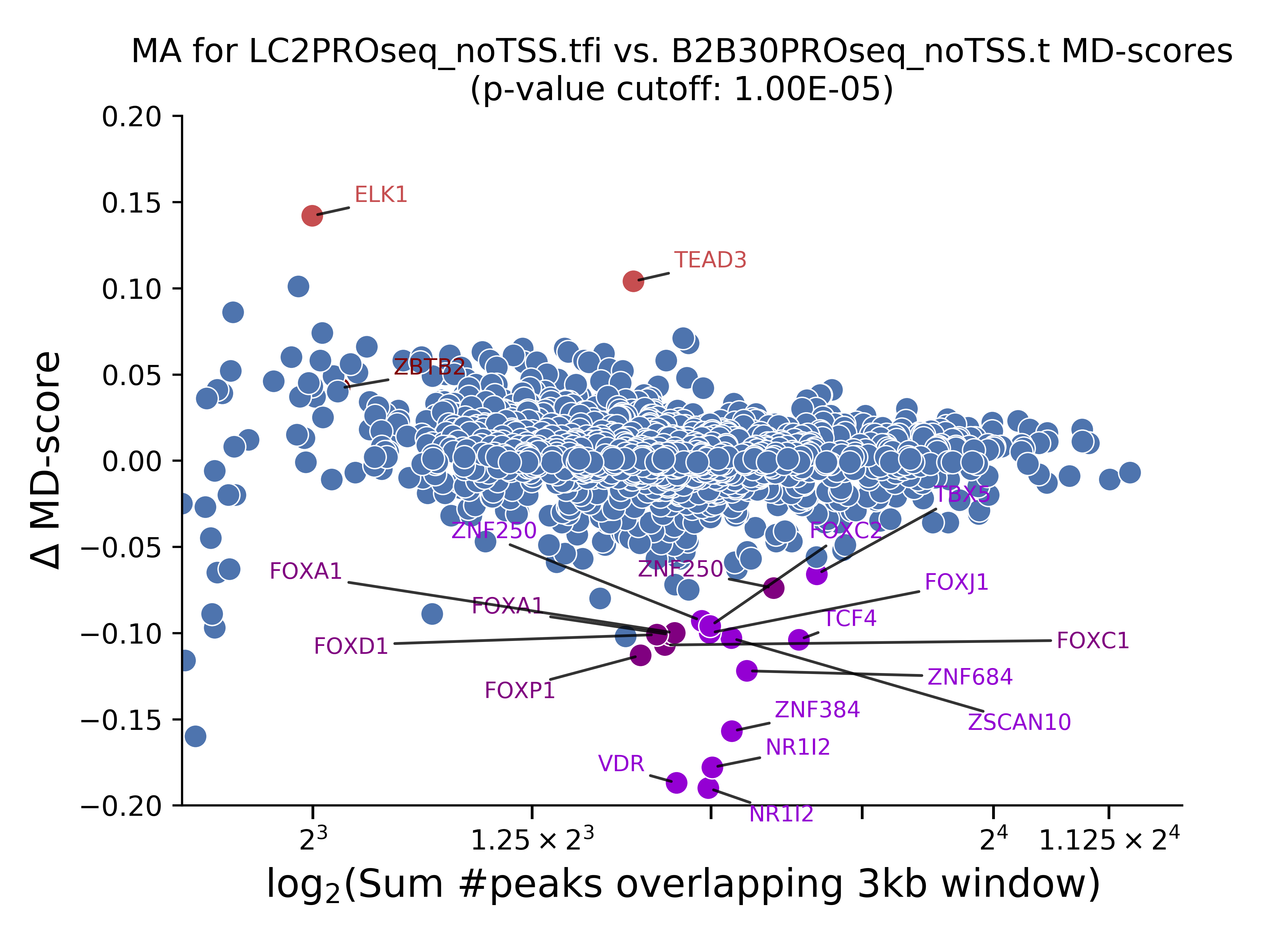

Supplement: Supplemental Data Set 1 [file jciinsight-6-144294-s076.zip › noTSS/best_curated_Human_TFs_p1e-5_grch38/LC2_vs_B2B/MA_LC2PROseq_noTSS.tfit_merged_to_B2B30PROseq_noTSS.tfit_merged_md_score.png]

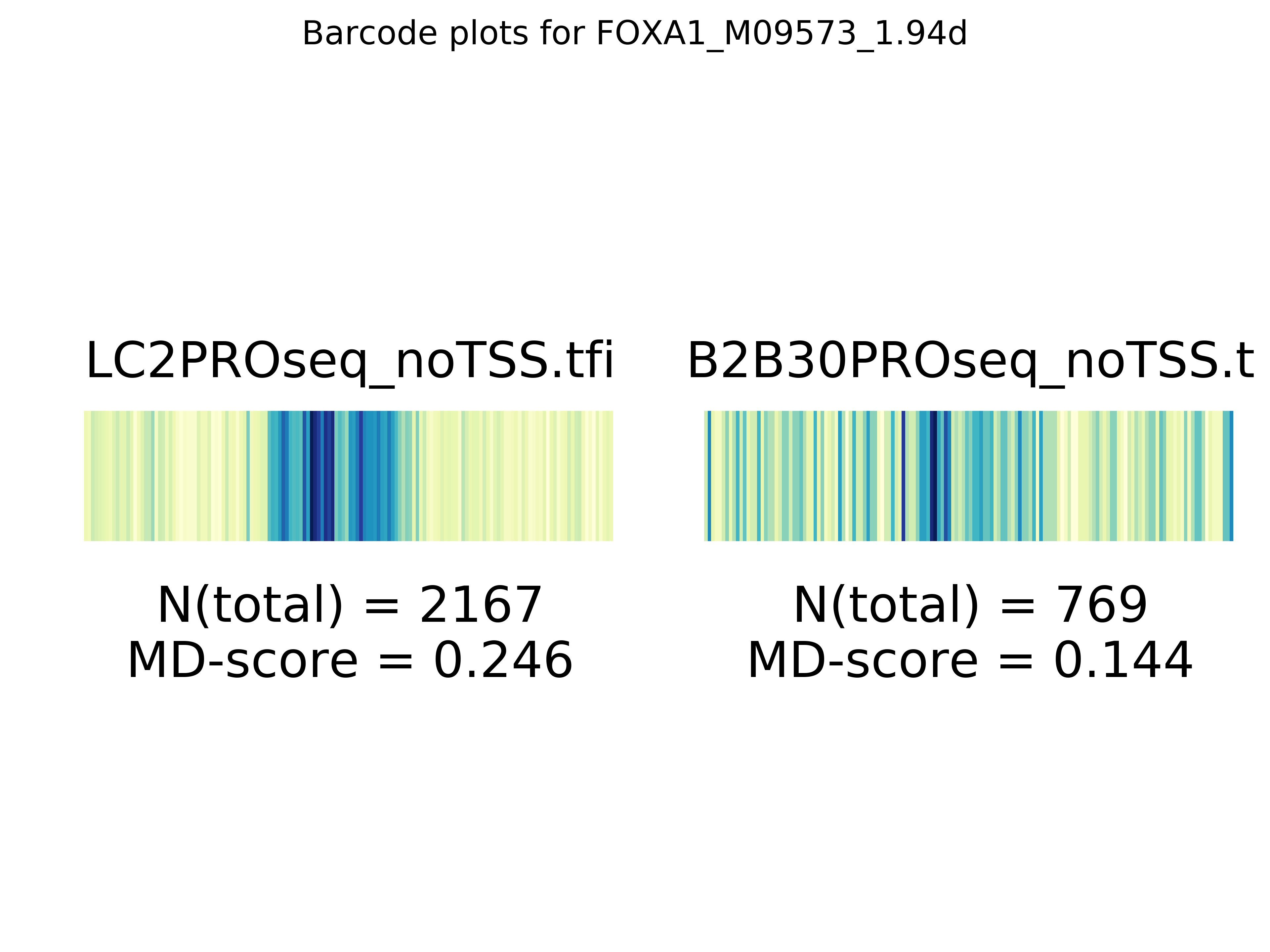

Supplement: Supplemental Data Set 1 [file jciinsight-6-144294-s076.zip › noTSS/best_curated_Human_TFs_p1e-5_grch38/LC2_vs_B2B/FOXA1_M09573_1.94d_barcode_LC2PROseq_noTSS.tfit_merged_vs_B2B30PROseq_noTSS.tfit_merged.png]

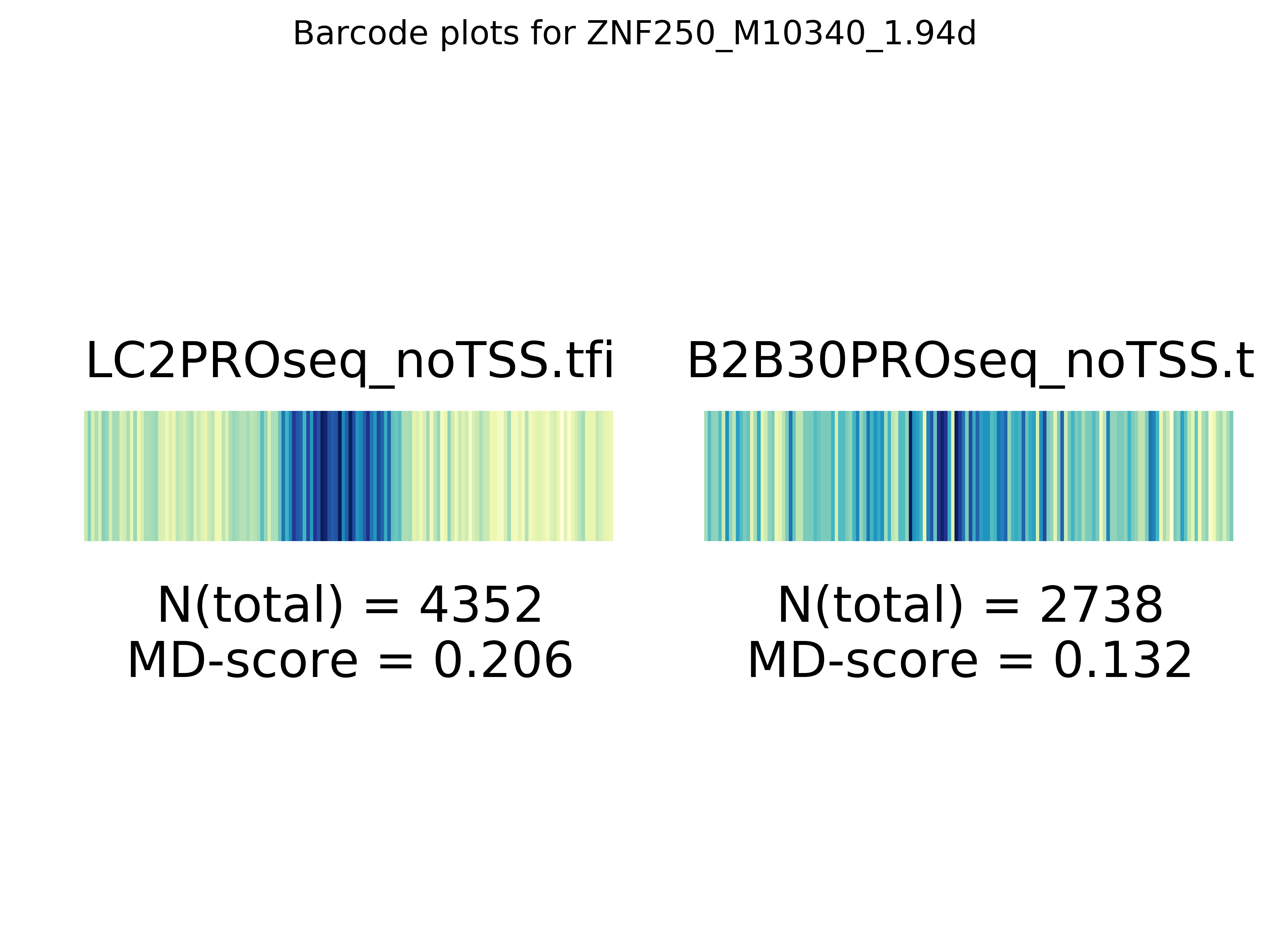

Supplement: Supplemental Data Set 1 [file jciinsight-6-144294-s076.zip › noTSS/best_curated_Human_TFs_p1e-5_grch38/LC2_vs_B2B/ZNF250_M10340_1.94d_barcode_LC2PROseq_noTSS.tfit_merged_vs_B2B30PROseq_noTSS.tfit_merged.png]

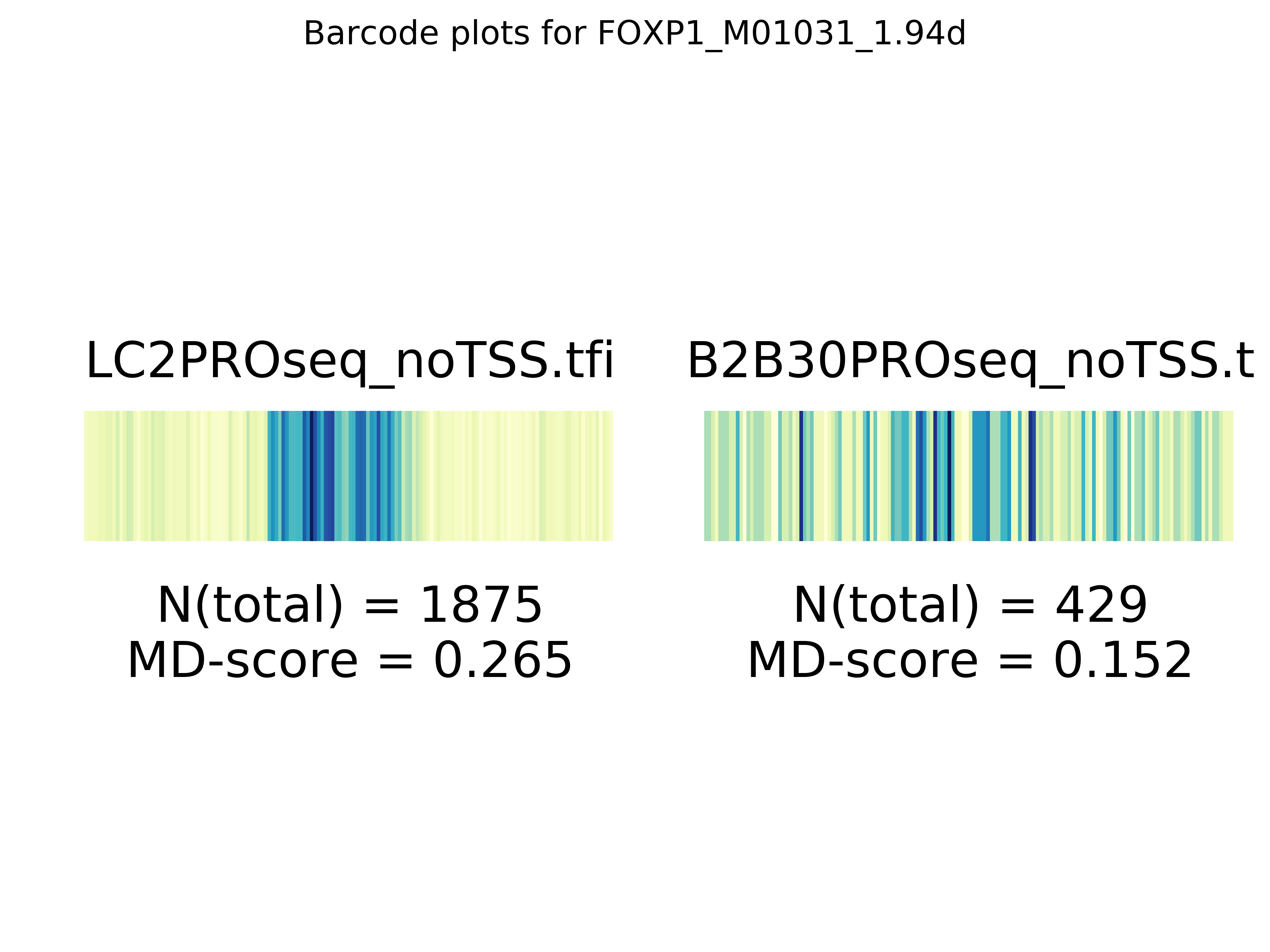

Supplement: Supplemental Data Set 1 [file jciinsight-6-144294-s076.zip › noTSS/best_curated_Human_TFs_p1e-5_grch38/LC2_vs_B2B/FOXP1_M01031_1.94d_barcode_LC2PROseq_noTSS.tfit_merged_vs_B2B30PROseq_noTSS.tfit_merged.png]

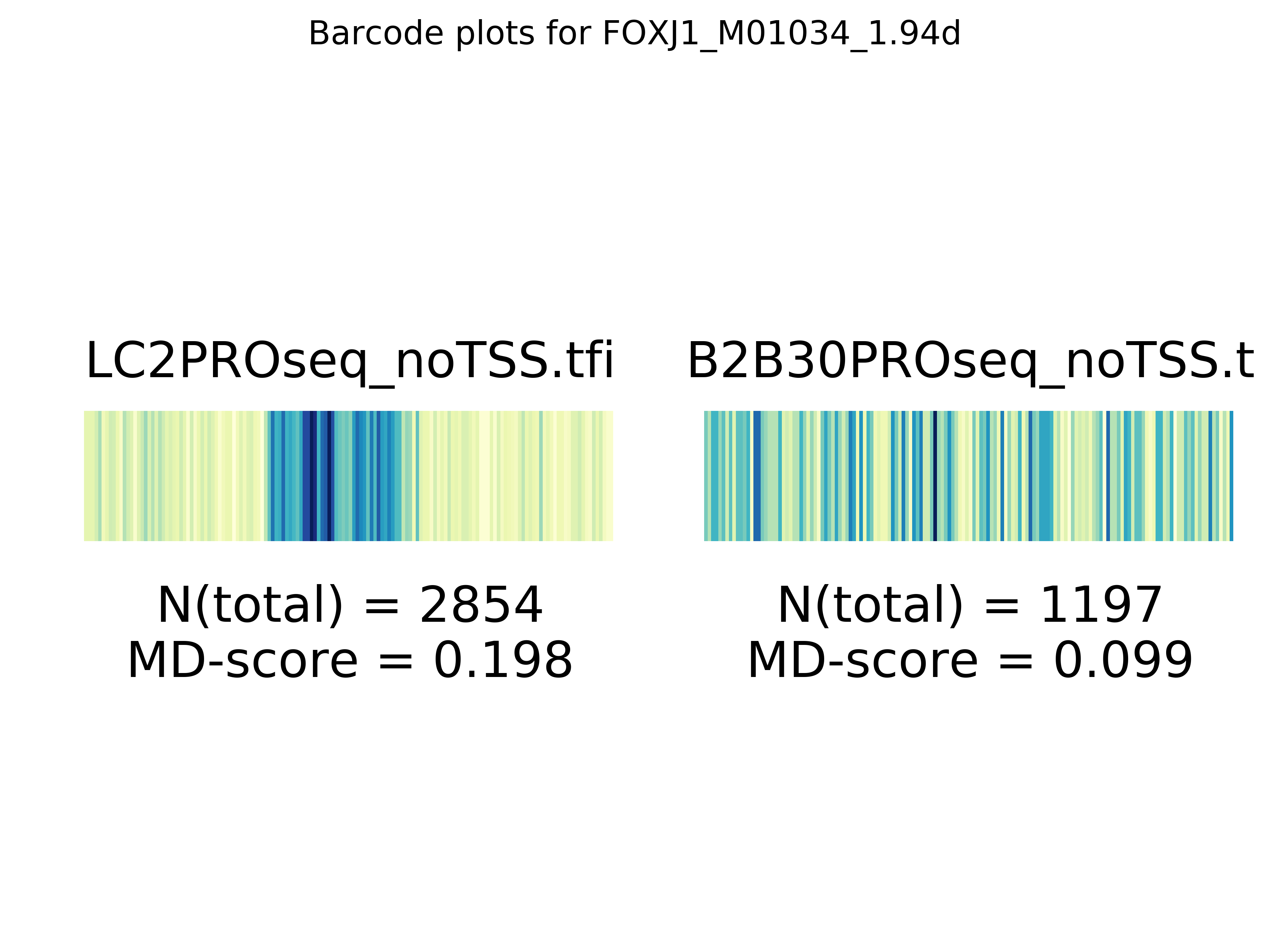

Supplement: Supplemental Data Set 1 [file jciinsight-6-144294-s076.zip › noTSS/best_curated_Human_TFs_p1e-5_grch38/LC2_vs_B2B/FOXJ1_M01034_1.94d_barcode_LC2PROseq_noTSS.tfit_merged_vs_B2B30PROseq_noTSS.tfit_merged.png]

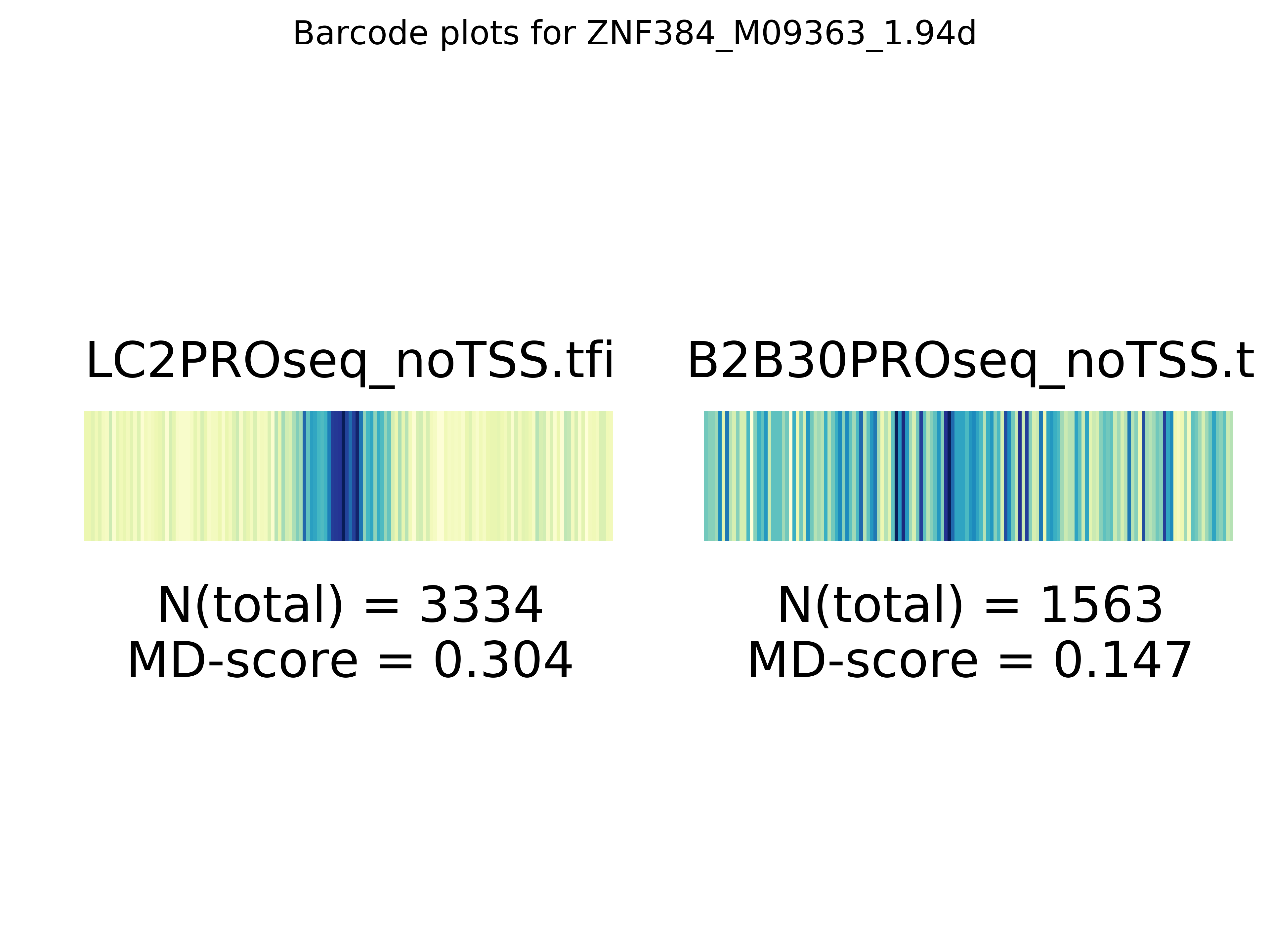

Supplement: Supplemental Data Set 1 [file jciinsight-6-144294-s076.zip › noTSS/best_curated_Human_TFs_p1e-5_grch38/LC2_vs_B2B/ZNF384_M09363_1.94d_barcode_LC2PROseq_noTSS.tfit_merged_vs_B2B30PROseq_noTSS.tfit_merged.png]

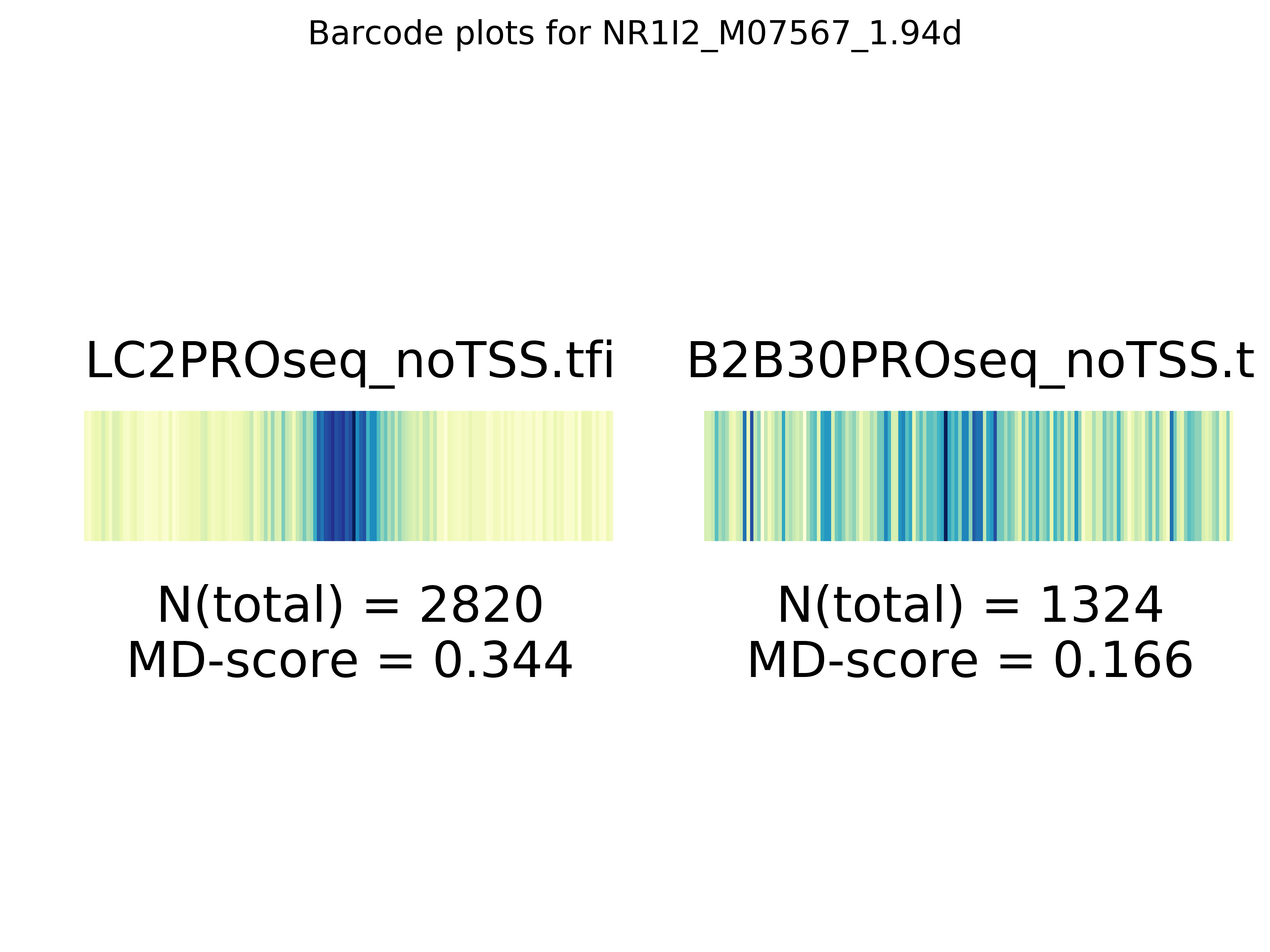

Supplement: Supplemental Data Set 1 [file jciinsight-6-144294-s076.zip › noTSS/best_curated_Human_TFs_p1e-5_grch38/LC2_vs_B2B/NR1I2_M07567_1.94d_barcode_LC2PROseq_noTSS.tfit_merged_vs_B2B30PROseq_noTSS.tfit_merged.png]

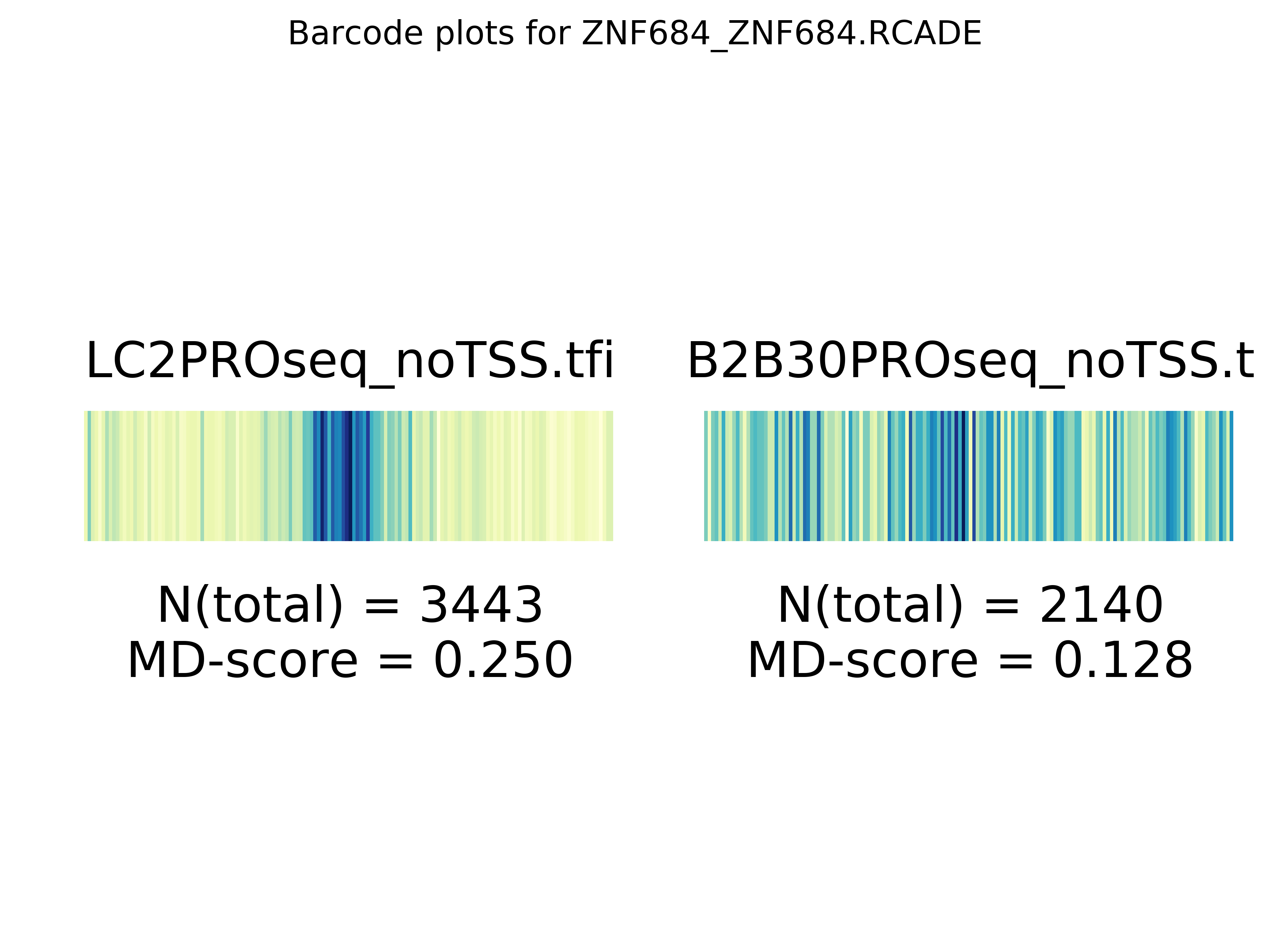

Supplement: Supplemental Data Set 1 [file jciinsight-6-144294-s076.zip › noTSS/best_curated_Human_TFs_p1e-5_grch38/LC2_vs_B2B/ZNF684_ZNF684.RCADE_barcode_LC2PROseq_noTSS.tfit_merged_vs_B2B30PROseq_noTSS.tfit_merged.png]

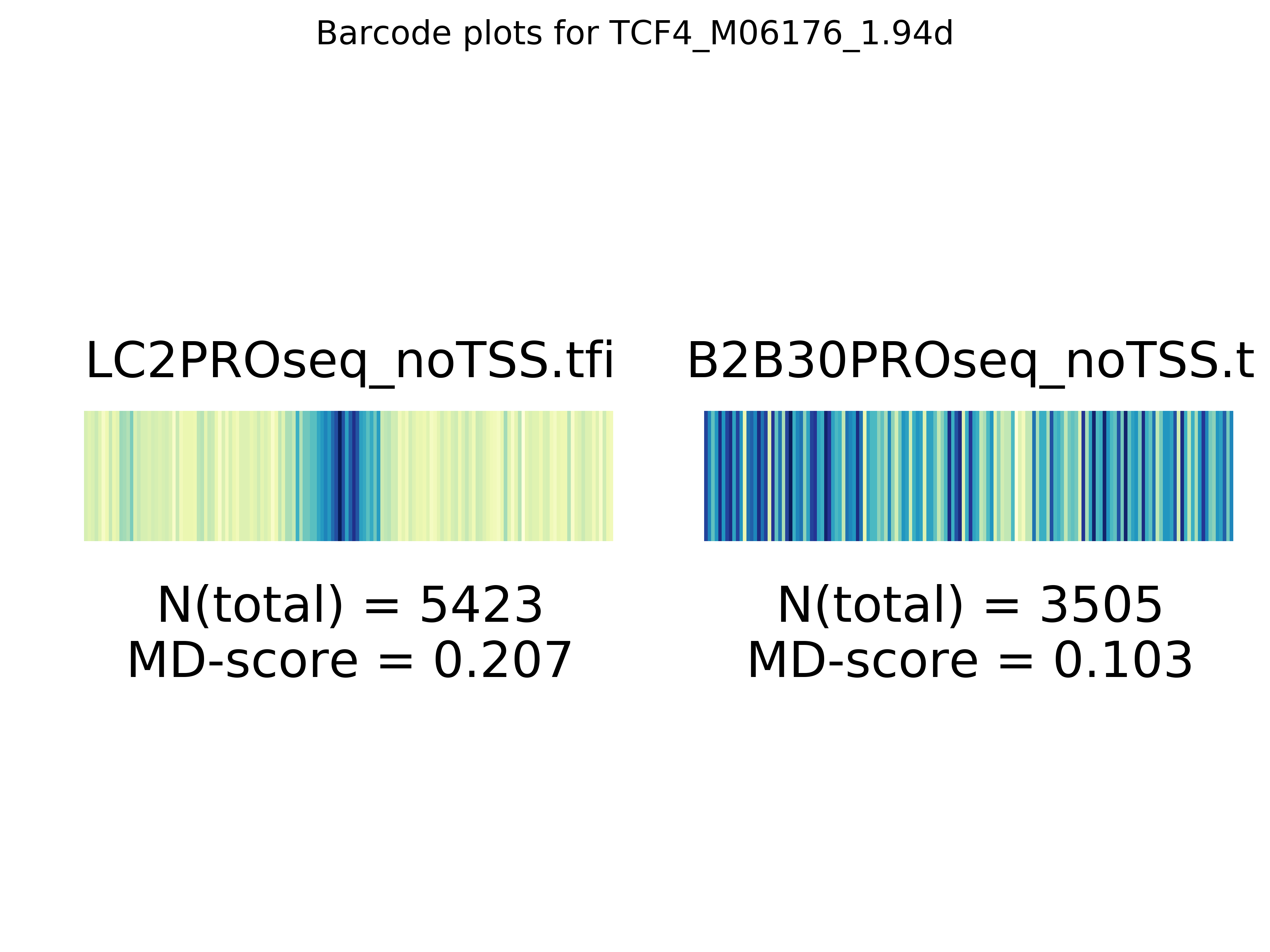

Supplement: Supplemental Data Set 1 [file jciinsight-6-144294-s076.zip › noTSS/best_curated_Human_TFs_p1e-5_grch38/LC2_vs_B2B/TCF4_M06176_1.94d_barcode_LC2PROseq_noTSS.tfit_merged_vs_B2B30PROseq_noTSS.tfit_merged.png]

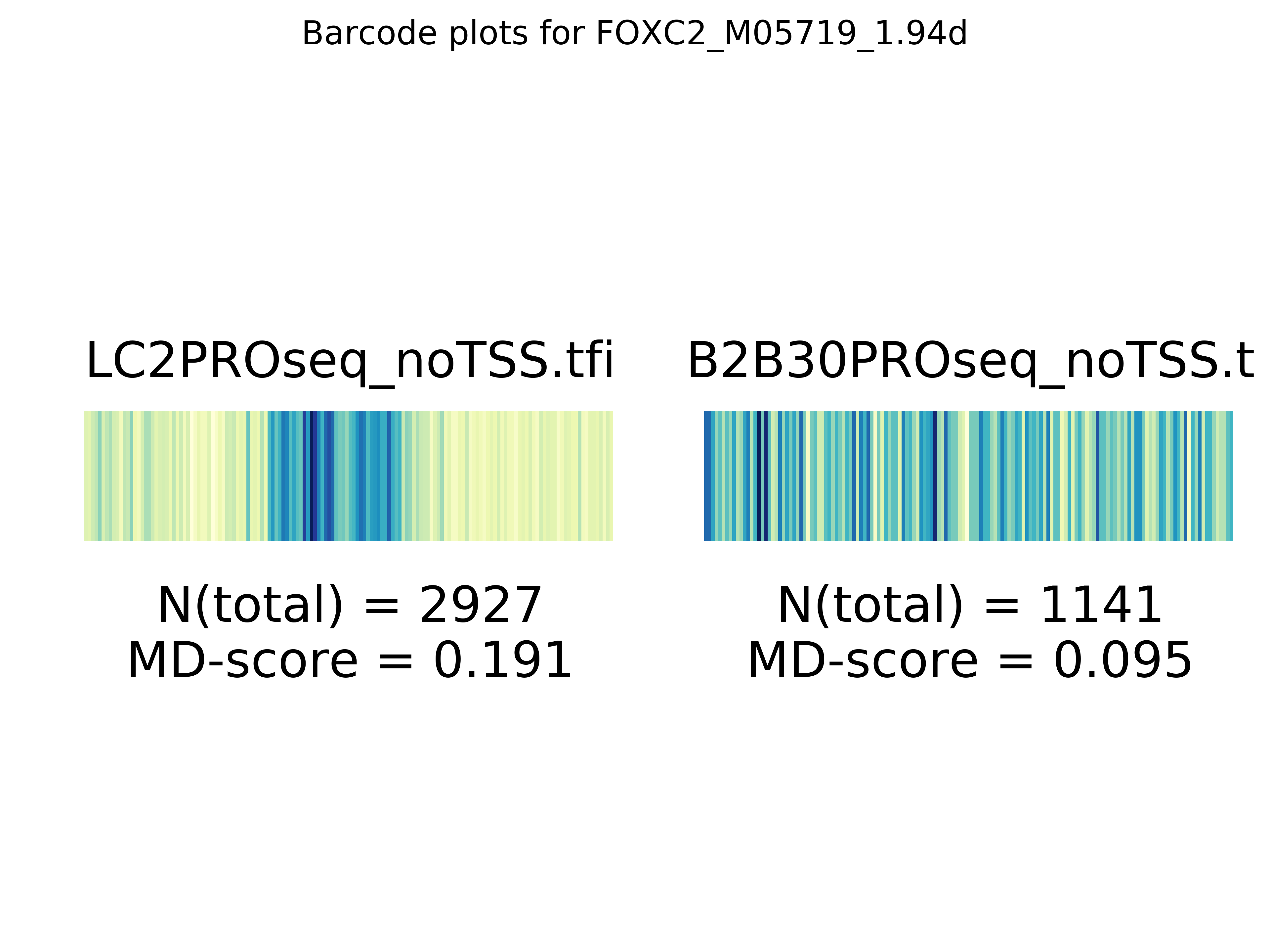

Supplement: Supplemental Data Set 1 [file jciinsight-6-144294-s076.zip › noTSS/best_curated_Human_TFs_p1e-5_grch38/LC2_vs_B2B/FOXC2_M05719_1.94d_barcode_LC2PROseq_noTSS.tfit_merged_vs_B2B30PROseq_noTSS.tfit_merged.png]

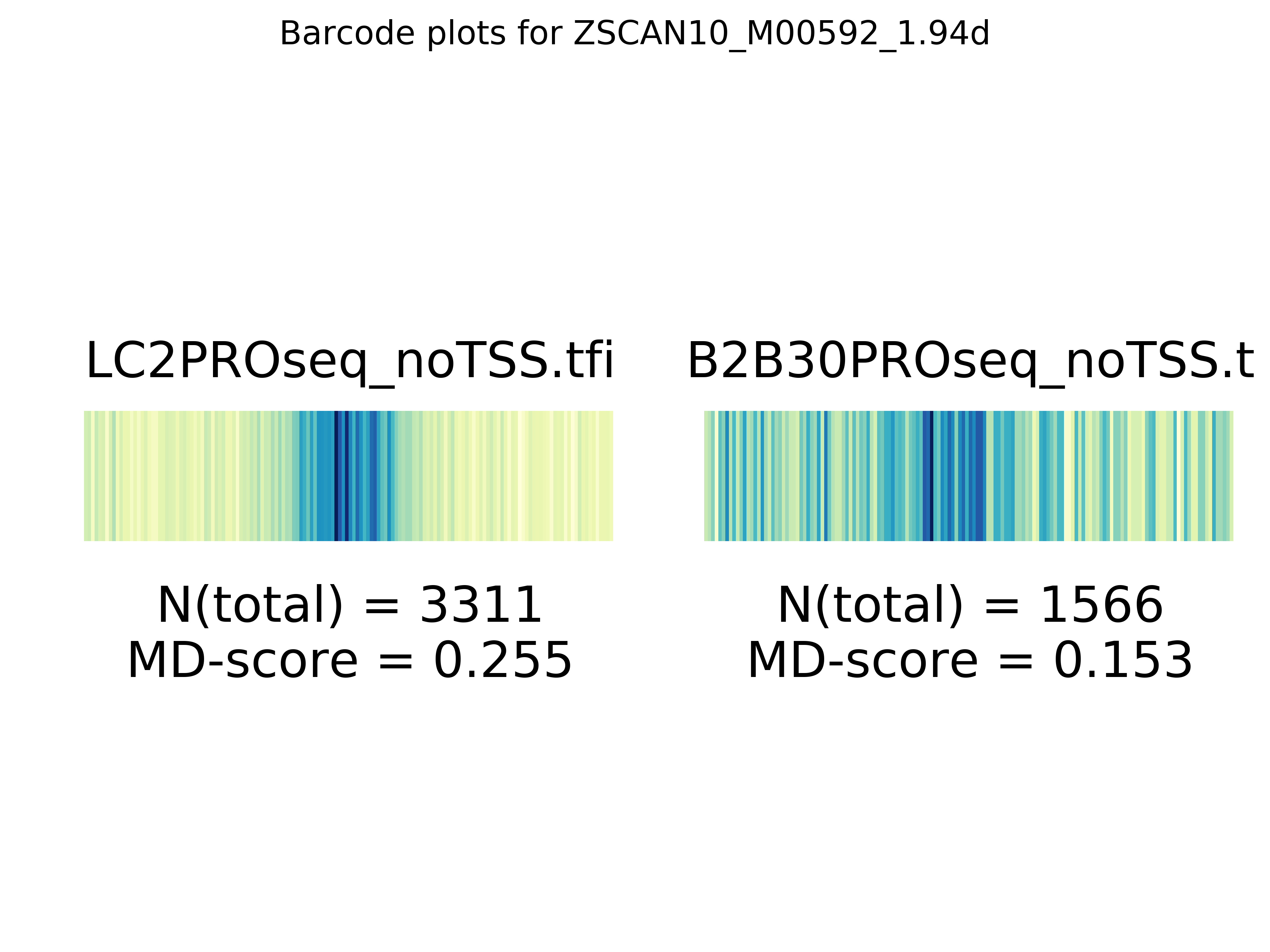

Supplement: Supplemental Data Set 1 [file jciinsight-6-144294-s076.zip › noTSS/best_curated_Human_TFs_p1e-5_grch38/LC2_vs_B2B/ZSCAN10_M00592_1.94d_barcode_LC2PROseq_noTSS.tfit_merged_vs_B2B30PROseq_noTSS.tfit_merged.png]

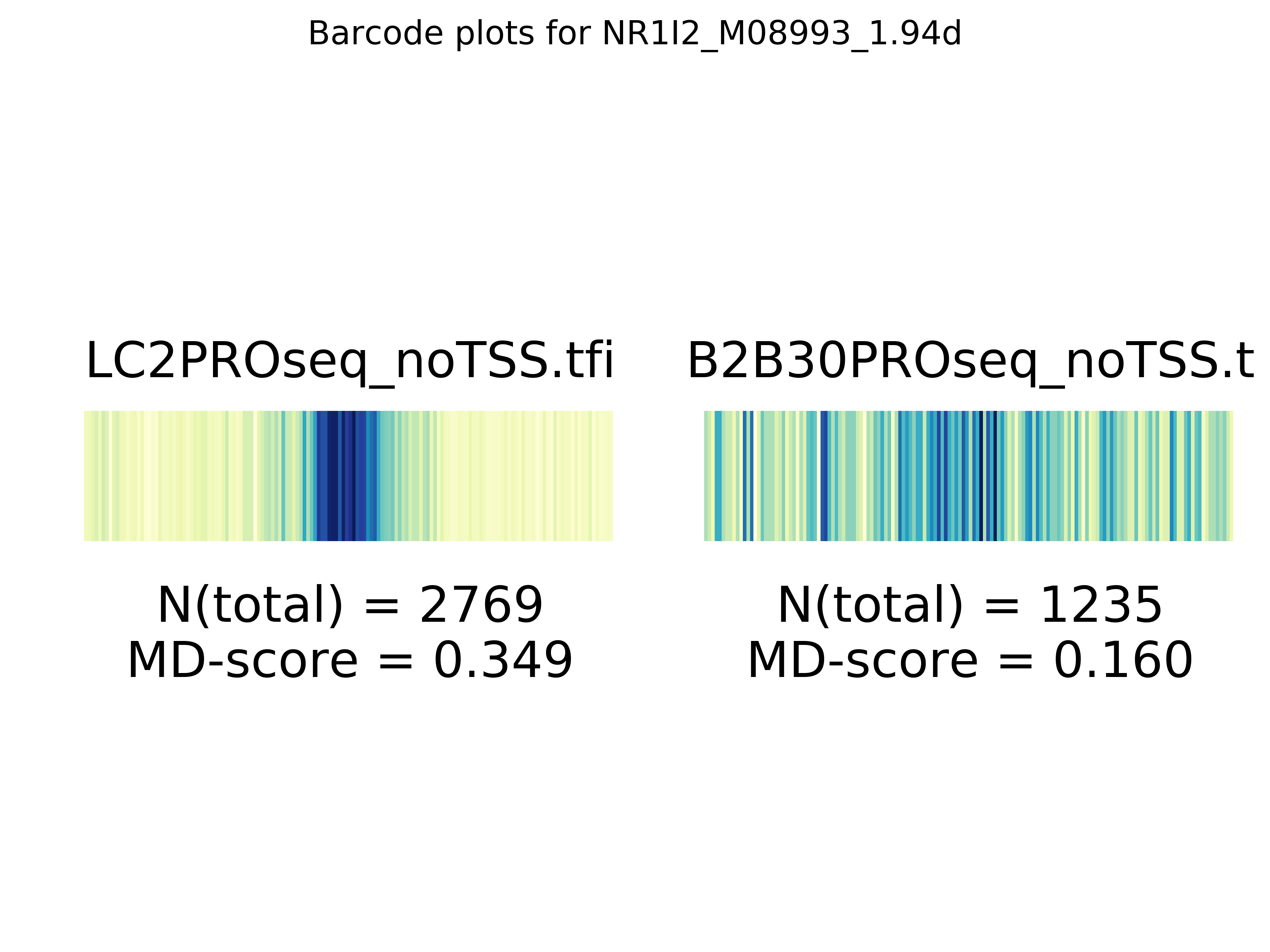

Supplement: Supplemental Data Set 1 [file jciinsight-6-144294-s076.zip › noTSS/best_curated_Human_TFs_p1e-5_grch38/LC2_vs_B2B/NR1I2_M08993_1.94d_barcode_LC2PROseq_noTSS.tfit_merged_vs_B2B30PROseq_noTSS.tfit_merged.png]

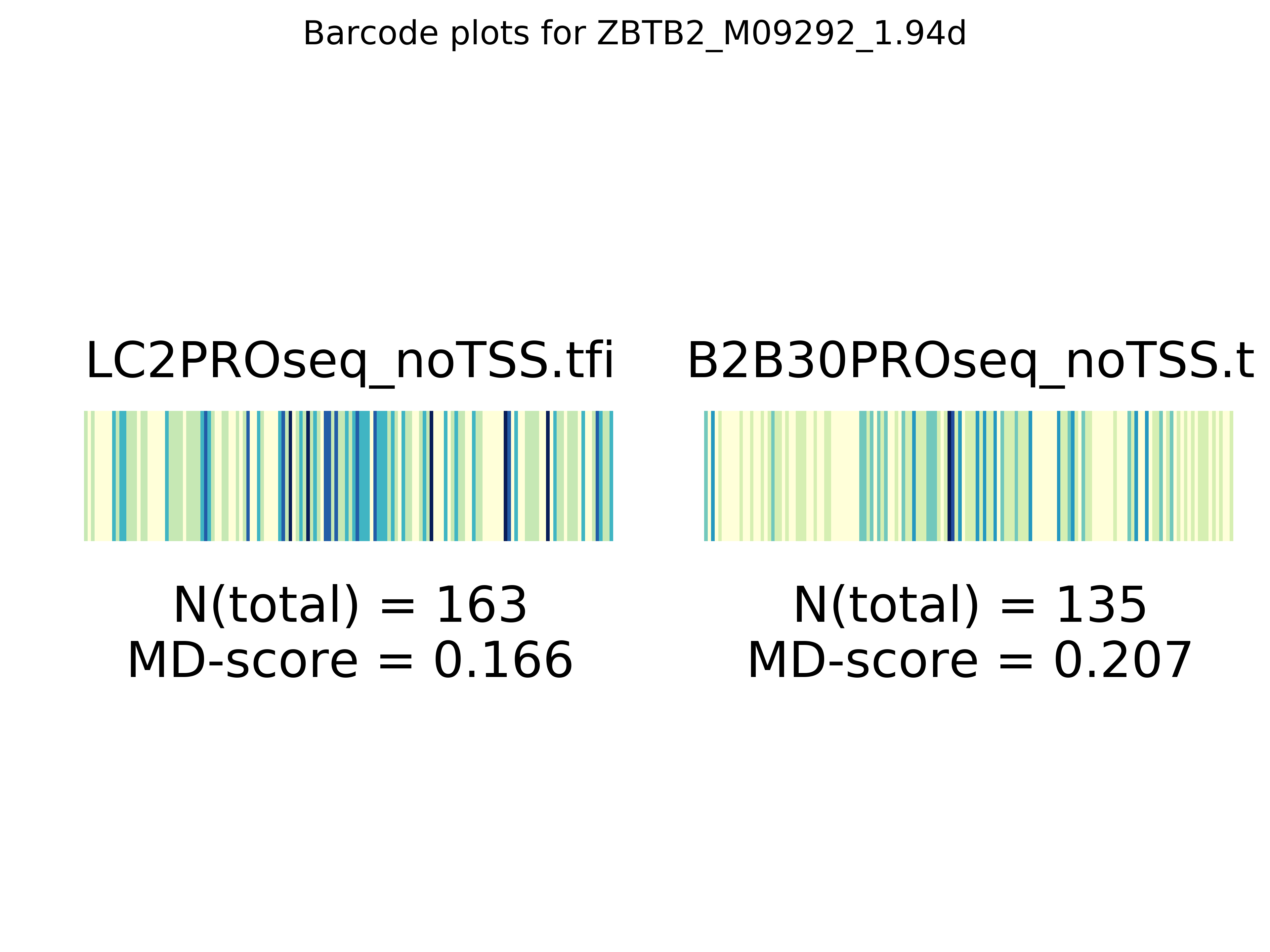

Supplement: Supplemental Data Set 1 [file jciinsight-6-144294-s076.zip › noTSS/best_curated_Human_TFs_p1e-5_grch38/LC2_vs_B2B/ZBTB2_M09292_1.94d_barcode_LC2PROseq_noTSS.tfit_merged_vs_B2B30PROseq_noTSS.tfit_merged.png]

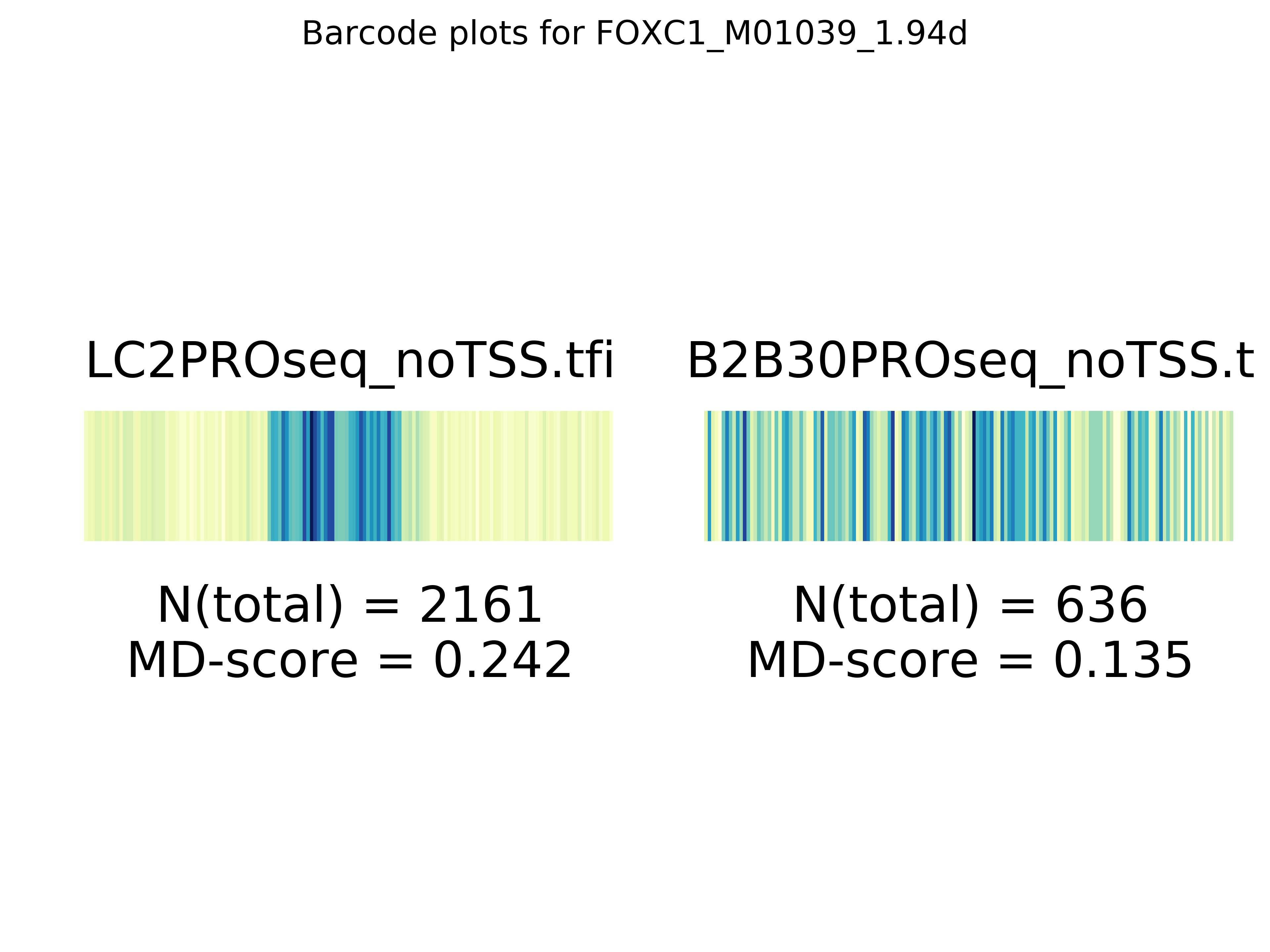

Supplement: Supplemental Data Set 1 [file jciinsight-6-144294-s076.zip › noTSS/best_curated_Human_TFs_p1e-5_grch38/LC2_vs_B2B/FOXC1_M01039_1.94d_barcode_LC2PROseq_noTSS.tfit_merged_vs_B2B30PROseq_noTSS.tfit_merged.png]

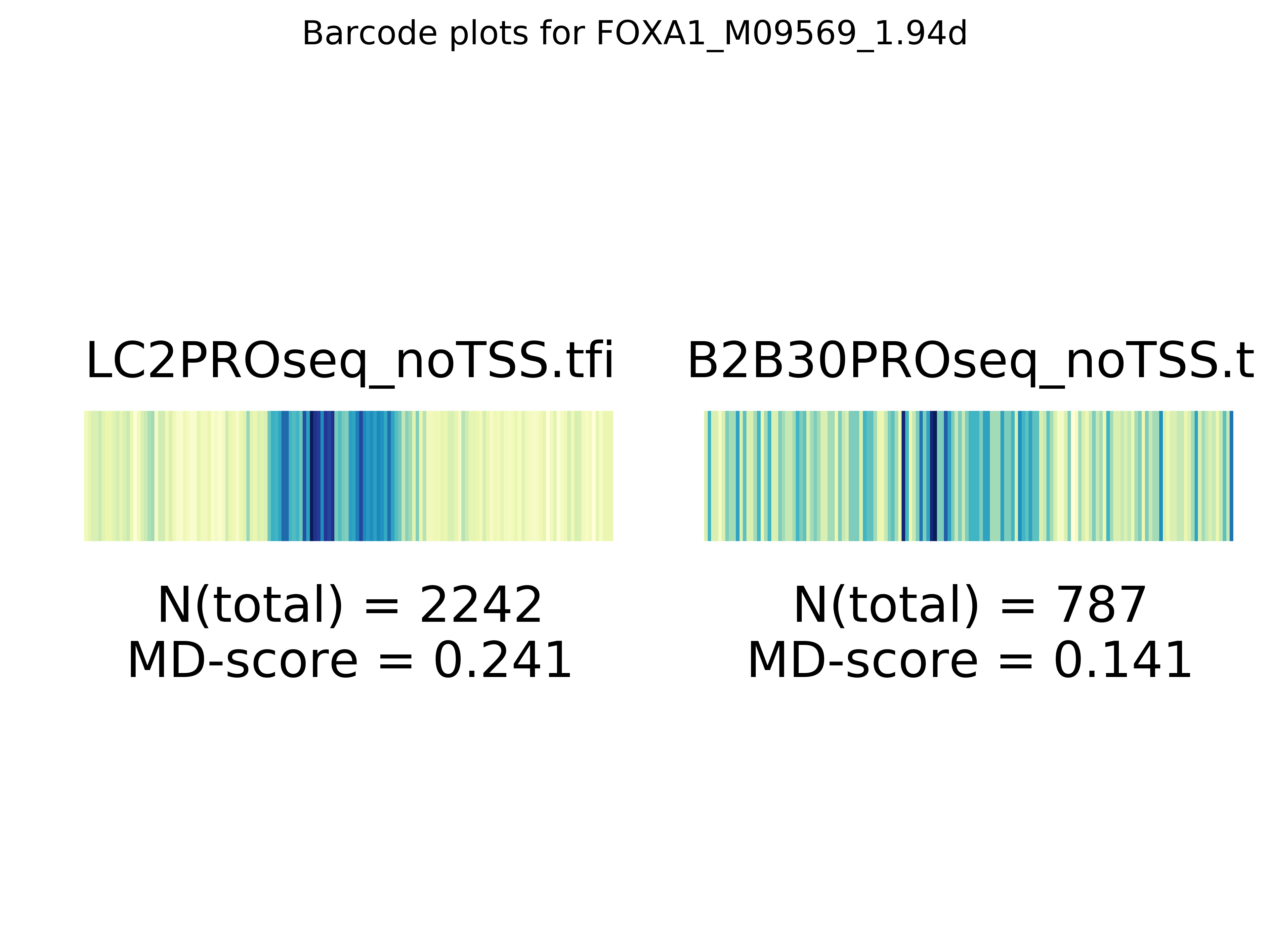

Supplement: Supplemental Data Set 1 [file jciinsight-6-144294-s076.zip › noTSS/best_curated_Human_TFs_p1e-5_grch38/LC2_vs_B2B/FOXA1_M09569_1.94d_barcode_LC2PROseq_noTSS.tfit_merged_vs_B2B30PROseq_noTSS.tfit_merged.png]

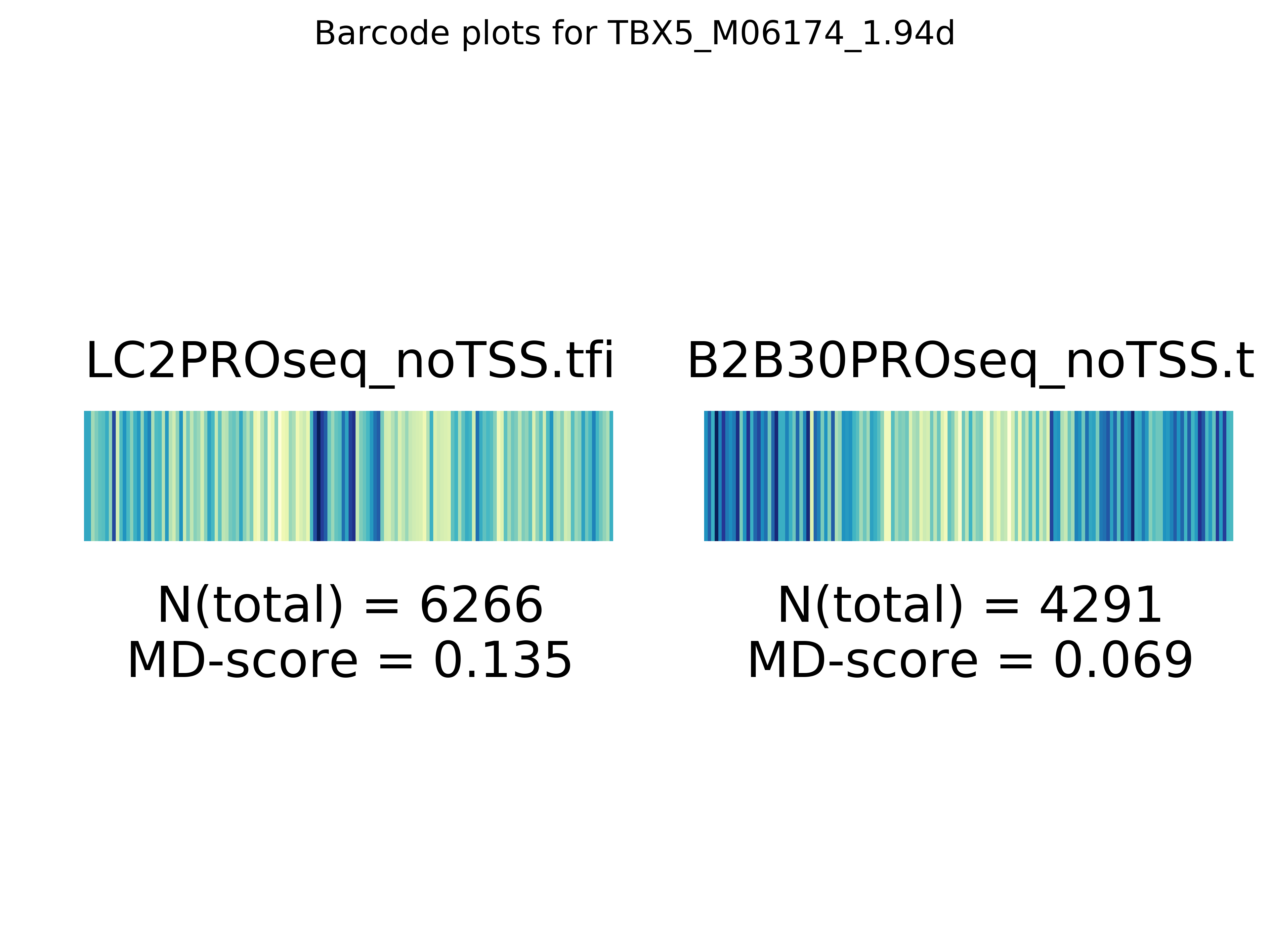

Supplement: Supplemental Data Set 1 [file jciinsight-6-144294-s076.zip › noTSS/best_curated_Human_TFs_p1e-5_grch38/LC2_vs_B2B/TBX5_M06174_1.94d_barcode_LC2PROseq_noTSS.tfit_merged_vs_B2B30PROseq_noTSS.tfit_merged.png]

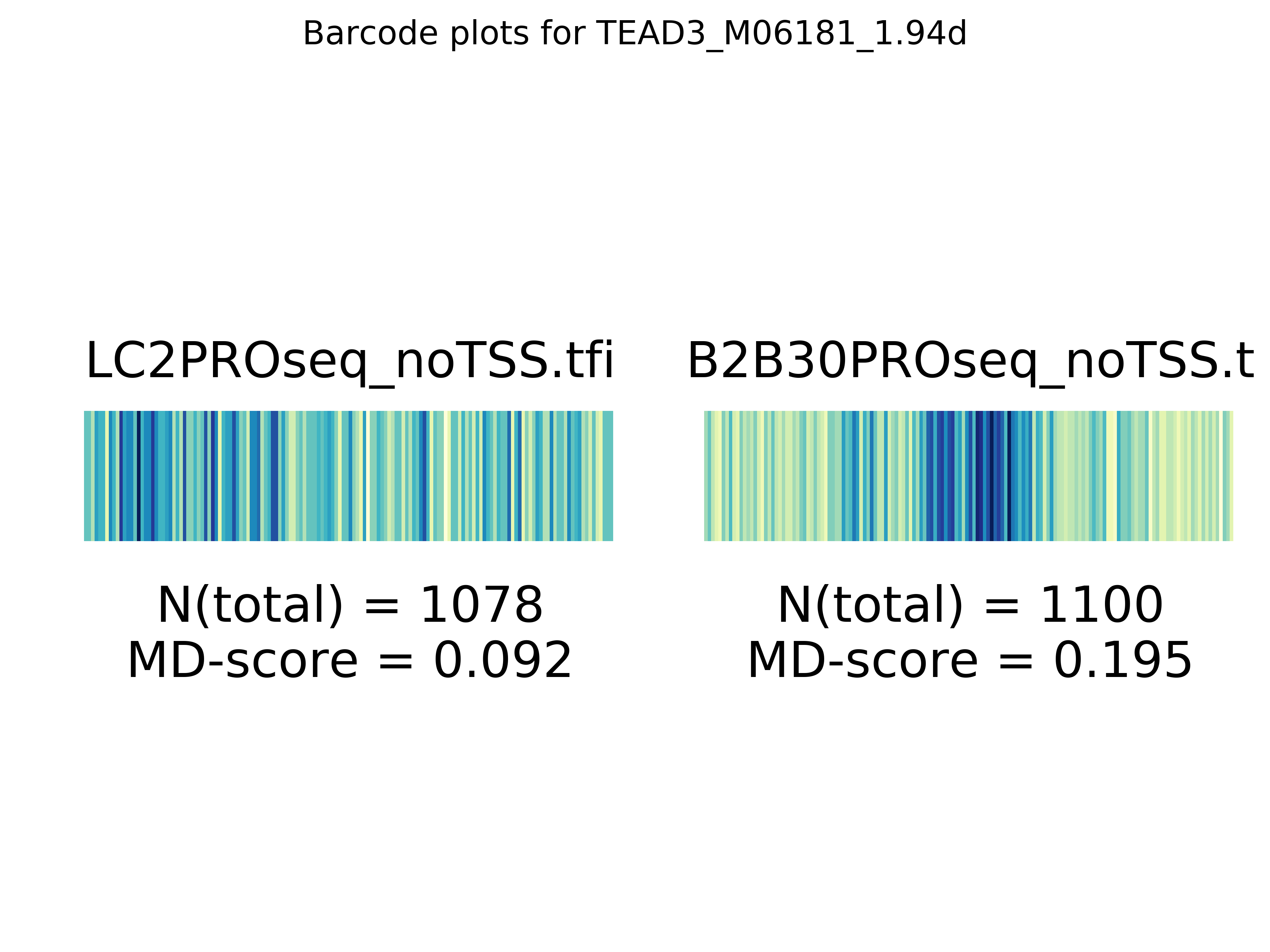

Supplement: Supplemental Data Set 1 [file jciinsight-6-144294-s076.zip › noTSS/best_curated_Human_TFs_p1e-5_grch38/LC2_vs_B2B/TEAD3_M06181_1.94d_barcode_LC2PROseq_noTSS.tfit_merged_vs_B2B30PROseq_noTSS.tfit_merged.png]

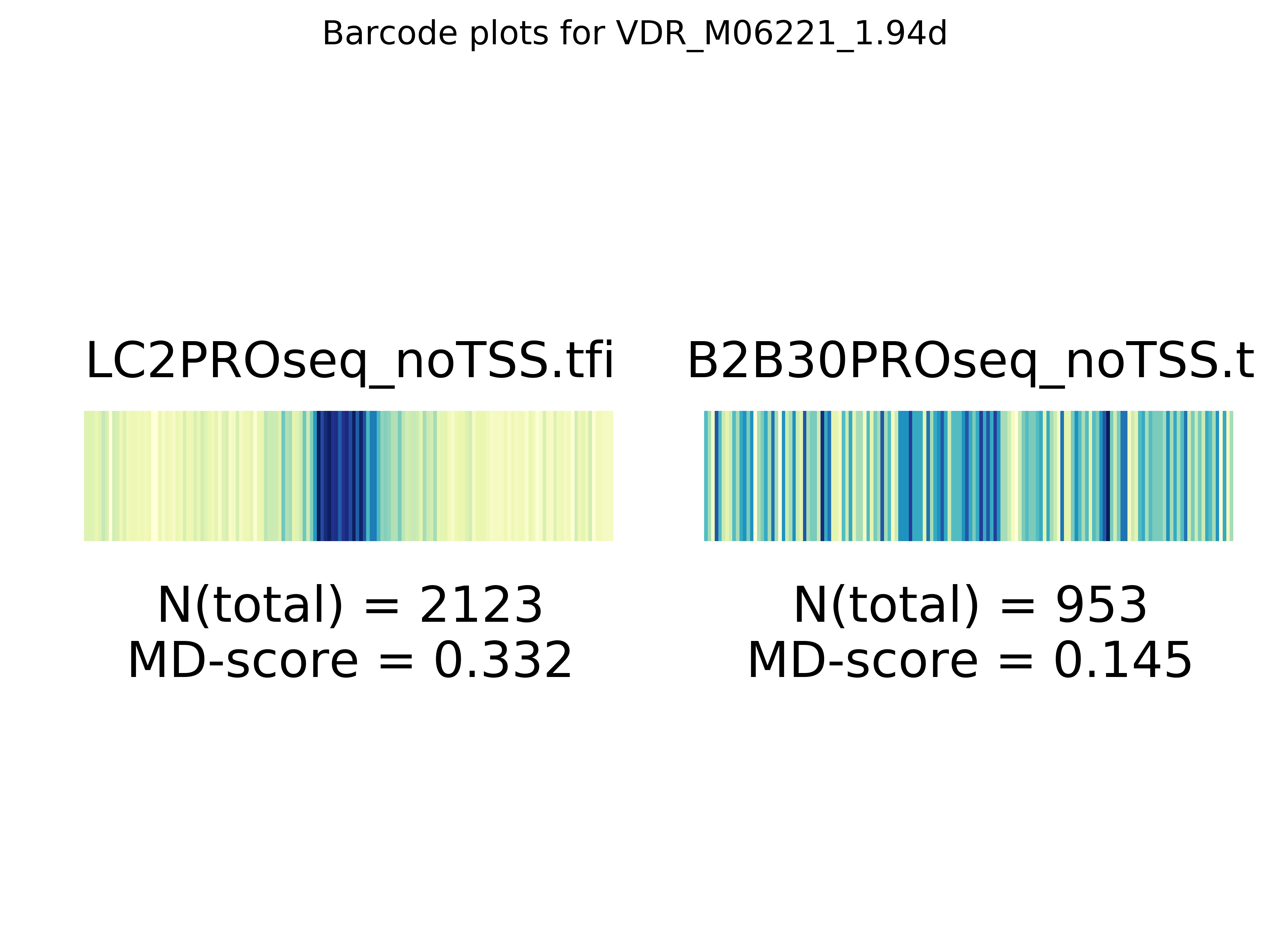

Supplement: Supplemental Data Set 1 [file jciinsight-6-144294-s076.zip › noTSS/best_curated_Human_TFs_p1e-5_grch38/LC2_vs_B2B/VDR_M06221_1.94d_barcode_LC2PROseq_noTSS.tfit_merged_vs_B2B30PROseq_noTSS.tfit_merged.png]

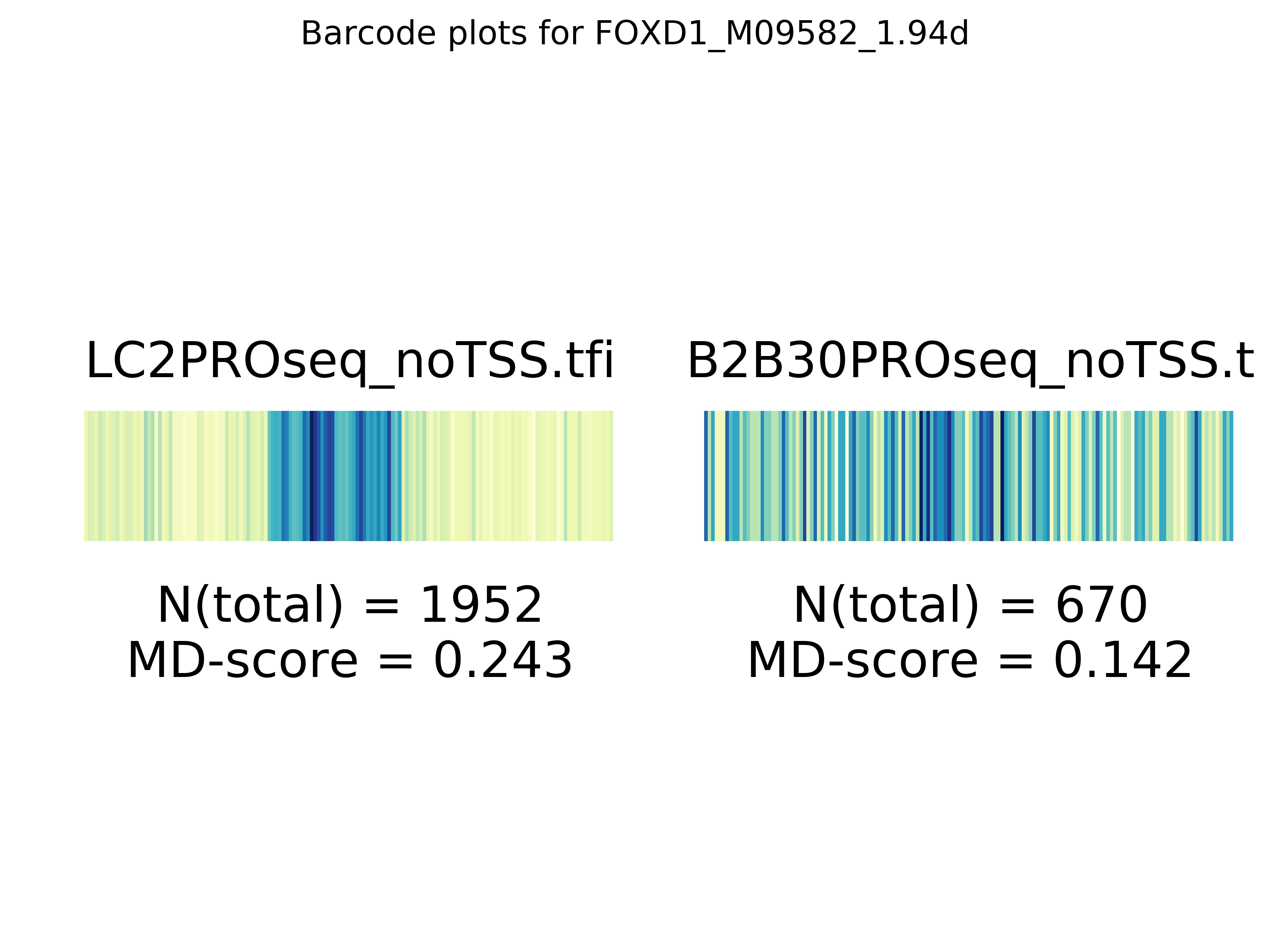

Supplement: Supplemental Data Set 1 [file jciinsight-6-144294-s076.zip › noTSS/best_curated_Human_TFs_p1e-5_grch38/LC2_vs_B2B/FOXD1_M09582_1.94d_barcode_LC2PROseq_noTSS.tfit_merged_vs_B2B30PROseq_noTSS.tfit_merged.png]

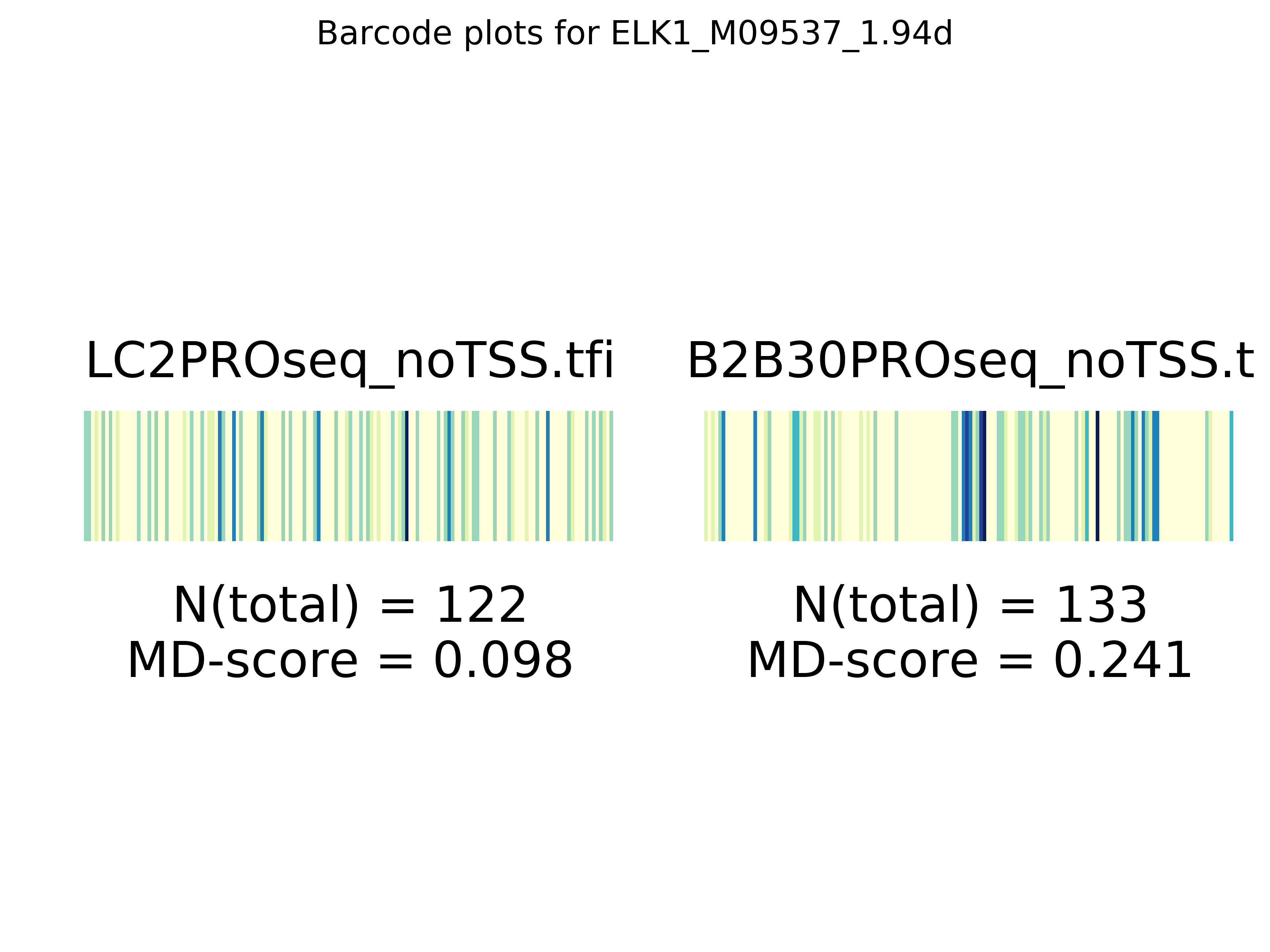

Supplement: Supplemental Data Set 1 [file jciinsight-6-144294-s076.zip › noTSS/best_curated_Human_TFs_p1e-5_grch38/LC2_vs_B2B/ELK1_M09537_1.94d_barcode_LC2PROseq_noTSS.tfit_merged_vs_B2B30PROseq_noTSS.tfit_merged.png]

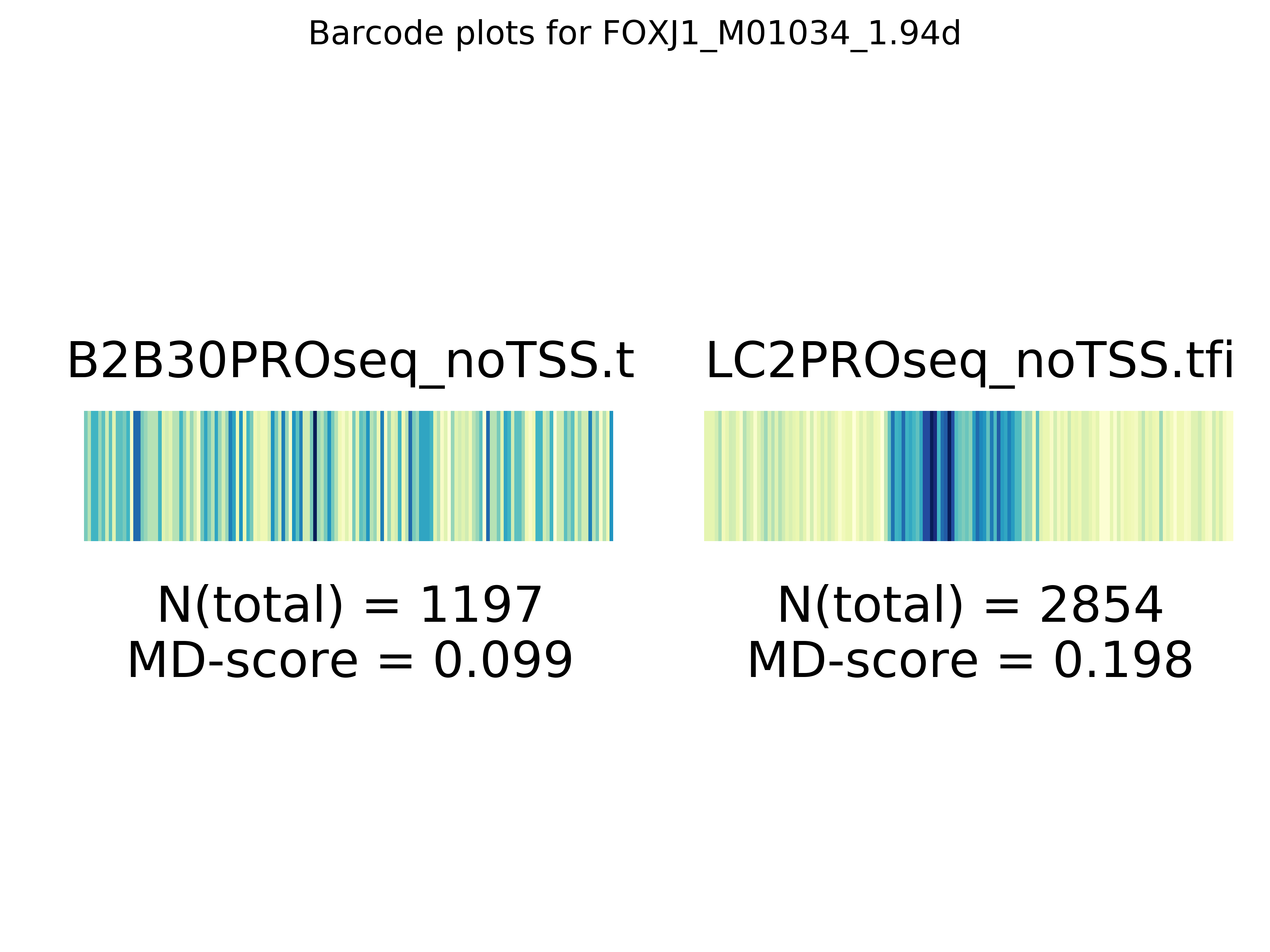

Supplement: Supplemental Data Set 1 [file jciinsight-6-144294-s076.zip › noTSS/best_curated_Human_TFs_p1e-5_grch38/B2B_vs_LC2/FOXJ1_M01034_1.94d_barcode_B2B30PROseq_noTSS.tfit_merged_vs_LC2PROseq_noTSS.tfit_merged.png]

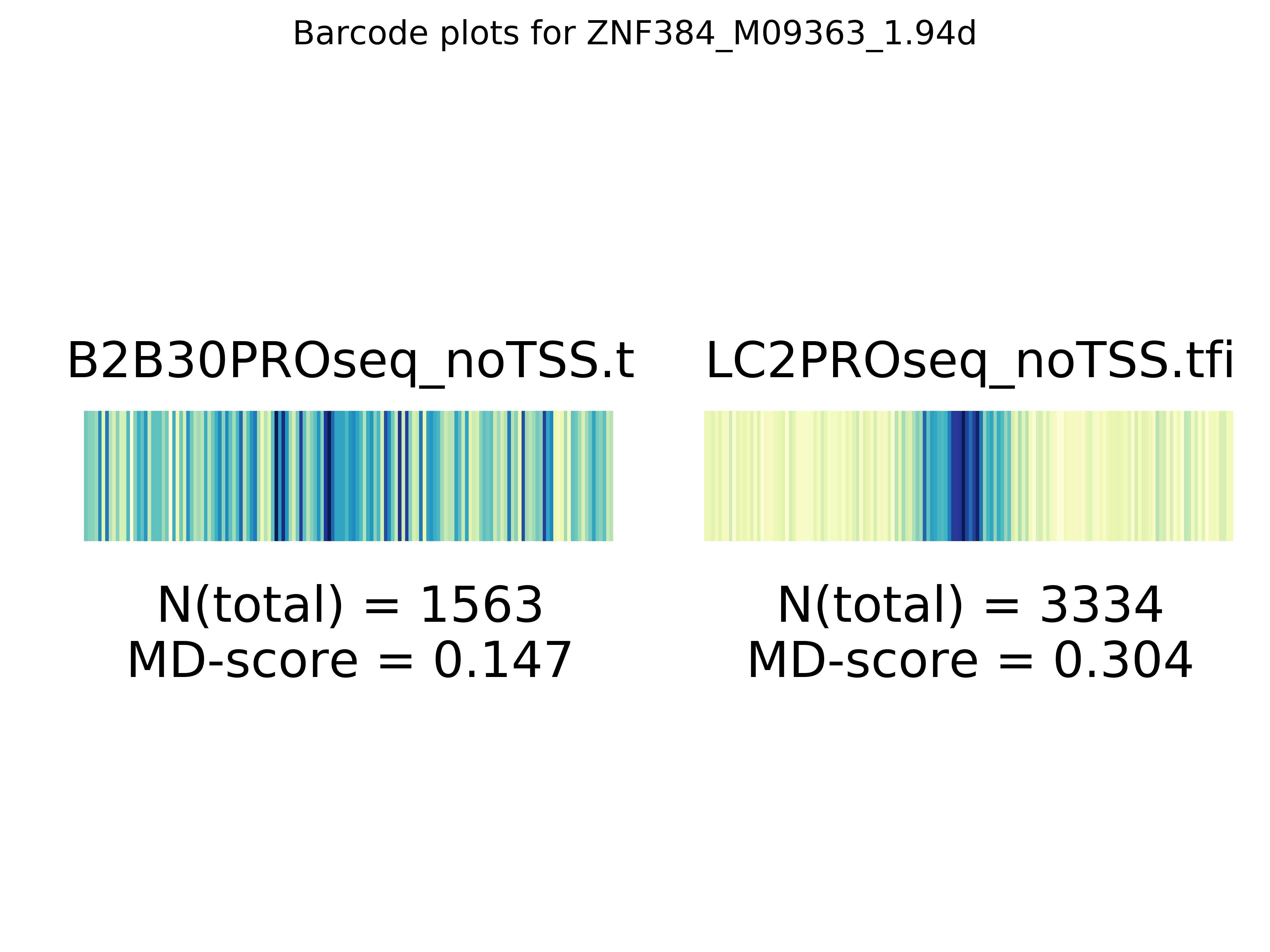

Supplement: Supplemental Data Set 1 [file jciinsight-6-144294-s076.zip › noTSS/best_curated_Human_TFs_p1e-5_grch38/B2B_vs_LC2/ZNF384_M09363_1.94d_barcode_B2B30PROseq_noTSS.tfit_merged_vs_LC2PROseq_noTSS.tfit_merged.png]

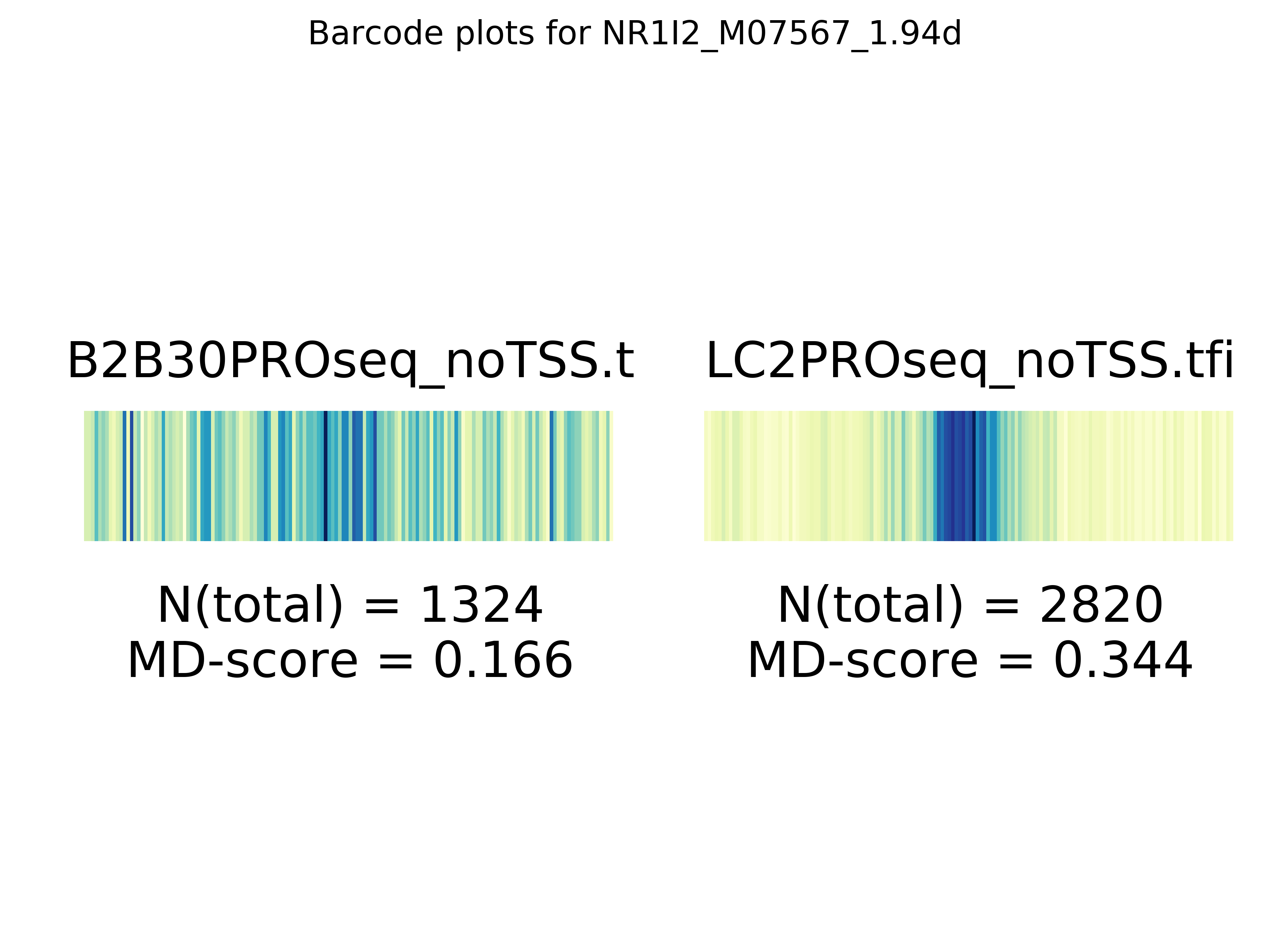

Supplement: Supplemental Data Set 1 [file jciinsight-6-144294-s076.zip › noTSS/best_curated_Human_TFs_p1e-5_grch38/B2B_vs_LC2/NR1I2_M07567_1.94d_barcode_B2B30PROseq_noTSS.tfit_merged_vs_LC2PROseq_noTSS.tfit_merged.png]

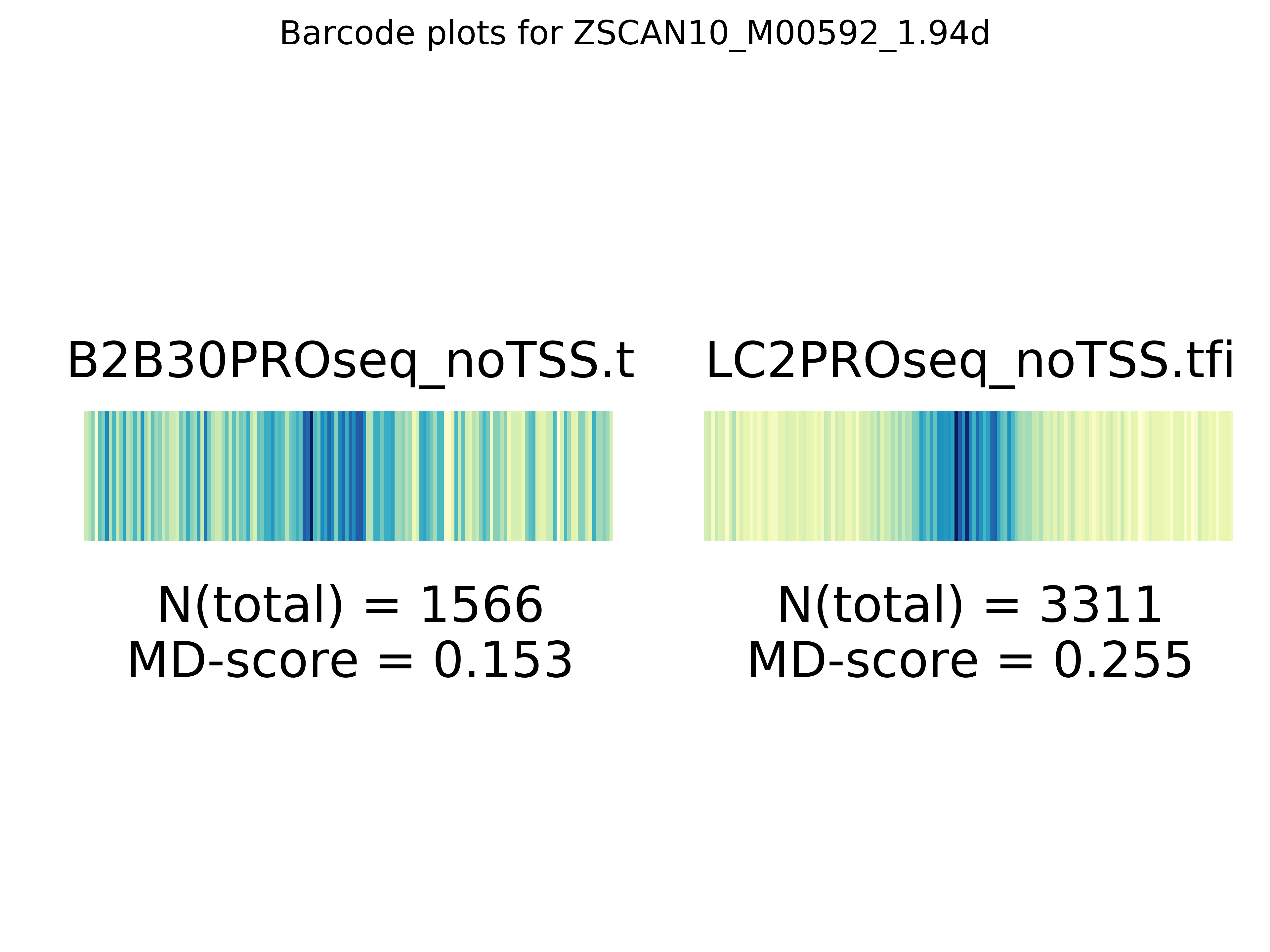

Supplement: Supplemental Data Set 1 [file jciinsight-6-144294-s076.zip › noTSS/best_curated_Human_TFs_p1e-5_grch38/B2B_vs_LC2/ZSCAN10_M00592_1.94d_barcode_B2B30PROseq_noTSS.tfit_merged_vs_LC2PROseq_noTSS.tfit_merged.png]

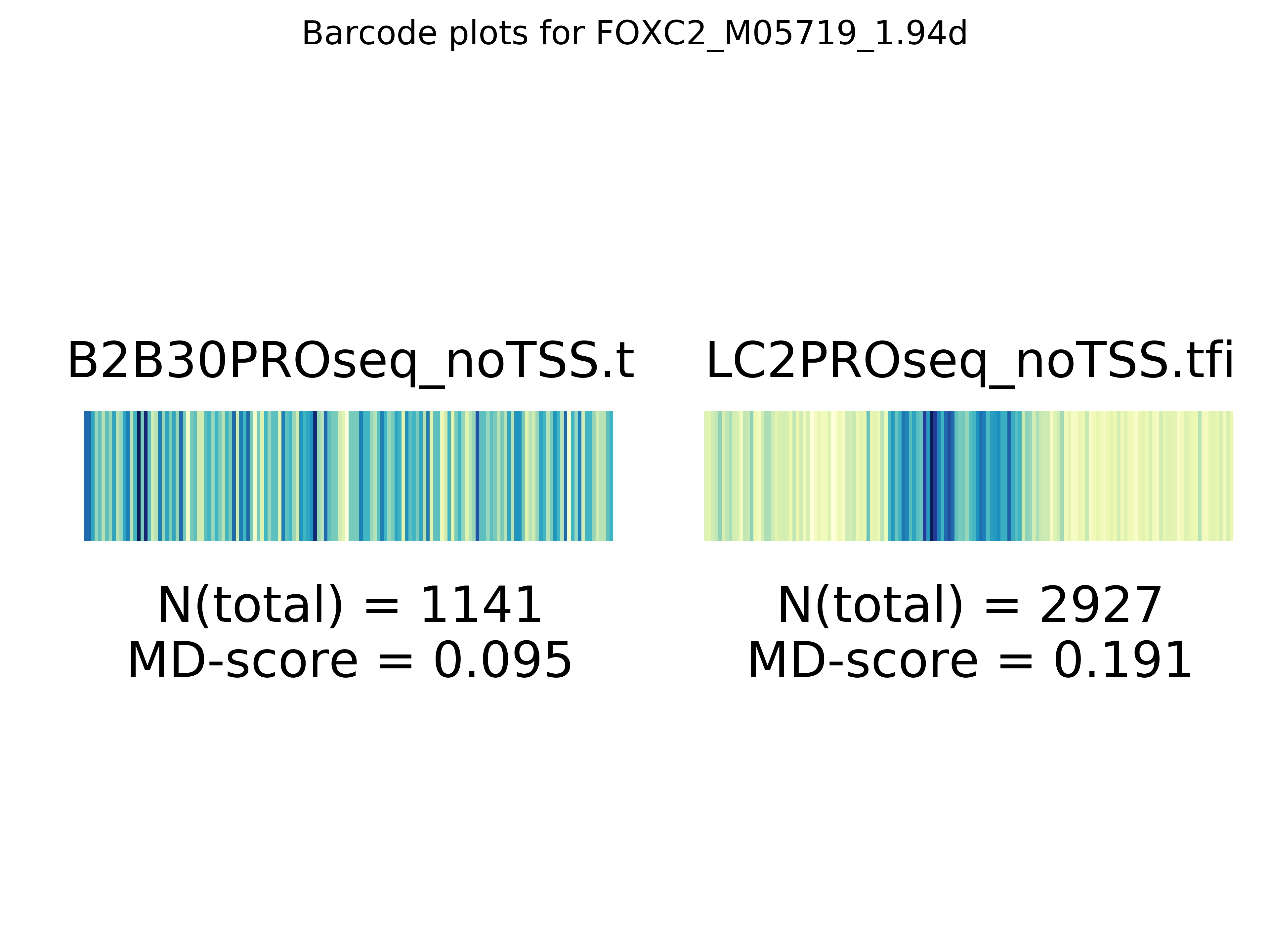

Supplement: Supplemental Data Set 1 [file jciinsight-6-144294-s076.zip › noTSS/best_curated_Human_TFs_p1e-5_grch38/B2B_vs_LC2/FOXC2_M05719_1.94d_barcode_B2B30PROseq_noTSS.tfit_merged_vs_LC2PROseq_noTSS.tfit_merged.png]

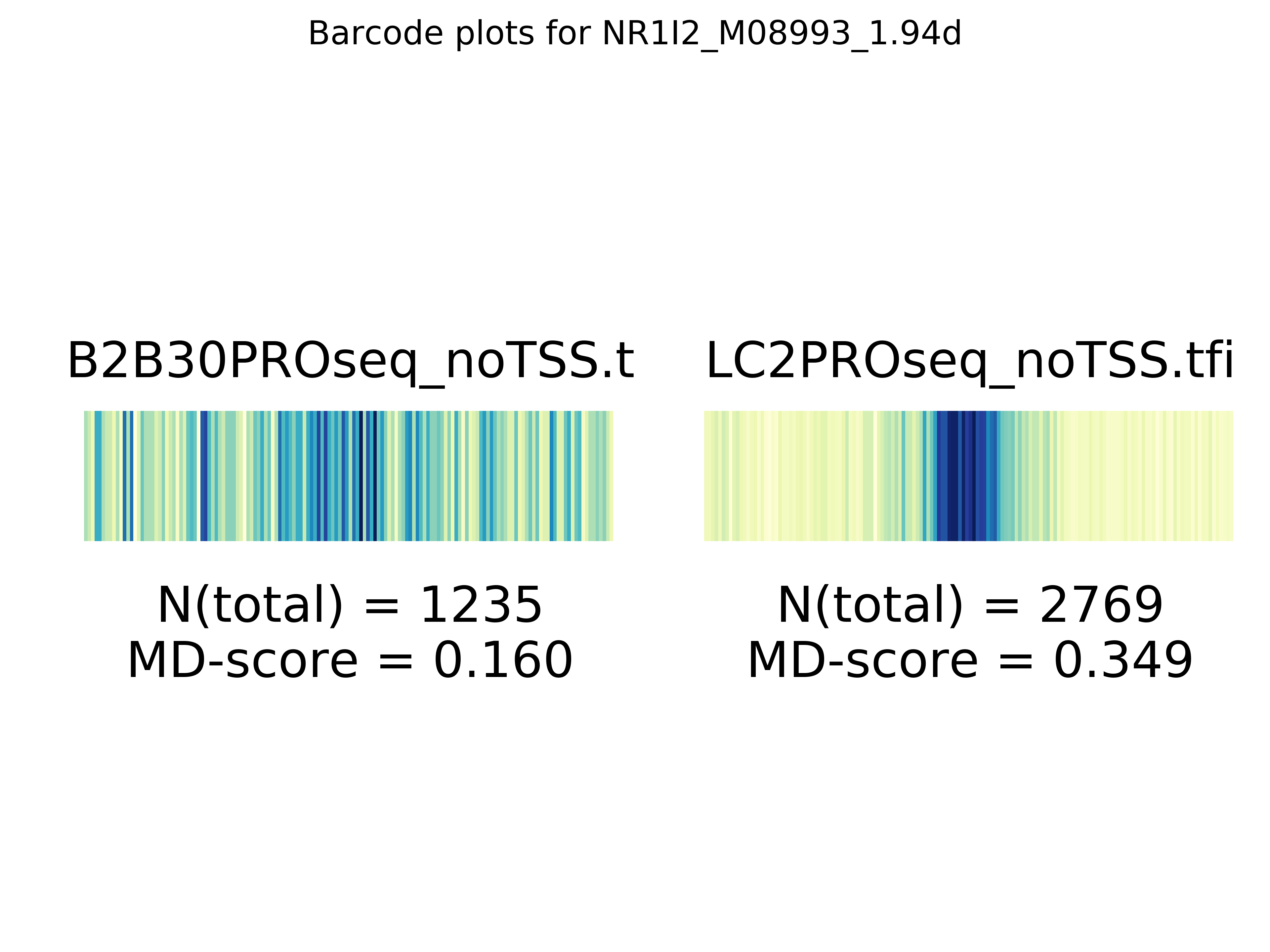

Supplement: Supplemental Data Set 1 [file jciinsight-6-144294-s076.zip › noTSS/best_curated_Human_TFs_p1e-5_grch38/B2B_vs_LC2/NR1I2_M08993_1.94d_barcode_B2B30PROseq_noTSS.tfit_merged_vs_LC2PROseq_noTSS.tfit_merged.png]

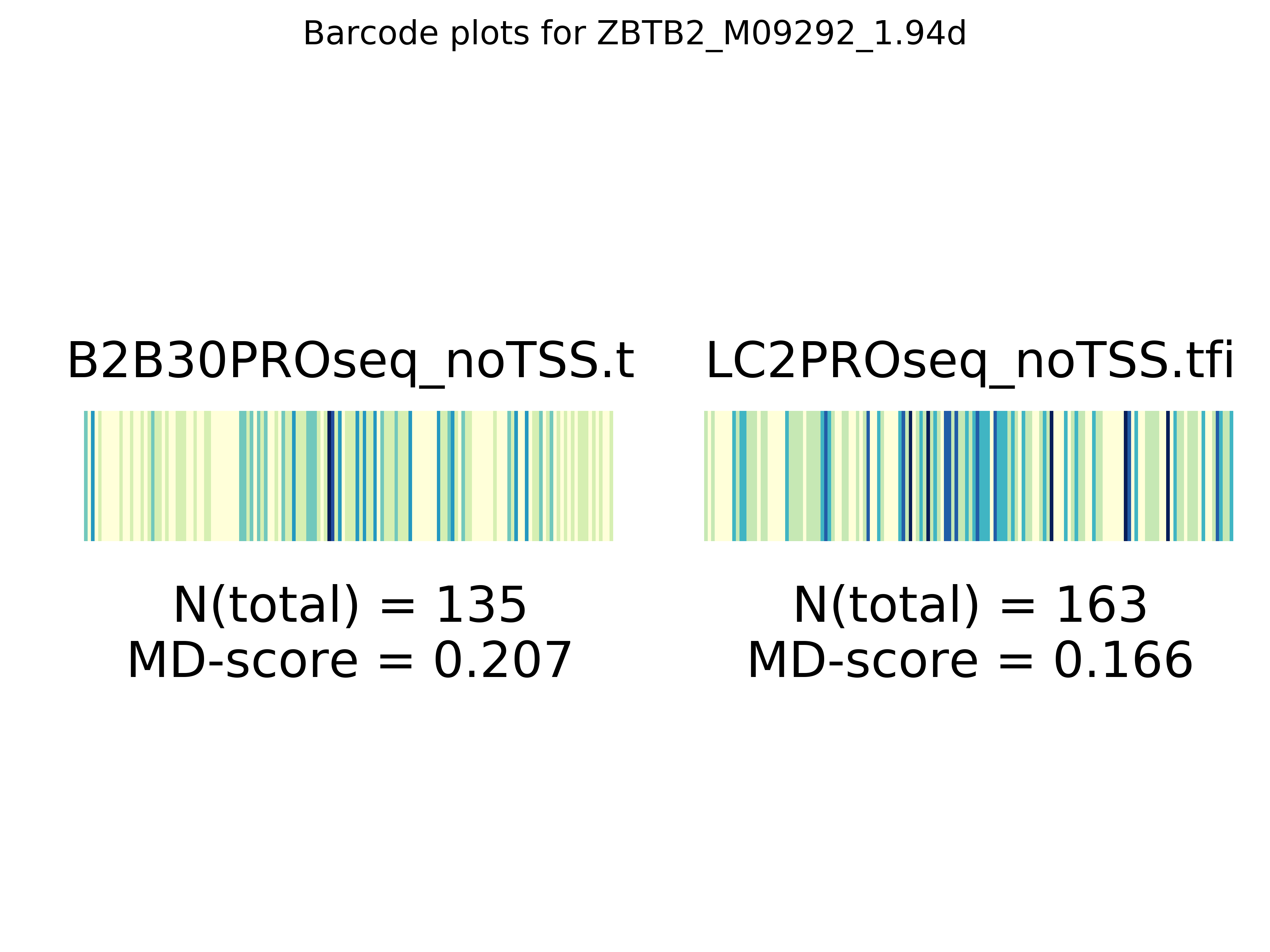

Supplement: Supplemental Data Set 1 [file jciinsight-6-144294-s076.zip › noTSS/best_curated_Human_TFs_p1e-5_grch38/B2B_vs_LC2/ZBTB2_M09292_1.94d_barcode_B2B30PROseq_noTSS.tfit_merged_vs_LC2PROseq_noTSS.tfit_merged.png]

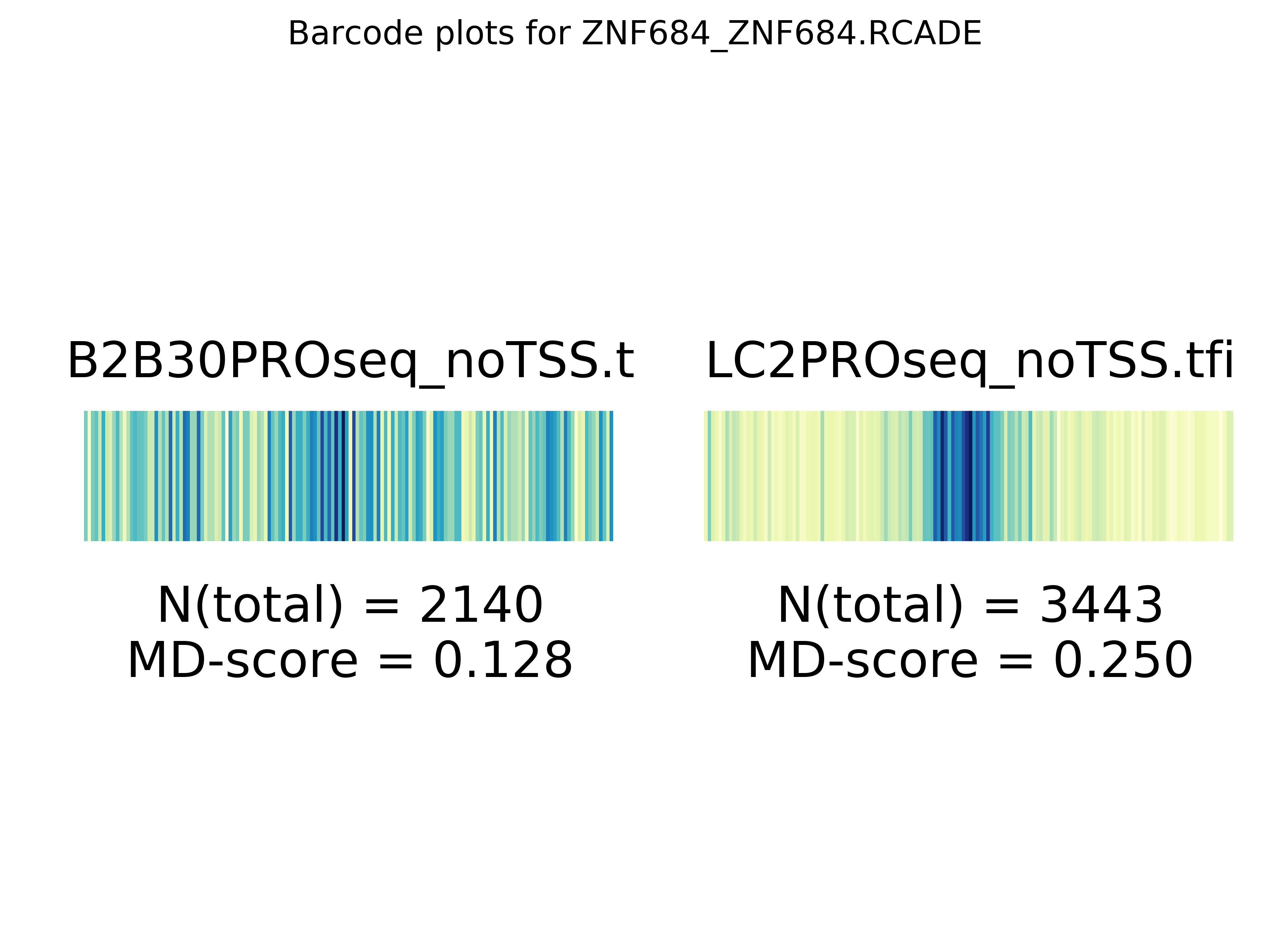

Supplement: Supplemental Data Set 1 [file jciinsight-6-144294-s076.zip › noTSS/best_curated_Human_TFs_p1e-5_grch38/B2B_vs_LC2/ZNF684_ZNF684.RCADE_barcode_B2B30PROseq_noTSS.tfit_merged_vs_LC2PROseq_noTSS.tfit_merged.png]

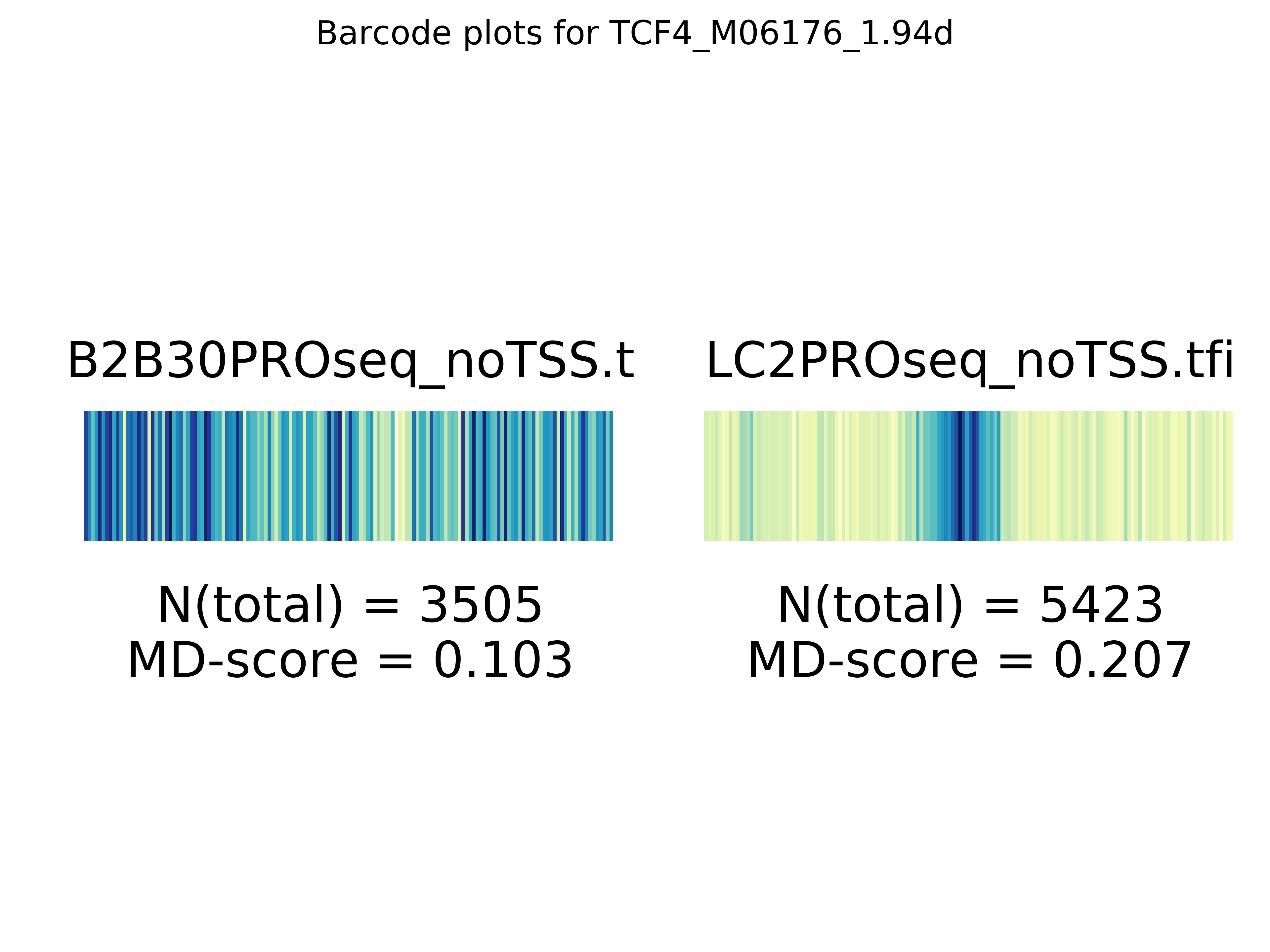

Supplement: Supplemental Data Set 1 [file jciinsight-6-144294-s076.zip › noTSS/best_curated_Human_TFs_p1e-5_grch38/B2B_vs_LC2/TCF4_M06176_1.94d_barcode_B2B30PROseq_noTSS.tfit_merged_vs_LC2PROseq_noTSS.tfit_merged.png]

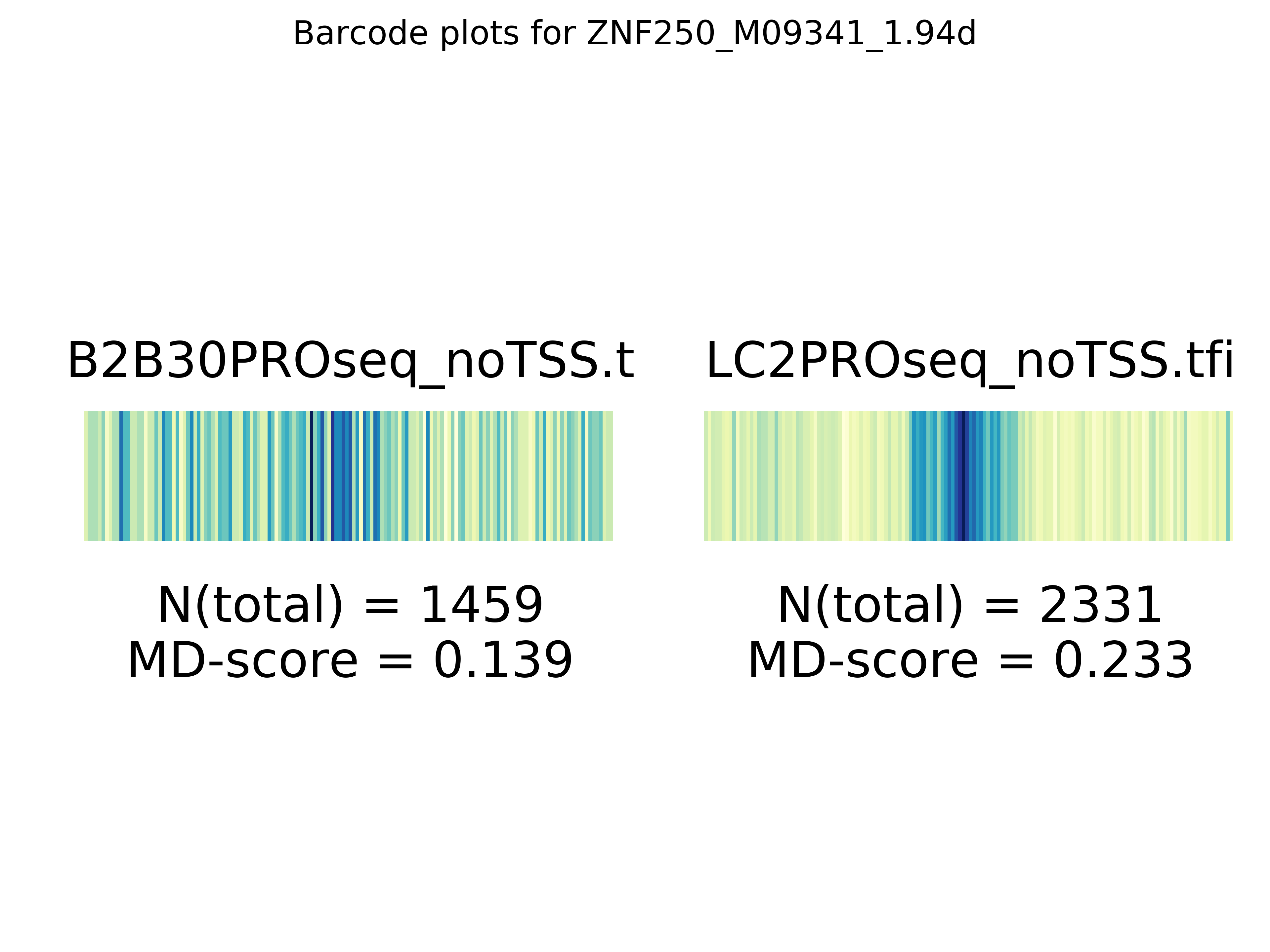

Supplement: Supplemental Data Set 1 [file jciinsight-6-144294-s076.zip › noTSS/best_curated_Human_TFs_p1e-5_grch38/B2B_vs_LC2/ZNF250_M09341_1.94d_barcode_B2B30PROseq_noTSS.tfit_merged_vs_LC2PROseq_noTSS.tfit_merged.png]

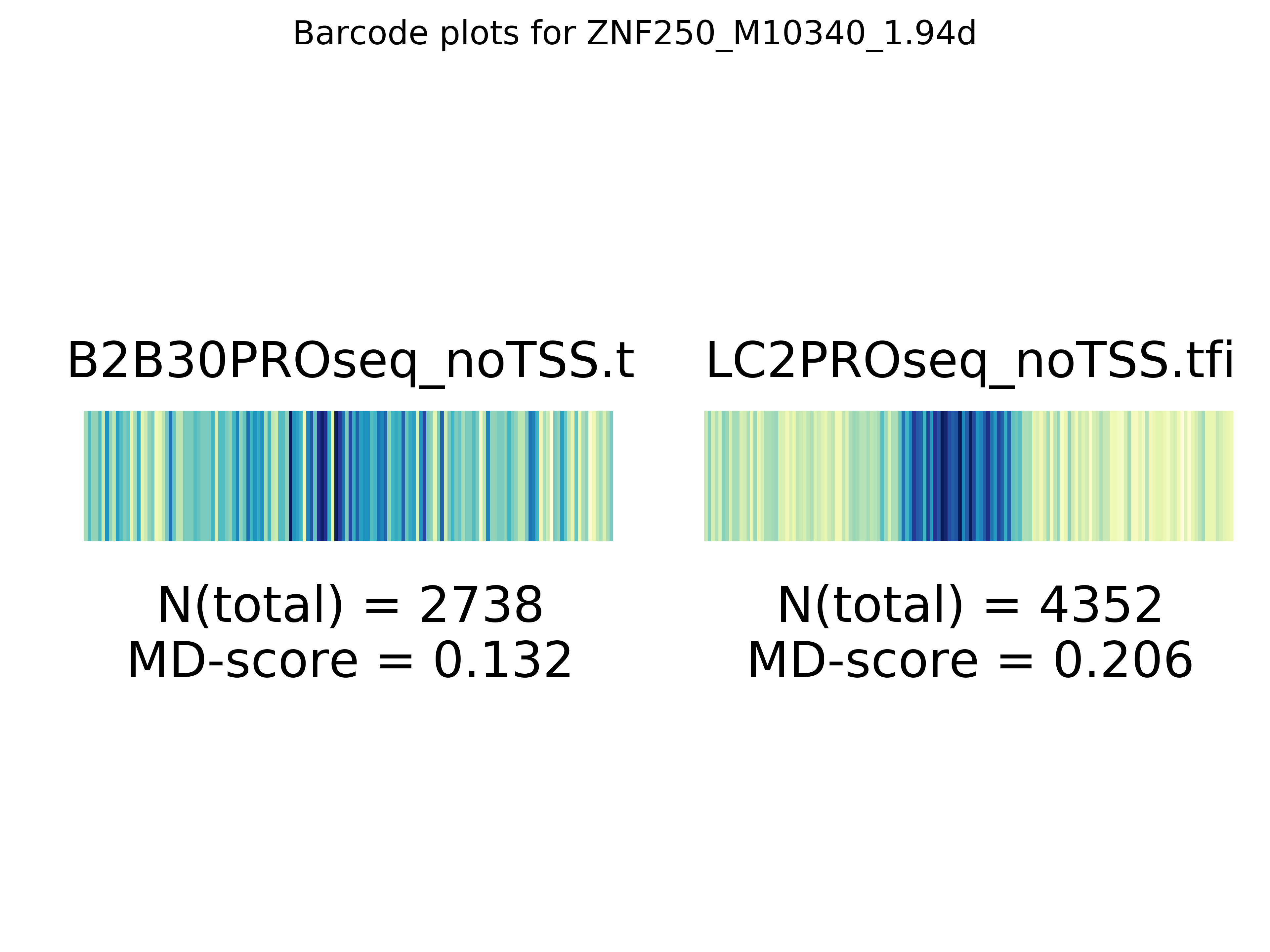

Supplement: Supplemental Data Set 1 [file jciinsight-6-144294-s076.zip › noTSS/best_curated_Human_TFs_p1e-5_grch38/B2B_vs_LC2/ZNF250_M10340_1.94d_barcode_B2B30PROseq_noTSS.tfit_merged_vs_LC2PROseq_noTSS.tfit_merged.png]

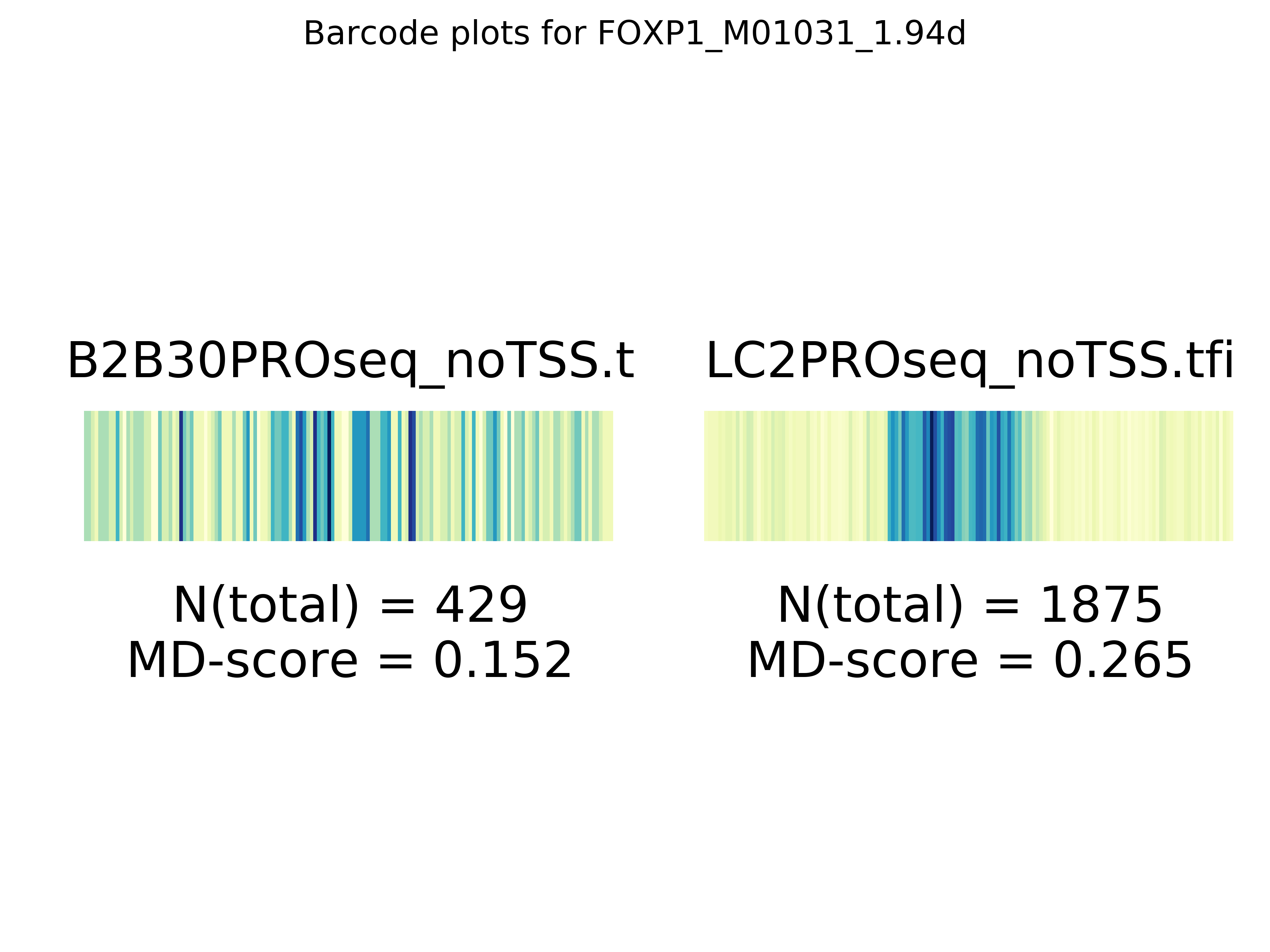

Supplement: Supplemental Data Set 1 [file jciinsight-6-144294-s076.zip › noTSS/best_curated_Human_TFs_p1e-5_grch38/B2B_vs_LC2/FOXP1_M01031_1.94d_barcode_B2B30PROseq_noTSS.tfit_merged_vs_LC2PROseq_noTSS.tfit_merged.png]

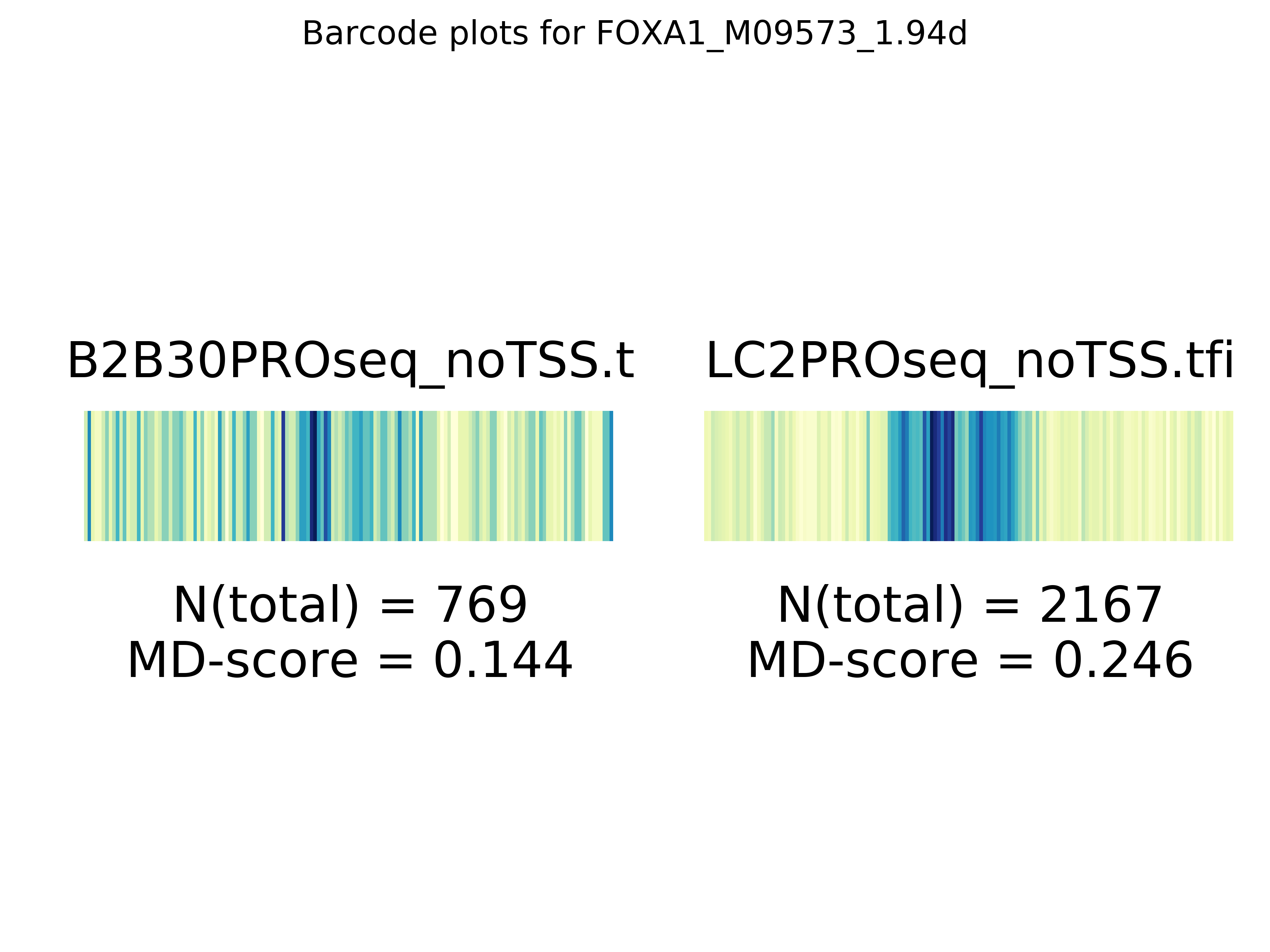

Supplement: Supplemental Data Set 1 [file jciinsight-6-144294-s076.zip › noTSS/best_curated_Human_TFs_p1e-5_grch38/B2B_vs_LC2/FOXA1_M09573_1.94d_barcode_B2B30PROseq_noTSS.tfit_merged_vs_LC2PROseq_noTSS.tfit_merged.png]

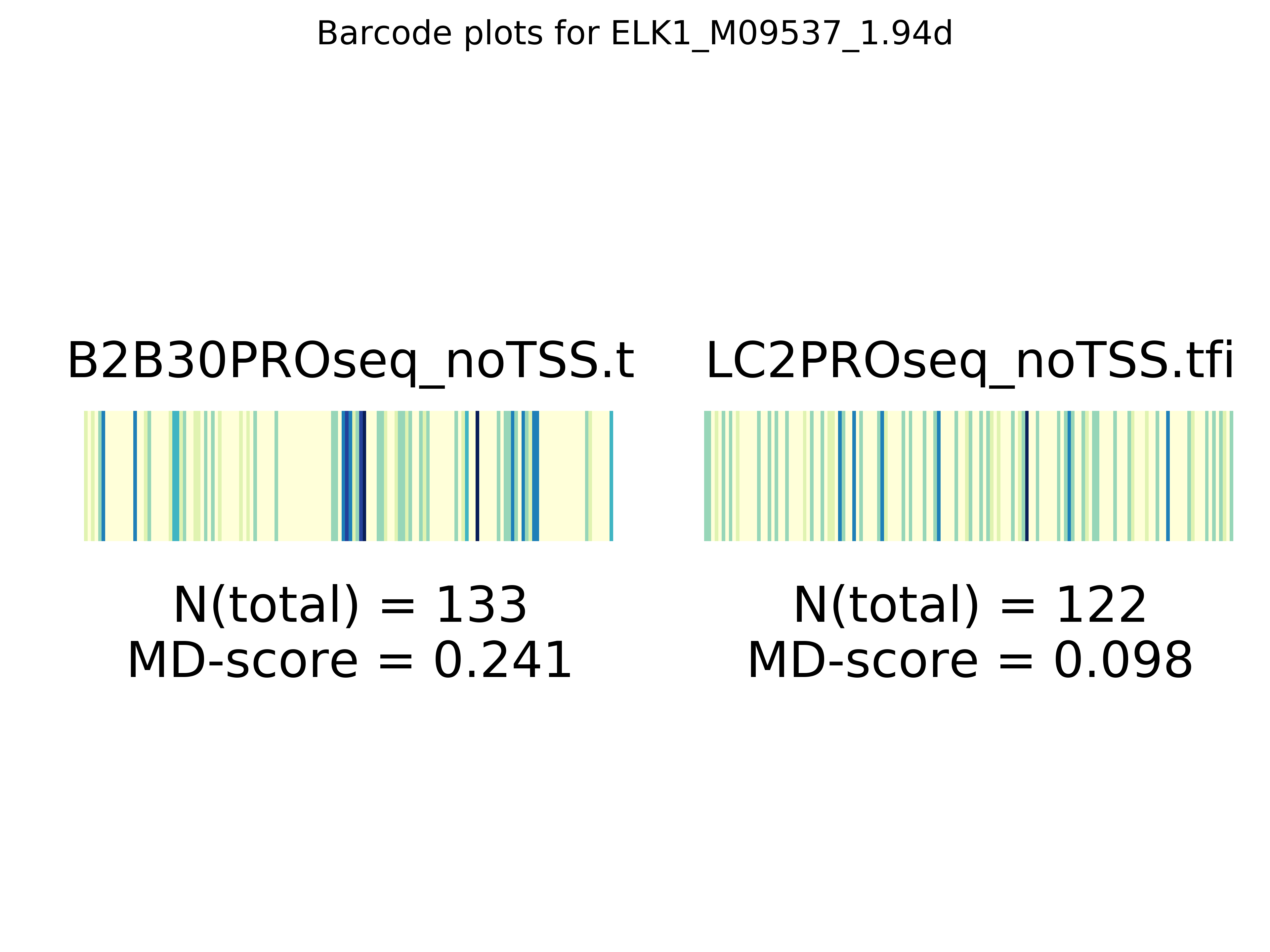

Supplement: Supplemental Data Set 1 [file jciinsight-6-144294-s076.zip › noTSS/best_curated_Human_TFs_p1e-5_grch38/B2B_vs_LC2/ELK1_M09537_1.94d_barcode_B2B30PROseq_noTSS.tfit_merged_vs_LC2PROseq_noTSS.tfit_merged.png]

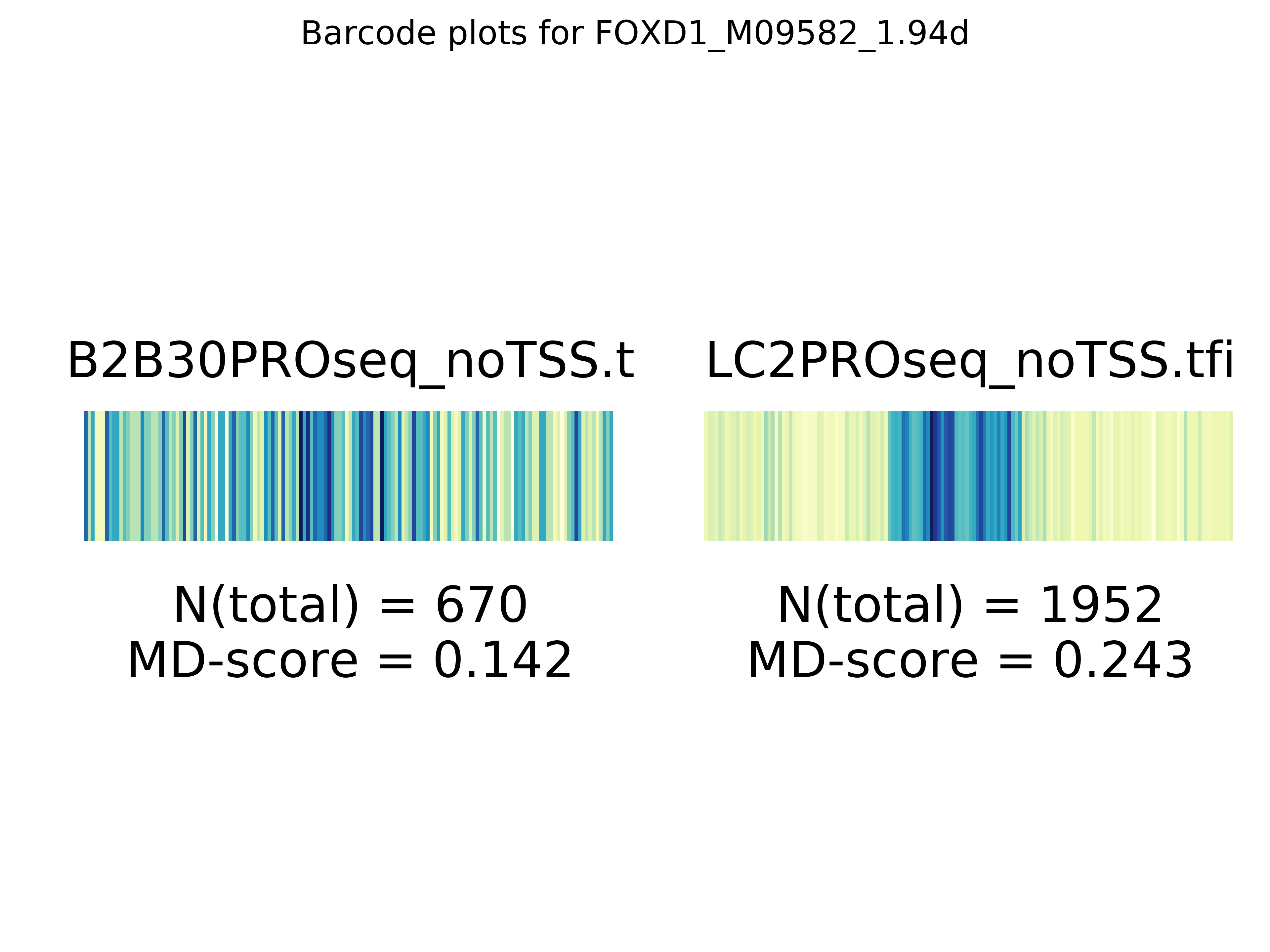

Supplement: Supplemental Data Set 1 [file jciinsight-6-144294-s076.zip › noTSS/best_curated_Human_TFs_p1e-5_grch38/B2B_vs_LC2/FOXD1_M09582_1.94d_barcode_B2B30PROseq_noTSS.tfit_merged_vs_LC2PROseq_noTSS.tfit_merged.png]

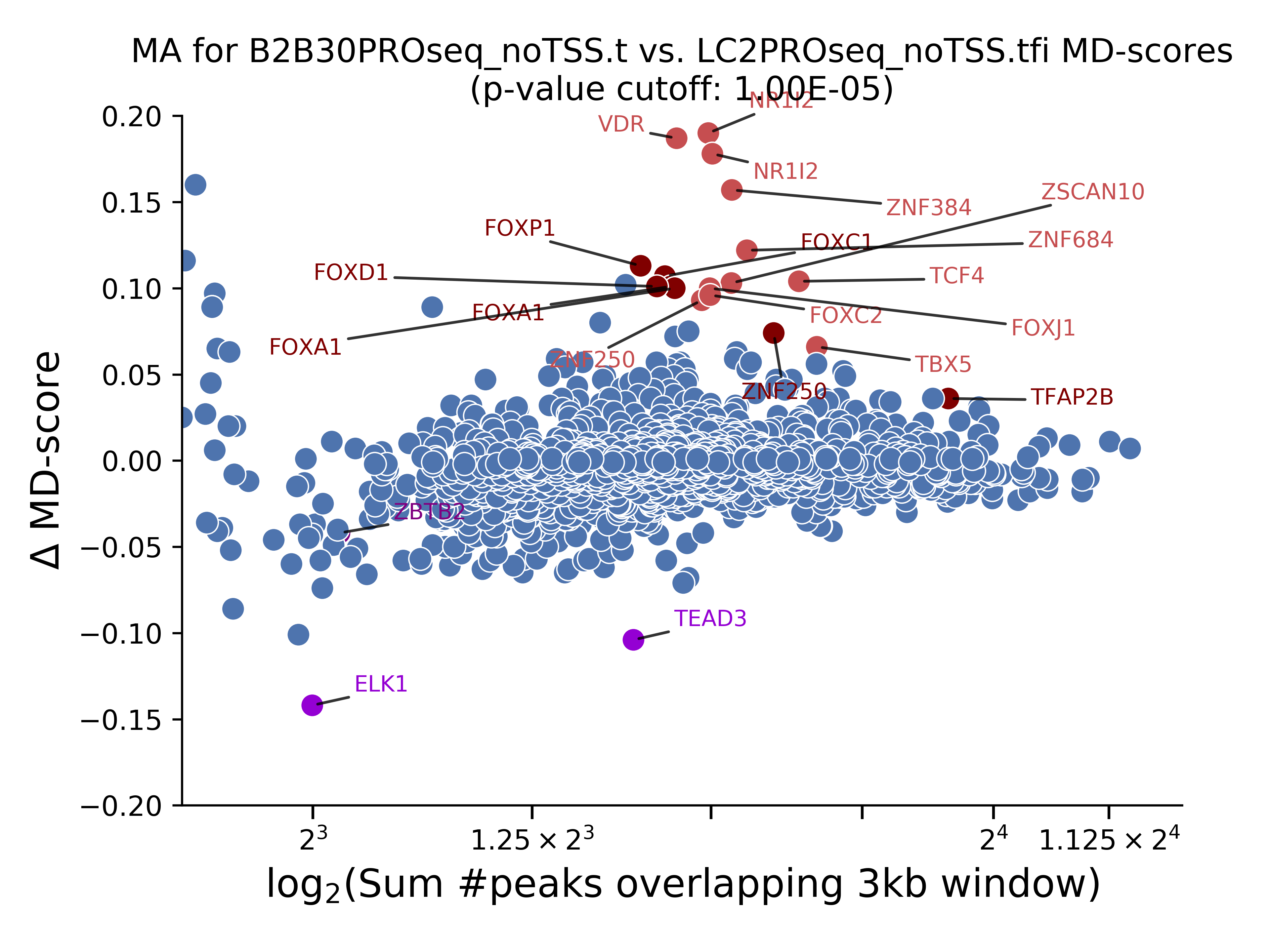

Supplement: Supplemental Data Set 1 [file jciinsight-6-144294-s076.zip › noTSS/best_curated_Human_TFs_p1e-5_grch38/B2B_vs_LC2/MA_B2B30PROseq_noTSS.tfit_merged_to_LC2PROseq_noTSS.tfit_merged_md_score.png]

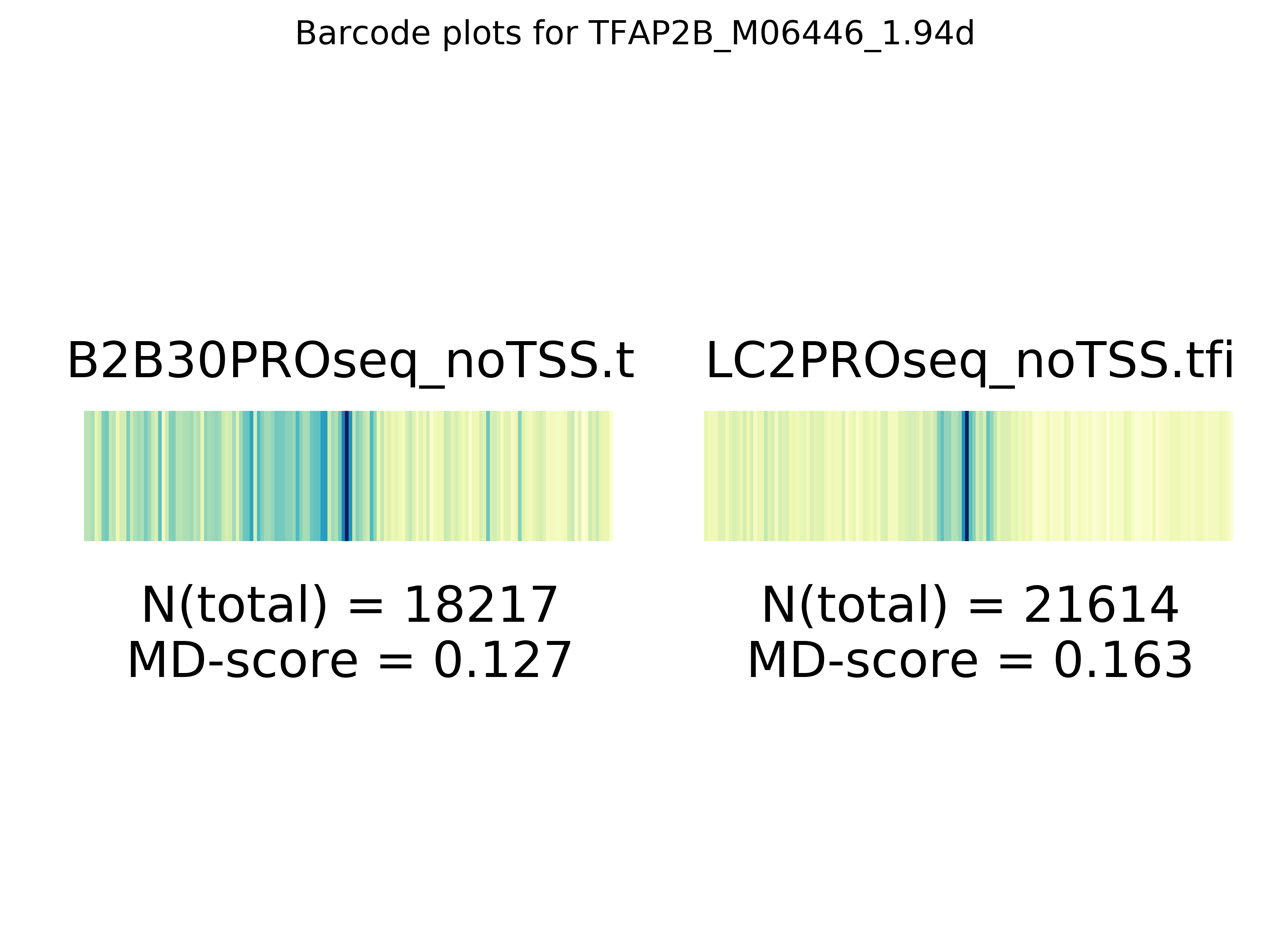

Supplement: Supplemental Data Set 1 [file jciinsight-6-144294-s076.zip › noTSS/best_curated_Human_TFs_p1e-5_grch38/B2B_vs_LC2/TFAP2B_M06446_1.94d_barcode_B2B30PROseq_noTSS.tfit_merged_vs_LC2PROseq_noTSS.tfit_merged.png]

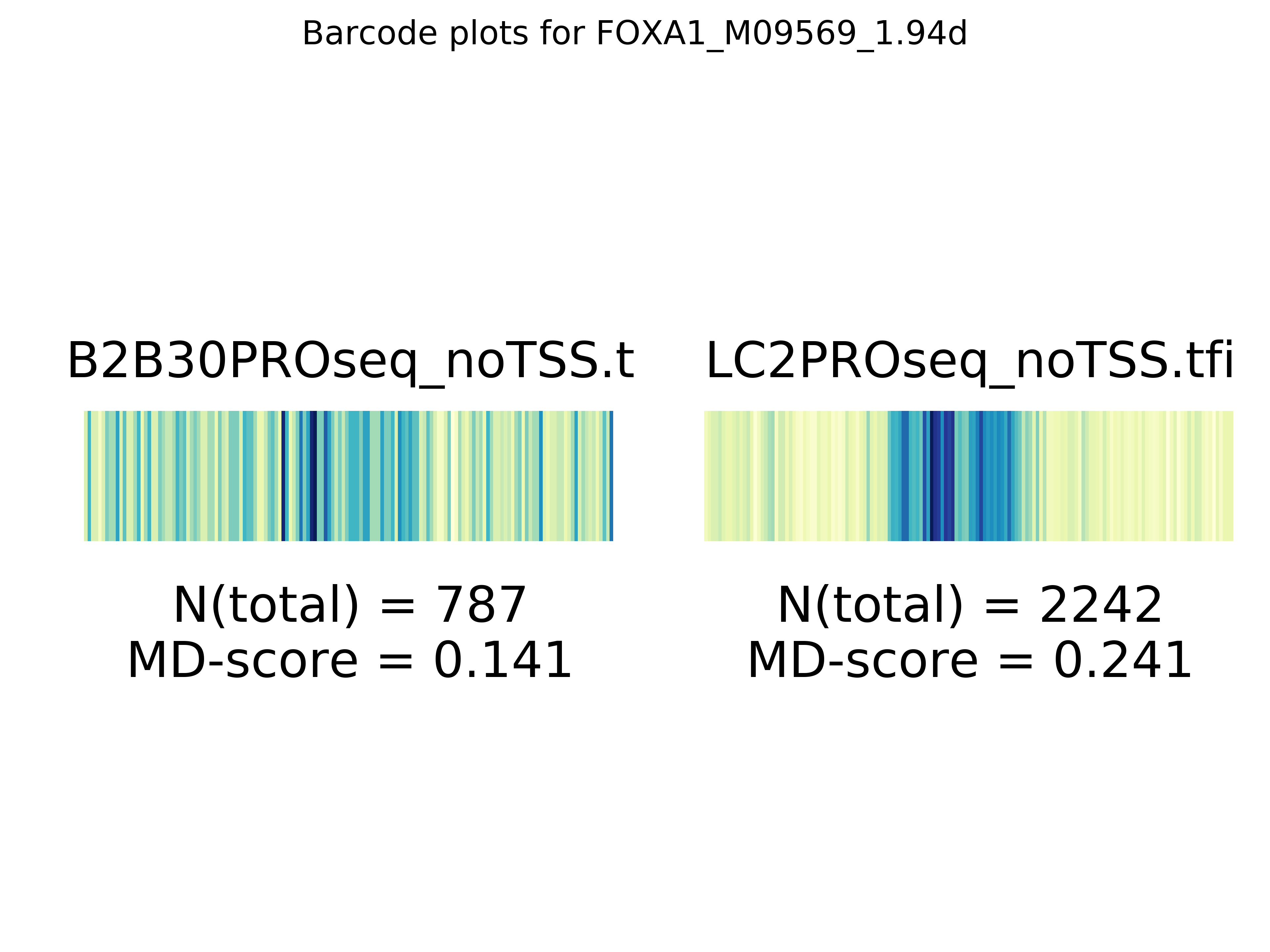

Supplement: Supplemental Data Set 1 [file jciinsight-6-144294-s076.zip › noTSS/best_curated_Human_TFs_p1e-5_grch38/B2B_vs_LC2/FOXA1_M09569_1.94d_barcode_B2B30PROseq_noTSS.tfit_merged_vs_LC2PROseq_noTSS.tfit_merged.png]

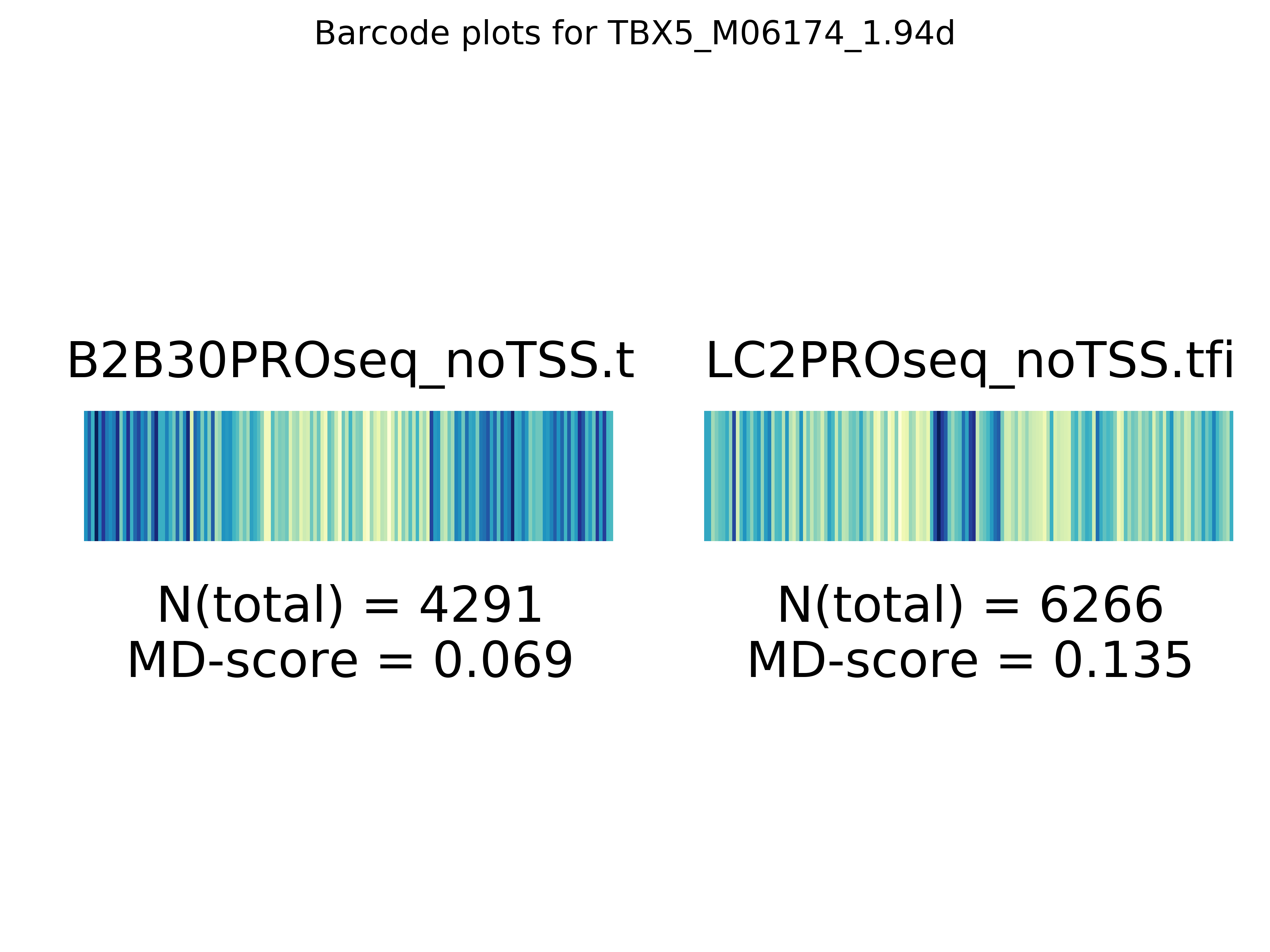

Supplement: Supplemental Data Set 1 [file jciinsight-6-144294-s076.zip › noTSS/best_curated_Human_TFs_p1e-5_grch38/B2B_vs_LC2/TBX5_M06174_1.94d_barcode_B2B30PROseq_noTSS.tfit_merged_vs_LC2PROseq_noTSS.tfit_merged.png]

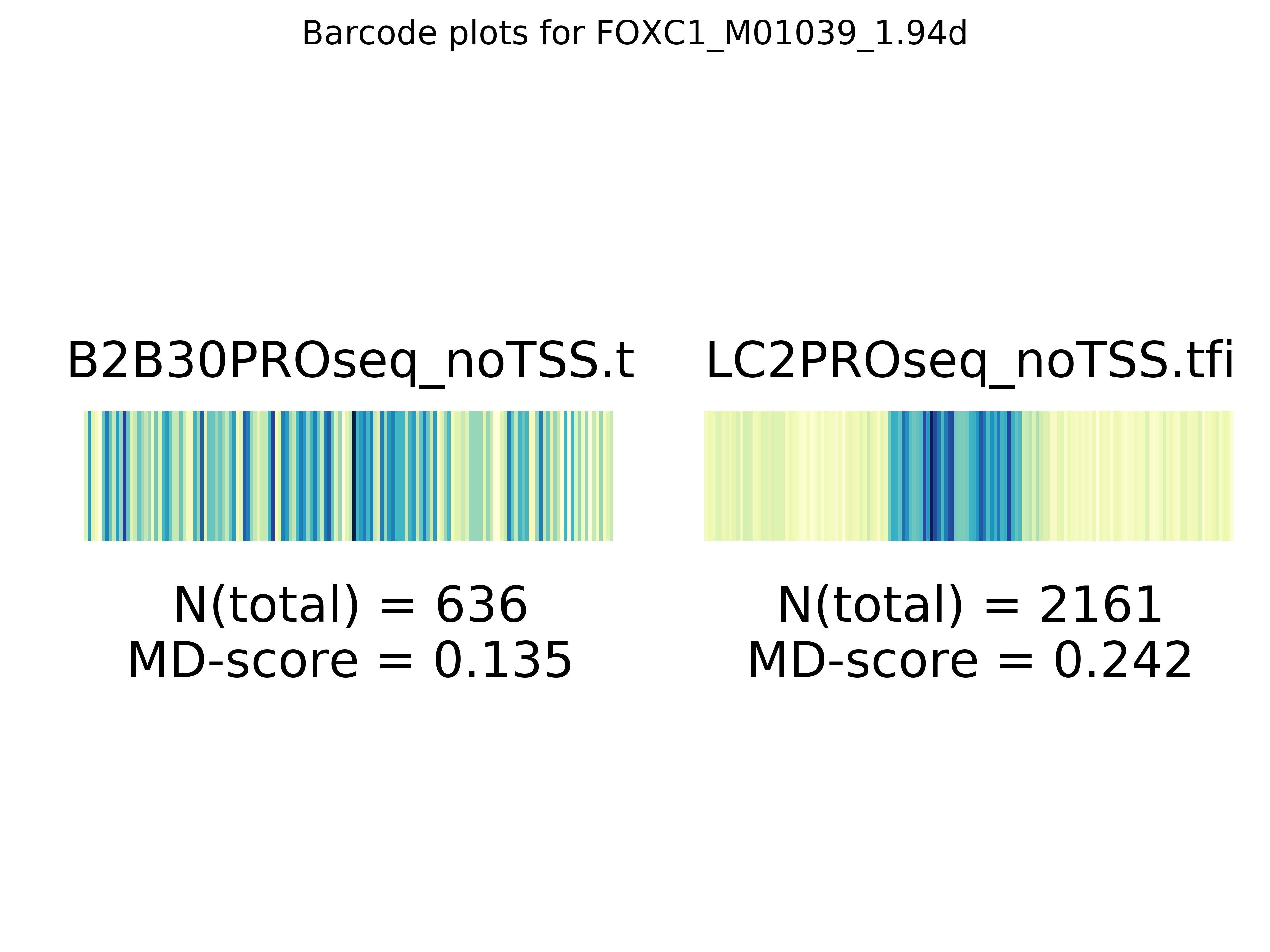

Supplement: Supplemental Data Set 1 [file jciinsight-6-144294-s076.zip › noTSS/best_curated_Human_TFs_p1e-5_grch38/B2B_vs_LC2/FOXC1_M01039_1.94d_barcode_B2B30PROseq_noTSS.tfit_merged_vs_LC2PROseq_noTSS.tfit_merged.png]

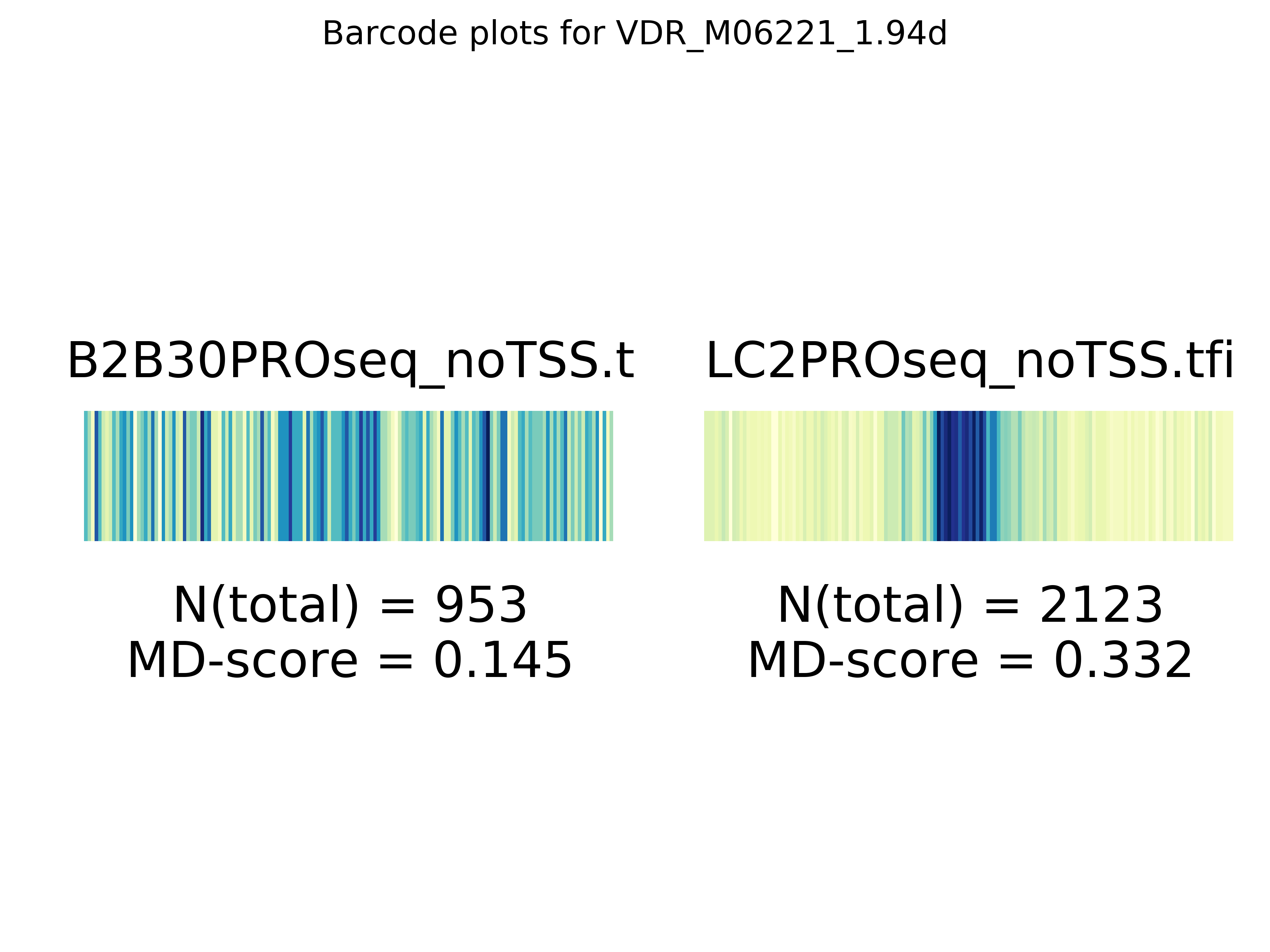

Supplement: Supplemental Data Set 1 [file jciinsight-6-144294-s076.zip › noTSS/best_curated_Human_TFs_p1e-5_grch38/B2B_vs_LC2/VDR_M06221_1.94d_barcode_B2B30PROseq_noTSS.tfit_merged_vs_LC2PROseq_noTSS.tfit_merged.png]

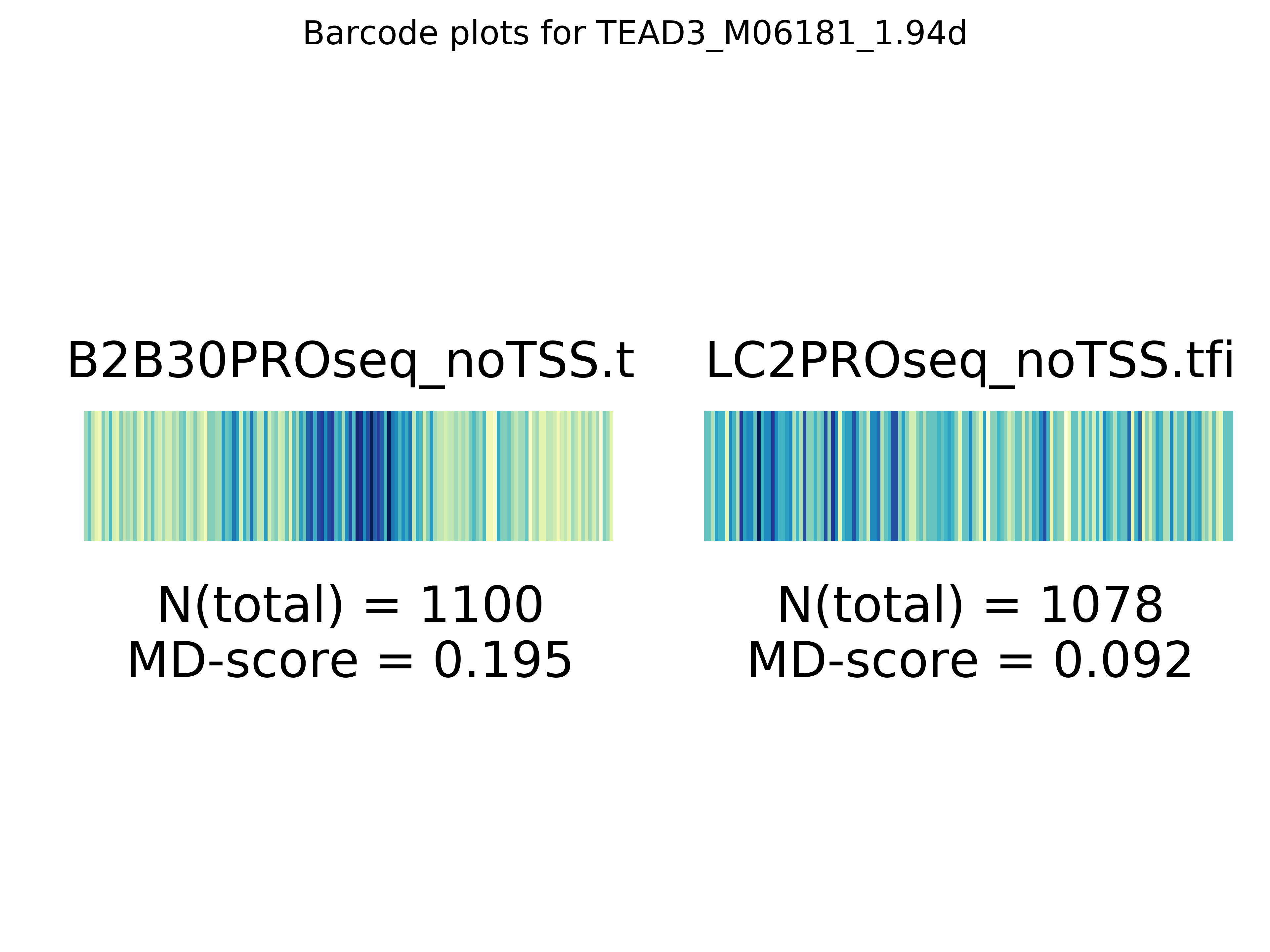

Supplement: Supplemental Data Set 1 [file jciinsight-6-144294-s076.zip › noTSS/best_curated_Human_TFs_p1e-5_grch38/B2B_vs_LC2/TEAD3_M06181_1.94d_barcode_B2B30PROseq_noTSS.tfit_merged_vs_LC2PROseq_noTSS.tfit_merged.png]

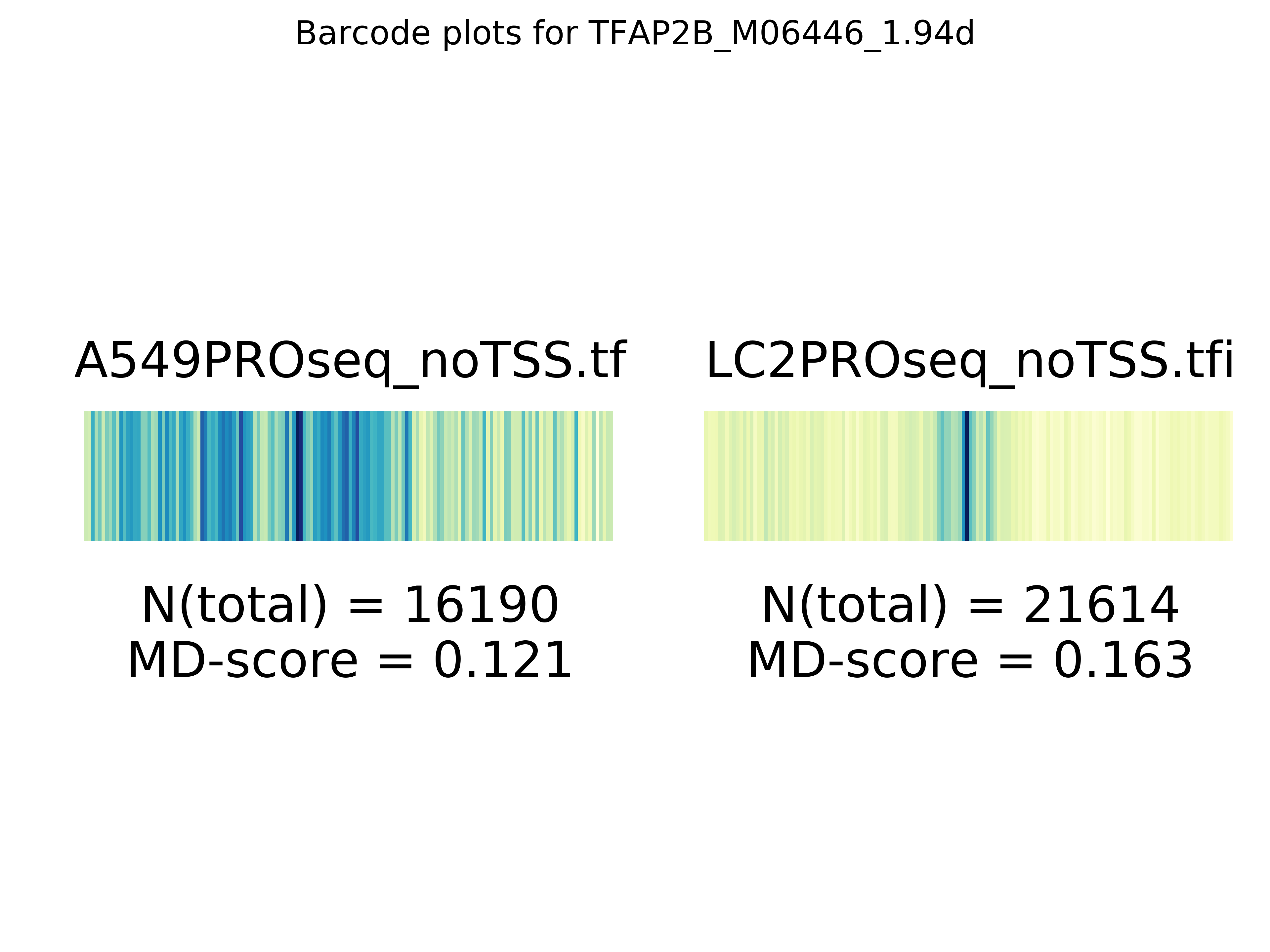

Supplement: Supplemental Data Set 1 [file jciinsight-6-144294-s076.zip › noTSS/best_curated_Human_TFs_p1e-5_grch38/A549_vs_LC2/TFAP2B_M06446_1.94d_barcode_A549PROseq_noTSS.tfit_merged_vs_LC2PROseq_noTSS.tfit_merged.png]

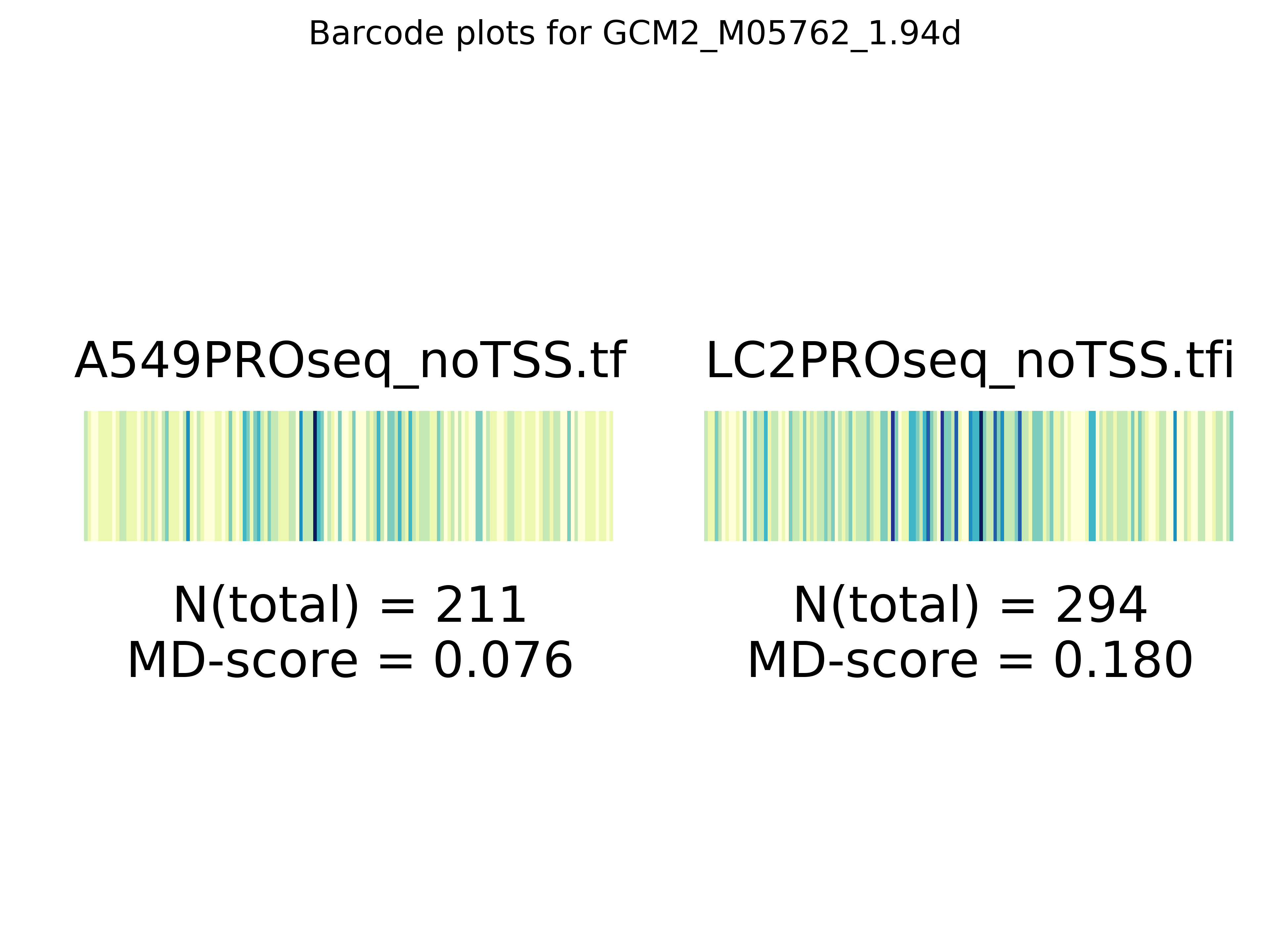

Supplement: Supplemental Data Set 1 [file jciinsight-6-144294-s076.zip › noTSS/best_curated_Human_TFs_p1e-5_grch38/A549_vs_LC2/GCM2_M05762_1.94d_barcode_A549PROseq_noTSS.tfit_merged_vs_LC2PROseq_noTSS.tfit_merged.png]

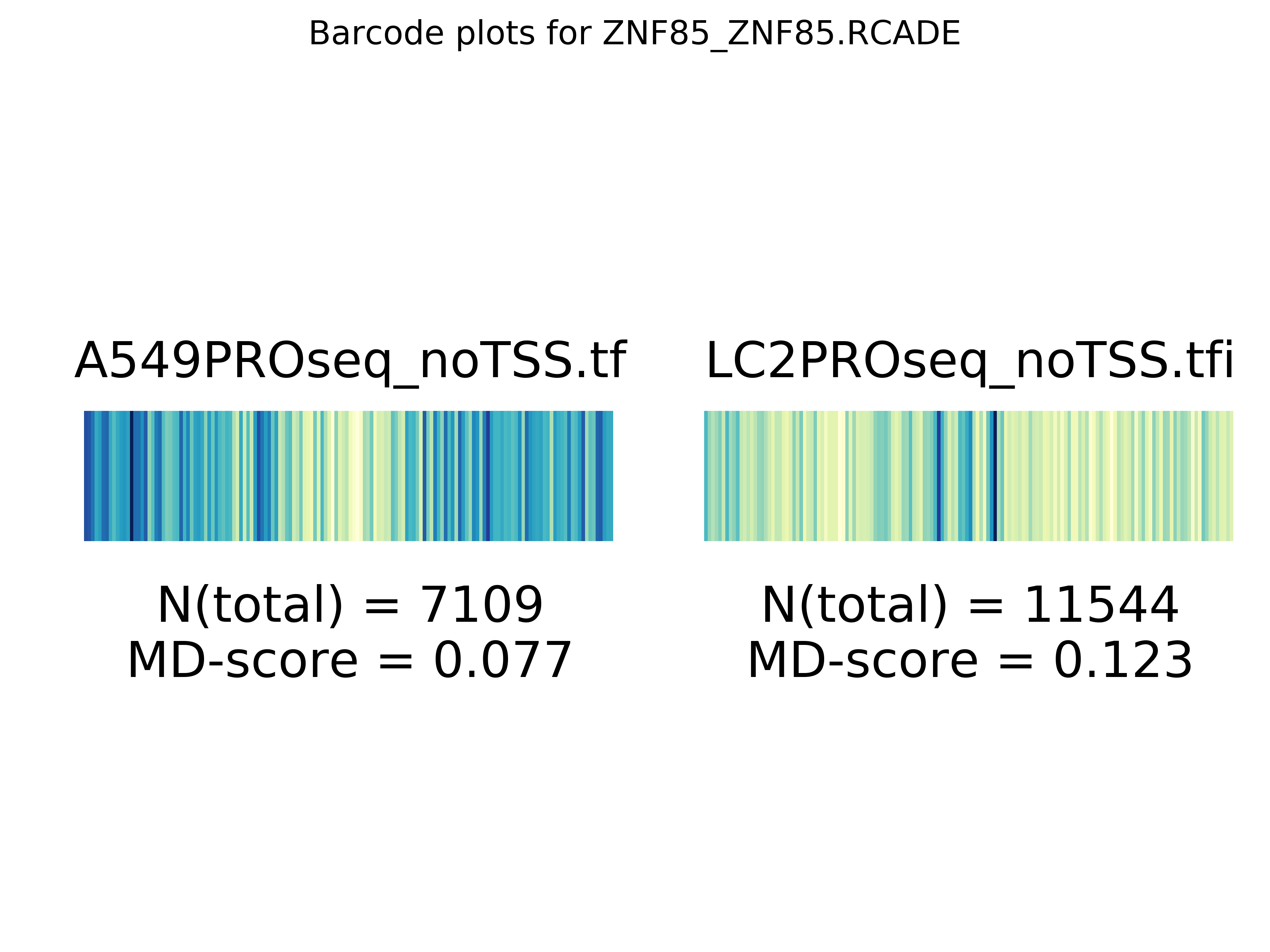

Supplement: Supplemental Data Set 1 [file jciinsight-6-144294-s076.zip › noTSS/best_curated_Human_TFs_p1e-5_grch38/A549_vs_LC2/ZNF85_ZNF85.RCADE_barcode_A549PROseq_noTSS.tfit_merged_vs_LC2PROseq_noTSS.tfit_merged.png]

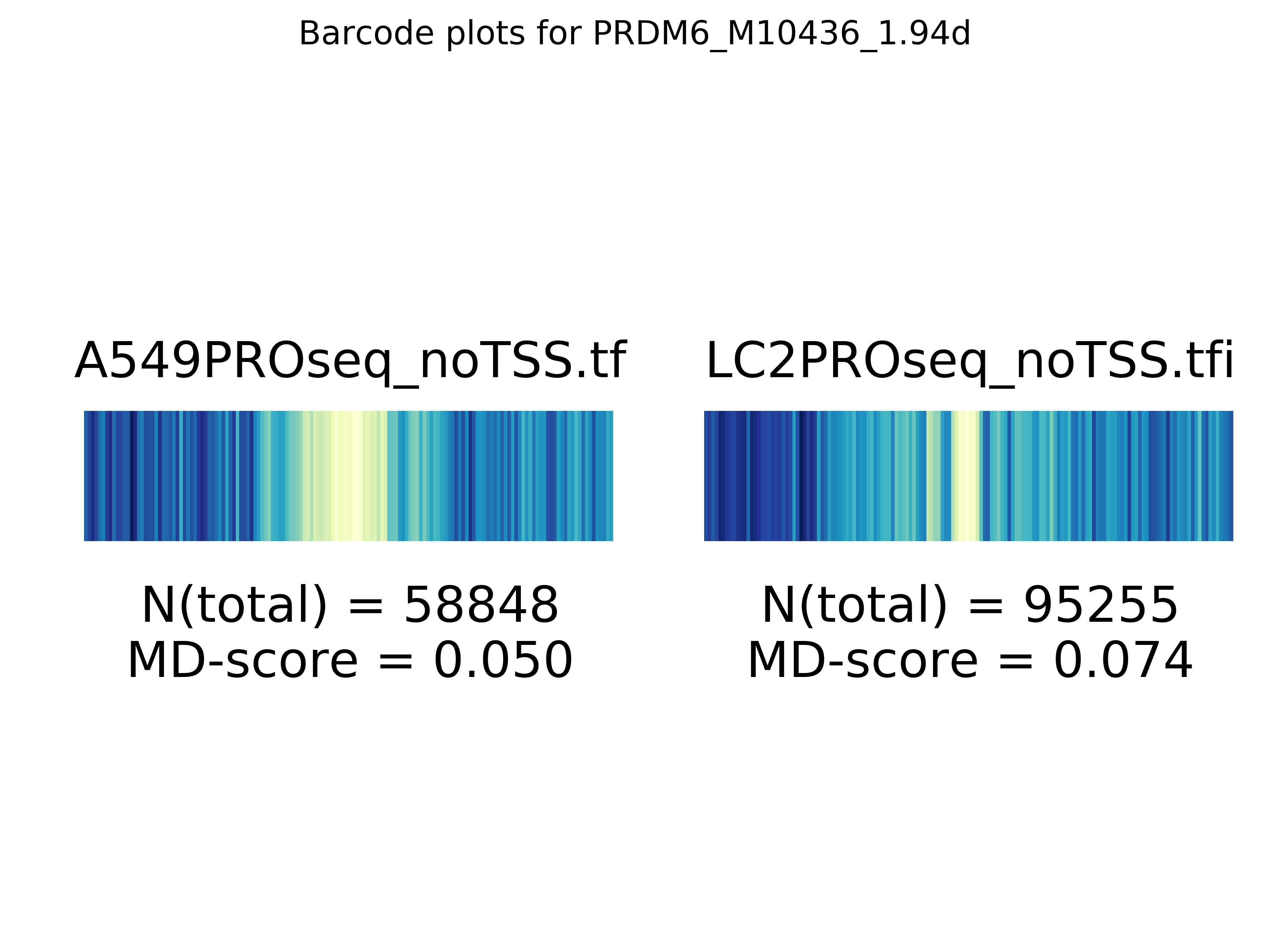

Supplement: Supplemental Data Set 1 [file jciinsight-6-144294-s076.zip › noTSS/best_curated_Human_TFs_p1e-5_grch38/A549_vs_LC2/PRDM6_M10436_1.94d_barcode_A549PROseq_noTSS.tfit_merged_vs_LC2PROseq_noTSS.tfit_merged.png]

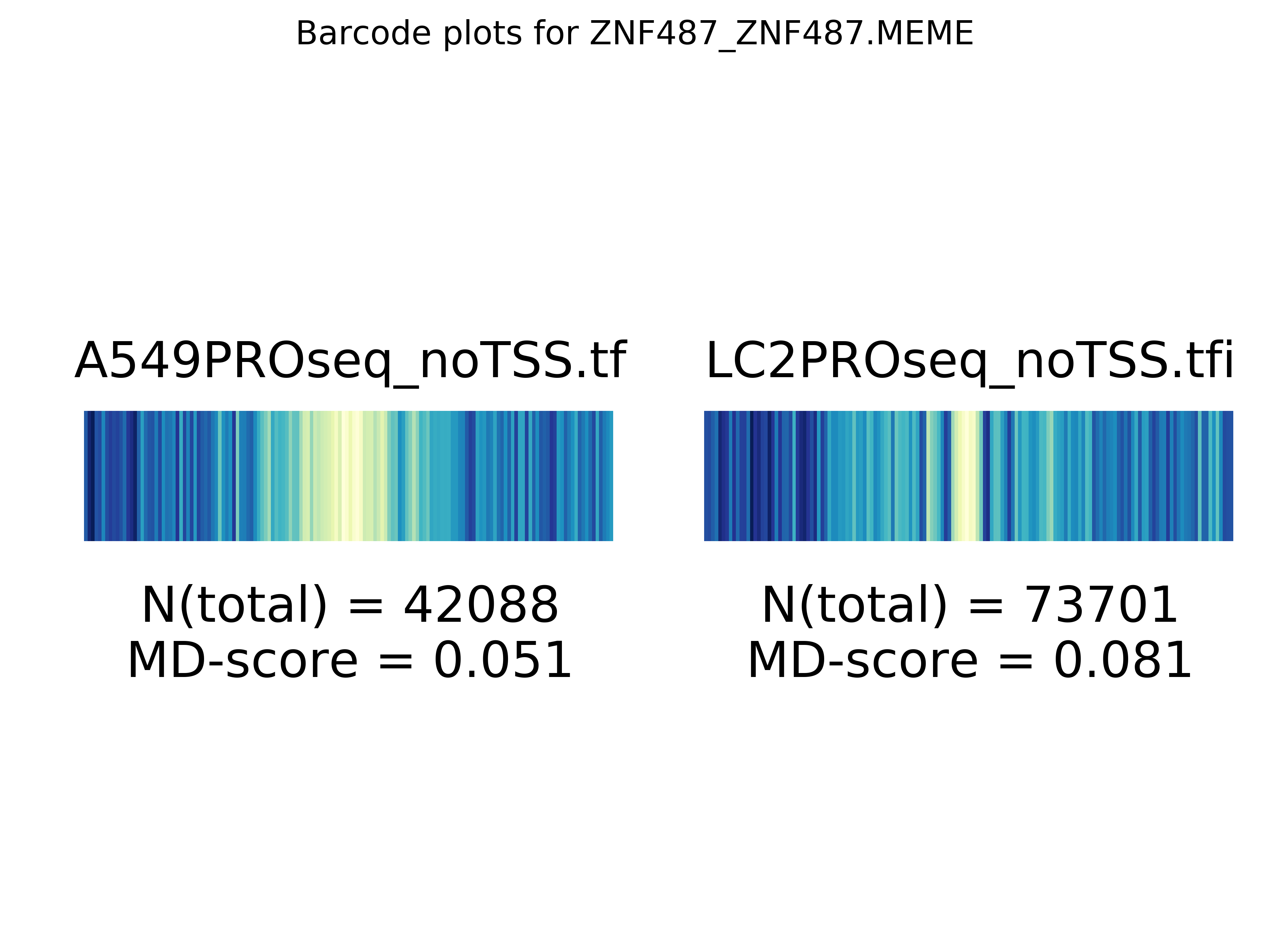

Supplement: Supplemental Data Set 1 [file jciinsight-6-144294-s076.zip › noTSS/best_curated_Human_TFs_p1e-5_grch38/A549_vs_LC2/ZNF487_ZNF487.MEME_barcode_A549PROseq_noTSS.tfit_merged_vs_LC2PROseq_noTSS.tfit_merged.png]

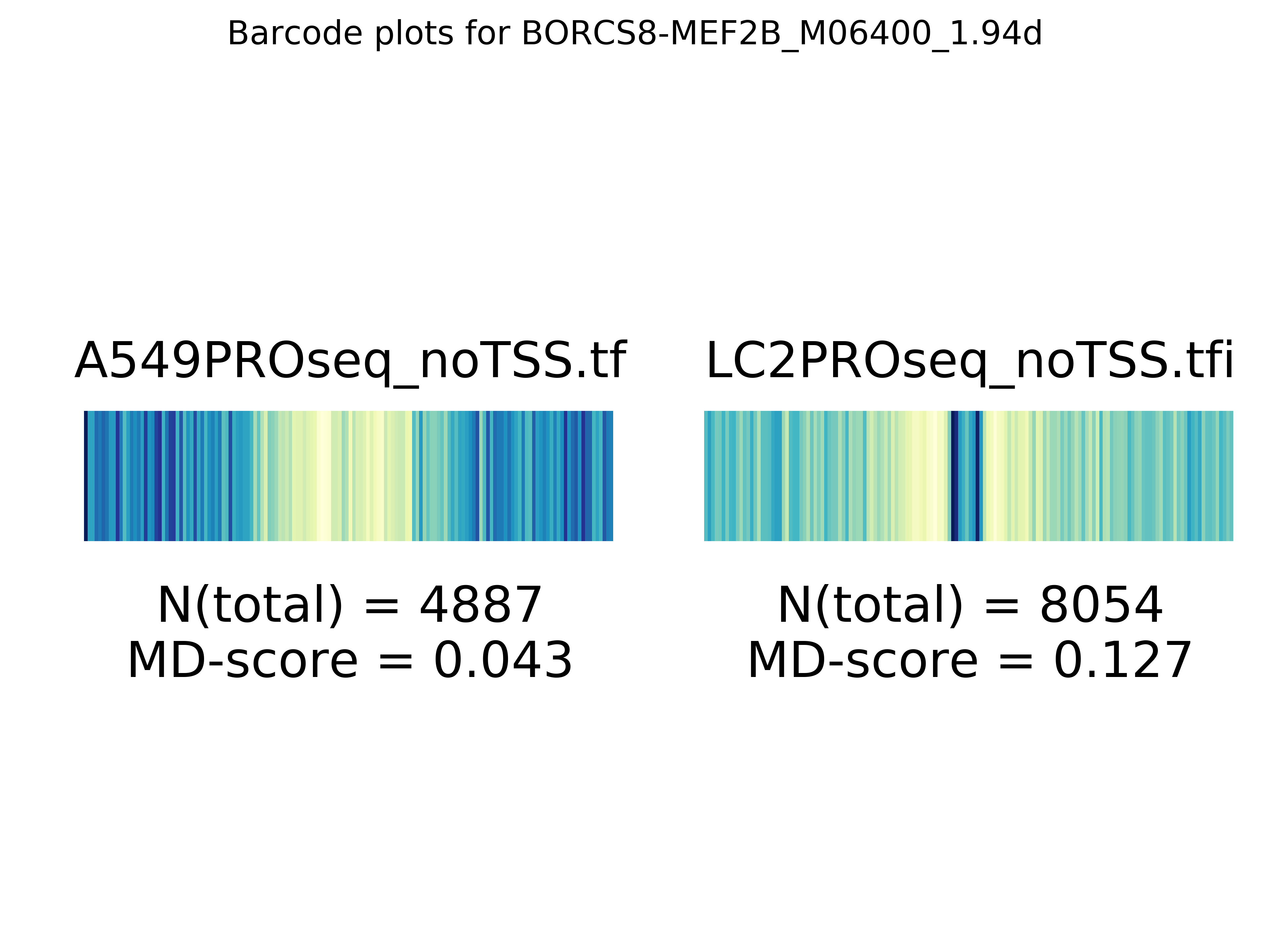

Supplement: Supplemental Data Set 1 [file jciinsight-6-144294-s076.zip › noTSS/best_curated_Human_TFs_p1e-5_grch38/A549_vs_LC2/BORCS8-MEF2B_M06400_1.94d_barcode_A549PROseq_noTSS.tfit_merged_vs_LC2PROseq_noTSS.tfit_merged.png]

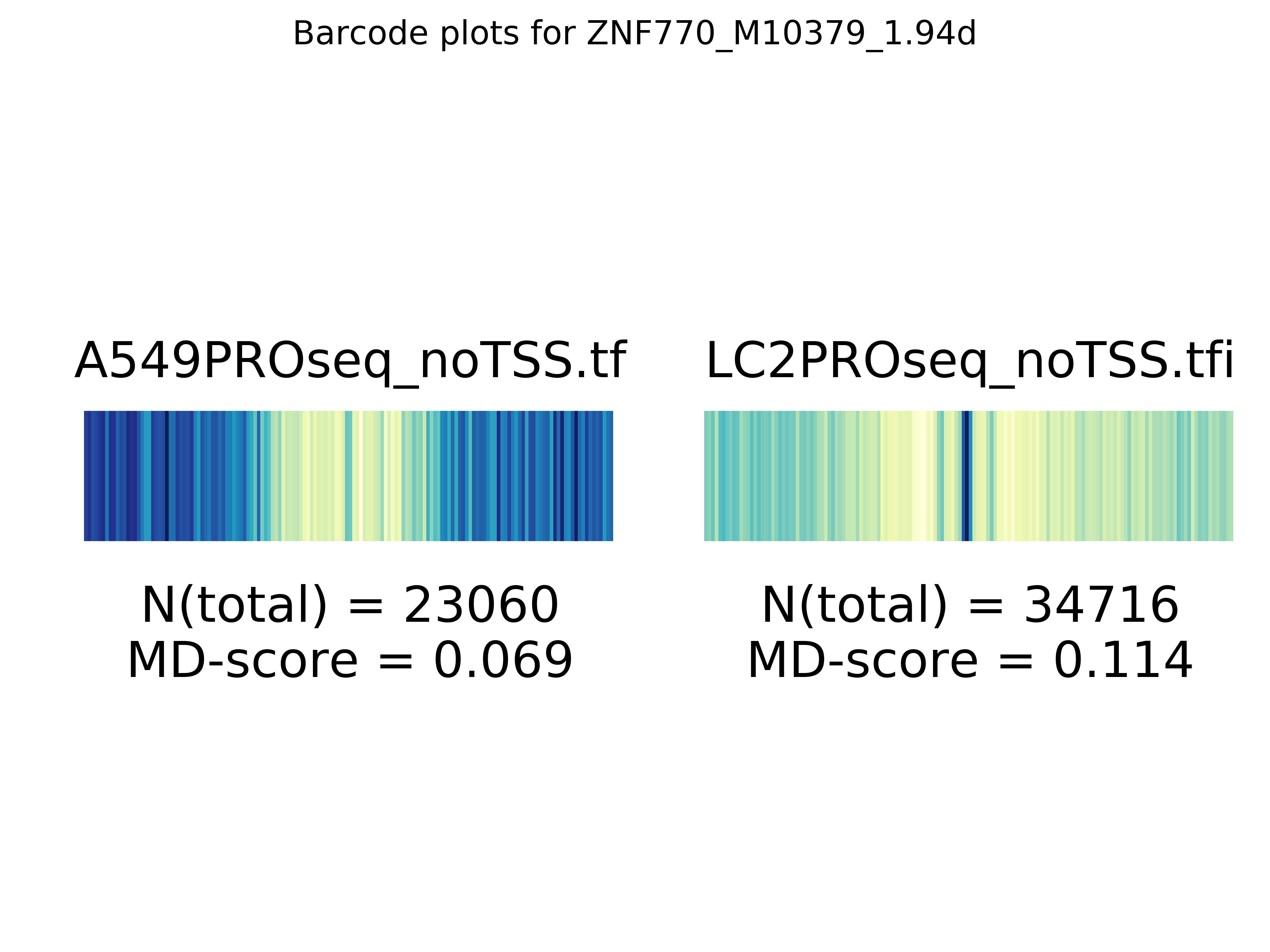

Supplement: Supplemental Data Set 1 [file jciinsight-6-144294-s076.zip › noTSS/best_curated_Human_TFs_p1e-5_grch38/A549_vs_LC2/ZNF770_M10379_1.94d_barcode_A549PROseq_noTSS.tfit_merged_vs_LC2PROseq_noTSS.tfit_merged.png]

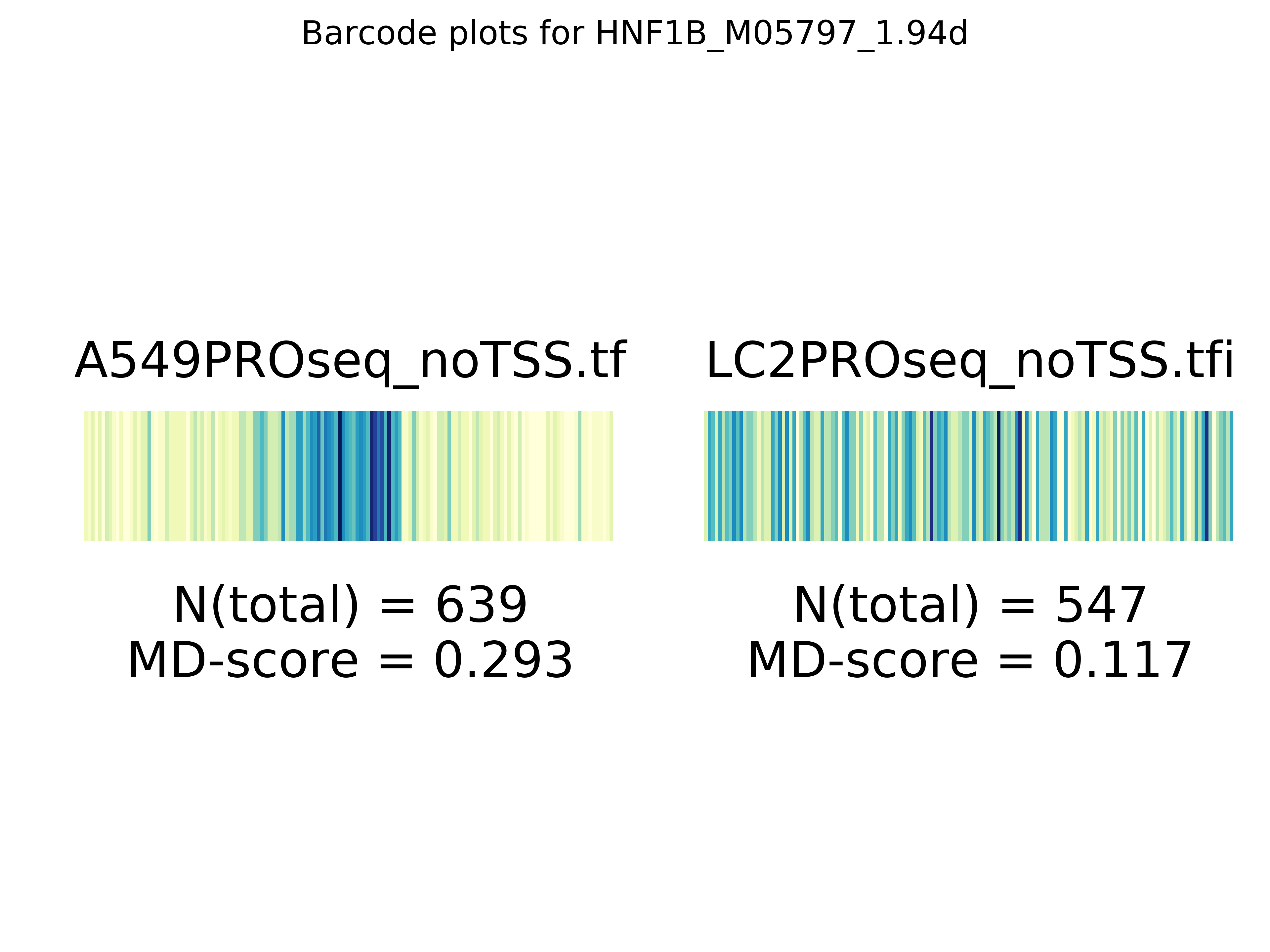

Supplement: Supplemental Data Set 1 [file jciinsight-6-144294-s076.zip › noTSS/best_curated_Human_TFs_p1e-5_grch38/A549_vs_LC2/HNF1B_M05797_1.94d_barcode_A549PROseq_noTSS.tfit_merged_vs_LC2PROseq_noTSS.tfit_merged.png]

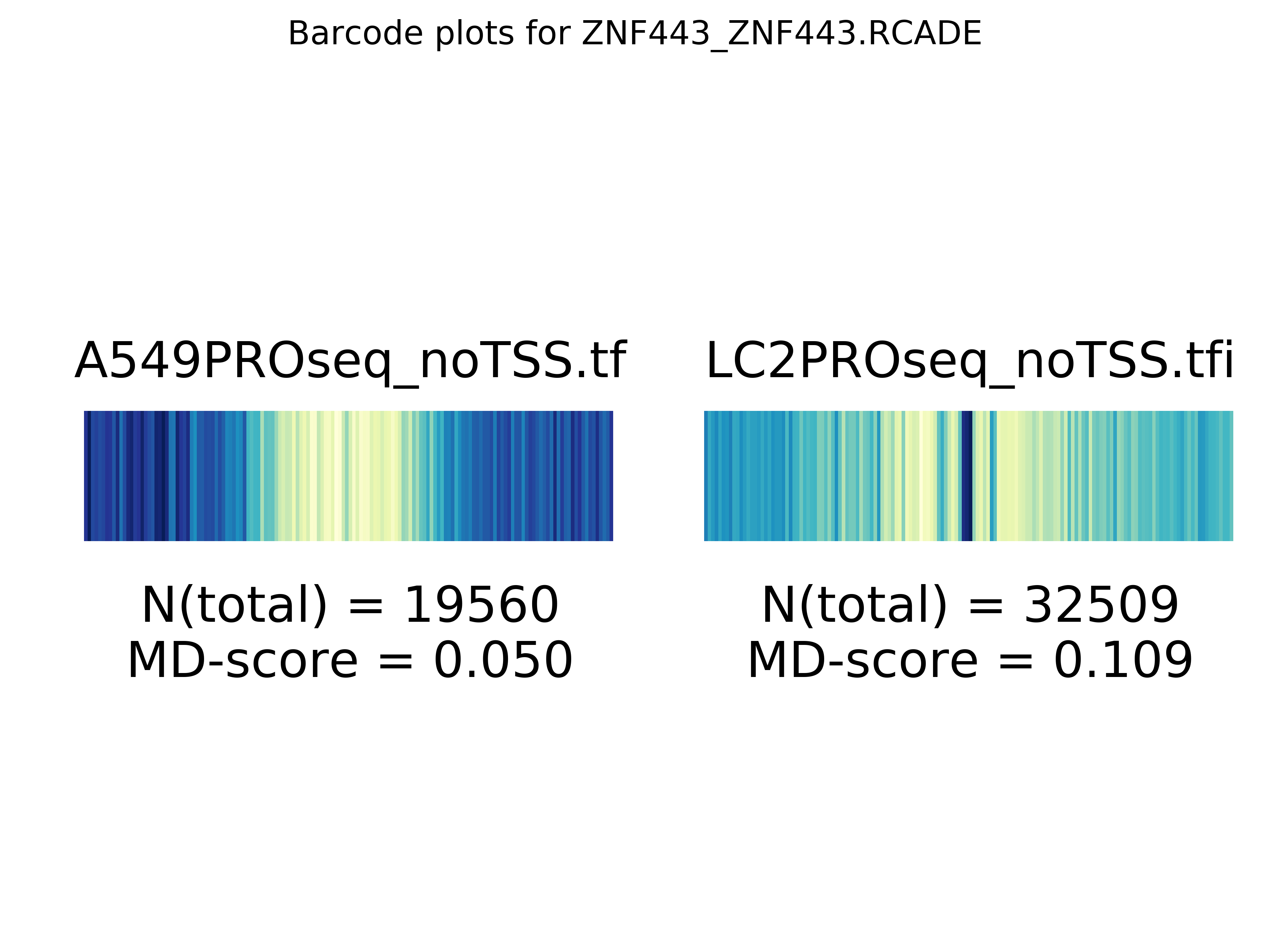

Supplement: Supplemental Data Set 1 [file jciinsight-6-144294-s076.zip › noTSS/best_curated_Human_TFs_p1e-5_grch38/A549_vs_LC2/ZNF443_ZNF443.RCADE_barcode_A549PROseq_noTSS.tfit_merged_vs_LC2PROseq_noTSS.tfit_merged.png]

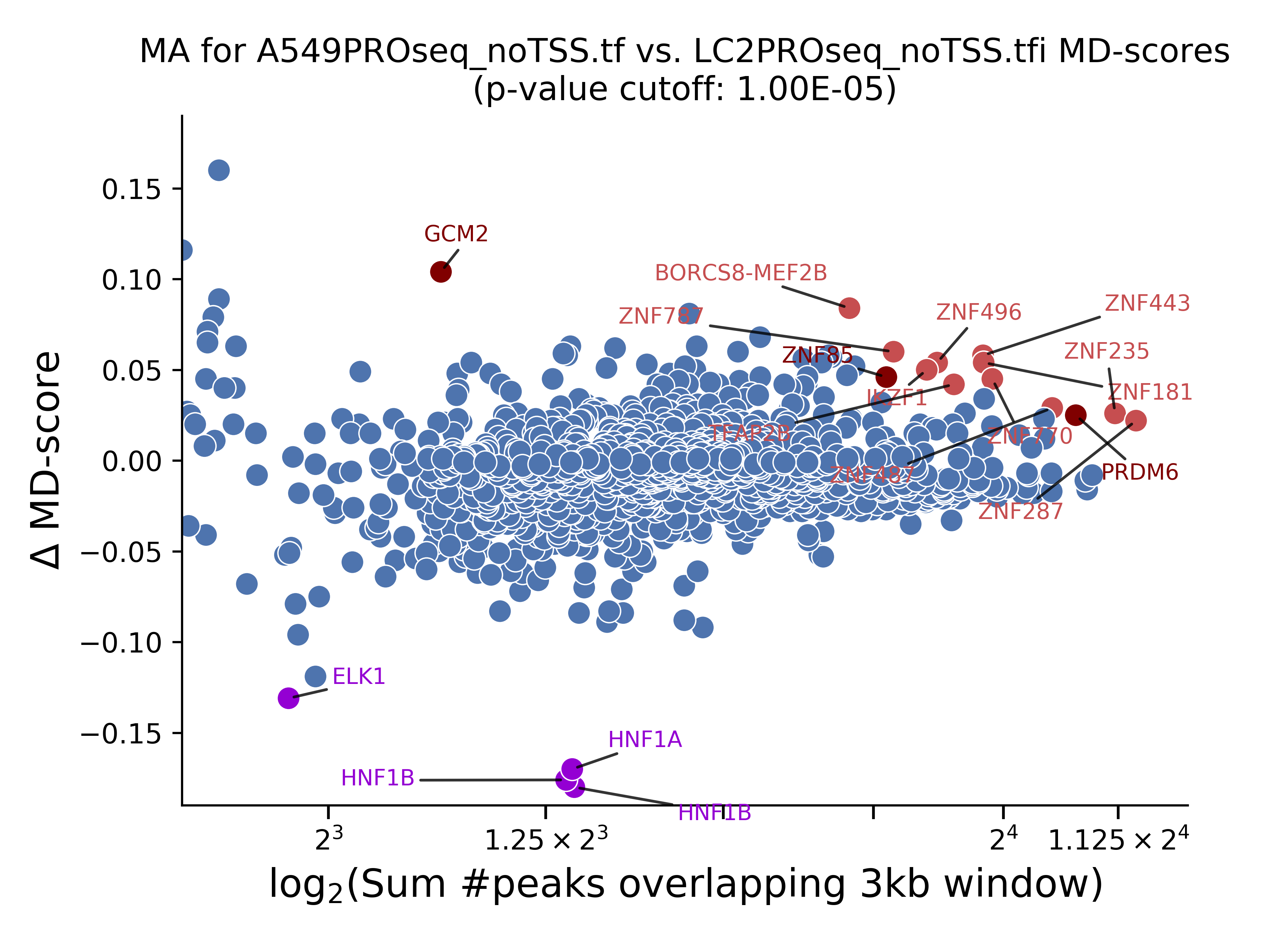

Supplement: Supplemental Data Set 1 [file jciinsight-6-144294-s076.zip › noTSS/best_curated_Human_TFs_p1e-5_grch38/A549_vs_LC2/MA_A549PROseq_noTSS.tfit_merged_to_LC2PROseq_noTSS.tfit_merged_md_score.png]

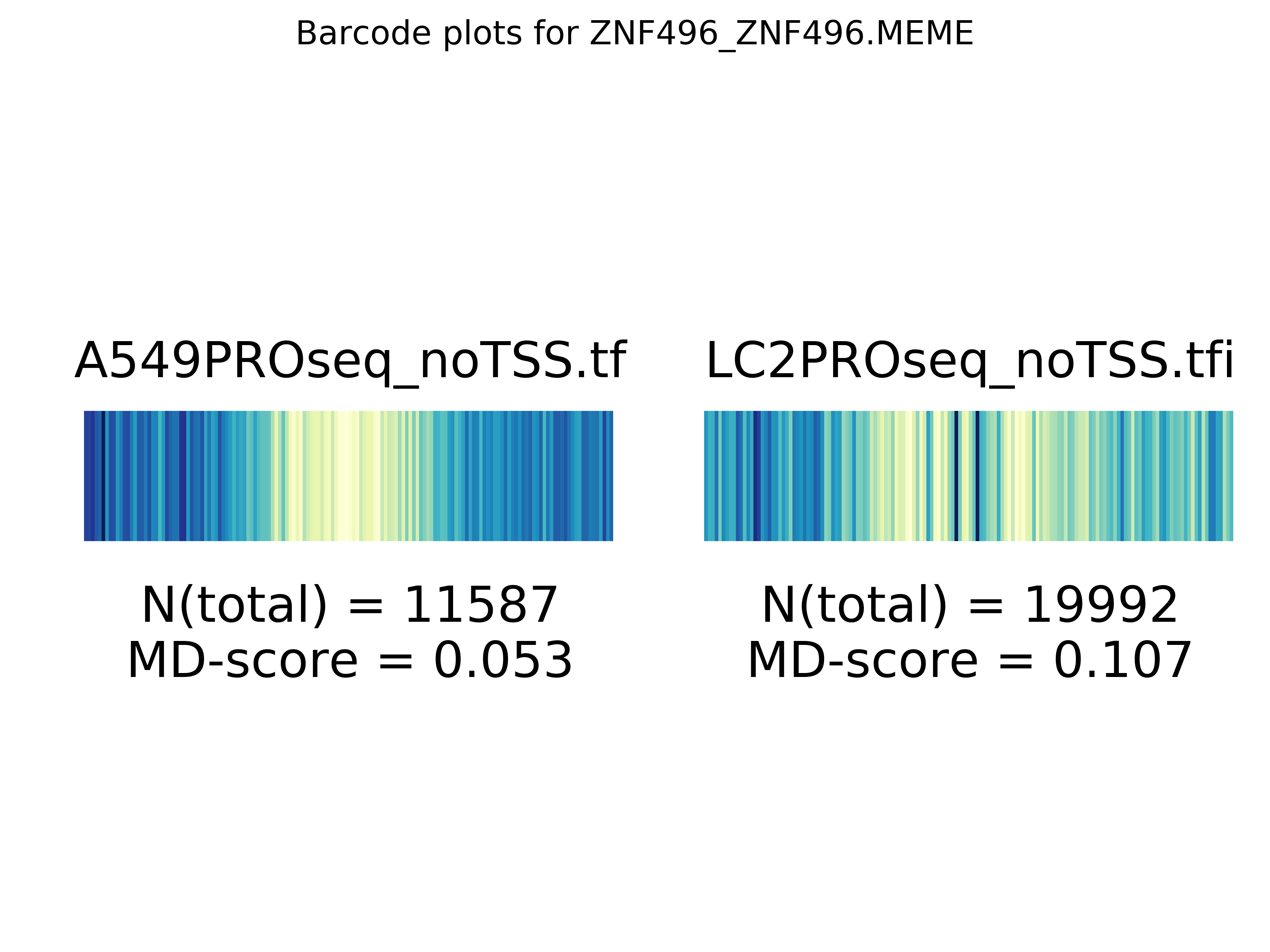

Supplement: Supplemental Data Set 1 [file jciinsight-6-144294-s076.zip › noTSS/best_curated_Human_TFs_p1e-5_grch38/A549_vs_LC2/ZNF496_ZNF496.MEME_barcode_A549PROseq_noTSS.tfit_merged_vs_LC2PROseq_noTSS.tfit_merged.png]

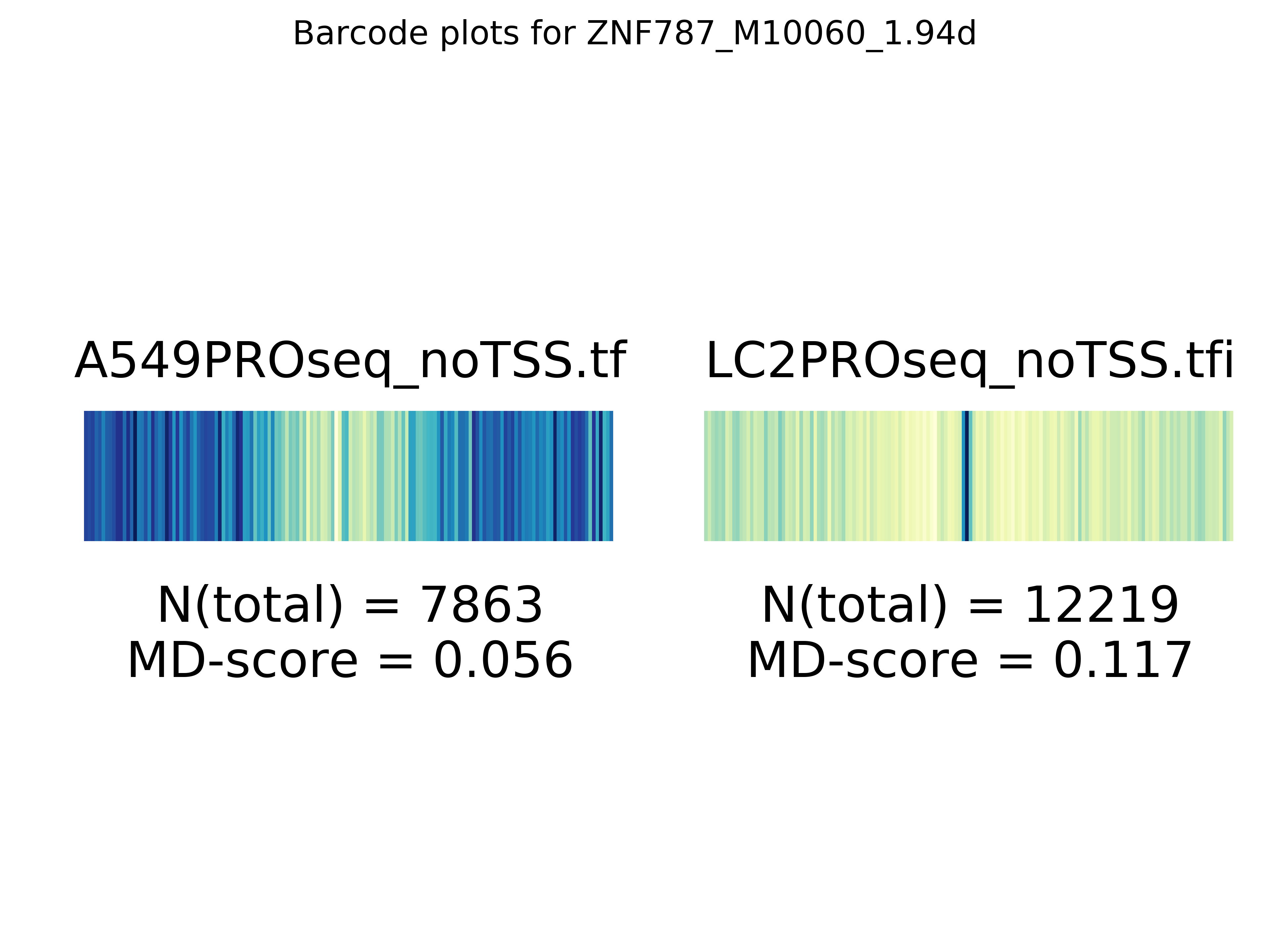

Supplement: Supplemental Data Set 1 [file jciinsight-6-144294-s076.zip › noTSS/best_curated_Human_TFs_p1e-5_grch38/A549_vs_LC2/ZNF787_M10060_1.94d_barcode_A549PROseq_noTSS.tfit_merged_vs_LC2PROseq_noTSS.tfit_merged.png]

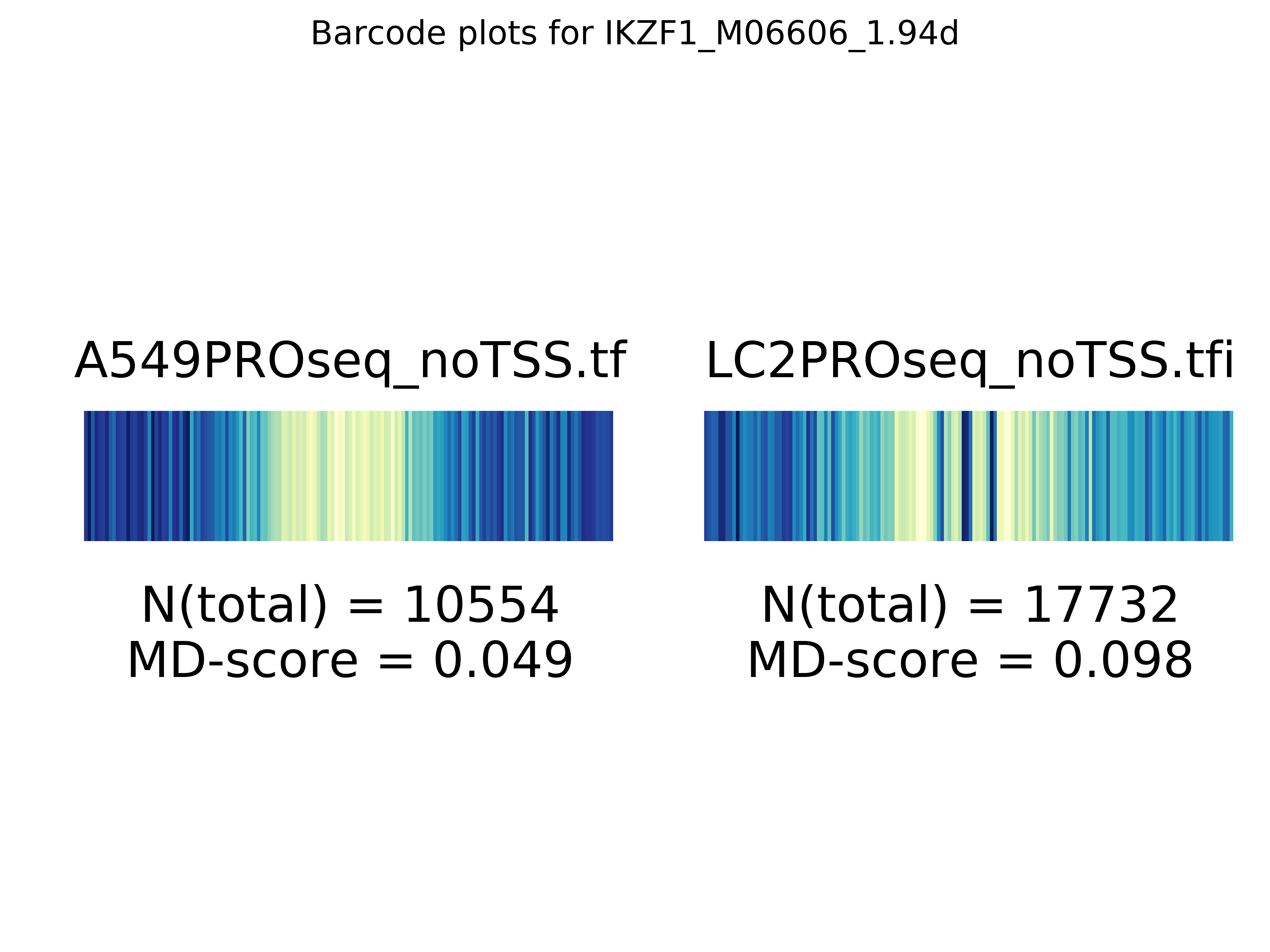

Supplement: Supplemental Data Set 1 [file jciinsight-6-144294-s076.zip › noTSS/best_curated_Human_TFs_p1e-5_grch38/A549_vs_LC2/IKZF1_M06606_1.94d_barcode_A549PROseq_noTSS.tfit_merged_vs_LC2PROseq_noTSS.tfit_merged.png]

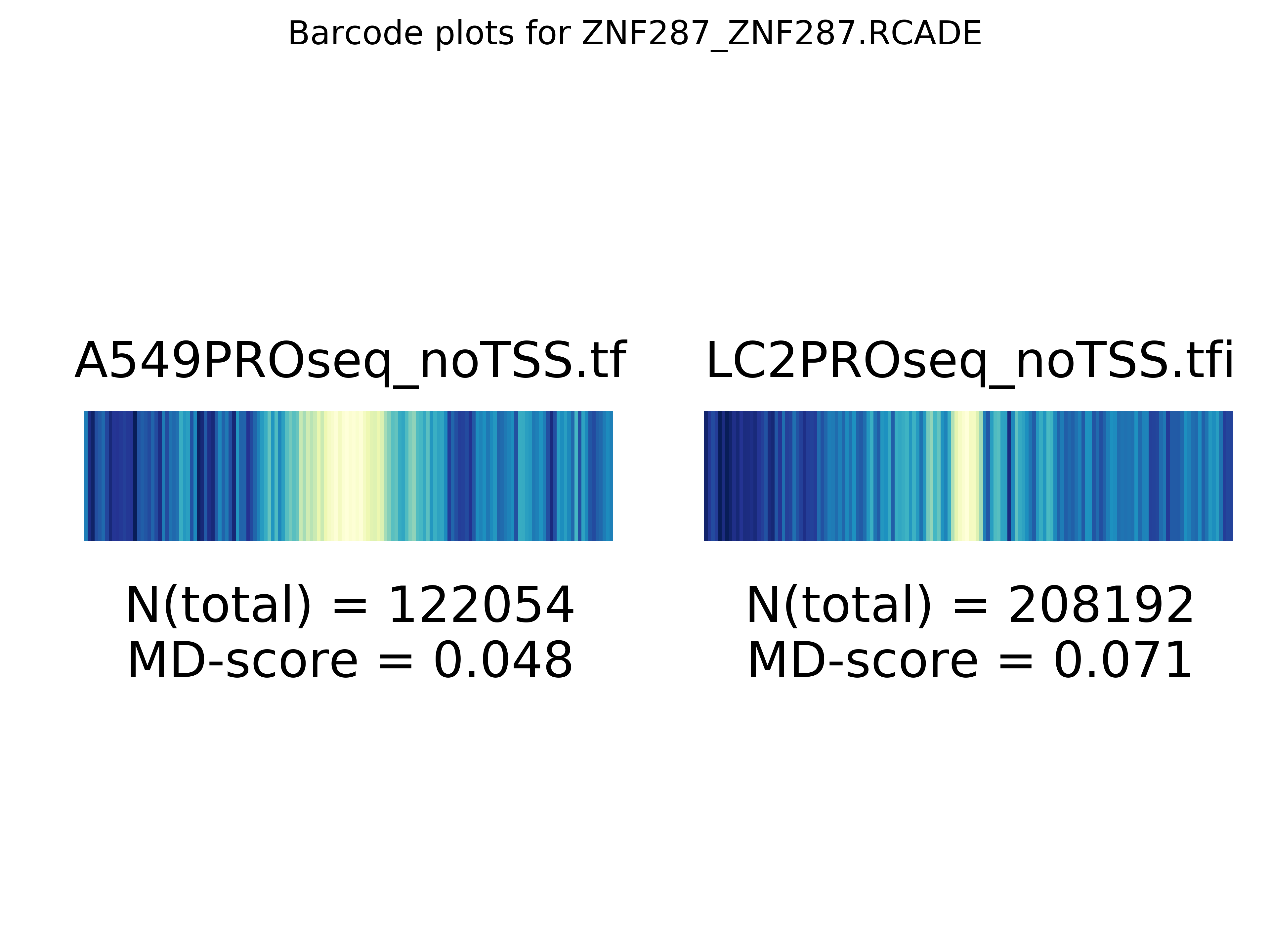

Supplement: Supplemental Data Set 1 [file jciinsight-6-144294-s076.zip › noTSS/best_curated_Human_TFs_p1e-5_grch38/A549_vs_LC2/ZNF287_ZNF287.RCADE_barcode_A549PROseq_noTSS.tfit_merged_vs_LC2PROseq_noTSS.tfit_merged.png]

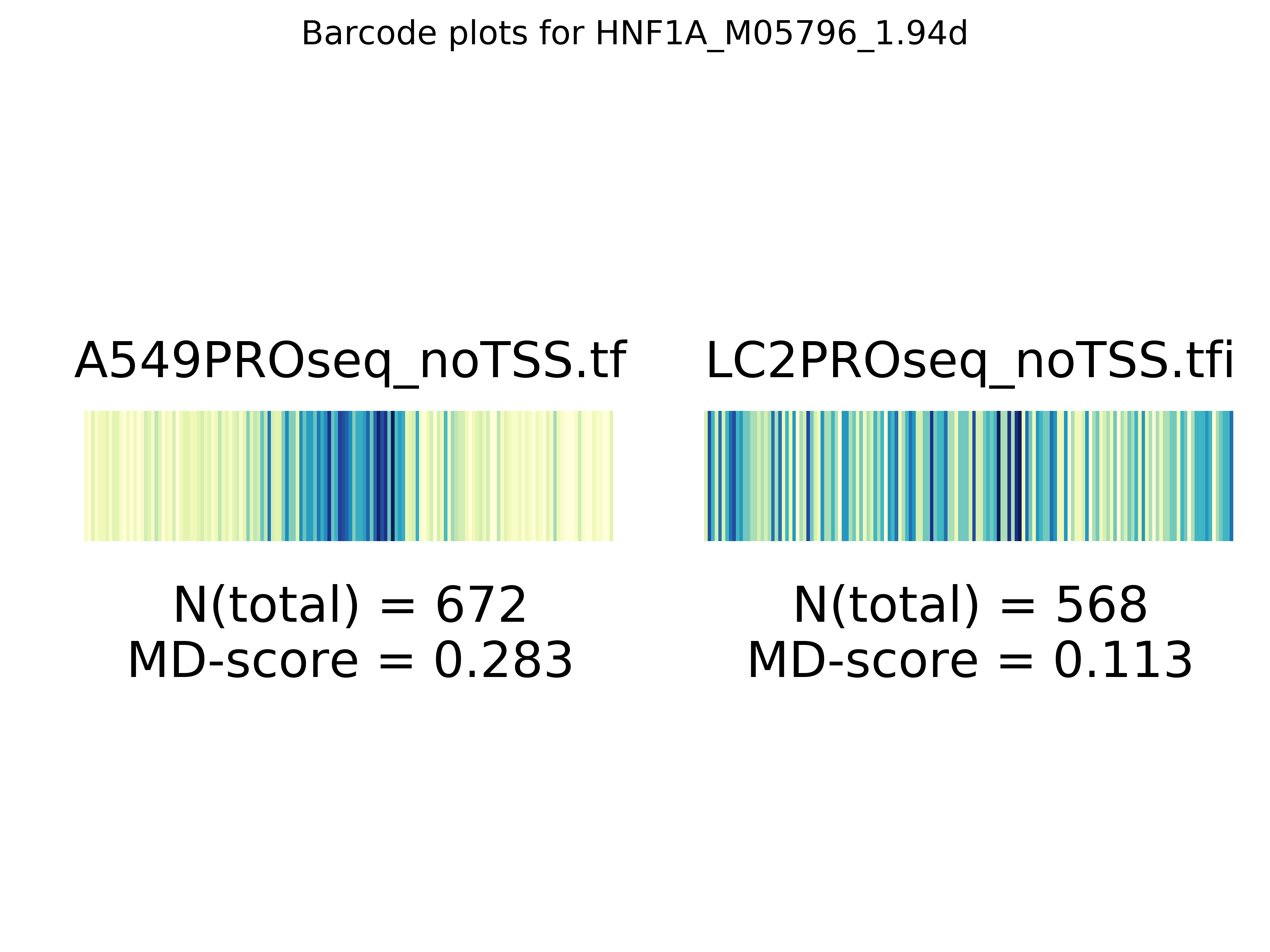

Supplement: Supplemental Data Set 1 [file jciinsight-6-144294-s076.zip › noTSS/best_curated_Human_TFs_p1e-5_grch38/A549_vs_LC2/HNF1A_M05796_1.94d_barcode_A549PROseq_noTSS.tfit_merged_vs_LC2PROseq_noTSS.tfit_merged.png]

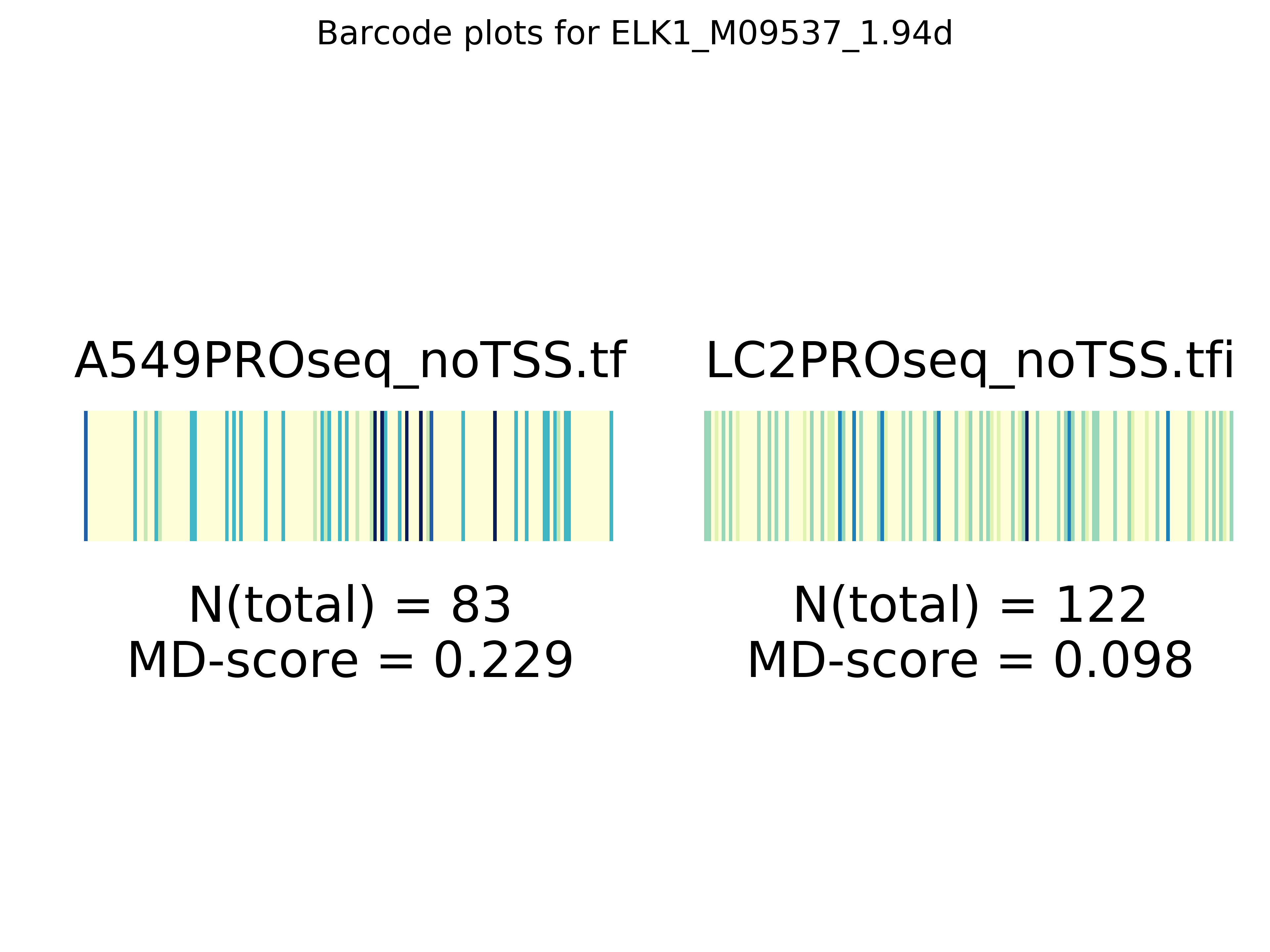

Supplement: Supplemental Data Set 1 [file jciinsight-6-144294-s076.zip › noTSS/best_curated_Human_TFs_p1e-5_grch38/A549_vs_LC2/ELK1_M09537_1.94d_barcode_A549PROseq_noTSS.tfit_merged_vs_LC2PROseq_noTSS.tfit_merged.png]

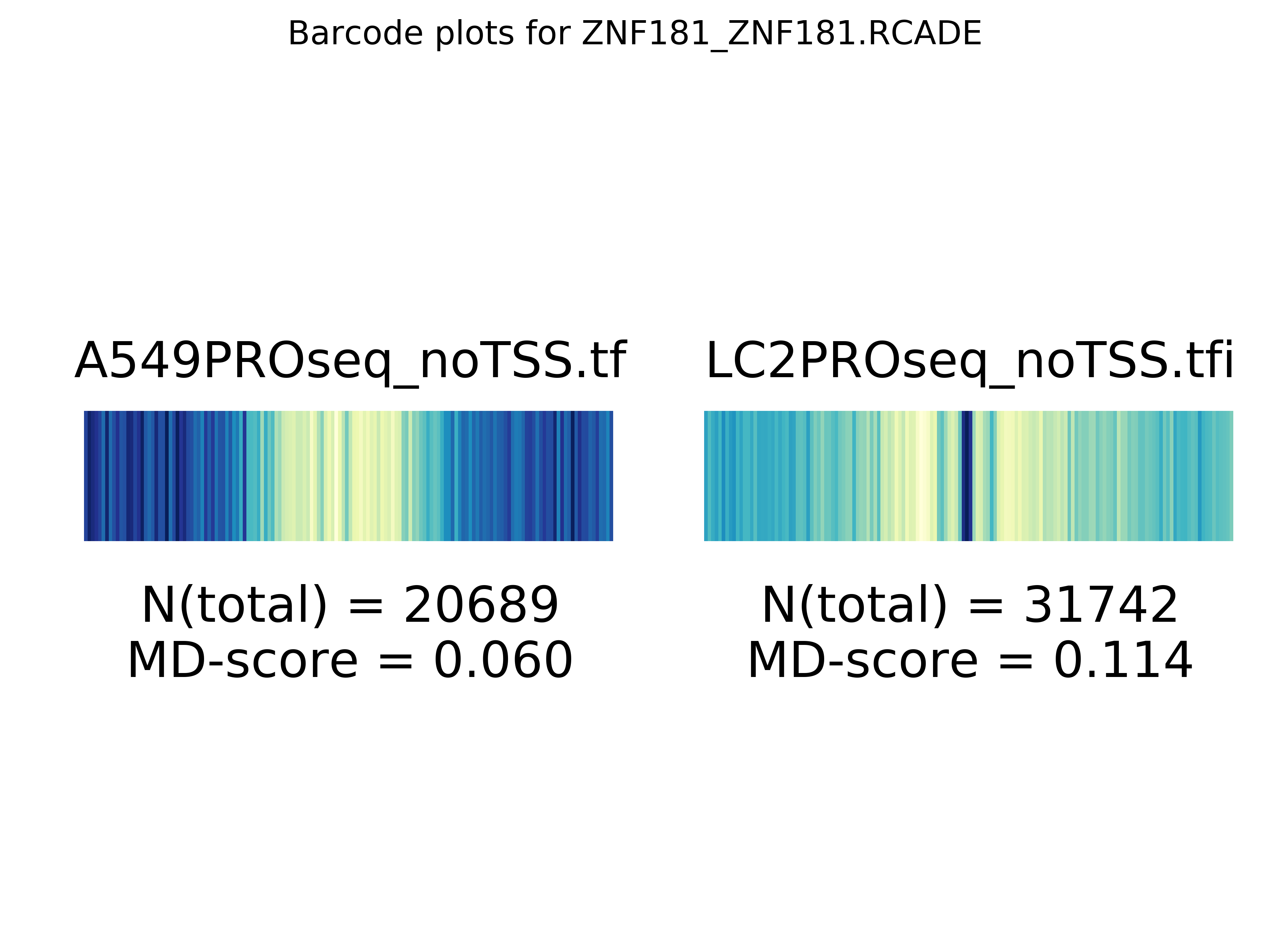

Supplement: Supplemental Data Set 1 [file jciinsight-6-144294-s076.zip › noTSS/best_curated_Human_TFs_p1e-5_grch38/A549_vs_LC2/ZNF181_ZNF181.RCADE_barcode_A549PROseq_noTSS.tfit_merged_vs_LC2PROseq_noTSS.tfit_merged.png]

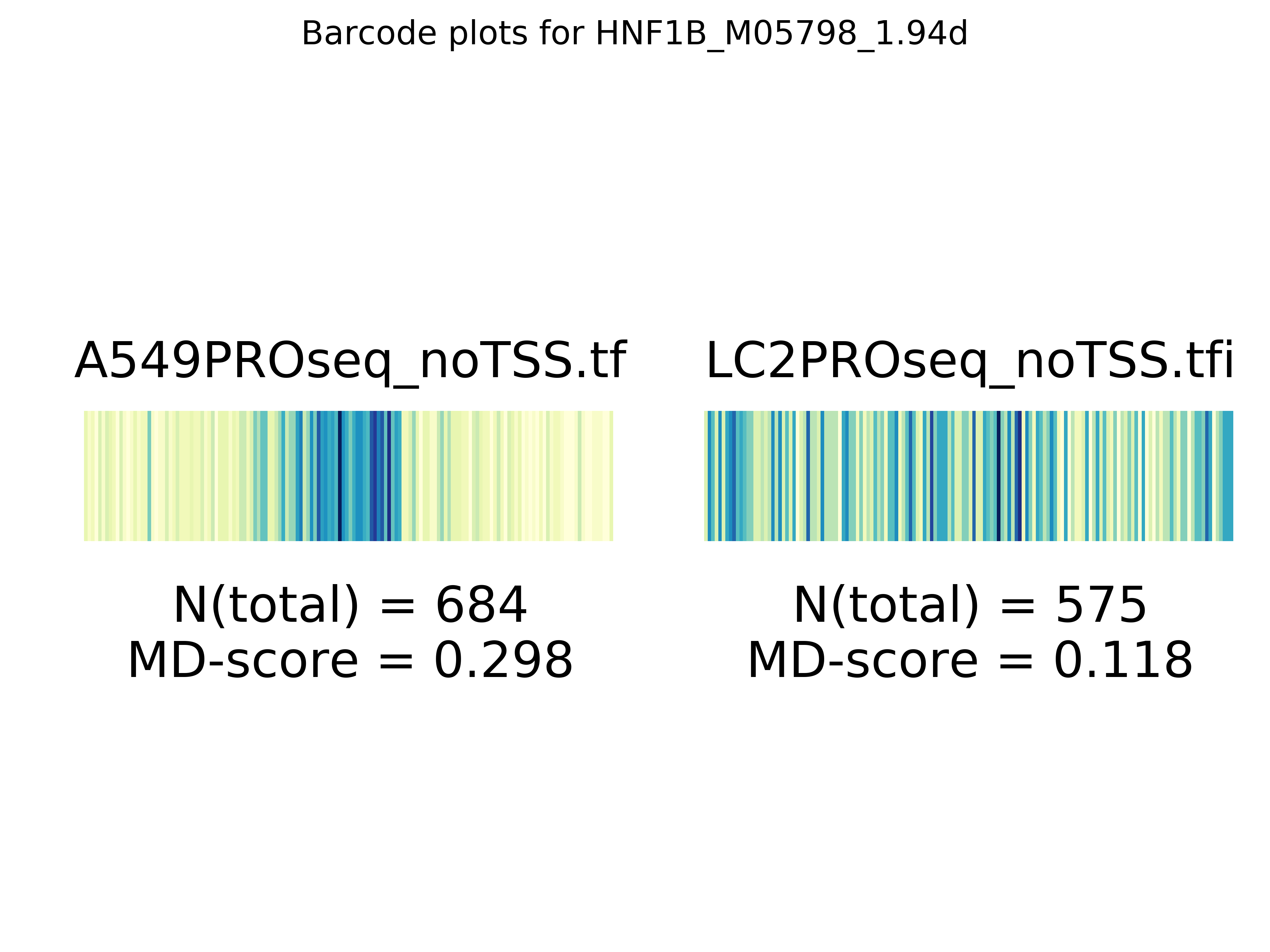

Supplement: Supplemental Data Set 1 [file jciinsight-6-144294-s076.zip › noTSS/best_curated_Human_TFs_p1e-5_grch38/A549_vs_LC2/HNF1B_M05798_1.94d_barcode_A549PROseq_noTSS.tfit_merged_vs_LC2PROseq_noTSS.tfit_merged.png]

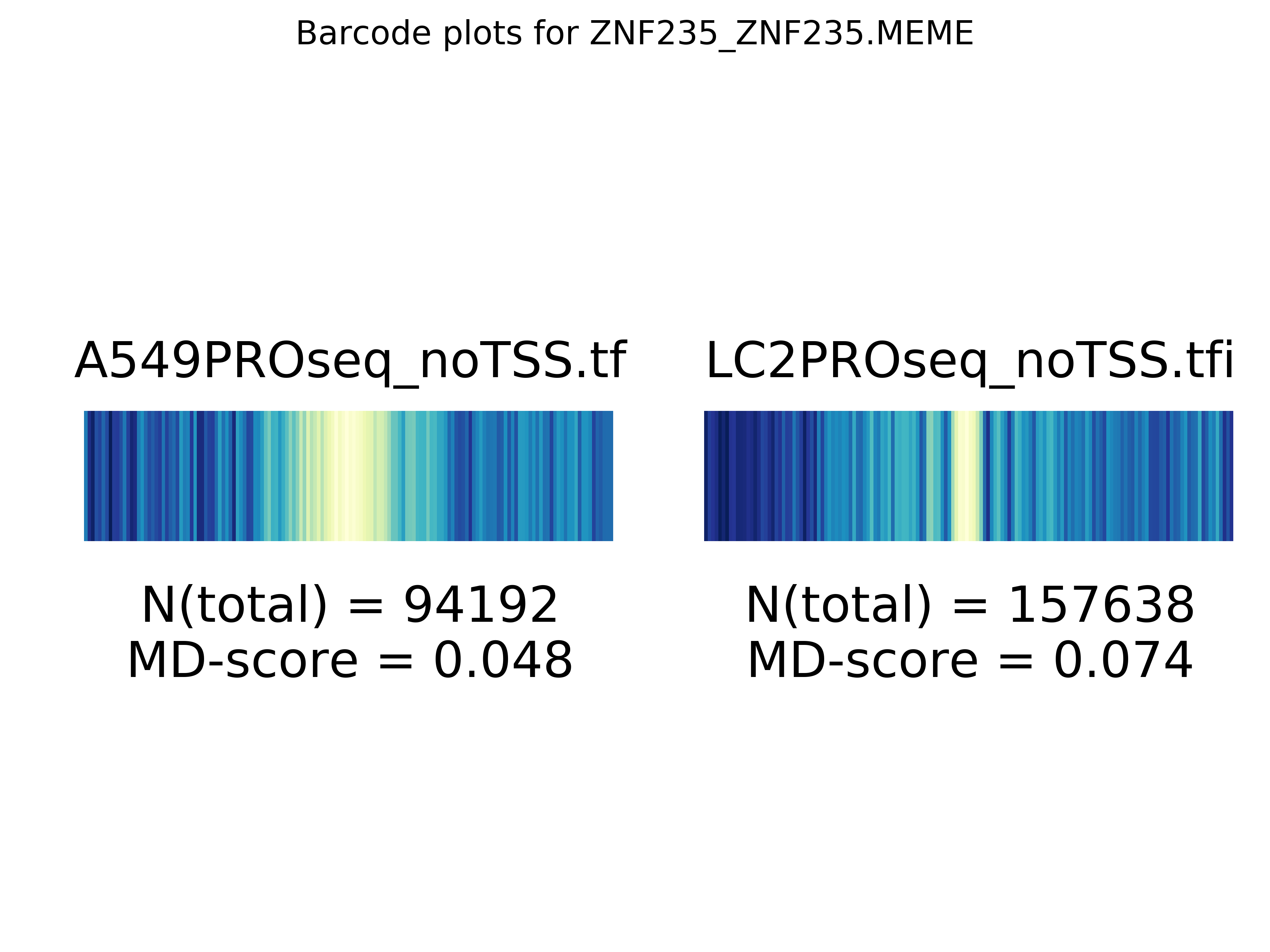

Supplement: Supplemental Data Set 1 [file jciinsight-6-144294-s076.zip › noTSS/best_curated_Human_TFs_p1e-5_grch38/A549_vs_LC2/ZNF235_ZNF235.MEME_barcode_A549PROseq_noTSS.tfit_merged_vs_LC2PROseq_noTSS.tfit_merged.png]

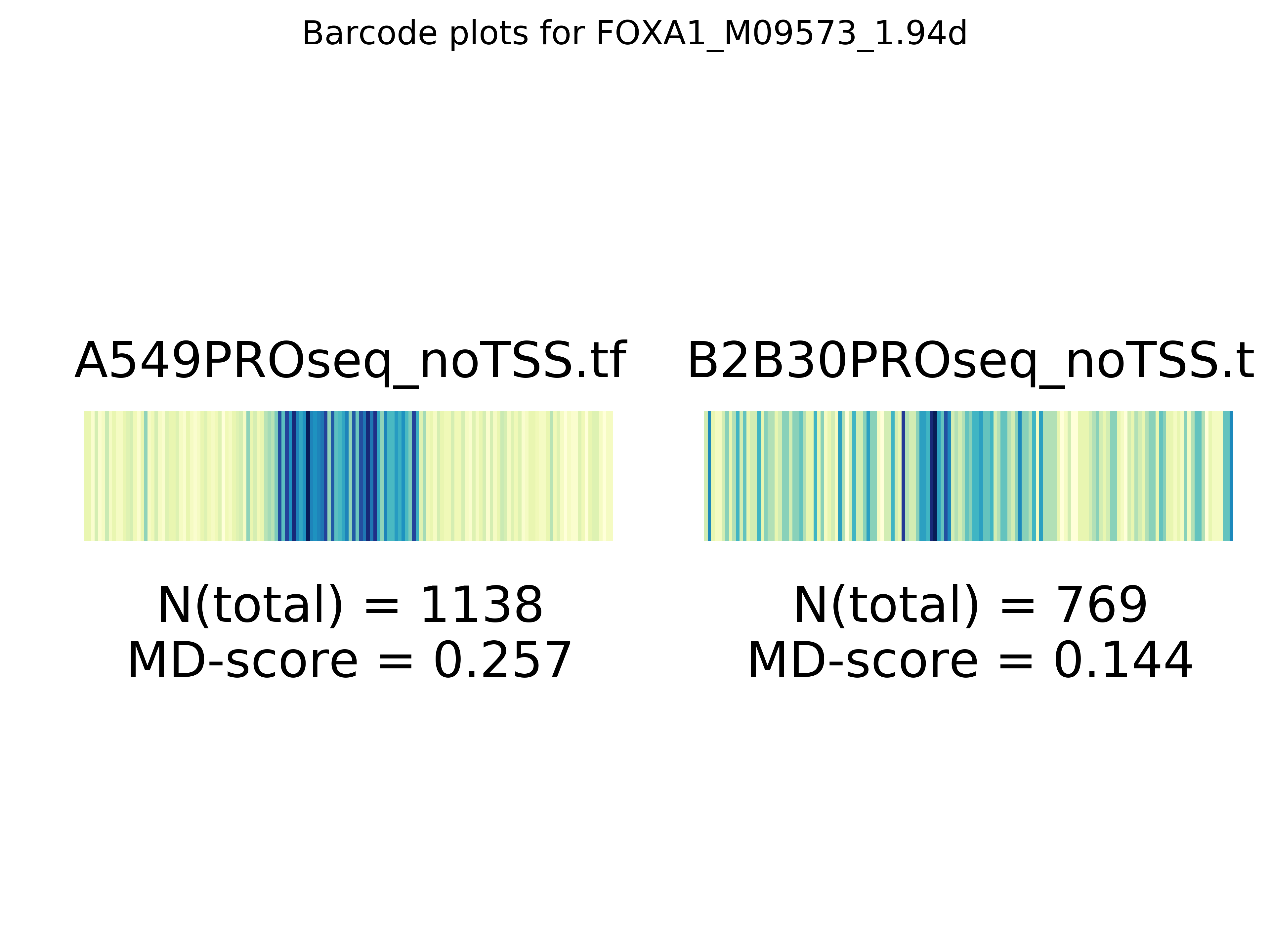

Supplement: Supplemental Data Set 1 [file jciinsight-6-144294-s076.zip › noTSS/best_curated_Human_TFs_p1e-5_grch38/A549_vs_B2B/FOXA1_M09573_1.94d_barcode_A549PROseq_noTSS.tfit_merged_vs_B2B30PROseq_noTSS.tfit_merged.png]

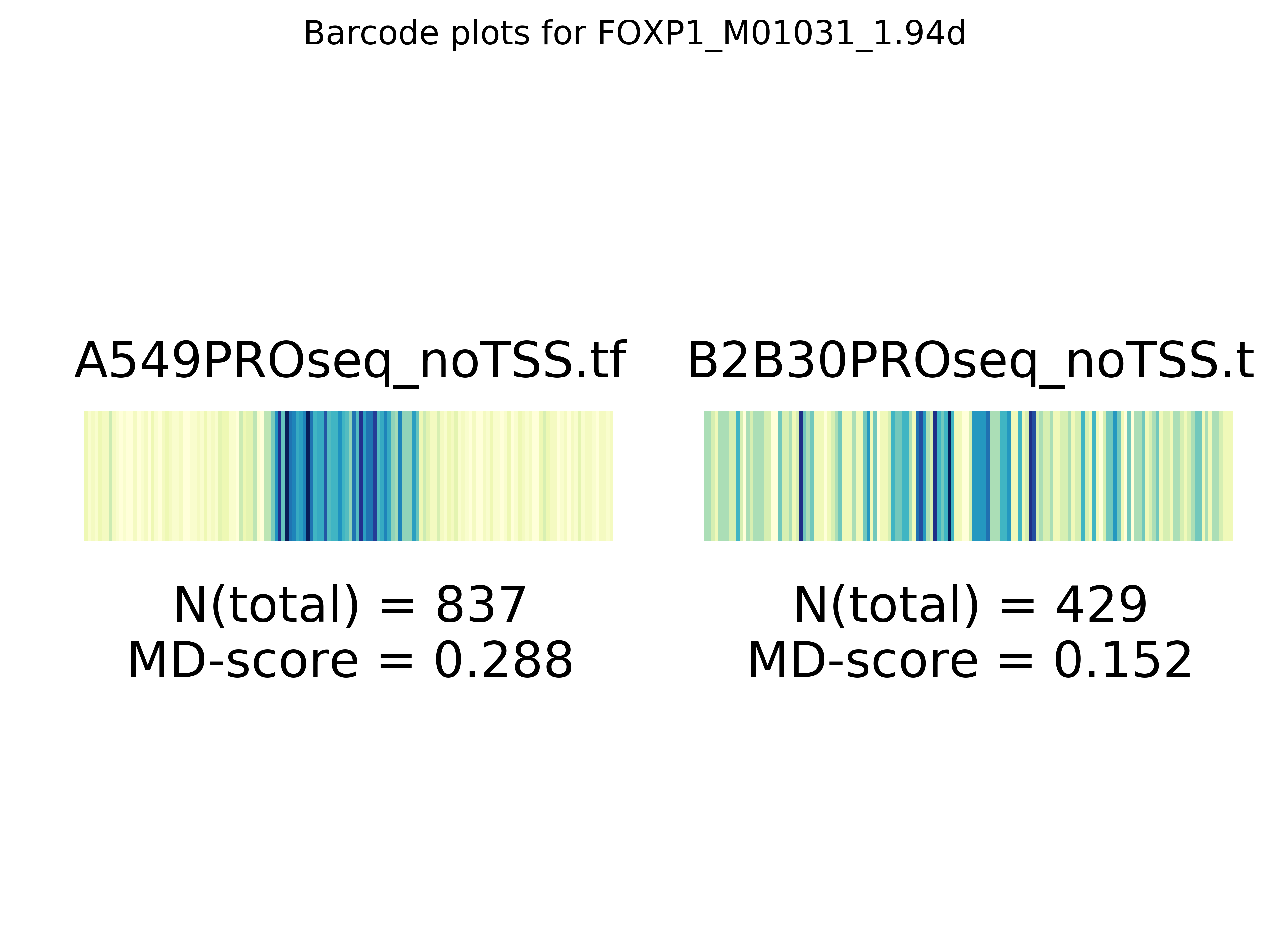

Supplement: Supplemental Data Set 1 [file jciinsight-6-144294-s076.zip › noTSS/best_curated_Human_TFs_p1e-5_grch38/A549_vs_B2B/FOXP1_M01031_1.94d_barcode_A549PROseq_noTSS.tfit_merged_vs_B2B30PROseq_noTSS.tfit_merged.png]

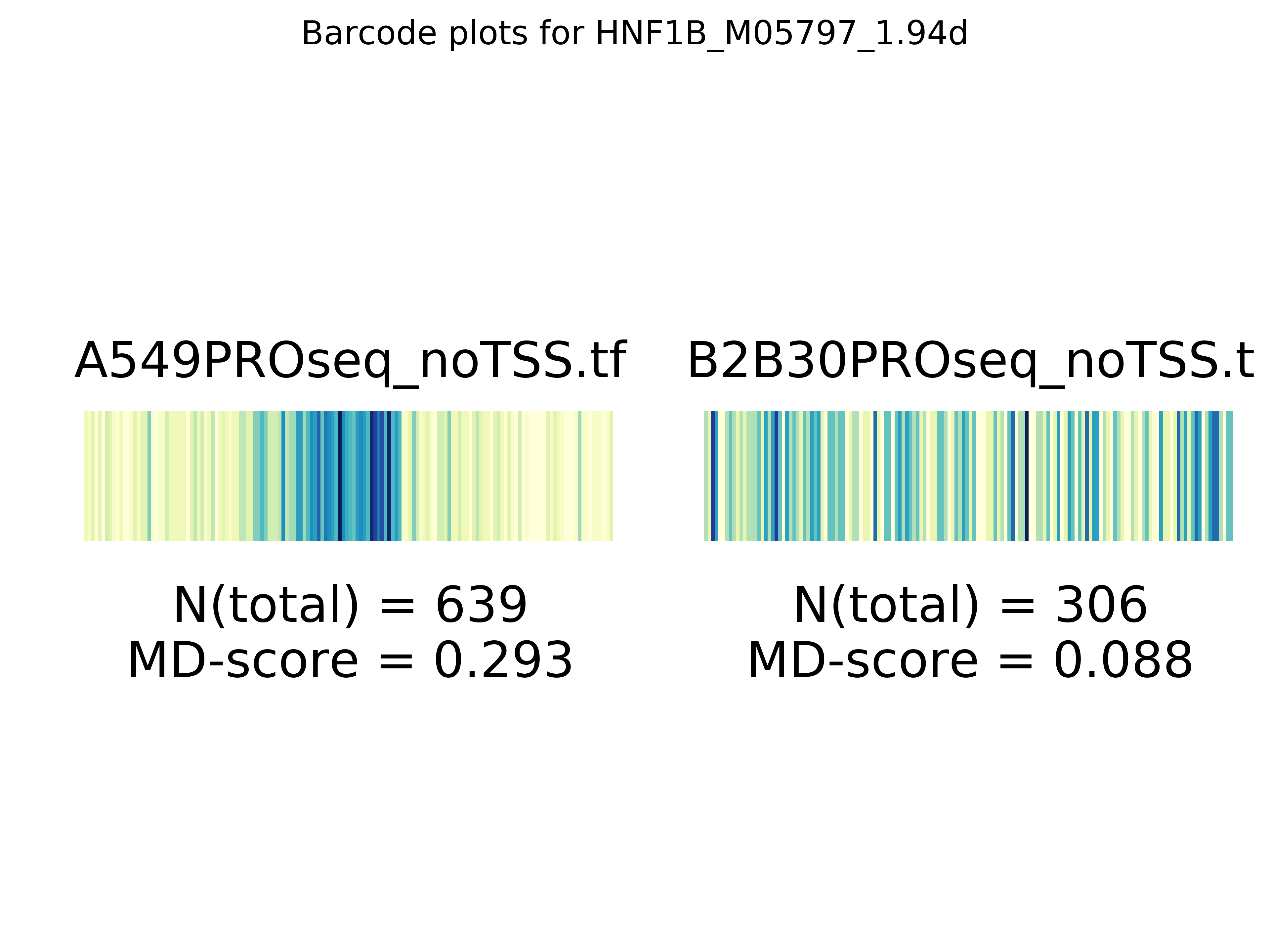

Supplement: Supplemental Data Set 1 [file jciinsight-6-144294-s076.zip › noTSS/best_curated_Human_TFs_p1e-5_grch38/A549_vs_B2B/HNF1B_M05797_1.94d_barcode_A549PROseq_noTSS.tfit_merged_vs_B2B30PROseq_noTSS.tfit_merged.png]

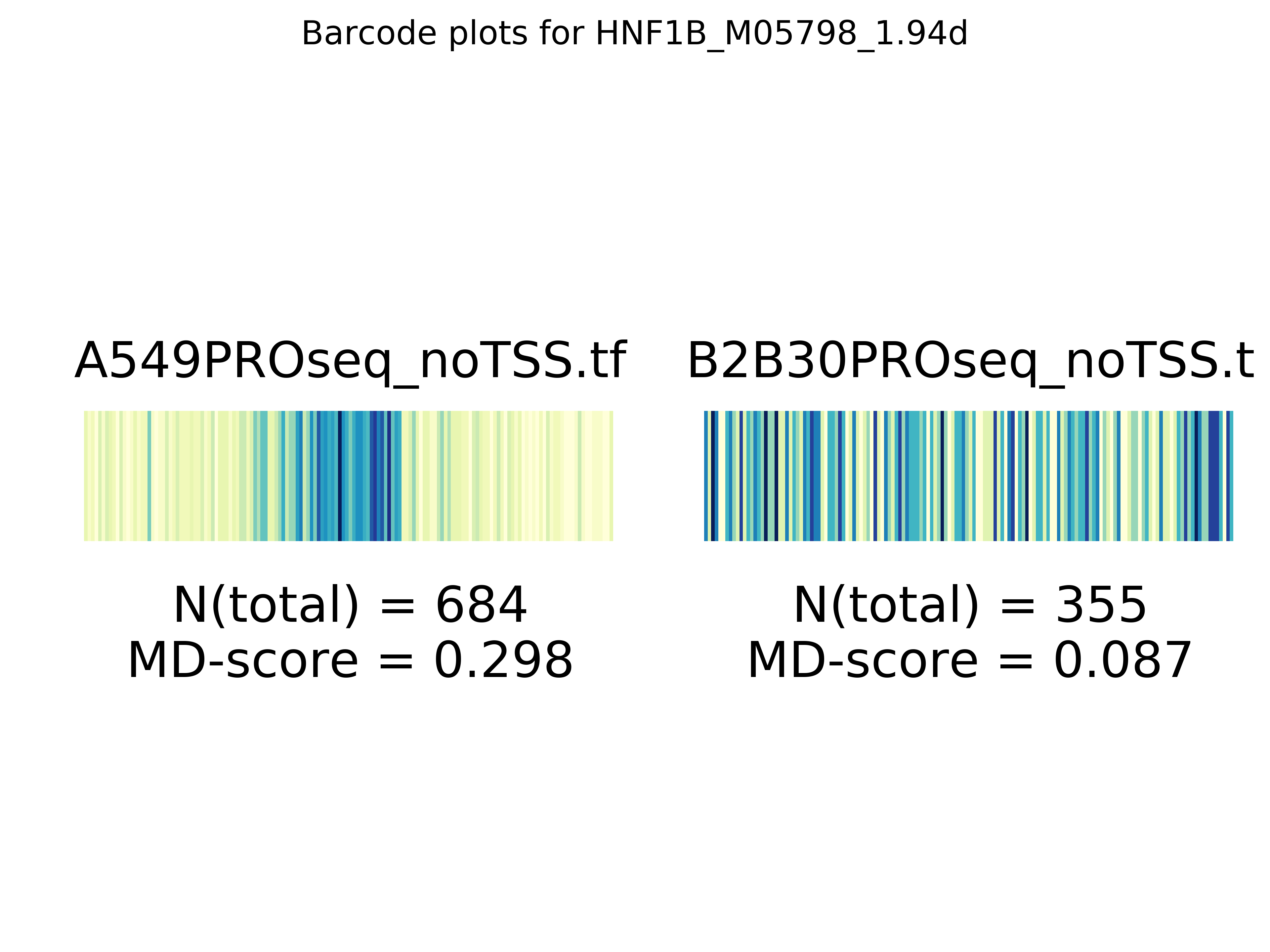

Supplement: Supplemental Data Set 1 [file jciinsight-6-144294-s076.zip › noTSS/best_curated_Human_TFs_p1e-5_grch38/A549_vs_B2B/HNF1B_M05798_1.94d_barcode_A549PROseq_noTSS.tfit_merged_vs_B2B30PROseq_noTSS.tfit_merged.png]

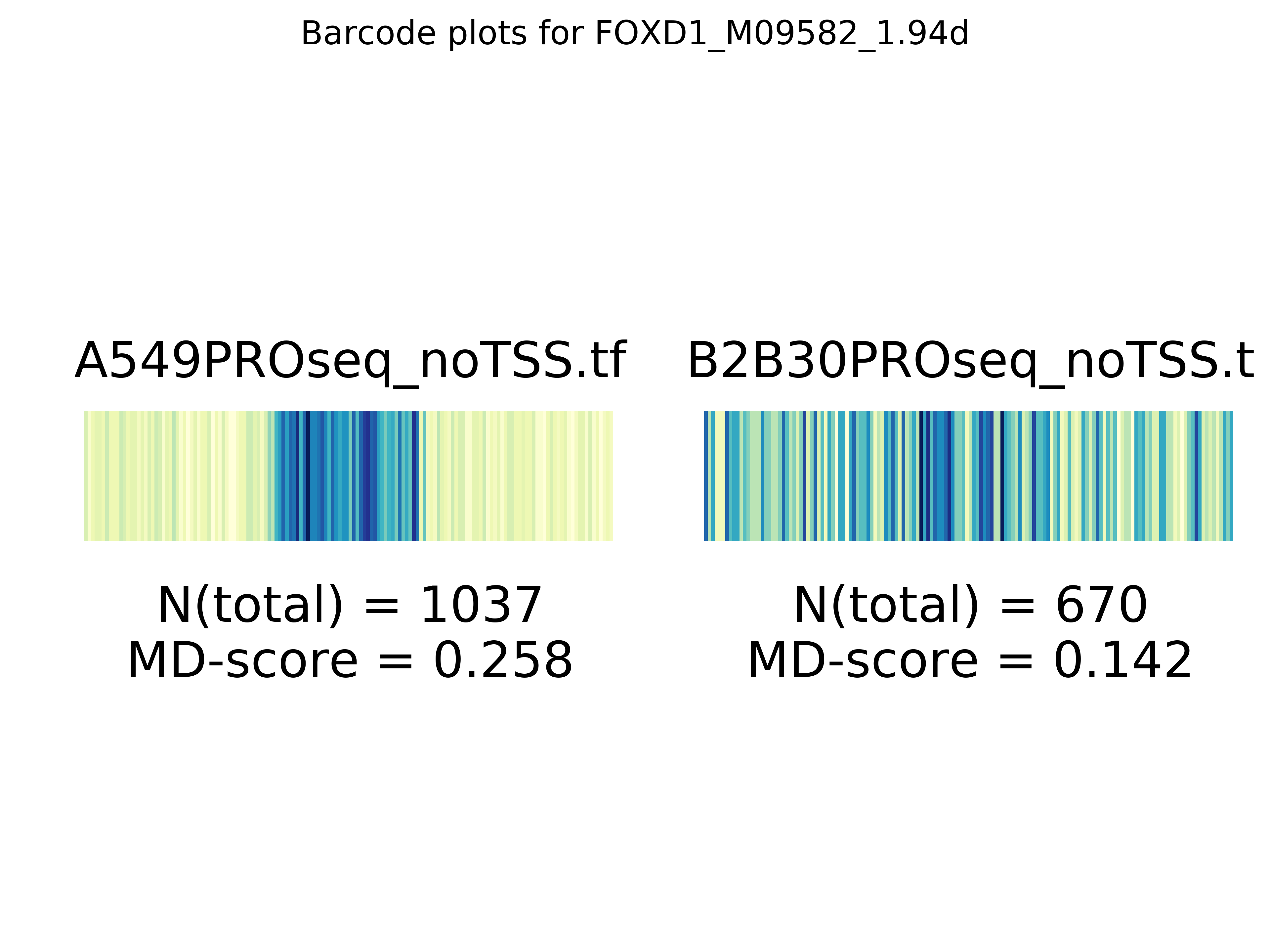

Supplement: Supplemental Data Set 1 [file jciinsight-6-144294-s076.zip › noTSS/best_curated_Human_TFs_p1e-5_grch38/A549_vs_B2B/FOXD1_M09582_1.94d_barcode_A549PROseq_noTSS.tfit_merged_vs_B2B30PROseq_noTSS.tfit_merged.png]

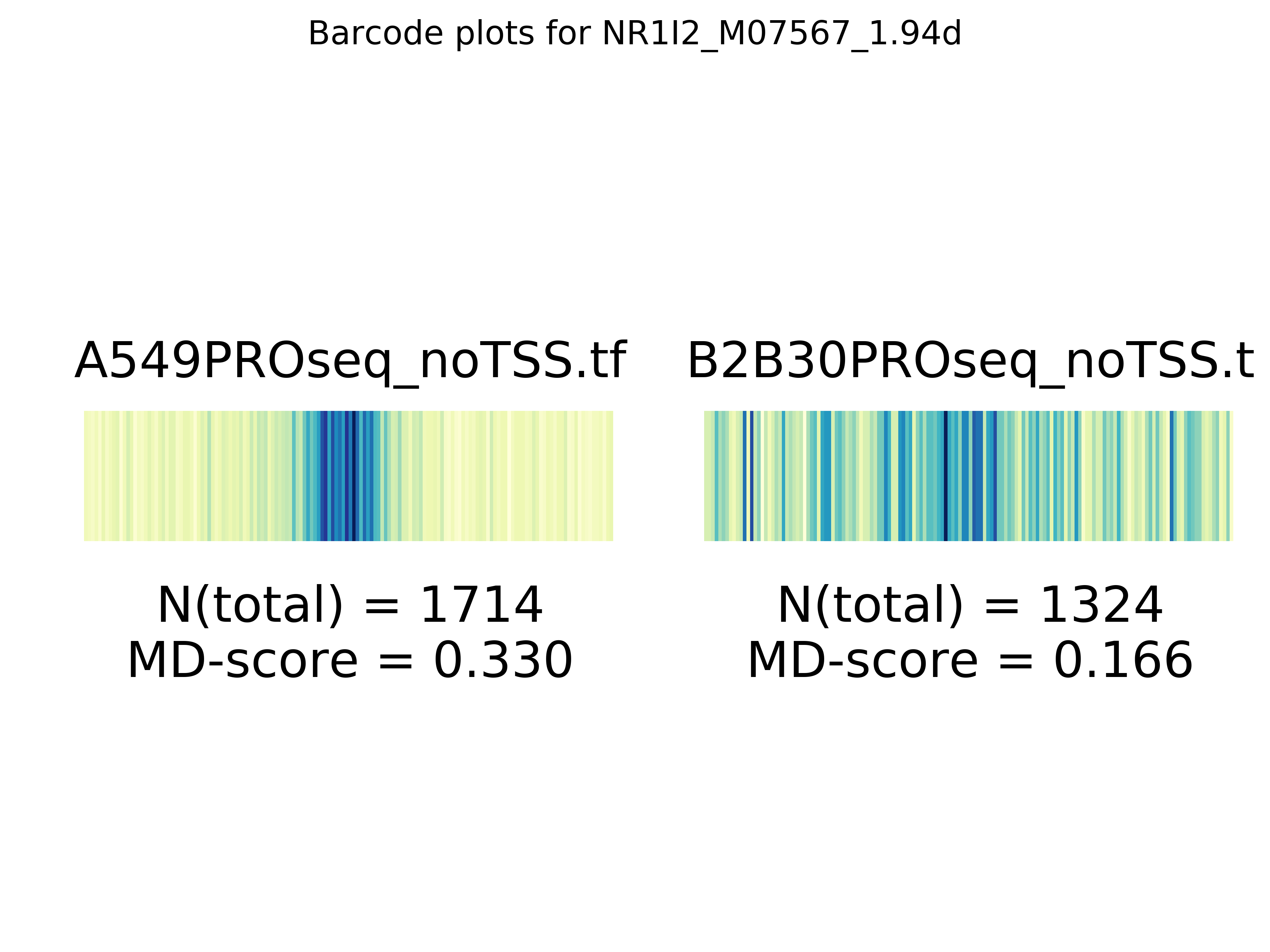

Supplement: Supplemental Data Set 1 [file jciinsight-6-144294-s076.zip › noTSS/best_curated_Human_TFs_p1e-5_grch38/A549_vs_B2B/NR1I2_M07567_1.94d_barcode_A549PROseq_noTSS.tfit_merged_vs_B2B30PROseq_noTSS.tfit_merged.png]

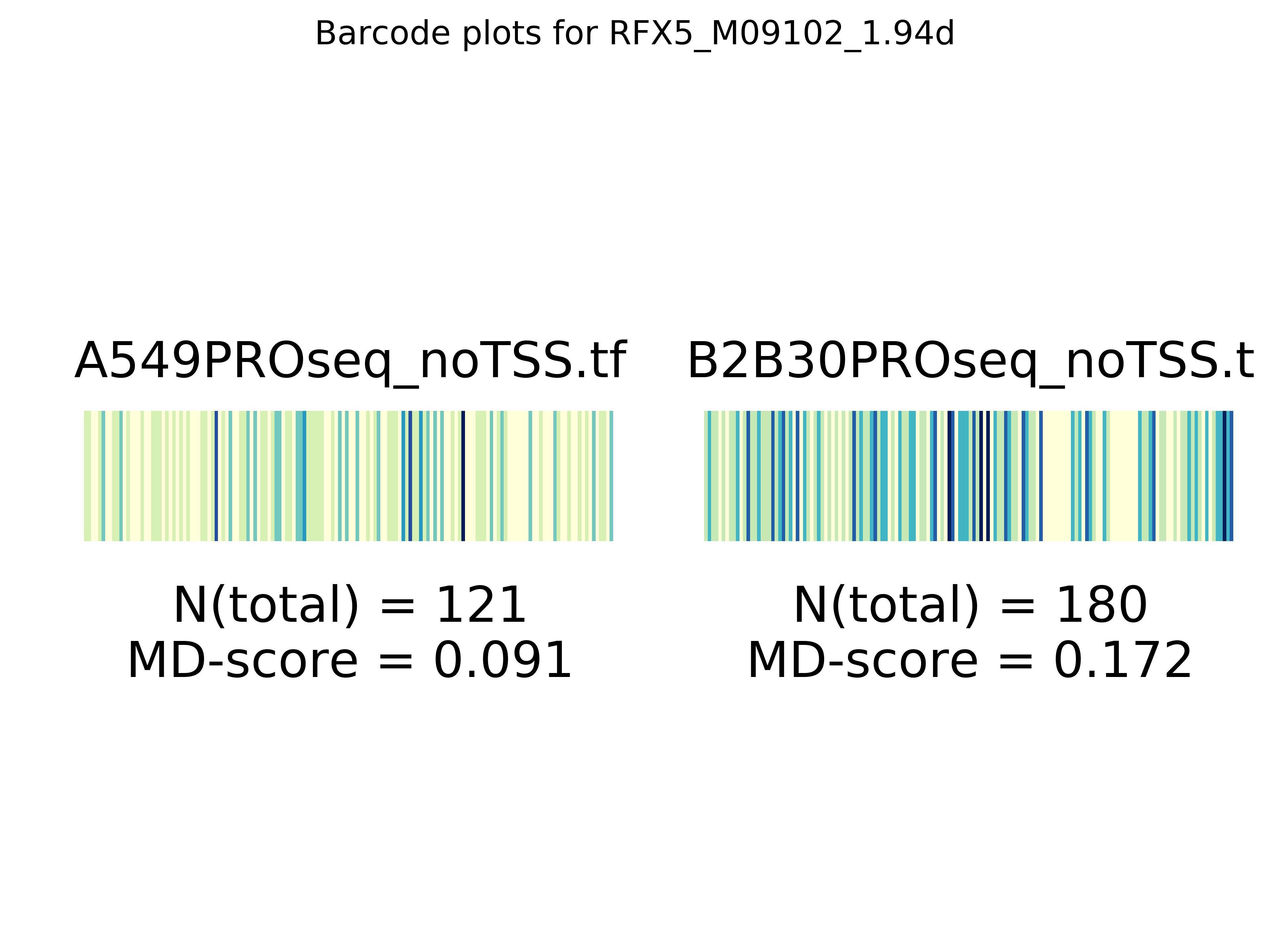

Supplement: Supplemental Data Set 1 [file jciinsight-6-144294-s076.zip › noTSS/best_curated_Human_TFs_p1e-5_grch38/A549_vs_B2B/RFX5_M09102_1.94d_barcode_A549PROseq_noTSS.tfit_merged_vs_B2B30PROseq_noTSS.tfit_merged.png]

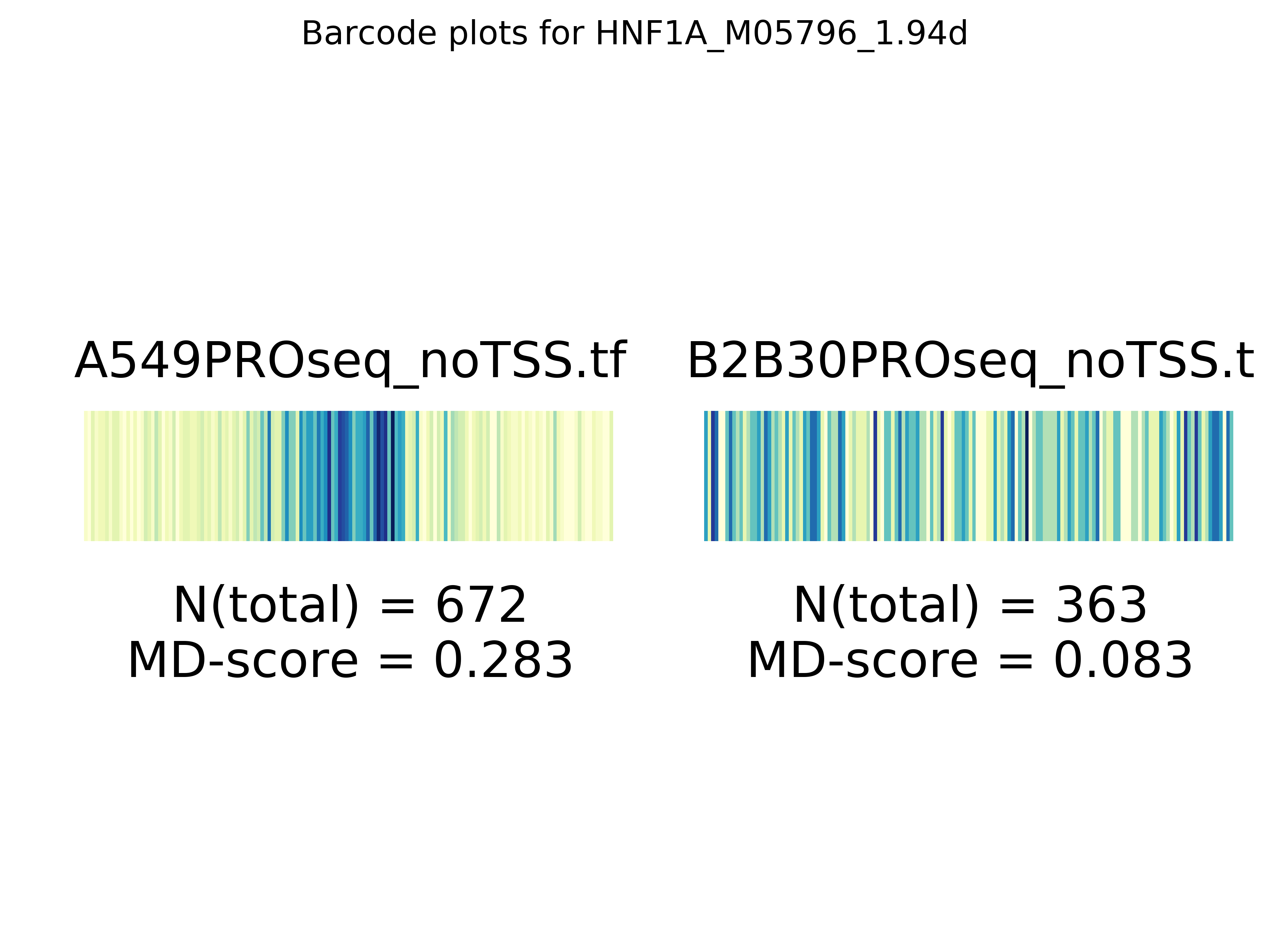

Supplement: Supplemental Data Set 1 [file jciinsight-6-144294-s076.zip › noTSS/best_curated_Human_TFs_p1e-5_grch38/A549_vs_B2B/HNF1A_M05796_1.94d_barcode_A549PROseq_noTSS.tfit_merged_vs_B2B30PROseq_noTSS.tfit_merged.png]

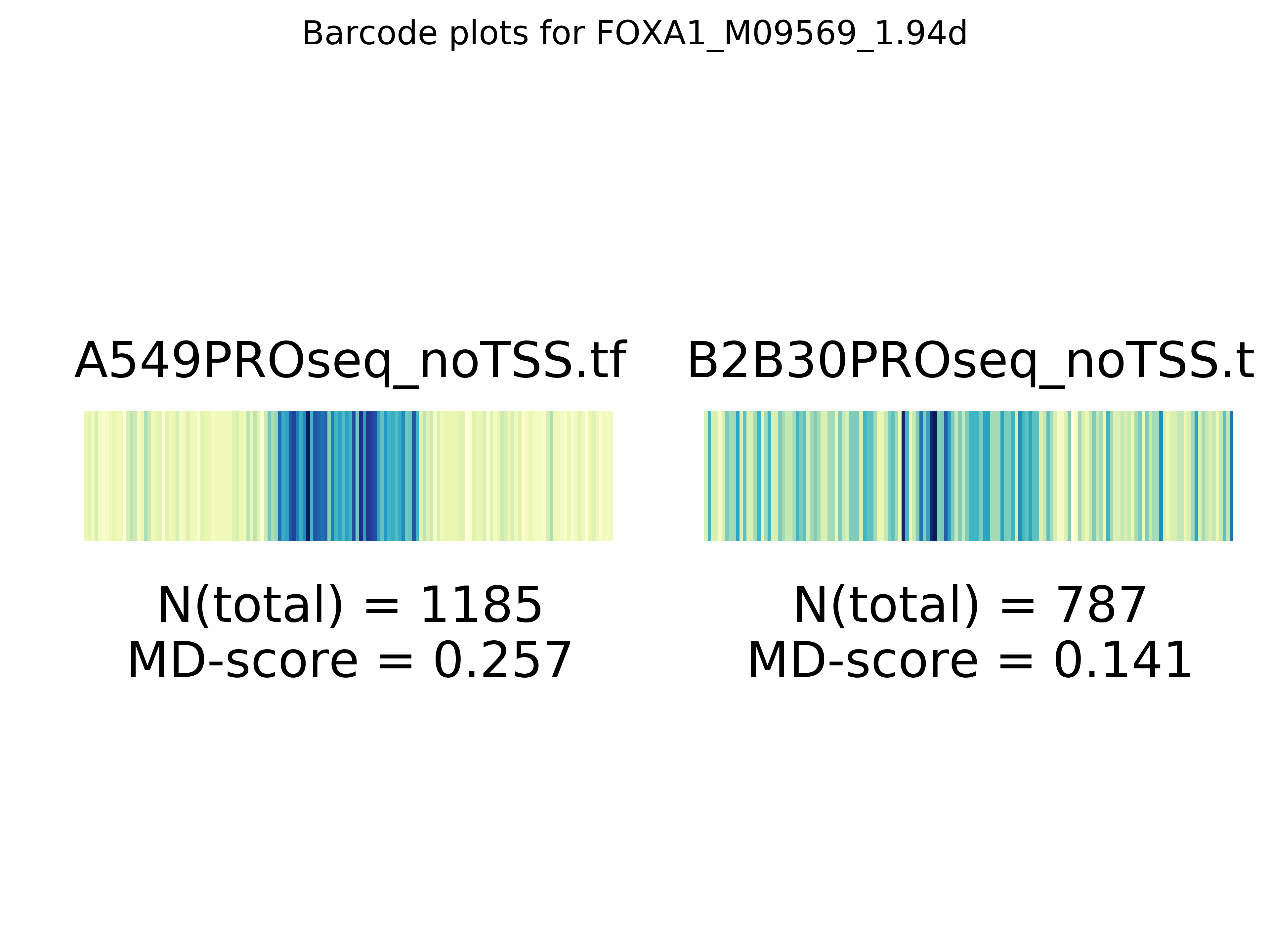

Supplement: Supplemental Data Set 1 [file jciinsight-6-144294-s076.zip › noTSS/best_curated_Human_TFs_p1e-5_grch38/A549_vs_B2B/FOXA1_M09569_1.94d_barcode_A549PROseq_noTSS.tfit_merged_vs_B2B30PROseq_noTSS.tfit_merged.png]

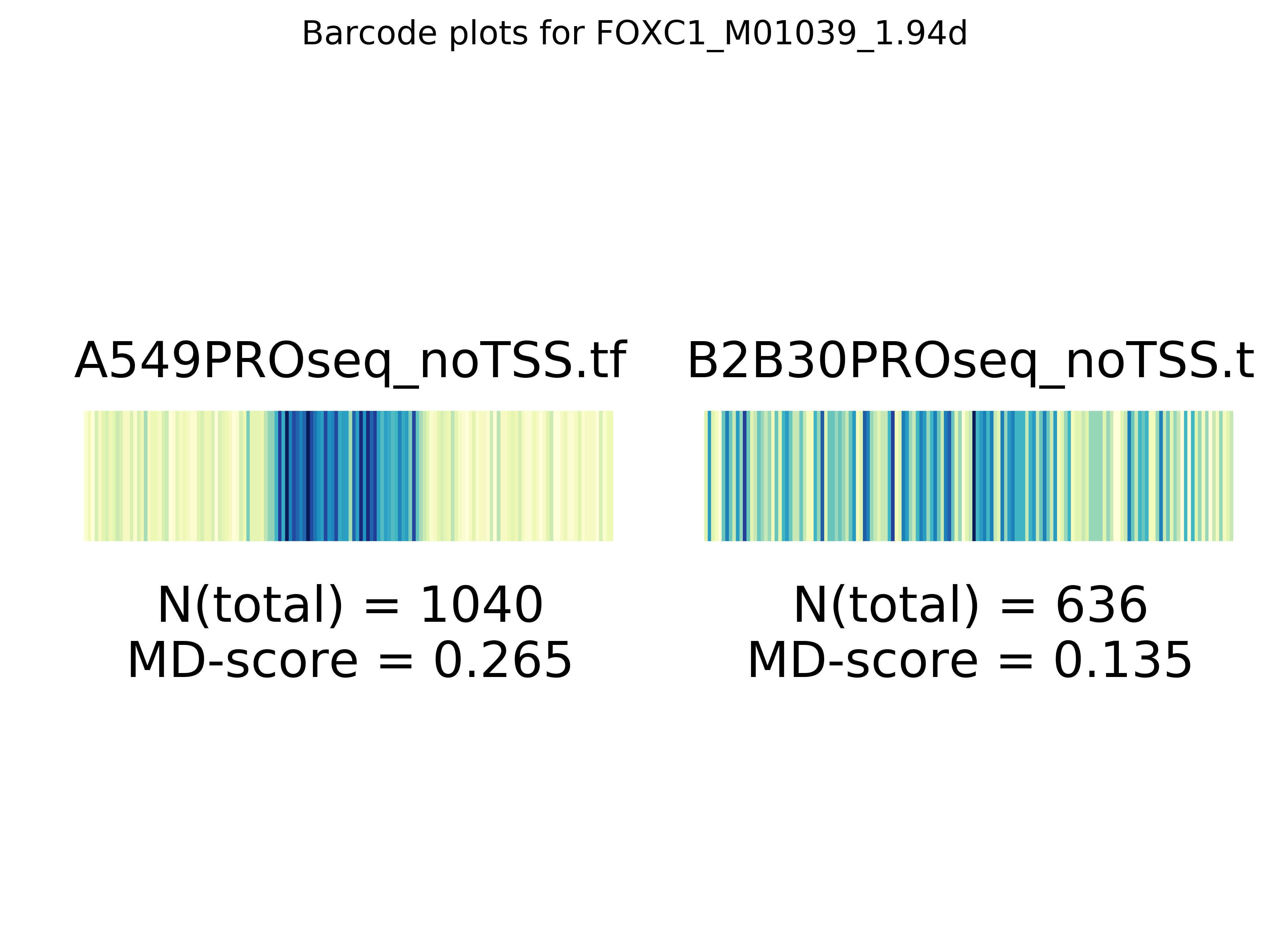

Supplement: Supplemental Data Set 1 [file jciinsight-6-144294-s076.zip › noTSS/best_curated_Human_TFs_p1e-5_grch38/A549_vs_B2B/FOXC1_M01039_1.94d_barcode_A549PROseq_noTSS.tfit_merged_vs_B2B30PROseq_noTSS.tfit_merged.png]

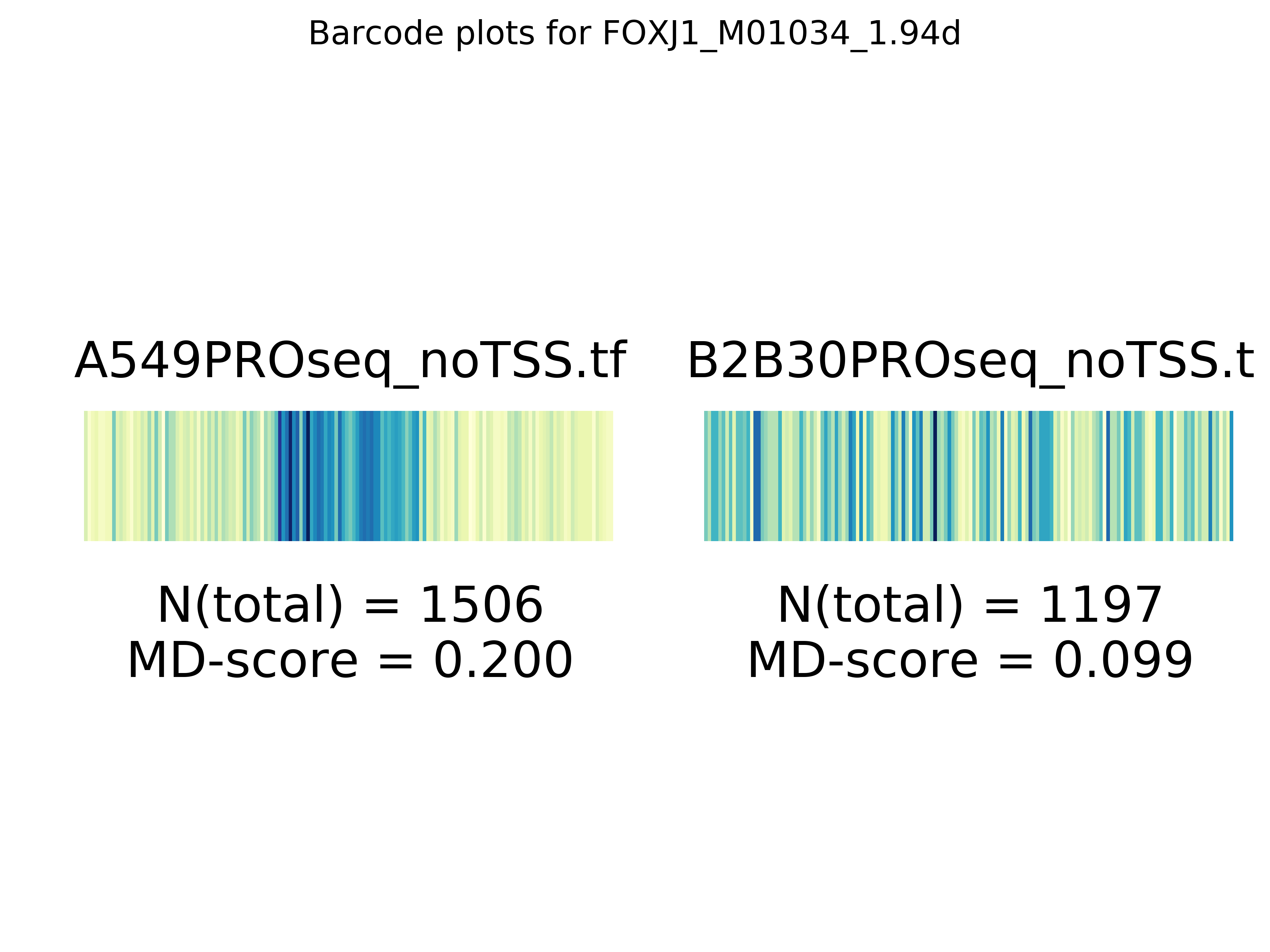

Supplement: Supplemental Data Set 1 [file jciinsight-6-144294-s076.zip › noTSS/best_curated_Human_TFs_p1e-5_grch38/A549_vs_B2B/FOXJ1_M01034_1.94d_barcode_A549PROseq_noTSS.tfit_merged_vs_B2B30PROseq_noTSS.tfit_merged.png]

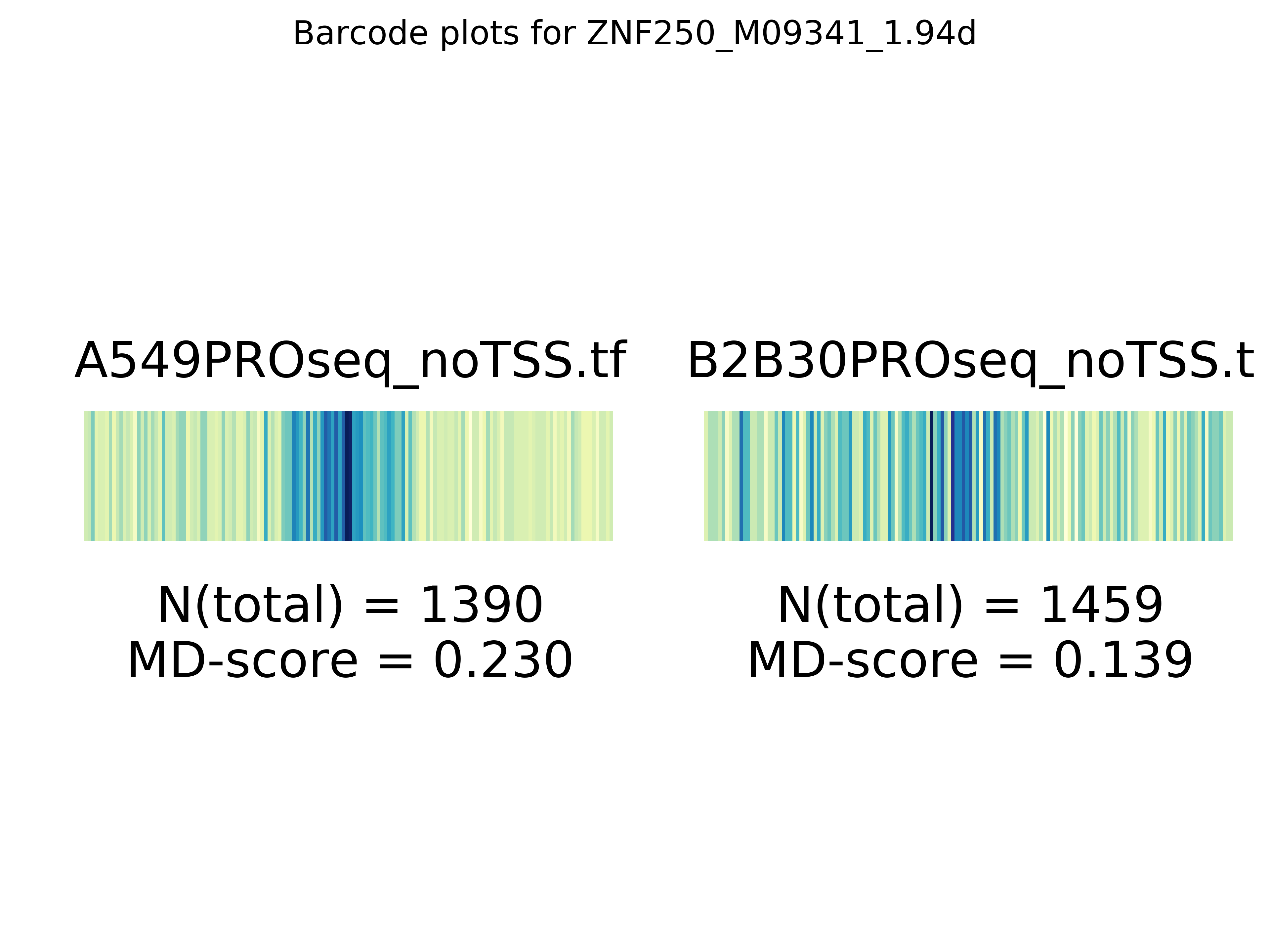

Supplement: Supplemental Data Set 1 [file jciinsight-6-144294-s076.zip › noTSS/best_curated_Human_TFs_p1e-5_grch38/A549_vs_B2B/ZNF250_M09341_1.94d_barcode_A549PROseq_noTSS.tfit_merged_vs_B2B30PROseq_noTSS.tfit_merged.png]

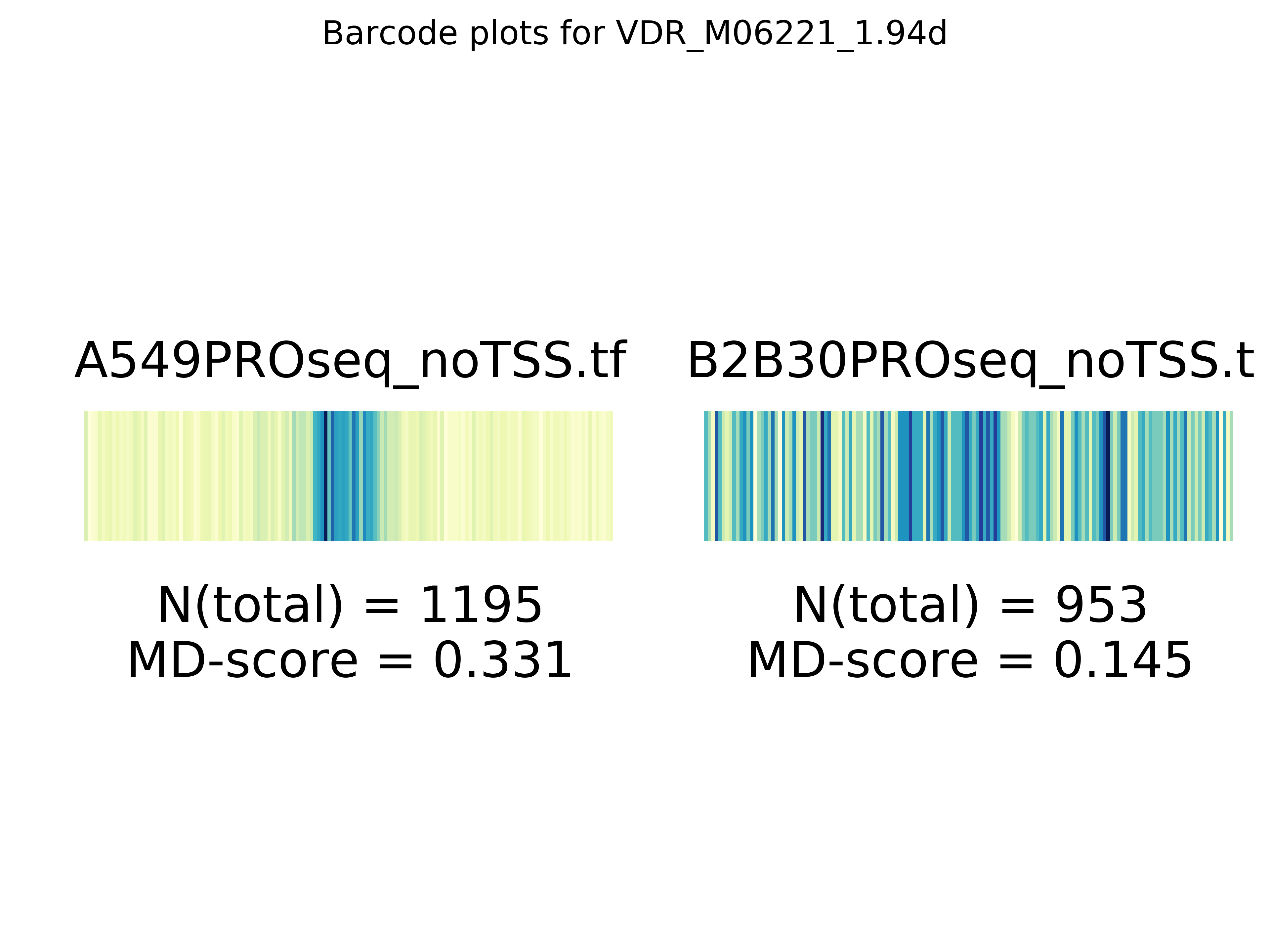

Supplement: Supplemental Data Set 1 [file jciinsight-6-144294-s076.zip › noTSS/best_curated_Human_TFs_p1e-5_grch38/A549_vs_B2B/VDR_M06221_1.94d_barcode_A549PROseq_noTSS.tfit_merged_vs_B2B30PROseq_noTSS.tfit_merged.png]

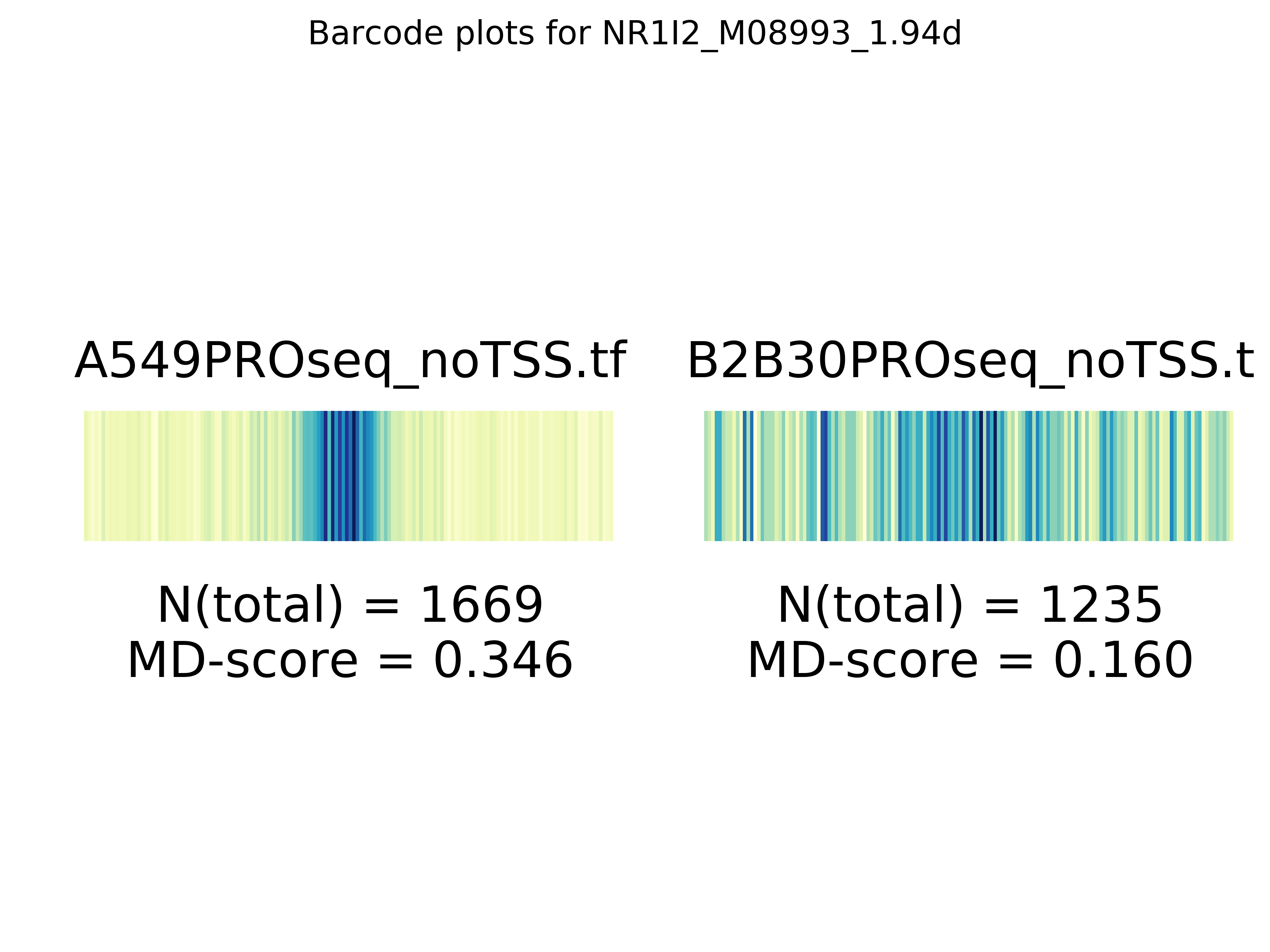

Supplement: Supplemental Data Set 1 [file jciinsight-6-144294-s076.zip › noTSS/best_curated_Human_TFs_p1e-5_grch38/A549_vs_B2B/NR1I2_M08993_1.94d_barcode_A549PROseq_noTSS.tfit_merged_vs_B2B30PROseq_noTSS.tfit_merged.png]

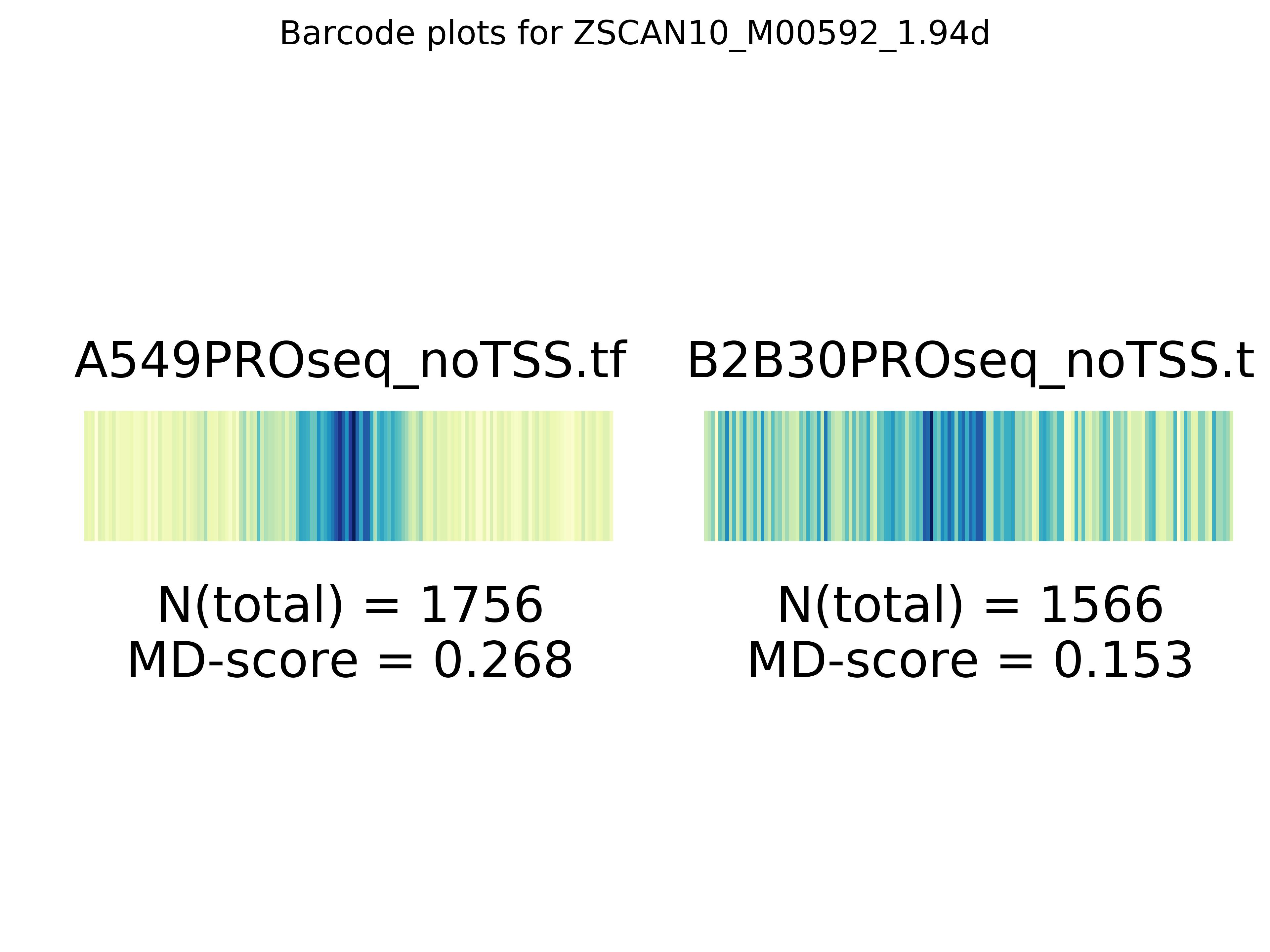

Supplement: Supplemental Data Set 1 [file jciinsight-6-144294-s076.zip › noTSS/best_curated_Human_TFs_p1e-5_grch38/A549_vs_B2B/ZSCAN10_M00592_1.94d_barcode_A549PROseq_noTSS.tfit_merged_vs_B2B30PROseq_noTSS.tfit_merged.png]

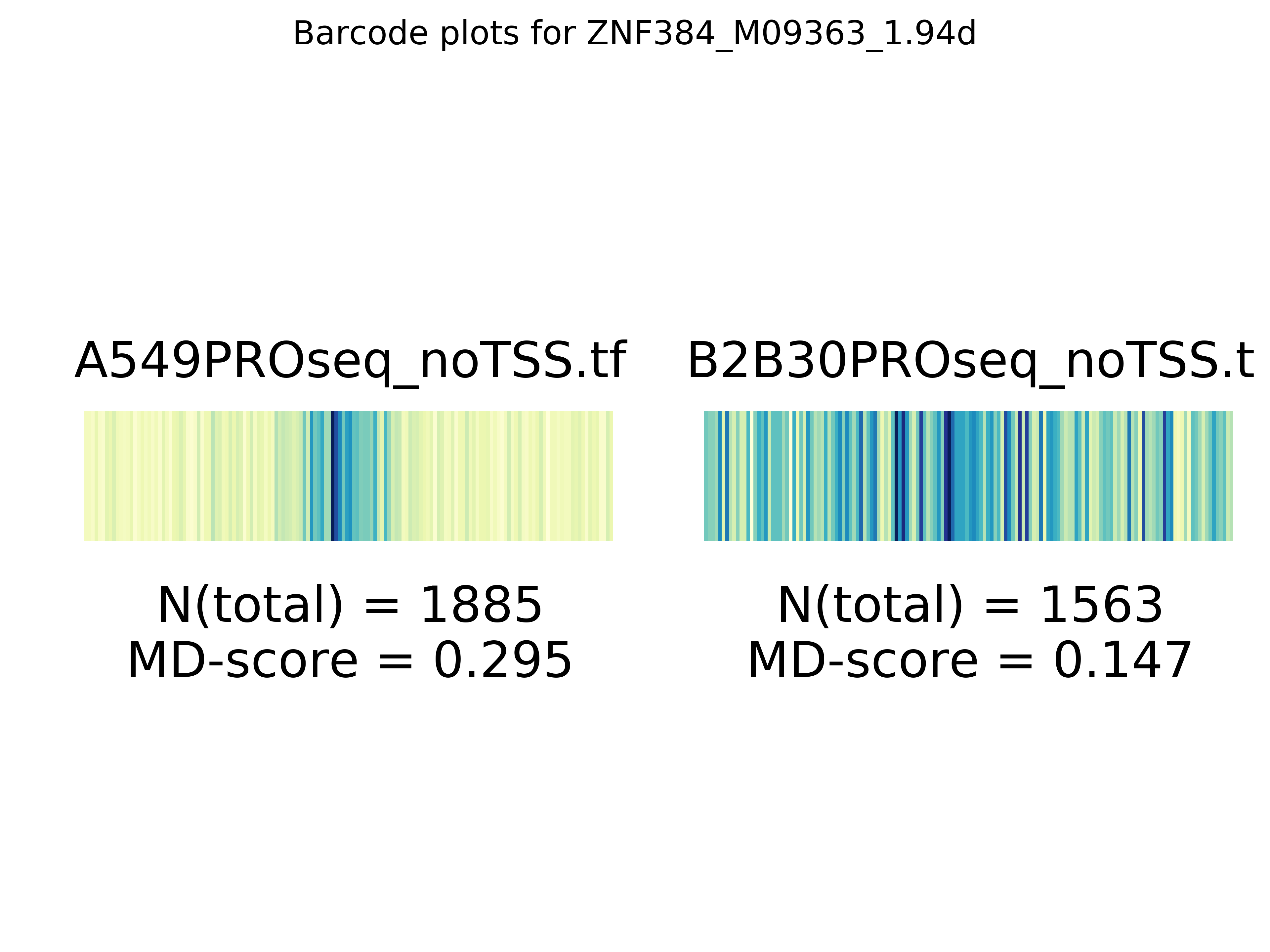

Supplement: Supplemental Data Set 1 [file jciinsight-6-144294-s076.zip › noTSS/best_curated_Human_TFs_p1e-5_grch38/A549_vs_B2B/ZNF384_M09363_1.94d_barcode_A549PROseq_noTSS.tfit_merged_vs_B2B30PROseq_noTSS.tfit_merged.png]

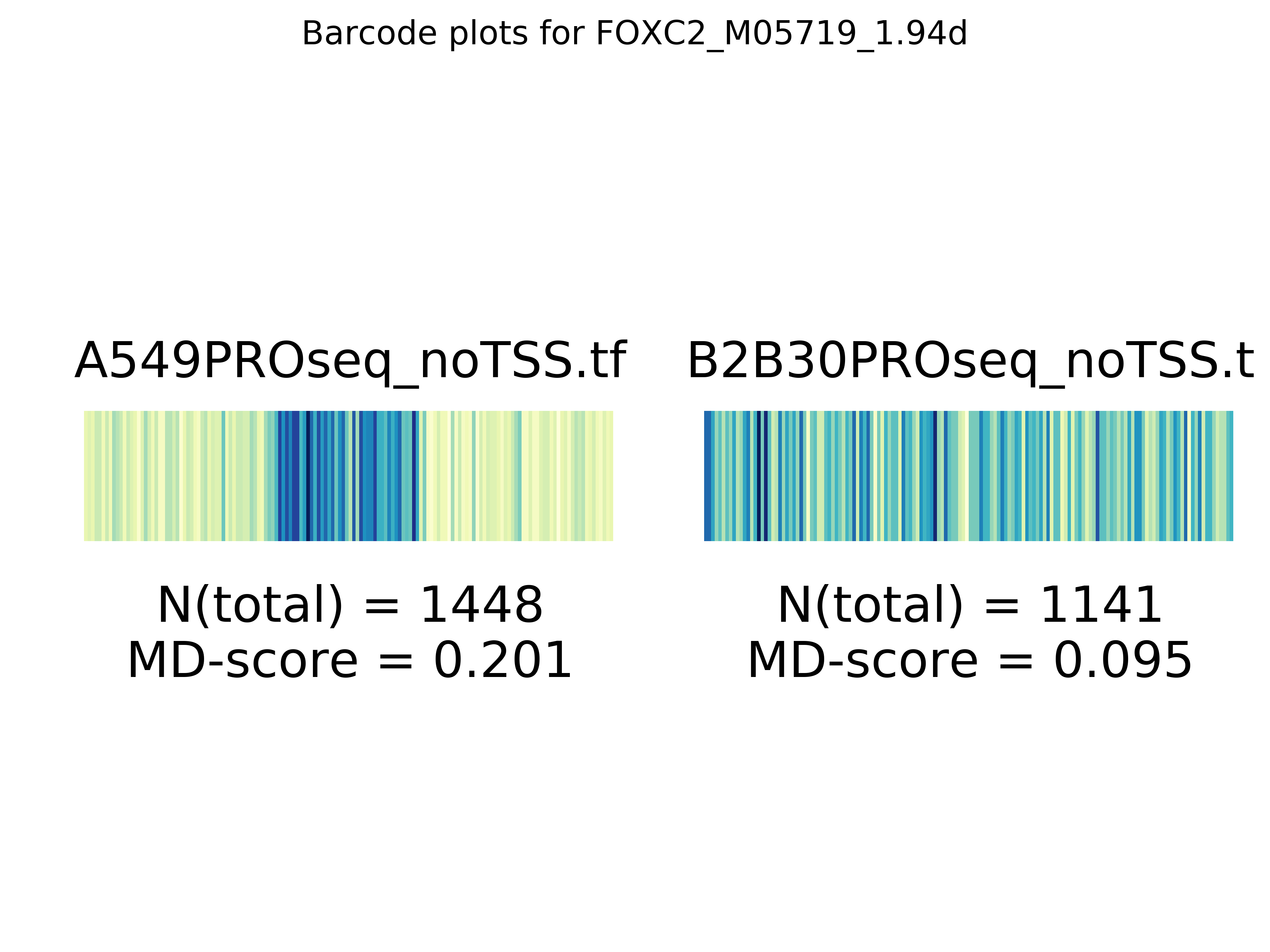

Supplement: Supplemental Data Set 1 [file jciinsight-6-144294-s076.zip › noTSS/best_curated_Human_TFs_p1e-5_grch38/A549_vs_B2B/FOXC2_M05719_1.94d_barcode_A549PROseq_noTSS.tfit_merged_vs_B2B30PROseq_noTSS.tfit_merged.png]

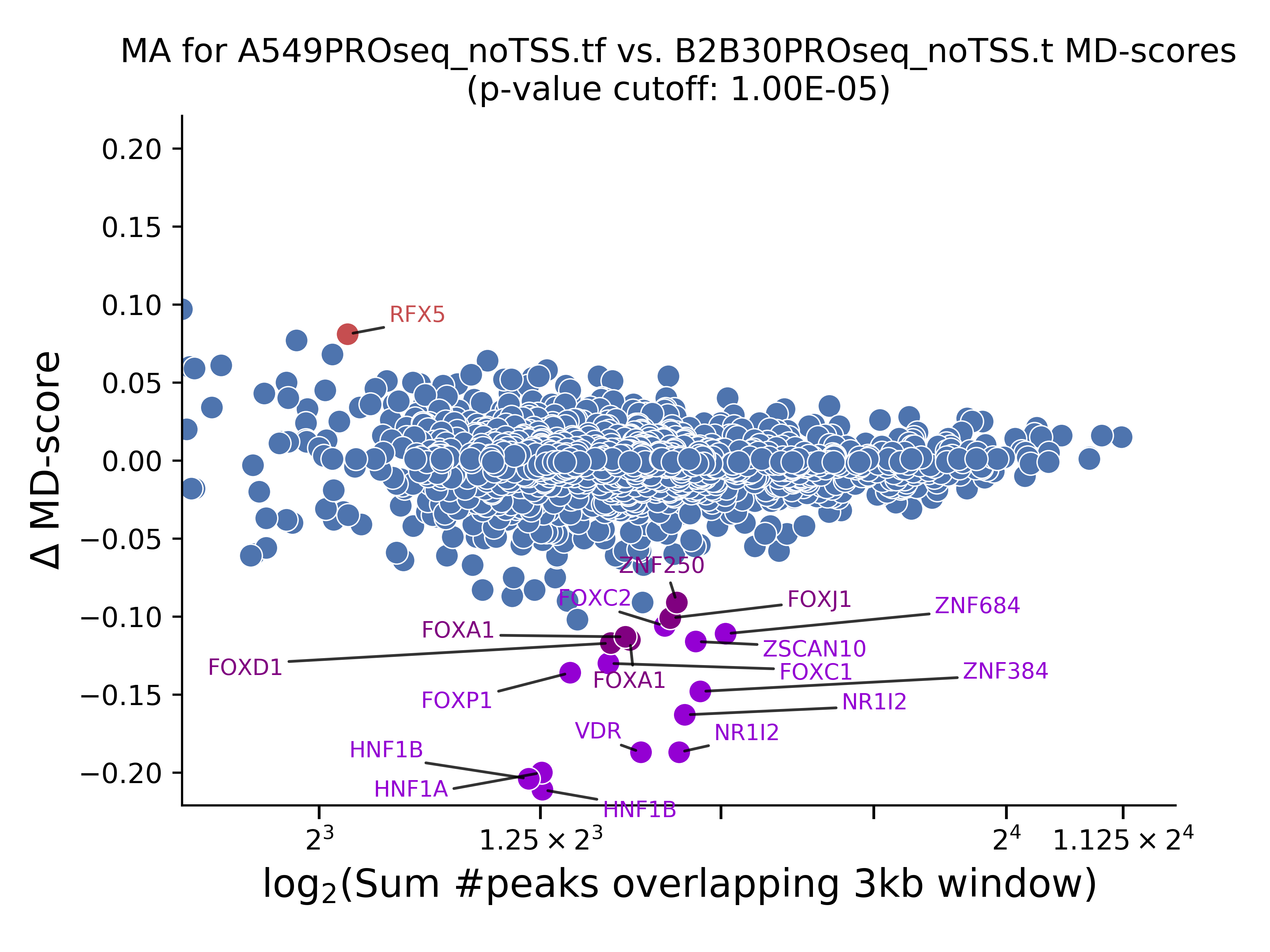

Supplement: Supplemental Data Set 1 [file jciinsight-6-144294-s076.zip › noTSS/best_curated_Human_TFs_p1e-5_grch38/A549_vs_B2B/MA_A549PROseq_noTSS.tfit_merged_to_B2B30PROseq_noTSS.tfit_merged_md_score.png]

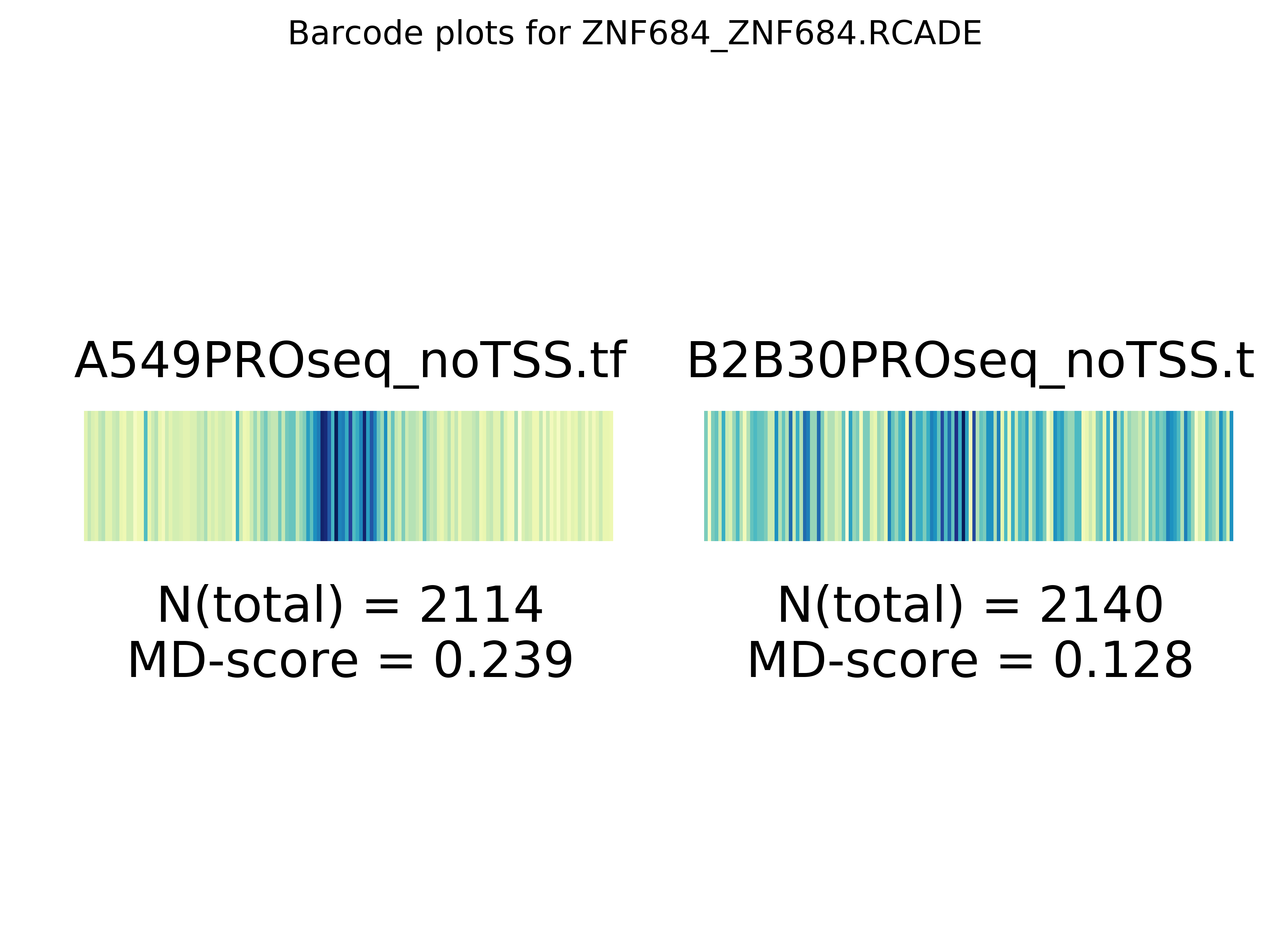

Supplement: Supplemental Data Set 1 [file jciinsight-6-144294-s076.zip › noTSS/best_curated_Human_TFs_p1e-5_grch38/A549_vs_B2B/ZNF684_ZNF684.RCADE_barcode_A549PROseq_noTSS.tfit_merged_vs_B2B30PROseq_noTSS.tfit_merged.png]

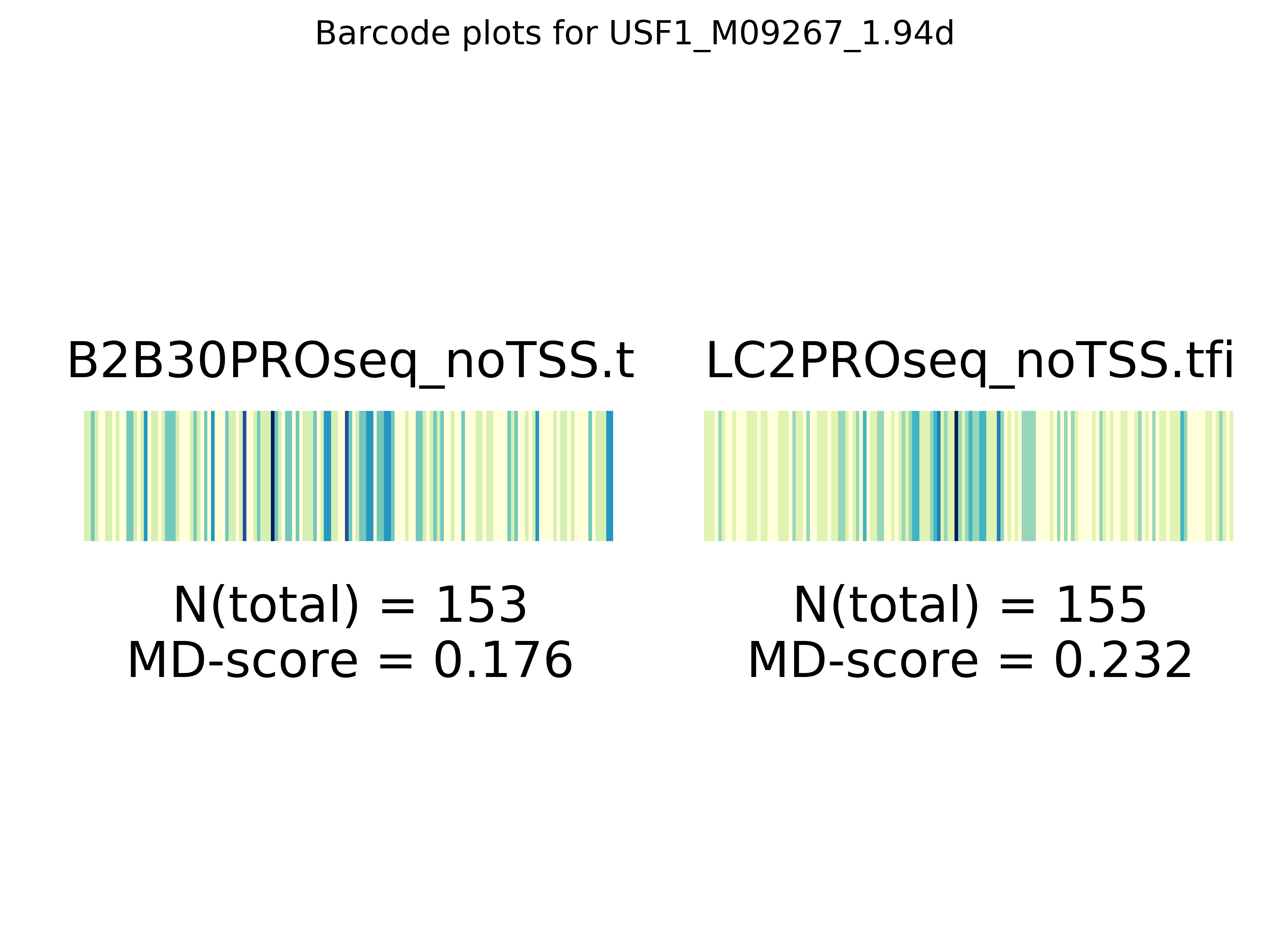

Supplement: Supplemental Data Set 1 [file jciinsight-6-144294-s076.zip › noTSS/best_curated_Human_TFs_p1e-6_grch38/B2B_vs_LC2/USF1_M09267_1.94d_barcode_B2B30PROseq_noTSS.tfit_merged_vs_LC2PROseq_noTSS.tfit_merged.png]

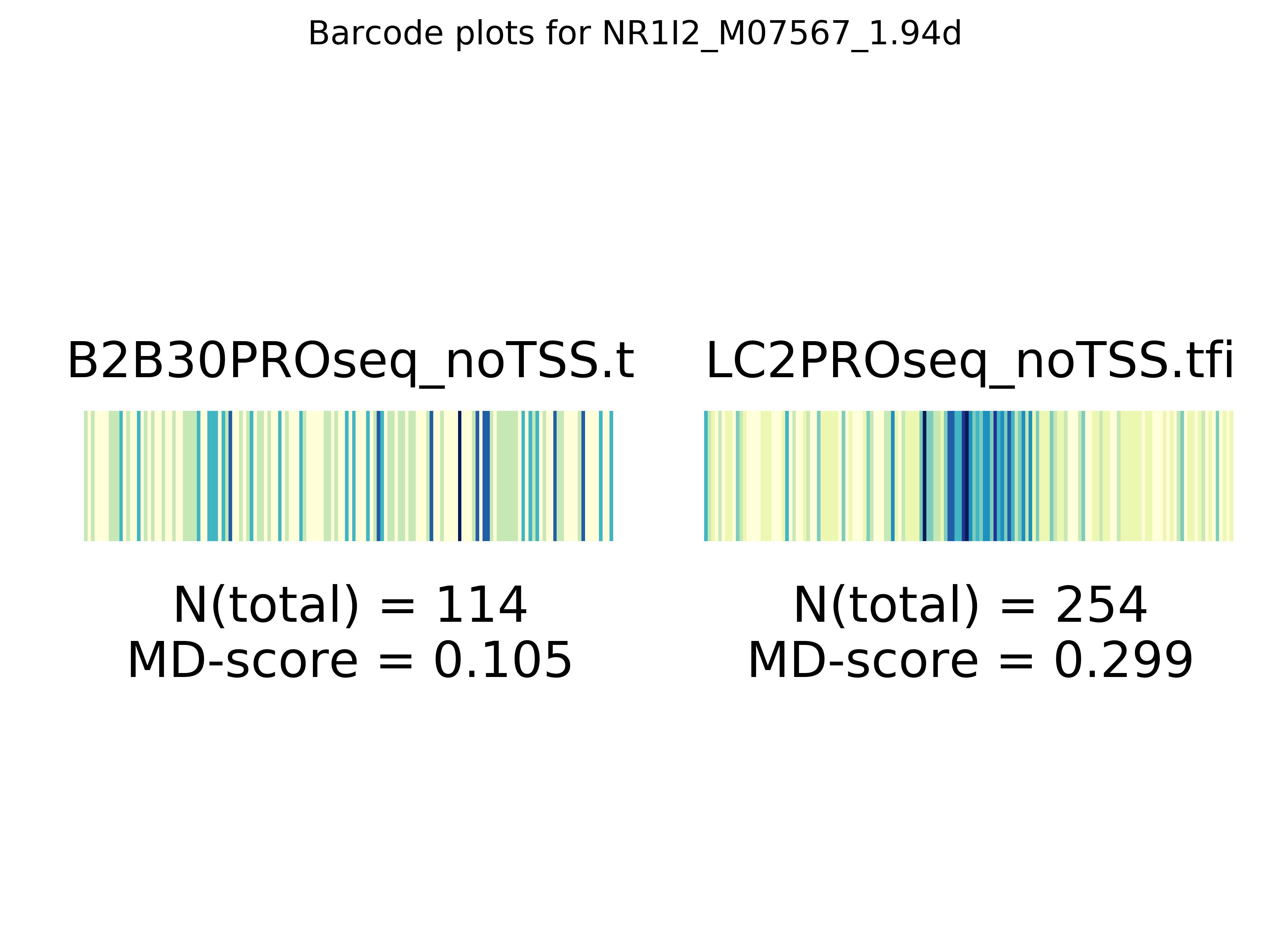

Supplement: Supplemental Data Set 1 [file jciinsight-6-144294-s076.zip › noTSS/best_curated_Human_TFs_p1e-6_grch38/B2B_vs_LC2/NR1I2_M07567_1.94d_barcode_B2B30PROseq_noTSS.tfit_merged_vs_LC2PROseq_noTSS.tfit_merged.png]

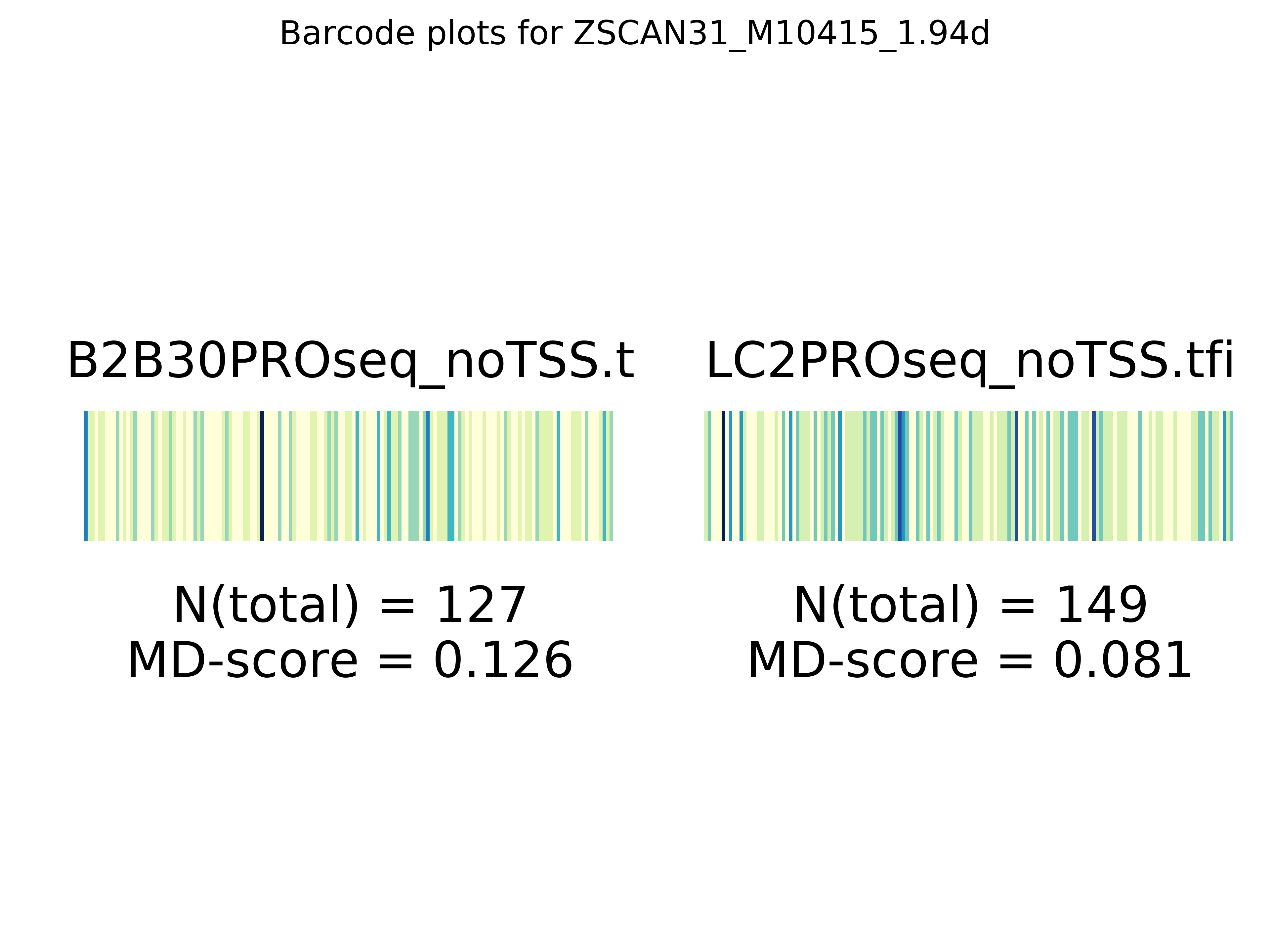

Supplement: Supplemental Data Set 1 [file jciinsight-6-144294-s076.zip › noTSS/best_curated_Human_TFs_p1e-6_grch38/B2B_vs_LC2/ZSCAN31_M10415_1.94d_barcode_B2B30PROseq_noTSS.tfit_merged_vs_LC2PROseq_noTSS.tfit_merged.png]

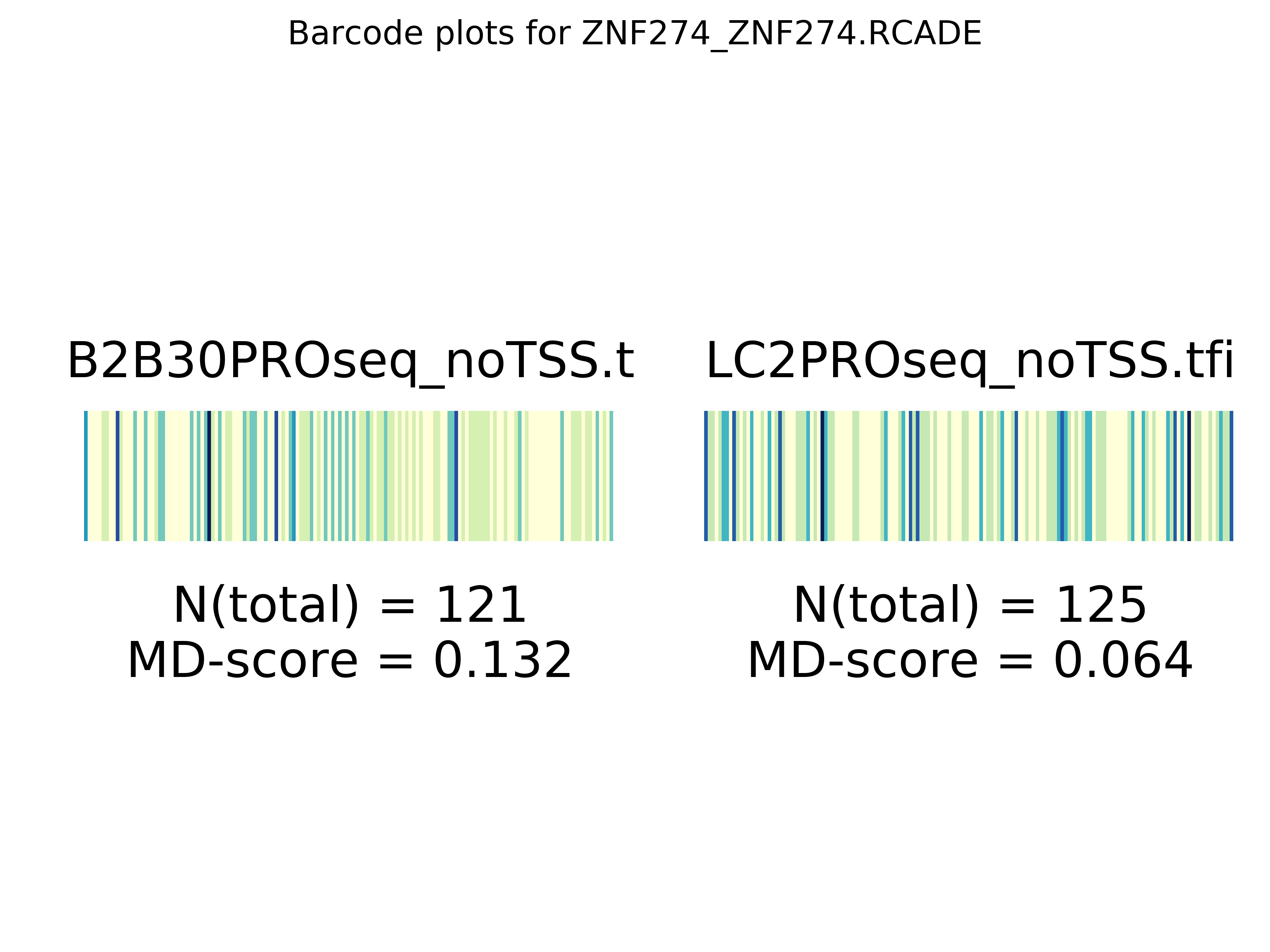

Supplement: Supplemental Data Set 1 [file jciinsight-6-144294-s076.zip › noTSS/best_curated_Human_TFs_p1e-6_grch38/B2B_vs_LC2/ZNF274_ZNF274.RCADE_barcode_B2B30PROseq_noTSS.tfit_merged_vs_LC2PROseq_noTSS.tfit_merged.png]

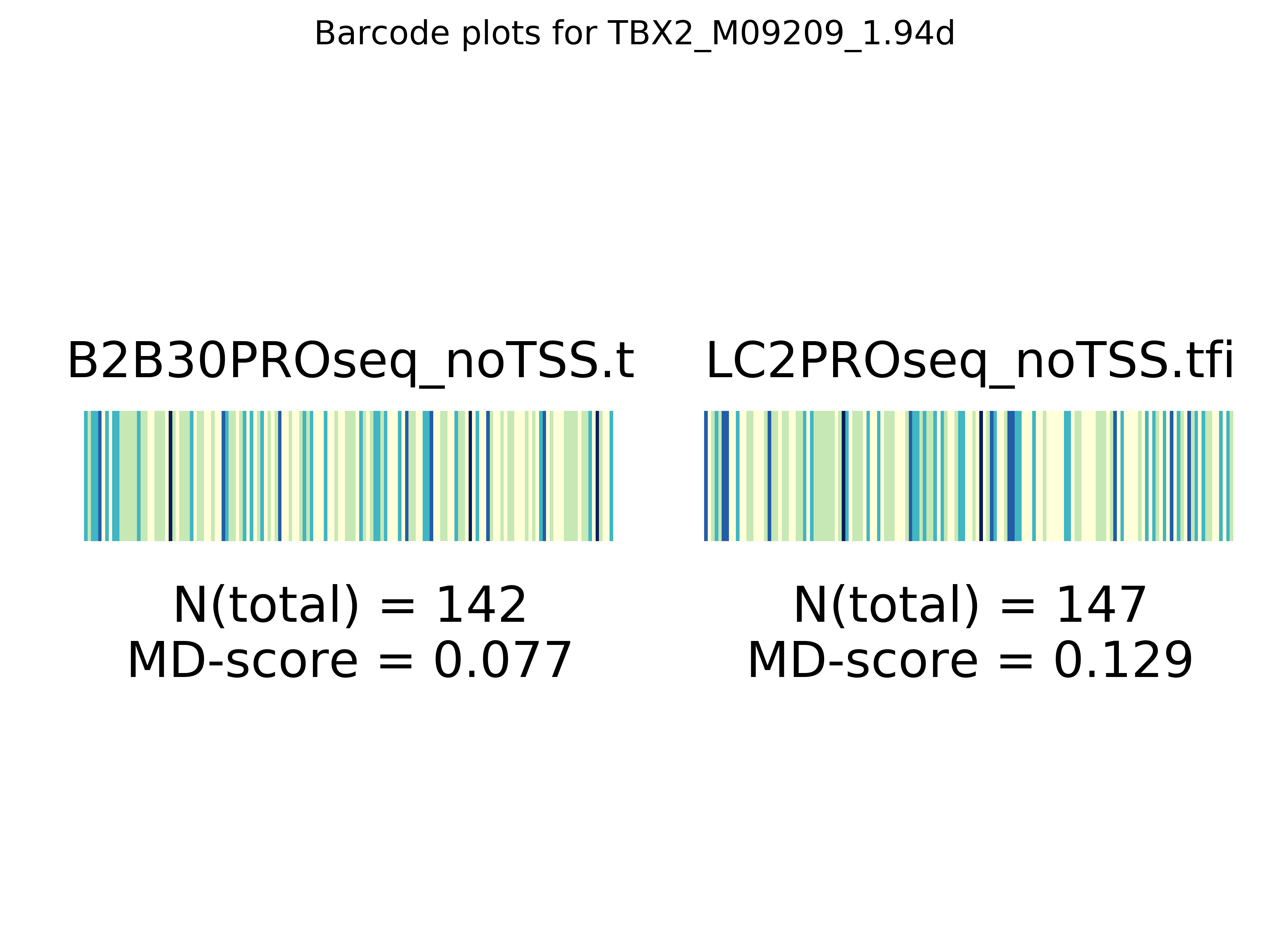

Supplement: Supplemental Data Set 1 [file jciinsight-6-144294-s076.zip › noTSS/best_curated_Human_TFs_p1e-6_grch38/B2B_vs_LC2/TBX2_M09209_1.94d_barcode_B2B30PROseq_noTSS.tfit_merged_vs_LC2PROseq_noTSS.tfit_merged.png]

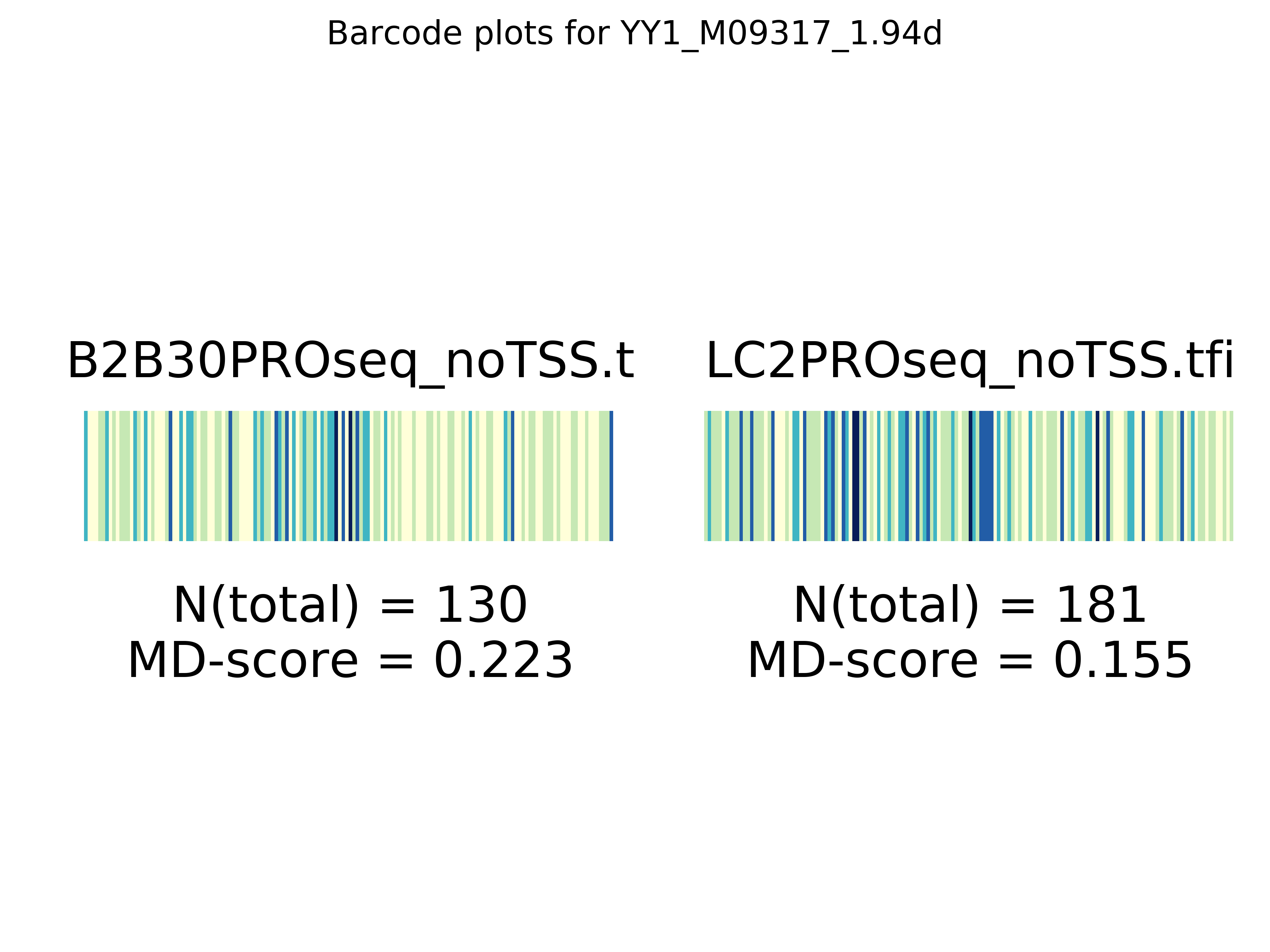

Supplement: Supplemental Data Set 1 [file jciinsight-6-144294-s076.zip › noTSS/best_curated_Human_TFs_p1e-6_grch38/B2B_vs_LC2/YY1_M09317_1.94d_barcode_B2B30PROseq_noTSS.tfit_merged_vs_LC2PROseq_noTSS.tfit_merged.png]

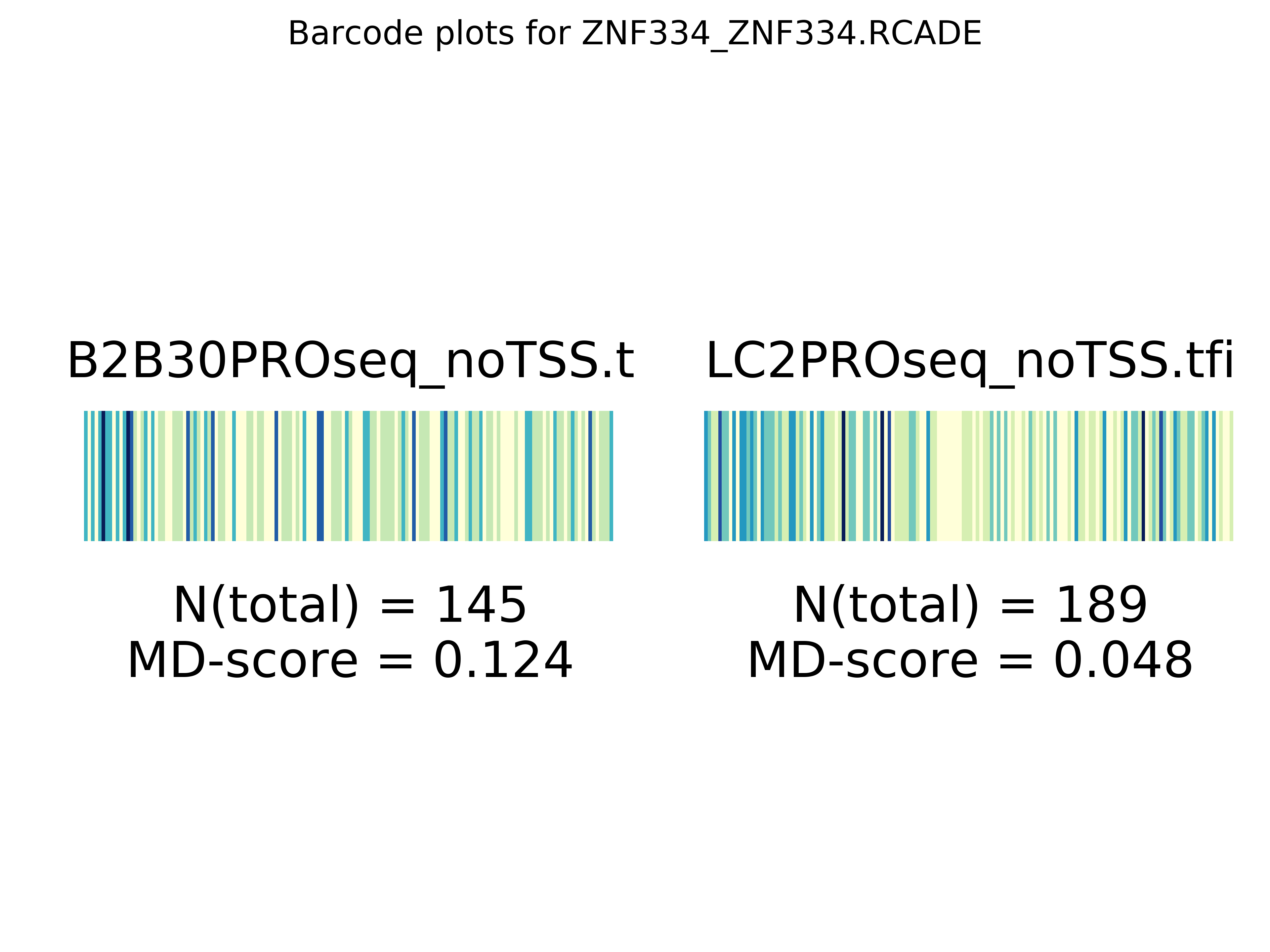

Supplement: Supplemental Data Set 1 [file jciinsight-6-144294-s076.zip › noTSS/best_curated_Human_TFs_p1e-6_grch38/B2B_vs_LC2/ZNF334_ZNF334.RCADE_barcode_B2B30PROseq_noTSS.tfit_merged_vs_LC2PROseq_noTSS.tfit_merged.png]

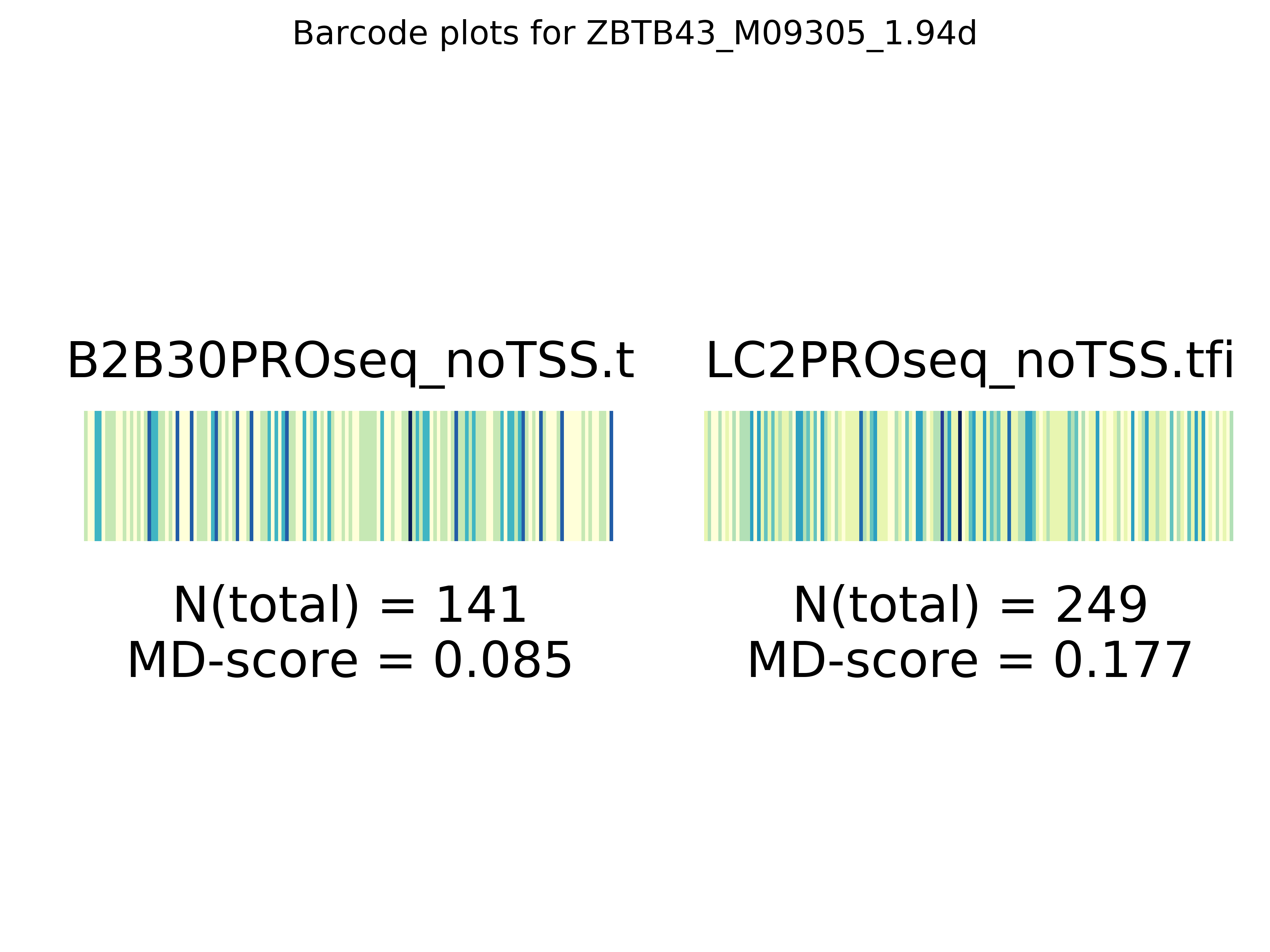

Supplement: Supplemental Data Set 1 [file jciinsight-6-144294-s076.zip › noTSS/best_curated_Human_TFs_p1e-6_grch38/B2B_vs_LC2/ZBTB43_M09305_1.94d_barcode_B2B30PROseq_noTSS.tfit_merged_vs_LC2PROseq_noTSS.tfit_merged.png]

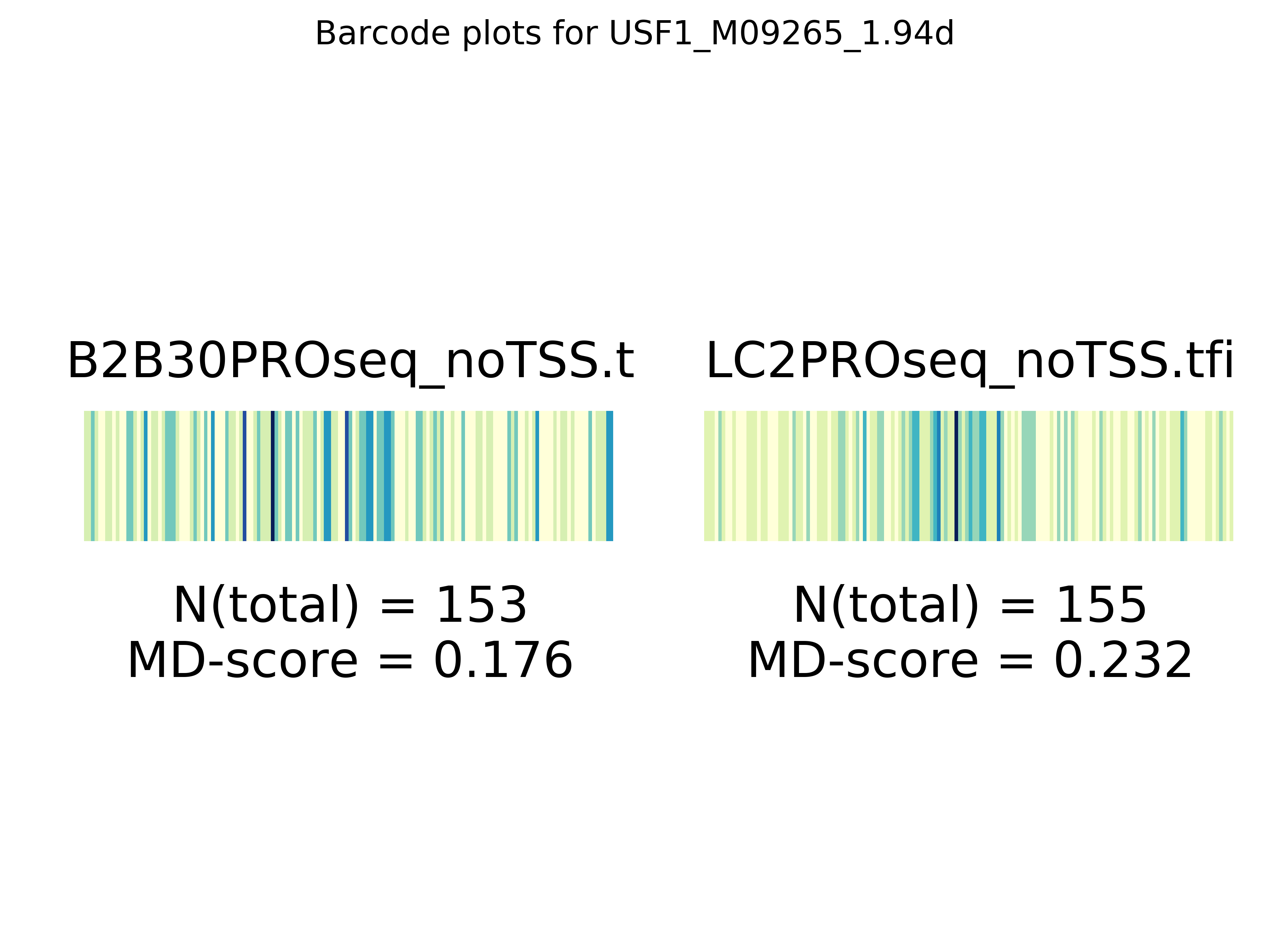

Supplement: Supplemental Data Set 1 [file jciinsight-6-144294-s076.zip › noTSS/best_curated_Human_TFs_p1e-6_grch38/B2B_vs_LC2/USF1_M09265_1.94d_barcode_B2B30PROseq_noTSS.tfit_merged_vs_LC2PROseq_noTSS.tfit_merged.png]

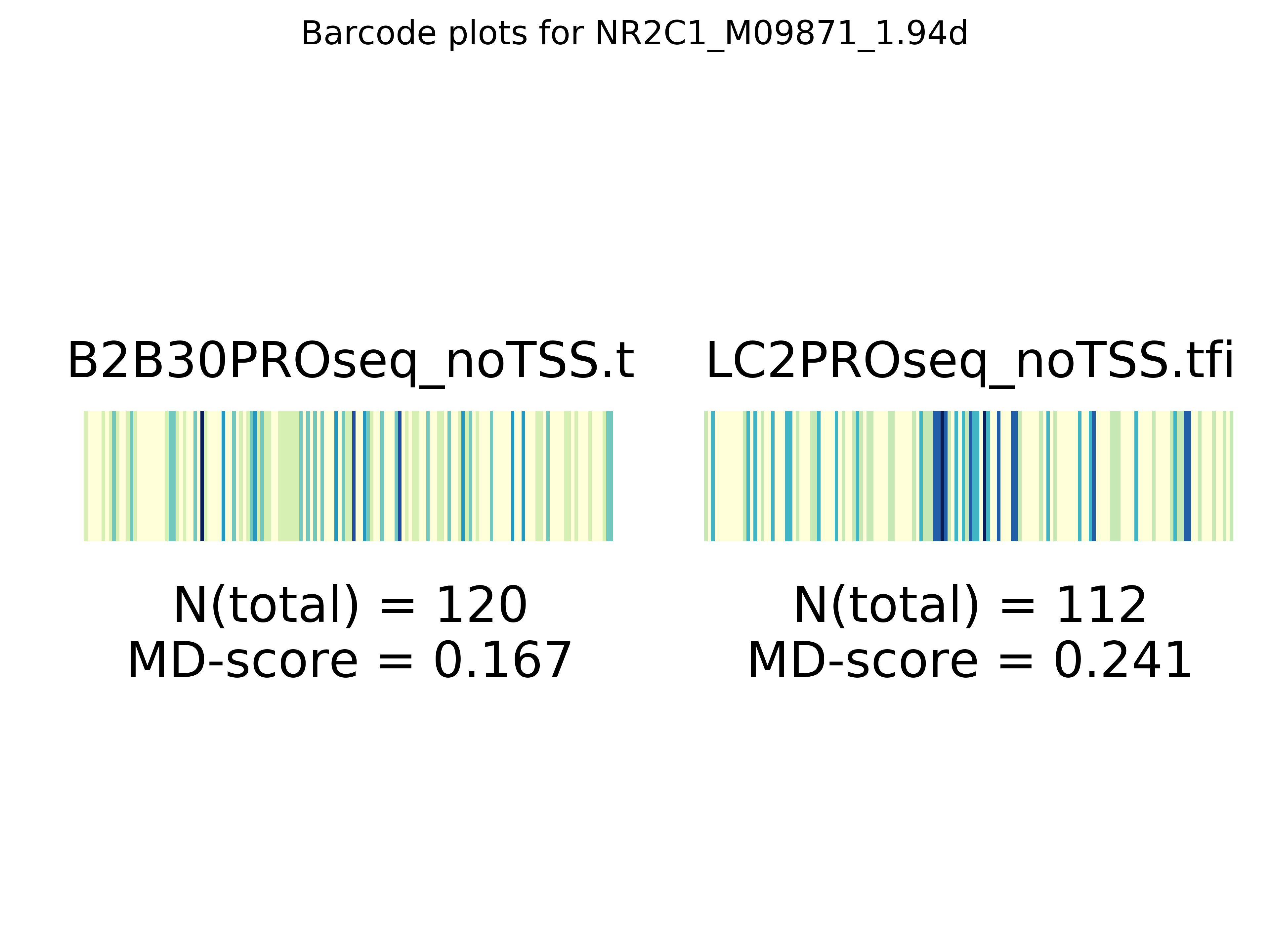

Supplement: Supplemental Data Set 1 [file jciinsight-6-144294-s076.zip › noTSS/best_curated_Human_TFs_p1e-6_grch38/B2B_vs_LC2/NR2C1_M09871_1.94d_barcode_B2B30PROseq_noTSS.tfit_merged_vs_LC2PROseq_noTSS.tfit_merged.png]

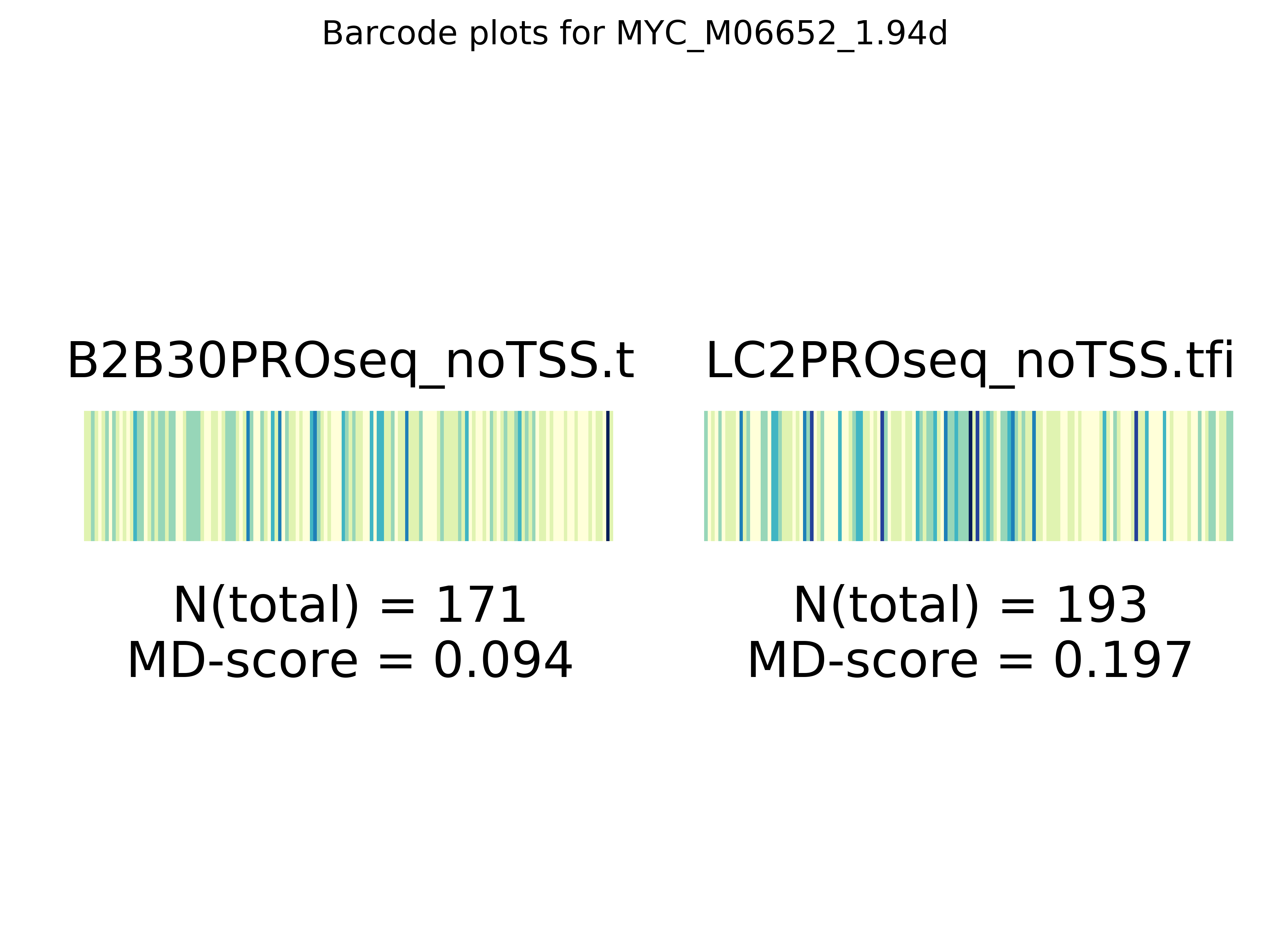

Supplement: Supplemental Data Set 1 [file jciinsight-6-144294-s076.zip › noTSS/best_curated_Human_TFs_p1e-6_grch38/B2B_vs_LC2/MYC_M06652_1.94d_barcode_B2B30PROseq_noTSS.tfit_merged_vs_LC2PROseq_noTSS.tfit_merged.png]

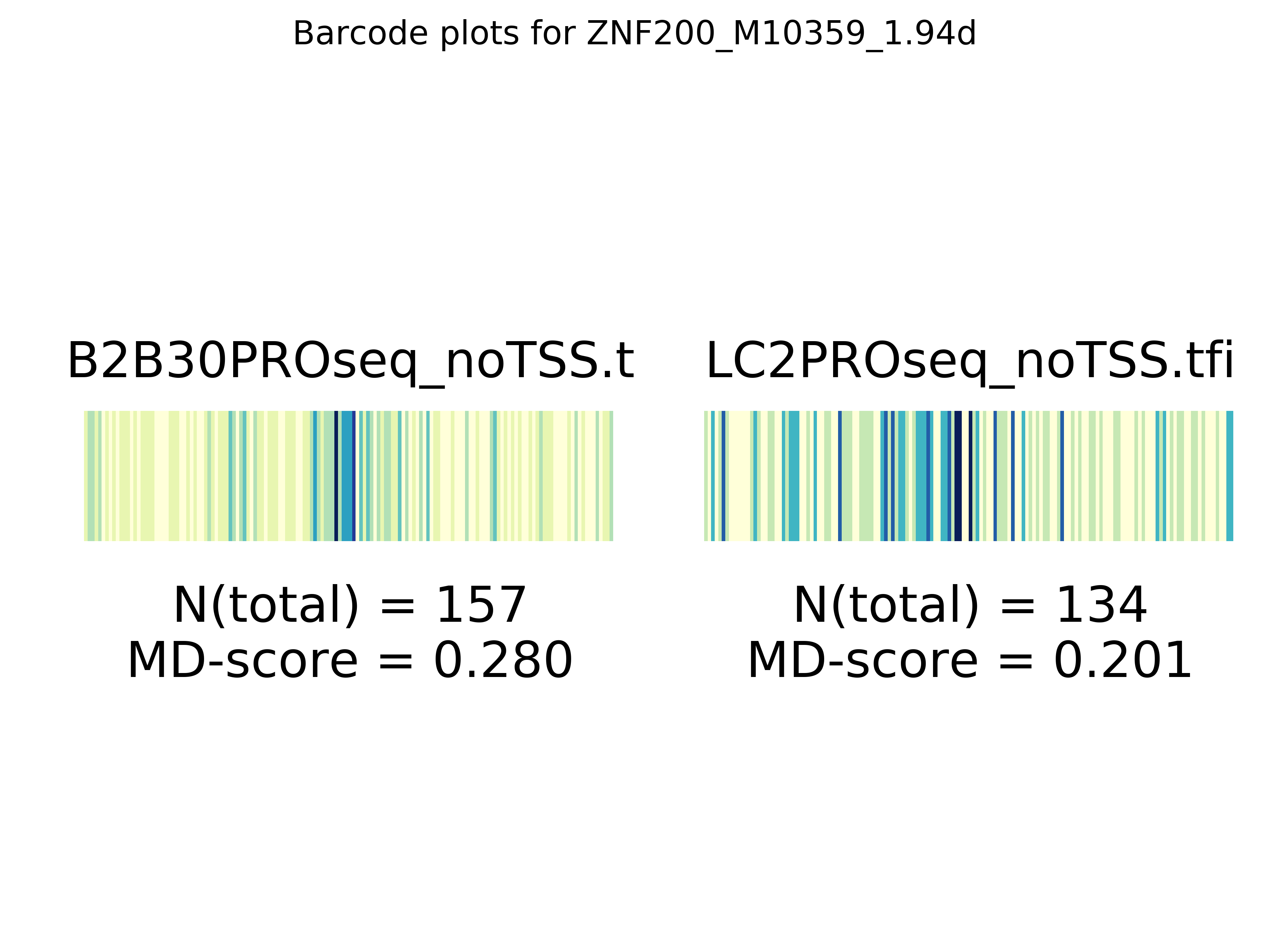

Supplement: Supplemental Data Set 1 [file jciinsight-6-144294-s076.zip › noTSS/best_curated_Human_TFs_p1e-6_grch38/B2B_vs_LC2/ZNF200_M10359_1.94d_barcode_B2B30PROseq_noTSS.tfit_merged_vs_LC2PROseq_noTSS.tfit_merged.png]

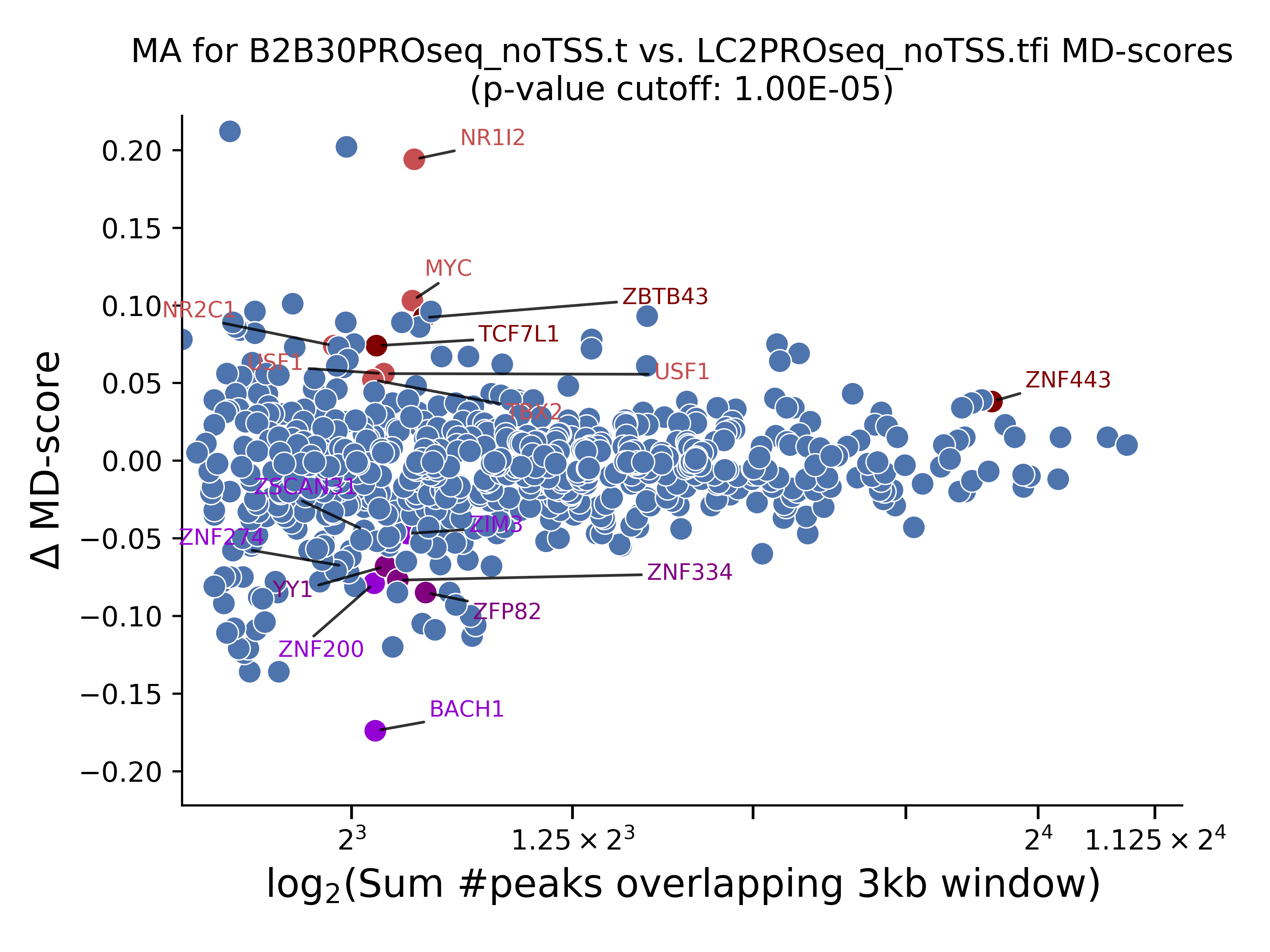

Supplement: Supplemental Data Set 1 [file jciinsight-6-144294-s076.zip › noTSS/best_curated_Human_TFs_p1e-6_grch38/B2B_vs_LC2/MA_B2B30PROseq_noTSS.tfit_merged_to_LC2PROseq_noTSS.tfit_merged_md_score.png]

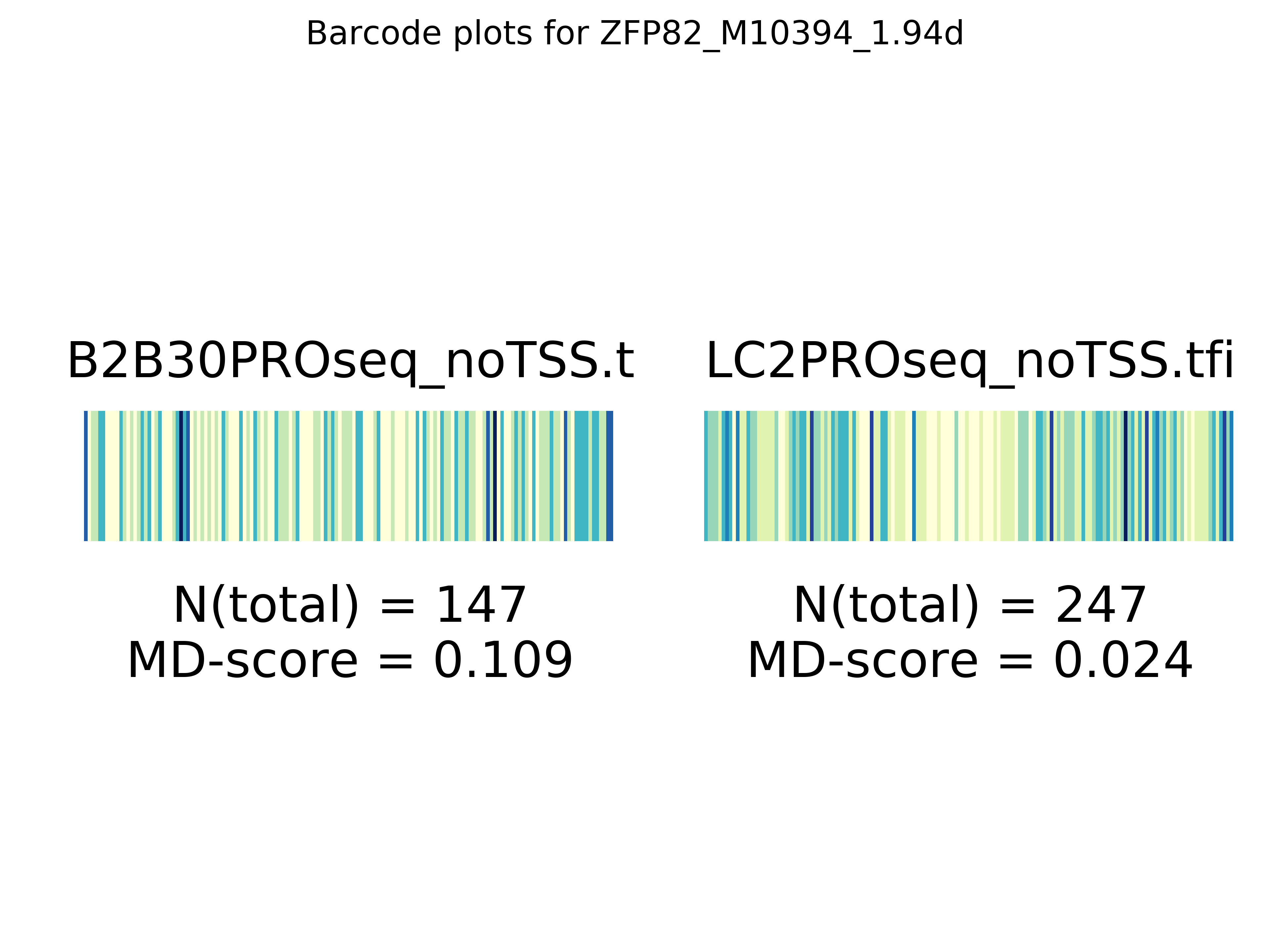

Supplement: Supplemental Data Set 1 [file jciinsight-6-144294-s076.zip › noTSS/best_curated_Human_TFs_p1e-6_grch38/B2B_vs_LC2/ZFP82_M10394_1.94d_barcode_B2B30PROseq_noTSS.tfit_merged_vs_LC2PROseq_noTSS.tfit_merged.png]

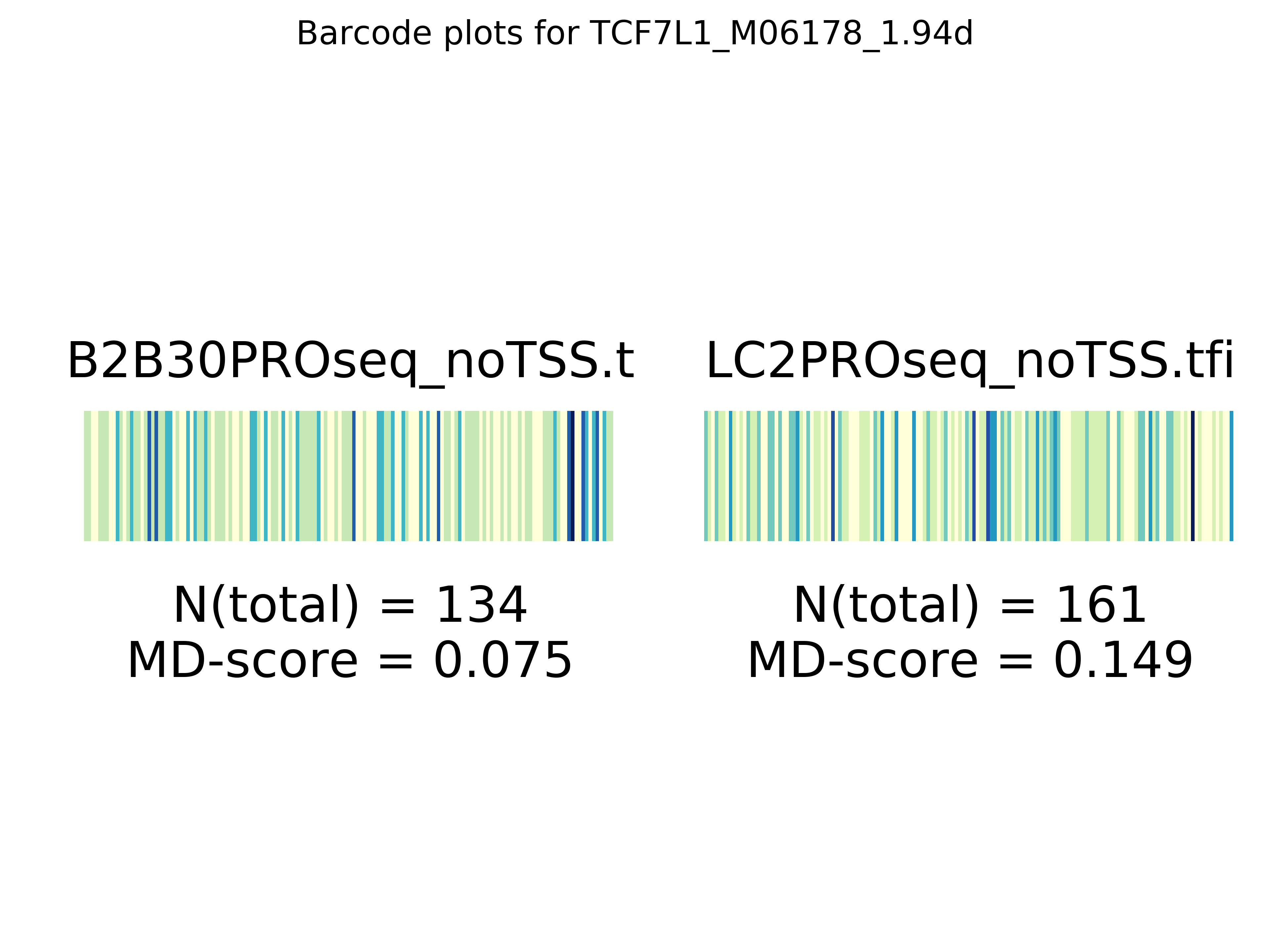

Supplement: Supplemental Data Set 1 [file jciinsight-6-144294-s076.zip › noTSS/best_curated_Human_TFs_p1e-6_grch38/B2B_vs_LC2/TCF7L1_M06178_1.94d_barcode_B2B30PROseq_noTSS.tfit_merged_vs_LC2PROseq_noTSS.tfit_merged.png]

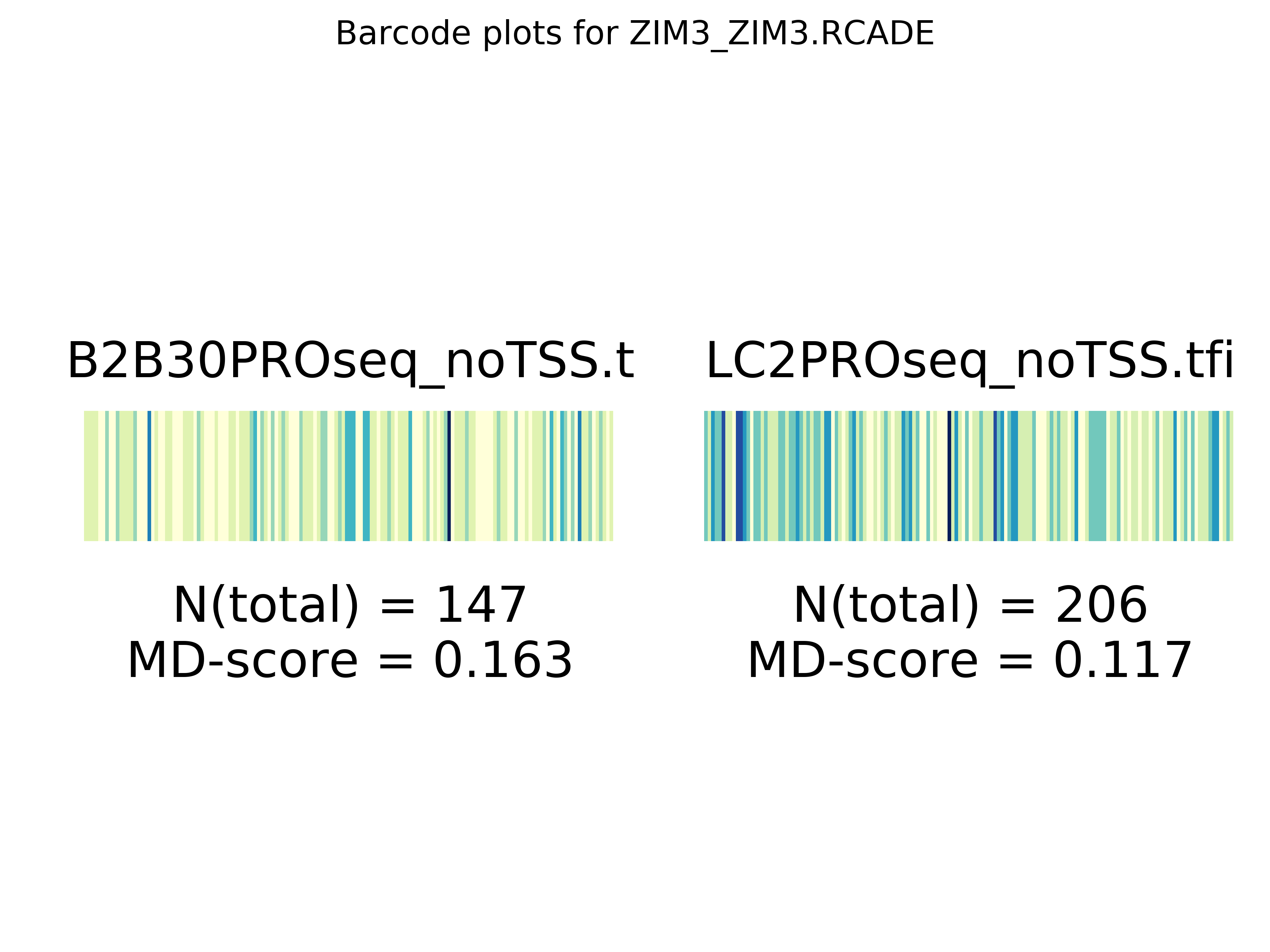

Supplement: Supplemental Data Set 1 [file jciinsight-6-144294-s076.zip › noTSS/best_curated_Human_TFs_p1e-6_grch38/B2B_vs_LC2/ZIM3_ZIM3.RCADE_barcode_B2B30PROseq_noTSS.tfit_merged_vs_LC2PROseq_noTSS.tfit_merged.png]

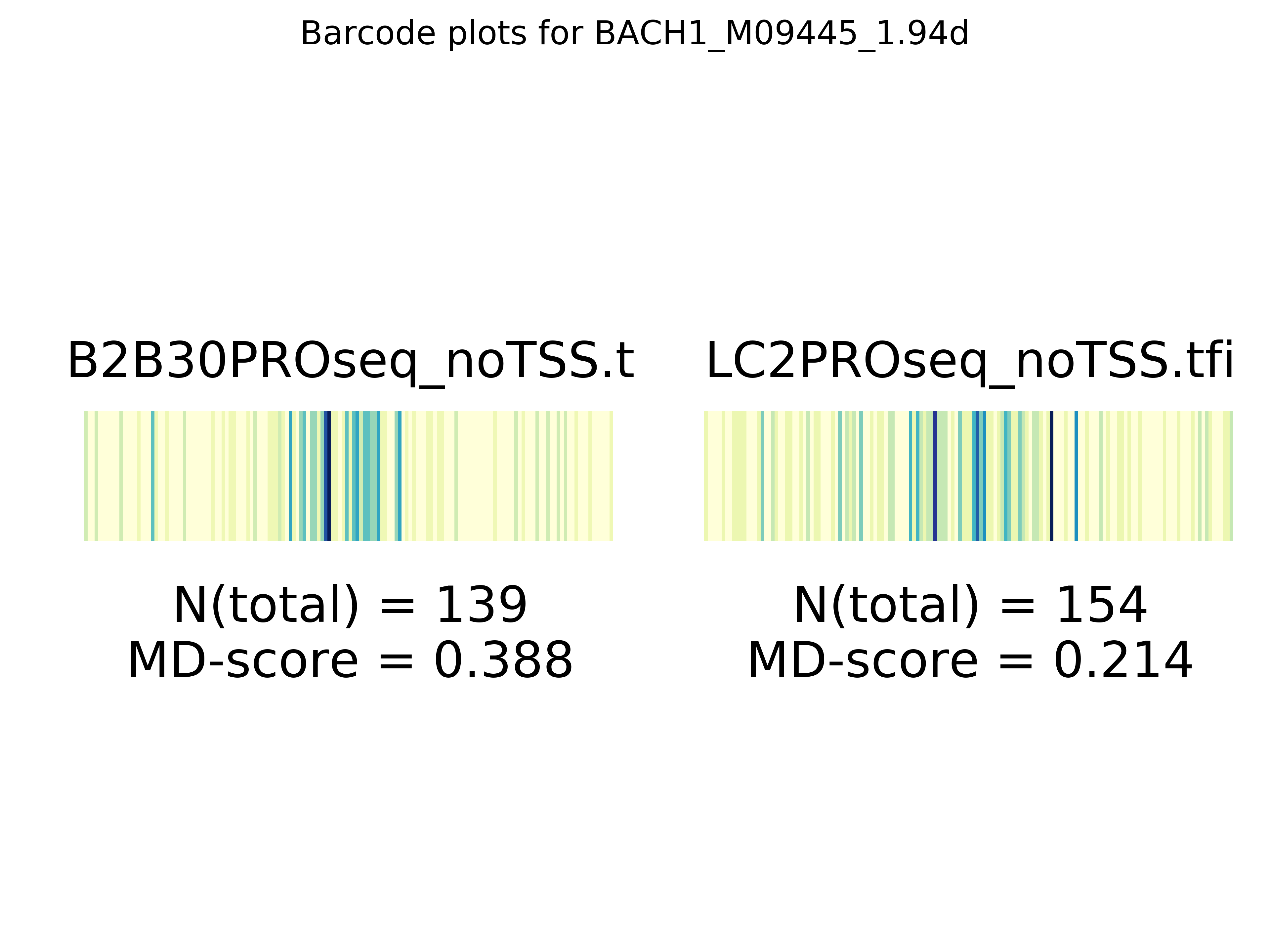

Supplement: Supplemental Data Set 1 [file jciinsight-6-144294-s076.zip › noTSS/best_curated_Human_TFs_p1e-6_grch38/B2B_vs_LC2/BACH1_M09445_1.94d_barcode_B2B30PROseq_noTSS.tfit_merged_vs_LC2PROseq_noTSS.tfit_merged.png]

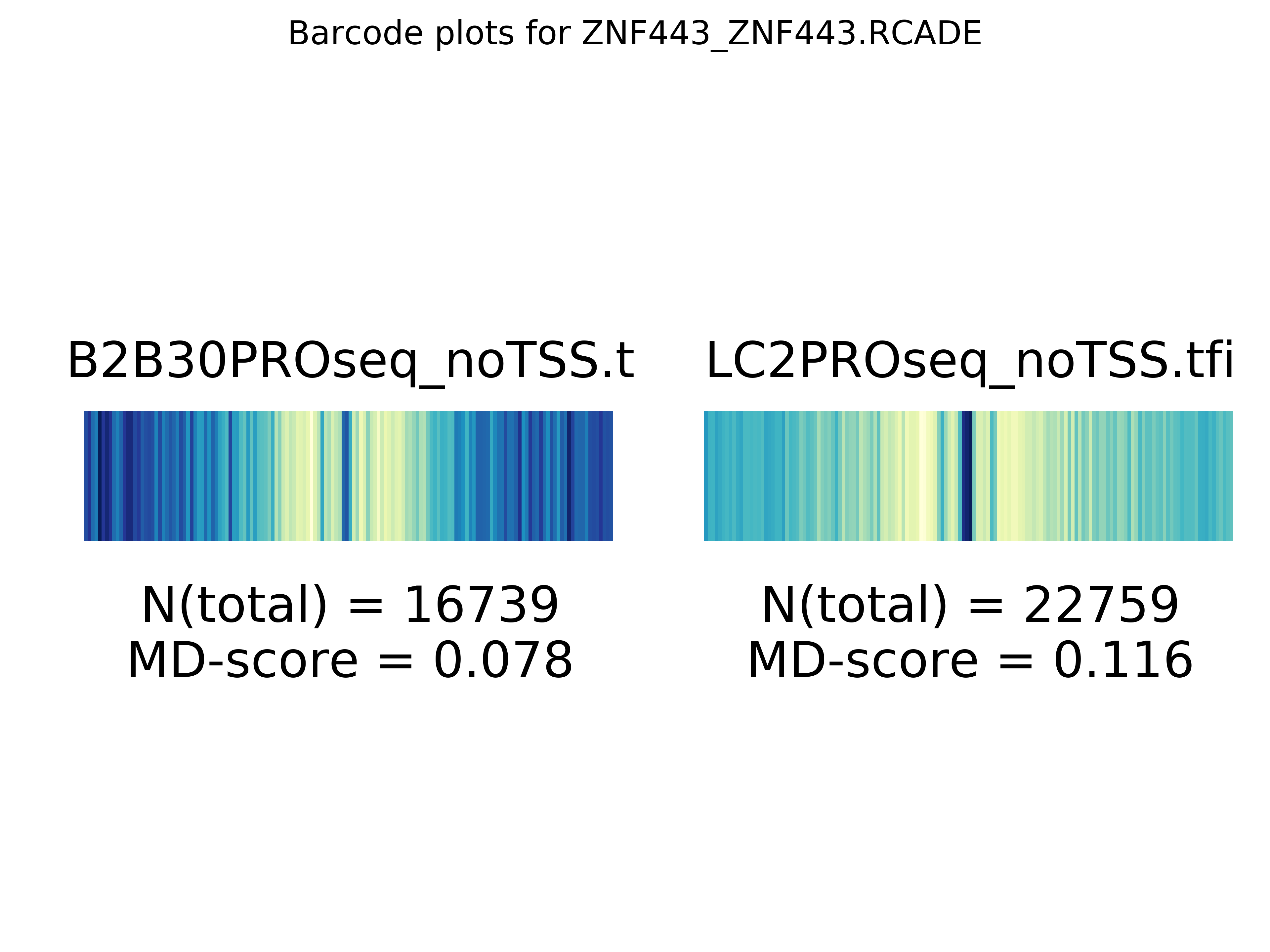

Supplement: Supplemental Data Set 1 [file jciinsight-6-144294-s076.zip › noTSS/best_curated_Human_TFs_p1e-6_grch38/B2B_vs_LC2/ZNF443_ZNF443.RCADE_barcode_B2B30PROseq_noTSS.tfit_merged_vs_LC2PROseq_noTSS.tfit_merged.png]

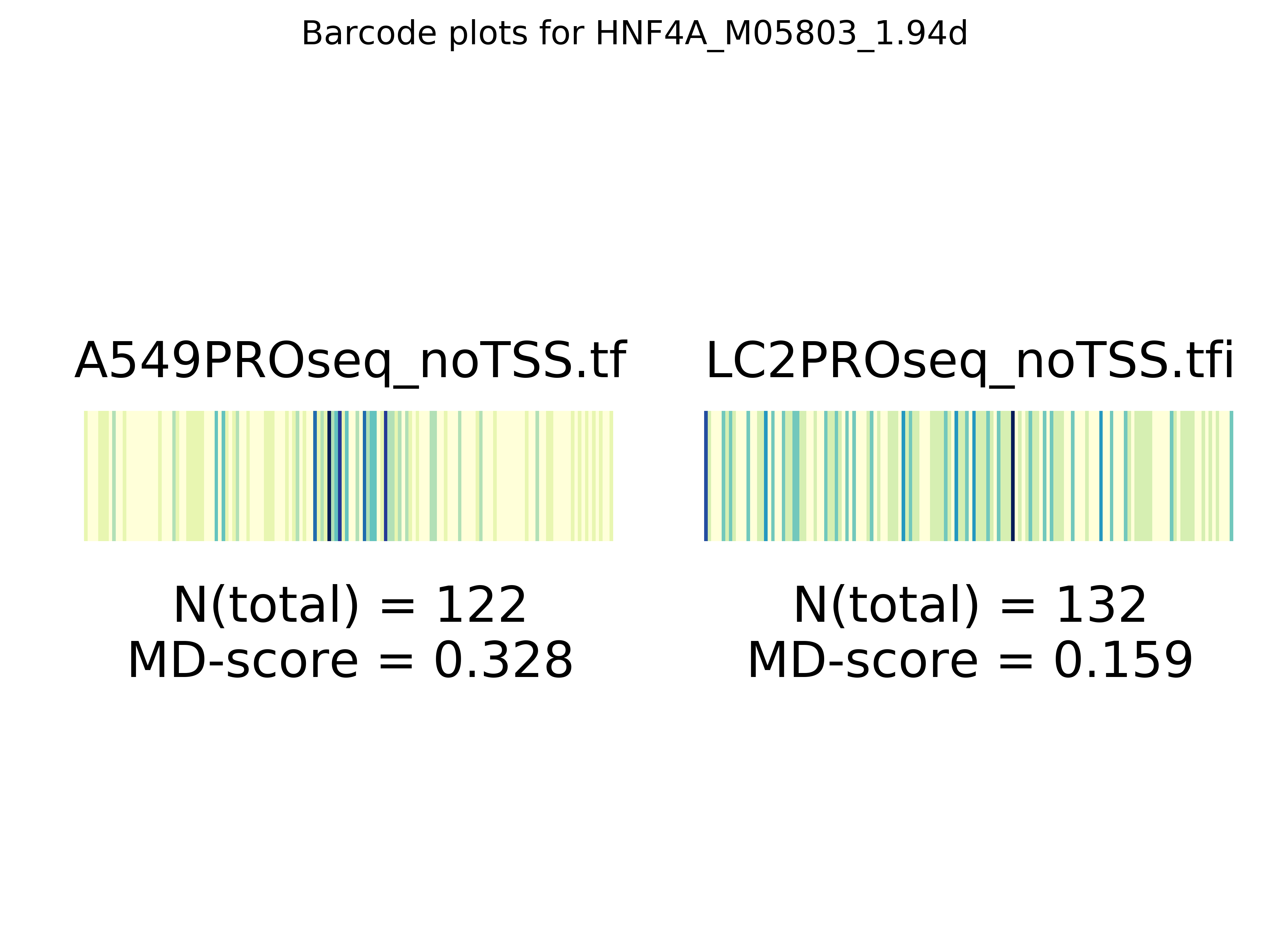

Supplement: Supplemental Data Set 1 [file jciinsight-6-144294-s076.zip › noTSS/best_curated_Human_TFs_p1e-6_grch38/A549_vs_LC2/HNF4A_M05803_1.94d_barcode_A549PROseq_noTSS.tfit_merged_vs_LC2PROseq_noTSS.tfit_merged.png]
