# Supplementary figures and images for: The MUC5B-associated variant rs35705950 resides within an enhancer subject to lineage- and disease-dependent epigenetic remodeling (part 3 of 3)
Source: JCI Insight. 2021 Jan 25;6(2):e144294. doi: 10.1172/jci.insight.144294 (PMC7934873; doi:10.1172/jci.insight.144294)

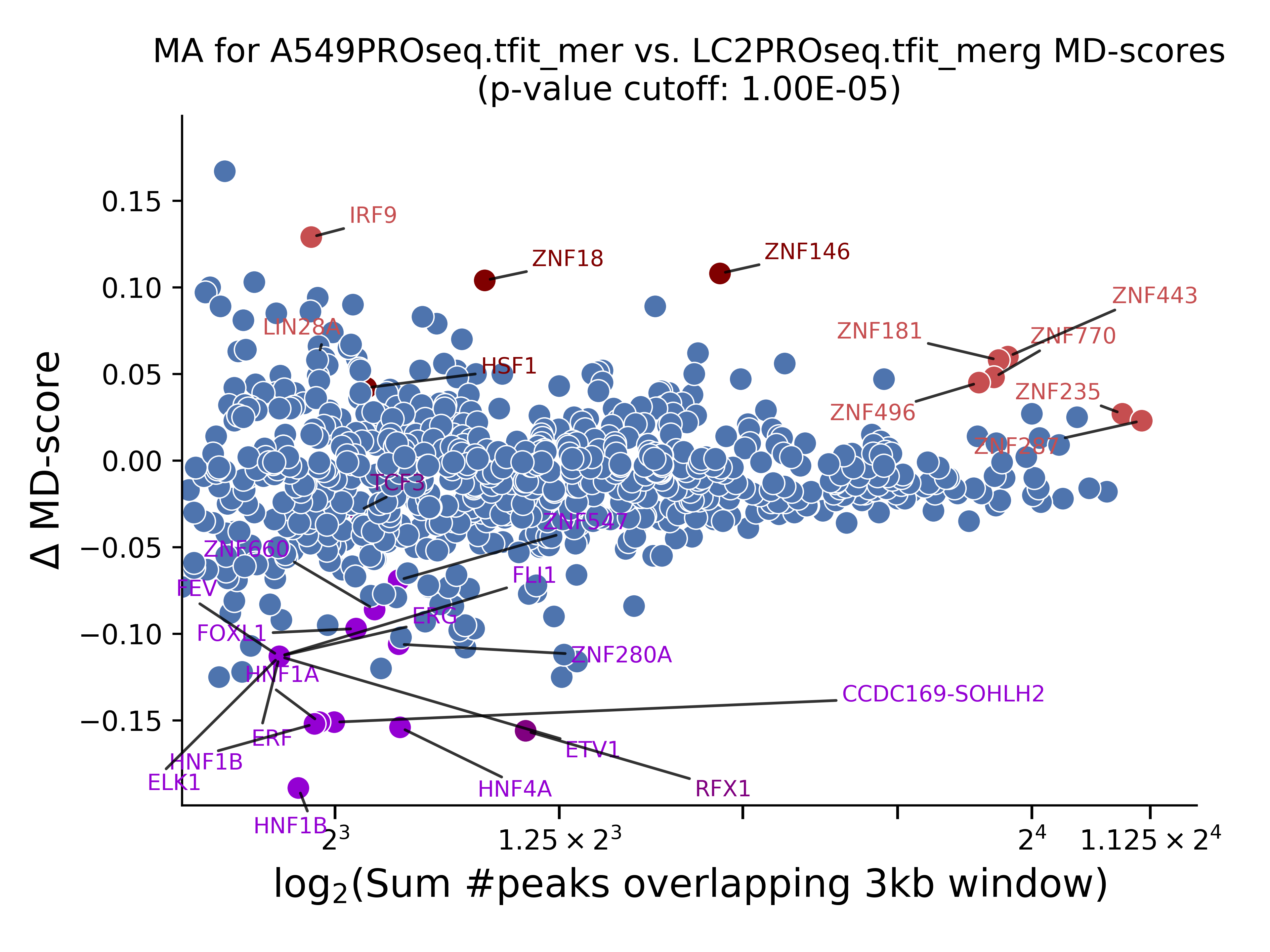

Supplement: Supplemental Data Set 2 [file jciinsight-6-144294-s077.zip › best_curated_Human_TFs_p1e-6_grch38/A549_vs_LC2/MA_A549PROseq.tfit_merged_to_LC2PROseq.tfit_merged_md_score.png]

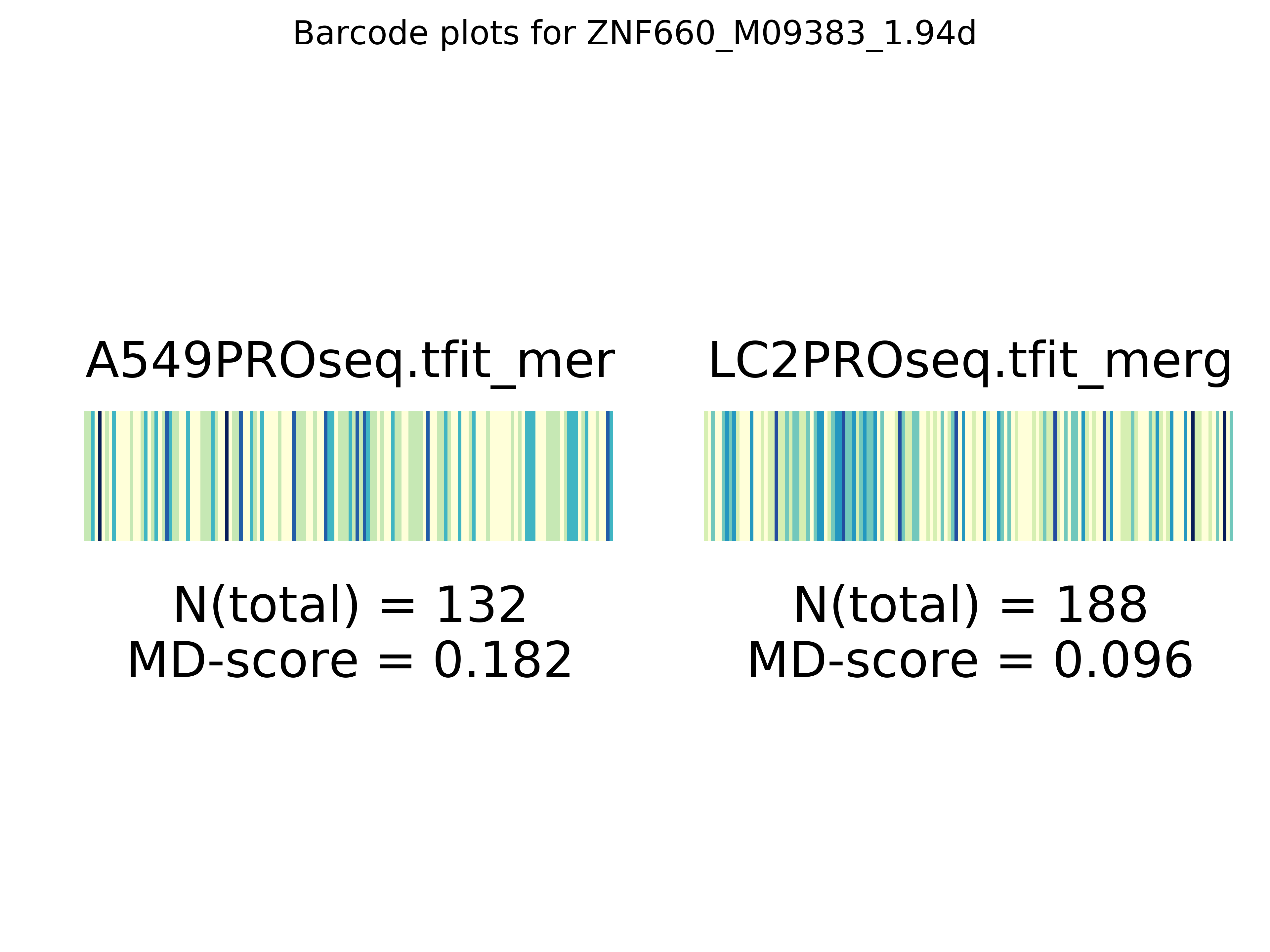

Supplement: Supplemental Data Set 2 [file jciinsight-6-144294-s077.zip › best_curated_Human_TFs_p1e-6_grch38/A549_vs_LC2/ZNF660_M09383_1.94d_barcode_A549PROseq.tfit_merged_vs_LC2PROseq.tfit_merged.png]

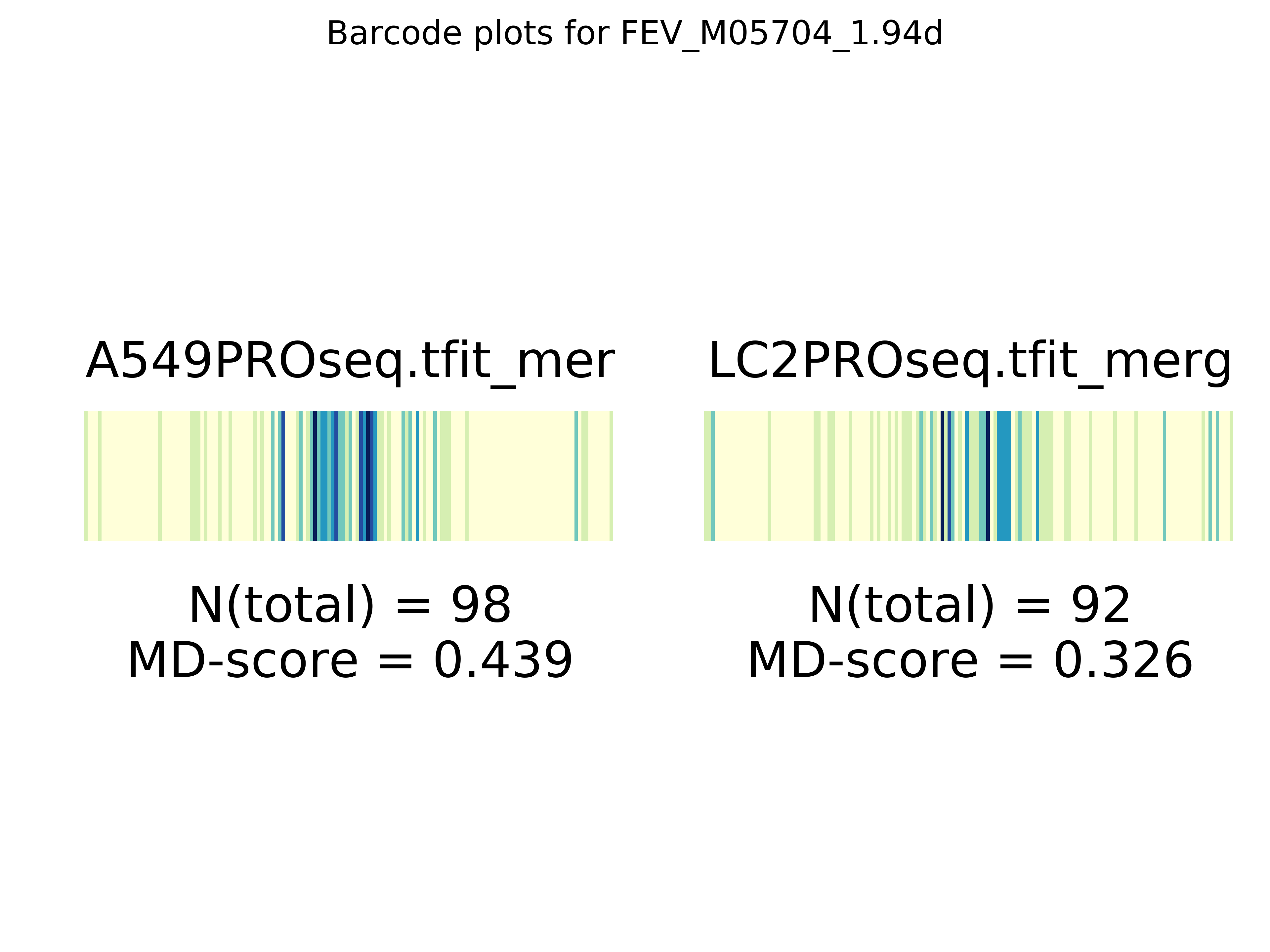

Supplement: Supplemental Data Set 2 [file jciinsight-6-144294-s077.zip › best_curated_Human_TFs_p1e-6_grch38/A549_vs_LC2/FEV_M05704_1.94d_barcode_A549PROseq.tfit_merged_vs_LC2PROseq.tfit_merged.png]

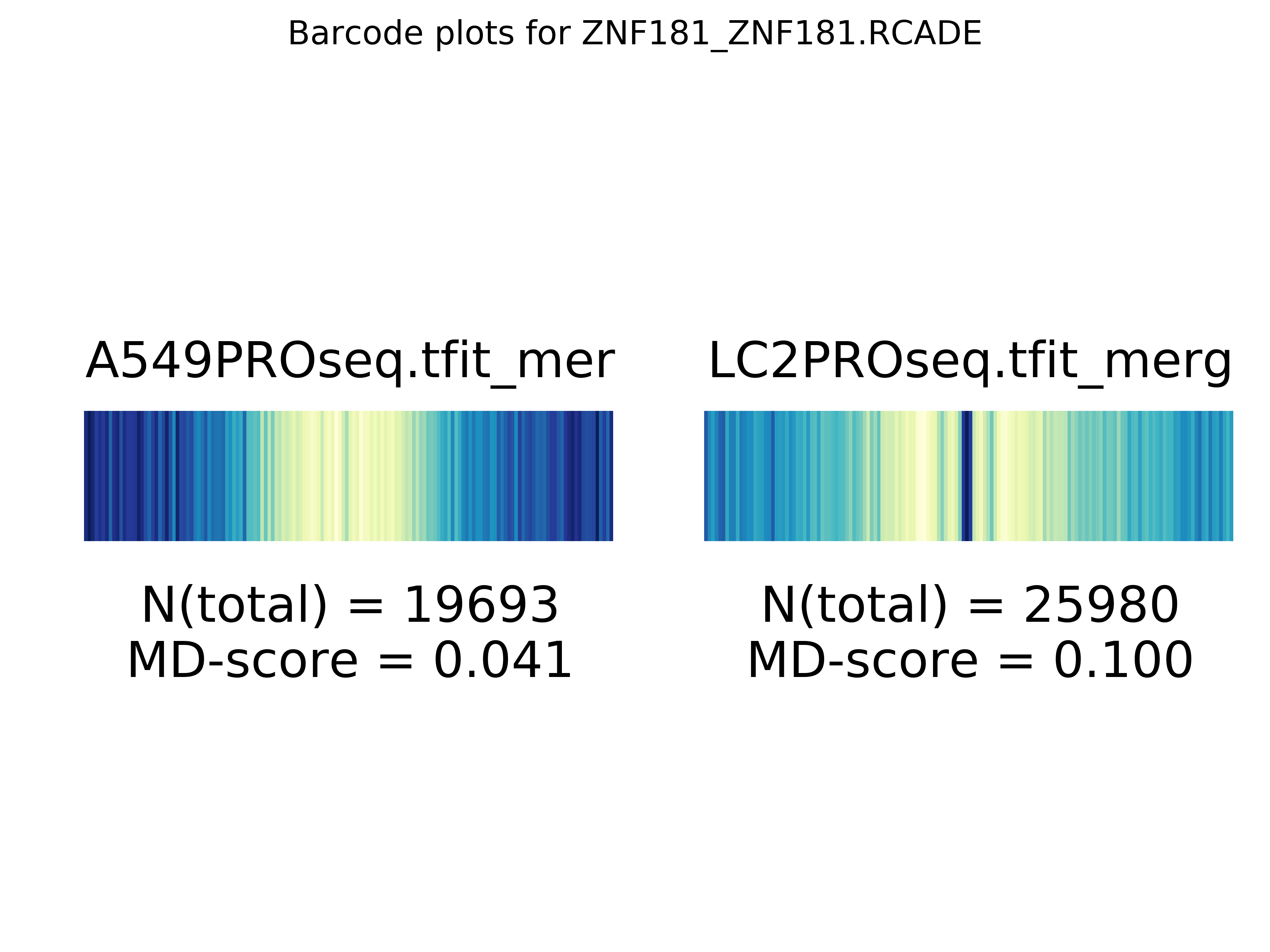

Supplement: Supplemental Data Set 2 [file jciinsight-6-144294-s077.zip › best_curated_Human_TFs_p1e-6_grch38/A549_vs_LC2/ZNF181_ZNF181.RCADE_barcode_A549PROseq.tfit_merged_vs_LC2PROseq.tfit_merged.png]

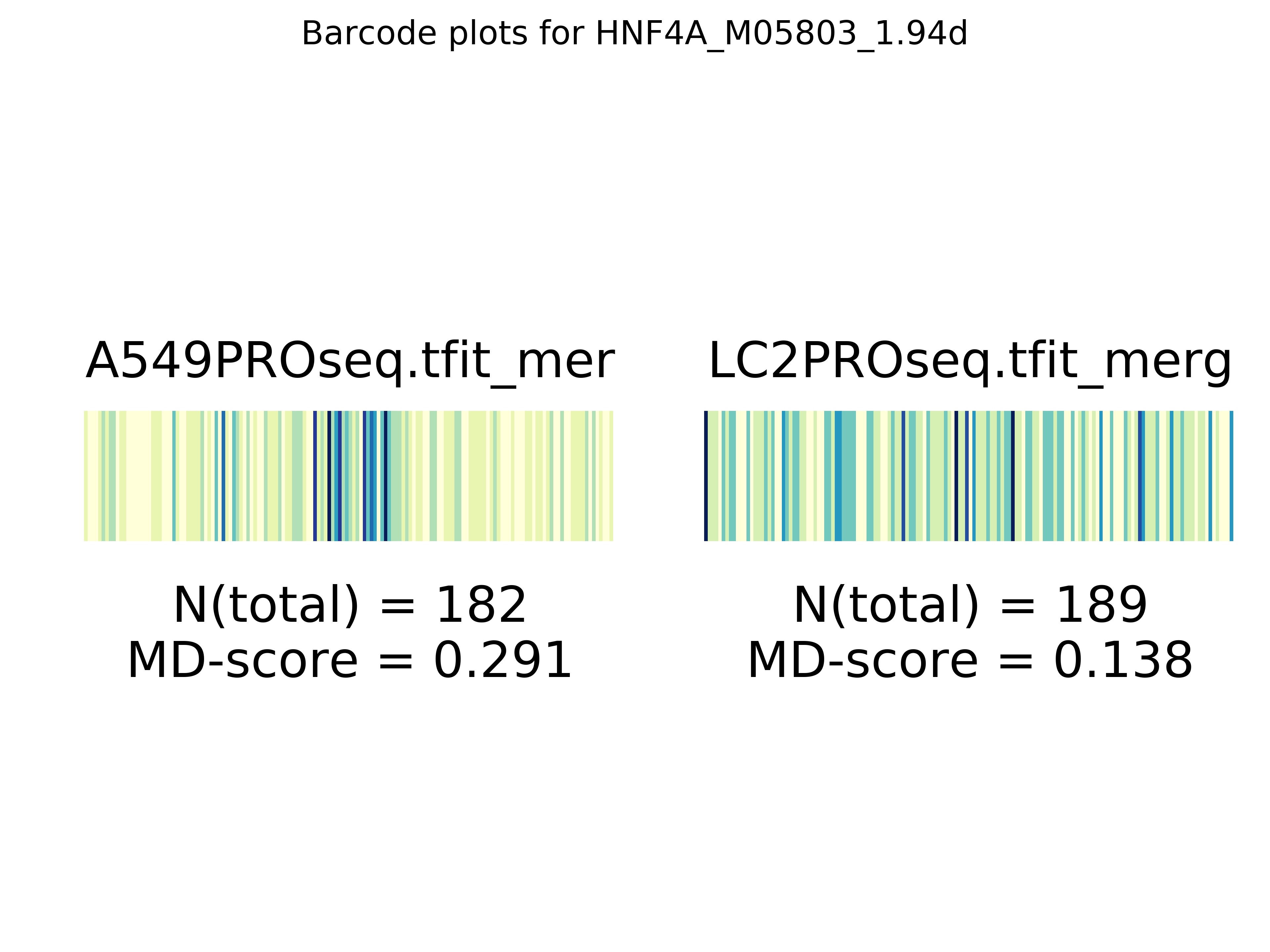

Supplement: Supplemental Data Set 2 [file jciinsight-6-144294-s077.zip › best_curated_Human_TFs_p1e-6_grch38/A549_vs_LC2/HNF4A_M05803_1.94d_barcode_A549PROseq.tfit_merged_vs_LC2PROseq.tfit_merged.png]

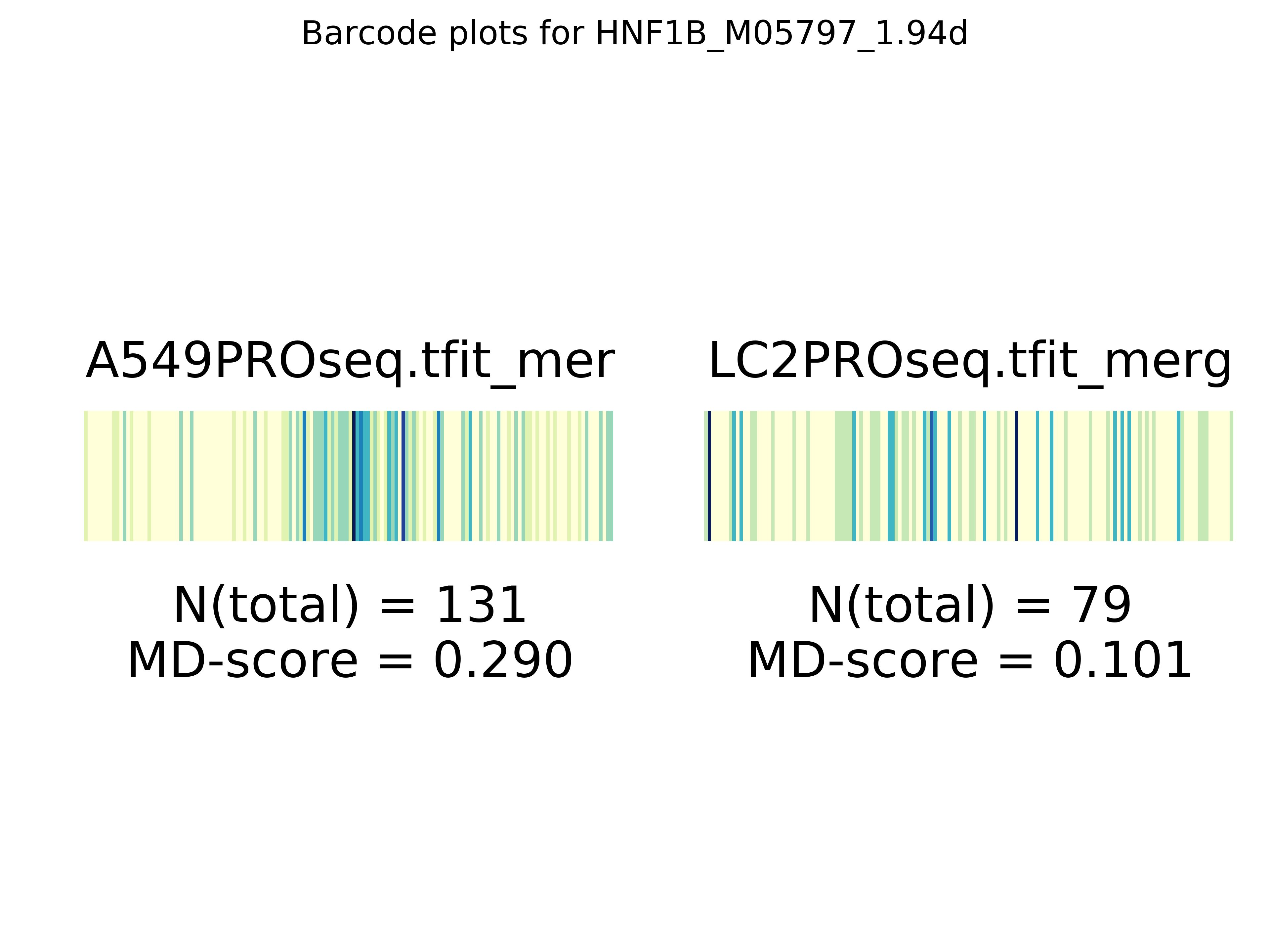

Supplement: Supplemental Data Set 2 [file jciinsight-6-144294-s077.zip › best_curated_Human_TFs_p1e-6_grch38/A549_vs_LC2/HNF1B_M05797_1.94d_barcode_A549PROseq.tfit_merged_vs_LC2PROseq.tfit_merged.png]

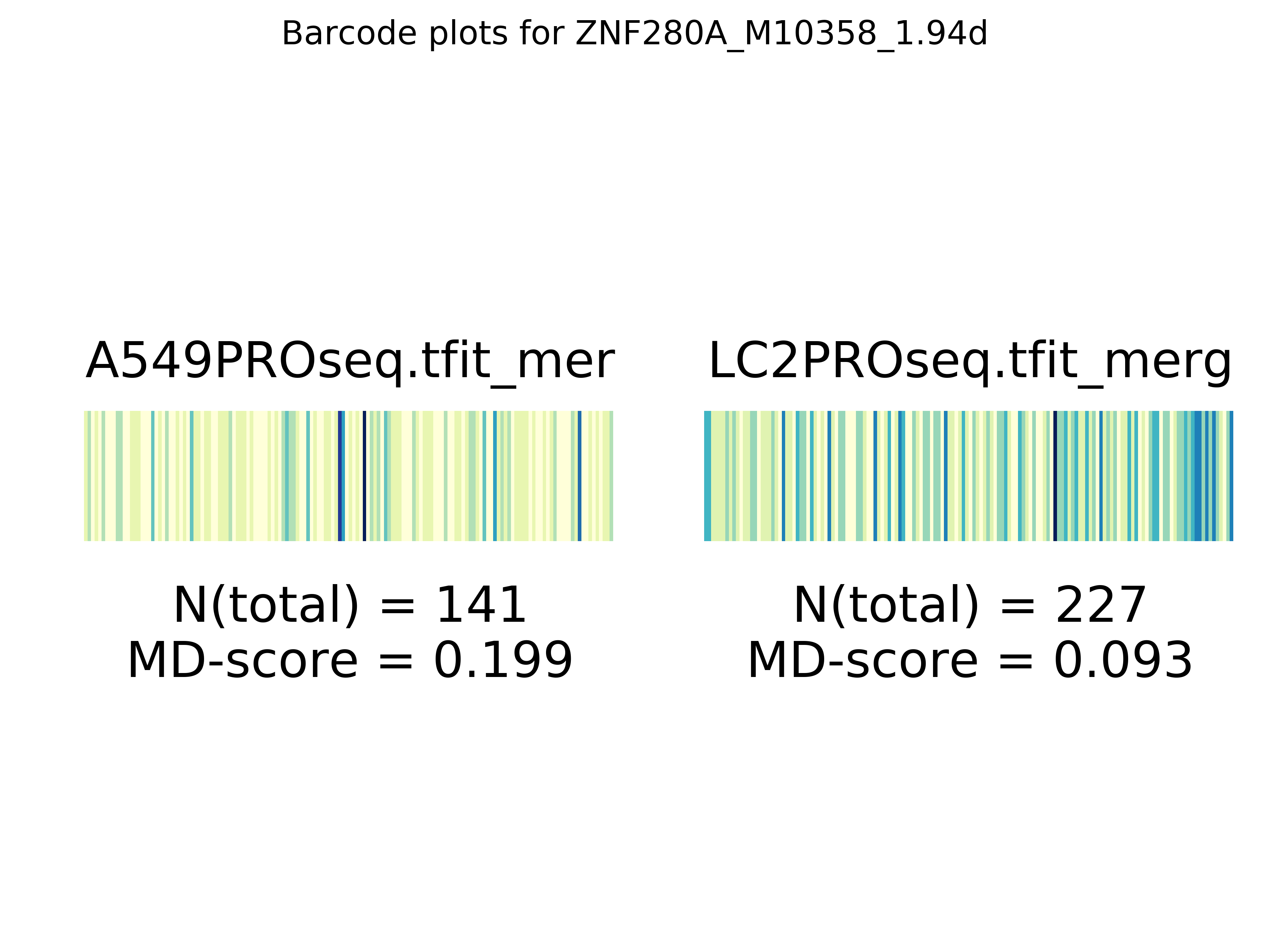

Supplement: Supplemental Data Set 2 [file jciinsight-6-144294-s077.zip › best_curated_Human_TFs_p1e-6_grch38/A549_vs_LC2/ZNF280A_M10358_1.94d_barcode_A549PROseq.tfit_merged_vs_LC2PROseq.tfit_merged.png]

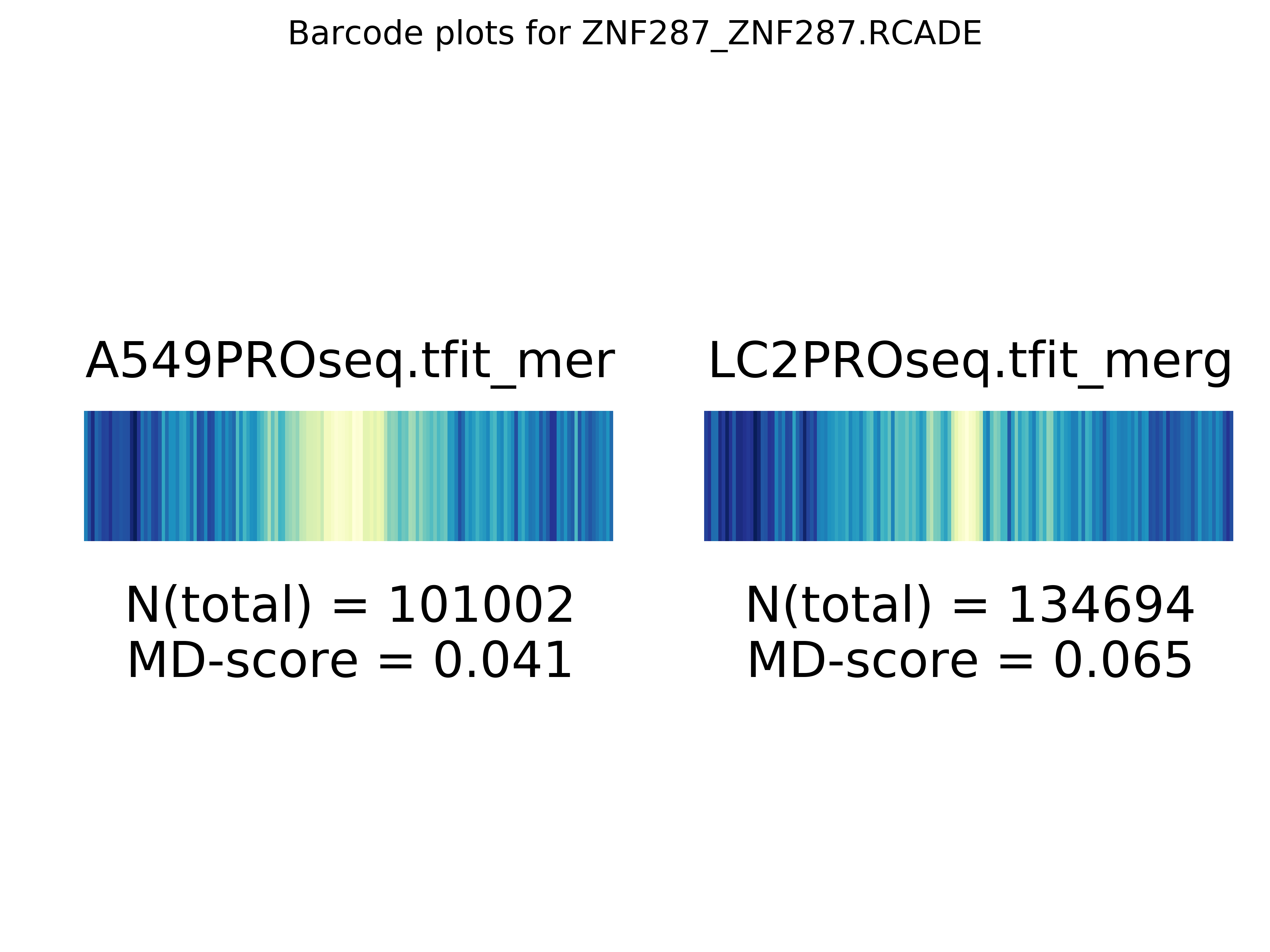

Supplement: Supplemental Data Set 2 [file jciinsight-6-144294-s077.zip › best_curated_Human_TFs_p1e-6_grch38/A549_vs_LC2/ZNF287_ZNF287.RCADE_barcode_A549PROseq.tfit_merged_vs_LC2PROseq.tfit_merged.png]

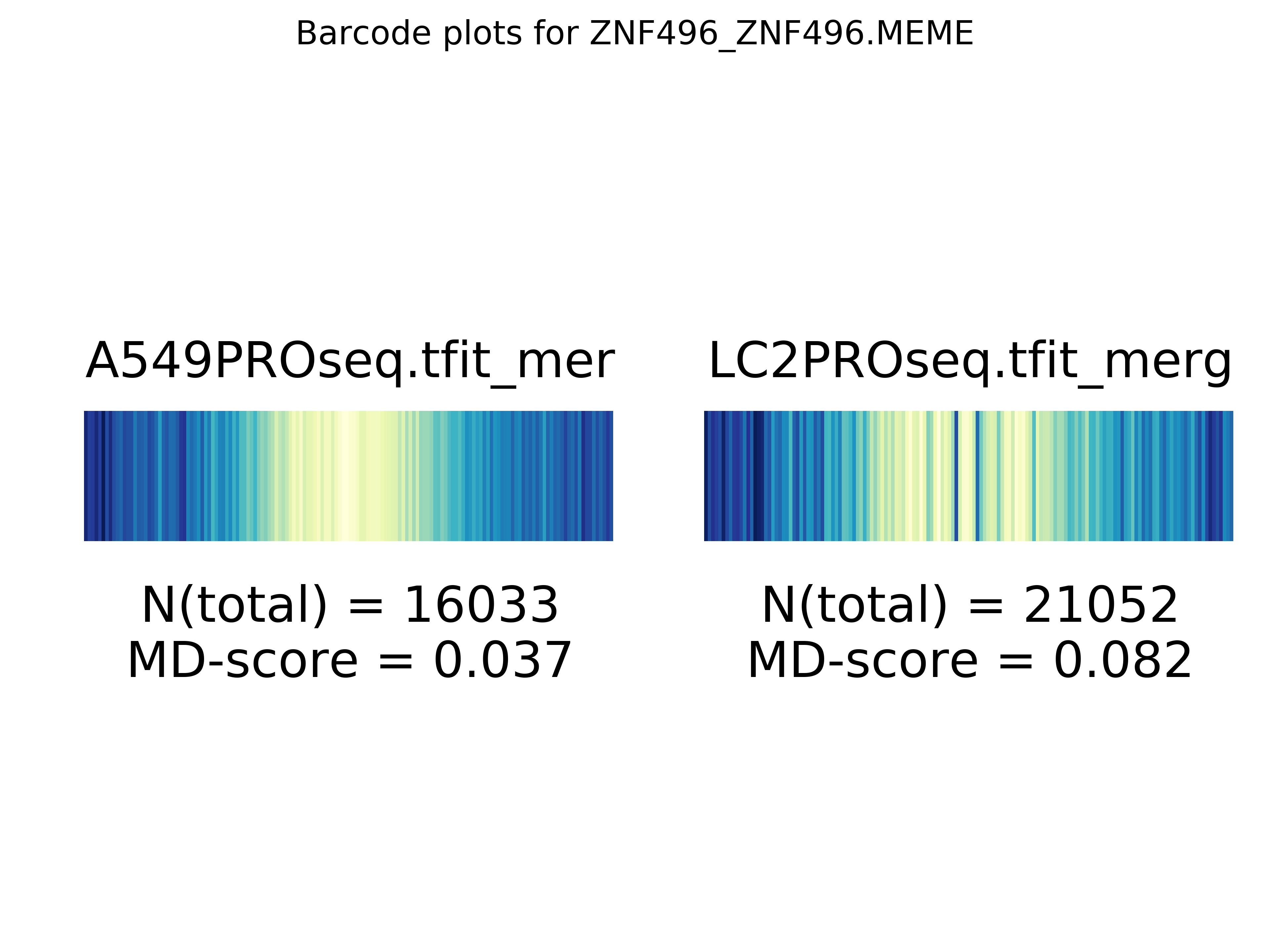

Supplement: Supplemental Data Set 2 [file jciinsight-6-144294-s077.zip › best_curated_Human_TFs_p1e-6_grch38/A549_vs_LC2/ZNF496_ZNF496.MEME_barcode_A549PROseq.tfit_merged_vs_LC2PROseq.tfit_merged.png]

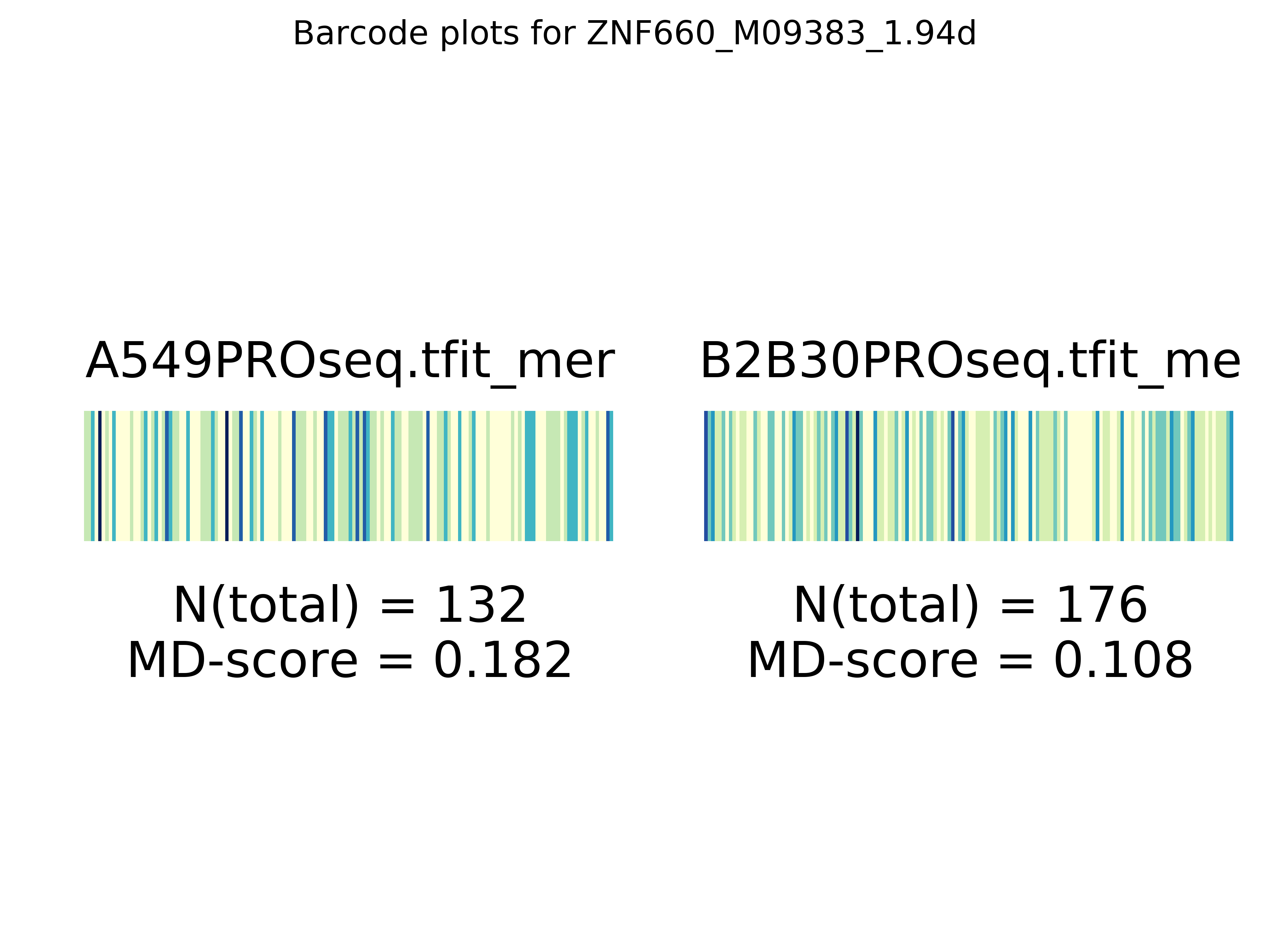

Supplement: Supplemental Data Set 2 [file jciinsight-6-144294-s077.zip › best_curated_Human_TFs_p1e-6_grch38/A549_vs_B2B/ZNF660_M09383_1.94d_barcode_A549PROseq.tfit_merged_vs_B2B30PROseq.tfit_merged.png]

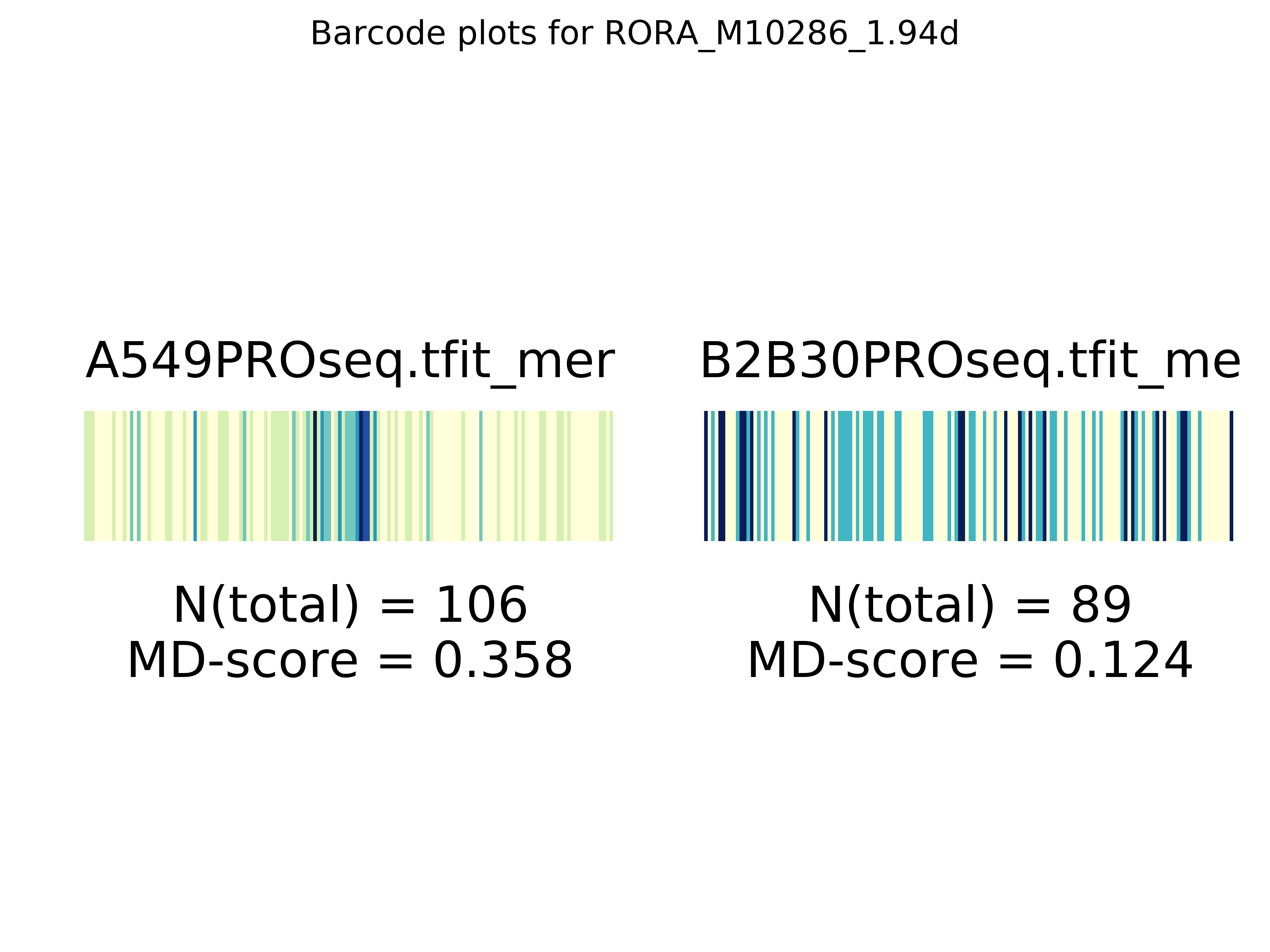

Supplement: Supplemental Data Set 2 [file jciinsight-6-144294-s077.zip › best_curated_Human_TFs_p1e-6_grch38/A549_vs_B2B/RORA_M10286_1.94d_barcode_A549PROseq.tfit_merged_vs_B2B30PROseq.tfit_merged.png]

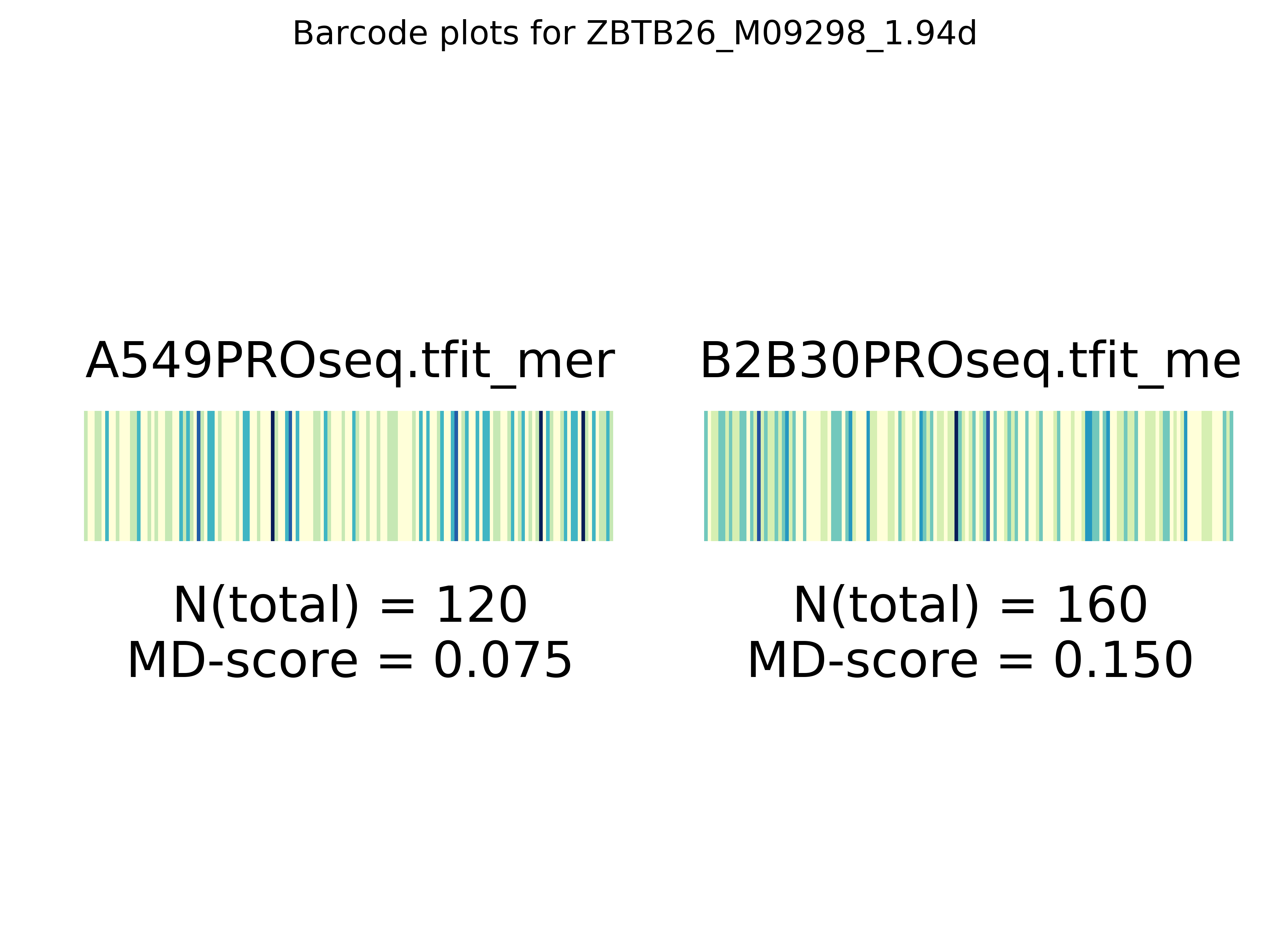

Supplement: Supplemental Data Set 2 [file jciinsight-6-144294-s077.zip › best_curated_Human_TFs_p1e-6_grch38/A549_vs_B2B/ZBTB26_M09298_1.94d_barcode_A549PROseq.tfit_merged_vs_B2B30PROseq.tfit_merged.png]

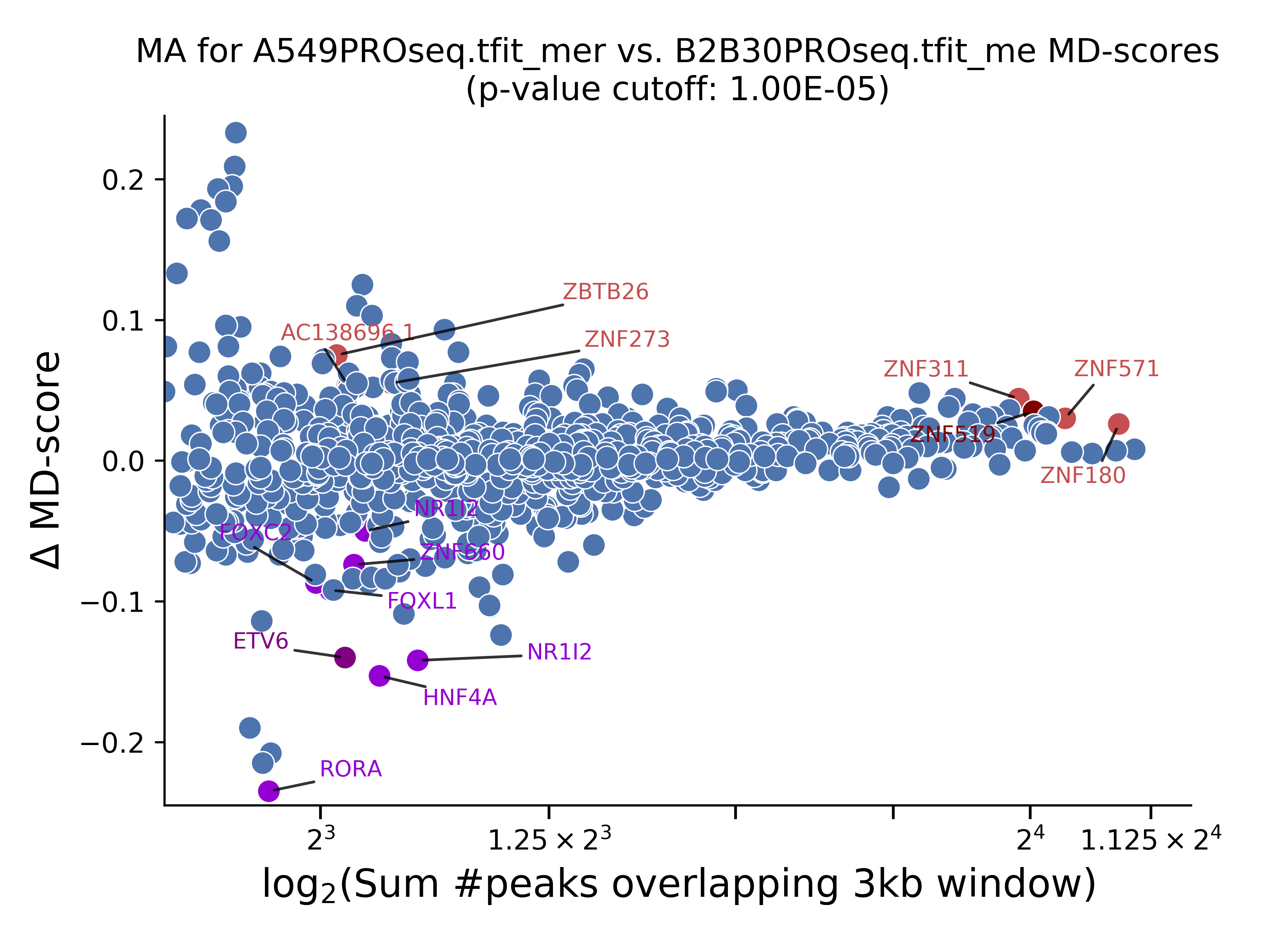

Supplement: Supplemental Data Set 2 [file jciinsight-6-144294-s077.zip › best_curated_Human_TFs_p1e-6_grch38/A549_vs_B2B/MA_A549PROseq.tfit_merged_to_B2B30PROseq.tfit_merged_md_score.png]

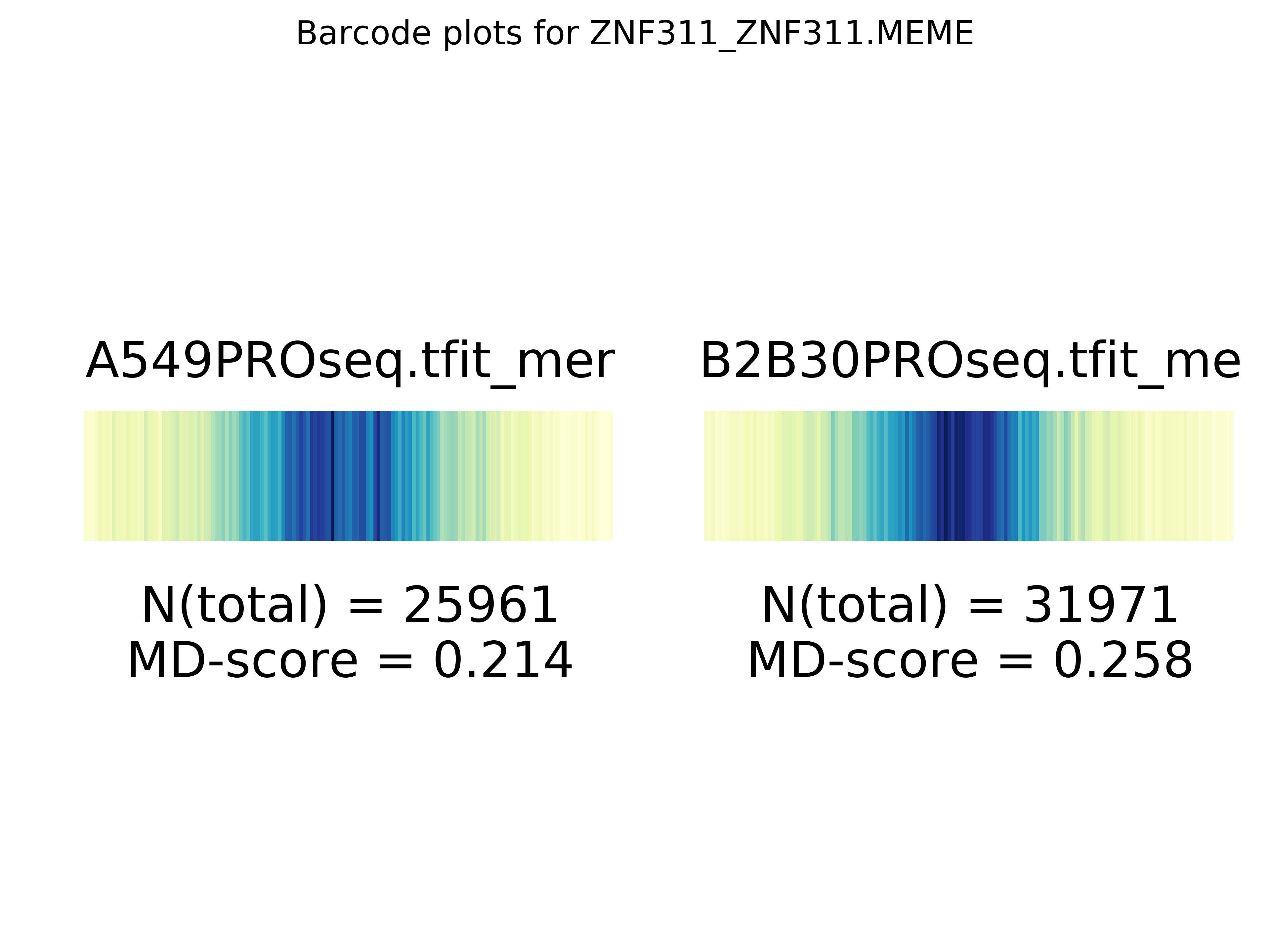

Supplement: Supplemental Data Set 2 [file jciinsight-6-144294-s077.zip › best_curated_Human_TFs_p1e-6_grch38/A549_vs_B2B/ZNF311_ZNF311.MEME_barcode_A549PROseq.tfit_merged_vs_B2B30PROseq.tfit_merged.png]

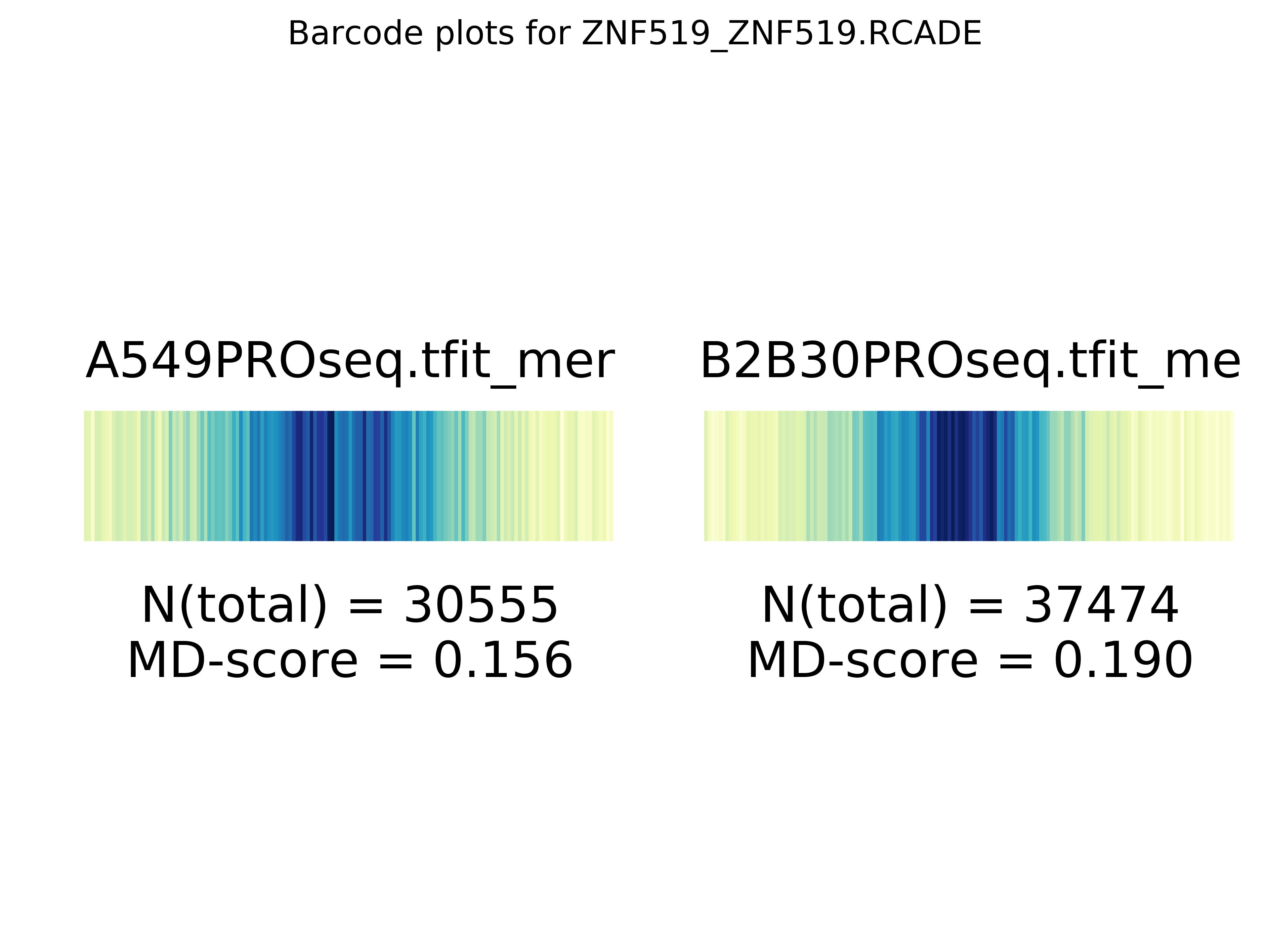

Supplement: Supplemental Data Set 2 [file jciinsight-6-144294-s077.zip › best_curated_Human_TFs_p1e-6_grch38/A549_vs_B2B/ZNF519_ZNF519.RCADE_barcode_A549PROseq.tfit_merged_vs_B2B30PROseq.tfit_merged.png]

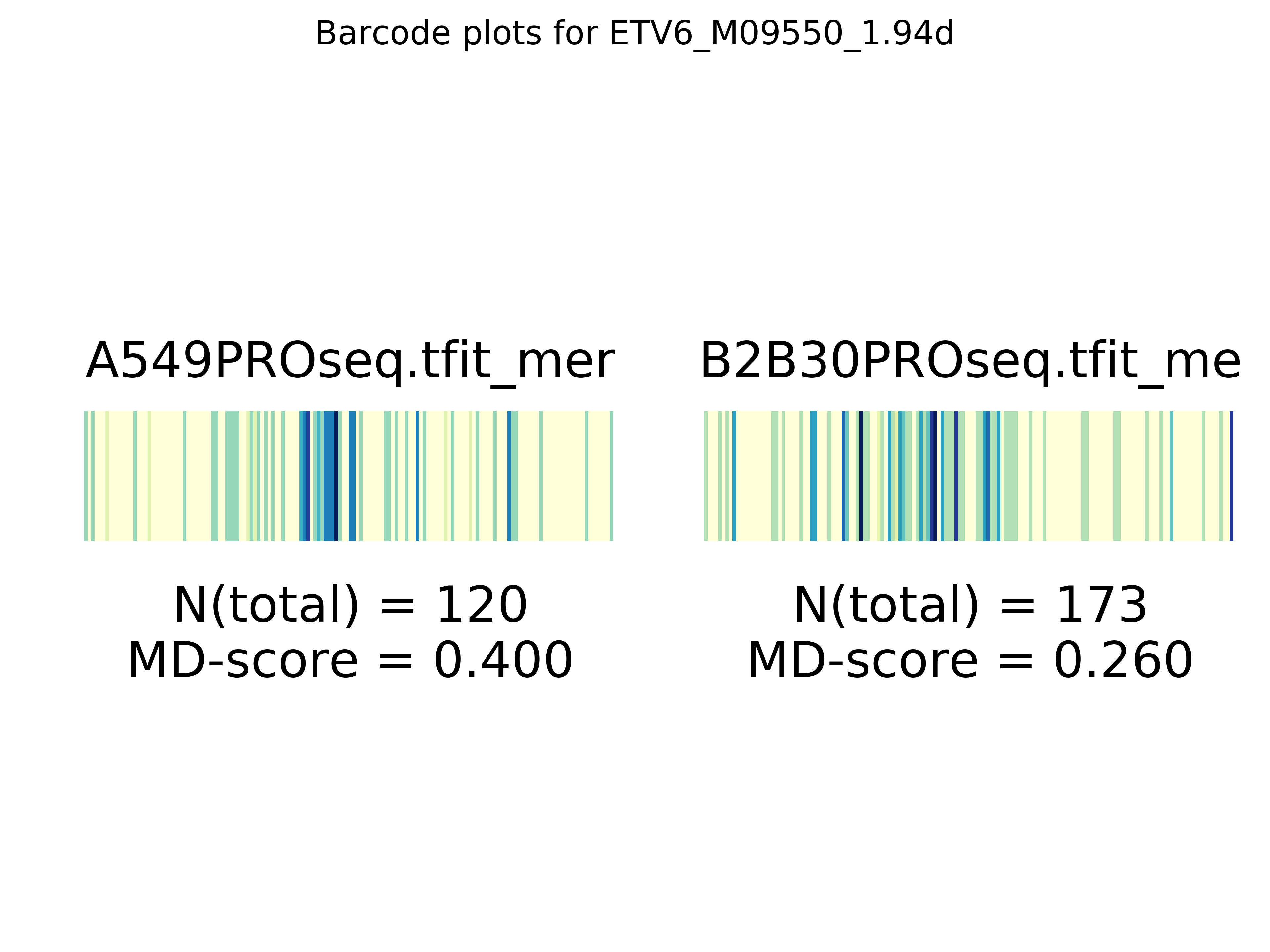

Supplement: Supplemental Data Set 2 [file jciinsight-6-144294-s077.zip › best_curated_Human_TFs_p1e-6_grch38/A549_vs_B2B/ETV6_M09550_1.94d_barcode_A549PROseq.tfit_merged_vs_B2B30PROseq.tfit_merged.png]

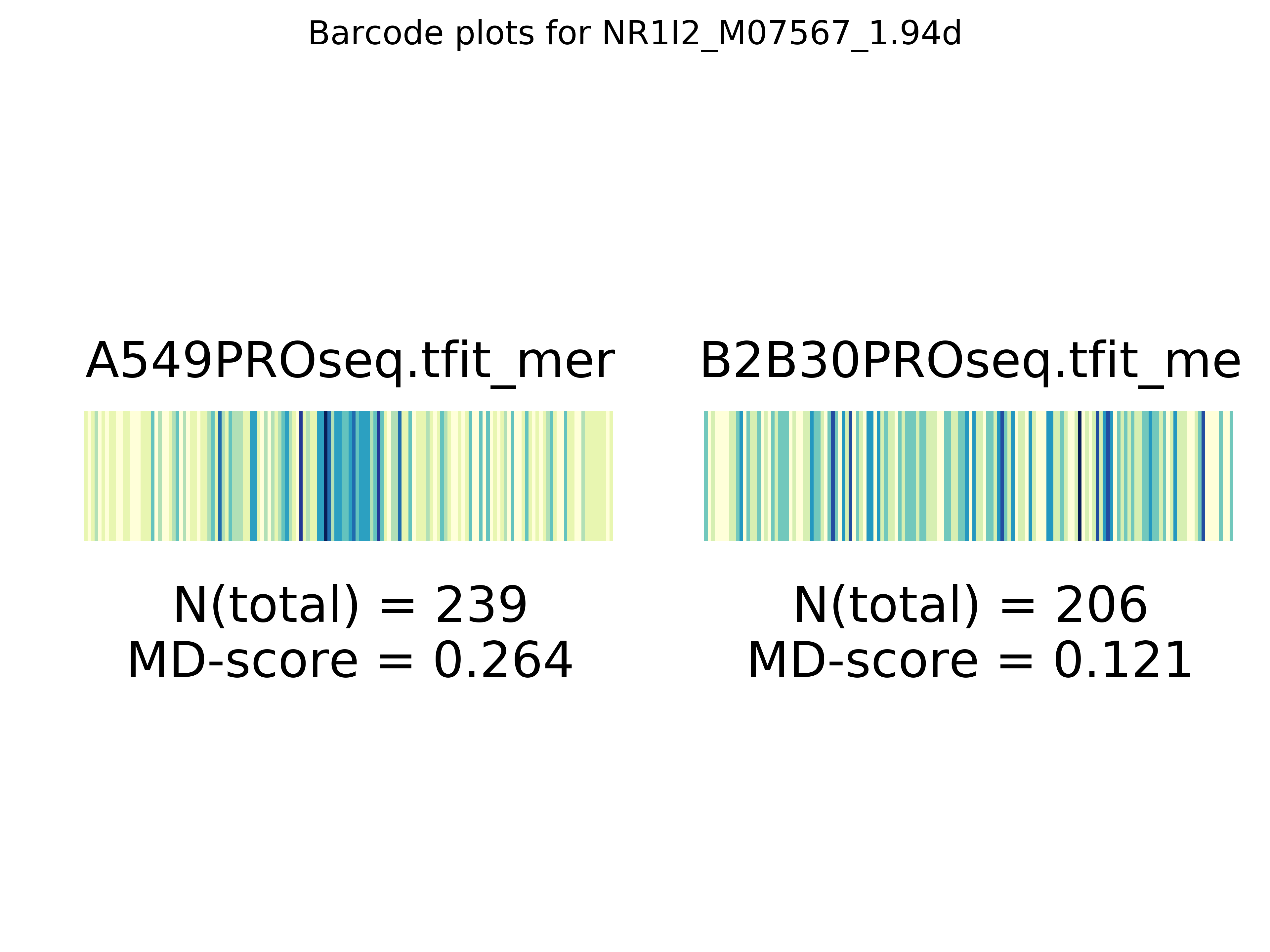

Supplement: Supplemental Data Set 2 [file jciinsight-6-144294-s077.zip › best_curated_Human_TFs_p1e-6_grch38/A549_vs_B2B/NR1I2_M07567_1.94d_barcode_A549PROseq.tfit_merged_vs_B2B30PROseq.tfit_merged.png]

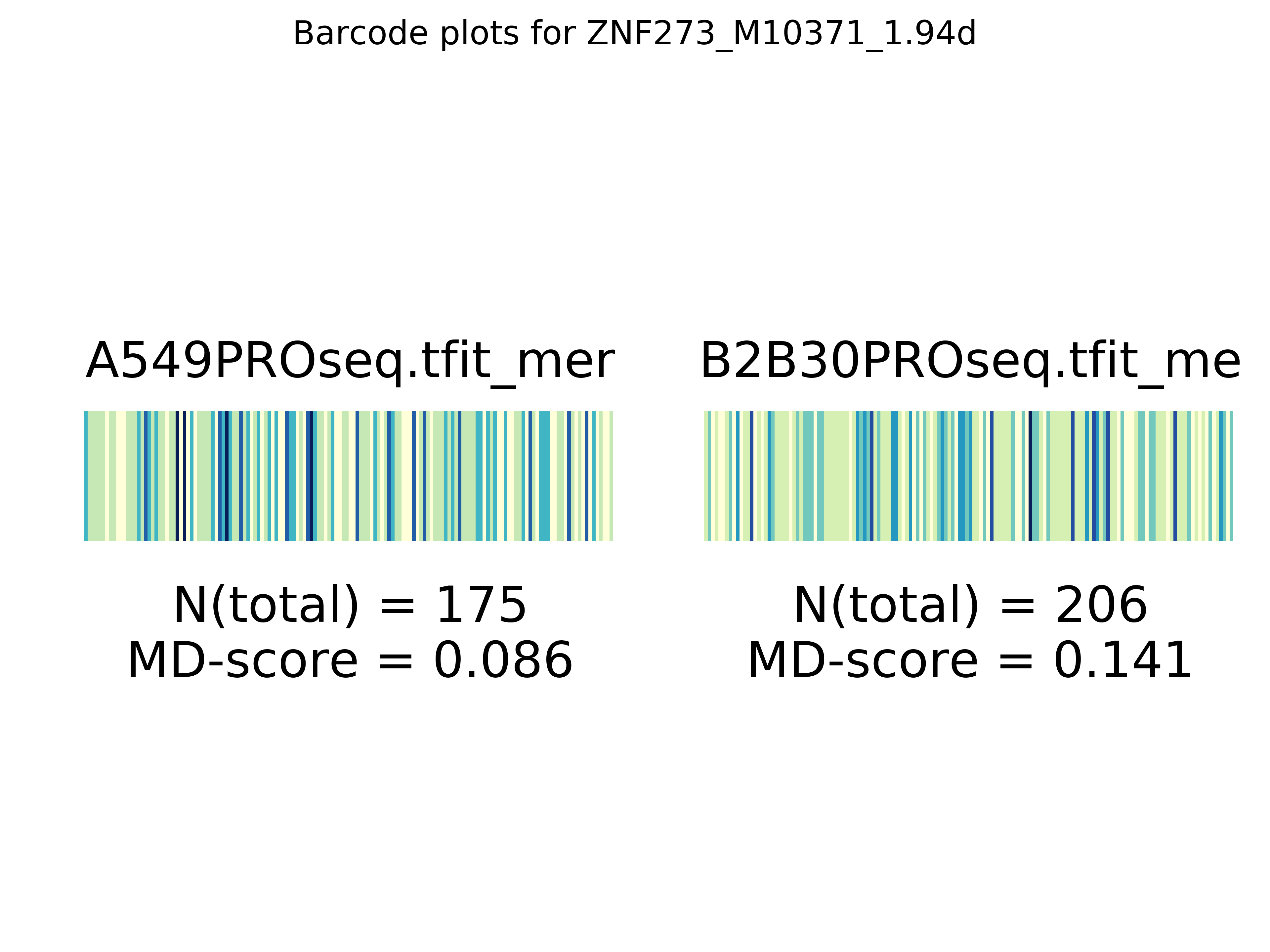

Supplement: Supplemental Data Set 2 [file jciinsight-6-144294-s077.zip › best_curated_Human_TFs_p1e-6_grch38/A549_vs_B2B/ZNF273_M10371_1.94d_barcode_A549PROseq.tfit_merged_vs_B2B30PROseq.tfit_merged.png]

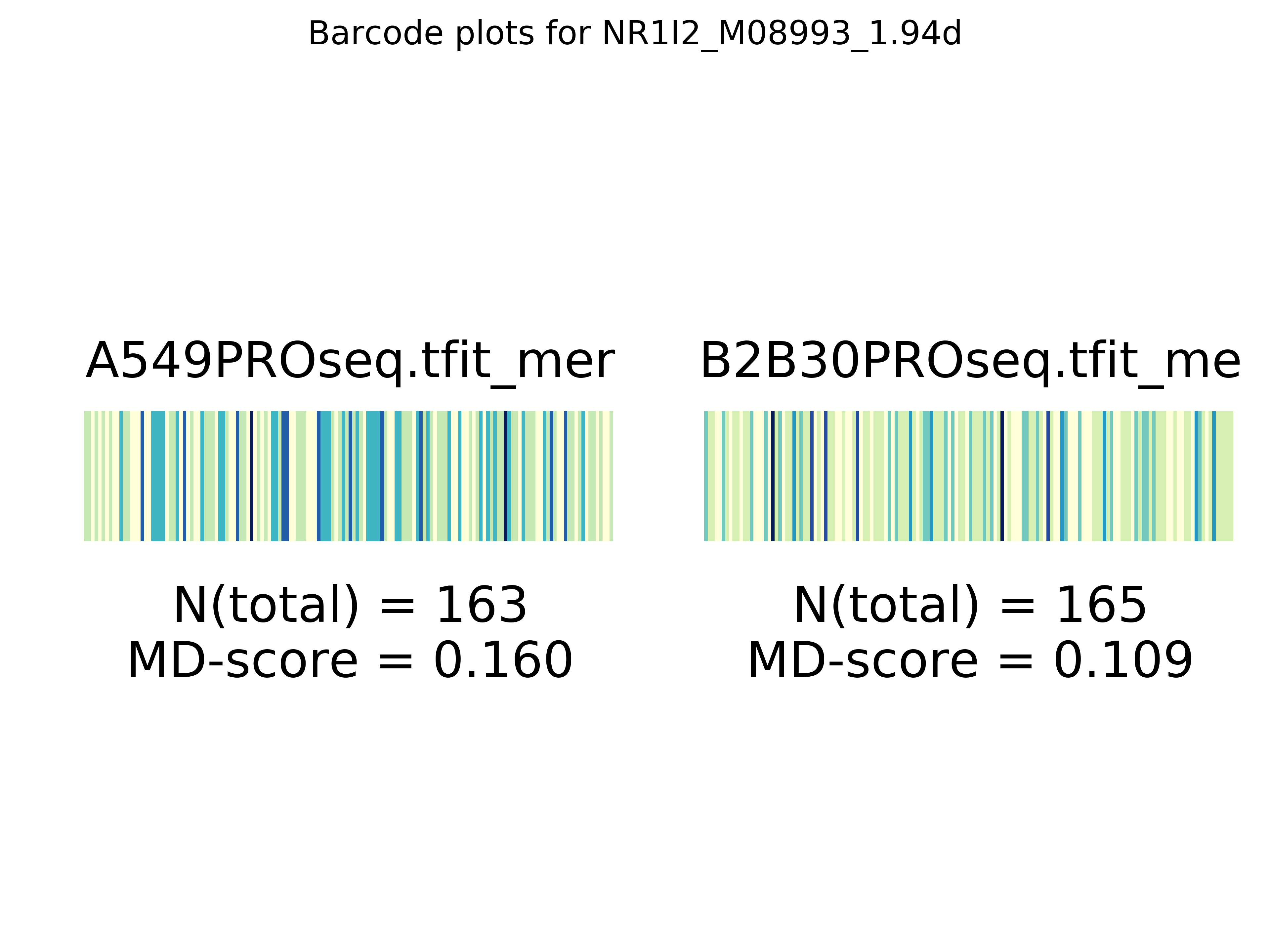

Supplement: Supplemental Data Set 2 [file jciinsight-6-144294-s077.zip › best_curated_Human_TFs_p1e-6_grch38/A549_vs_B2B/NR1I2_M08993_1.94d_barcode_A549PROseq.tfit_merged_vs_B2B30PROseq.tfit_merged.png]

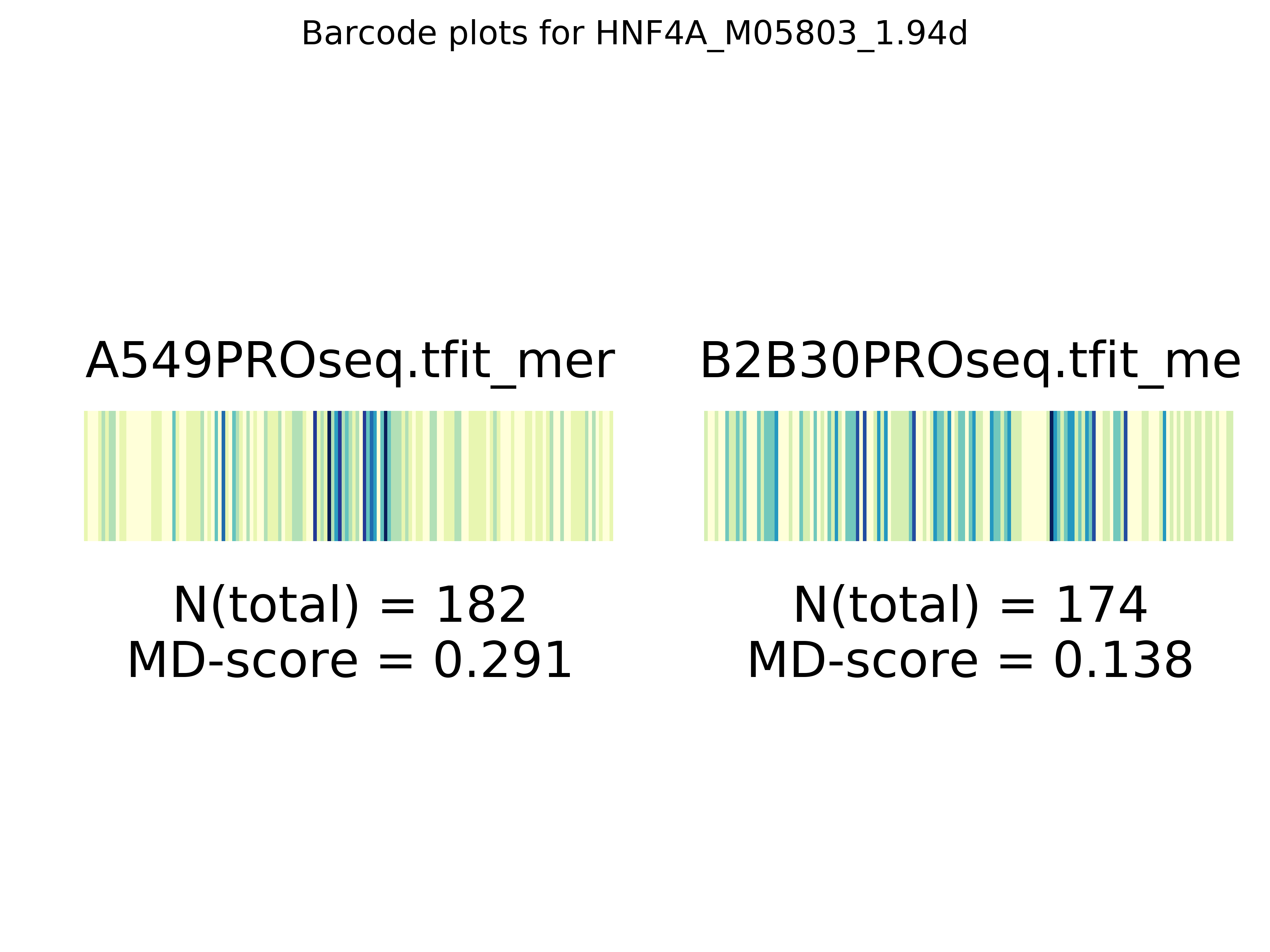

Supplement: Supplemental Data Set 2 [file jciinsight-6-144294-s077.zip › best_curated_Human_TFs_p1e-6_grch38/A549_vs_B2B/HNF4A_M05803_1.94d_barcode_A549PROseq.tfit_merged_vs_B2B30PROseq.tfit_merged.png]

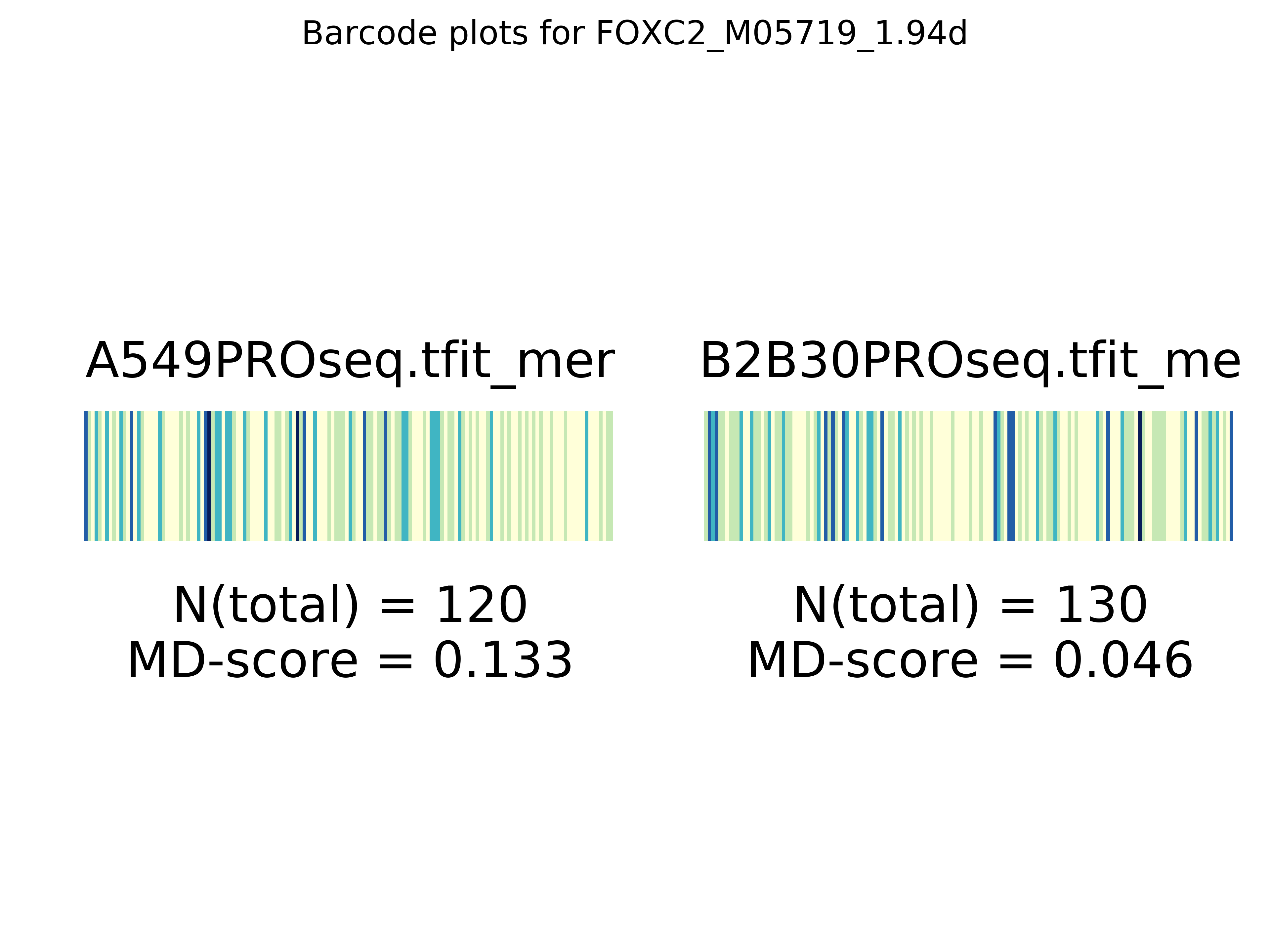

Supplement: Supplemental Data Set 2 [file jciinsight-6-144294-s077.zip › best_curated_Human_TFs_p1e-6_grch38/A549_vs_B2B/FOXC2_M05719_1.94d_barcode_A549PROseq.tfit_merged_vs_B2B30PROseq.tfit_merged.png]

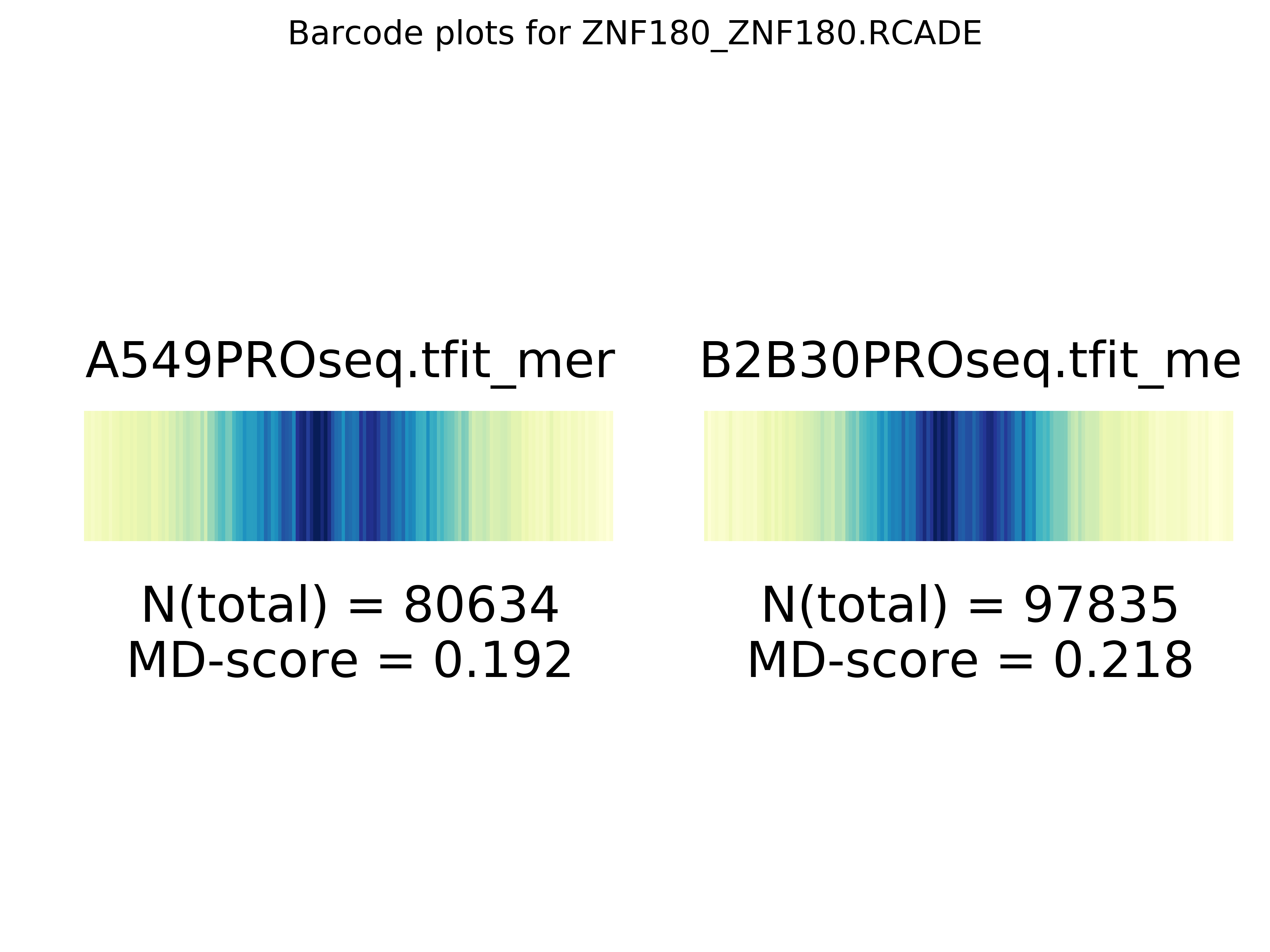

Supplement: Supplemental Data Set 2 [file jciinsight-6-144294-s077.zip › best_curated_Human_TFs_p1e-6_grch38/A549_vs_B2B/ZNF180_ZNF180.RCADE_barcode_A549PROseq.tfit_merged_vs_B2B30PROseq.tfit_merged.png]

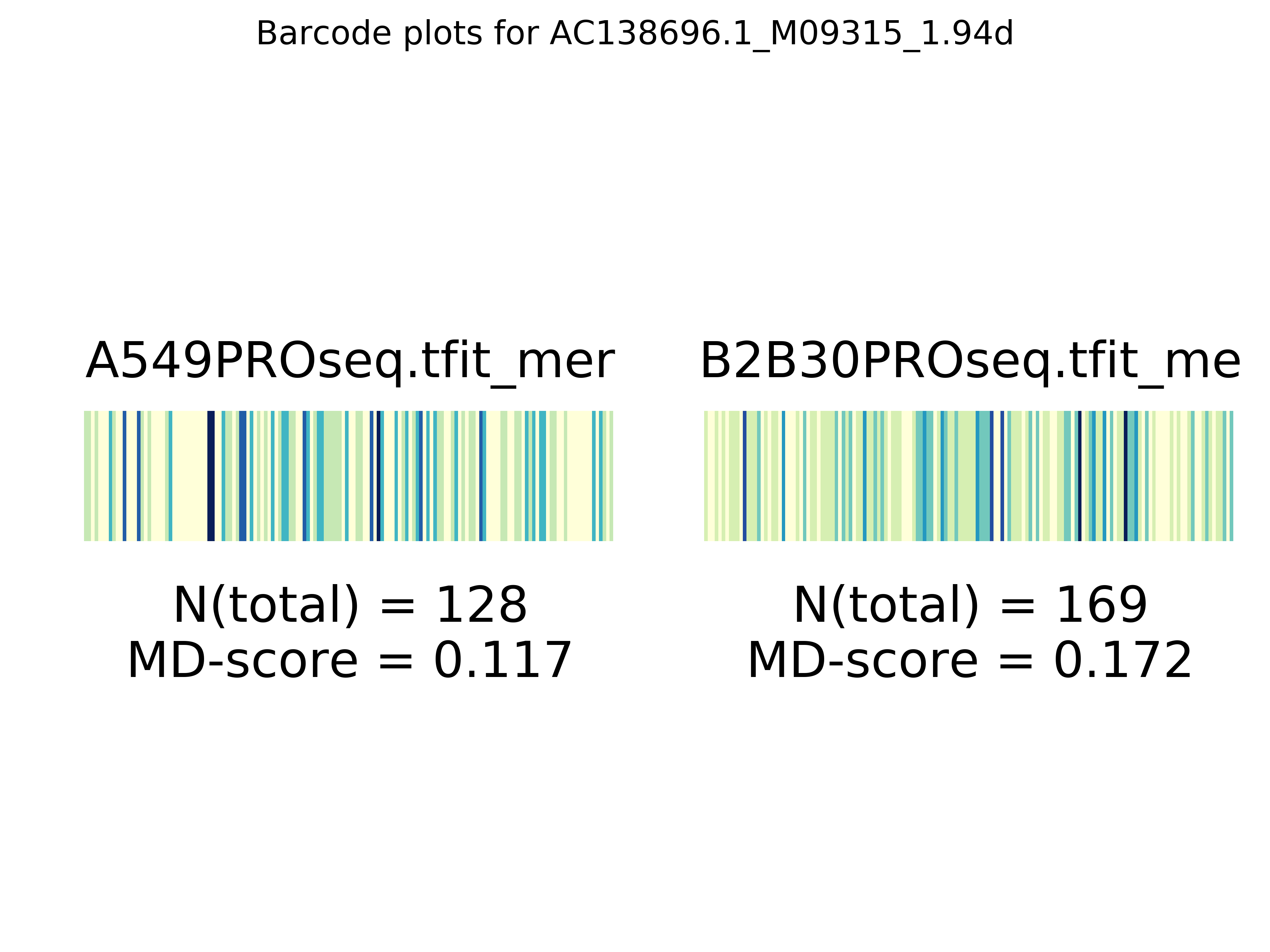

Supplement: Supplemental Data Set 2 [file jciinsight-6-144294-s077.zip › best_curated_Human_TFs_p1e-6_grch38/A549_vs_B2B/AC138696.1_M09315_1.94d_barcode_A549PROseq.tfit_merged_vs_B2B30PROseq.tfit_merged.png]

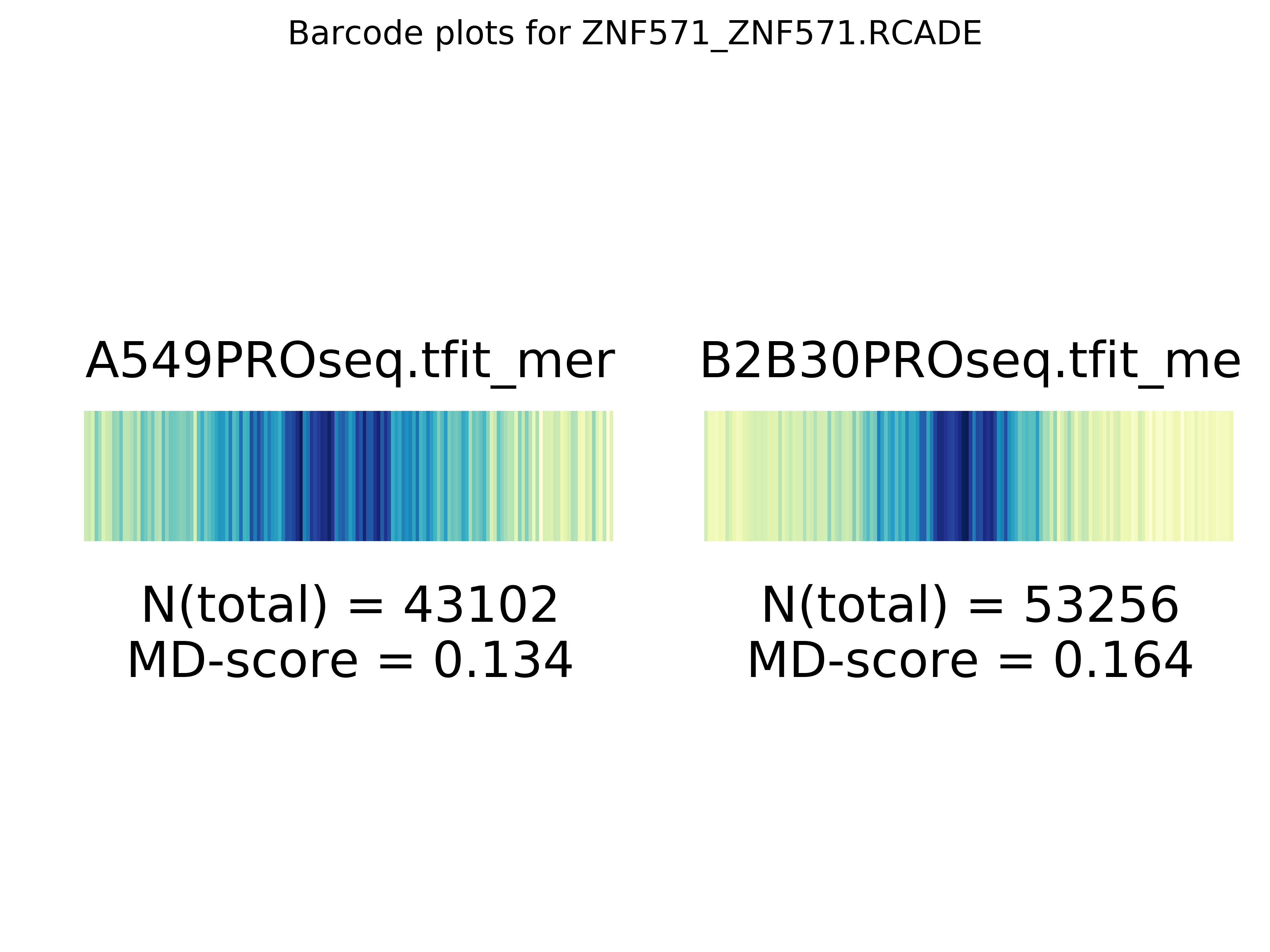

Supplement: Supplemental Data Set 2 [file jciinsight-6-144294-s077.zip › best_curated_Human_TFs_p1e-6_grch38/A549_vs_B2B/ZNF571_ZNF571.RCADE_barcode_A549PROseq.tfit_merged_vs_B2B30PROseq.tfit_merged.png]

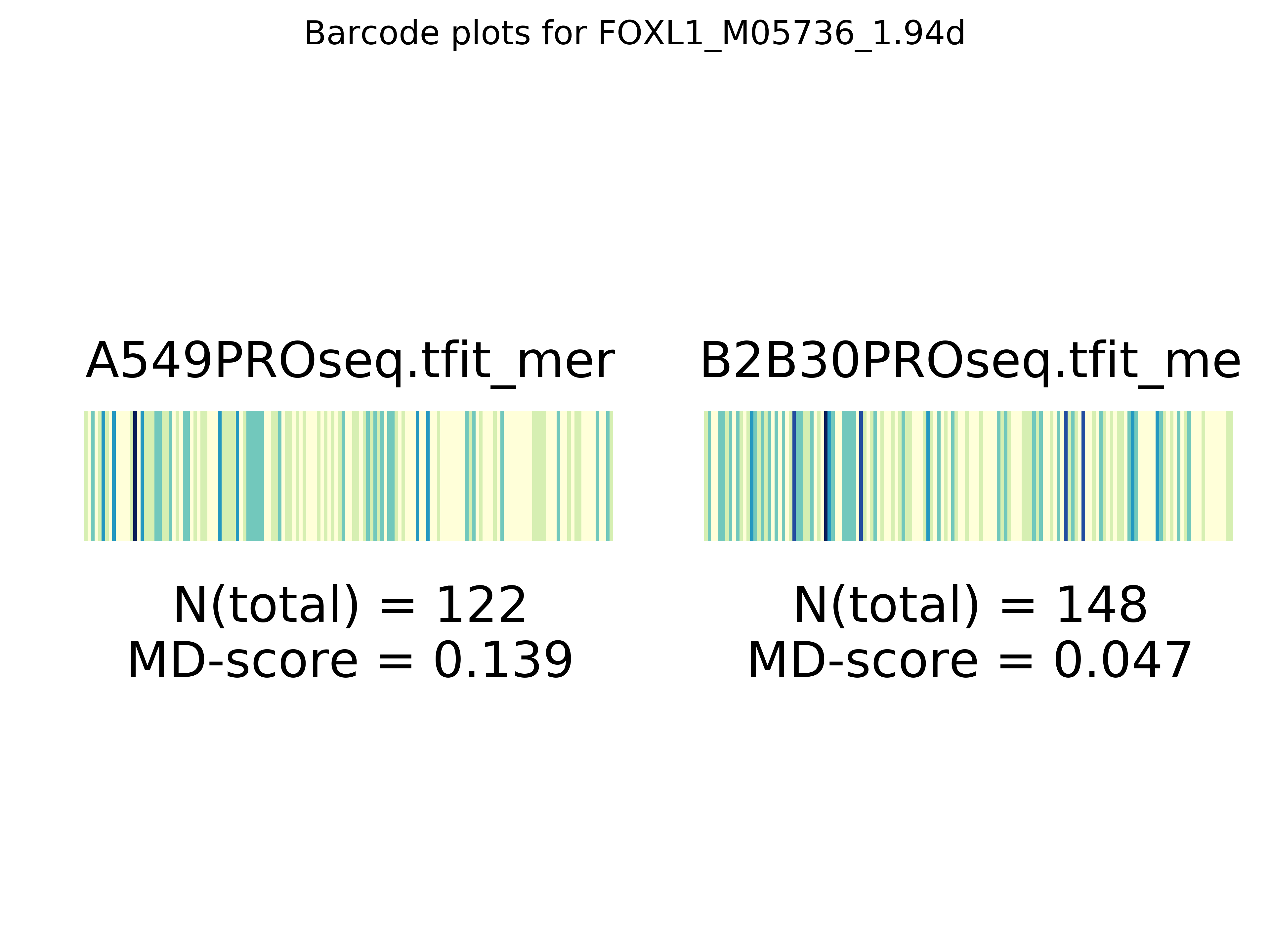

Supplement: Supplemental Data Set 2 [file jciinsight-6-144294-s077.zip › best_curated_Human_TFs_p1e-6_grch38/A549_vs_B2B/FOXL1_M05736_1.94d_barcode_A549PROseq.tfit_merged_vs_B2B30PROseq.tfit_merged.png]
