# Supplementary material for: The MUC5B-associated variant rs35705950 resides within an enhancer subject to lineage- and disease-dependent epigenetic remodeling
Source: JCI Insight. 2021 Jan 25;6(2):e144294. doi: 10.1172/jci.insight.144294 (PMC7934873; doi:10.1172/jci.insight.144294)
Supplement: Supplemental Data Set 3 [file jciinsight-6-144294-s078.zip › Supplemental File S3_PRO-seq Pipeline & QC Reports/PRO-seq_Nextflow_pipeline_report/nascent_pipeline_report.html]

[romantic\_lichterman] Nextflow Workflow Report


Nextflow Report


- Summary
- Resources
- Tasks

[romantic\_lichterman]

# Nextflow workflow report

## `[romantic_lichterman]`

Workflow execution completed successfully!

Run times
:   Wed Oct 23 15:17:35 MDT 2019 - Thu Oct 24 14:33:29 MDT 2019
    (duration: **23h 15m 54s**)

98 succeeded

0 cached

0 ignored

0 failed

Nextflow command
:   ```
    nextflow run /Users/magr0763/Nascent-Flow/main.nf -profile hg38 --fastqs '/scratch/Shares/dowell/Sasse/Unstimulated_PROseq/fastq/*.fastq.gz' --workdir /scratch/Shares/dowell/Sasse/Unstimulated_PROseq/temp --email margaret.gruca@colorado.edu --outdir /scratch/Shares/dowell/Sasse/Unstimulated_PROseq --genome_id hg38 --singleEnd --flip --savebg --savebw --dastk --tfit --fstitch --counts
    ```

CPU-Hours
:   `1'529.2`

Launch directory
:   `/scratch/Shares/dowell/Sasse`

Work directory
:   `/scratch/Shares/dowell/Sasse/Unstimulated_PROseq/temp`

Project directory
:   `/Users/magr0763/Nascent-Flow`

Script name
:   `main.nf`

Script ID
:   `6af6ada925af93c15815d311d1a63f88`

Workflow session
:   `fb945a7e-76c1-49bb-a651-dcc213c9b056`

Workflow profile
:   hg38

Workflow container
:   `skptic/nascentflow:latest`

Container engine
:   `-`

Nextflow version
:   version 19.04.1, build 5072 (03-05-2019 12:29 UTC)

## Resource Usage

These plots give an overview of the distribution of resource usage for each process.

#### CPU

- Raw Usage
- % Allocated

#### Memory

- Physical (RAM)
- Virtual (RAM + Disk swap)
- % RAM Allocated

#### Job Duration

- Raw Usage
- % Allocated

#### I/O

- Read
- Write

## Tasks

This table shows information about each task in the workflow. Use the search box on the right
to filter rows for specific values. Clicking headers will sort the table by that value and
scrolling side to side will reveal more columns.

Values shown as:

Human readable
Raw values

(tasks table omitted because the dataset is too big)

Generated by Nextflow, version 19.04.1
