# Supplementary material for: The MUC5B-associated variant rs35705950 resides within an enhancer subject to lineage- and disease-dependent epigenetic remodeling
Source: JCI Insight. 2021 Jan 25;6(2):e144294. doi: 10.1172/jci.insight.144294 (PMC7934873; doi:10.1172/jci.insight.144294)
Supplement: Supplemental Data Set 3 [file jciinsight-6-144294-s078.zip › Supplemental File S3_PRO-seq Pipeline & QC Reports/PRO-seq_Nextflow_pipeline_report/nascent_pipeline_timeline.html]

### Processes execution timeline

Launch time:    
Elapsed time:    
Legend: job wall time / memory usage (RAM)

Created with Nextflow -- http://nextflow.io
