# Supplementary material for: The MUC5B-associated variant rs35705950 resides within an enhancer subject to lineage- and disease-dependent epigenetic remodeling
Source: JCI Insight. 2021 Jan 25;6(2):e144294. doi: 10.1172/jci.insight.144294 (PMC7934873; doi:10.1172/jci.insight.144294)
Supplement: Supplemental Data Set 3 [file jciinsight-6-144294-s078.zip › Supplemental File S3_PRO-seq Pipeline & QC Reports/PRO-seq_Nextflow_pipeline_report/pipeline_report.html]

NascentFlow Pipeline Report


# NascentFlow v1.2

## Run Name: romantic\_lichterman

NascentFlow execution completed successfully!

The workflow was completed at **Thu Oct 24 14:33:29 MDT 2019** (duration: **23h 15m 54s**)

The command used to launch the workflow was as follows:

```
nextflow run /Users/magr0763/Nascent-Flow/main.nf -profile hg38 --fastqs '/scratch/Shares/dowell/Sasse/Unstimulated_PROseq/fastq/*.fastq.gz' --workdir /scratch/Shares/dowell/Sasse/Unstimulated_PROseq/temp --email margaret.gruca@colorado.edu --outdir /scratch/Shares/dowell/Sasse/Unstimulated_PROseq --genome_id hg38 --singleEnd --flip --savebg --savebw --dastk --tfit --fstitch --counts
```

### Pipeline Configuration:

|  |  |
| --- | --- |
| Pipeline Name | ``` NascentFlow ``` |
| Help Message | ``` false ``` |
| Pipeline Version | ``` 1.2 ``` |
| Run Name | ``` romantic_lichterman ``` |
| Reads | ``` data/*_{1,2}.fastq.gz ``` |
| Fastqs | ``` /scratch/Shares/dowell/Sasse/Unstimulated_PROseq/fastq/*.fastq.gz ``` |
| Genome Ref | ``` /scratch/Shares/dowell/genomes/hg38/hg38.fa ``` |
| Thread fqdump | ``` NO ``` |
| Data Type | ``` Single-End ``` |
| Save All fastq | ``` NO ``` |
| Save BAM | ``` NO ``` |
| Save BigWig | ``` YES ``` |
| Save bedGraph | ``` YES ``` |
| Save fastq | ``` NO ``` |
| Save Trimmed | ``` NO ``` |
| Reverse Comp | ``` YES ``` |
| Run Multicov | ``` YES ``` |
| Nascent QC | ``` NO ``` |
| Run FastQC | ``` YES ``` |
| Run preseq | ``` YES ``` |
| Run pileup | ``` YES ``` |
| Run RSeQC | ``` YES ``` |
| Run MultiQC | ``` YES ``` |
| Skip All QC | ``` NO ``` |
| Max Memory | ``` 20 GB ``` |
| Max CPUs | ``` 1 ``` |
| Max Time | ``` 3d 4h ``` |
| Output dir | ``` /scratch/Shares/dowell/Sasse/Unstimulated_PROseq ``` |
| FStitch | ``` YES ``` |
| Prelim Tfit | ``` NO ``` |
| Tfit | ``` YES ``` |
| DAStk | ``` YES ``` |
| dREG | ``` NO ``` |
| FStitch dir | ``` /scratch/Shares/dowell/FStitch/src/FStitch ``` |
| FStitch train | ``` /scratch/Shares/dowell/Sasse/untreated_proseq_train.bed ``` |
| Tfit dir | ``` /scratch/Shares/dowell/Tfit/src/Tfit ``` |
| Working dir | ``` /scratch/Shares/dowell/Sasse/Unstimulated_PROseq/temp ``` |
| Container Engine | ``` null ``` |
| Current home | ``` /Users/magr0763 ``` |
| Current user | ``` magr0763 ``` |
| Current path | ``` /scratch/Shares/dowell/Sasse ``` |
| Script dir | ``` /Users/magr0763/Nascent-Flow ``` |
| Config Profile | ``` hg38 ``` |
| E-mail Address | ``` margaret.gruca@colorado.edu ``` |
| Date Started | ``` Wed Oct 23 15:17:35 MDT 2019 ``` |
| Date Completed | ``` Thu Oct 24 14:33:29 MDT 2019 ``` |
| Pipeline script file path | ``` /Users/magr0763/Nascent-Flow/main.nf ``` |
| Pipeline script hash ID | ``` 6af6ada925af93c15815d311d1a63f88 ``` |
| Nextflow Version | ``` 19.04.1 ``` |
| Nextflow Build | ``` 5072 ``` |
| Nextflow Compile Timestamp | ``` 03-05-2019 12:29 UTC ``` |

NascentFlow

https://biof-git.colorado.edu/dowelllab/GRO-seq-workflow

https://github.com/Dowell-Lab/Nascent-Flow
