# Supplementary material for: The MUC5B-associated variant rs35705950 resides within an enhancer subject to lineage- and disease-dependent epigenetic remodeling
Source: JCI Insight. 2021 Jan 25;6(2):e144294. doi: 10.1172/jci.insight.144294 (PMC7934873; doi:10.1172/jci.insight.144294)
Supplement: Supplemental Data Set 4 [file jciinsight-6-144294-s079.zip › Supplemental File S4_ATAC-seq Pipeline & QC Reports/ATAC-seq_Nextflow_pipeline_reports/BronchBrush_ATAC-seq_Nextflow_pipeline_report/chip_pipeline_report.html]

[cheesy\_celsius] Nextflow Workflow Report


Nextflow Report


- Summary
- Resources
- Tasks

[cheesy\_celsius]

# Nextflow workflow report

## `[cheesy_celsius]`

Workflow execution completed successfully!

Run times
:   Tue Nov 19 18:46:49 MST 2019 - Tue Nov 19 22:01:11 MST 2019
    (duration: **3h 14m 22s**)

22 succeeded

0 cached

0 ignored

0 failed

Nextflow command
:   ```
    nextflow run /Users/magr0763/ChIP-Flow/main.nf -profile fiji --fastqs '/scratch/Shares/dowell/Sasse/bronch_brush/ATAC-seq/fastq/*{1,2}.fastq.gz' --workdir /scratch/Shares/dowell/Sasse/bronch_brush/ATAC-seq/temp --email margaret.gruca@colorado.edu --outdir /scratch/Shares/dowell/Sasse/bronch_brush/ATAC-seq --dedup --savedup
    ```

CPU-Hours
:   `78.7`

Launch directory
:   `/scratch/Shares/dowell/Sasse/bronch_brush/ATAC-seq/fastq`

Work directory
:   `/scratch/Shares/dowell/Sasse/bronch_brush/ATAC-seq/temp`

Project directory
:   `/Users/magr0763/ChIP-Flow`

Script name
:   `main.nf`

Script ID
:   `7b9d38a1b8f7ef0415c764b3aec6985d`

Workflow session
:   `140cbd13-4108-4467-91a7-518b43cfdfdb`

Workflow profile
:   fiji

Workflow container
:   `skptic/chipflow:latest`

Container engine
:   `-`

Nextflow version
:   version 19.04.1, build 5072 (03-05-2019 12:29 UTC)

## Resource Usage

These plots give an overview of the distribution of resource usage for each process.

#### CPU

- Raw Usage
- % Allocated

#### Memory

- Physical (RAM)
- Virtual (RAM + Disk swap)
- % RAM Allocated

#### Job Duration

- Raw Usage
- % Allocated

#### I/O

- Read
- Write

## Tasks

This table shows information about each task in the workflow. Use the search box on the right
to filter rows for specific values. Clicking headers will sort the table by that value and
scrolling side to side will reveal more columns.

Values shown as:

Human readable
Raw values

(tasks table omitted because the dataset is too big)

Generated by Nextflow, version 19.04.1
