# Supplementary material for: The MUC5B-associated variant rs35705950 resides within an enhancer subject to lineage- and disease-dependent epigenetic remodeling
Source: JCI Insight. 2021 Jan 25;6(2):e144294. doi: 10.1172/jci.insight.144294 (PMC7934873; doi:10.1172/jci.insight.144294)
Supplement: Supplemental Data Set 4 [file jciinsight-6-144294-s079.zip › Supplemental File S4_ATAC-seq Pipeline & QC Reports/ATAC-seq_Nextflow_pipeline_reports/BronchBrush_ATAC-seq_Nextflow_pipeline_report/pipeline_report.html]

ChIP\_Flow Pipeline Report


# ChIPFlow v1.0.1

## Run Name: cheesy\_celsius

ChIP-Flow execution completed successfully!

The workflow was completed at **Tue Nov 19 22:01:11 MST 2019** (duration: **3h 14m 22s**)

The command used to launch the workflow was as follows:

```
nextflow run /Users/magr0763/ChIP-Flow/main.nf -profile fiji --fastqs '/scratch/Shares/dowell/Sasse/bronch_brush/ATAC-seq/fastq/*{1,2}.fastq.gz' --workdir /scratch/Shares/dowell/Sasse/bronch_brush/ATAC-seq/temp --email margaret.gruca@colorado.edu --outdir /scratch/Shares/dowell/Sasse/bronch_brush/ATAC-seq --dedup --savedup
```

### Pipeline Configuration:

|  |  |
| --- | --- |
| Pipeline Name | ``` ChIPFlow ``` |
| Help Message | ``` false ``` |
| Pipeline Version | ``` 1.0.1 ``` |
| Run Name | ``` cheesy_celsius ``` |
| Reads | ``` data/*{R1,R2}*.fastq ``` |
| Fastqs | ``` /scratch/Shares/dowell/Sasse/bronch_brush/ATAC-seq/fastq/*{1,2}.fastq.gz ``` |
| SRAs | ``` false ``` |
| Genome Ref | ``` /scratch/Shares/dowell/genomes/hg38/hg38.fa ``` |
| Thread fqdump | ``` NO ``` |
| Data Type | ``` Paired-End ``` |
| Save All fastq | ``` NO ``` |
| Save fastq | ``` NO ``` |
| Save Trimmed | ``` NO ``` |
| Save Dup | ``` YES ``` |
| Remove Dup | ``` YES ``` |
| Run FastQC | ``` YES ``` |
| Run preseq | ``` YES ``` |
| Run pileup | ``` YES ``` |
| Run RSeQC | ``` YES ``` |
| Run MultiQC | ``` YES ``` |
| Skip All QC | ``` NO ``` |
| Max Memory | ``` 20 GB ``` |
| Max CPUs | ``` 1 ``` |
| Max Time | ``` 2d ``` |
| Output dir | ``` /scratch/Shares/dowell/Sasse/bronch_brush/ATAC-seq ``` |
| Working dir | ``` /scratch/Shares/dowell/Sasse/bronch_brush/ATAC-seq/temp ``` |
| Container Engine | ``` null ``` |
| Current home | ``` /Users/magr0763 ``` |
| Current user | ``` magr0763 ``` |
| Current path | ``` /scratch/Shares/dowell/Sasse/bronch_brush/ATAC-seq/fastq ``` |
| Script dir | ``` /Users/magr0763/ChIP-Flow ``` |
| Config Profile | ``` fiji ``` |
| E-mail Address | ``` margaret.gruca@colorado.edu ``` |
| Date Started | ``` Tue Nov 19 18:46:49 MST 2019 ``` |
| Date Completed | ``` Tue Nov 19 22:01:11 MST 2019 ``` |
| Pipeline script file path | ``` /Users/magr0763/ChIP-Flow/main.nf ``` |
| Pipeline script hash ID | ``` 7b9d38a1b8f7ef0415c764b3aec6985d ``` |
| Nextflow Version | ``` 19.04.1 ``` |
| Nextflow Build | ``` 5072 ``` |
| Nextflow Compile Timestamp | ``` 03-05-2019 12:29 UTC ``` |

NascentFlow

https://github.com/Dowell-Lab/ChIP-Flow
